# PyPhi: A toolbox for integrated information theory

**DOI:** 10.1371/journal.pcbi.1006343

**Published:** 2018-07-26

**Authors:** William G. P. Mayner, William Marshall, Larissa Albantakis, Graham Findlay, Robert Marchman, Giulio Tononi

**Affiliations:** 1 Neuroscience Training Program, University of Wisconsin–Madison, Madison, Wisconsin, United States of America; 2 Psychiatry Department, University of Wisconsin–Madison, Madison, Wisconsin, United States of America; George Mason University, UNITED STATES

## Abstract

Integrated information theory provides a mathematical framework to fully characterize the cause-effect structure of a physical system. Here, we introduce *PyPhi*, a Python software package that implements this framework for causal analysis and unfolds the full cause-effect structure of discrete dynamical systems of binary elements. The software allows users to easily study these structures, serves as an up-to-date reference implementation of the formalisms of integrated information theory, and has been applied in research on complexity, emergence, and certain biological questions. We first provide an overview of the main algorithm and demonstrate PyPhi’s functionality in the course of analyzing an example system, and then describe details of the algorithm’s design and implementation. PyPhi can be installed with Python’s package manager via the command ‘pip install pyphi’ on Linux and macOS systems equipped with Python 3.4 or higher. PyPhi is open-source and licensed under the GPLv3; the source code is hosted on GitHub at https://github.com/wmayner/pyphi. Comprehensive and continually-updated documentation is available at https://pyphi.readthedocs.io. The pyphi-users mailing list can be joined at https://groups.google.com/forum/#!forum/pyphi-users. A web-based graphical interface to the software is available at http://integratedinformationtheory.org/calculate.html.

This is a *PLOS Computational Biology* Software paper.

## Introduction

Integrated information theory (IIT) has been proposed as a theory of consciousness. The central hypothesis is that a physical system has to meet five requirements (‘postulates’) in order to be a physical substrate of subjective experience: (1) *intrinsic existence* (the system must be able to make a difference to itself); (2) *composition* (it must be composed of parts that have causal power within the whole); (3) *information* (its causal power must be specific); (4) *integration* (its causal power must not be reducible to that of its parts); and (5) *exclusion* (it must be maximally irreducible) [1–5].

From these postulates, IIT develops a mathematical framework to assess the cause-effect structure (CES) of a physical system that is applicable to discrete dynamical systems. This framework has proven useful not only for the study of consciousness but has also been applied in research on complexity [6–9], emergence [10–12], and certain biological questions [13].

The main measure of cause-effect power, *integrated information* (denoted Φ), quantifies how irreducible a system’s CES is to those of its parts. Φ also serves as a general measure of complexity that captures to what extent a system is both integrated [6] and differentiated (informative) [14].

Here we describe *PyPhi*, a Python software package that implements IIT’s framework for causal analysis and unfolds the full CES of discrete Markovian dynamical systems of binary elements. The software allows users to easily study these CESs and serves as an up-to-date reference implementation of the formalisms of IIT.

Details of the mathematical framework are published elsewhere [1, 3]; in § Results we describe the output and input of the software and give an overview of the main algorithm in the course of reproducing results obtained from an example system studied in [3]. In § Design and implementation we discuss specific issues concerning the algorithm’s implementation. Finally in § Availability and future directions we describe how the software can be obtained and discuss new functionality planned for future versions.

## Results

### Output

**Fig 1:**
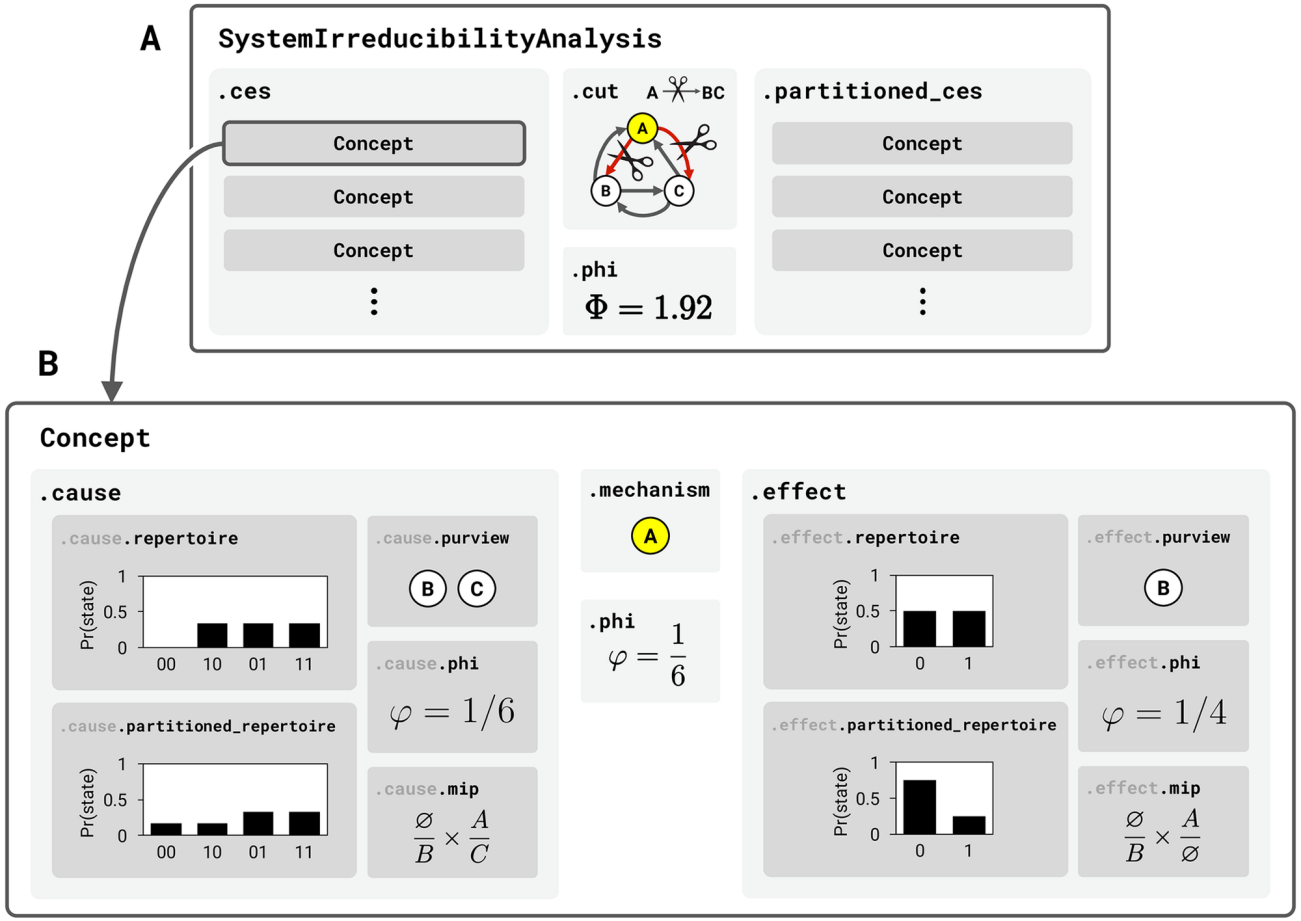
Output. **(A)** The SystemIrreducibilityAnalysis object is the main output of the software. It represents the results of the analysis of the system in question. It has several attributes (grey boxes): ‘ces’ is a CauseEffectStructure object containing all of the system’s Concepts; ‘cut’ is a Cut object that represents the minimum-information partition (MIP) of the system (the partition of the system that makes the least difference to its CES); ‘partitioned_ces’ is the CauseEffectStructure of Concepts specified by the system after applying the MIP; and ‘phi’ is the Φ value, which measures the difference between the unpartitioned and partitioned CES. **(B)** A Concept represents the maximally-irreducible cause (MIC) and maximally-irreducible effect (MIE) of a mechanism in a state. The ‘mechanism’ attribute contains the indices of the mechanism elements. The ‘cause’ and ‘effect’ attributes contain MaximallyIrreducibleCause and MaximallyIrreducibleEffect objects that describe the mechanism’s MIC and MIE, respectively; each of these contains a purview, repertoire, MIP, partitioned repertoire, and *φ* value. The ‘phi’ attribute contains the concept’s *φ* value, which is the minimum of the *φ* values of the MIC and MIE.

The software has two primary functions: (1) to unfold the full CES of a discrete dynamical system of interacting elements and compute its Φ value, and (2) to compute the maximally-irreducible cause-effect repertoires of a particular set of elements within the system. The first is function is implemented by pyphi.compute.major_complex(), which returns a SystemIrreducibilityAnalysis object (Fig 1A). The system’s CES is contained in the ‘ces’ attribute and its Φ value is contained in ‘phi’. Other attributes are detailed in the online documentation.

The CES is composed of Concept objects, which are the output of the second main function: Subsystem.concept() (Fig 1B). Each Concept is specified by a set of elements within the system (contained in its ‘mechanism’ attribute). A Concept contains a maximally-irreducible cause and effect repertoire (‘cause_repertoire’ and ‘effect_repertoire’), which are probability distributions that capture how the mechanism elements in their current state constrain the previous and next state of the system, respectively; a *φ* value (‘phi’), which measures the irreducibility of the repertoires; and several other attributes discussed below and detailed in the online documentation.

### Input

**Fig 2:**
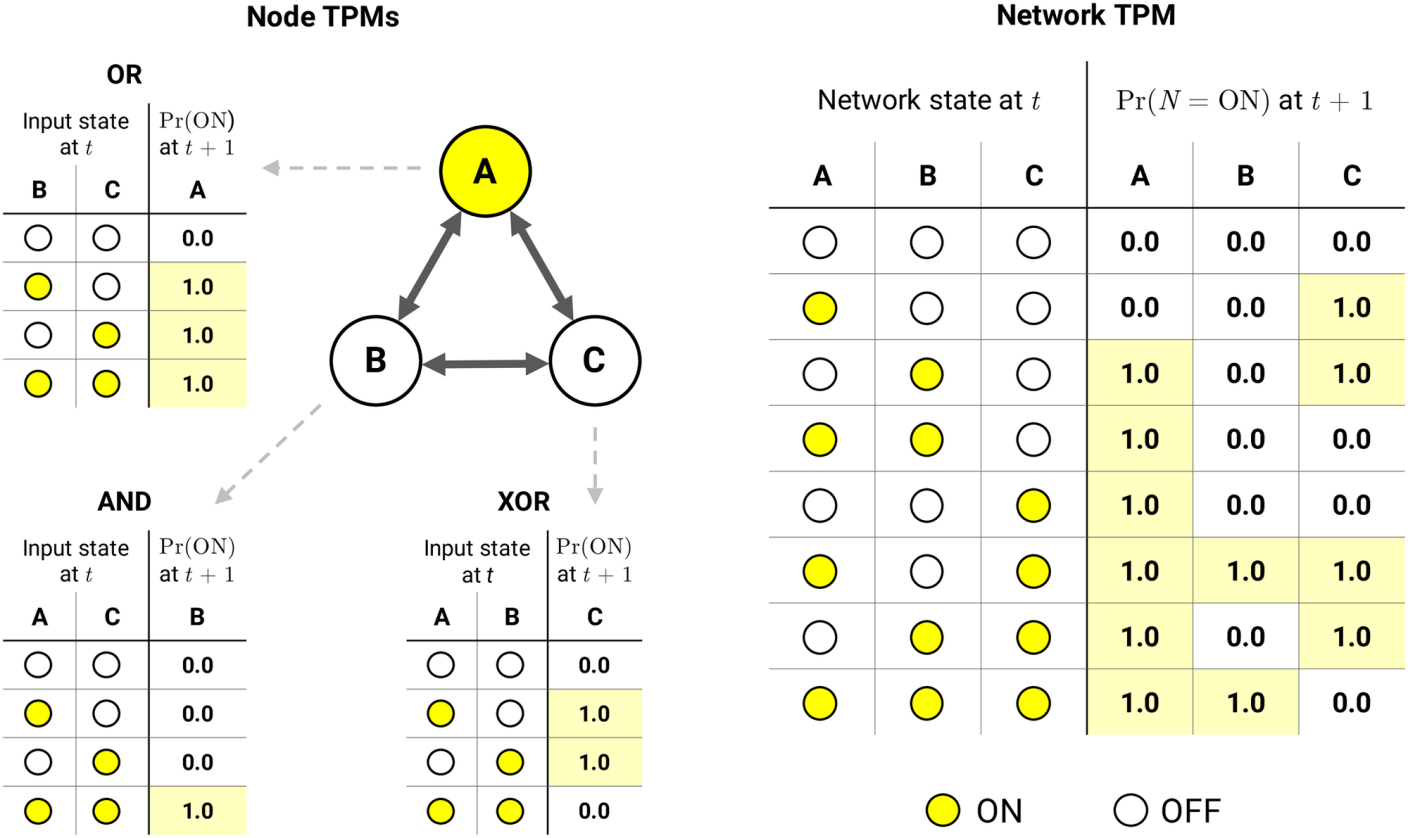
A network of nodes and its TPM. Each node has its own TPM—in this case, the truth-table of a deterministic logic gate. Yellow signifies the “ON” state; white signifies “OFF”. The system’s TPM (right) is composed of the TPMs of its nodes (left), here shown in state-by-node form (see § Representation of the TPM and probability distributions). Note that in PyPhi’s TPM representation, the first node’s state varies the fastest, according to the little-endian convention (see § 2-dimensional state-by-node form).

The starting point for the IIT analysis is a discrete Markovian dynamical system *S* composed of *n* interacting elements. Such a system can be represented by a directed graph of interconnected nodes, each equipped with a function that outputs the node’s state at the next timestep *t* + 1 given the state of its parents at the previous timestep *t* (Fig 2). At present, PyPhi can analyze both deterministic and stochastic discrete Markovian dynamical systems consisting of elements with two states.

Such a discrete dynamical system is completely specified by its transition probability matrix (TPM), which contains the probabilities of all state transitions from *t* to *t* + 1. It can be obtained from the graphical representation of the system by perturbing the system into each of its possible states and observing the following state at the next timestep (for stochastic systems, repeated trials of perturbation/observation will yield the probabilities of each state transition). In PyPhi, the TPM is the fundamental representation of the system.

Formally, if we let *S*_*t*_ be the random variable of the system state at *t*, the TPM specifies the conditional probability distribution over the next state *S*_*t*+1_ given each current state *s*_*t*_:

$$\begin{array}{c} \text{Pr}\left(S_{t+1}|S_{t}=s_{t}\right),\;\forall \;s_{t}\in \Omega_{S}, \end{array}$$

where Ω_*S*_ denotes the set of possible states. Furthermore, given a marginal distribution over the previous states of the system, the TPM fully specifies the joint distribution over state transitions. Here IIT imposes uniformity on the marginal distribution of the previous states because the aim of the analysis is to capture direct causal relationships across a single timestep without confounding factors, such as influences from system states before *t* − 1 [3, 4, 11, 15, 16]. The marginal distribution thus corresponds to an interventional (causal), not observed, state distribution.

Moreover, IIT assumes that there is no instantaneous causation; that is, it is assumed that the elements of the dynamical system influence one another only from one timestep to the next. Therefore we require that the system satisfies the following Markov condition, called the *conditional independence property*: each element’s state at *t* + 1 must be independent of the state of the others, given the state of the system at *t* [17],

$$\begin{array}{c} \text{Pr}\left(S_{t+1}|S_{t}=s_{t}\right)\;=\prod_{N\;\in \;S}\text{Pr}\left(N_{t+1}|S_{t}=s_{t}\right)\;,\;\forall \;s_{t}\in S. \end{array}$$ (1)

For systems of binary elements, a TPM that satisfies Eq (1) can be represented in state-by-node form (Fig 2, right), since we need only store each element’s marginal distribution rather than the full joint distribution.

In PyPhi, the system under analysis is represented by a Network object. A Network is created by passing its TPM as the first argument: network = pyphi.Network(tpm) (see § Setup). Optionally, a connectivity matrix (CM) can also be provided, where

$$\begin{array}{c} {\left[\text{CM}\right]}_{i,j}=\{\begin{array}{cc} 1 & \text{if}\;\text{there}\;\text{is}\;\text{an}\;\text{edge}\;\text{from}\;\text{element}\;i\;\text{to}\;\text{element}\;j\\ 0 & \text{otherwise}, \end{array} \end{array}$$

via the cm keyword argument: network = pyphi.Network(tpm, cm=cm). Because the TPM completely specifies the system, providing a CM is not necessary; however, explicit connectivity information can be used to make computations more efficient, especially for sparse networks, because PyPhi can rule out certain causal influences *a priori* if there are missing connections (see § Connectivity optimizations). Note that this means providing an incorrect CM can result in inaccurate output. If no CM is given, PyPhi assumes full connectivity; *i.e*., it assumes each element may have an effect on any other, which guarantees correct results.

Once the Network is created, a subset of elements within the system (called a *candidate system*), together with a particular system state, can be selected for analysis by creating a Subsystem object. Hereafter we refer to a candidate system as a *subsystem*.

### Demonstration

The mathematical framework of IIT is typically formulated using graphical causal models as representations of physical systems of elements. The framework builds on the causal calculus of the *do*(⋅) operator introduced by Pearl [17]. In order to assess causal relationships among the elements, interventions (manipulations, perturbations) are used to actively set elements into a specific state, after which the resulting state transition is observed.

For reference, we define a set of graphical operations that are used during the IIT analysis. To *fix* an element is to use system interventions to keep it in the same state for every observation. To *noise* an element is to use system interventions to set it into a state chosen uniformly at random. Finally, to *cut* a connection from a source element to a target element is to make the source appear noised to the target, while the remaining, uncut connections from the source still correctly transmit its state.

**Fig 3:**
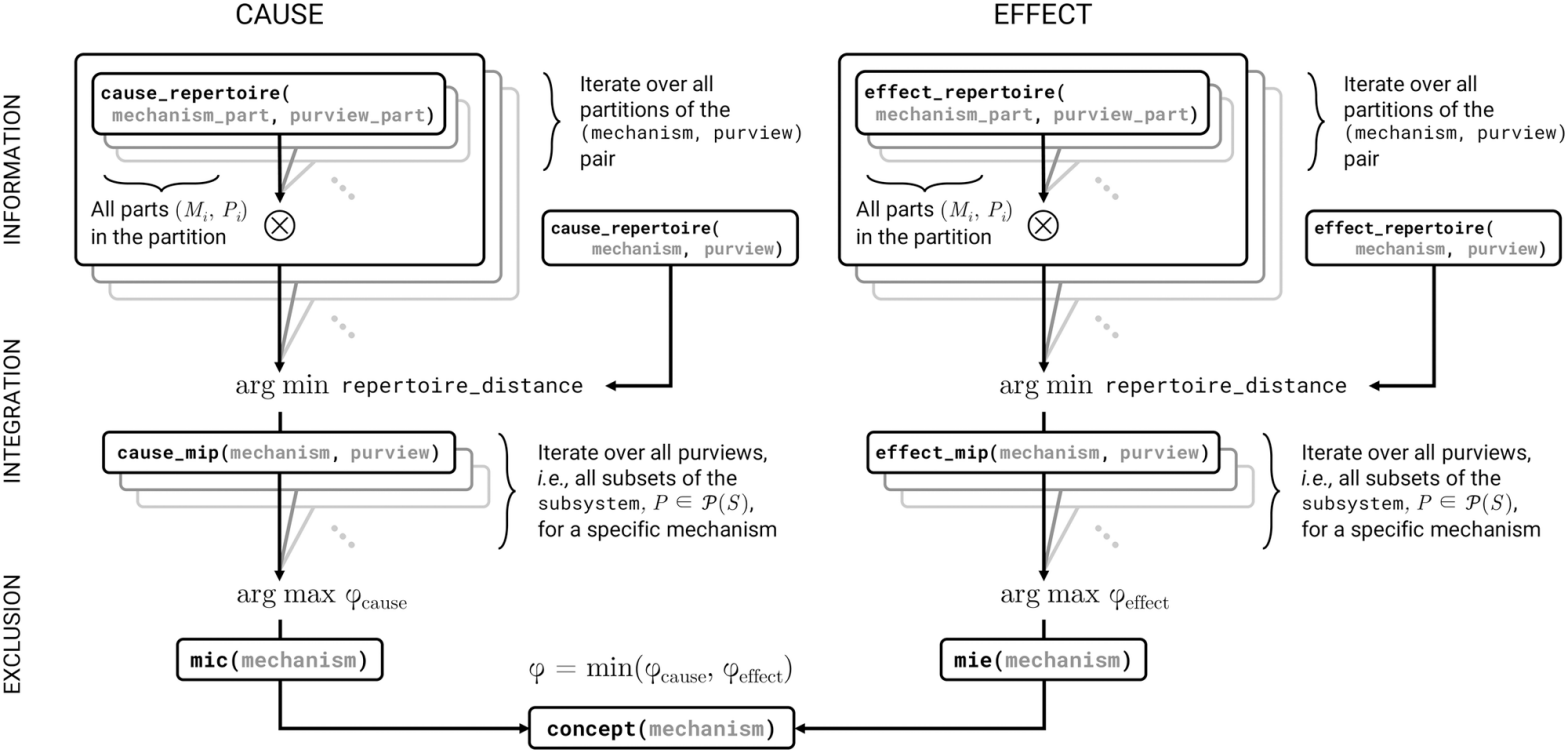
Algorithm schematic at the mechanism level. PyPhi functions are named in boxes, with arguments in grey. Arrows point from callee to caller. Functions are organized by the postulate they correspond to (left). ⊗ denotes the tensor product; P denotes the power set.

**Fig 4:**
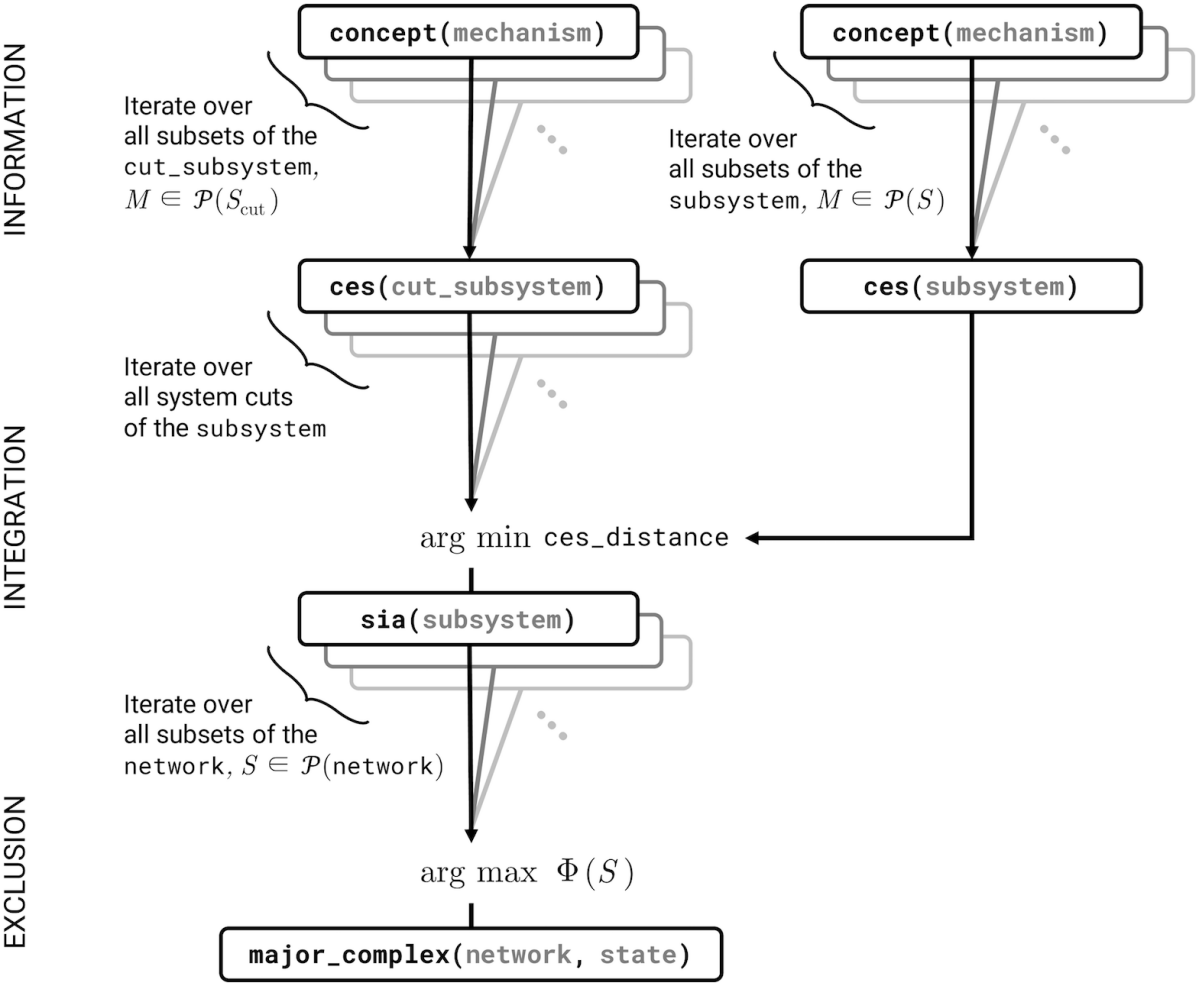
Algorithm schematic at the system level. PyPhi functions are named in boxes, with arguments in grey. Arrows point from callee to caller. Functions are organized by the postulate they correspond to (left). P denotes the power set.

In this section we demonstrate some of the capabilities of the software by unfolding the CES of a small deterministic system of logic gates as described in [3] while describing how the algorithm is implemented in terms of TPM manipulations, which we link to the graphical operations defined above. A schematic of the algorithm is shown in Figs 3 and 4, and a more detailed illustration is given in S1 Text.

#### Setup

The first step is to create the Network object. Here we choose to provide the TPM in 2-dimensional state-by-node form (see § 2-dimensional state-by-node form). The TPM is the only required argument, but we provide the CM as well, since we know that there are no self-loops in the system and PyPhi will use this information to speed up the computation. We also label the nodes *A*, *B*, and *C* to make the output easier to read.

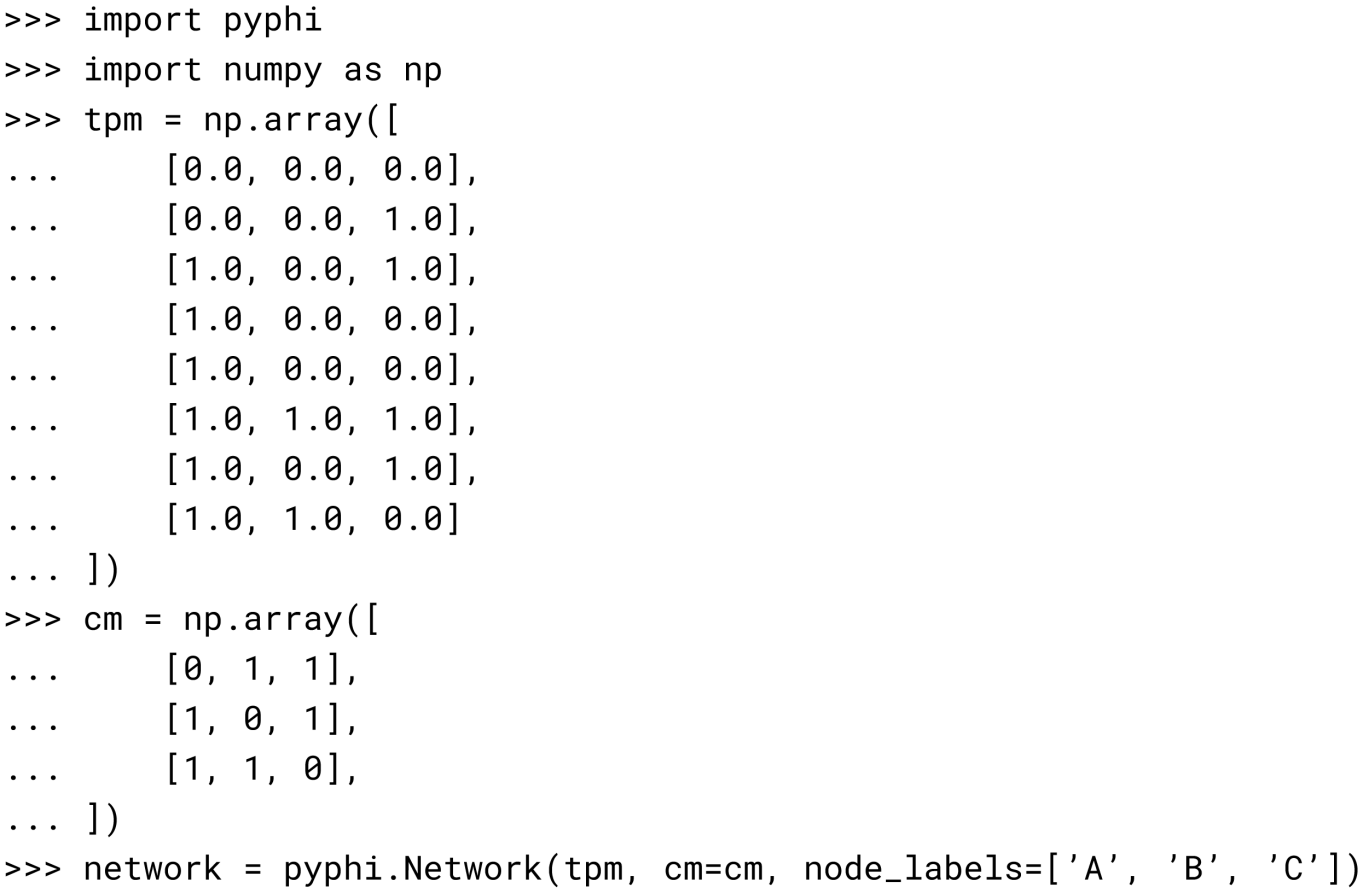


We select a subsystem and a system state for analysis by creating a Subsystem object. System states are represented by tuples of 1s and 0s, with 1 meaning “ON” and 0 meaning “OFF.” In this case we will analyze the entire system, so the subsystem will contain all three nodes. The nodes to include can be specified with either their labels or their indices (note that in other PyPhi functions, nodes must be specified with their indices).

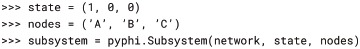


If there are nodes outside the subsystem, they are considered as *background conditions* for the causal analysis [3]. In the graphical representation of the system, the background conditions are *fixed* in their current state while the subsystem is perturbed and observed in order to derive its TPM. In the TPM representation, the equivalent operation is performed by *conditioning* the system TPM on the state at *t* of the nodes outside the subsystem and then *marginalizing out* those nodes at *t* + 1 (illustrated in S1 Text). In PyPhi, this is done when the subsystem is created; the subsystem TPM can be accessed with the tpm attribute, *e.g*. subsystem.tpm.

#### Cause/Effect repertoires (mechanism-level information)

The lowest-level objects in the CES of a system are the *cause repertoire* and *effect repertoire* of a set of nodes within the subsystem, called a *mechanism*, over another set of nodes within the subsystem, called a *purview* of the mechanism. The cause (effect) repertoire is a probability distribution that captures the information specified by the mechanism about the purview by describing how the previous (next) state of the purview is constrained by the current state of the mechanism.

In terms of graphical operations, the effect repertoire is obtained by (1) *fixing* the mechanism nodes in their state at *t*; (2) *noising* the non-mechanism nodes at time *t*, so as to remove their causal influence on the purview; and (3) observing the resulting state transition from *t* to *t* + 1 while ignoring the state at *t* + 1 of non-purview nodes, in order to derive a distribution over purview states at *t* + 1.

The cause repertoire is obtained similarly, but in that case, the purview nodes at time *t* − 1 are noised, and the resulting state transition from *t* − 1 to *t* is observed while ignoring the state of non-mechanism nodes. Bayes’ rule is then applied, resulting in a distribution over purview states at *t* − 1. The corresponding operations on the TPM are detailed in § Calculation of cause/effect repertoires from the TPM and illustrated in S1 Text.

Note that, operationally, we enforce that each input from a noised node conveys *independent* noise during the perturbation/observation step. In this way, we avoid counting correlations from outside the mechanism-purview pair as constraints due to the current state of the mechanism. Graphically, this process would correspond to replacing each noised node that is a parent of multiple purview nodes (for the effect repertoire) or mechanism nodes (for the cause repertoire) with multiple, independent “virtual nodes” (as in [3, Supplementary Methods]). However, the equivalent definition of repertoires in Eqs (3) and (5) obviates the need to actually implement virtual nodes in PyPhi.

With the cause_repertoire() method of the Subsystem, we can obtain the cause repertoire of, for example, mechanism *A* over the purview *ABC* depicted in Fig. 4 of [3]:

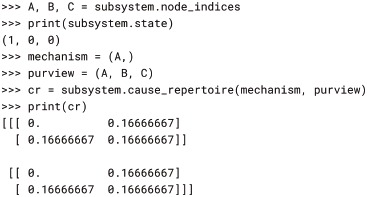


We see that mechanism *A* in its current state, ON (1), specifies information by ruling out the previous states in which *B* and *C* are OFF (0). That is, the probability that either (0, 0, 0) or (1, 0, 0) was the previous state, given that *A* is currently ON, is zero:

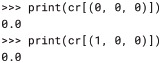


Note that repertoires are returned in multidimensional form, so they can be indexed with state tuples as above. Repertoires can be reshaped to be 1-dimensional if needed, *e.g*. for plotting, but care must be taken that NumPy’s FORTRAN (column-major) ordering is used so that PyPhi’s little-endian convention for indexing states is respected (see § 2-dimensional state-by-node form). PyPhi provides the pyphi.distribution.flatten() function for this:

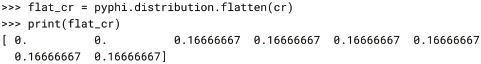


#### Minimum-information partitions (mechanism-level integration)

Having assessed the information of a mechanism over a purview, the next step is to assess its *integrated information* (denoted *φ*) by quantifying the extent to which the cause and effect repertoires of the mechanism-purview pair cannot be reduced to the repertoires of its parts.

In terms of graphical operations, the irreducibility of a mechanism-purview pair is tested by partitioning it into parts and *cutting* the connections between them. By applying the perturbation/observation procedure after cutting the connections we obtain a *partitioned repertoire*. Since the partition renders the parts independent, in terms of TPM manipulations, the partitioned repertoire can be calculated as the product of the individual repertoires for each of the parts. If the partitioned repertoire is no different than the original unpartitioned repertoire, then the mechanism as a whole did not specify integrated information about the purview. By contrast, if a repertoire cannot be factored in this way, then some of its selectivity is due to the causal influence of the mechanism *as an integrated whole* on the purview, and the repertoire is said to be *irreducible*.

The amount of irreducibility of a mechanism over a purview with respect to a partition is quantified as the distance between the unpartitioned repertoire and the partitioned repertoire (calculated with pyphi.distance.repertoire_distance()). There are many ways to divide the mechanism and purview into parts, so the irreducibility is measured for every partition and the partition that yields the minimum irreducibility is called the *minimum-information partition* (MIP). The integrated information (*φ*) of a mechanism-purview pair is the distance between the unpartitioned repertoire and the partitioned repertoire associated with the MIP. PyPhi supports several distance measures and partitioning schemes (see § Configuration).

The MIP search procedure is implemented by the Subsystem.cause_mip() and Subsystem.effect_mip() methods. Each returns a RepertoireIrreducibilityAnalysis object that contains the MIP, as well as the *φ* value, mechanism, purview, temporal direction (cause or effect), unpartitioned repertoire, and partitioned repertoire. For example, we compute the effect MIP of mechanism *ABC* over purview *ABC* from Fig. 6 of [3] as follows:

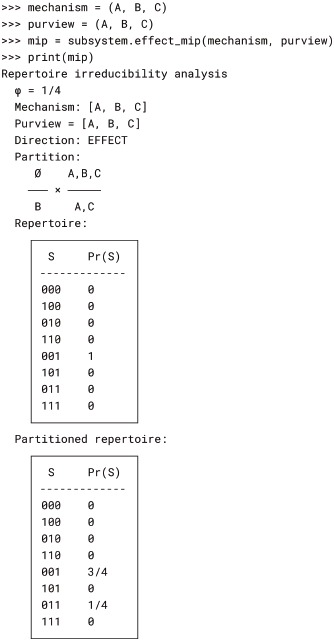


Here we can see that the MIP attempts to factor the repertoire of *ABC* over *ABC* into the product of the repertoire of *ABC* over *AC* and the repertoire of the empty mechanism ∅ over *B*. However, the repertoire cannot be factored in this way without information loss; the distance between the unpartitioned and partitioned repertoire is nonzero ($\varphi =\frac{1}{4}$). Thus mechanism *ABC* over the purview *ABC* is irreducible.

#### Maximally-irreducible cause-effect repertoires (mechanism-level exclusion)

Next, we apply IIT’s postulate of exclusion at the mechanism level by finding the *maximally-irreducible cause* (MIC) and *maximally irreducible effect* (MIE) specified by a mechanism. This is done by searching over all possible purviews for the RepertoireIrreducibilityAnalysis object with the maximal *φ* value. The Subsystem.mic() and Subsystem.mie() methods implement this search procedure; they return a MaximallyIrreducibleCause and a MaximallyIrreducibleEffect object, respectively. The MIC of mechanism *BC*, for example, is the purview *AB* (Fig. 8 of [3]). This is computed like so:

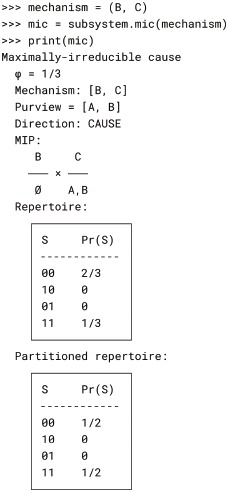


#### Concepts

If the mechanism’s MIC has *φ*_cause_ > 0 and its MIE has *φ*_effect_ > 0, then the mechanism is said to specify a *concept*. The *φ* value of the concept as a whole is the minimum of *φ*_cause_ and *φ*_effect_.

We can compute the concept depicted in Fig. 9 of [3] using the Subsystem.concept() method, which takes a mechanism and returns a Concept object containing the *φ* value, the MIC (in the ‘cause’ attribute), and the MIE (in the ‘effect’ attribute):

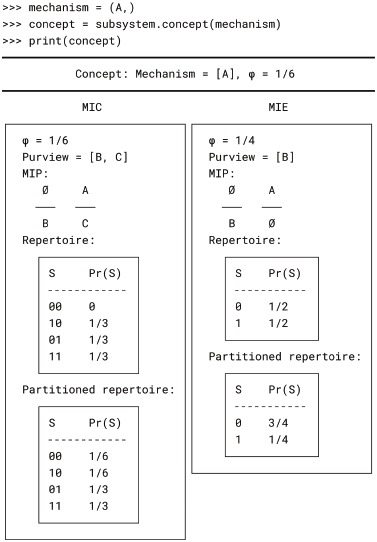


Note that in PyPhi, the repertoires are distributions over purview states, rather than system states. Occasionally it is more convenient to represent repertoires as distributions over the entire system. This can be done with the expand_cause_repertoire() and expand_effect_repertoire() methods of the Concept object, which assume the unconstrained (maximum-entropy) distribution over the states of non-purview nodes:

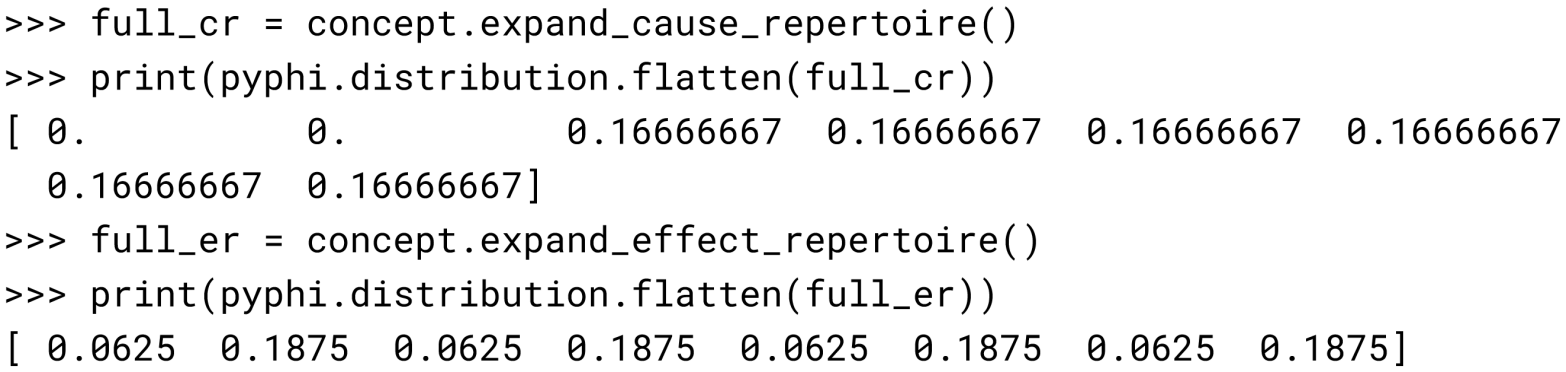


Also note that Subsystem.concept() will return a Concept object when *φ* = 0 even though these are not concepts, strictly speaking. For convenience, bool(concept) evaluates to True if *φ* > 0 and False otherwise.

#### Cause-effect structures (system-level information)

The next step is to compute the CES, the set of all concepts specified by the subsystem. The CES characterizes all of the causal constraints that are intrinsic to a physical system. This is implemented by the pyphi.compute.ces() function, which simply calls Subsystem.concept() for every mechanism M∈P(S), where P(S) is the power set of subsystem nodes. It returns a CauseEffectStructure object containing those Concepts for which *φ* > 0.

We see that every mechanism in P(S) except for *AC* specifies a concept, as described in Fig. 10 of [3]:

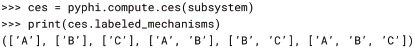


#### Irreducible cause-effect structures (system-level integration)

At this point, the irreducibility of the subsystem’s CES is evaluated by applying the integration postulate at the system level. As with integration at the mechanism level, the idea is to measure the difference made by each partition and then take the minimal value as the irreducibility of the subsystem.

We begin by performing a *system cut*. Graphically, the subsystem is partitioned into two parts and the edges going from one part to the other are *cut*, rendering them causally ineffective. This is implemented as an operation on the TPM as follows: Let *E*_cut_ denote the set of directed edges in the subsystem that are to be cut, where each edge *e* ∈ *E*_cut_ has a source node *a* and a target node *b*. For each edge, we modify the individual TPM of node *b* (Fig 2) by marginalizing over the states of *a* at *t*. The resulting TPM specifies the function implemented by *b* with the causal influence of *a* removed. We then combine the modified node TPMs to recover the full TPM of the partitioned subsystem. Finally, we recalculate the CES of the subsystem with this modified TPM (the *partitioned CES*).

The irreducibility of a CES with respect to a partition is the distance between the unpartitioned and partitioned CESs (calculated with pyphi.compute.ces_distance(); several distances are supported; see § Configuration). This distance is evaluated for every partition, and the minimum value across all partitions is the subsystem’s integrated information Φ, which measures the extent to which the CES specified by the subsystem is irreducible to the CES under the minimal partition.

This procedure is implemented by the pyphi.compute.sia() function, which returns a SystemIrreducibilityAnalysis object (Fig 1). We can verify that the Φ value of the example system in [3] is 1.92 and the minimal partition is that which removes the causal connections from *AB* to *C*:

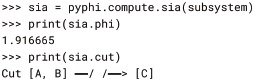


#### Complexes (system-level exclusion)

The final step in unfolding the CES of the system is to apply the postulate of exclusion at the system level. We compute the CES of each subset of the network, considered as a subsystem (that is, *fixing* the external nodes as background conditions), and find the CES with maximal Φ, called the *maximally-irreducible cause-effect structure* (MICS) of the system. The subsystem giving rise to it is called the *major complex*; any overlapping subsets with lower Φ are excluded. Non-overlapping subsets may be further analyzed to find additional complexes within the system.

In this example, we find that the whole system *ABC* is the system’s major complex, and all proper subsets are excluded:

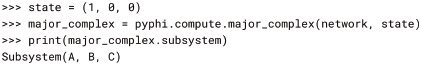


Note that since pyphi.compute.major_complex() is a function of the Network, rather than a particular Subsystem, it is necessary to specify the state in which the system should be analyzed.

## Design and implementation

PyPhi was designed to be easy to use in interactive, exploratory research settings while nonetheless remaining suitable for use in large-scale simulations or as a component in larger applications. It was also designed to be efficient, given the high computational complexity of the algorithms in IIT. Here we describe some implementation details and optimizations used in the software.

### Representation of the TPM and probability distributions

PyPhi supports three different TPM representations: 2-*dimensional state-by-node*, *multidimensional state-by-node*, and *state-by-state*. The state-by-node form is the canonical representation in PyPhi, with the 2-dimensional form used for input and visualization and the multidimensional form used for internal computation. The state-by-state representation is given as an input option for those accustomed to this more general form. If the TPM is given in state-by-state form, PyPhi will raise an error if it does not satisfy Eq (1) (conditional independence).

#### 2-dimensional state-by-node form

A TPM in state-by-node form is a matrix where the entry (*i*, *j*) gives the probability that the *j*^th^ node will be ON at *t* + 1 if the system is in the *i*^th^ state at *t*. This representation has the advantage of being more compact than the state-by-state form, with 2^*n*^ × *n* entries instead of 2^*n*^ × 2^*n*^, where *n* is the number of nodes. Note that the TPM admits this representation because in PyPhi the nodes are binary; both Pr(*N*_*t*+1_ = ON) and Pr(*N*_*t*+1_ = OFF) can be specified by a single entry, in our case Pr(*N*_*t*+1_ = ON), since the two probabilities must sum to 1.

Because the possible system states at *t* are represented implicitly as row indices in 2-dimensional TPMs, there must be an implicit mapping from states to indices. In PyPhi this mapping is achieved by listing the state tuples in lexicographical order and then interpreting them as binary numbers where the state of the first node corresponds to the least-significant bit, so that *e.g*. the state (0, 0, 0, 1) is mapped to the row with index 8 (the ninth row, since Python uses zero-based indexing [18]). Designating the first node’s state as the least-significant bit is analogous to choosing the little-endian convention in organizing computer memory. This convention is preferable because the mapping is stable under the inclusion of new nodes: including another node in a subsystem only requires concatenating new rows and a new column to its TPM rather than interleaving them. Note that this is opposite convention to that used in writing numbers in positional notation; care must be taken when converting between states and indices and between different TPM representations (the pyphi.convert module provides convenience functions for these purposes).

#### Multidimensional state-by-node form

When a state-by-state TPM is provided to PyPhi by the user, it is converted to state-by-node form and the conditional independence property (Eq (1)) is checked. Note that any TPM in state-by-node form necessarily satisfies Eq (1). For internal computations, the TPM is then reshaped so that it has *n* + 1 dimensions rather than two: the first *n* dimensions correspond to the states of each of the *n* nodes at *t*, while the last dimension corresponds to the probabilities of each node being ON at *t* + 1. In other words, the indices of the rows (current states) in the 2-dimensional TPM are “unraveled” into *n* dimensions, with the *i*^th^ dimension indexed by the *i*^*th*^ bit of the 2-dimensional row index according to the little-endian convention. Because the TPM is stored in a NumPy array, this multidimensional form allows us to take advantage of NumPy indexing [19] and use a state tuple as an index directly, without converting it to an integer index:

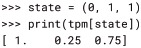


The first entry of this array signifies that if the state of the system is (0, 1, 1) at *t*, then the probability of the first node *N*_0_ being ON at *t* + 1 is Pr(*N*_0,*t*+1_ = ON) = 1. Similarly, the second entry means Pr(*N*_1,*t*+1_ = ON) = 0.25 and the third entry means Pr(*N*_2,*t*+1_ = ON) = 0.75.

Most importantly, the multidimensional representation simplifies the calculation of marginal and conditional distributions and cause/effect repertoires, because it allows efficient “broadcasting” [19] of probabilities when multiplying distributions. Specifically, the Python multiplication operator ‘*’ acts as the tensor product when the operands are NumPy arrays A and B of equal dimensionality such that for each dimension d, either A.shape[d] == 1 or B.shape[d] == 1.

### Calculation of cause/effect repertoires from the TPM

The cause and effect repertoires of a mechanism over a purview describe how the mechanism nodes in a particular state at *t* constrain the possible states of the purview nodes at *t* − 1 and *t* + 1, respectively. Here we describe how they are derived from the TPM in PyPhi.

#### The effect repertoire

We begin with the simplest case: calculating the effect repertoire of a mechanism *M* ⊆ *S* over a purview consisting of a single element *P*_*i*_ ∈ *S*. This is defined as a conditional probability distribution over states of the purview element at *t* + 1 given the current state of the mechanism,

$$\begin{array}{c} \text{effect}\_\text{repertoire}\left(M,\;P_{i}\right)≔\text{Pr}\left(P_{i,t+1}|M_{t}=m_{t}\right). \end{array}$$ (2)

It is derived from the TPM by conditioning on the state of the mechanism elements, marginalizing over the states of non-purview elements *P*′ = *S*\*P*_*i*_ (these states correspond to columns in the state-by-state TPM), and marginalizing over the states of non-mechanism elements *M*′ = *S*\*M* (these correspond to rows):

$$\begin{array}{l} \text{Pr}(P_{i,t+1}|M_{t}=m_{t})\;=\\ \;\;\;\;\frac{1}{\left|\Omega_{M^{\prime }}\right|}\;\sum_{m_{t}^{\prime }\;\in \;\Omega_{M^{\prime }}}\;\frac{1}{\left|\Omega_{P^{\prime }}\right|}\;\sum_{p_{t+1}^{\prime }\;\in \;\Omega_{P^{\prime }}}\;\text{Pr}(P_{i,t+1},p_{t+1}^{\prime }|M=m_{t},M^{\prime }=m_{t}^{\prime }). \end{array}$$

This operation is implemented in PyPhi by several subroutines. First, in a pre-processing step performed when the Subsystem object is created, a Node object is created for each element in the subsystem. Each Node contains its own individual TPM, extracted from the subsystem’s TPM; this is a 2^*s*^ × 2 matrix where *s* is the number of the node’s parents and the entry (*i*, *j*) gives the probability that the node is in state *j* (0 or 1) at *t* + 1 given that its parents are in state *i* at *t*. This node TPM is represented internally in multidimensional state-by-node form as usual, with singleton dimensions for those subsystem elements that are not parents of the node. The effect repertoire is then calculated by conditioning the purview node’s TPM on the state of the mechanism nodes that are also parents of the purview node, via the pyphi.tpm.condition_tpm() function, and marginalizing out non-mechanism nodes, with pyphi.tpm.marginalize_out().

In cases where there are mechanism nodes that are not parents of the purview node, the resulting array is multiplied by an array of ones that has the desired shape (dimensions of size two for each mechanism node, and singleton dimensions for each non-mechanism node). Because of NumPy’s broadcasting feature, this step is equivalent to taking the tensor product of the array with the maximum-entropy distribution over mechanism nodes that are not parents, so that the final result is a distribution over all mechanism nodes, as desired.

The effect repertoire over a purview of more than one element is given by the tensor product of the effect repertories over each individual purview element,

$$\begin{array}{c} \text{effect}\_\text{repertoire}\left(M,\;P\right)≔\underset{P_{i}\;\in \;P}{⊗}\text{effect}\_\text{repertoire}\left(M,\;P_{i}\right). \end{array}$$ (3)

Again, because PyPhi TPMs and repertoires are represented as tensors (multidimensional arrays), with each dimension corresponding to a node, the NumPy multiplication operator between distributions over different nodes is equivalent to the tensor product. Thus the effect repertoire over an arbitrary purview is trivially implemented by taking the product of the effect repertoires over each purview node with numpy.multiply().

#### The cause repertoire

The cause repertoire of a single-element mechanism *M*_*i*_ ∈ *S* over a purview *P* ⊆ *S* is defined as a conditional probability distribution over the states of the purview at *t* − 1 given the current state of the mechanism,

$$\begin{array}{c} \text{cause}\_\text{repertoire}\left(M_{i},\;P\right)≔\text{Pr}\left(P_{t-1}|M_{i,t}=m_{i,t}\right). \end{array}$$ (4)

As with the effect repertoire, it is obtained by conditioning and marginalizing the TPM. However, because the TPM gives conditional probabilities of states at *t* + 1 given the state at *t*, Bayes’ rule is first applied to express the cause repertoire in terms of a conditional distribution over states at *t* − 1 given the state at *t*,

$$\begin{array}{c} \text{Pr}\left(P_{t-1}|M_{i,t}=m_{i,t}\right)\;=\;\frac{\text{Pr}\left(m_{i,t}|P_{t-1}\right)\;\text{Pr}\left(P_{t-1}\right)}{\text{Pr}(m_{i,t})}. \end{array}$$

where the marginal distribution Pr(*P*_*t*−1_) over previous states is the uniform distribution. In this way, the analysis captures how a mechanism in a state constrains a purview without being biased by whether certain states arise more frequently than others in the dynamical evolution of the system [3, 4, 11, 16]. Then the cause repertoire can be calculated by marginalizing over the states of non-mechanism elements *M*′ = *S*\*M*_*i*_ (now corresponding to columns in the state-by-state TPM) and non-purview elements *P*′ = *S*\*P* (now corresponding to rows),

$$\begin{array}{l} \frac{\text{Pr}(m_{i,t}|P_{t-1})\;\text{Pr}(P_{t-1})}{\text{Pr}(m_{i,t})}\\ \;=\;\frac{\left(\frac{1}{\left|\Omega_{P^{\prime }}\right|}\;\sum_{p_{t-1}^{\prime }\;\in \;\Omega_{P^{\prime }}}\;\frac{1}{\left|\Omega_{M^{\prime }}\right|}\;\sum_{m_{t}^{\prime }\;\in \;\Omega_{M^{\prime }}}\;\text{Pr}(m_{i,t},m_{t}^{\prime }|P_{t-1},P_{t-1}^{\prime }=p_{t-1}^{\prime })\right)\text{Pr}(P_{t-1})}{\frac{1}{\left|\Omega_{M^{\prime }}\right|}\;\sum_{m_{t}^{\prime }\;\in \;\Omega_{M^{\prime }}}\;\text{Pr}(m_{i,t},m_{t}^{\prime })}\\ \;=\;\frac{\left(\frac{1}{\left|\Omega_{P^{\prime }}\right|}\;\sum_{p_{t-1}^{\prime }\in \;\Omega_{P^{\prime }}}\;\sum_{m_{t}^{\prime }\;\in \;\Omega_{M^{\prime }}}\;\text{Pr}(m_{t},m_{t}^{\prime }|P_{t-1},P_{t-1}^{\prime }=p_{t-1}^{\prime })\right)\text{Pr}(P_{t-1})}{\sum_{m_{t}^{\prime }\;\in \;\Omega_{M^{\prime }}}\;\text{Pr}(m_{i,t},m_{t}^{\prime })}. \end{array}$$

In PyPhi, the “backward” conditional probabilities Pr(*m*_*i*,*t*_ | *P*_*t*−1_) for a single mechanism node are obtained by indexing into the last dimension of the node’s TPM with the state *m*_*i*,*t*_ and then marginalizing out non-purview nodes via pyphi.tpm.marginalize_out(). As with the effect repertoire, the resulting array is then multiplied by an array of ones with the desired shape in order to obtain a distribution over the entire purview. Finally, because in this case the probabilities were obtained from columns of the TPM, which do not necessarily sum to 1, the distribution is normalized with pyphi.distribution.normalize().

The cause repertoire of a mechanism with multiple elements is the normalized tensor product of the cause repertoires of each individual mechanism element,

$$\begin{array}{c} \text{cause}\_\text{repertoire}\left(M,\;P\right)\;=\;\frac{1}{K}\;\underset{M_{i}\;\in \;M}{⊗}\text{cause}\_\text{repertoire}\left(M_{i},\;P\right), \end{array}$$ (5)

where

$$\begin{array}{c} K\;=\;\sum_{p_{t-1}\;\in \;\Omega_{P}}\;\prod_{m_{i,t}\;\in \;\Omega_{M}}\text{Pr}\left(P_{t-1}=p_{t-1}|M_{i,t}=m_{i,t}\right) \end{array}$$

is a normalization factor that ensures that the distribution sums to 1. This is implemented in PyPhi via numpy.multiply() and pyphi.distribution.normalize(). For a more complete illustration of these procedures, see S1 Text.

### Code organization and interface design

The postulates of IIT induce a natural hierarchy of computations [1, Supplementary Information S2], and PyPhi’s implementation mirrors this hierarchy by using object-oriented programming (Table 1) and factoring the computations into compositions of separate functions where possible. One advantage of this approach is that each level of the computation can be performed independently of the higher levels; for example, if one were interested only in the MIE of certain mechanisms rather than the full MICS, then one could simply call Subsystem.effect_mip() on those mechanisms instead of calling pyphi.compute.sia() and extracting them from the resulting SystemIrreducibilityAnalysis object (this is especially important in the case of large systems where the full calculation is infeasible). Separating the calculation into many subroutines and exposing them to the user also has the advantage that they can be easily composed to implement functionality that is not already built-in.

**Table 1:** Correspondence between theoretical objects and PyPhi objects.

Theoretical object	PyPhi object
Discrete dynamical system	Network
Candidate system	Subsystem
System element	Node in Subsystem.nodes
System state	Python tuple containing a 0 or 1 for each node
Mechanism	Python tuple of node indices
Purview	Python tuple of node indices
Repertoire over a purview *P*	NumPy array with |*P*| dimensions, each of size 2
MIP	The partition attribute of the RepertoireIrreducibilityAnalysis returned by Subsystem.cause_mip() or Subsystem.effect_mip()
MIC and MIE	MaximallyIrreducibleCause and MaximallyIrreducibleEffect
Concept	Concept
*φ*	The phi attribute of a Concept
CES	CauseEffectStructure
Φ	The phi attribute of a CauseEffectStructure
MICS	The ces attribute of the SystemIrreducibilityAnalysis returned by pyphi.compute.major_complex()
Complex	The subsystem attribute of the SystemIrreducibilityAnalysis returned by pyphi.compute.major_complex()

### Configuration

Many aspects of PyPhi’s behavior may be configured via the pyphi.config object. The configuration can be specified in a YAML file [20]; an example is available in the GitHub repository. When PyPhi is imported, it checks the current directory for a file named pyphi_config.yml and automatically loads it if it exists. Configuration settings can also be loaded on the fly from an arbitrary file with the pyphi.config.load_config_file() function.

Alternatively, pyphi.config.load_config_dict() can load configuration settings from a Python dictionary. Many settings can also be changed by directly assigning them a new value.

Default settings are used if no configuration is provided. A full description of the available settings and their default values is available in the “Configuration” section of the online documentation.

### Optimizations and approximations

Here we describe various optimizations and approximations used by the software to reduce the complexity of the calculations (see § Limitations). Memoization and caching optimizations are described in S2 Text.

#### Connectivity optimizations

As mentioned in § Input, providing connectivity information explicitly with a CM can greatly reduce the time complexity of the computations, because in certain cases missing connections imply reducibility *a priori*.

For example, at the system level, if the subsystem is not strongly connected then Φ is necessarily zero. This is because a unidirectional cut between one system part and the rest can always be found that will not actually remove any edges, so the CESs with and without the cut will be identical (see S3 Text for proof). Accordingly, PyPhi immediately excludes these subsystems when finding the major complex of a system.

Similarly, at the mechanism level, PyPhi uses the CM to exclude certain purviews from consideration when computing a MIC or MIE by efficiently determining that repertoires over those purviews are reducible without needing to explicitly compute them. Suppose there are two sets of nodes *X* and *Y* for which there exist partitions *X* = (*X*_1_, *X*_2_) and *Y* = (*Y*_1_, *Y*_2_) such that there are no edges from *X*_1_ to *Y*_2_ and no edges from *X*_2_ to *Y*_1_. Then the effect repertoire of mechanism *X* over purview *Y* can be factored as

$$\begin{array}{l} \text{effect\_repertoire}(X,Y)\;=\;\\ \;\;\text{effect\_repertoire}(X_{1},Y_{1})\;⊗\;\text{effect\_repertoire}(X_{2},Y_{2}), \end{array}$$

and the cause repertoire of mechanism *Y* over purview *X* can be factored as

$$\begin{array}{l} \text{cause\_repertoire}(Y,X)\;=\;\\ \;\text{cause\_repertoire}(Y_{1},X_{1})\;⊗\;\text{cause\_repertoire}(Y_{2},X_{2}). \end{array}$$

Thus in these cases the mechanism is reducible for that purview and *φ* = 0 (see S4 Text for proof).

#### Analytical solution to the earth mover’s distance

One of the repertoire distances available in PyPhi is the earth mover’s distance (EMD), with the Hamming distance as the ground metric. Computing the EMD between repertoires is a costly operation, with time complexity *O*(*n*2^3*n*^) where *n* is the number of nodes in the purview [21]. However, when comparing effect repertoires, PyPhi exploits a theorem that states that the EMD between two distributions *p* and *q* over multiple nodes is the sum of the EMDs between the marginal distributions over each individual node, if *p* and *q* are independent. This analytical solution has time complexity *O*(*n*), a significant improvement over the general EMD algorithm (note that this estimate does not include the cost of computing the marginals, which already have been computed to obtain the repertoires). By the conditional independence property (Eq 1), the conditions of the theorem hold for EMD calculations between effect repertoires, and thus the analytical solution can be used for half of all repertoire calculations performed in the analysis. The theorem is formally stated and proved in S5 Text.

#### Approximations

Currently, two approximate methods of computing Φ are available. These can be used via settings in the PyPhi configuration file (they are disabled by default):

1. pyphi.config.CUT_ONE_APPROXIMATION (the “cut one” approximation), and
2. pyphi.config.ASSUME_CUTS_CANNOT_CREATE_NEW_CONCEPTS (the “no new concepts” approximation).

In both cases, the complexity of the calculation is greatly reduced by replacing the optimal partitioned CES by an approximate solution. The system’s Φ value is then computed as usual as the difference between the unpartitioned CES and the approximate partitioned CES.

#### Cut one

The “cut one” approximation reduces the scope of the search for the MIP over possible system cuts. Instead of evaluating the partitioned CES for each of the 2^*n*^ unidirectional bipartitions of the system, only those 2*n* bipartitions are evaluated that sever the edges from a single node to the rest of the network or vice versa. Since the goal is to find the minimal Φ value across all possible partitions, the “cut one” approximation provides an upper bound on the exact Φ value of the system.

#### No new concepts

For most choices of mechanism partitioning schemes and distance measures, it is possible that the CES of the partitioned system contains concepts that are reducible in the unpartitioned system and thus not part of the unpartitioned CES. For this reason, PyPhi by default computes the partitioned CES from scratch from the partitioned TPM. Under the “no new concepts” approximation, such new concepts are ignored. Instead of repeating the entire CES computation for each system partition, which requires reevaluating all possible candidate mechanisms for irreducibility, only those mechanisms are taken into account that already specify concepts in the unpartitioned CES. In many types of systems, new concepts due to the partition are rare. Approximations using the “no new concepts” option are thus often accurate. Note, however, that this approximation provides neither a theoretical upper nor lower bound on the exact Φ value of the system.

### Limitations

PyPhi’s main limitation is that the algorithm is exponential time in the number of nodes, *O*(*n*53^*n*^). This is because the number of states, subsystems, mechanisms, purviews, and partitions that must be considered each grows exponentially in the size of the system. This limits the size of systems that can be practically analyzed to ~10–12 nodes. For example, calculating the major complex of systems of three, five, and seven stochastic majority gates, connected in a circular chain of bidirectional edges, takes ~1 s, ~16 s, and ~2.75 h respectively (parallel evaluation of system cuts, 32 × 3.1GHz CPU cores). Using the “cut one” approximation, these calculations take ~1 s, ~12 s, and ~0.63 h. In practice, actual execution times are substantially variable and depend on the specific network under analysis, because network structure determines the effectiveness of the optimizations discussed above.

Another limitation is that the analysis can only be meaningfully applied to a system that is Markovian and satisfies the conditional independence property. These are reasonable assumptions for the intended use case of the software: analyzing a causal TPM derived using the calculus of perturbations [17]. However, there is no guarantee that these assumptions will be valid in other circumstances, such as TPMs derived from observed time series (*e.g*., EEG recordings). Whether a system has the Markov property and conditional independence property should be carefully checked before applying the software in novel contexts.

## Availability and future directions

PyPhi can be installed with Python’s package manager via the command ‘pip install pyphi’ on Linux and macOS systems equipped with Python 3.4 or higher. It is open-source and licensed under the GNU General Public License v3.0. The source code is version-controlled with git and hosted in a public repository on GitHub at https://github.com/wmayner/pyphi. Comprehensive and continually-updated documentation is available online at https://pyphi.readthedocs.io. The pyphi-users mailing list can be joined at https://groups.google.com/forum/#!forum/pyphi-users. A web-based graphical interface to the software is available at http://integratedinformationtheory.org/calculate.html.

Several additional features are in development and will be released in future versions. These include a module for calculating Φ over multiple spatial and temporal scales, as theoretically required by the exclusion postulate (in the current version, the Network is assumed to represent the system at the spatiotemporal timescale at which Φ is maximized [10, 12]), and a module implementing a calculus for “actual causation” as formulated in [15] (preliminary versions of these modules are available in the current release). The software will also be updated to reflect developments in IIT and further optimizations in the algorithm.

## Supporting information

S1 Text Calculating Φ. Illustration of the algorithm.
(PDF)

S2 Text Memoization and caching optimizations. (PDF)

S3 Text Proof of the strong connectivity optimization. (PDF)

S4 Text Proof of the block-factorable optimization. (PDF)

S5 Text Proof of an analytical solution to the EMD between effect repertoires. (PDF)

S1 File PyPhi v1.1.0 source code. Note that installing PyPhi via ‘pip’ or downloading the source code from GitHub is recommended in order to obtain the most up-to-date version of the software.
(ZIP)

S2 File PyPhi v1.1.0 documentation. Note that accessing the documentation online at https://pyphi.readthedocs.io is recommended, as it is updated for each new version of the software.
(ZIP)

## References

[1] Tononi G, Boly M, Massimini M, Koch C. Integrated information theory: from consciousness to its physical substrate. Nature Reviews Neuroscience. 2016;17(7):450–461. 10.1038/nrn.2016.44 27225071

[2] Tononi G. Integrated information theory. Scholarpedia. 2015;10(1):4164 10.4249/scholarpedia.4164

[3] Oizumi M, Albantakis L, Tononi G. From the Phenomenology to the Mechanisms of Consciousness: Integrated Information Theory 3.0. PLoS computational biology. 2014;10(5):e1003588 10.1371/journal.pcbi.1003588 24811198 PMC4014402

[4] Balduzzi D, Tononi G. Integrated information in discrete dynamical systems: motivation and theoretical framework. PLoS computational biology. 2008;4(6):e1000091 10.1371/journal.pcbi.1000091 18551165 PMC2386970

[5] Tononi G. An information integration theory of consciousness. BMC neuroscience. 2004;5(1):42 10.1186/1471-2202-5-42 15522121 PMC543470

[6] Albantakis L, Tononi G. The Intrinsic Cause-Effect Power of Discrete Dynamical Systems—From Elementary Cellular Automata to Adapting Animats. Entropy. 2015;17(8):5472 10.3390/e17085472

[7] Albantakis L, Hintze A, Koch C, Adami C, Tononi G. Evolution of Integrated Causal Structures in Animats Exposed to Environments of Increasing Complexity. PLoS computational biology. 2014;10(12):e1003966 10.1371/journal.pcbi.1003966 25521484 PMC4270440

[8] Oizumi M, Tsuchiya N, Amari S. Unified framework for information integration based on information geometry. Proceedings of the National Academy of Sciences. 2016;113(51):14817–14822. 10.1073/pnas.1603583113 PMC5187746 27930289

[9] Albantakis L, Tononi G. Chapter 14: Automata and Animats. From Matter to Life: Information and Causality. 2017; p. 334 10.1017/9781316584200.014

[10] Hoel EP, Albantakis L, Marshall W, Tononi G. Can the macro beat the micro? Integrated information across spatiotemporal scales. Neuroscience of Consciousness. 2016;2016(1):niw012 10.1093/nc/niw012 PMC6367968 30788150

[11] Hoel EP, Albantakis L, Tononi G. Quantifying causal emergence shows that macro can beat micro. PNAS. 2013;110(49):19790–19795. 10.1073/pnas.1314922110 24248356 PMC3856819

[12] Marshall W, Albantakis L, Tononi G. Black-boxing and cause-effect power. PLoS computational biology. 2018;14(4):e1006114 10.1371/journal.pcbi.1006114 29684020 PMC5933815

[13] Marshall W, Kim H, Walker SI, Tononi G, Albantakis L. How causal analysis can reveal autonomy in models of biological systems. Philosophical Transactions of the Royal Society of London A: Mathematical, Physical and Engineering Sciences. 2017;375 (2109). 10.1098/rsta.2016.0358 PMC5686412 29133455

[14] Marshall W, Gomez-Ramirez J, Tononi G. Integrated Information and State Differentiation. Frontiers in Psychology. 2016;7:926 10.3389/fpsyg.2016.00926 27445896 PMC4923128

[15] Albantakis L, Marshall W, Tononi G. What caused what? An irreducible account of actual causation. arXiv:170806716 [csAI]. 2017.

[16] Ay N, Polani D. Information flows in causal networks. Advances in complex systems. 2008;11(01):17–41. 10.1142/S0219525908001465

[17] Pearl J. Causality. Cambridge university press; 2009.

[18] Dijkstra EW. Why numbering should start at zero (EWD 831); 1982. Available from: https://www.cs.utexas.edu/users/EWD/transcriptions/EWD08xx/EWD831.html.

[19] Walt Svd, Colbert SC, Varoquaux G. The NumPy array: a structure for efficient numerical computation. Computing in Science & Engineering. 2011;13(2):22–30. 10.1109/MCSE.2011.37

[20] Ben-Kiki O, Evans C, Net Id. YAML specification; 2009. Available from: http://yaml.org/spec/.

[21] Pele O, Werman M. Fast and robust earth mover’s distances. In: 2009 IEEE 12th International Conference on Computer Vision. IEEE; 2009. p. 460–467. Available from: 10.1109/ICCV.2009.5459199.

