# Supplementary material for: PyPhi: A toolbox for integrated information theory
Source: PLoS Comput Biol. 2018 Jul 26;14(7):e1006343. doi: 10.1371/journal.pcbi.1006343 (PMC6080800; doi:10.1371/journal.pcbi.1006343)

Calculating Φ

William G. P. Mayner[1,2], William Marshall[2], Larissa Albantakis[2], Graham Findlay[1,2], Robert Marchman[2], Giulio Tononi[2]

[1] Neuroscience Training Program, University of Wisconsin–Madison, Madison, WI, USA

[2] Department of Psychiatry, Wisconsin Institute for Sleep and Consciousness, University of Wisconsin–Madison, Madison, WI, USA

Outline

- Elements, states, and the TPM
- Background conditions
- Cause-effect repertoires
- Integrated mechanisms: φ
- Concepts and cause-effect structures
- Integrated systems: Φ
- Complexes

Outline

- Elements, states, and the TPM
- Background conditions
- Cause-effect repertoires
- Integrated mechanisms: φ
- Concepts and cause-effect structures
- Integrated systems: Φ
- Complexes

Introduction:

Elements, states, and the TPM

- In integrated information theory, a physical system is represented as a network of interconnected elements
- Elements are in one of at least two **states**
- Each element receives input and provides output
- Each element has an **input-output function** for transitioning from one state to another

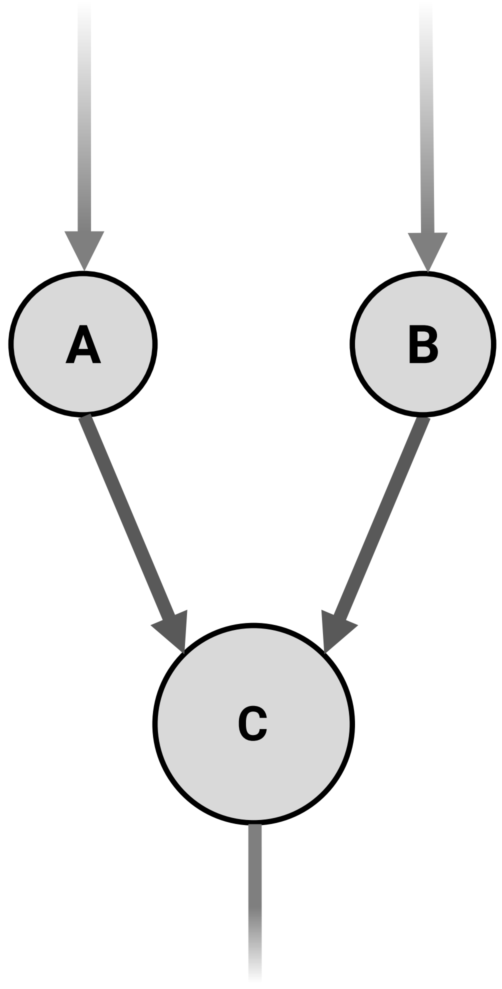

Introduction:
Elements, states, and the TPM

- An element's input-output function can be fully characterized by a **transition probability matrix** (TPM) that gives the probabilities of each possible state transition
- The TPM can be calculated by perturbing the element's inputs into all possible configurations and recording the results

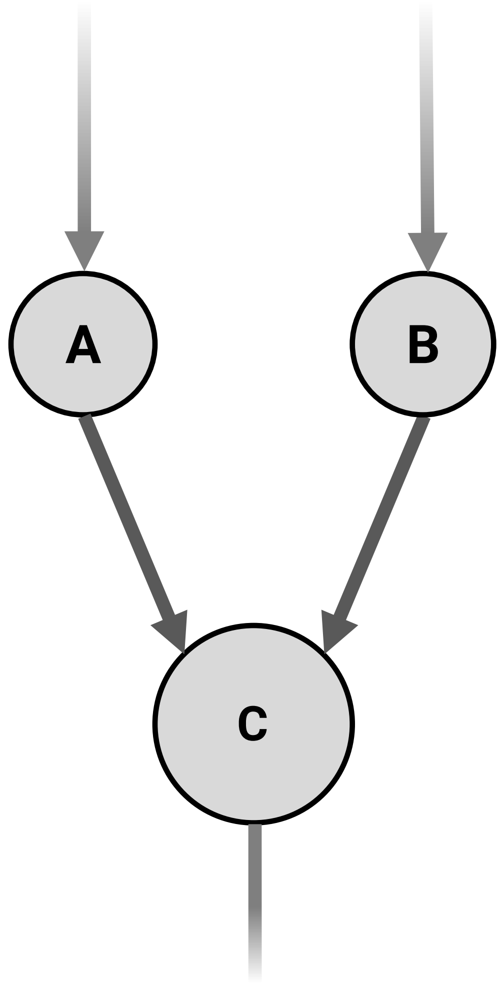

Introduction:
Elements, states, and the TPM

- We'll do this for element **C**
- We start by setting **A** and **B** to their OFF state in the current timestep, t

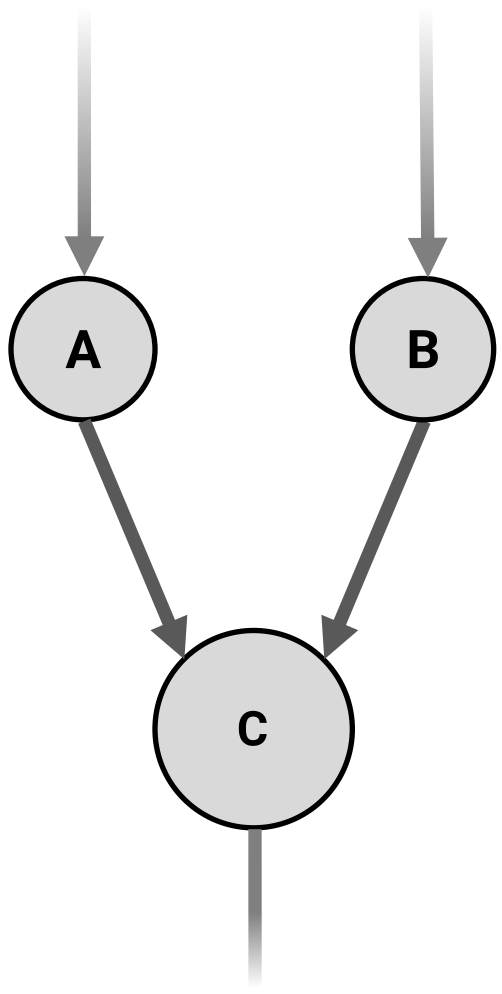

Introduction:
Elements, states, and the TPM

- We'll do this for element **C**
- We start by setting **A** and **B** to their OFF state in the current timestep, t

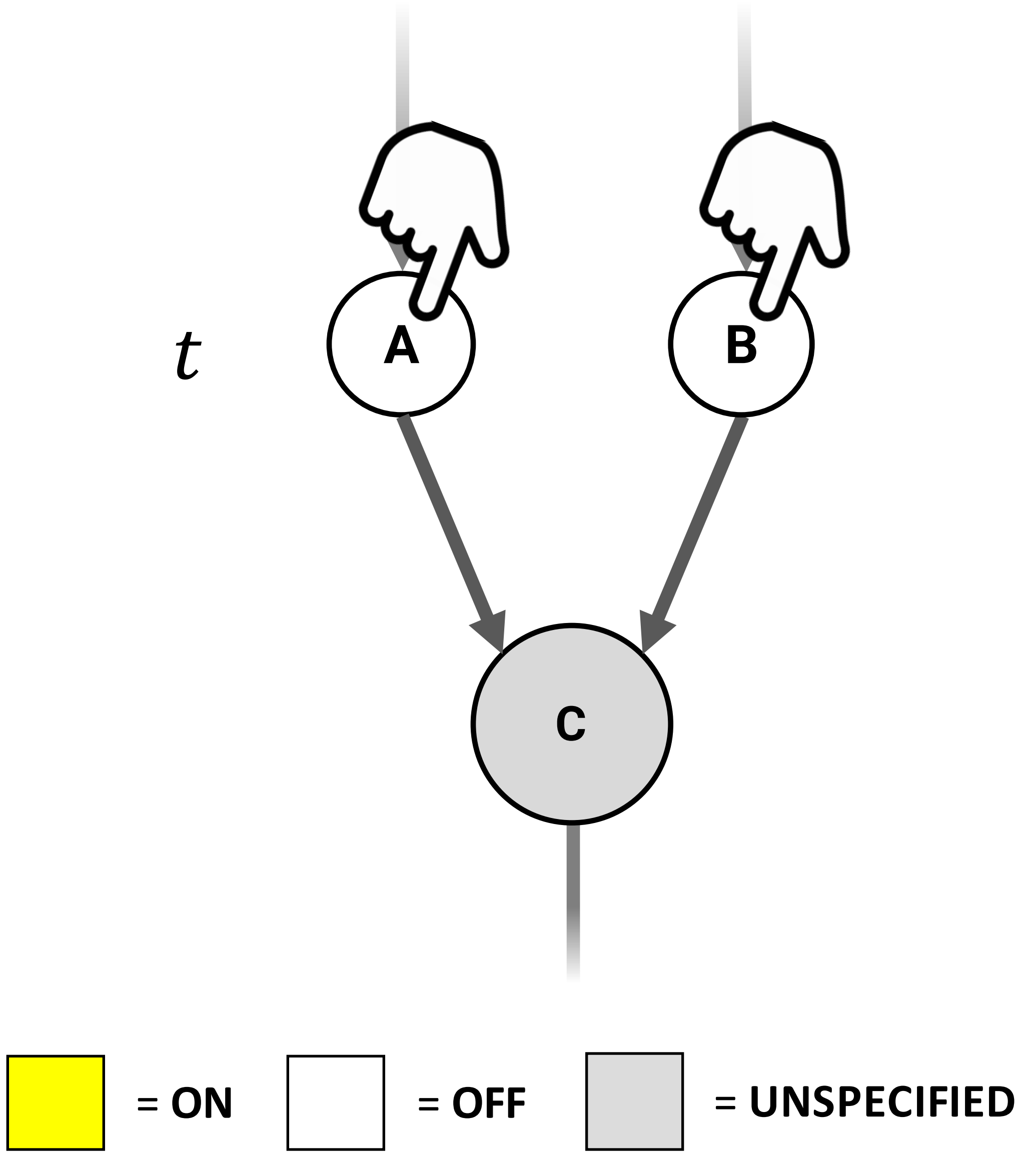

Introduction:

Elements, states, and the TPM

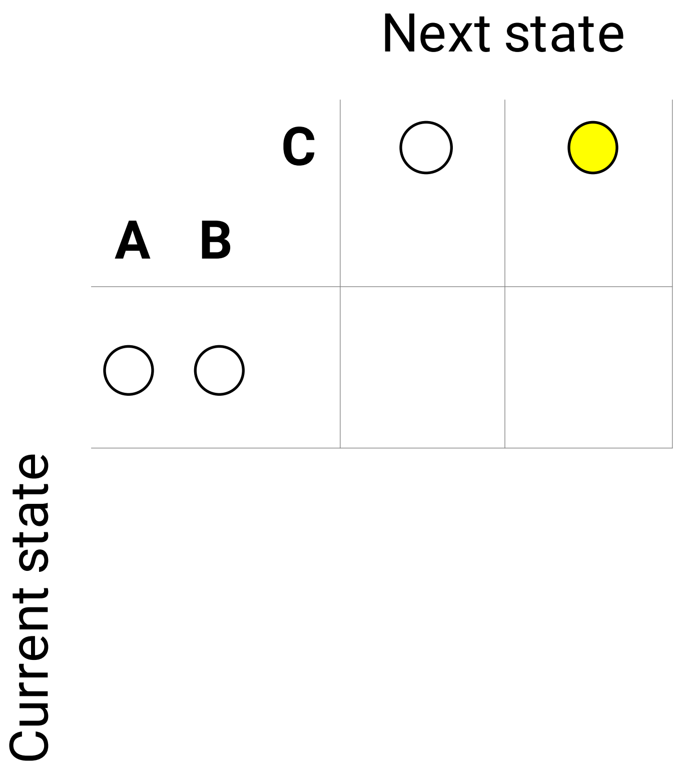

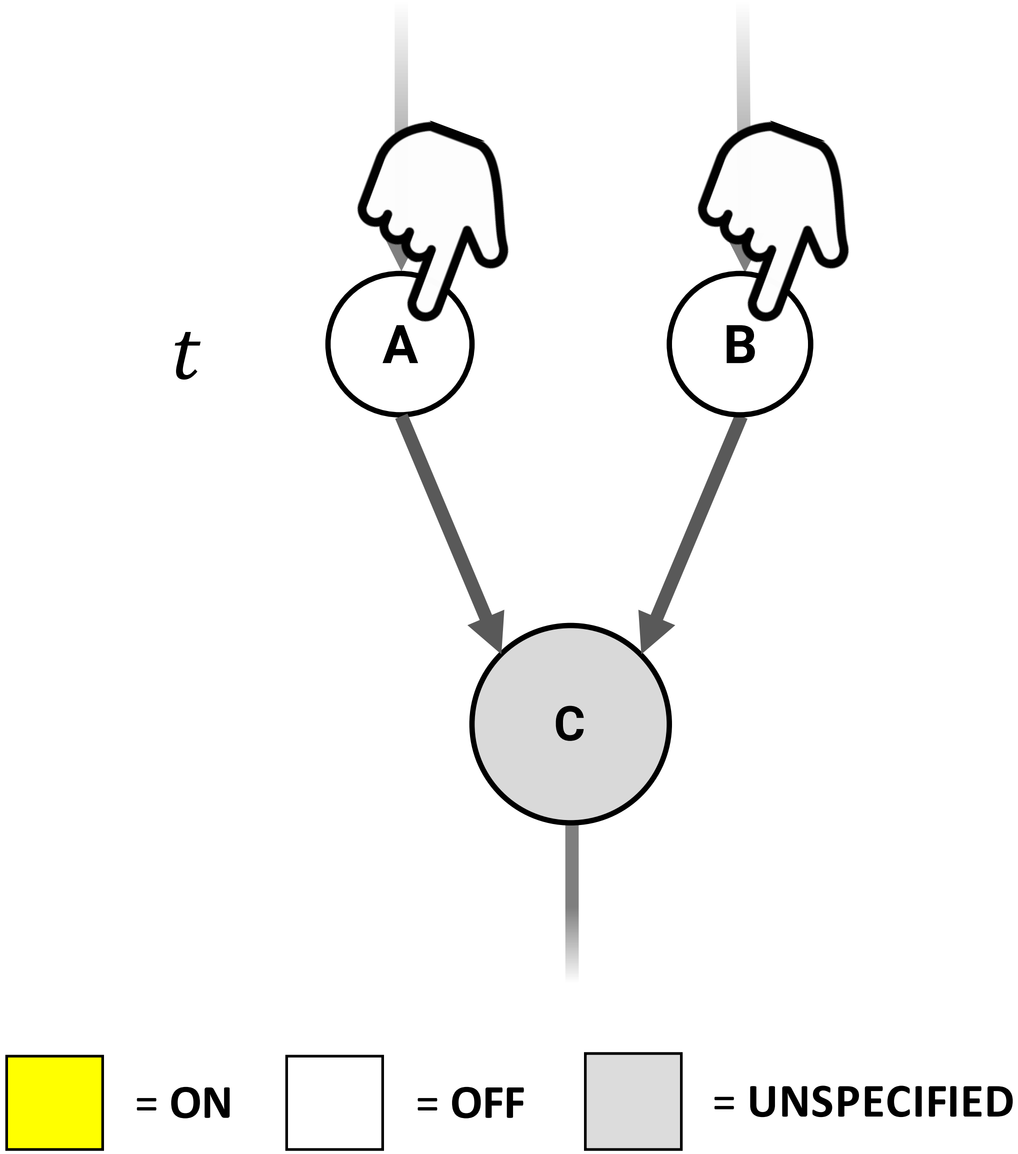

Introduction:
Elements, states, and the TPM

Next state

A	B	C: ○	C: ●
○	○	1	0

Current state

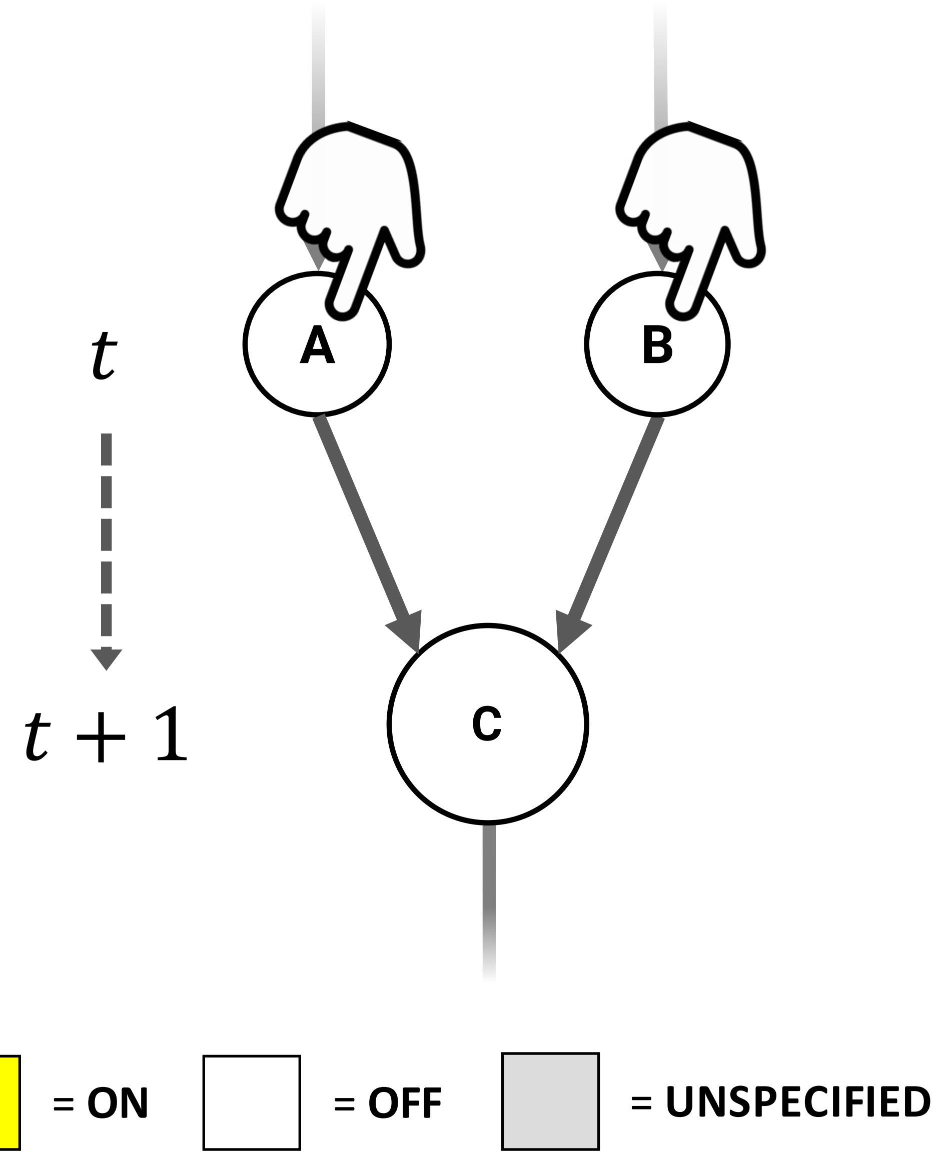

Introduction:

Elements, states, and the TPM

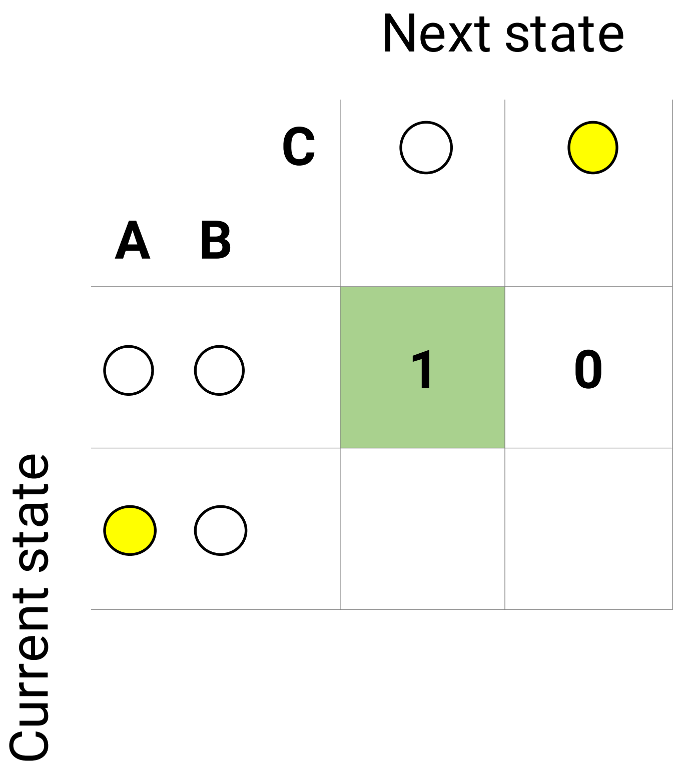

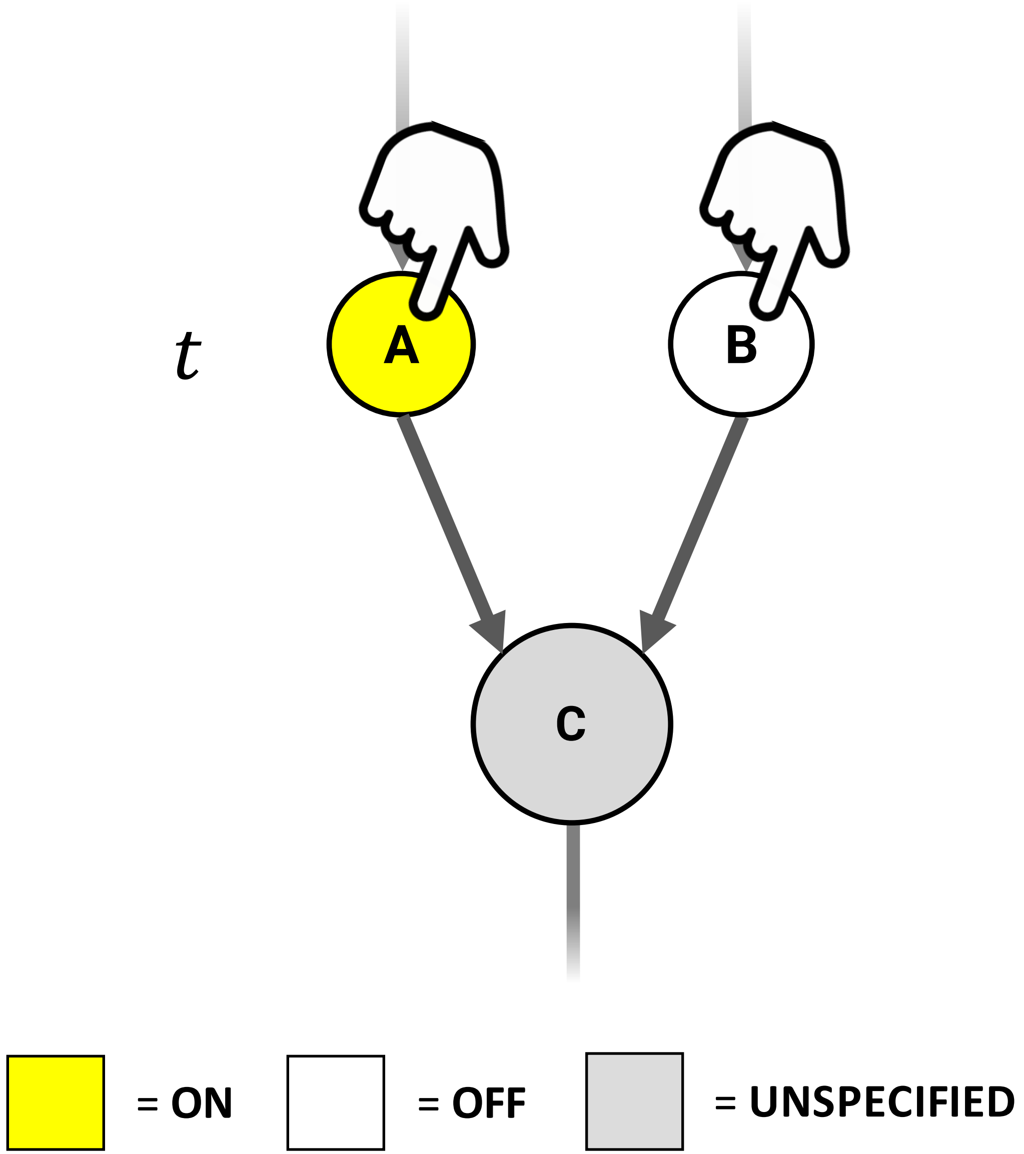

Introduction:
Elements, states, and the TPM

Next state

Current state: A	B	C: ○	C: ●
○	○	1	0
●	○	1	0

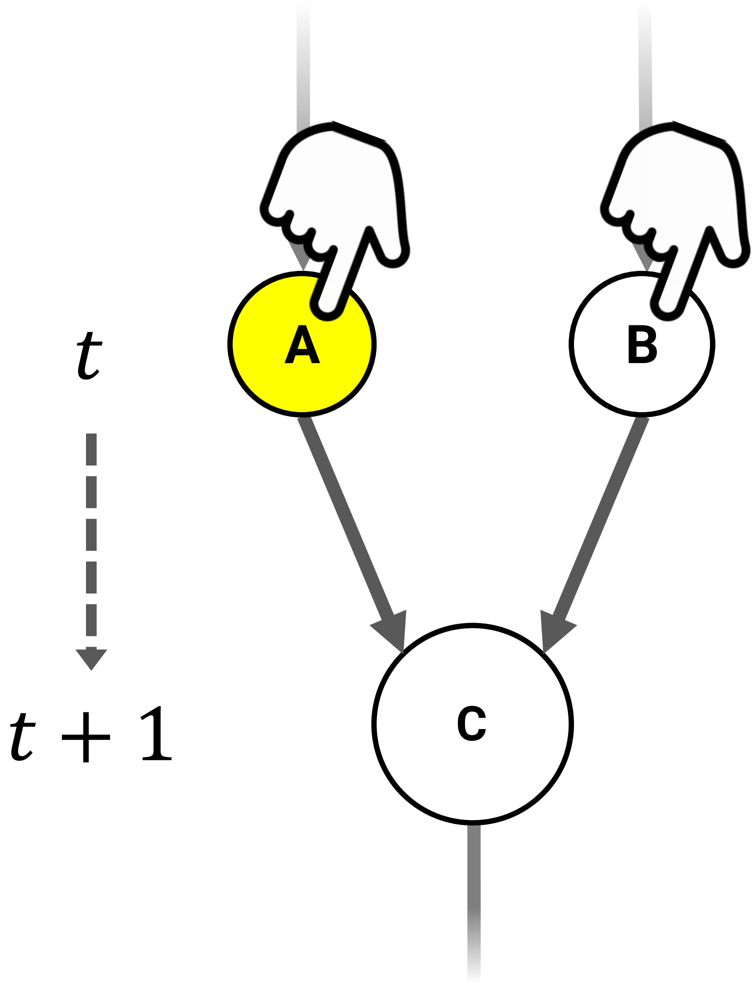

= ON   = OFF   = UNSPECIFIED

Introduction:
Elements, states, and the TPM

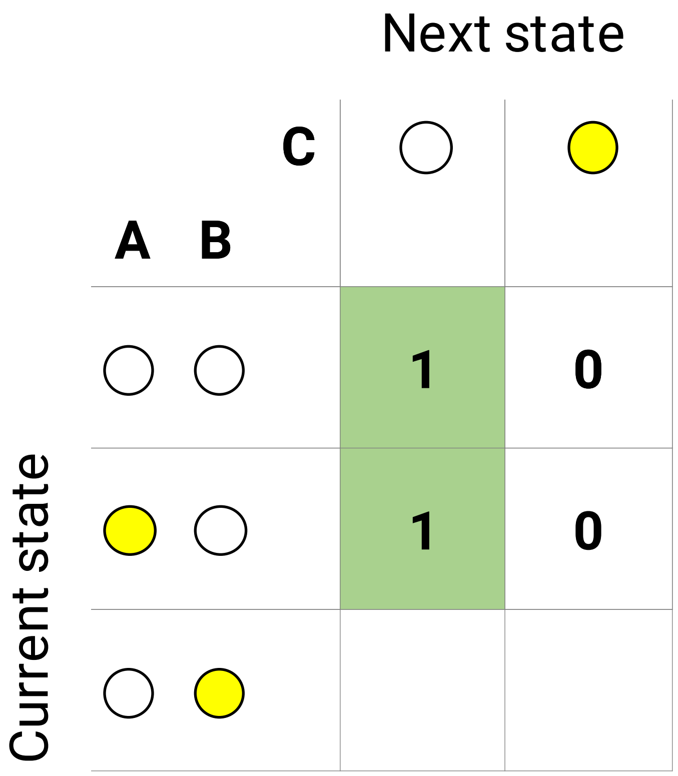

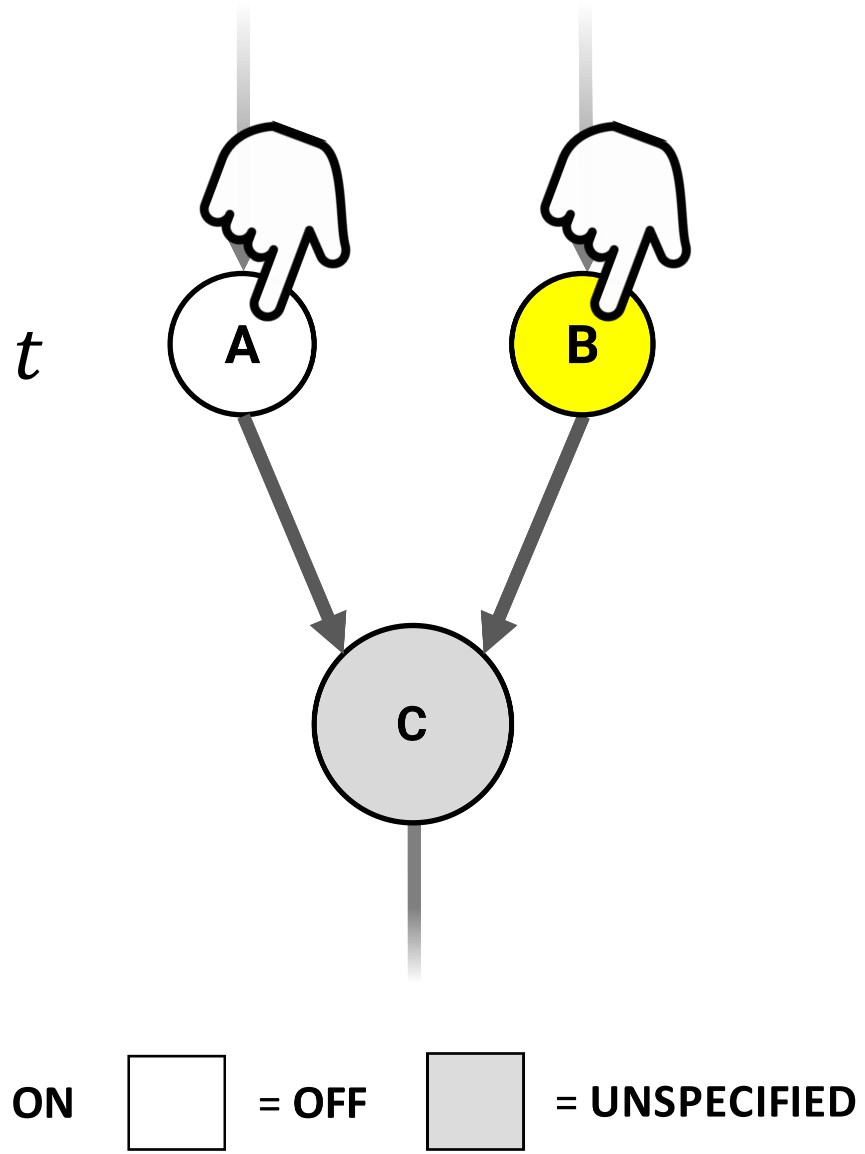

Introduction:
Elements, states, and the TPM

Next state

Current state: A	Current state: B	C: ○	C: ●
○	○	1	0
●	○	1	0
○	●	1	0

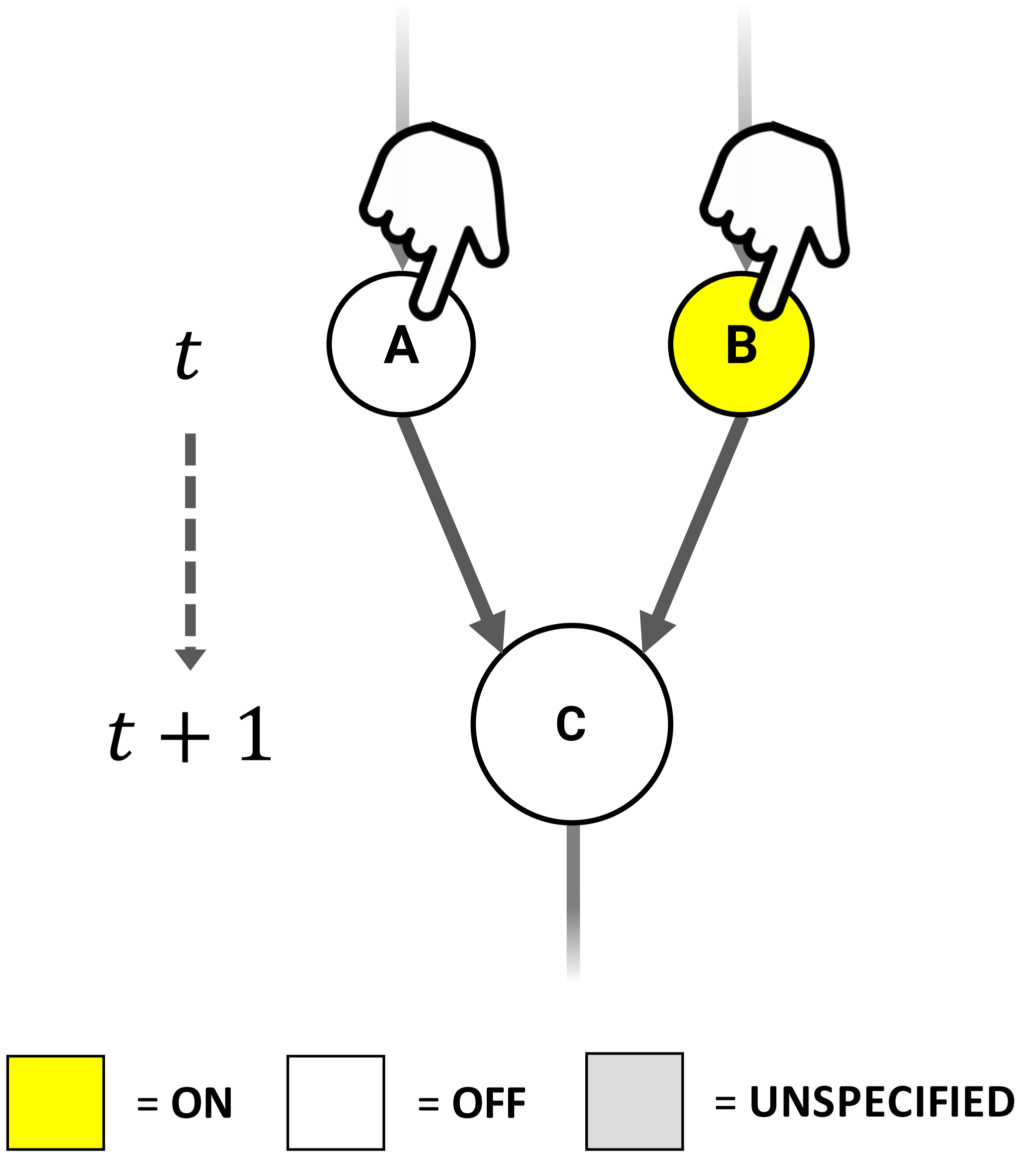

Introduction:

Elements, states, and the TPM

Next state

Current state: A	B	C: ○	C: ●
○	○	1	0
●	○	1	0
○	●	1	0
●	●		

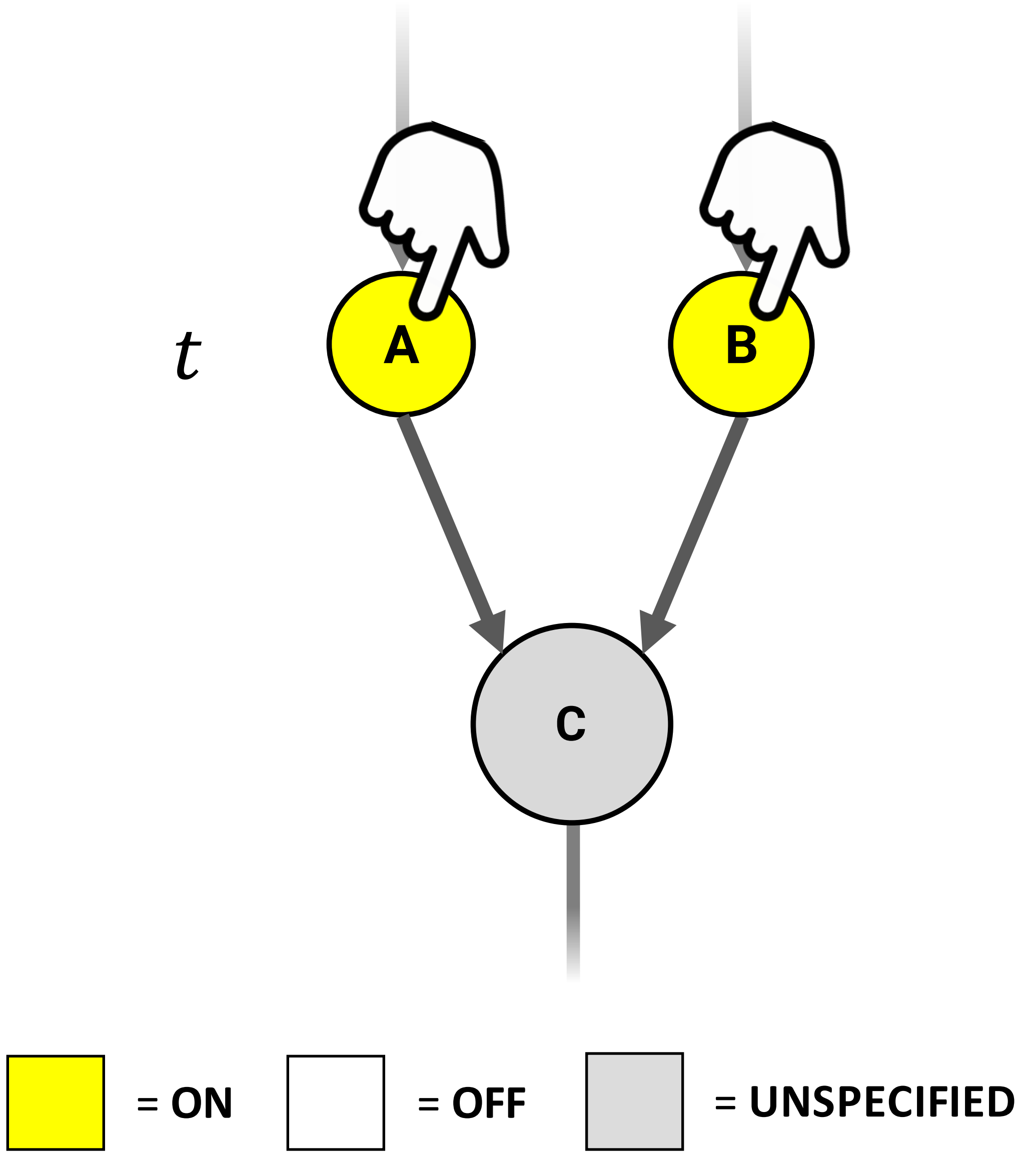

Introduction:

Elements, states, and the TPM

Next state

Current state (A B) \ C	OFF	ON
OFF OFF	1	0
ON OFF	1	0
OFF ON	1	0
ON ON	0	1

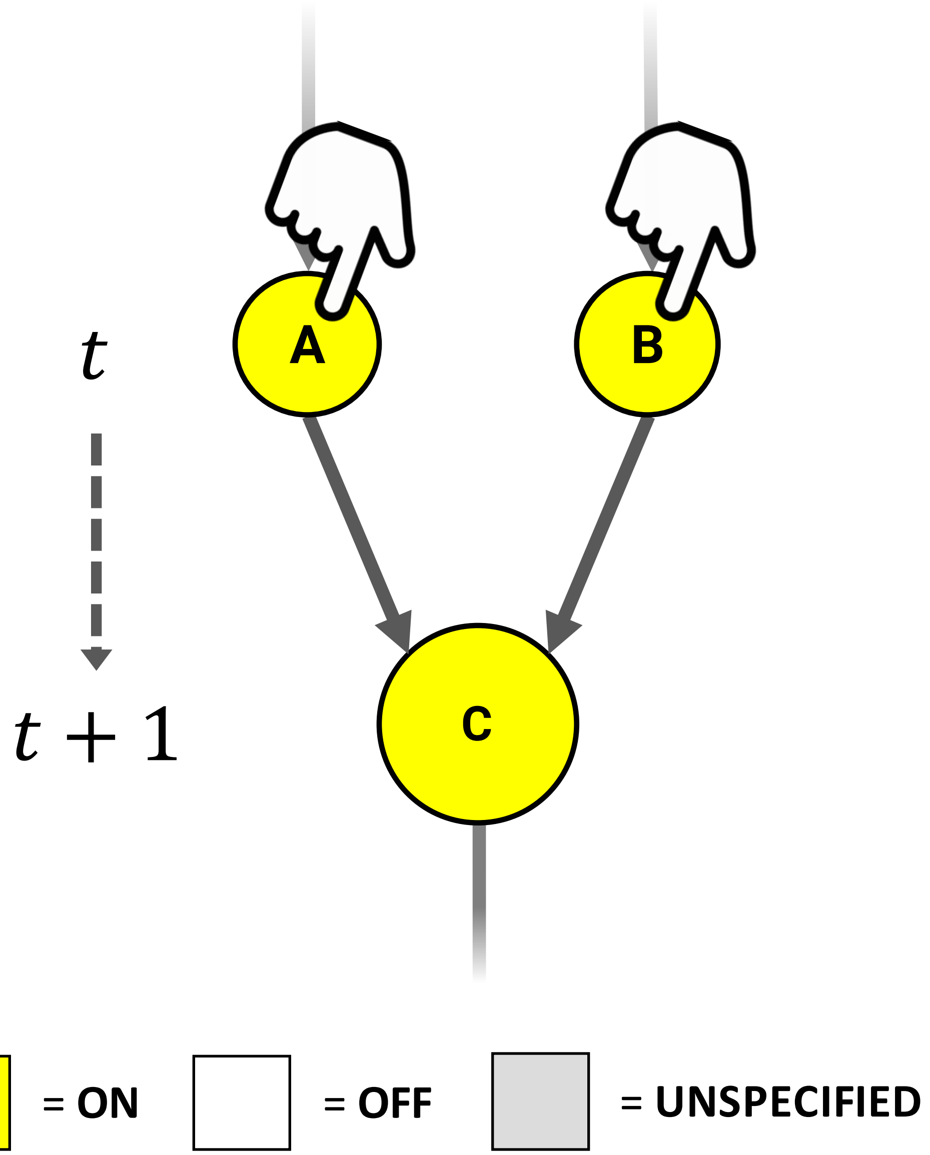

Introduction:
Elements, states, and the TPM

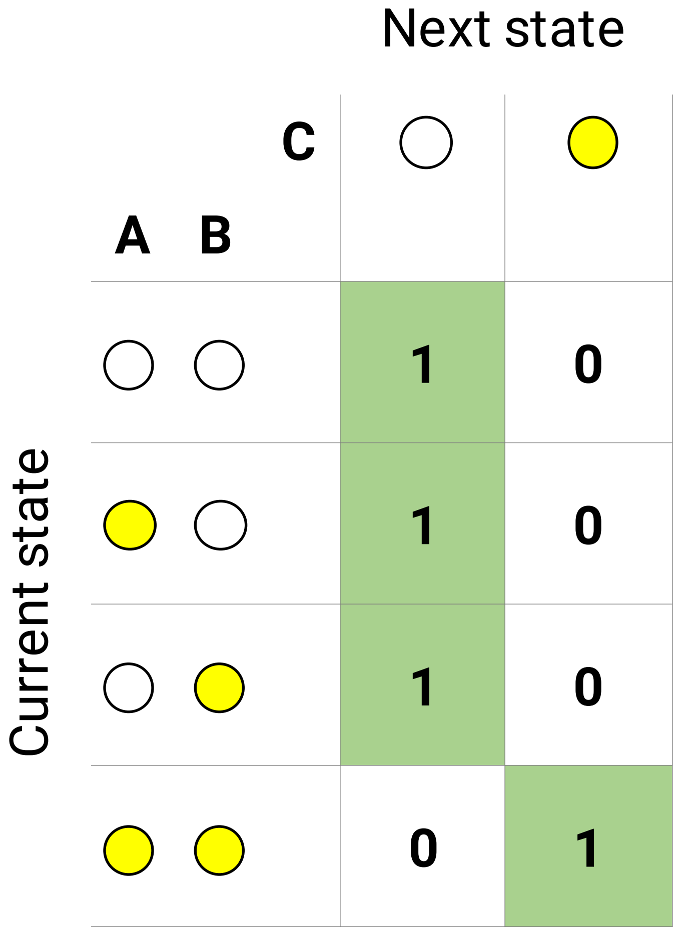

- Here we can see that **C** is in fact an AND gate
- It is on at $t+1$ when its inputs are both on at t, and off otherwise

Introduction:
Nondeterministic mechanisms

- In general, input-output functions can be nondeterministic
- For example, we could have an element with this TPM:
- Here, **C** is a noisy AND gate; its next state is somewhat uncertain (so we repeat the perturbations many times)

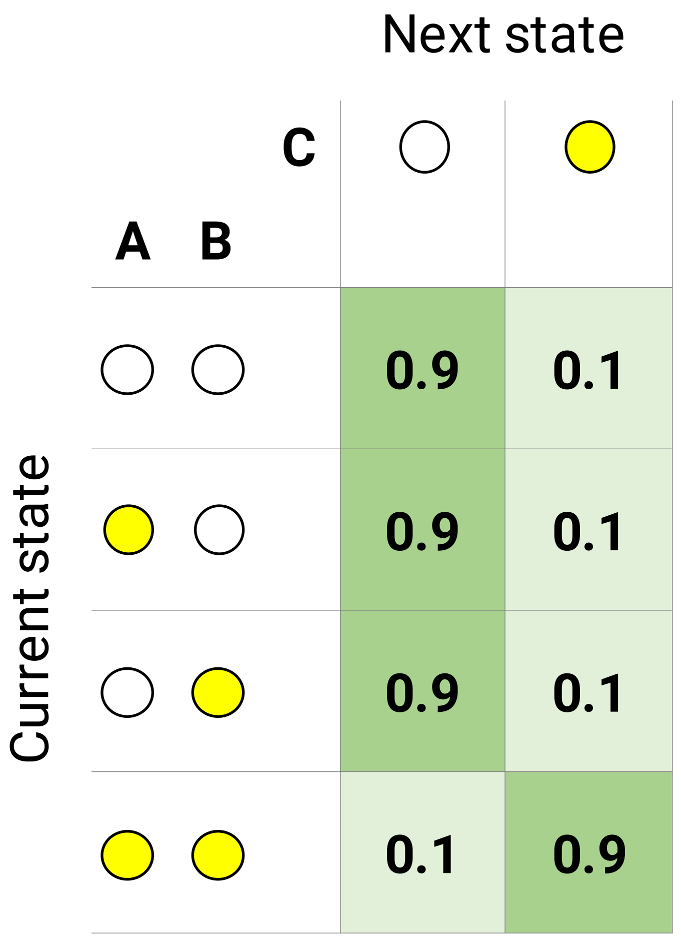

An example network

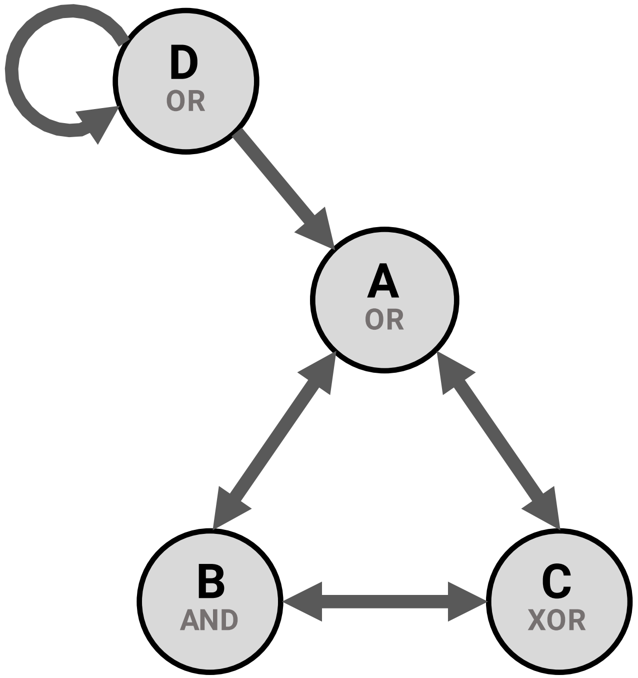

Network with 4 binary elements
($2^4 = 16$ possible states)

- Now let's consider a larger network of interconnected elements
- Just as with a single element, we can determine the TPM of the network as a whole
- Again, to do so we perturb the system into each of its possible states and record the results

An example network

t

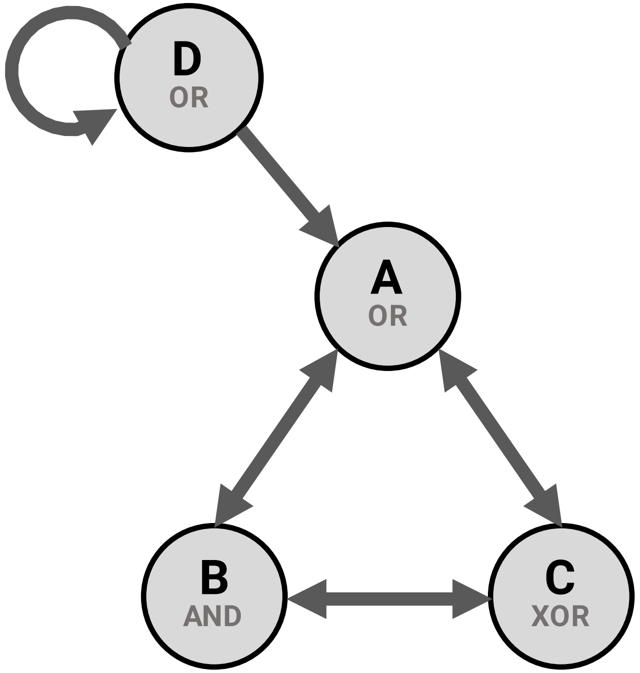

An example network

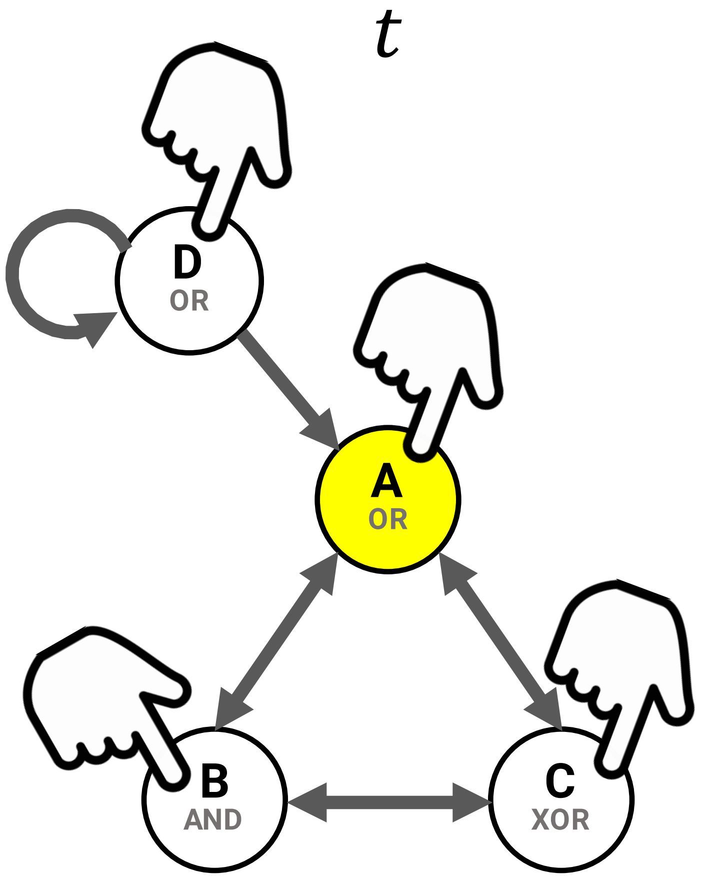

Example perturbation

An example network

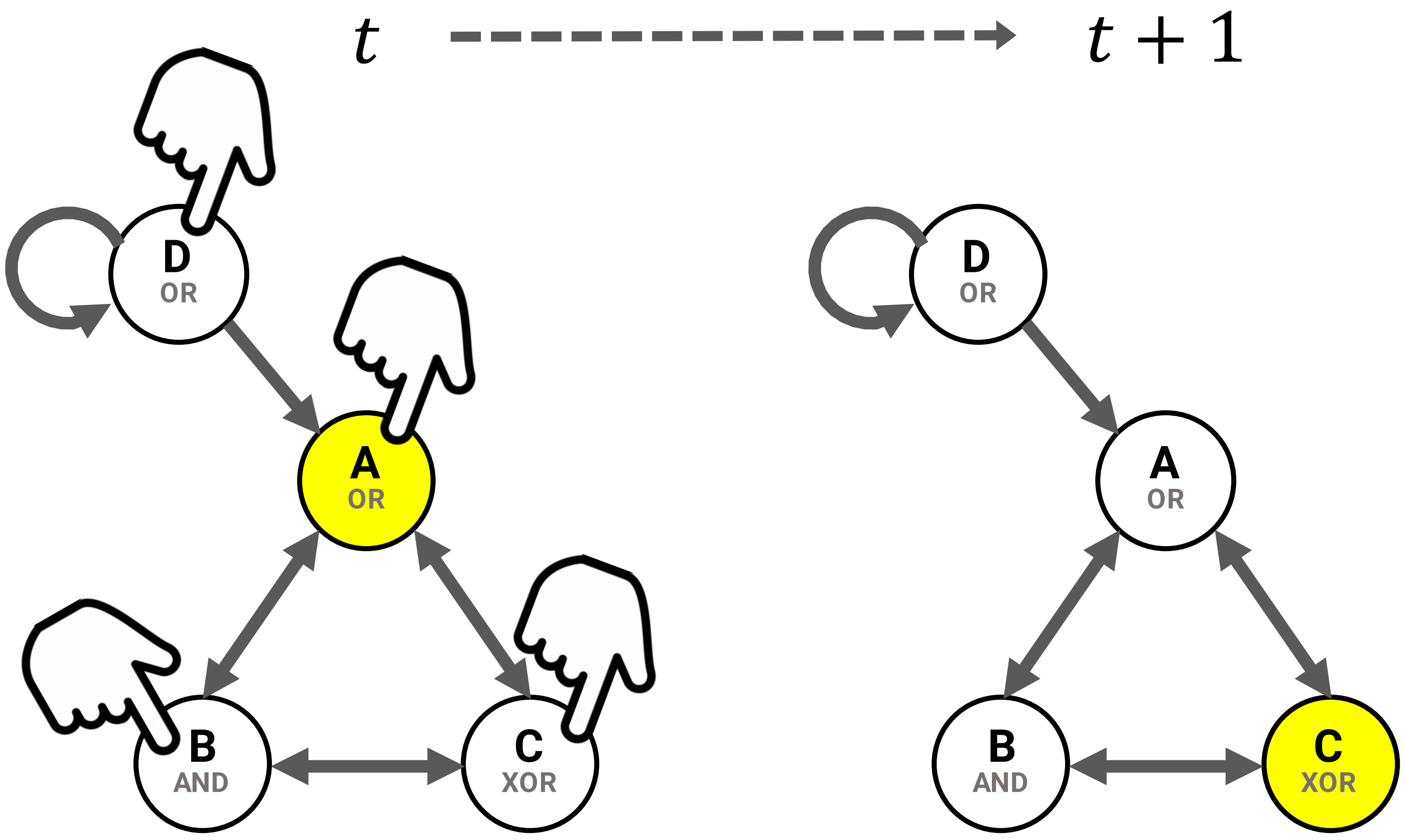

Example perturbation

Result of perturbation

An example network

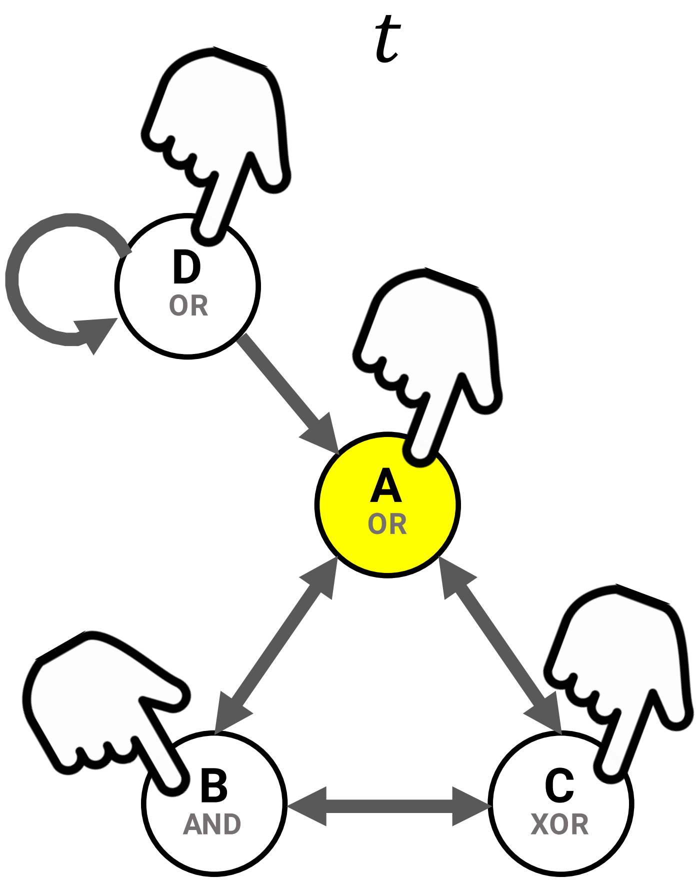

Perturbation

- Note that in this example, we're assuming that the structure of the network is as shown
- In general, we don't know the underlying structure
- Perturbation and observation is what allows the experimenter to determine the TPM

An example network

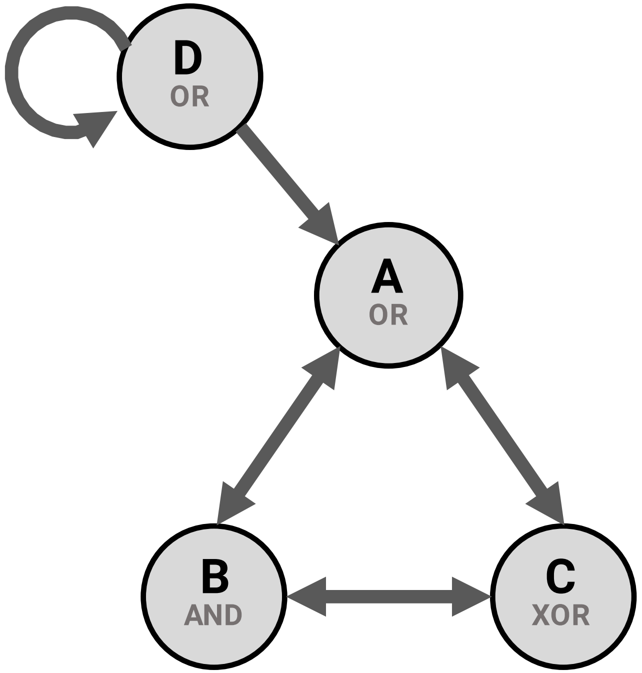

Network with 4 binary elements
($2^4 = 16$ possible states)

Next state

Current state A	B	C	D	○○○○	●○○○	○●○○	●●○○	○○●○	●○●○	○●●○	●●●○	○○○●	●○○●	○●○●	●●○●	○○●●	●○●●	○●●●	●●●●
○	○	○	○	1	0	0	0	0	0	0	0	0	0	0	0	0	0	0	0
●	○	○	○	0	0	0	0	1	0	0	0	0	0	0	0	0	0	0	0
○	●	○	○	0	0	0	0	0	1	0	0	0	0	0	0	0	0	0	0
●	●	○	○	0	1	0	0	0	0	0	0	0	0	0	0	0	0	0	0
○	○	●	○	0	1	0	0	0	0	0	0	0	0	0	0	0	0	0	0
●	○	●	○	0	0	0	0	0	0	0	1	0	0	0	0	0	0	0	0
○	●	●	○	0	0	0	0	0	1	0	0	0	0	0	0	0	0	0	0
●	●	●	○	0	0	0	1	0	0	0	0	0	0	0	0	0	0	0	0
○	○	○	●	0	0	0	0	0	0	0	0	0	1	0	0	0	0	0	0
●	○	○	●	0	0	0	0	0	0	0	0	0	0	0	0	0	1	0	0
○	●	○	●	0	0	0	0	0	0	0	0	0	0	0	0	0	1	0	0
●	●	○	●	0	0	0	0	0	0	0	0	0	1	0	0	0	0	0	0
○	○	●	●	0	0	0	0	0	0	0	0	0	1	0	0	0	0	0	0
●	○	●	●	0	0	0	0	0	0	0	0	0	0	0	0	0	0	0	1
○	●	●	●	0	0	0	0	0	0	0	0	0	0	0	0	0	1	0	0
●	●	●	●	0	0	0	0	0	0	0	0	0	0	0	1	0	0	0	0

Corresponding TPM (16×16)
Deterministic network $\Leftrightarrow$ single column with
1.0 probability in each row

Outline

- Elements, states, and the TPM
- Background conditions
- Cause-effect repertoires
- Integrated mechanisms: φ
- Concepts and cause-effect structures
- Integrated systems: Φ
- Complexes

Candidate systems and background conditions

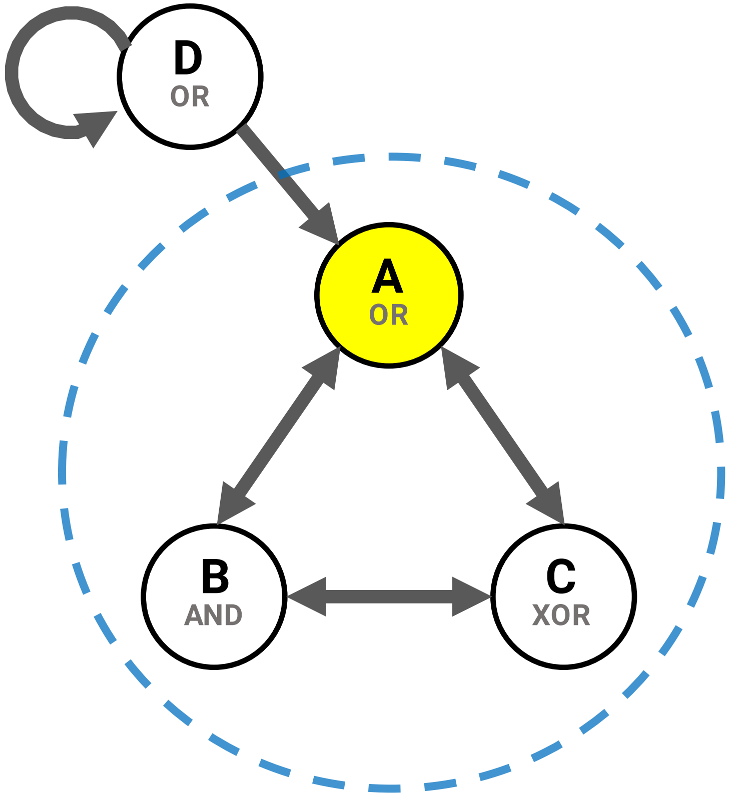

Candidate system **ABC**

- Given a network in some state at some moment in time, we want to evaluate the integrated information of a subset of its elements, called a **candidate system**

Candidate systems and background conditions

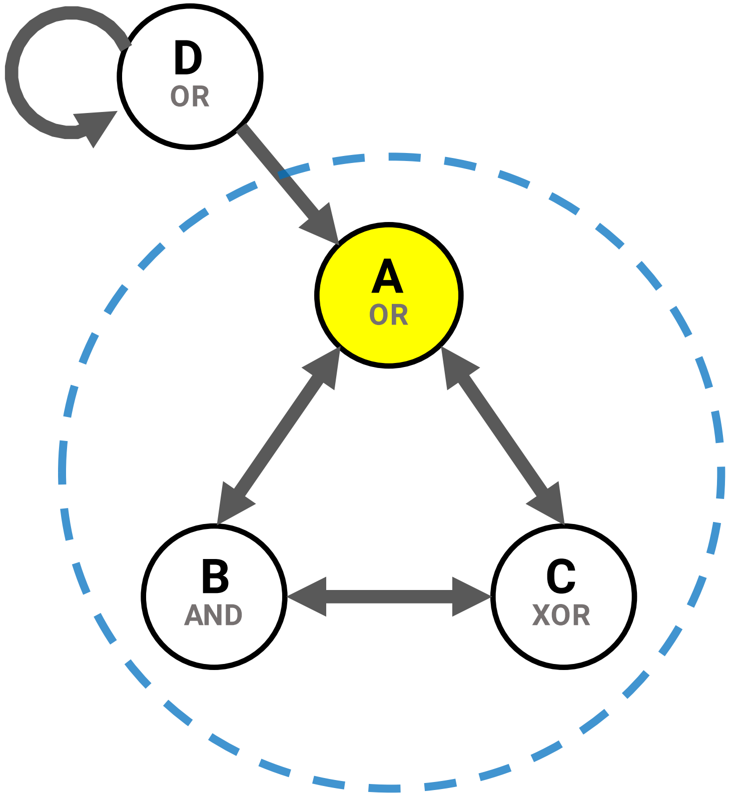

Candidate system **ABC**

- In order to do so, we use the TPM of those elements
- Since the aim is to assess the integrated information of the candidate system *when the network is in a particular state*, we want to determine the TPM of the candidate system by perturbing it while the external elements are **fixed** in that state

Candidate systems and background conditions

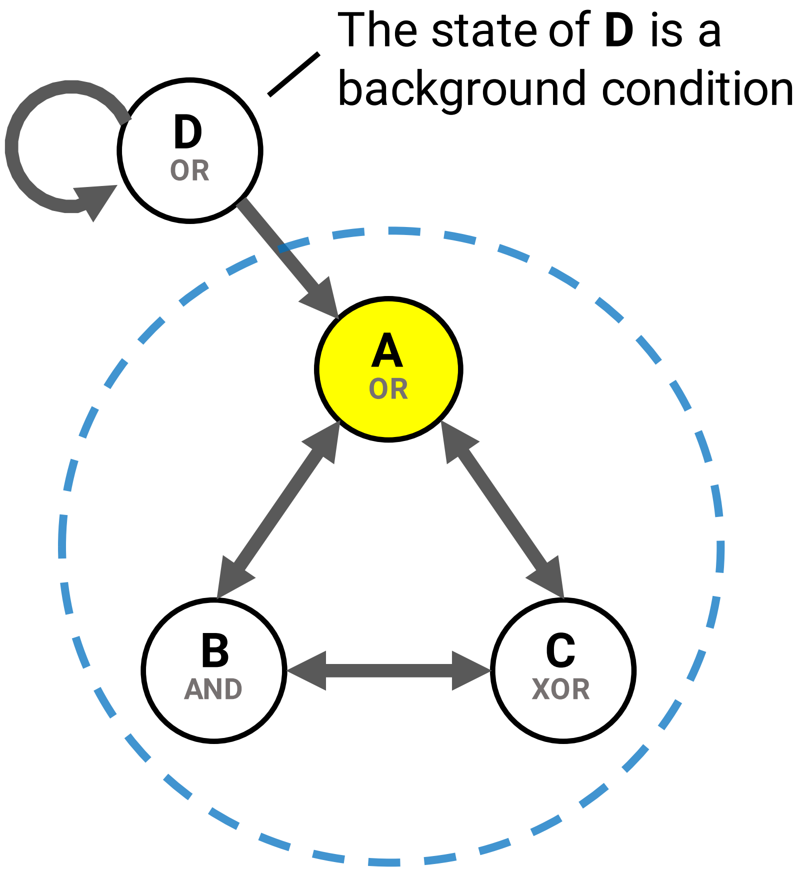

Candidate system **ABC**

- These fixed external elements constitute the **background conditions** for the candidate system
- Calculating the TPM of the candidate system **given** background conditions is a process called **conditioning** on the background conditions

Fixing background conditions

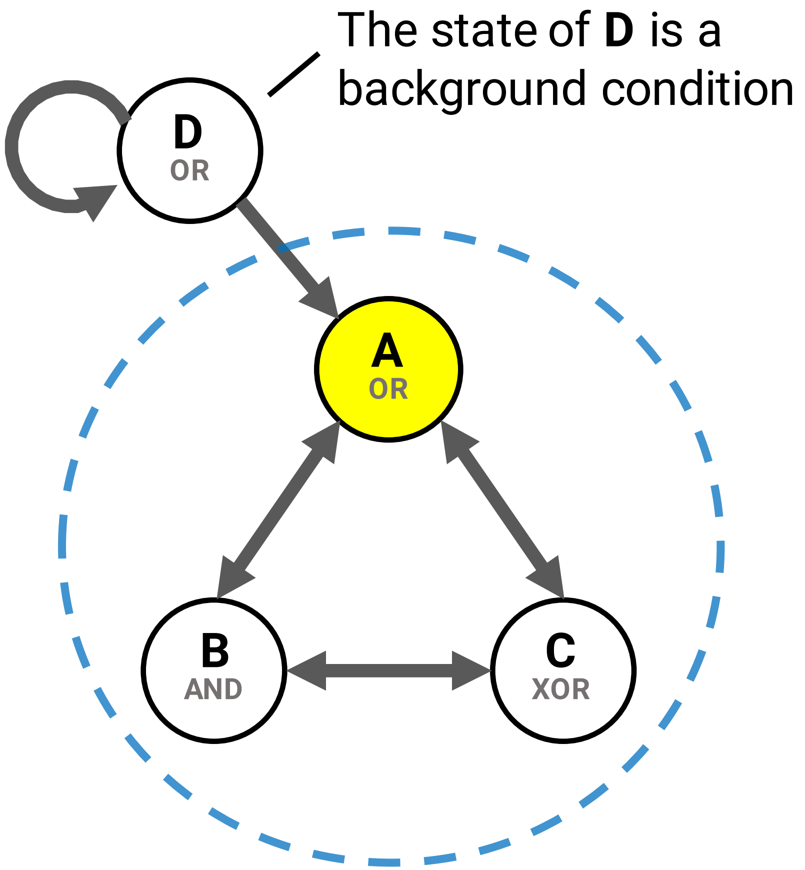

Candidate system **ABC**

Next state

				A	○	●	○	●	○	●	○	●	○	●	○	●	○	●	○	●
				B	○	○	●	●	○	○	●	●	○	○	●	●	○	○	●	●
				C	○	○	○	○	●	●	●	●	○	○	○	○	●	●	●	●
				D	○	○	○	○	○	○	○	○	●	●	●	●	●	●	●	●
A	B	C	D																	
○	○	○	○		1	0	0	0	0	0	0	0	0	0	0	0	0	0	0	0
●	○	○	○		0	0	0	0	1	0	0	0	0	0	0	0	0	0	0	0
○	●	○	○		0	0	0	0	0	1	0	0	0	0	0	0	0	0	0	0
●	●	○	○		0	1	0	0	0	0	0	0	0	0	0	0	0	0	0	0
○	○	●	○		0	1	0	0	0	0	0	0	0	0	0	0	0	0	0	0
●	○	●	○		0	0	0	0	0	0	0	1	0	0	0	0	0	0	0	0
○	●	●	○		0	0	0	0	0	1	0	0	0	0	0	0	0	0	0	0
●	●	●	○		0	0	0	1	0	0	0	0	0	0	0	0	0	0	0	0
○	○	○	●		0	0	0	0	0	0	0	0	0	1	0	0	0	0	0	0
●	○	○	●		0	0	0	0	0	0	0	0	0	0	0	0	0	1	0	0
○	●	○	●		0	0	0	0	0	0	0	0	0	0	0	0	0	1	0	0
●	●	○	●		0	0	0	0	0	0	0	0	0	1	0	0	0	0	0	0
○	○	●	●		0	0	0	0	0	0	0	0	0	1	0	0	0	0	0	0
●	○	●	●		0	0	0	0	0	0	0	0	0	0	0	0	0	0	0	1
○	●	●	●		0	0	0	0	0	0	0	0	0	0	0	0	0	1	0	0
●	●	●	●		0	0	0	0	0	0	0	0	0	0	0	1	0	0	0	0

Current state

Network TPM

Fixing background conditions

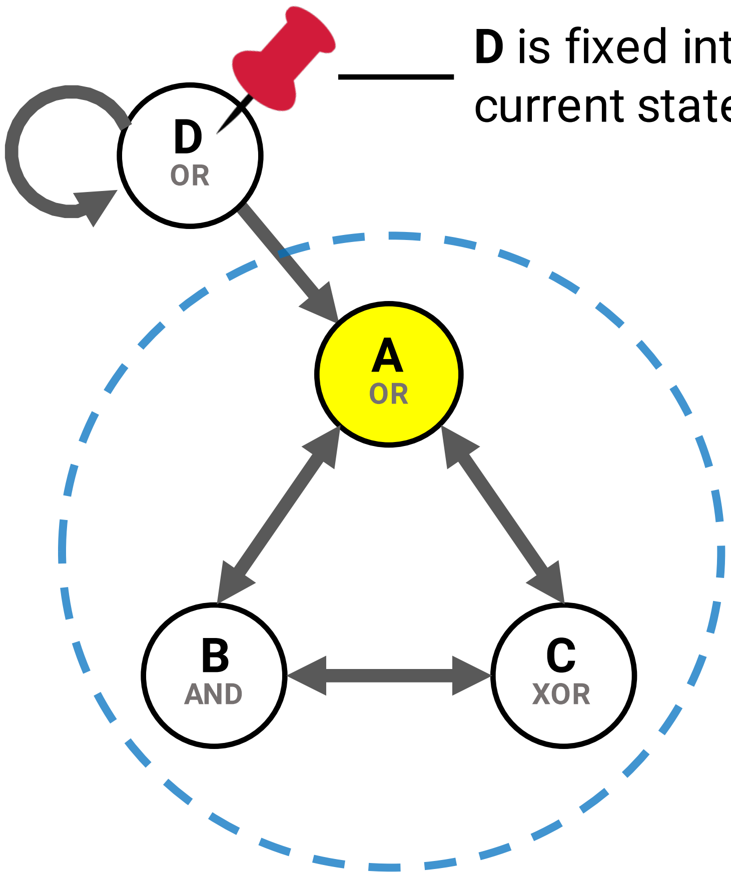

D is fixed into its current state (off)

Next state

			A	○	●	○	●	○	●	○	●	○	●	○	●	○	●	○	●
			B	○	○	●	●	○	○	●	●	○	○	●	●	○	○	●	●
			C	○	○	○	○	●	●	●	●	○	○	○	○	●	●	●	●
			D	○	○	○	○	○	○	○	○	●	●	●	●	●	●	●	●
A	B	C	D																
○	○	○	○	1	0	0	0	0	0	0	0	0	0	0	0	0	0	0	0
●	○	○	○	0	0	0	0	1	0	0	0	0	0	0	0	0	0	0	0
○	●	○	○	0	0	0	0	0	1	0	0	0	0	0	0	0	0	0	0
●	●	○	○	0	1	0	0	0	0	0	0	0	0	0	0	0	0	0	0
○	○	●	○	0	1	0	0	0	0	0	0	0	0	0	0	0	0	0	0
●	○	●	○	0	0	0	0	0	0	0	1	0	0	0	0	0	0	0	0
○	●	●	○	0	0	0	0	0	1	0	0	0	0	0	0	0	0	0	0
●	●	●	○	0	0	0	1	0	0	0	0	0	0	0	0	0	0	0	0
○	○	○	●	0	0	0	0	0	0	0	0	0	1	0	0	0	0	0	0
●	○	○	●	0	0	0	0	0	0	0	0	0	0	0	0	0	1	0	0
○	●	○	●	0	0	0	0	0	0	0	0	0	0	0	0	0	1	0	0
●	●	○	●	0	0	0	0	0	0	0	0	0	1	0	0	0	0	0	0
○	○	●	●	0	0	0	0	0	0	0	0	0	1	0	0	0	0	0	0
●	○	●	●	0	0	0	0	0	0	0	0	0	0	0	0	0	0	0	1
○	●	●	●	0	0	0	0	0	0	0	0	0	0	0	0	0	1	0	0
●	●	●	●	0	0	0	0	0	0	0	0	0	0	0	1	0	0	0	0

Current state

We **fix** the elements outside the candidate system

This corresponds to **conditioning** the TPM on the current state of **D** (off)

Fixing background conditions

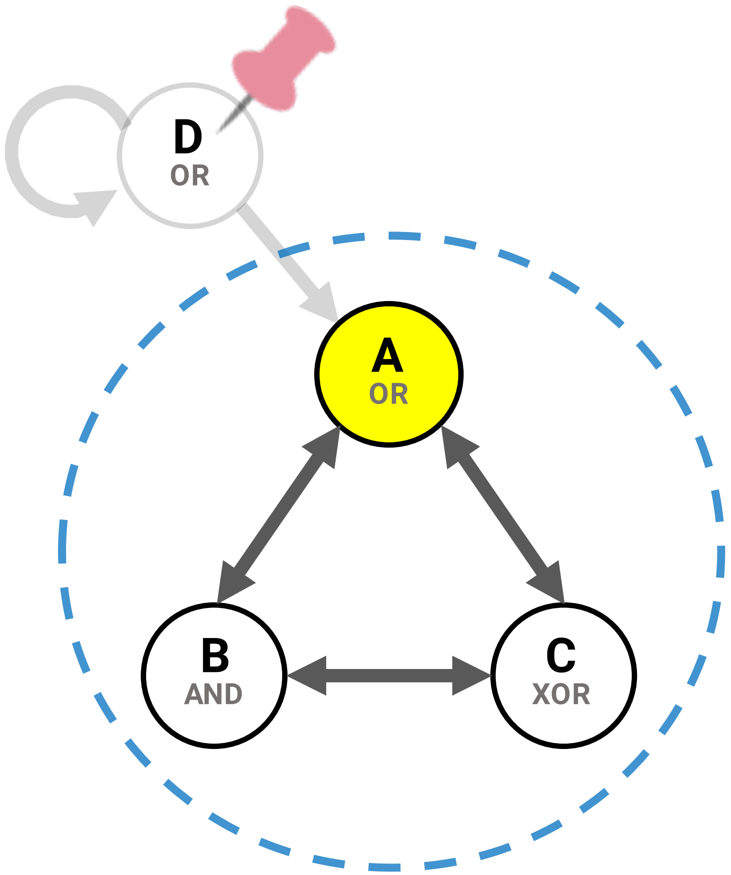

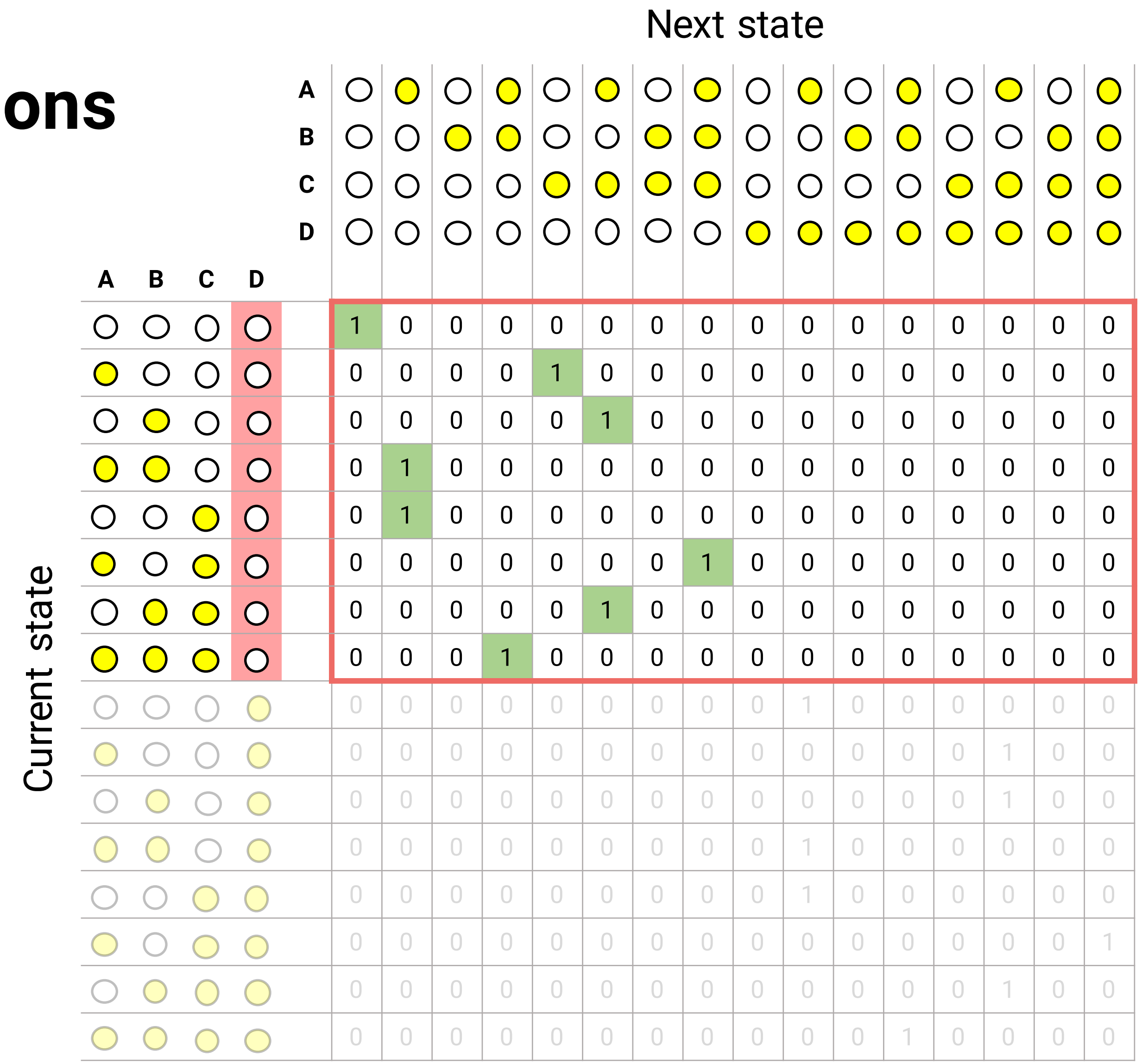

We **fix** the elements outside the candidate system

This corresponds to **conditioning** the TPM on the current state of **D** (off)

Fixing background conditions

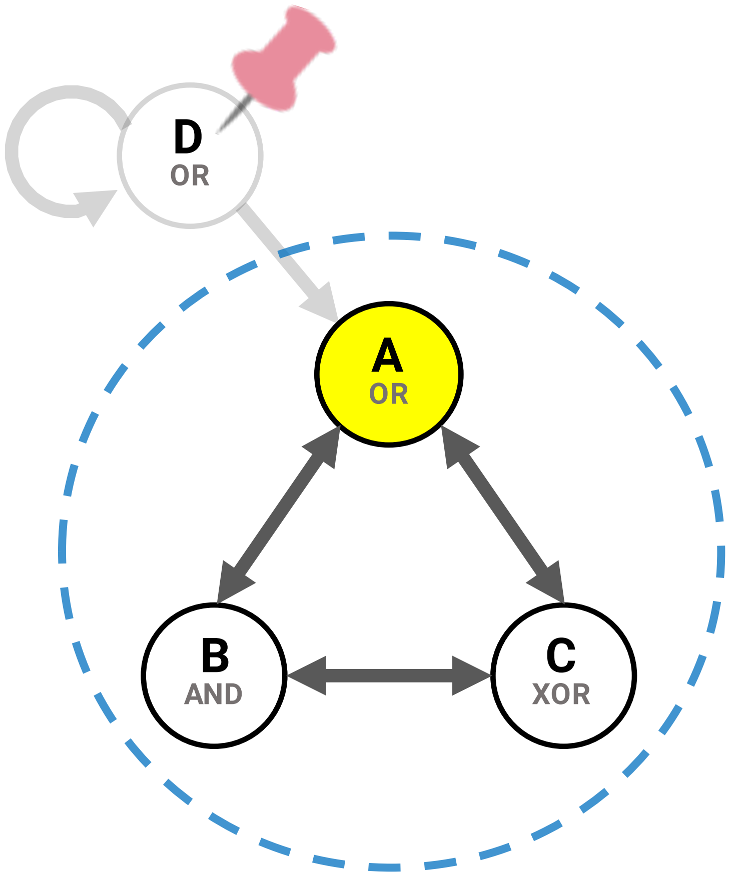

Next state (A B C D)

Current state A	B	C	D	○○○○	●○○○	○●○○	●●○○	○○●○	●○●○	○●●○	●●●○	○○○●	●○○●	○●○●	●●○●	○○●●	●○●●	○●●●	●●●●
○	○	○	○	1	0	0	0	0	0	0	0	0	0	0	0	0	0	0	0
●	○	○	○	0	0	0	0	1	0	0	0	0	0	0	0	0	0	0	0
○	●	○	○	0	0	0	0	0	1	0	0	0	0	0	0	0	0	0	0
●	●	○	○	0	1	0	0	0	0	0	0	0	0	0	0	0	0	0	0
○	○	●	○	0	1	0	0	0	0	0	0	0	0	0	0	0	0	0	0
●	○	●	○	0	0	0	0	0	0	0	1	0	0	0	0	0	0	0	0
○	●	●	○	0	0	0	0	0	1	0	0	0	0	0	0	0	0	0	0
●	●	●	○	0	0	0	1	0	0	0	0	0	0	0	0	0	0	0	0
○	○	○	●	0	0	0	0	0	0	0	0	0	1	0	0	0	0	0	0
●	○	○	●	0	0	0	0	0	0	0	0	0	0	0	0	0	1	0	0
○	●	○	●	0	0	0	0	0	0	0	0	0	0	0	0	0	1	0	0
●	●	○	●	0	0	0	0	0	0	0	0	0	1	0	0	0	0	0	0
○	○	●	●	0	0	0	0	0	0	0	0	0	1	0	0	0	0	0	0
●	○	●	●	0	0	0	0	0	0	0	0	0	0	0	0	0	0	0	1
○	●	●	●	0	0	0	0	0	0	0	0	0	0	0	0	0	1	0	0
●	●	●	●	0	0	0	0	0	0	0	0	0	0	0	1	0	0	0	0

To condition the TPM, we simply take the part of it that corresponds to the current state of **D** being off

Fixing background conditions

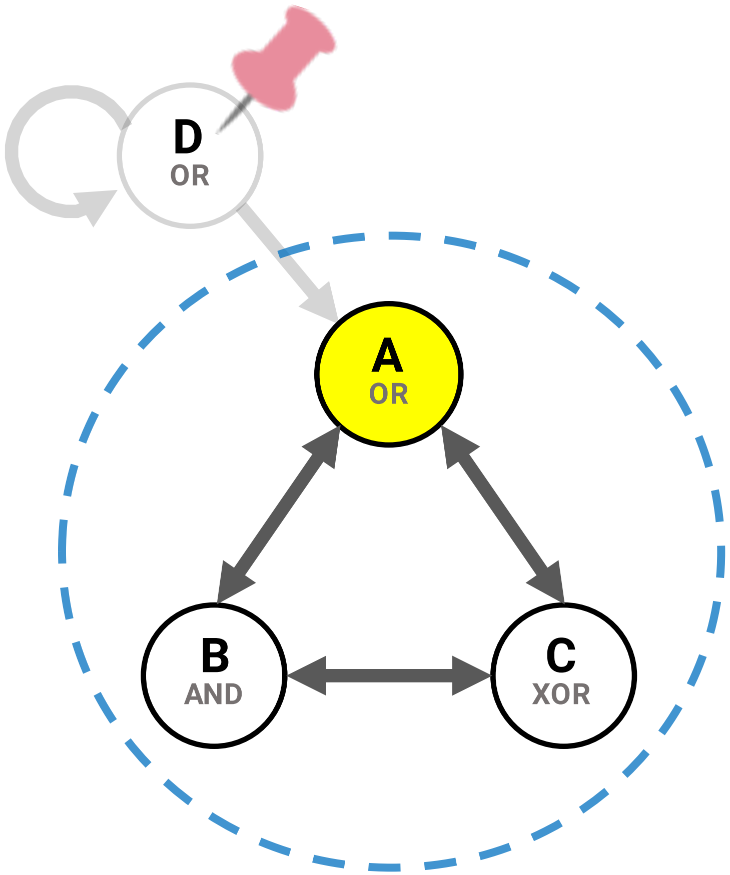

Next state

			A	○	●	○	●	○	●	○	●	○	●	○	●	○	●	○	●
			B	○	○	●	●	○	○	●	●	○	○	●	●	○	○	●	●
			C	○	○	○	○	●	●	●	●	○	○	○	○	●	●	●	●
			D	○	○	○	○	○	○	○	○	●	●	●	●	●	●	●	●
A	B	C	D																
○	○	○	○	1	0	0	0	0	0	0	0	0	0	0	0	0	0	0	0
●	○	○	○	0	0	0	0	1	0	0	0	0	0	0	0	0	0	0	0
○	●	○	○	0	0	0	0	0	1	0	0	0	0	0	0	0	0	0	0
●	●	○	○	0	1	0	0	0	0	0	0	0	0	0	0	0	0	0	0
○	○	●	○	0	1	0	0	0	0	0	0	0	0	0	0	0	0	0	0
●	○	●	○	0	0	0	0	0	0	0	1	0	0	0	0	0	0	0	0
○	●	●	○	0	0	0	0	0	1	0	0	0	0	0	0	0	0	0	0
●	●	●	○	0	0	0	1	0	0	0	0	0	0	0	0	0	0	0	0

Current state

To condition the TPM, we simply take the part of it that corresponds to the current state of **D** being off

Fixing background conditions

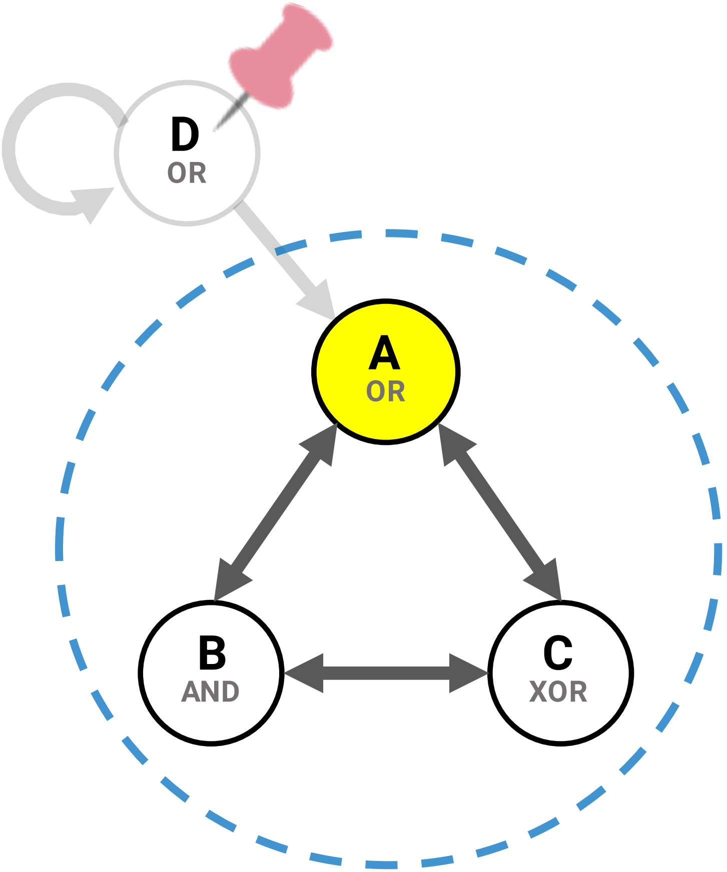

Next state (columns, each given as the states of A B C D); Current state (rows)

A	B	C	D	○○○○	●○○○	○●○○	●●○○	○○●○	●○●○	○●●○	●●●○	○○○●	●○○●	○●○●	●●○●	○○●●	●○●●	○●●●	●●●●
○	○	○	○	1	0	0	0	0	0	0	0	0	0	0	0	0	0	0	0
●	○	○	○	0	0	0	0	1	0	0	0	0	0	0	0	0	0	0	0
○	●	○	○	0	0	0	0	0	1	0	0	0	0	0	0	0	0	0	0
●	●	○	○	0	1	0	0	0	0	0	0	0	0	0	0	0	0	0	0
○	○	●	○	0	1	0	0	0	0	0	0	0	0	0	0	0	0	0	0
●	○	●	○	0	0	0	0	0	0	0	1	0	0	0	0	0	0	0	0
○	●	●	○	0	0	0	0	0	1	0	0	0	0	0	0	0	0	0	0
●	●	●	○	0	0	0	1	0	0	0	0	0	0	0	0	0	0	0	0

- However, the states at $t + 1$ still include **D**, but we're only interested in the probabilities of the states of the candidate system, **ABC**
- We would like to **ignore** the future state of **D**
- This is accomplished by **marginalization**
- We **marginalize-out** element **D** by taking the sum of the probabilities of states that differ only by **D**'s state

Fixing background conditions:
Marginalization

- Note that the first and ninth columns differ only by **D**'s state

		A	○	●	○	●	○	●	○	●	○	●	○	●	○	●	○	●
		B	○	○	●	●	○	○	●	●	○	○	●	●	○	○	●	●
		C	○	○	○	○	●	●	●	●	○	○	○	○	●	●	●	●
		D	○	○	○	○	○	○	○	○	●	●	●	●	●	●	●	●
A	B	C																
○	○	○	1	0	0	0	0	0	0	0	0	0	0	0	0	0	0	0
●	○	○	0	0	0	0	1	0	0	0	0	0	0	0	0	0	0	0
○	●	○	0	0	0	0	0	1	0	0	0	0	0	0	0	0	0	0
●	●	○	0	1	0	0	0	0	0	0	0	0	0	0	0	0	0	0
○	○	●	0	1	0	0	0	0	0	0	0	0	0	0	0	0	0	0
●	○	●	0	0	0	0	0	0	0	1	0	0	0	0	0	0	0	0
○	●	●	0	0	0	0	0	1	0	0	0	0	0	0	0	0	0	0
●	●	●	0	0	0	1	0	0	0	0	0	0	0	0	0	0	0	0

		A	○
		B	○
		C	○
A	B	C	
○	○	○	
●	○	○	
○	●	○	
●	●	○	
○	○	●	
●	○	●	
○	●	●	
●	●	●	

Fixing background conditions:

Marginalization

- Note that the first and ninth columns differ only by **D**’s state
- We sum those columns together to get the probabilities of transitioning to state **ABC** = (0, 0, 0), **ignoring the state of D**, from each previous state

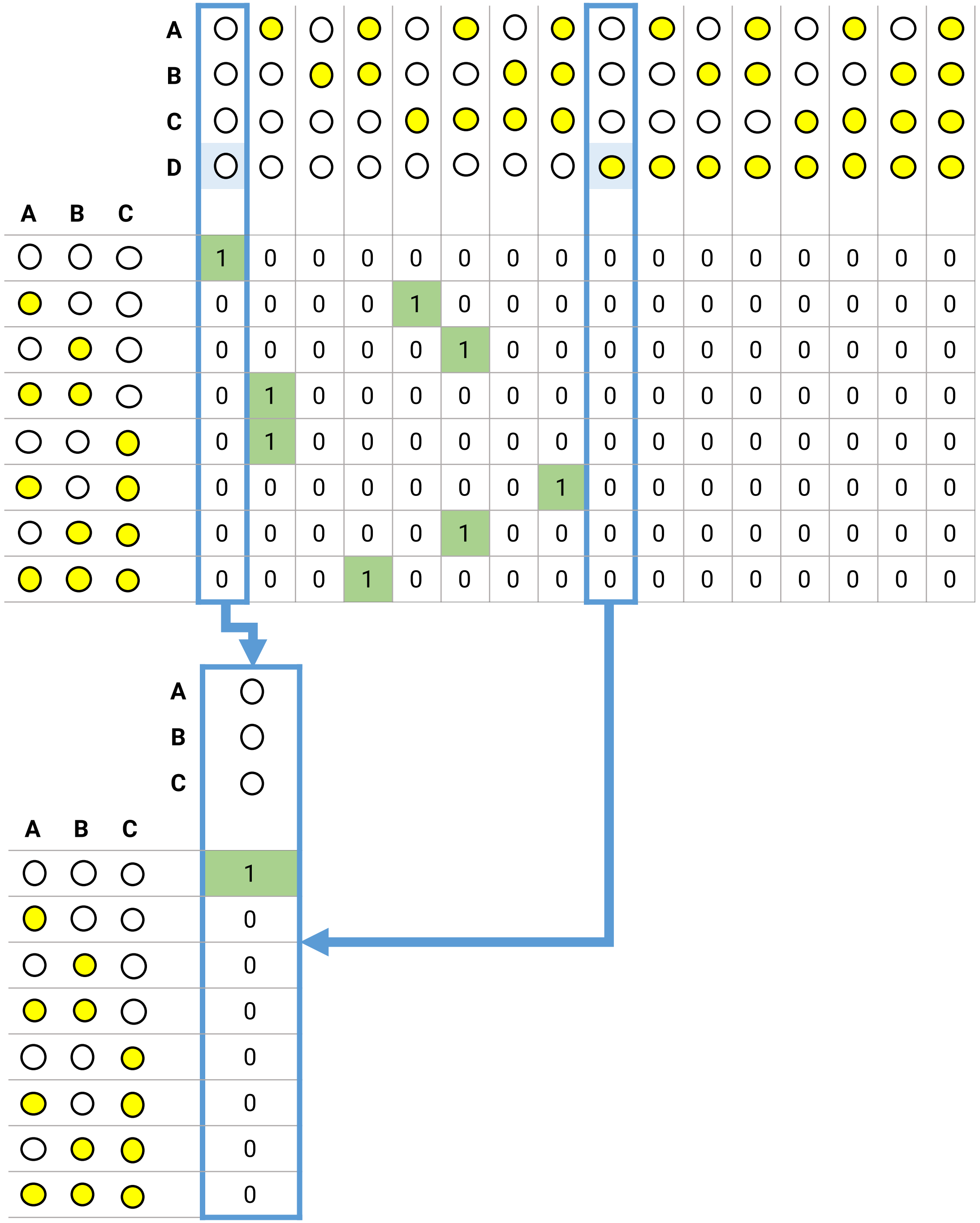

Fixing background conditions:
Marginalization

- Note that the first and ninth columns differ only by **D**'s state
- We sum those columns together to get the probabilities of transitioning to state **ABC** = (0, 0, 0), **ignoring the state of D**, from each previous state
- We repeat this for **ABC** = (1, 0, 0)…

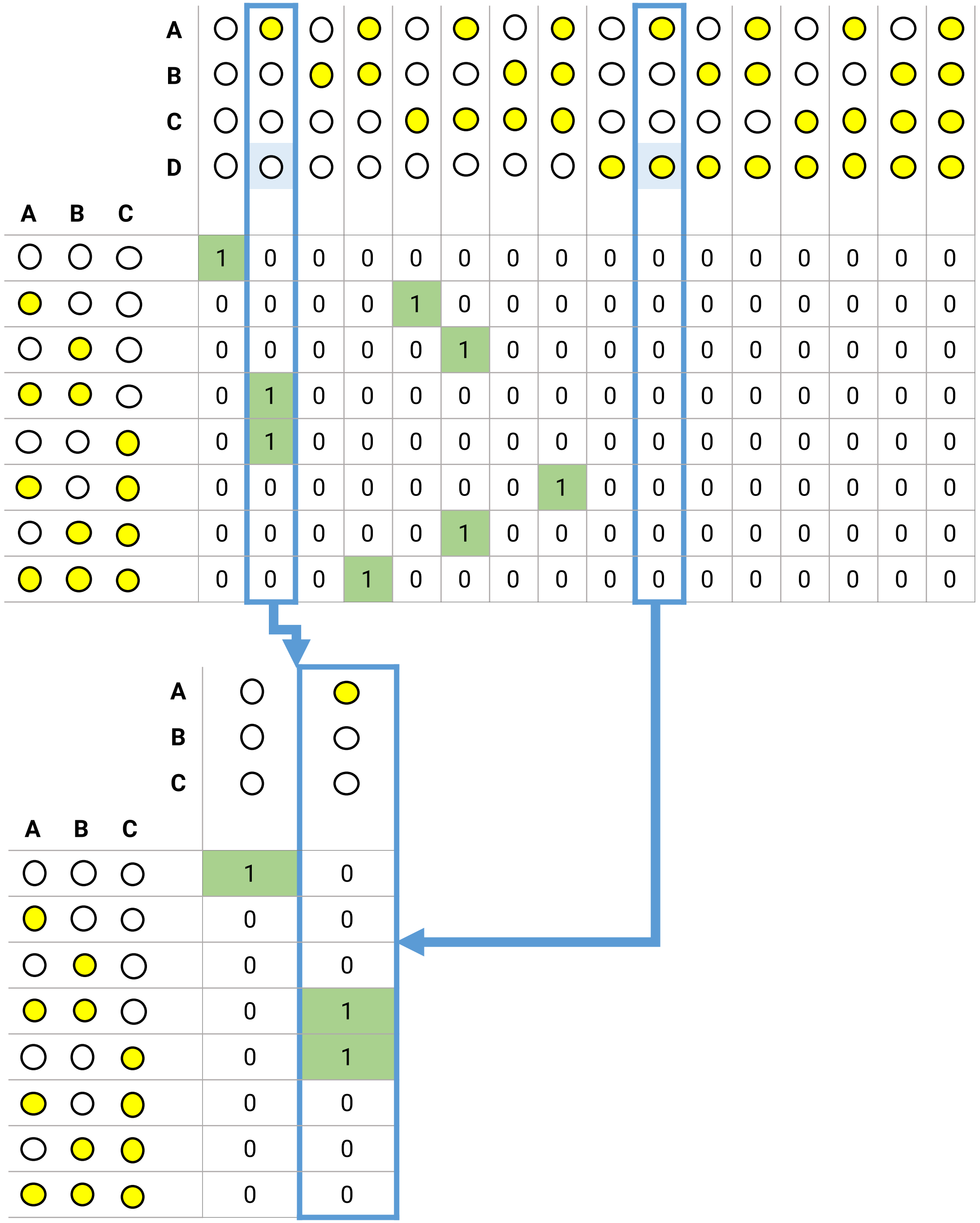

Fixing background conditions:

Marginalization

- Note that the first and ninth columns differ only by **D**'s state
- We sum those columns together to get the probabilities of transitioning to state **ABC** = (0, 0, 0), **ignoring the state of D**, from each previous state
- We repeat this for **ABC** = (1, 0, 0)…
- And so on, until we've obtained a TPM for just the elements of the candidate system **ABC**

		A	○	●	○	●	○	●	○	●	○	●	○	●	○	●	○	●
		B	○	○	●	●	○	○	●	●	○	○	●	●	○	○	●	●
		C	○	○	○	○	●	●	●	●	○	○	○	○	●	●	●	●
		D	○	○	○	○	○	○	○	○	●	●	●	●	●	●	●	●
A	B	C																
○	○	○	1	0	0	0	0	0	0	0	0	0	0	0	0	0	0	0
●	○	○	0	0	0	0	1	0	0	0	0	0	0	0	0	0	0	0
○	●	○	0	0	0	0	0	1	0	0	0	0	0	0	0	0	0	0
●	●	○	0	1	0	0	0	0	0	0	0	0	0	0	0	0	0	0
○	○	●	0	1	0	0	0	0	0	0	0	0	0	0	0	0	0	0
●	○	●	0	0	0	0	0	0	0	1	0	0	0	0	0	0	0	0
○	●	●	0	0	0	0	0	1	0	0	0	0	0	0	0	0	0	0
●	●	●	0	0	0	1	0	0	0	0	0	0	0	0	0	0	0	0

		A	○	●	○	●	○	●	○	●
		B	○	○	●	●	○	○	●	●
		C	○	○	○	○	●	●	●	●
A	B	C								
○	○	○	1	0	0	0	0	0	0	0
●	○	○	0	0	0	0	1	0	0	0
○	●	○	0	0	0	0	0	1	0	0
●	●	○	0	1	0	0	0	0	0	0
○	○	●	0	1	0	0	0	0	0	0
●	○	●	0	0	0	0	0	0	0	1
○	●	●	0	0	0	0	0	1	0	0
●	●	●	0	0	0	1	0	0	0	0

Fixing background conditions:
Marginalization

- Note that the first and ninth columns differ only by **D**'s state
- We sum those columns together to get the probabilities of transitioning to state **ABC** = (0, 0, 0), **ignoring the state of D**, from each previous state
- We repeat this for **ABC** = (1, 0, 0)…
- And so on, until we've obtained a TPM for just the elements of the candidate system **ABC**
- This TPM gives the probabilities of the state transitions of **ABC** when **D** is **fixed** in its off state in the current timestep and we **ignore** its state in the next timestep

			A	○	●	○	●	○	●	○	●	○	●	○	●	○	●	○	●
			B	○	○	●	●	○	○	●	●	○	○	●	●	○	○	●	●
			C	○	○	○	○	●	●	●	●	○	○	○	○	●	●	●	●
			D	○	○	○	○	○	○	○	○	●	●	●	●	●	●	●	●
A	B	C																	
○	○	○		1	0	0	0	0	0	0	0	0	0	0	0	0	0	0	0
●	○	○		0	0	0	0	1	0	0	0	0	0	0	0	0	0	0	0
○	●	○		0	0	0	0	0	1	0	0	0	0	0	0	0	0	0	0
●	●	○		0	1	0	0	0	0	0	0	0	0	0	0	0	0	0	0
○	○	●		0	1	0	0	0	0	0	0	0	0	0	0	0	0	0	0
●	○	●		0	0	0	0	0	0	0	1	0	0	0	0	0	0	0	0
○	●	●		0	0	0	0	0	1	0	0	0	0	0	0	0	0	0	0
●	●	●		0	0	0	1	0	0	0	0	0	0	0	0	0	0	0	0

			A	○	●	○	●	○	●	○	●
			B	○	○	●	●	○	○	●	●
			C	○	○	○	○	●	●	●	●
A	B	C									
○	○	○		1	0	0	0	0	0	0	0
●	○	○		0	0	0	0	1	0	0	0
○	●	○		0	0	0	0	0	1	0	0
●	●	○		0	1	0	0	0	0	0	0
○	○	●		0	1	0	0	0	0	0	0
●	○	●		0	0	0	0	0	0	0	1
○	●	●		0	0	0	0	0	1	0	0
●	●	●		0	0	0	1	0	0	0	0

				**A**	○	●	○	●	○	●	○	●	○	●	○	●	○	●	○	●
				**B**	○	○	●	●	○	○	●	●	○	○	●	●	○	○	●	●
				**C**	○	○	○	○	●	●	●	●	○	○	○	○	●	●	●	●
				**D**	○	○	○	○	○	○	○	○	●	●	●	●	●	●	●	●
**A**	**B**	**C**	**D**																	
○	○	○	○		1	0	0	0	0	0	0	0	0	0	0	0	0	0	0	0
●	○	○	○		0	0	0	0	1	0	0	0	0	0	0	0	0	0	0	0
○	●	○	○		0	0	0	0	0	1	0	0	0	0	0	0	0	0	0	0
●	●	○	○		0	1	0	0	0	0	0	0	0	0	0	0	0	0	0	0
○	○	●	○		0	1	0	0	0	0	0	0	0	0	0	0	0	0	0	0
●	○	●	○		0	0	0	0	0	0	0	1	0	0	0	0	0	0	0	0
○	●	●	○		0	0	0	0	0	1	0	0	0	0	0	0	0	0	0	0
●	●	●	○		0	0	0	1	0	0	0	0	0	0	0	0	0	0	0	0
○	○	○	●		0	0	0	0	0	0	0	0	0	1	0	0	0	0	0	0
●	○	○	●		0	0	0	0	0	0	0	0	0	0	0	0	0	1	0	0
○	●	○	●		0	0	0	0	0	0	0	0	0	0	0	0	0	1	0	0
●	●	○	●		0	0	0	0	0	0	0	0	0	1	0	0	0	0	0	0
○	○	●	●		0	0	0	0	0	0	0	0	0	1	0	0	0	0	0	0
●	○	●	●		0	0	0	0	0	0	0	0	0	0	0	0	0	0	0	1
○	●	●	●		0	0	0	0	0	0	0	0	0	0	0	0	0	1	0	0
●	●	●	●		0	0	0	0	0	0	0	0	0	0	0	1	0	0	0	0

Network TPM

→ Fixing background conditions

			**A**	○	●	○	●	○	●	○	●
			**B**	○	○	●	●	○	○	●	●
			**C**	○	○	○	○	●	●	●	●
**A**	**B**	**C**									
○	○	○		1	0	0	0	0	0	0	0
●	○	○		0	0	0	0	1	0	0	0
○	●	○		0	0	0	0	0	1	0	0
●	●	○		0	1	0	0	0	0	0	0
○	○	●		0	1	0	0	0	0	0	0
●	○	●		0	0	0	0	0	0	0	1
○	●	●		0	0	0	0	0	1	0	0
●	●	●		0	0	0	1	0	0	0	0

Candidate system TPM
conditioned on **D** = OFF

Fixing background conditions:

Updated from IIT 3.0

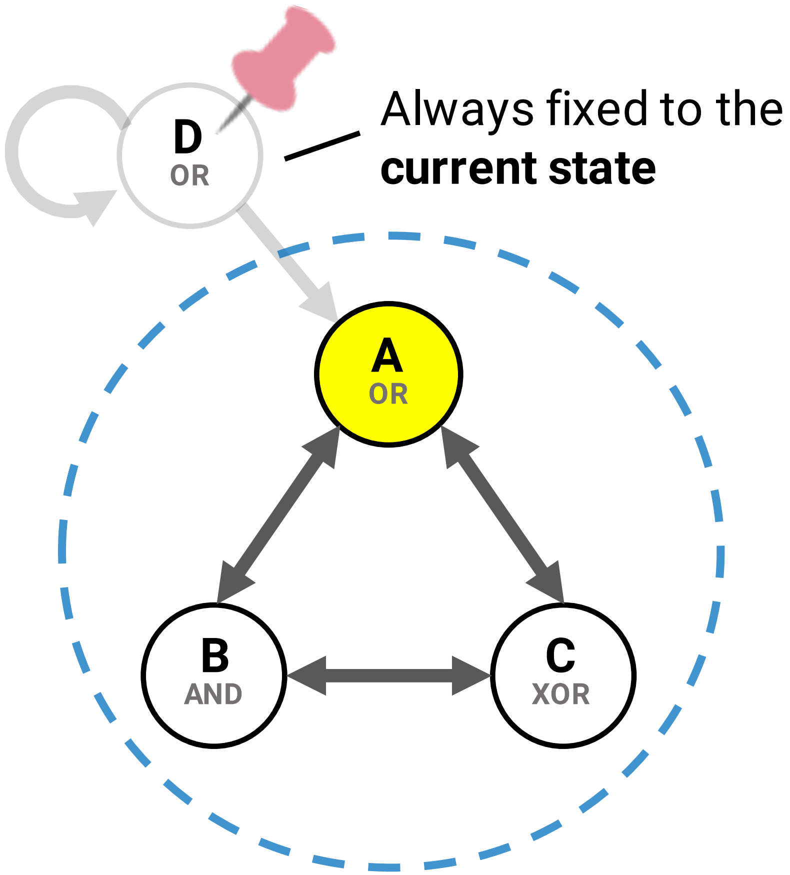

- Note that the external elements are fixed in their *current* state throughout the analysis
- This is an update to the formalism compared to IIT 3.0, where the previous state was used instead of the current state in certain parts of the analysis

Outline

- Elements, states, and the TPM
- Background conditions
- Cause-effect repertoires
- Integrated mechanisms: φ
- Concepts and cause-effect structures
- Integrated systems: Φ
- Complexes

Cause-effect repertoires

- Having chosen a candidate system and conditioned on the state of external, background elements, we now consider all subsets of the candidate system
- We call these subsets of elements **candidate mechanisms**
- We would like to evaluate the causal properties of each candidate mechanism

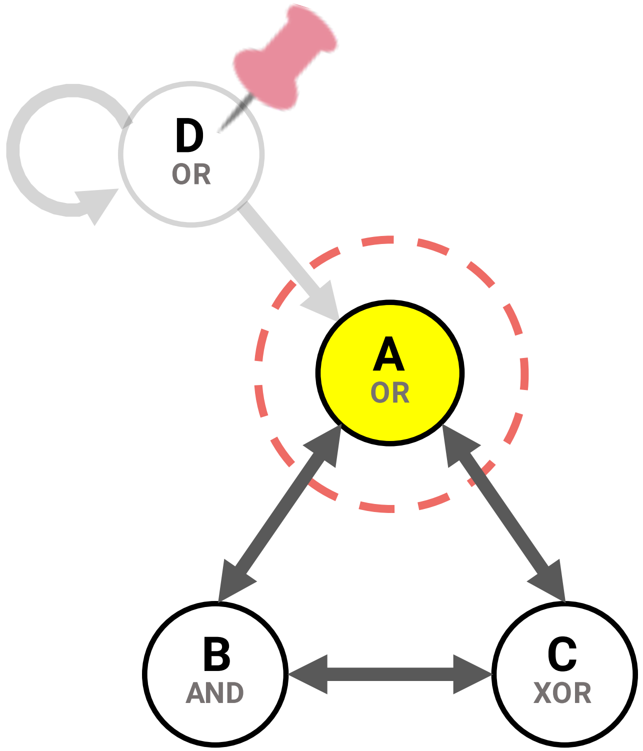

Candidate mechanism **A**

Cause-effect repertoires

- Having chosen a candidate system and conditioned on the state of external, background elements, we now consider all subsets of the candidate system
- We call these subsets of elements **candidate mechanisms**
- We would like to evaluate the causal properties of each candidate mechanism

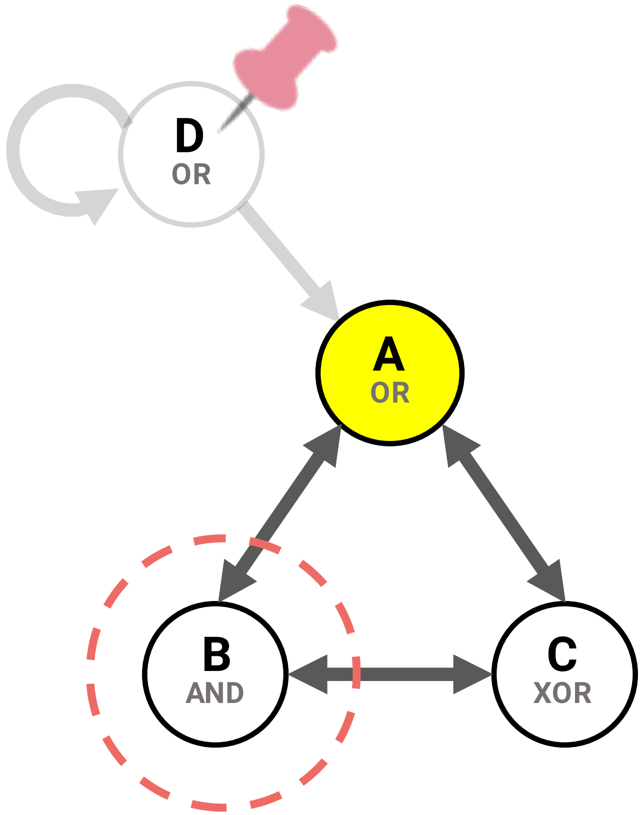

Candidate mechanism **B**

Cause-effect repertoires

- Having chosen a candidate system and conditioned on the state of external, background elements, we now consider all subsets of the candidate system
- We call these subsets of elements **candidate mechanisms**
- We would like to evaluate the causal properties of each candidate mechanism

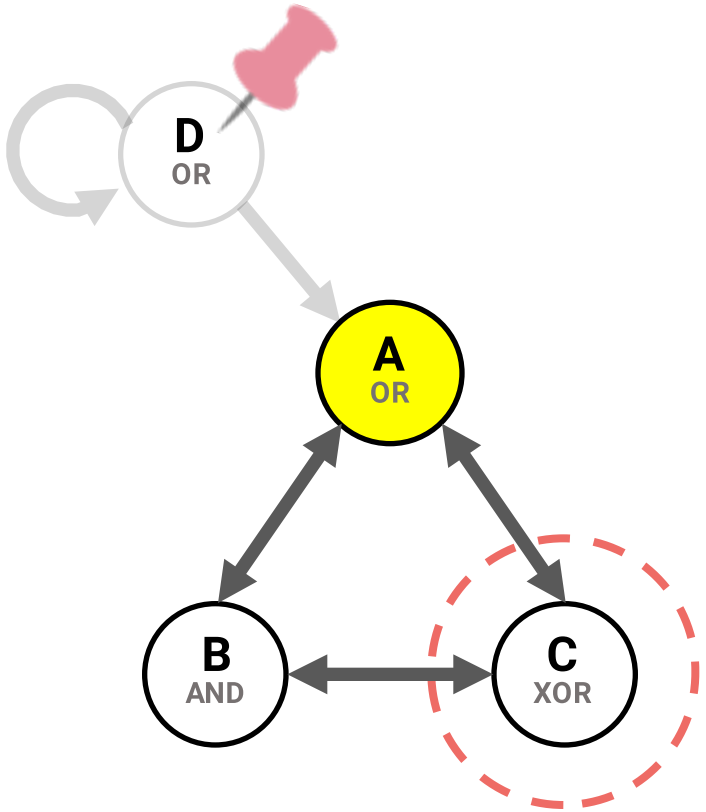

Candidate mechanism **C**

Cause-effect repertoires

- Having chosen a candidate system and conditioned on the state of external, background elements, we now consider all subsets of the candidate system
- We call these subsets of elements **candidate mechanisms**
- We would like to evaluate the causal properties of each candidate mechanism

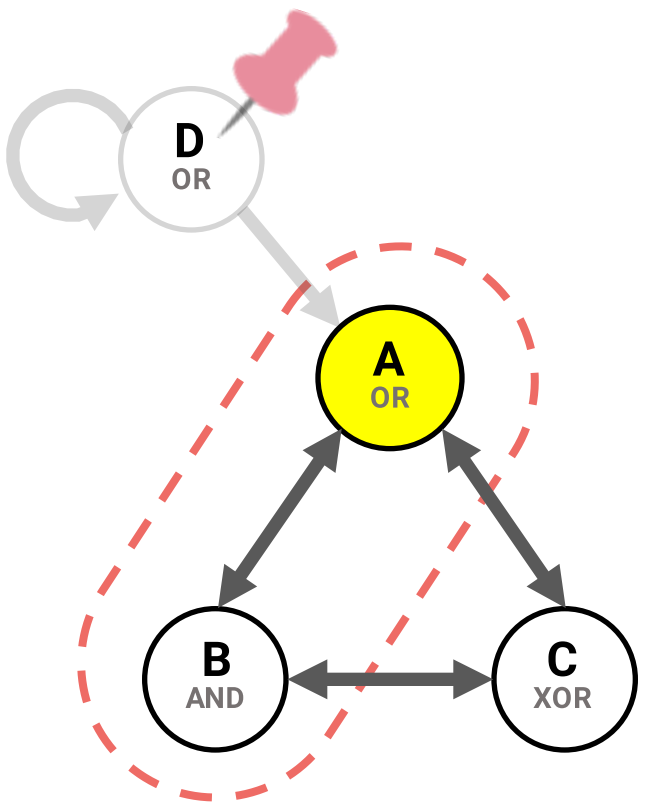

Candidate mechanism **AB**

Cause-effect repertoires

- Having chosen a candidate system and conditioned on the state of external, background elements, we now consider all subsets of the candidate system
- We call these subsets of elements **candidate mechanisms**
- We would like to evaluate the causal properties of each candidate mechanism

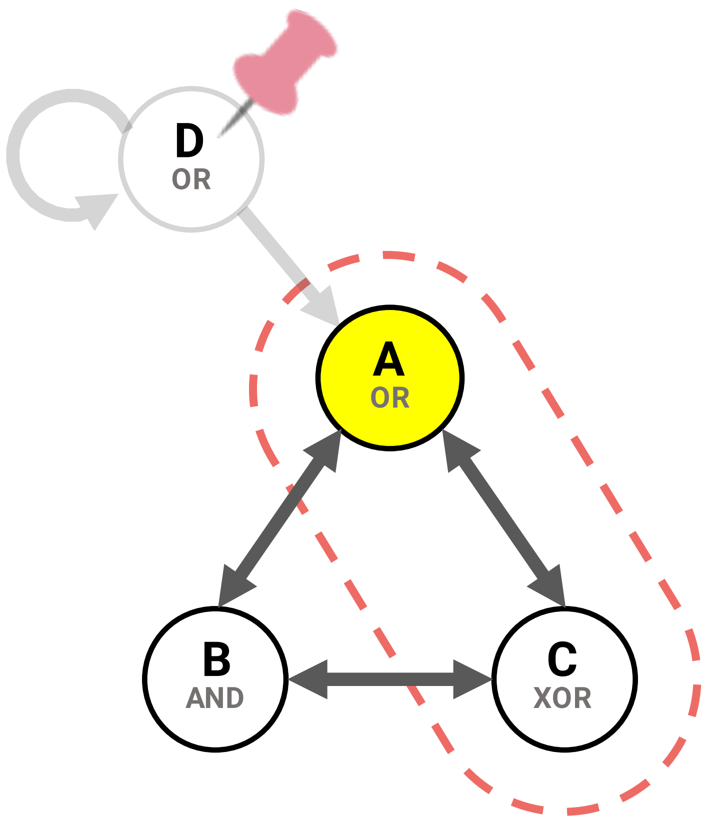

Candidate mechanism **AC**

Cause-effect repertoires

- Having chosen a candidate system and conditioned on the state of external, background elements, we now consider all subsets of the candidate system
- We call these subsets of elements **candidate mechanisms**
- We would like to evaluate the causal properties of each candidate mechanism

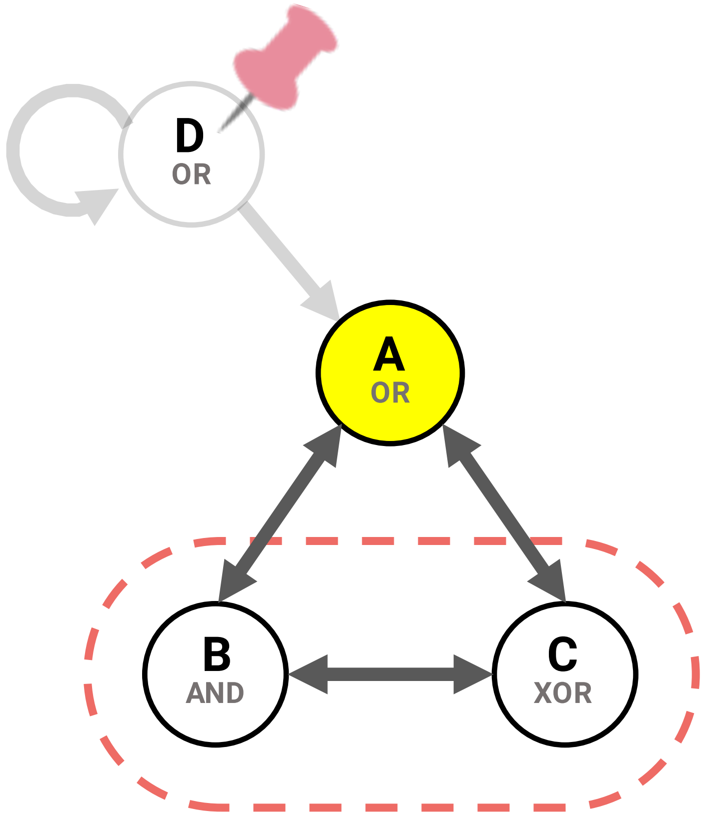

Candidate mechanism **BC**

Cause-effect repertoires

- Having chosen a candidate system and conditioned on the state of external, background elements, we now consider all subsets of the candidate system
- We call these subsets of elements **candidate mechanisms**
- We would like to evaluate the causal properties of each candidate mechanism

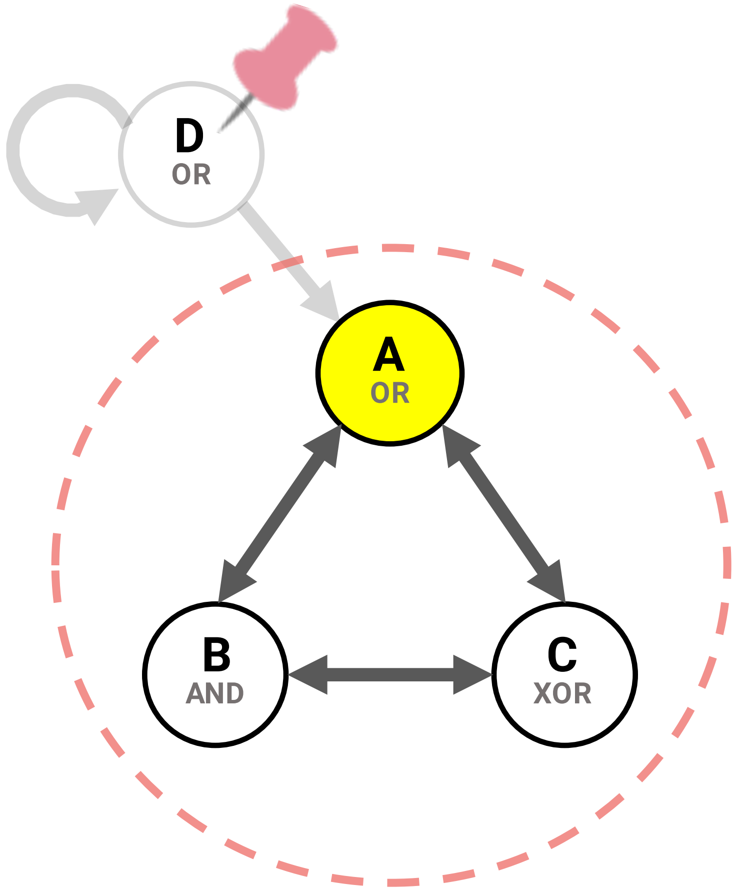

Candidate mechanism **ABC**

Cause-effect repertoires

- The notion of “causal properties” is made precise with the **cause repertoire and effect repertoire** of a candidate mechanism
- These repertoires are probability distributions over states of the system at $t-1$ and $t+1$, respectively
- They describe how the mechanism in its current state at t causally constrains the other elements
- First we’ll focus on the effect repertoire

Calculating an effect repertoire:

Conditioning on the mechanism ABC

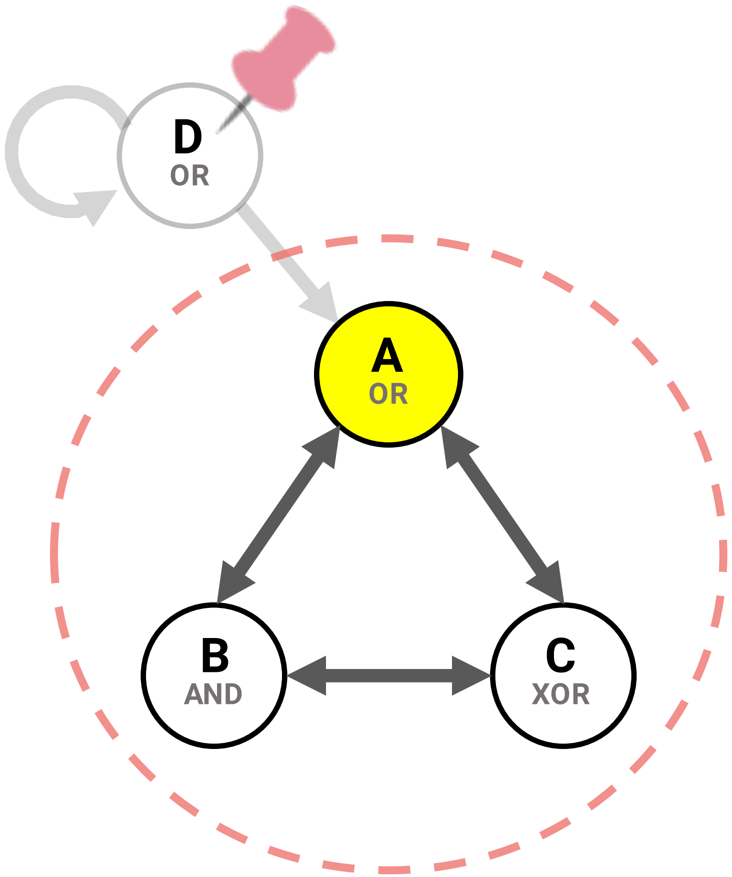

Next state

Current state A	Current state B	Current state C								
			A	○	●	○	●	○	●	○	●
			B	○	○	●	●	○	○	●	●
			C	○	○	○	○	●	●	●	●
○	○	○		1	0	0	0	0	0	0	0
●	○	○		0	0	0	0	1	0	0	0
○	●	○		0	0	0	0	0	1	0	0
●	●	○		0	1	0	0	0	0	0	0
○	○	●		0	1	0	0	0	0	0	0
●	○	●		0	0	0	0	0	0	0	1
○	●	●		0	0	0	0	0	1	0	0
●	●	●		0	0	0	1	0	0	0	0

For example, let's calculate the effect repertoire of the candidate mechanism **ABC**

Calculating an effect repertoire:
Conditioning on the mechanism ABC

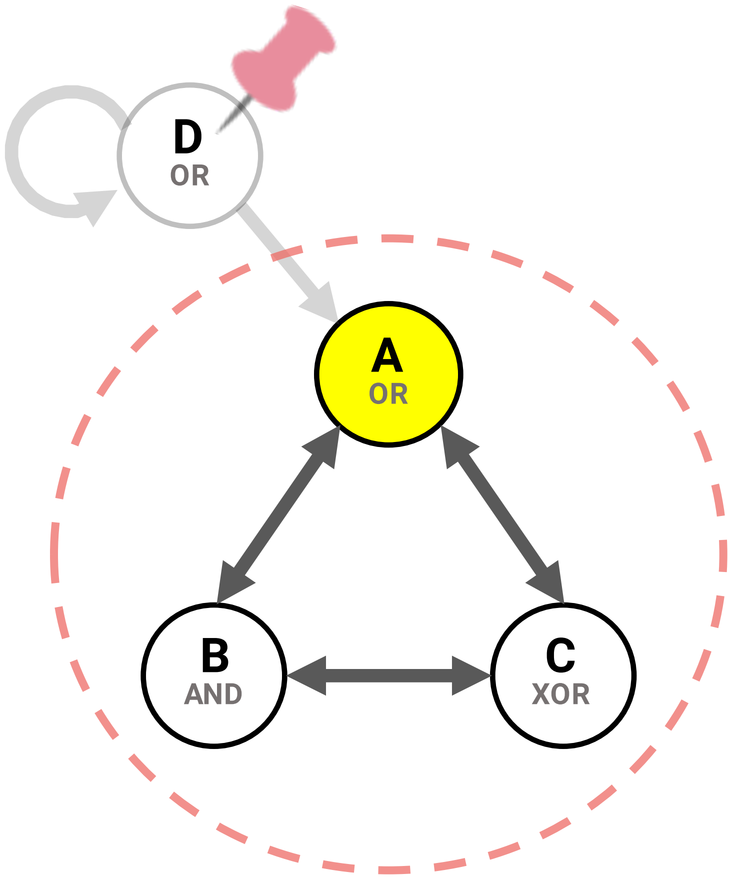

We want to determine how the current state of **ABC** constrains the next state…

Next state

		A	○	●	○	●	○	●	○	●
		B	○	○	●	●	○	○	●	●
		C	○	○	○	○	●	●	●	●
A	B	C								
○	○	○	1	0	0	0	0	0	0	0
●	○	○	0	0	0	0	1	0	0	0
○	●	○	0	0	0	0	0	1	0	0
●	●	○	0	1	0	0	0	0	0	0
○	○	●	0	1	0	0	0	0	0	0
●	○	●	0	0	0	0	0	0	0	1
○	●	●	0	0	0	0	0	1	0	0
●	●	●	0	0	0	1	0	0	0	0

Current state

Calculating an effect repertoire:

Conditioning on the mechanism ABC

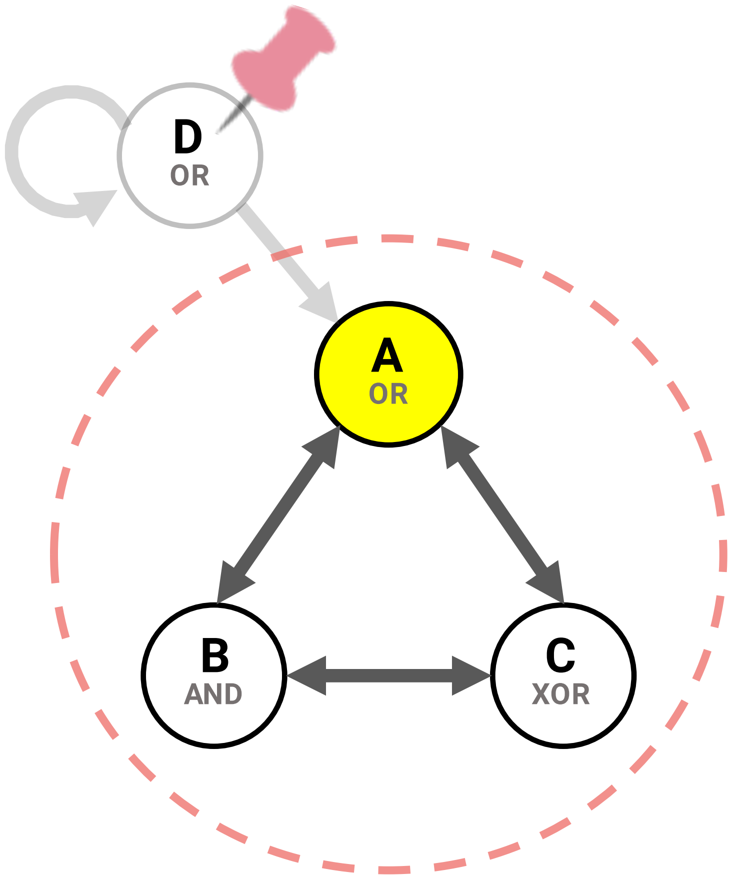

Next state (columns, A B C); Current state (rows, A B C)

A	B	C	○○○	●○○	○●○	●●○	○○●	●○●	○●●	●●●
○	○	○	1	0	0	0	0	0	0	0
●	○	○	0	0	0	0	1	0	0	0
○	●	○	0	0	0	0	0	1	0	0
●	●	○	0	1	0	0	0	0	0	0
○	○	●	0	1	0	0	0	0	0	0
●	○	●	0	0	0	0	0	0	0	1
○	●	●	0	0	0	0	0	1	0	0
●	●	●	0	0	0	1	0	0	0	0

We want to determine how the current state of **ABC** constrains the next state…

…so, as with background conditions, we condition on the current state by looking at the rows of the TPM that correspond to it

Calculating an effect repertoire:

Conditioning on the mechanism ABC

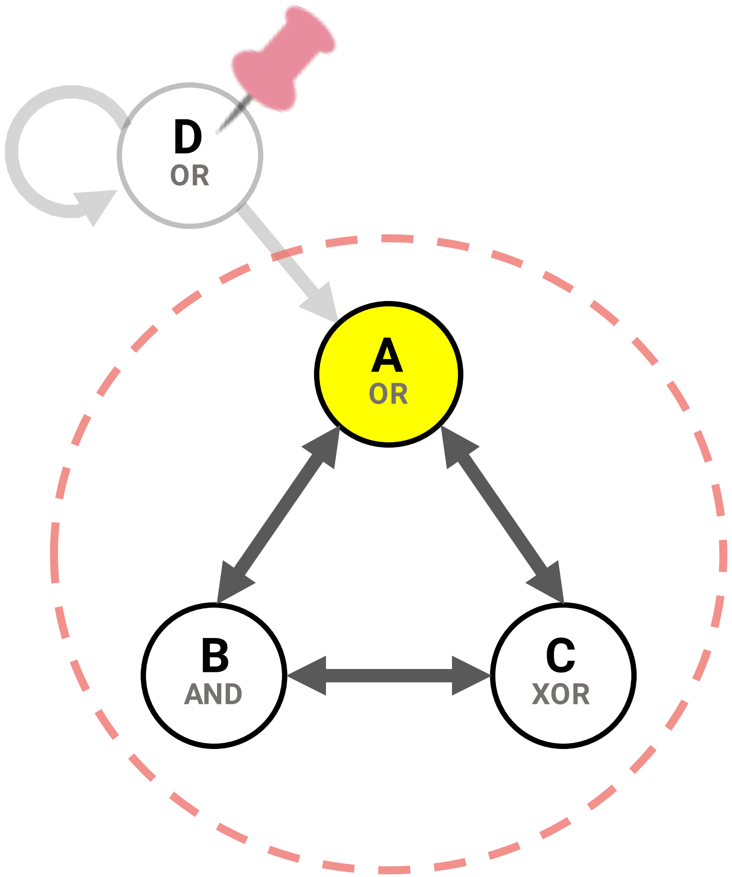

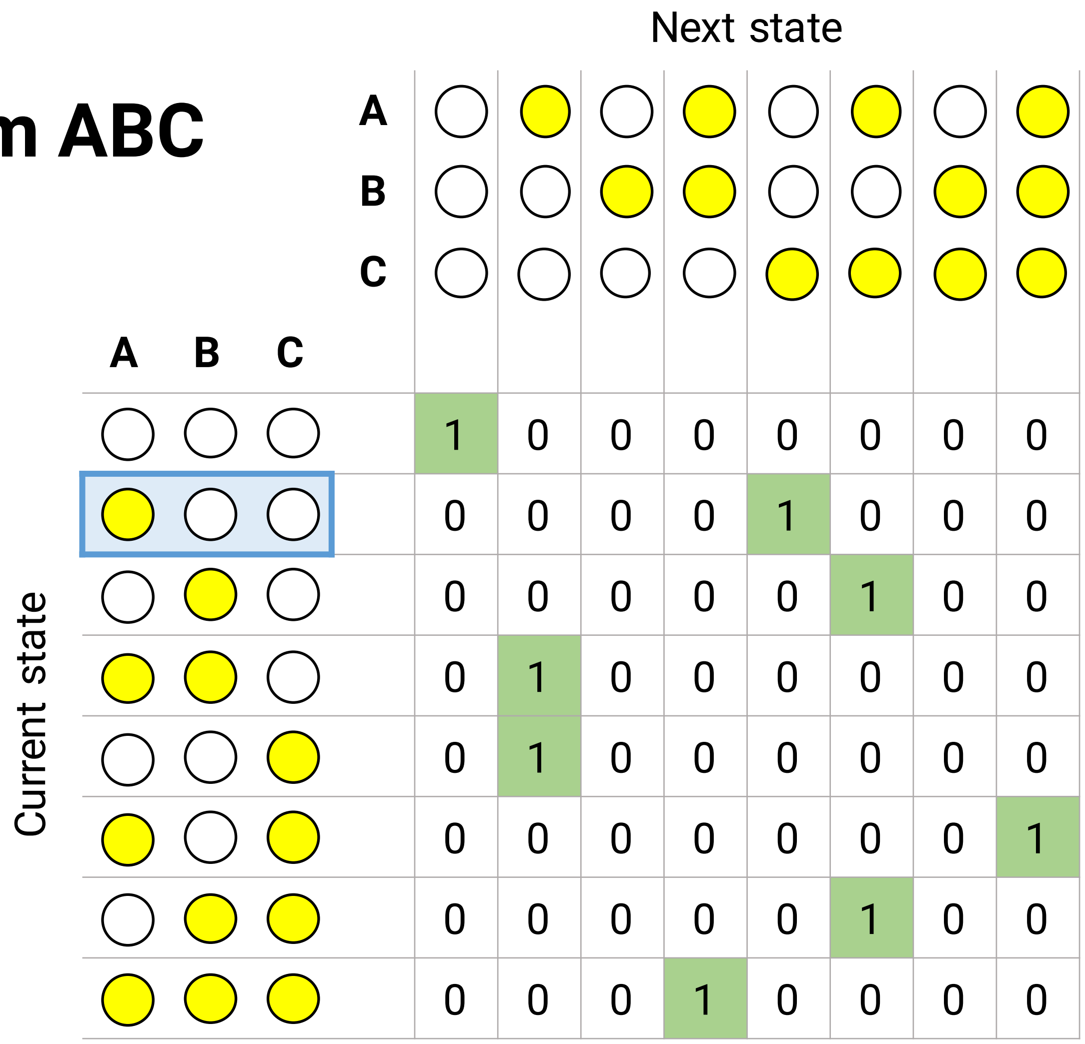

We want to determine how the current state of **ABC** constrains the next state…

…so, as with background conditions, we condition on the current state by looking at the rows of the TPM that correspond to it

Calculating an effect repertoire:

Conditioning on the mechanism ABC

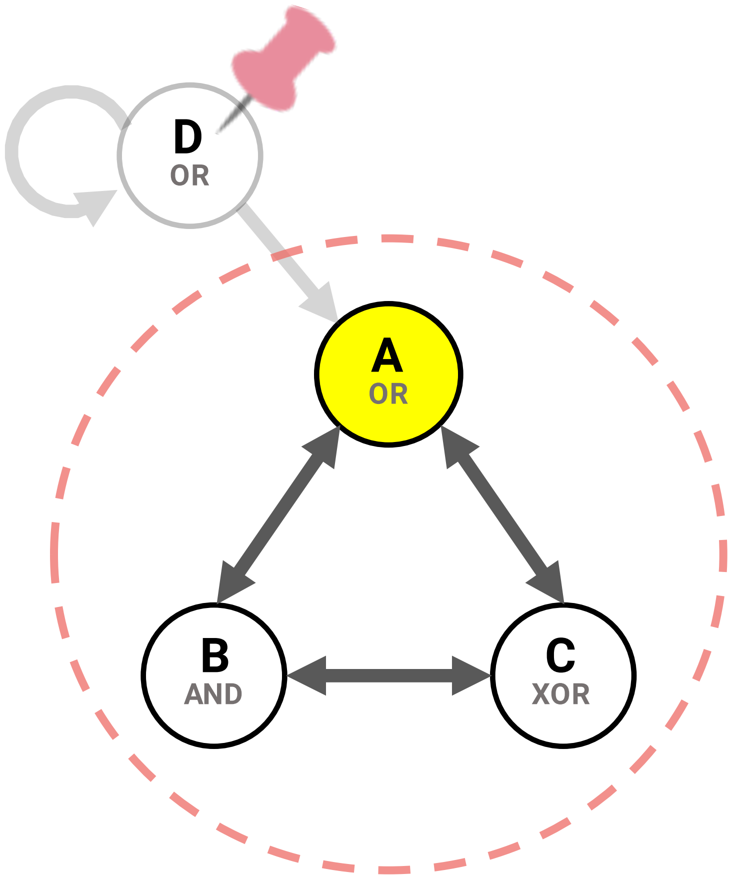

Next state

			A	○	●	○	●	○	●	○	●
			B	○	○	●	●	○	○	●	●
			C	○	○	○	○	●	●	●	●
A	B	C									
○	○	○		1	0	0	0	0	0	0	0
●	○	○		0	0	0	0	1	0	0	0
○	●	○		0	0	0	0	0	1	0	0
●	●	○		0	1	0	0	0	0	0	0
○	○	●		0	1	0	0	0	0	0	0
●	○	●		0	0	0	0	0	0	0	1
○	●	●		0	0	0	0	0	1	0	0
●	●	●		0	0	0	1	0	0	0	0

Current state

We want to determine how the current state of **ABC** constrains the next state…

…so, as with background conditions, we condition on the current state by looking at the rows of the TPM that correspond to it

Calculating an effect repertoire:

Conditioning on the mechanism ABC

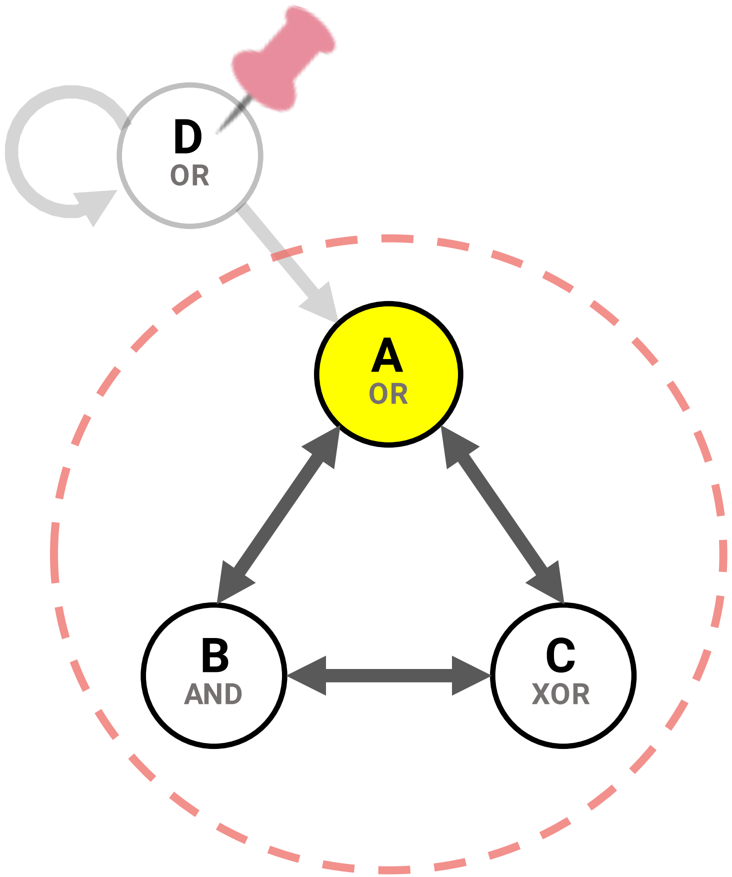

Next state

Current state										
		A	○	●	○	●	○	●	○	●
		B	○	○	●	●	○	○	●	●
		C	○	○	○	○	●	●	●	●
A	B	C								
○	○	○	1	0	0	0	0	0	0	0
●	○	○	0	0	0	0	1	0	0	0
○	●	○	0	0	0	0	0	1	0	0
●	●	○	0	1	0	0	0	0	0	0
○	○	●	0	1	0	0	0	0	0	0
●	○	●	0	0	0	0	0	0	0	1
○	●	●	0	0	0	0	0	1	0	0
●	●	●	0	0	0	1	0	0	0	0

We want to determine how the current state of **ABC** constrains the next state…

…so, as with background conditions, we condition on the current state by looking at the rows of the TPM that correspond to it

Calculating an effect repertoire:

Conditioning on the mechanism ABC

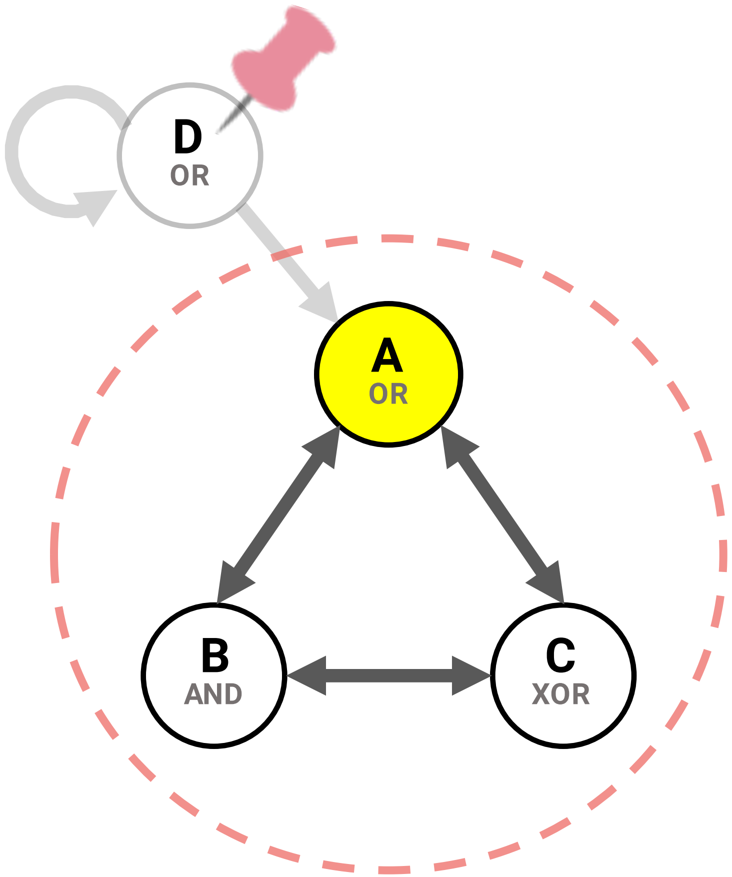

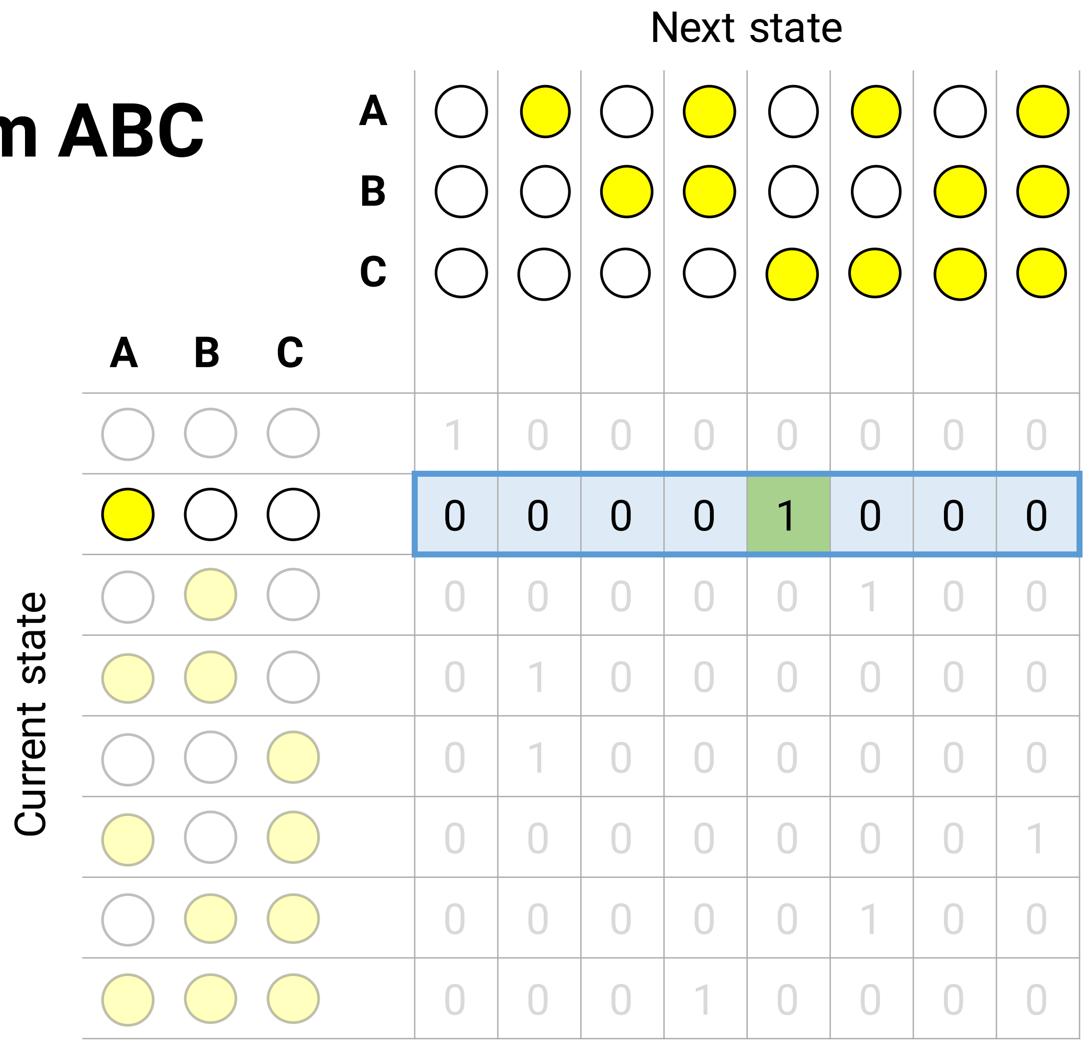

This row in the TPM is a distribution over the states at $t + 1$ (and since this is a deterministic system, the next state is fully specified by the current state)

Calculating an effect repertoire:

Conditioning on the mechanism ABC

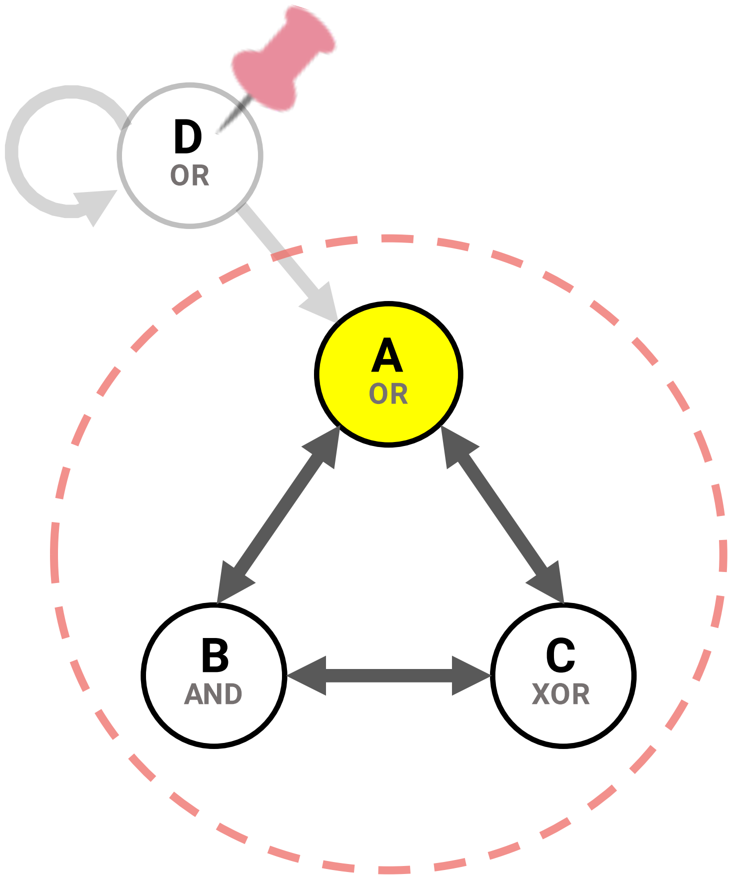

Next state

Current state A	B	C	A=0 B=0 C=0	A=1 B=0 C=0	A=0 B=1 C=0	A=1 B=1 C=0	A=0 B=0 C=1	A=1 B=0 C=1	A=0 B=1 C=1	A=1 B=1 C=1
0	0	0	1	0	0	0	0	0	0	0
1	0	0	0	0	0	0	1	0	0	0
0	1	0	0	0	0	0	0	1	0	0
1	1	0	0	1	0	0	0	0	0	0
0	0	1	0	1	0	0	0	0	0	0
1	0	1	0	0	0	0	0	0	0	1
0	1	1	0	0	0	0	0	1	0	0
1	1	1	0	0	0	1	0	0	0	0

This is the effect repertoire of **ABC** when the system is in state (1, 0, 0)

Calculating an effect repertoire:

Conditioning on the mechanism ABC

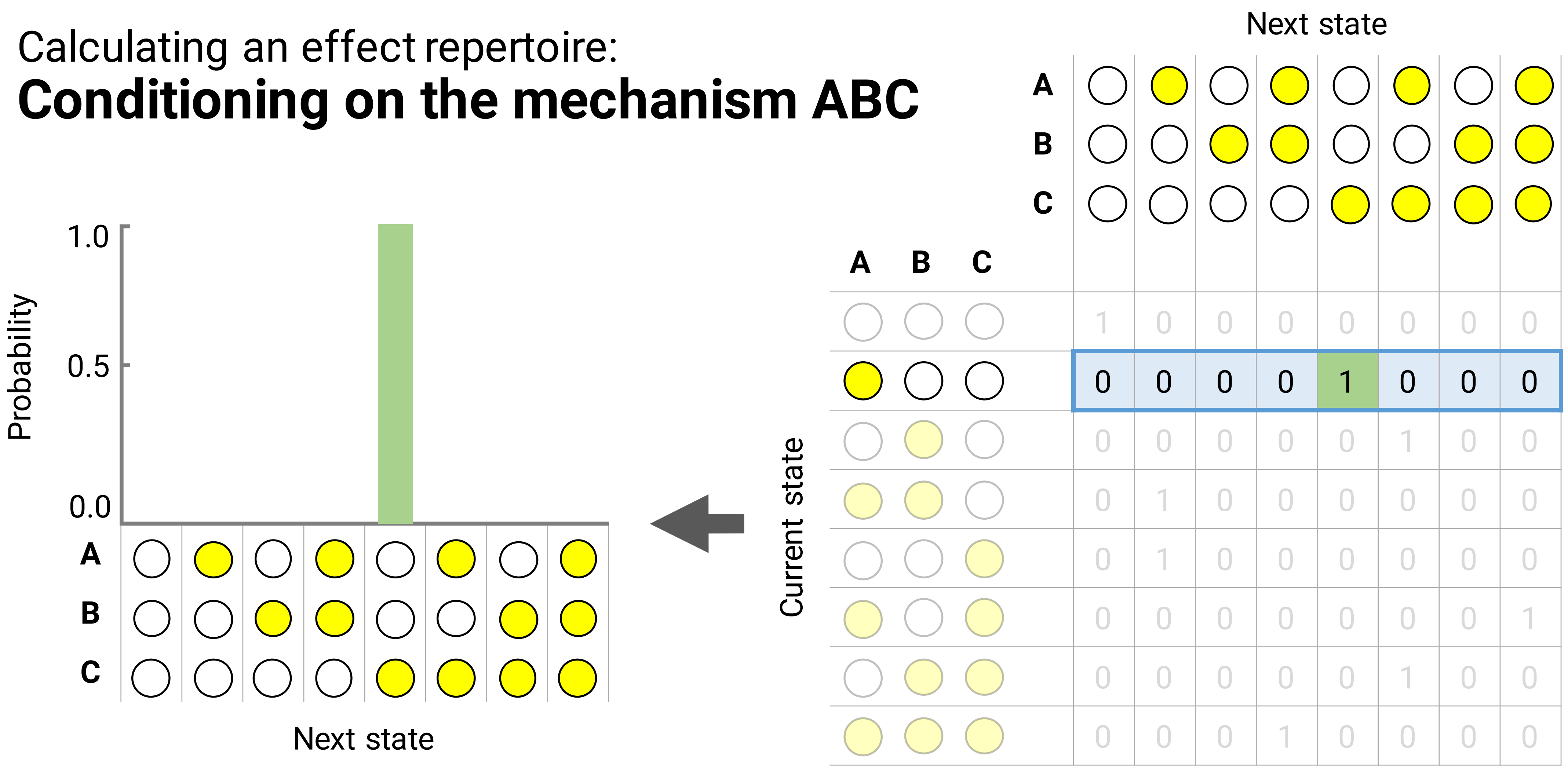

This is the effect repertoire of **ABC** when the system is in state (1, 0, 0)

Calculating an effect repertoire:
Purviews

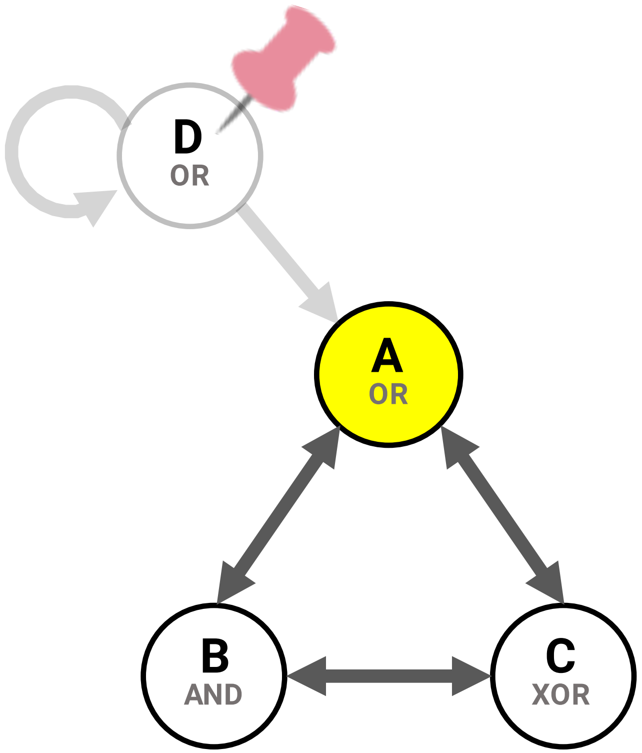

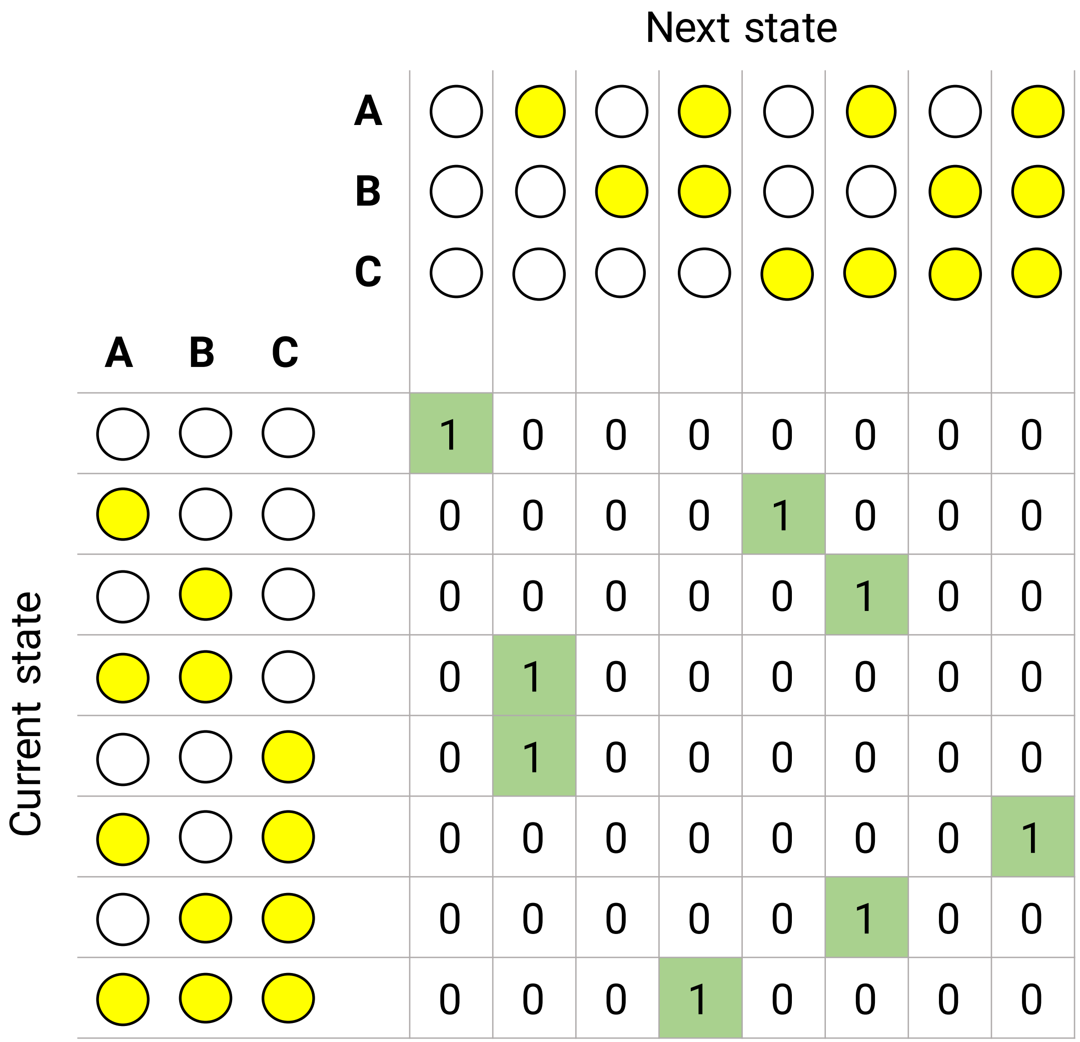

But in general, we can determine how knowing the current state constrains the next state of a *subset* of elements, rather than that of the whole system

Calculating an effect repertoire:

Purviews

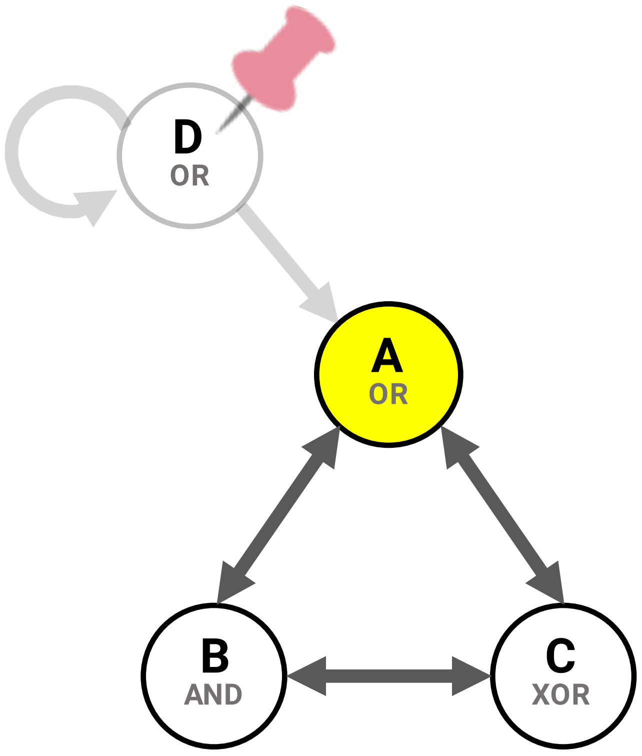

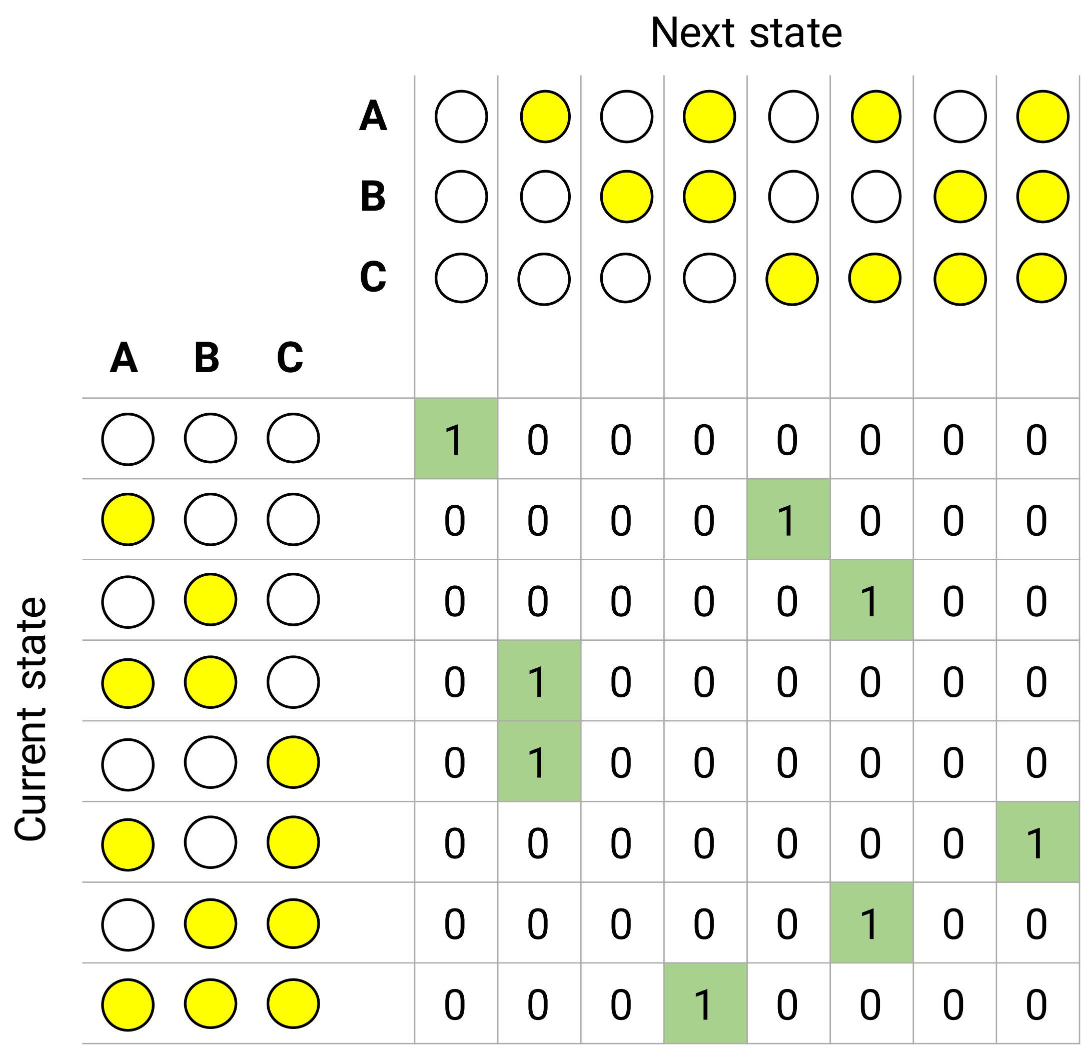

The subset of elements whose next state we're interested in is called the **purview**

Calculating an effect repertoire:

ABC over purview BC

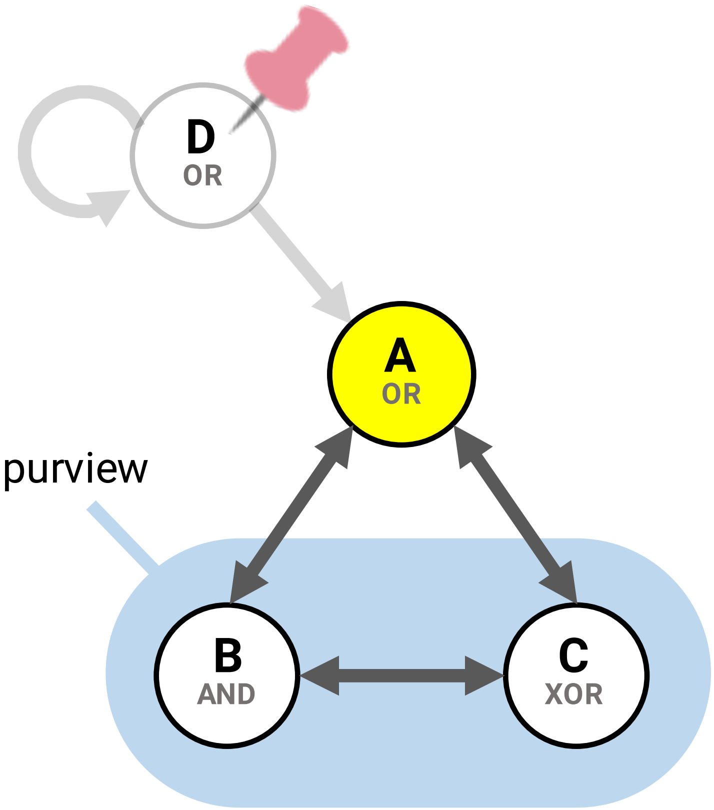

Current state A	Current state B	Current state C	Next state							
		A	○	●	○	●	○	●	○	●
		B	○	○	●	●	○	○	●	●
		C	○	○	○	○	●	●	●	●
○	○	○	1	0	0	0	0	0	0	0
●	○	○	0	0	0	0	1	0	0	0
○	●	○	0	0	0	0	0	1	0	0
●	●	○	0	1	0	0	0	0	0	0
○	○	●	0	1	0	0	0	0	0	0
●	○	●	0	0	0	0	0	0	0	1
○	●	●	0	0	0	0	0	1	0	0
●	●	●	0	0	0	1	0	0	0	0

For example, let's calculate how knowing the current state of **ABC** constrains the next state of the purview **BC**

Calculating an effect repertoire:
ABC over purview BC

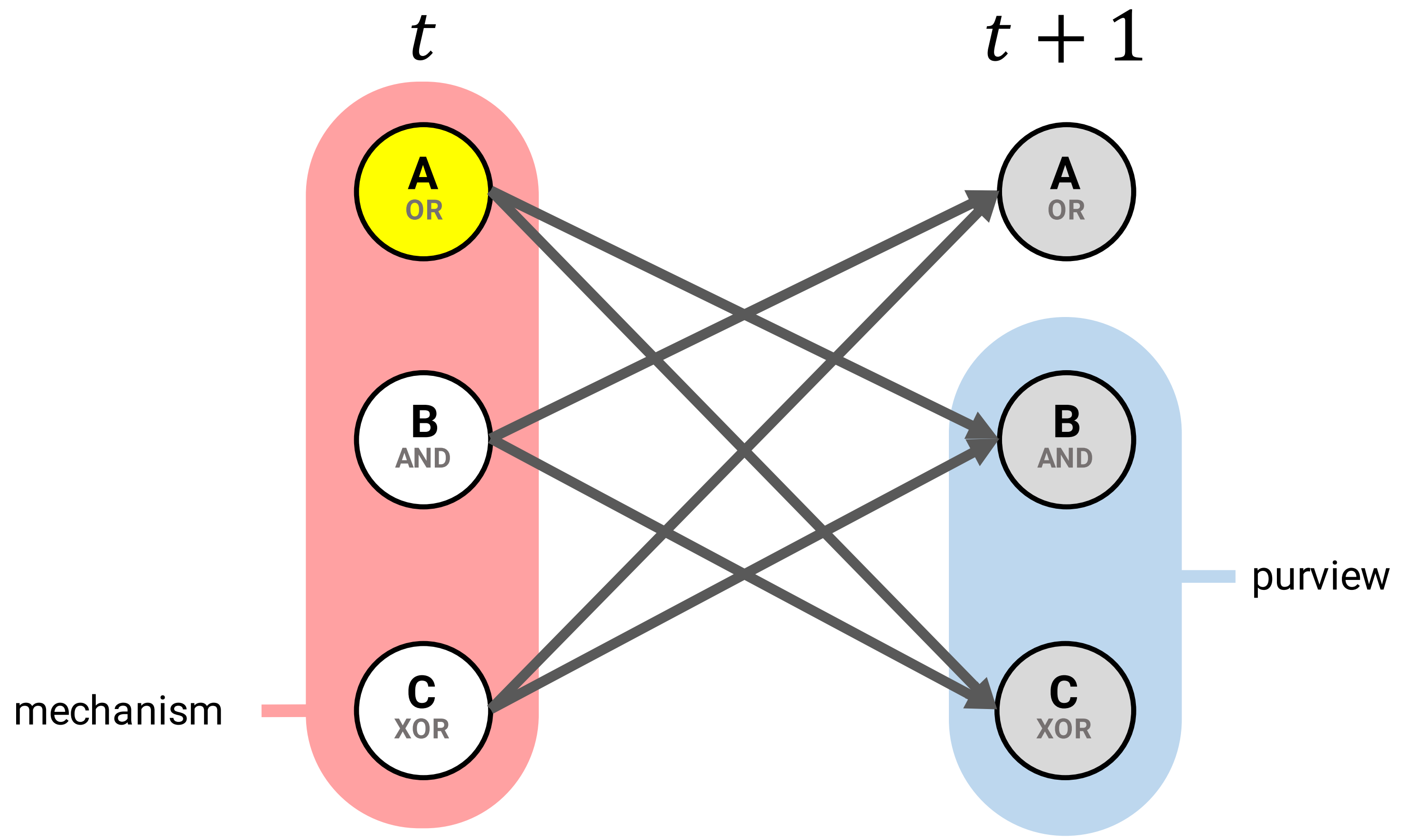

Let's unfold the graph in time between the current and next timestep

Calculating an effect repertoire:

ABC over purview BC

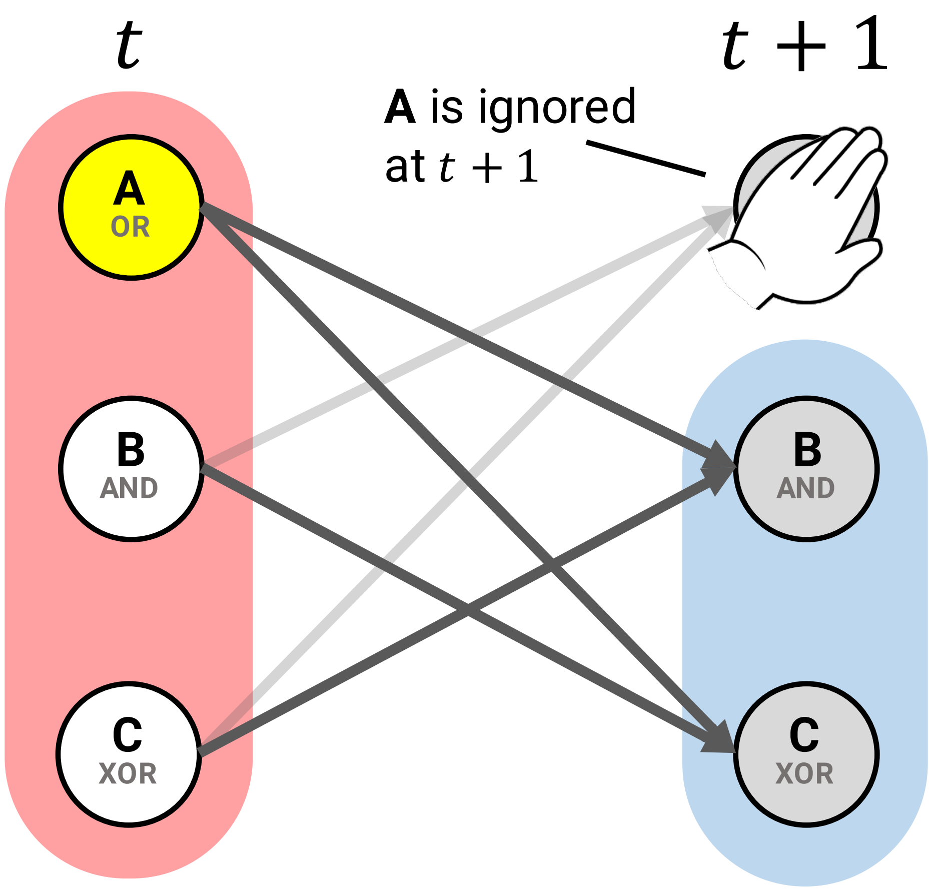

			Next state A	○	●	○	●	○	●	○	●
			**Next state B**	○	○	●	●	○	○	●	●
			**Next state C**	○	○	○	○	●	●	●	●
**Current state A**	**B**	**C**									
○	○	○		1	0	0	0	0	0	0	0
●	○	○		0	0	0	0	1	0	0	0
○	●	○		0	0	0	0	0	1	0	0
●	●	○		0	1	0	0	0	0	0	0
○	○	●		0	1	0	0	0	0	0	0
●	○	●		0	0	0	0	0	0	0	1
○	●	●		0	0	0	0	0	1	0	0
●	●	●		0	0	0	1	0	0	0	0

Since we're only interested in the next state of the purview **BC**, we want to **ignore** the next state of **A**

Calculating an effect repertoire:

ABC over purview BC

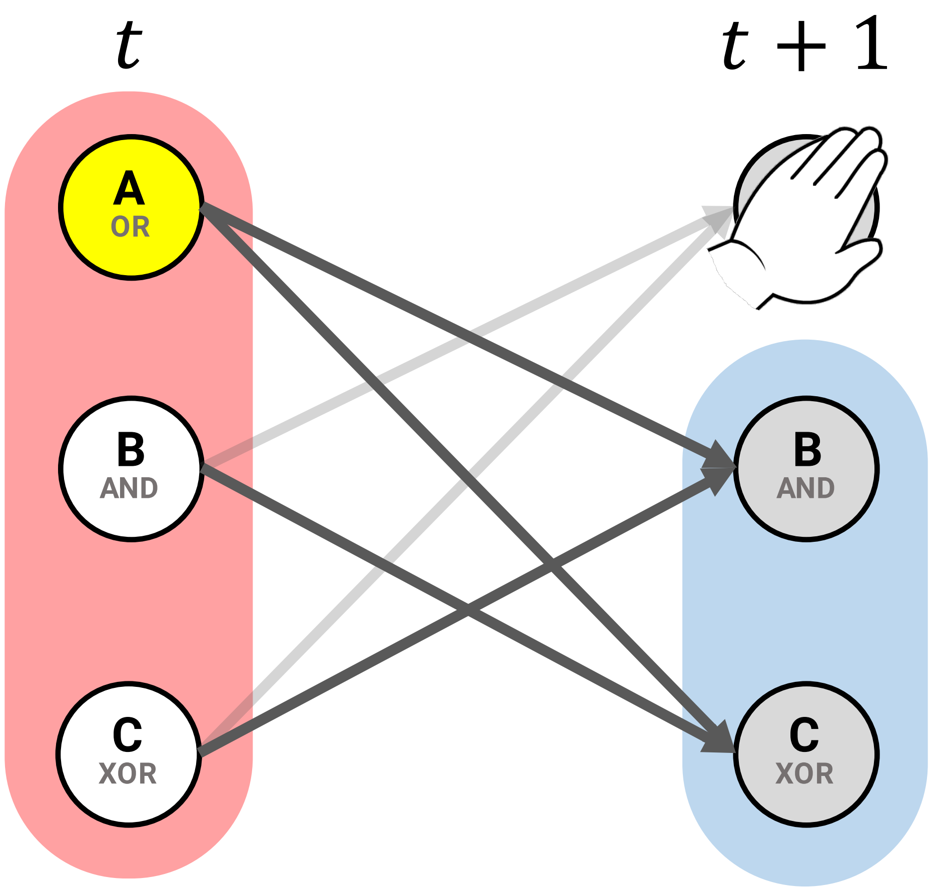

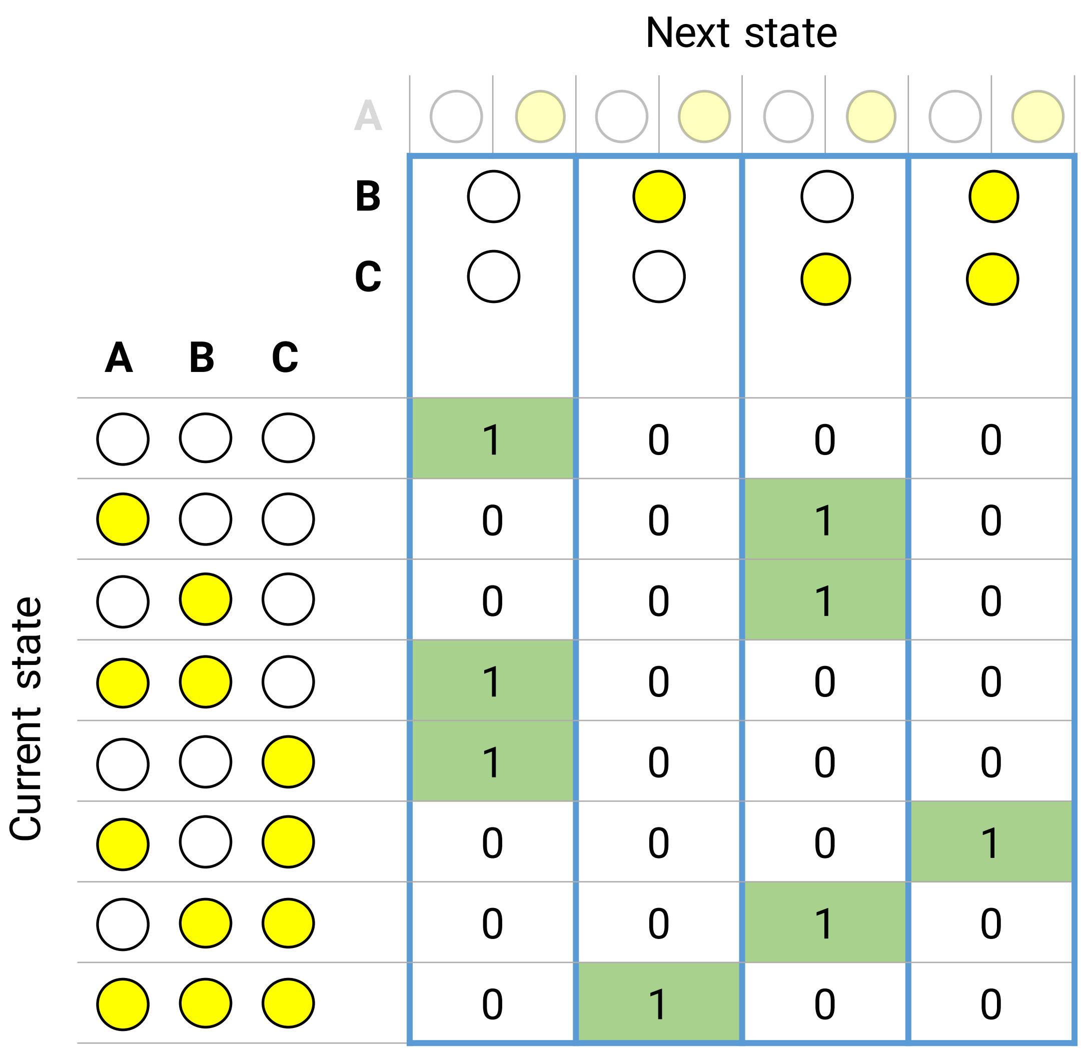

So, we marginalize the next state of **A** out of the TPM

Calculating an effect repertoire:
ABC over purview BC

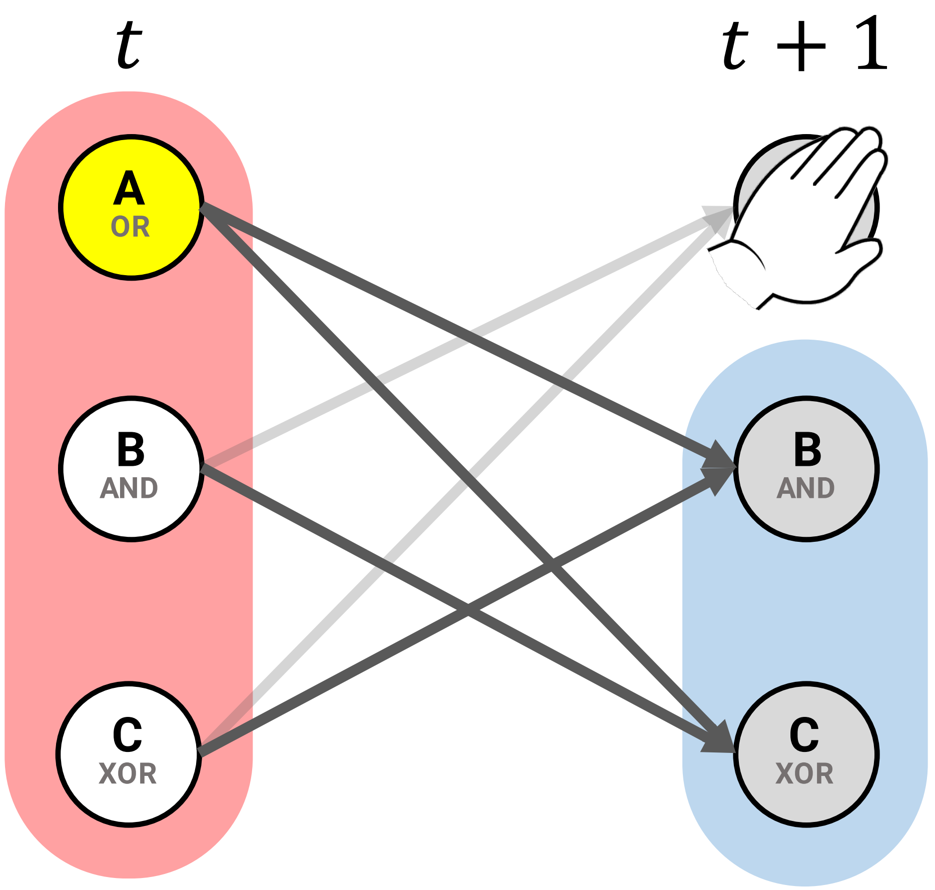

Next state

Current state: A	B	C	B ○ C ○	B ● C ○	B ○ C ●	B ● C ●
○	○	○	1	0	0	0
●	○	○	0	0	1	0
○	●	○	0	0	1	0
●	●	○	1	0	0	0
○	○	●	1	0	0	0
●	○	●	0	0	0	1
○	●	●	0	0	1	0
●	●	●	0	1	0	0

So, we marginalize the next state of **A** out of the TPM

Calculating an effect repertoire:

ABC over purview BC

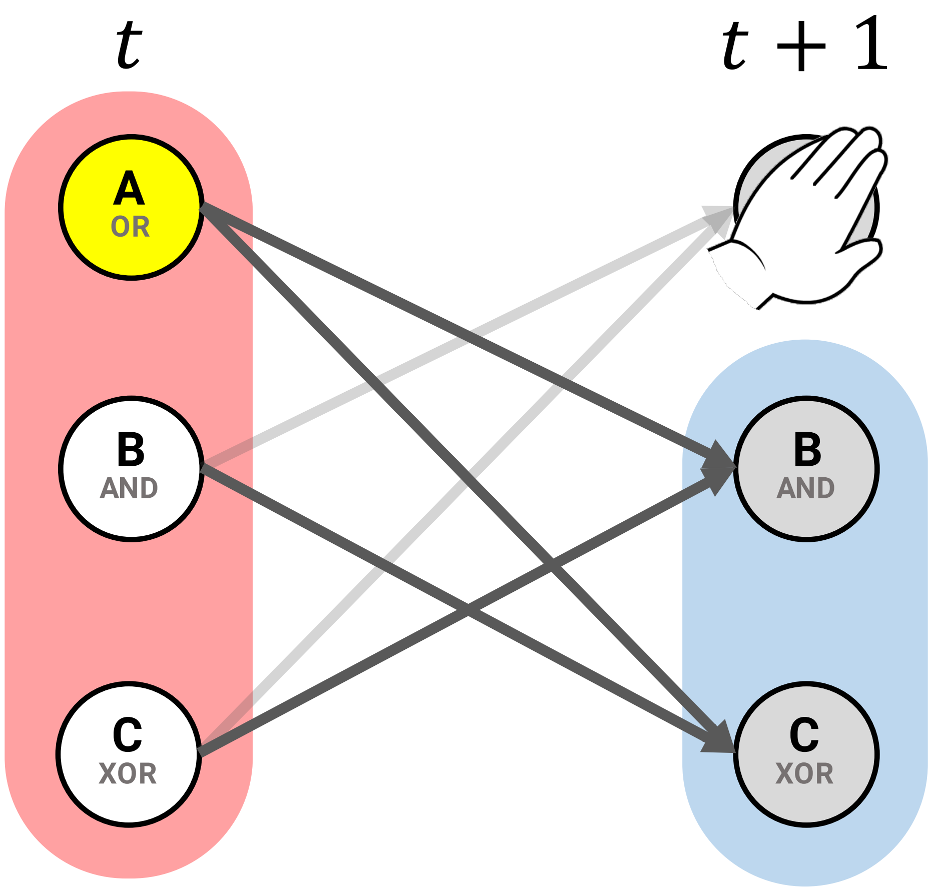

Current state (A)	Current state (B)	Current state (C)	Next state B: ○ C: ○	Next state B: ● C: ○	Next state B: ○ C: ●	Next state B: ● C: ●
○	○	○	1	0	0	0
●	○	○	0	0	1	0
○	●	○	0	0	1	0
●	●	○	1	0	0	0
○	○	●	1	0	0	0
●	○	●	0	0	0	1
○	●	●	0	0	1	0
●	●	●	0	1	0	0

Now we have a TPM that just gives the probabilities of the next states of **B** and **C**

Calculating an effect repertoire:

ABC over purview BC

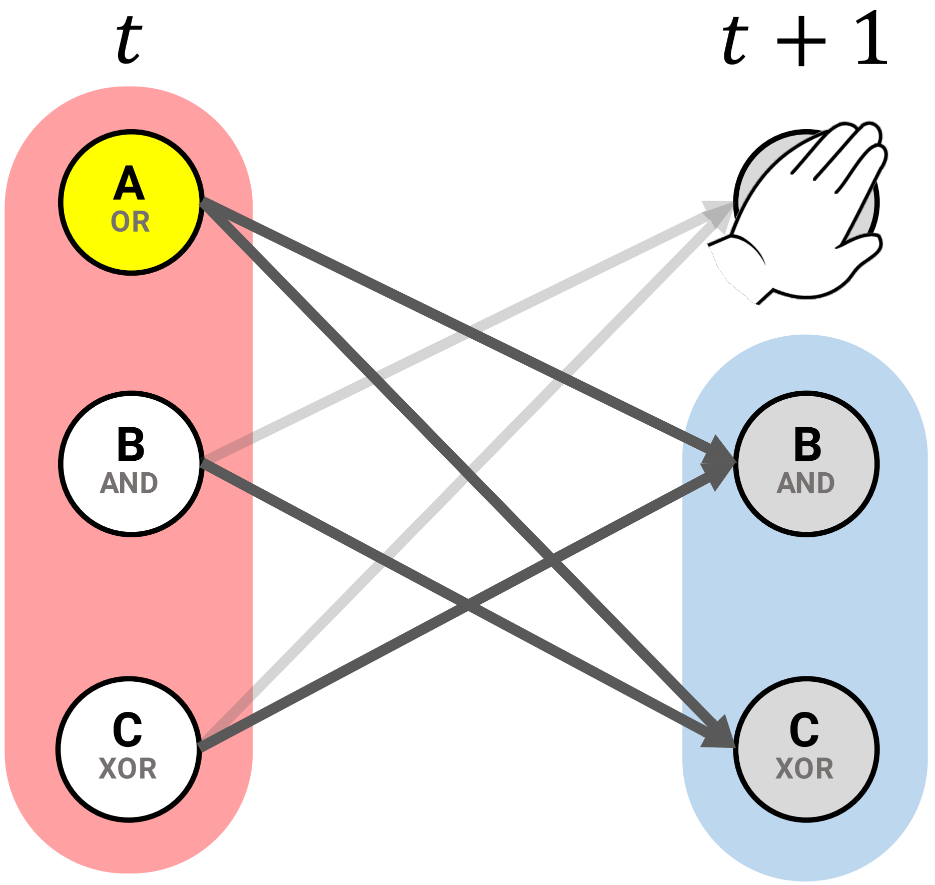

Next state

A	B	C	B: ○ C: ○	B: ● C: ○	B: ○ C: ●	B: ● C: ●
○	○	○	1	0	0	0
●	○	○	0	0	1	0
○	●	○	0	0	1	0
●	●	○	1	0	0	0
○	○	●	1	0	0	0
●	○	●	0	0	0	1
○	●	●	0	0	1	0
●	●	●	0	1	0	0

Current state

So we can condition on the current state of the mechanism to get the effect repertoire of mechanism **ABC** over purview **BC** when the system is in state (1, 0, 0)

Calculating an effect repertoire:

ABC over purview BC

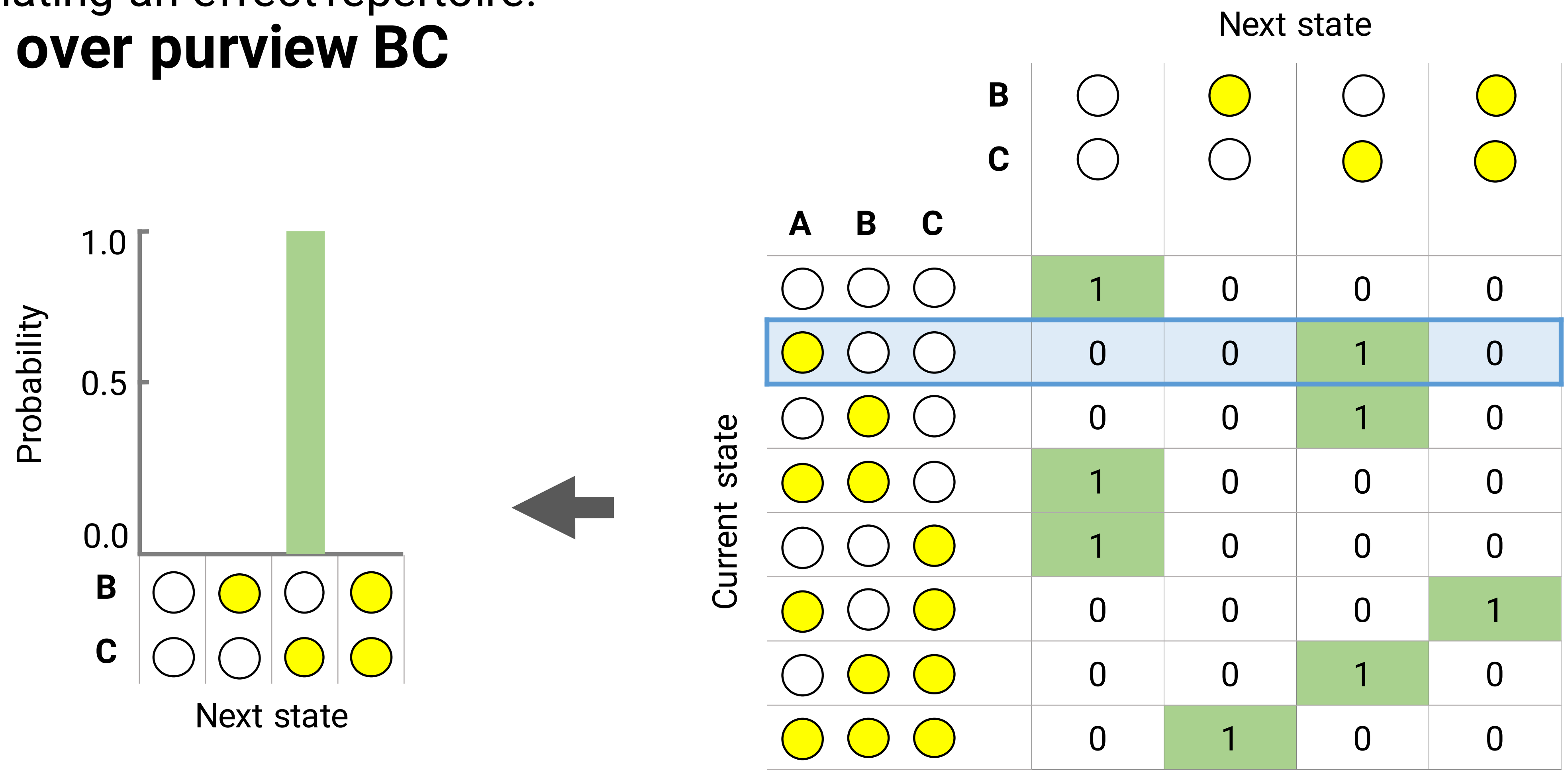

So we can condition on the current state of the mechanism to get the effect repertoire of mechanism **ABC** over purview **BC** when the system is in state (1, 0, 0)

Calculating an effect repertoire:
Mechanism C over purview BC

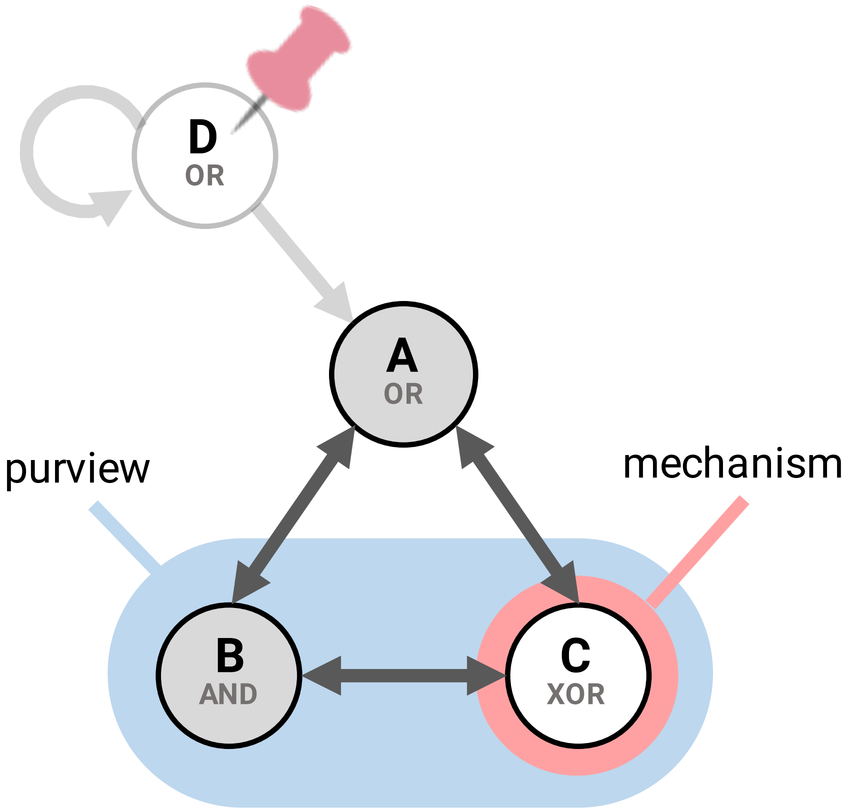

Current state			Next state							
		A	○	●	○	●	○	●	○	●
		B	○	○	●	●	○	○	●	●
		C	○	○	○	○	●	●	●	●
**A**	**B**	**C**								
○	○	○	1	0	0	0	0	0	0	0
●	○	○	0	0	0	0	1	0	0	0
○	●	○	0	0	0	0	0	1	0	0
●	●	○	0	1	0	0	0	0	0	0
○	○	●	0	1	0	0	0	0	0	0
●	○	●	0	0	0	0	0	0	0	1
○	●	●	0	0	0	0	0	1	0	0
●	●	●	0	0	0	1	0	0	0	0

Now let's consider an example of both a limited mechanism and a limited purview:
The effect repertoire of candidate mechanism **C** (red) over the purview **BC** (blue)

Calculating an effect repertoire:
Mechanism C over purview BC

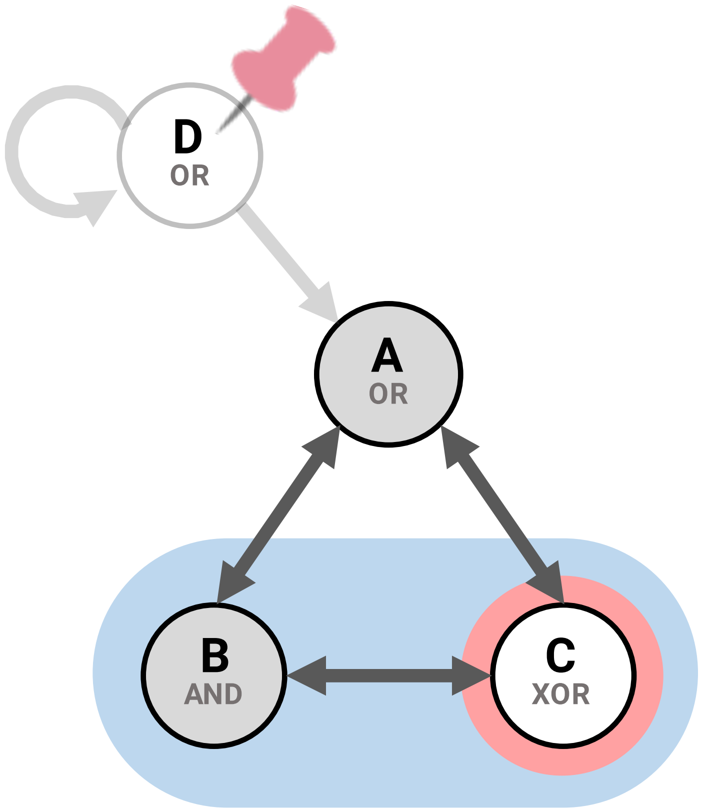

Current state			Next state							
		A	○	●	○	●	○	●	○	●
		B	○	○	●	●	○	○	●	●
		C	○	○	○	○	●	●	●	●
A	B	C								
○	○	○	1	0	0	0	0	0	0	0
●	○	○	0	0	0	0	1	0	0	0
○	●	○	0	0	0	0	0	1	0	0
●	●	○	0	1	0	0	0	0	0	0
○	○	●	0	1	0	0	0	0	0	0
●	○	●	0	0	0	0	0	0	0	1
○	●	●	0	0	0	0	0	1	0	0
●	●	●	0	0	0	1	0	0	0	0

The idea is to fix the current state of the mechanism **C** and perturb the other, unconstrained elements **A** and **B** into all their possible states (with equal likelihood) and observe the effects on the purview, **B** and **C**

Calculating an effect repertoire:

Mechanism C over purview BC

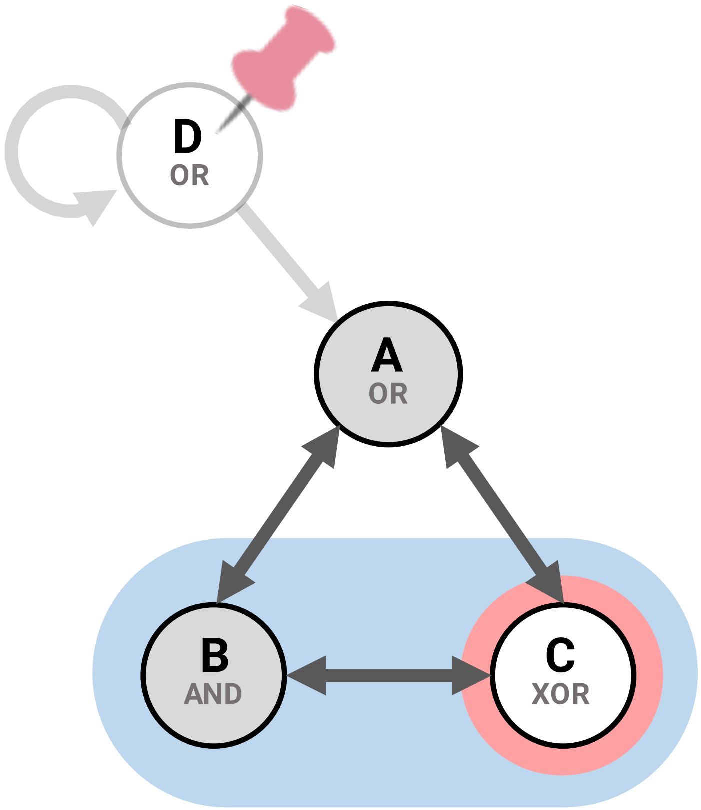

Next state

Current state

		**A**	○	●	○	●	○	●	○	●
		**B**	○	○	●	●	○	○	●	●
		**C**	○	○	○	○	●	●	●	●
**A**	**B**	**C**								
○	○	○	1	0	0	0	0	0	0	0
●	○	○	0	0	0	0	1	0	0	0
○	●	○	0	0	0	0	0	1	0	0
●	●	○	0	1	0	0	0	0	0	0
○	○	●	0	1	0	0	0	0	0	0
●	○	●	0	0	0	0	0	0	0	1
○	●	●	0	0	0	0	0	1	0	0
●	●	●	0	0	0	1	0	0	0	0

However, note that the two purview elements **B** and **C** share common input from **A**

Calculating an effect repertoire:

Mechanism C over purview BC

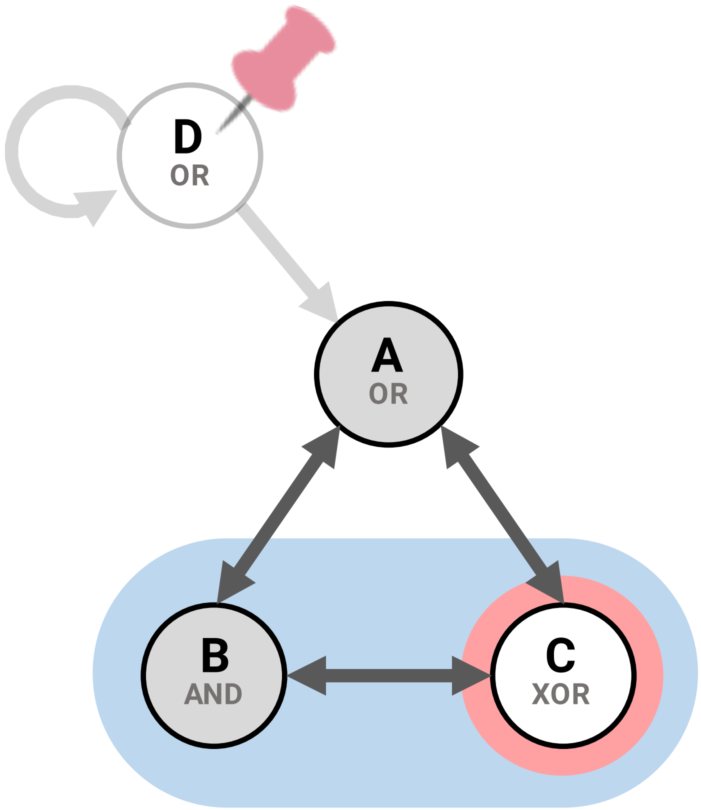

Next state

Current state A	B	C	A: ○ B: ○ C: ○	A: ● B: ○ C: ○	A: ○ B: ● C: ○	A: ● B: ● C: ○	A: ○ B: ○ C: ●	A: ● B: ○ C: ●	A: ○ B: ● C: ●	A: ● B: ● C: ●
○	○	○	1	0	0	0	0	0	0	0
●	○	○	0	0	0	0	1	0	0	0
○	●	○	0	0	0	0	0	1	0	0
●	●	○	0	1	0	0	0	0	0	0
○	○	●	0	1	0	0	0	0	0	0
●	○	●	0	0	0	0	0	0	0	1
○	●	●	0	0	0	0	0	1	0	0
●	●	●	0	0	0	1	0	0	0	0

This means that when we set **A**'s state during the perturbation, the observed effects on **B** and **C** might depend in part on correlations due to this common input, rather than depending only on the current state of the mechanism **C**

Calculating an effect repertoire:

Virtual elements

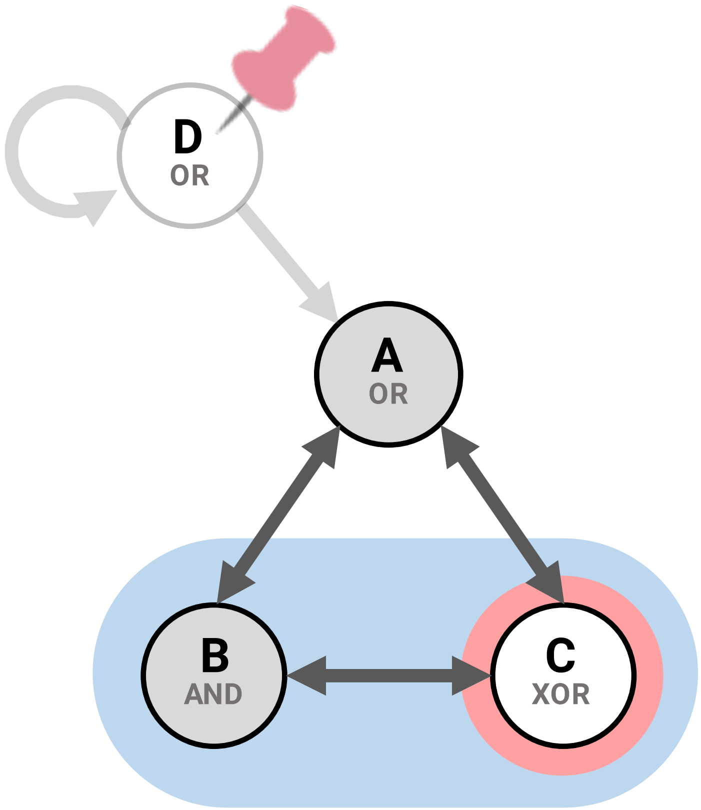

Next state

Current state

			A	○	●	○	●	○	●	○	●
			B	○	○	●	●	○	○	●	●
			C	○	○	○	○	●	●	●	●
A	B	C									
○	○	○		1	0	0	0	0	0	0	0
●	○	○		0	0	0	0	1	0	0	0
○	●	○		0	0	0	0	0	1	0	0
●	●	○		0	1	0	0	0	0	0	0
○	○	●		0	1	0	0	0	0	0	0
●	○	●		0	0	0	0	0	0	0	1
○	●	●		0	0	0	0	0	1	0	0
●	●	●		0	0	0	1	0	0	0	0

To remove the unwanted effects of correlations due to common input, we introduce **virtual elements** that we can perturb independently

Calculating an effect repertoire:
Virtual elements

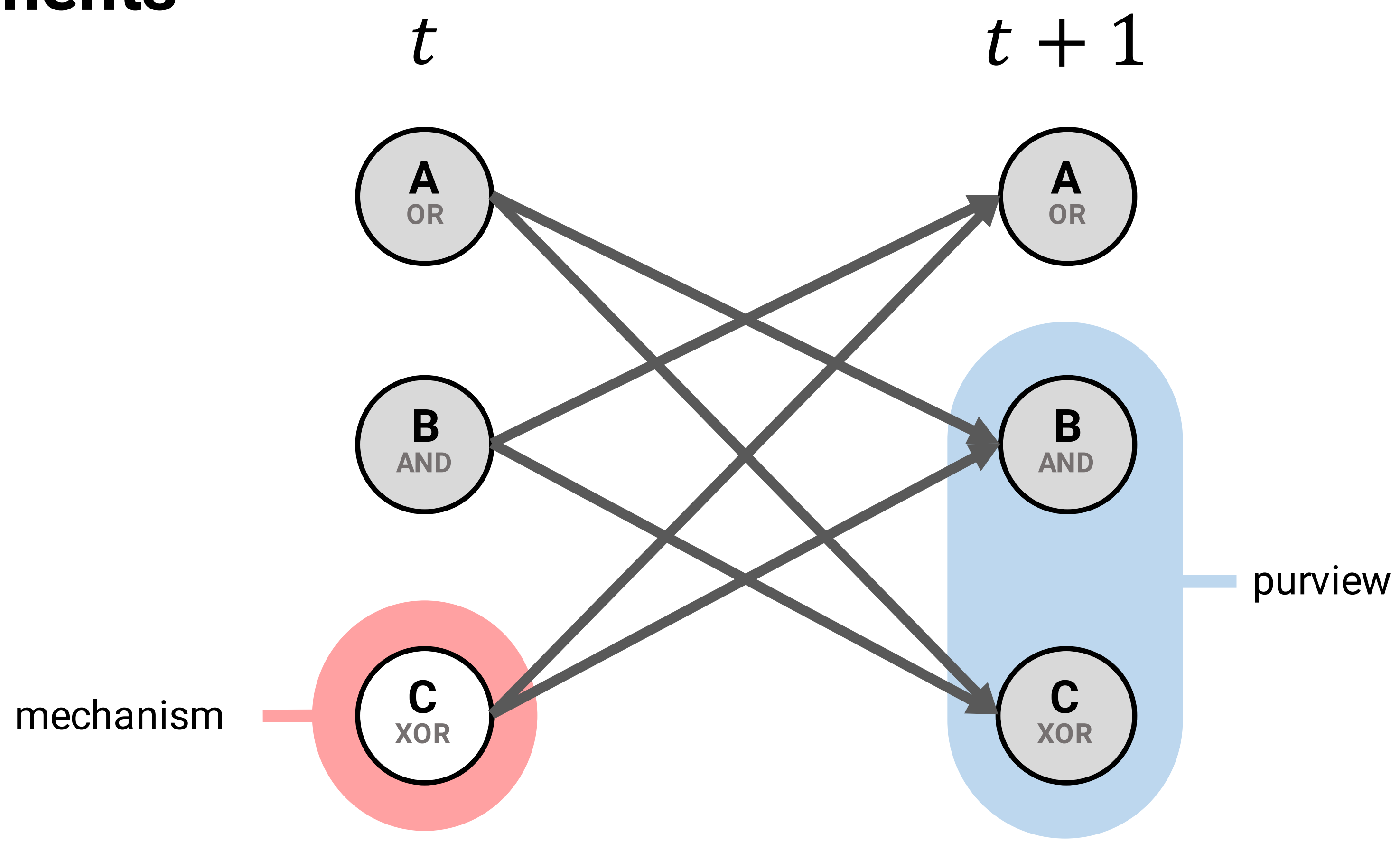

Let's unfold the graph in time between t and $t+1$ again

Calculating an effect repertoire:

Virtual elements

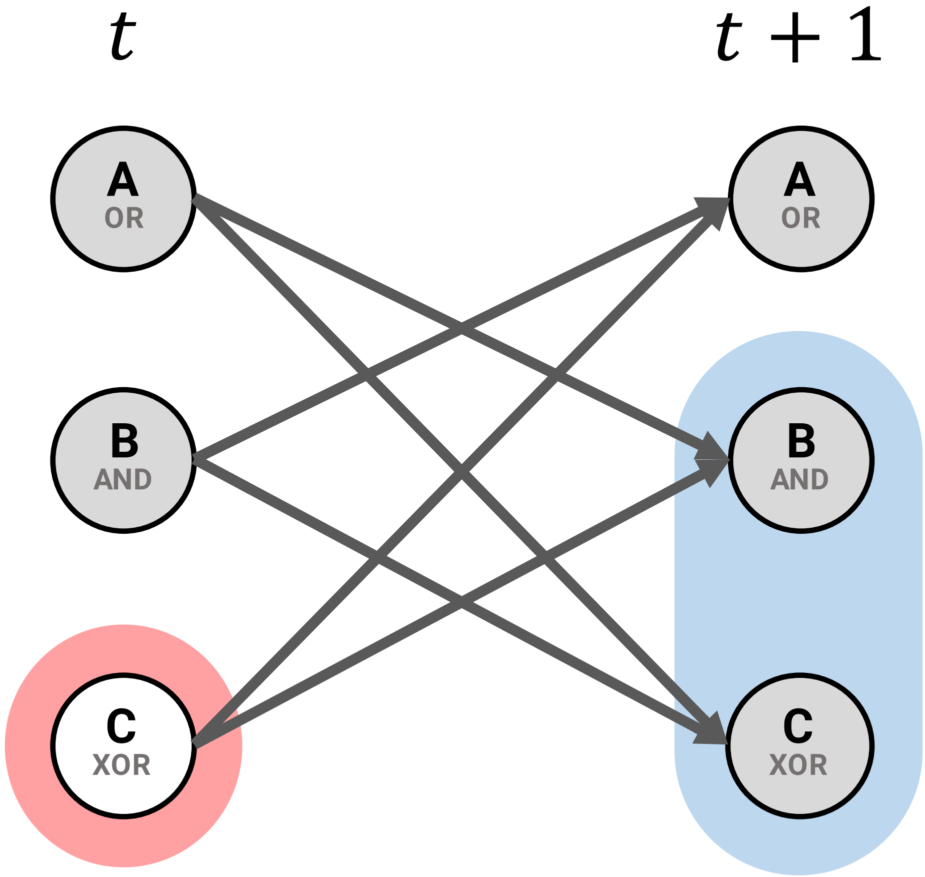

Since **A** is outside the mechanism (and thus will be perturbed) and it outputs to more than one purview element, we introduce virtual elements $\mathbf{A_B}$ and $\mathbf{A_C}$ at time t that independently provide input to **B** and **C**

Calculating an effect repertoire:
Virtual elements

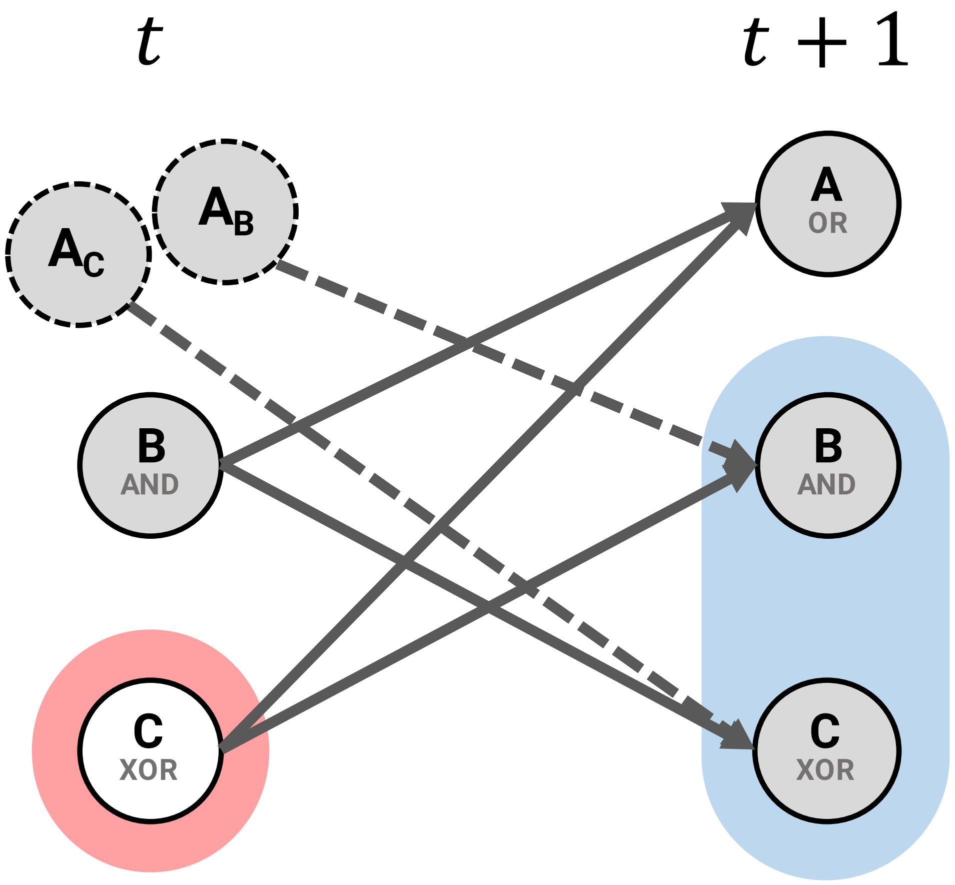

Since **A** is outside the mechanism (and thus will be perturbed) and it outputs to more than one purview element, we introduce virtual elements $\mathbf{A_B}$ and $\mathbf{A_C}$ at time t that independently provide input to **B** and **C**

Calculating an effect repertoire:

Virtual elements

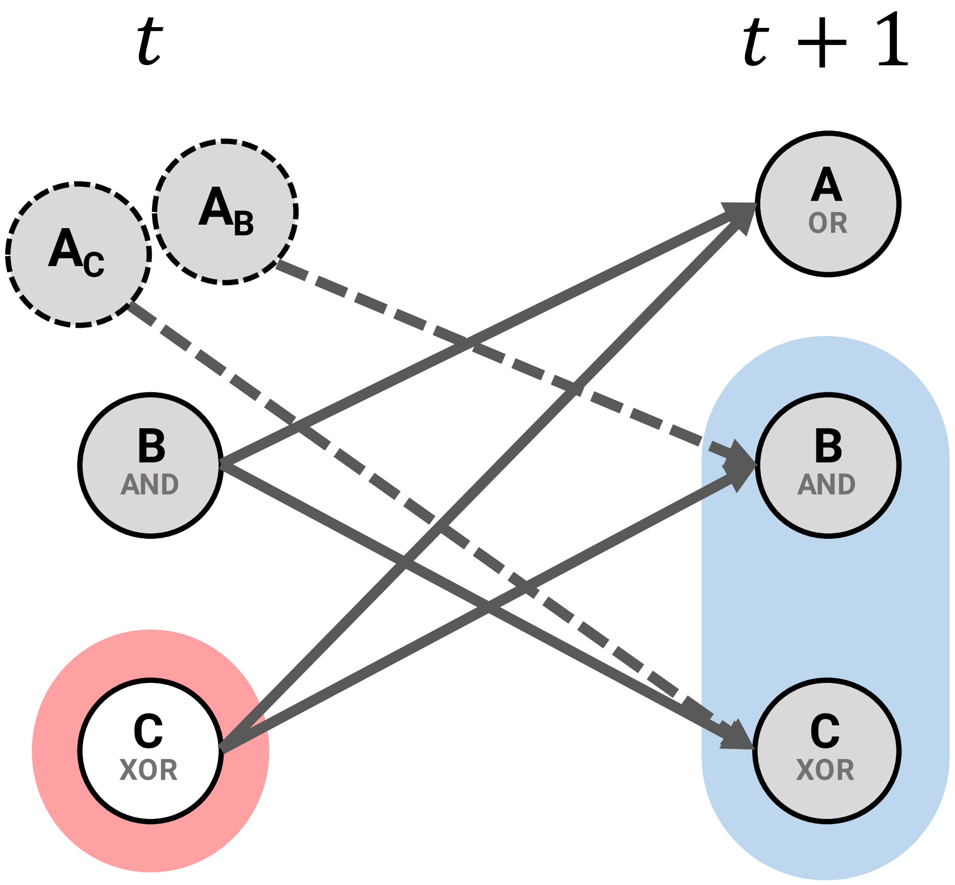

We can now perturb the non-mechanism elements into all their possible states at t to get a "virtual TPM" that doesn't contain correlations due to common input

Calculating an effect repertoire:
Virtual elements

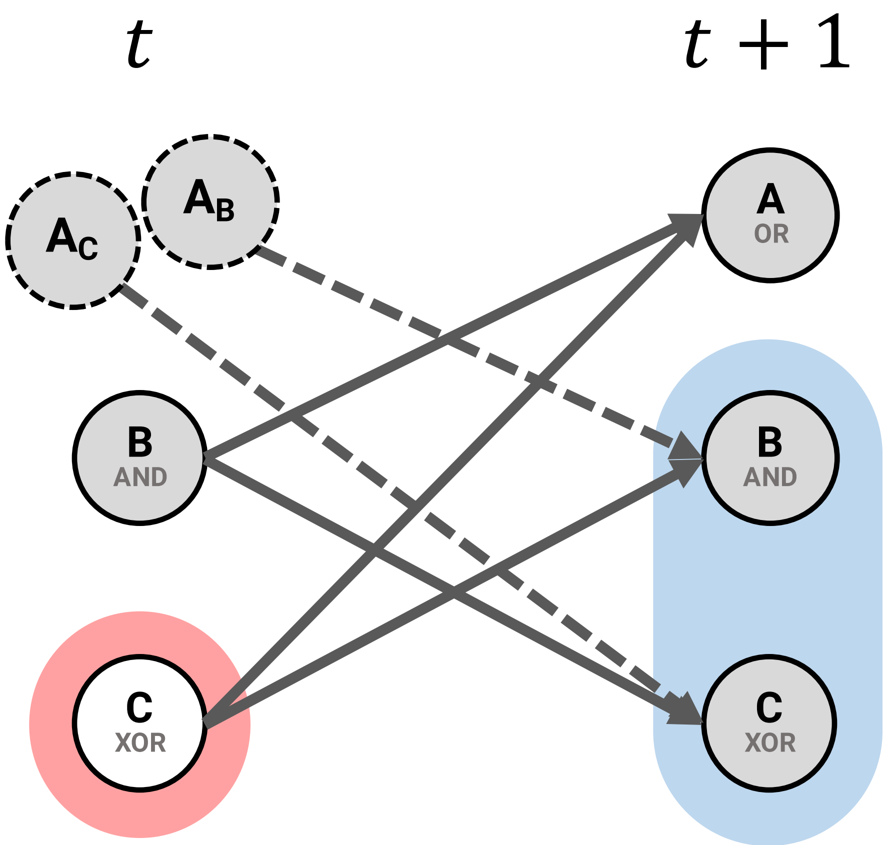

Next state

Current state

				A	○	●	○	●	○	●	○	●
				B	○	○	●	●	○	○	●	●
				C	○	○	○	○	●	●	●	●
A_B	A_C	B	C									
○	○	○	○		1	0	0	0	0	0	0	0
●	○	○	○		1	0	0	0	0	0	0	0
○	●	○	○		0	0	0	0	1	0	0	0
●	●	○	○		0	0	0	0	1	0	0	0
○	○	●	○		0	0	0	0	0	1	0	0
●	○	●	○		0	0	0	0	0	1	0	0
○	●	●	○		0	1	0	0	0	0	0	0
●	●	●	○		0	1	0	0	0	0	0	0
○	○	○	●		0	1	0	0	0	0	0	0
●	○	○	●		0	0	0	1	0	0	0	0
○	●	○	●		0	0	0	0	0	1	0	0
●	●	○	●		0	0	0	0	0	0	0	1
○	○	●	●		0	0	0	0	0	1	0	0
●	○	●	●		0	0	0	0	0	0	0	1
○	●	●	●		0	1	0	0	0	0	0	0
●	●	●	●		0	0	0	1	0	0	0	0

We can now perturb the non-mechanism elements into all their possible states at t to get a “virtual TPM” that doesn’t contain correlations due to common input

Calculating an effect repertoire:
Virtual elements

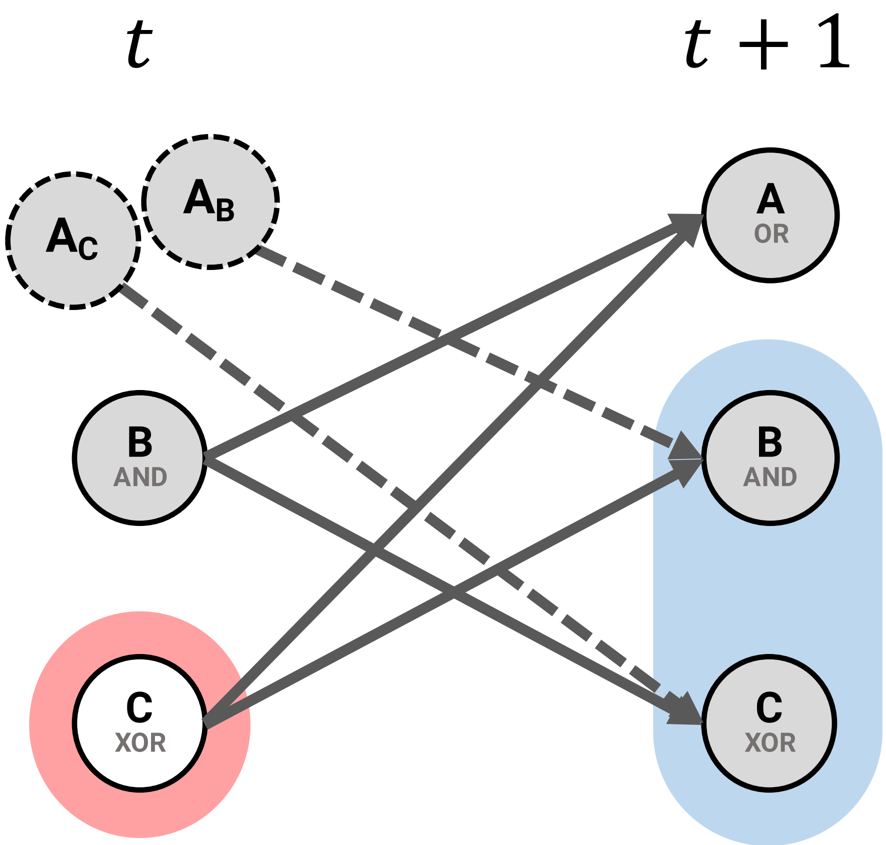

Next state

				A	○	●	○	●	○	●	○	●
				B	○	○	●	●	○	○	●	●
				C	○	○	○	○	●	●	●	●
A_B	A_C	B	C									
○	○	○	○		1	0	0	0	0	0	0	0
●	○	○	○		1	0	0	0	0	0	0	0
○	●	○	○		0	0	0	0	1	0	0	0
●	●	○	○		0	0	0	0	1	0	0	0
○	○	●	○		0	0	0	0	0	1	0	0
●	○	●	○		0	0	0	0	0	1	0	0
○	●	●	○		0	1	0	0	0	0	0	0
●	●	●	○		0	1	0	0	0	0	0	0
○	○	○	●		0	1	0	0	0	0	0	0
●	○	○	●		0	0	0	1	0	0	0	0
○	●	○	●		0	0	0	0	0	1	0	0
●	●	○	●		0	0	0	0	0	0	0	1
○	○	●	●		0	0	0	0	0	1	0	0
●	○	●	●		0	0	0	0	0	0	0	1
○	●	●	●		0	1	0	0	0	0	0	0
●	●	●	●		0	0	0	1	0	0	0	0

Current state

Now, since we’re only interested in how the current state of **C** constrains the next state of the purview **BC**, rather than the whole system, we want to **ignore** the next state of **A**

Calculating an effect repertoire:
Virtual elements

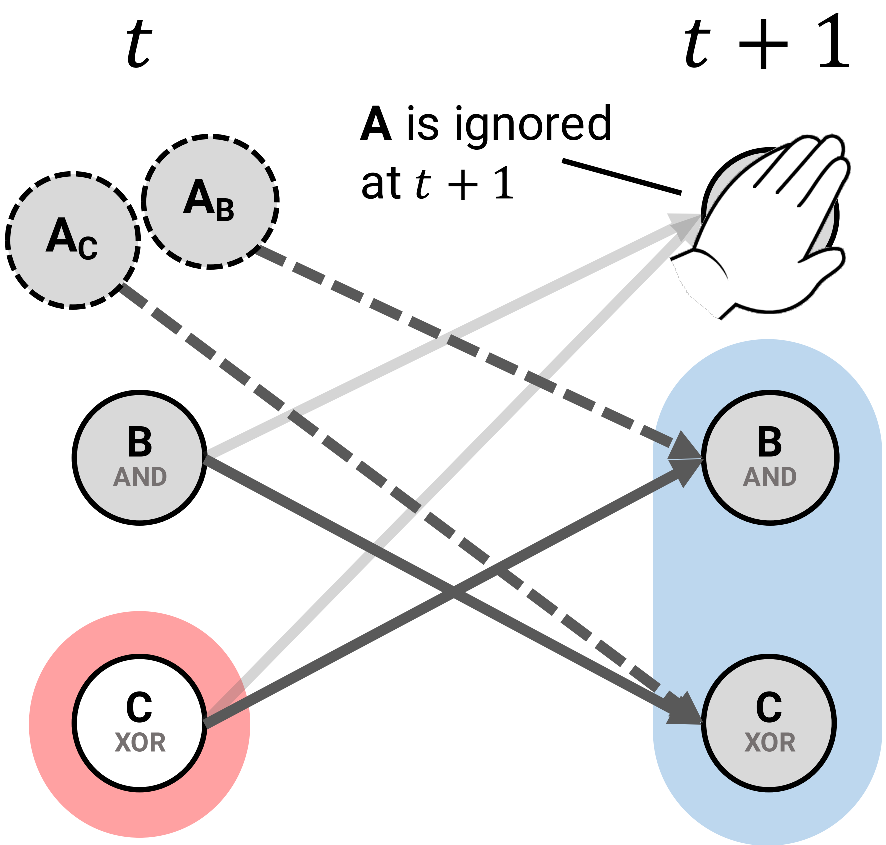

Next state

				A	○	●	○	●	○	●	○	●
				**B**	○	○	●	●	○	○	●	●
				**C**	○	○	○	○	●	●	●	●
**A_B**	**A_C**	**B**	**C**									
○	○	○	○		1	0	0	0	0	0	0	0
●	○	○	○		1	0	0	0	0	0	0	0
○	●	○	○		0	0	0	0	1	0	0	0
●	●	○	○		0	0	0	0	1	0	0	0
○	○	●	○		0	0	0	0	0	1	0	0
●	○	●	○		0	0	0	0	0	1	0	0
○	●	●	○		0	1	0	0	0	0	0	0
●	●	●	○		0	1	0	0	0	0	0	0
○	○	○	●		0	1	0	0	0	0	0	0
●	○	○	●		0	0	0	1	0	0	0	0
○	●	○	●		0	0	0	0	0	1	0	0
●	●	○	●		0	0	0	0	0	0	0	1
○	○	●	●		0	0	0	0	0	1	0	0
●	○	●	●		0	0	0	0	0	0	0	1
○	●	●	●		0	1	0	0	0	0	0	0
●	●	●	●		0	0	0	1	0	0	0	0

Current state

Now, since we're only interested in how the current state of **C** constrains the next state of the purview **BC**, rather than the whole system, we want to **ignore** the next state of **A**

Calculating an effect repertoire:

Marginalizing-out non-purview elements

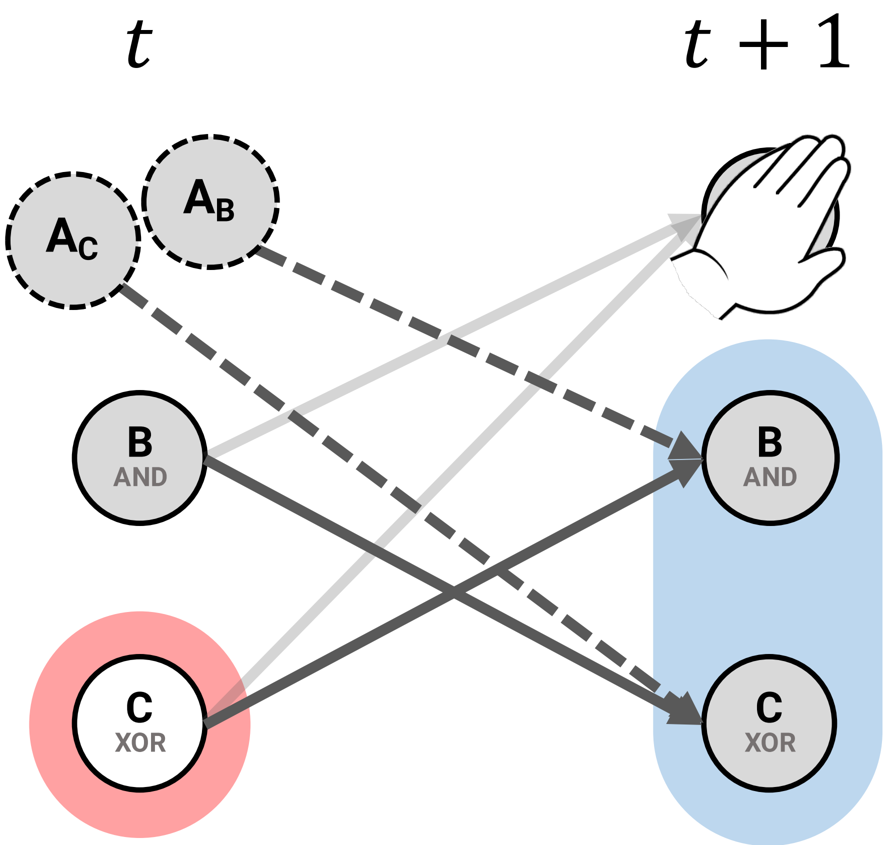

Next state / Current state

A_B	A_C	B	C								
				A: ○	A: ●	A: ○	A: ●	A: ○	A: ●	A: ○	A: ●
				B: ○	B: ○	B: ●	B: ●	B: ○	B: ○	B: ●	B: ●
				C: ○	C: ○	C: ○	C: ○	C: ●	C: ●	C: ●	C: ●
○	○	○	○	1	0	0	0	0	0	0	0
●	○	○	○	1	0	0	0	0	0	0	0
○	●	○	○	0	0	0	0	1	0	0	0
●	●	○	○	0	0	0	0	1	0	0	0
○	○	●	○	0	0	0	0	0	1	0	0
●	○	●	○	0	0	0	0	0	1	0	0
○	●	●	○	0	1	0	0	0	0	0	0
●	●	●	○	0	1	0	0	0	0	0	0
○	○	○	●	0	1	0	0	0	0	0	0
●	○	○	●	0	0	0	1	0	0	0	0
○	●	○	●	0	0	0	0	0	1	0	0
●	●	○	●	0	0	0	0	0	0	0	1
○	○	●	●	0	0	0	0	0	1	0	0
●	○	●	●	0	0	0	0	0	0	0	1
○	●	●	●	0	1	0	0	0	0	0	0
●	●	●	●	0	0	0	1	0	0	0	0

As usual, ignoring the next state of **A** corresponds to **marginalizing it out** of the TPM

Calculating an effect repertoire:

Marginalizing-out non-purview elements

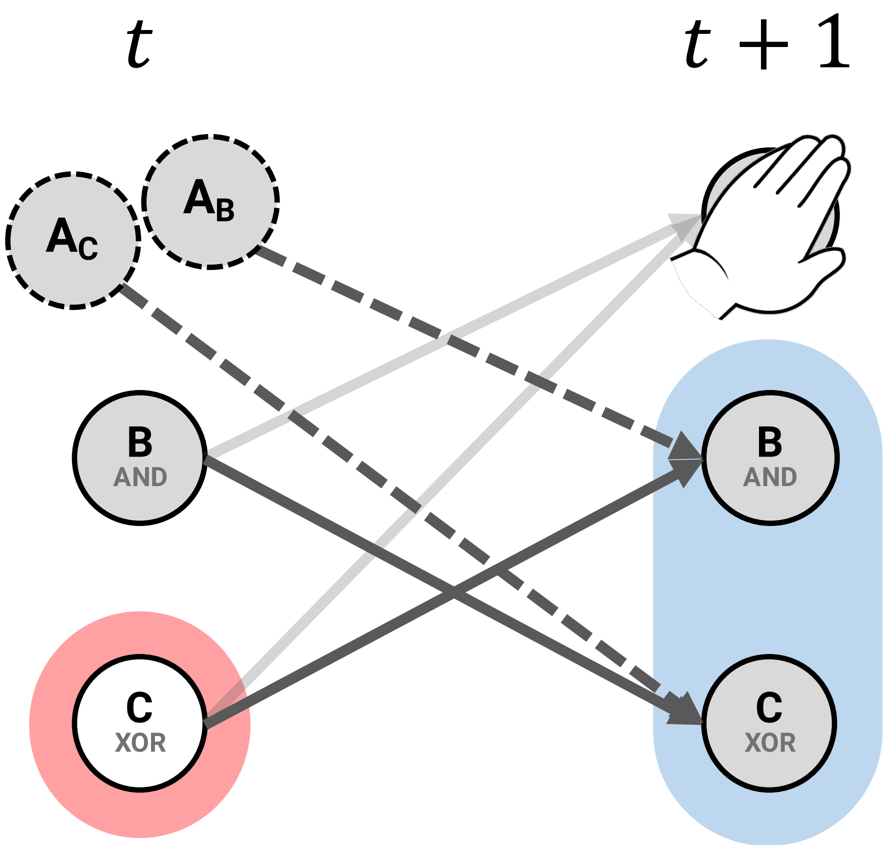

Next state

Current state

A_B	A_C	B	C								
			A	○	●	○	●	○	●	○	●
			B	○	○	●	●	○	○	●	●
			C	○	○	○	○	●	●	●	●
○	○	○	○	1	0	0	0	0	0	0	0
●	○	○	○	1	0	0	0	0	0	0	0
○	●	○	○	0	0	0	0	1	0	0	0
●	●	○	○	0	0	0	0	1	0	0	0
○	○	●	○	0	0	0	0	0	1	0	0
●	○	●	○	0	0	0	0	0	1	0	0
○	●	●	○	0	1	0	0	0	0	0	0
●	●	●	○	0	1	0	0	0	0	0	0
○	○	○	●	0	1	0	0	0	0	0	0
●	○	○	●	0	0	0	1	0	0	0	0
○	●	○	●	0	0	0	0	0	1	0	0
●	●	○	●	0	0	0	0	0	0	0	1
○	○	●	●	0	0	0	0	0	1	0	0
●	○	●	●	0	0	0	0	0	0	0	1
○	●	●	●	0	1	0	0	0	0	0	0
●	●	●	●	0	0	0	1	0	0	0	0

The process is the same as when we marginalized-out **D** as a background condition:
We sum pairs of columns whose corresponding states differ only by **A**'s state

Calculating an effect repertoire:

Marginalizing-out non-purview elements

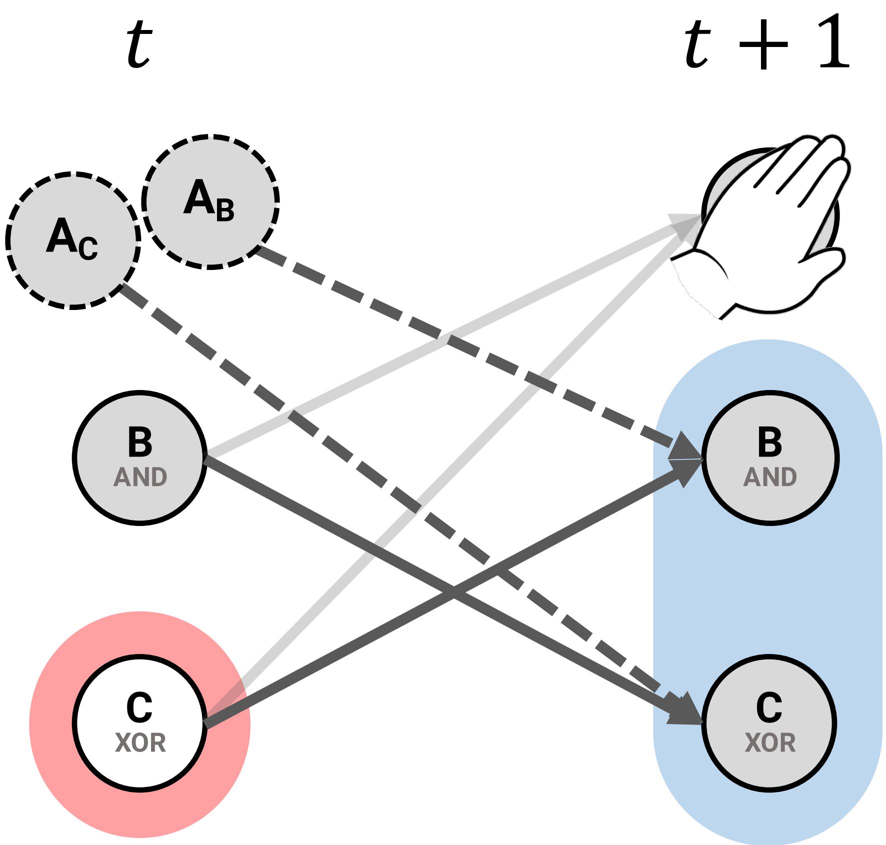

Next state

Current state: A_B	A_C	B	C	A: ○ B: ○ C: ○	A: ● B: ○ C: ○	A: ○ B: ● C: ○	A: ● B: ● C: ○	A: ○ B: ○ C: ●	A: ● B: ○ C: ●	A: ○ B: ● C: ●	A: ● B: ● C: ●
○	○	○	○	1	0	0	0	0	0	0	0
●	○	○	○	1	0	0	0	0	0	0	0
○	●	○	○	0	0	0	0	1	0	0	0
●	●	○	○	0	0	0	0	1	0	0	0
○	○	●	○	0	0	0	0	0	1	0	0
●	○	●	○	0	0	0	0	0	1	0	0
○	●	●	○	0	1	0	0	0	0	0	0
●	●	●	○	0	1	0	0	0	0	0	0
○	○	○	●	0	1	0	0	0	0	0	0
●	○	○	●	0	0	0	1	0	0	0	0
○	●	○	●	0	0	0	0	0	1	0	0
●	●	○	●	0	0	0	0	0	0	0	1
○	○	●	●	0	0	0	0	0	1	0	0
●	○	●	●	0	0	0	0	0	0	0	1
○	●	●	●	0	1	0	0	0	0	0	0
●	●	●	●	0	0	0	1	0	0	0	0

The process is the same as when we marginalized-out **D** as a background condition:
We sum pairs of columns whose corresponding states differ only by **A**'s state

Calculating an effect repertoire:
Marginalizing-out non-purview elements

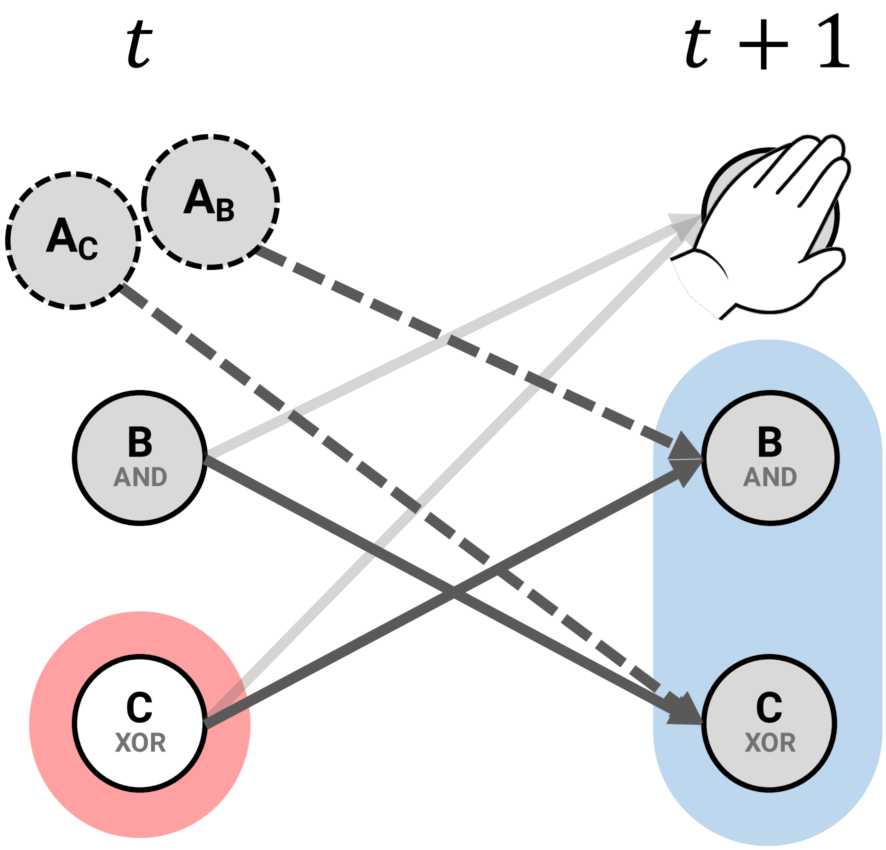

Next state

Current state: A_B	A_C	B	C	B ○ C ○	B ● C ○	B ○ C ●	B ● C ●
○	○	○	○	1	0	0	0
●	○	○	○	1	0	0	0
○	●	○	○	0	0	1	0
●	●	○	○	0	0	1	0
○	○	●	○	0	0	1	0
●	○	●	○	0	0	1	0
○	●	●	○	1	0	0	0
●	●	●	○	1	0	0	0
○	○	○	●	1	0	0	0
●	○	○	●	0	1	0	0
○	●	○	●	0	0	1	0
●	●	○	●	0	0	0	1
○	○	●	●	0	0	1	0
●	○	●	●	0	0	0	1
○	●	●	●	1	0	0	0
●	●	●	●	0	1	0	0

The process is the same as when we marginalized-out **D** as a background condition:
We sum pairs of columns whose corresponding states differ only by **A**'s state

Calculating an effect repertoire:
Marginalizing-out non-purview elements

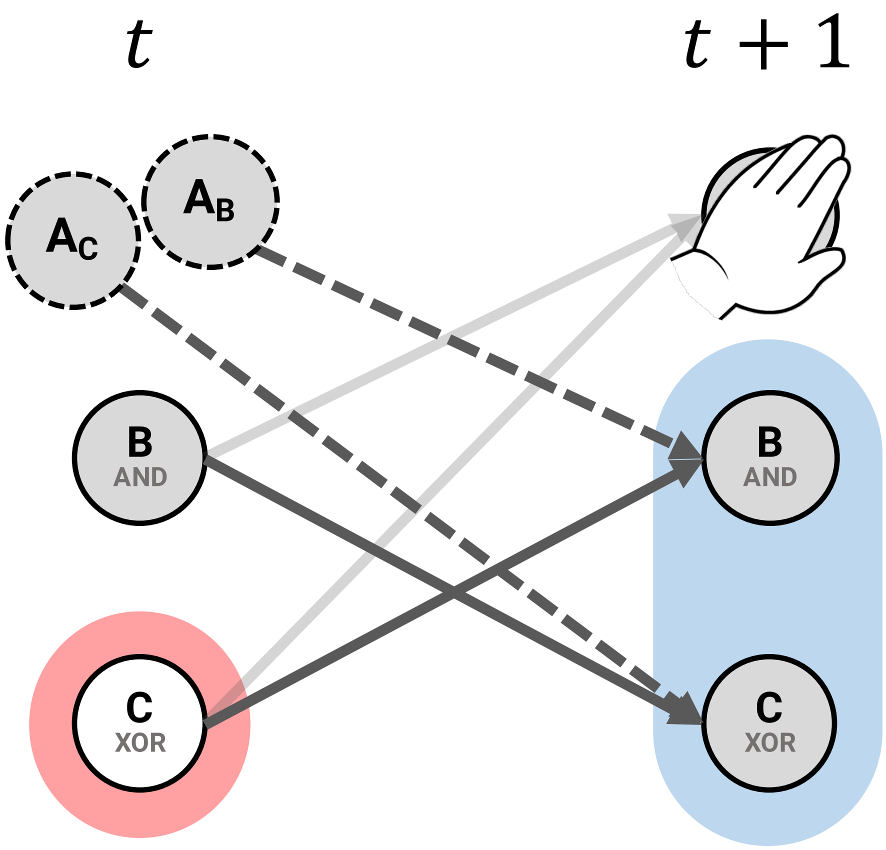

Next state

				B	○	●	○	●
				C	○	○	●	●
A_B	A_C	B	C					
○	○	○	○		1	0	0	0
●	○	○	○		1	0	0	0
○	●	○	○		0	0	1	0
●	●	○	○		0	0	1	0
○	○	●	○		0	0	1	0
●	○	●	○		0	0	1	0
○	●	●	○		1	0	0	0
●	●	●	○		1	0	0	0
○	○	○	●		1	0	0	0
●	○	○	●		0	1	0	0
○	●	○	●		0	0	1	0
●	●	○	●		0	0	0	1
○	○	●	●		0	0	1	0
●	○	●	●		0	0	0	1
○	●	●	●		1	0	0	0
●	●	●	●		0	1	0	0

Current state

The process is the same as when we marginalized-out **D** as a background condition:
We sum pairs of columns whose corresponding states differ only by **A**'s state

Calculating an effect repertoire:

Marginalizing-out non-purview elements

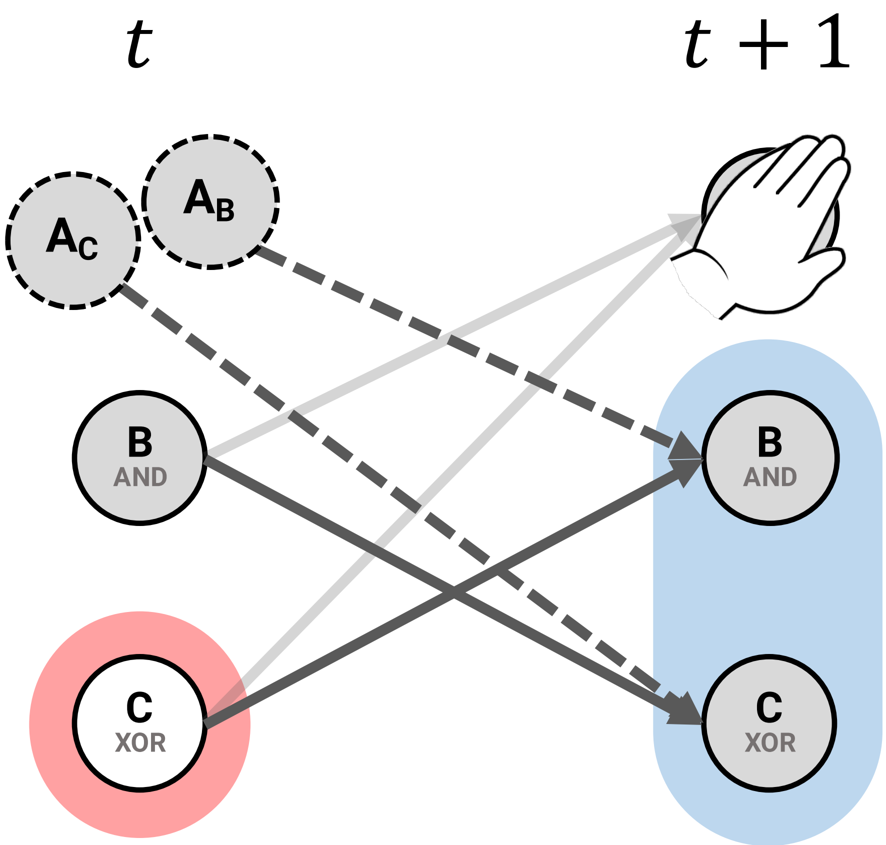

Next state

				B: ○ C: ○	B: ● C: ○	B: ○ C: ●	B: ● C: ●
A_B	A_C	B	C				
○	○	○	○	1	0	0	0
●	○	○	○	1	0	0	0
○	●	○	○	0	0	1	0
●	●	○	○	0	0	1	0
○	○	●	○	0	0	1	0
●	○	●	○	0	0	1	0
○	●	●	○	1	0	0	0
●	●	●	○	1	0	0	0
○	○	○	●	1	0	0	0
●	○	○	●	0	1	0	0
○	●	○	●	0	0	1	0
●	●	○	●	0	0	0	1
○	○	●	●	0	0	1	0
●	○	●	●	0	0	0	1
○	●	●	●	1	0	0	0
●	●	●	●	0	1	0	0

Current state

The process is the same as when we marginalized-out **D** as a background condition:
We sum pairs of columns whose corresponding states differ only by **A**'s state

Calculating an effect repertoire:
Marginalizing-out non-mechanism elements

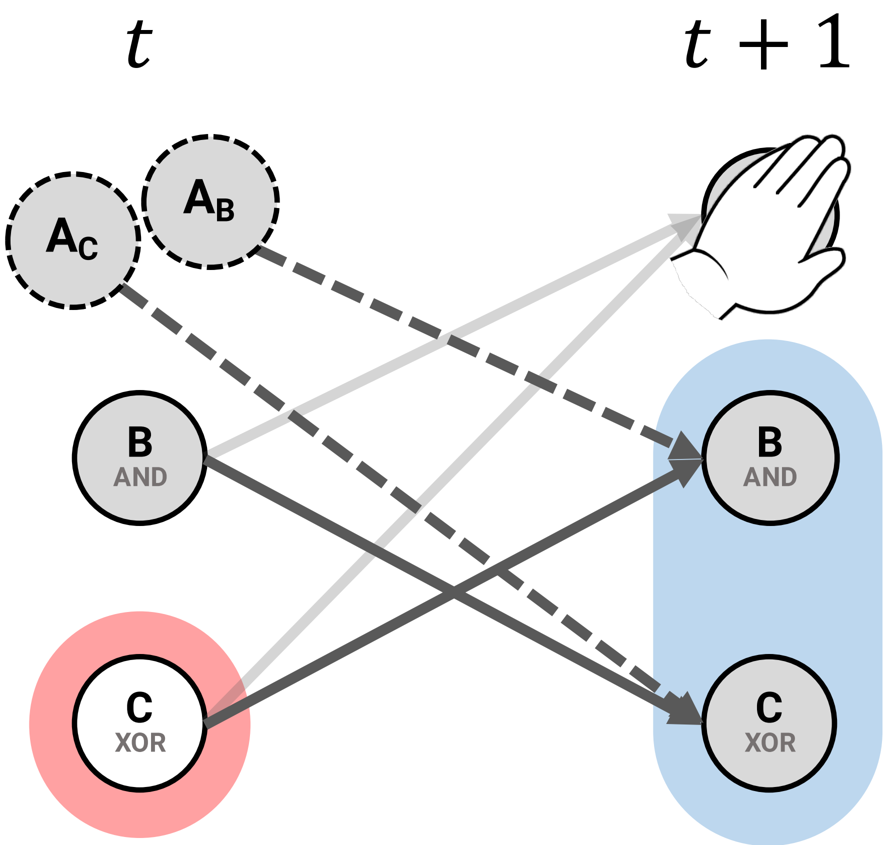

Next state

Current state

A_B	A_C	B	C	B: ○ C: ○	B: ● C: ○	B: ○ C: ●	B: ● C: ●
○	○	○	○	1	0	0	0
●	○	○	○	1	0	0	0
○	●	○	○	0	0	1	0
●	●	○	○	0	0	1	0
○	○	●	○	0	0	1	0
●	○	●	○	0	0	1	0
○	●	●	○	1	0	0	0
●	●	●	○	1	0	0	0
○	○	○	●	1	0	0	0
●	○	○	●	0	1	0	0
○	●	○	●	0	0	1	0
●	●	○	●	0	0	0	1
○	○	●	●	0	0	1	0
●	○	●	●	0	0	0	1
○	●	●	●	1	0	0	0
●	●	●	●	0	1	0	0

Now, to find the effect repertoire of the mechanism, **C**, as before, we want a TPM that gives the probabilities of next purview states given **only** the current state of **C**

Calculating an effect repertoire:
Marginalizing-out non-mechanism elements

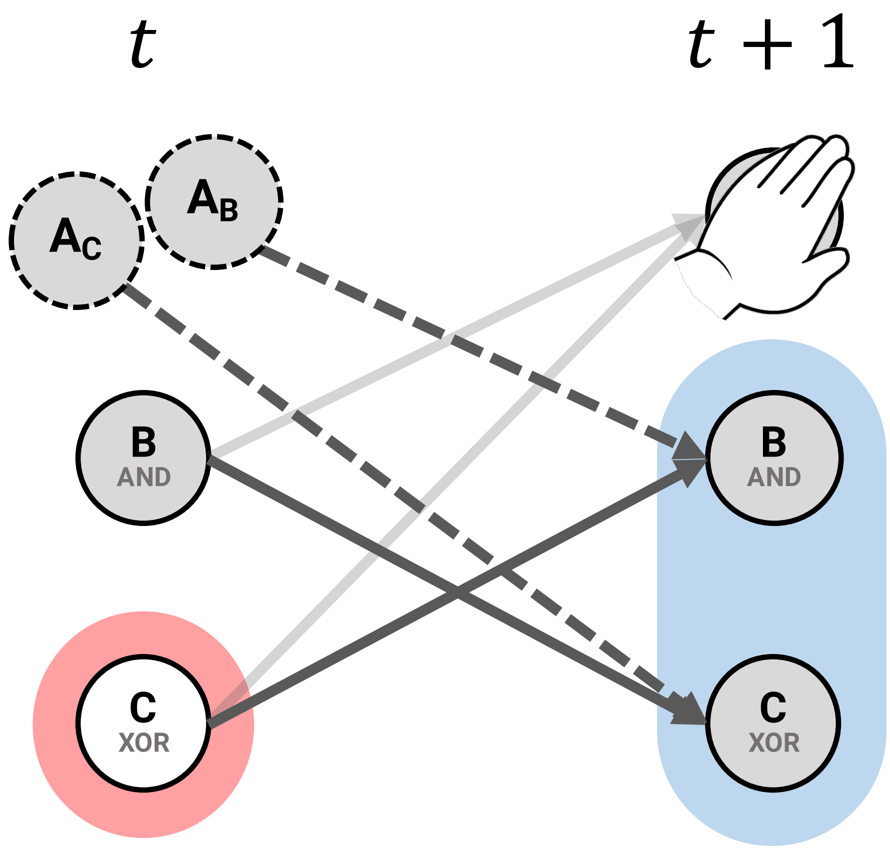

Next state

Current state: A_B	A_C	B	C	B ○, C ○	B ●, C ○	B ○, C ●	B ●, C ●
○	○	○	○	1	0	0	0
●	○	○	○	1	0	0	0
○	●	○	○	0	0	1	0
●	●	○	○	0	0	1	0
○	○	●	○	0	0	1	0
●	○	●	○	0	0	1	0
○	●	●	○	1	0	0	0
●	●	●	○	1	0	0	0
○	○	○	●	1	0	0	0
●	○	○	●	0	1	0	0
○	●	○	●	0	0	1	0
●	●	○	●	0	0	0	1
○	○	●	●	0	0	1	0
●	○	●	●	0	0	0	1
○	●	●	●	1	0	0	0
●	●	●	●	0	1	0	0

In other words, we want to ignore the current state of the non-mechanism elements—so we marginalize them out of the TPM

Calculating an effect repertoire:
Marginalizing-out non-mechanism elements

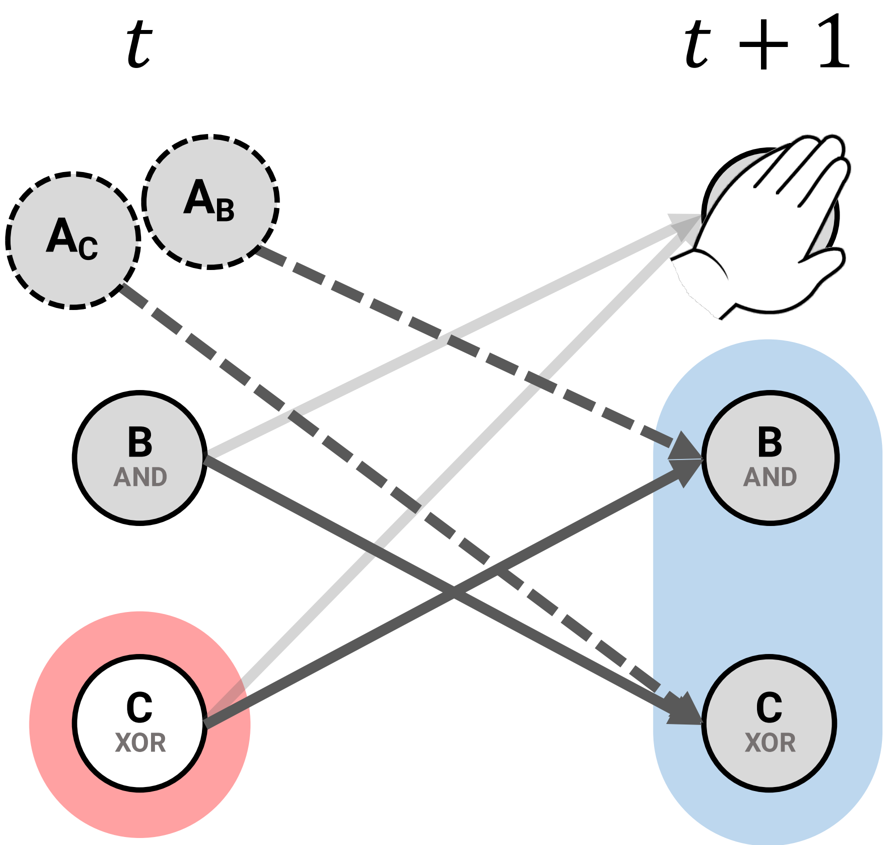

Next state

Current state

				B	○	●	○	●
				C	○	○	●	●
A_B	A_C	B	C					
○	○	○	○		1	0	0	0
●	○	○	○		1	0	0	0
○	●	○	○		0	0	1	0
●	●	○	○		0	0	1	0
○	○	●	○		0	0	1	0
●	○	●	○		0	0	1	0
○	●	●	○		1	0	0	0
●	●	●	○		1	0	0	0
○	○	○	●		1	0	0	0
●	○	○	●		0	1	0	0
○	●	○	●		0	0	1	0
●	●	○	●		0	0	0	1
○	○	●	●		0	0	1	0
●	○	●	●		0	0	0	1
○	●	●	●		1	0	0	0
●	●	●	●		0	1	0	0

As with the previous example, marginalizing over the current states of elements means we sum over rows, rather than columns (and renormalize the resulting rows)

Calculating an effect repertoire:
Marginalizing-out non-mechanism elements

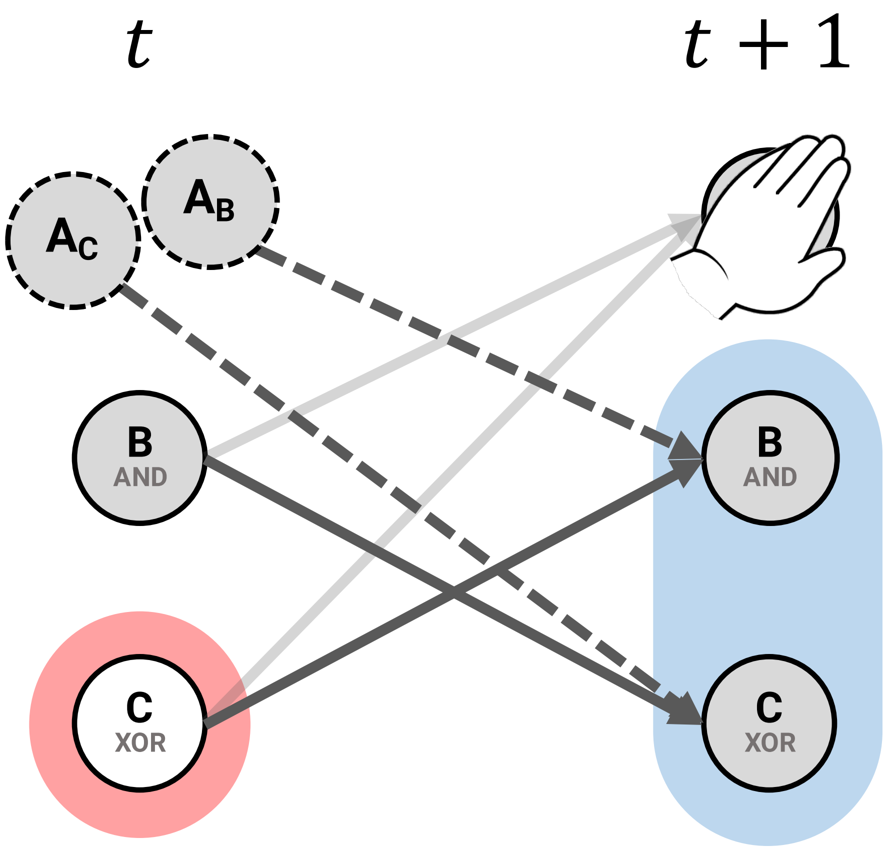

First we'll marginalize-out $\mathbf{A_B}$

Next state

					○	●	○	●
				C	○	○	●	●
A_B	A_C	B	C					
○	○	○	○		1	0	0	0
●	○	○	○		1	0	0	0
○	●	○	○		0	0	1	0
●	●	○	○		0	0	1	0
○	○	●	○		0	0	1	0
●	○	●	○		0	0	1	0
○	●	●	○		1	0	0	0
●	●	●	○		1	0	0	0
○	○	○	●		1	0	0	0
●	○	○	●		0	1	0	0
○	●	○	●		0	0	1	0
●	●	○	●		0	0	0	1
○	○	●	●		0	0	1	0
●	○	●	●		0	0	0	1
○	●	●	●		1	0	0	0
●	●	●	●		0	1	0	0

Current state

Calculating an effect repertoire:
Marginalizing-out non-mechanism elements

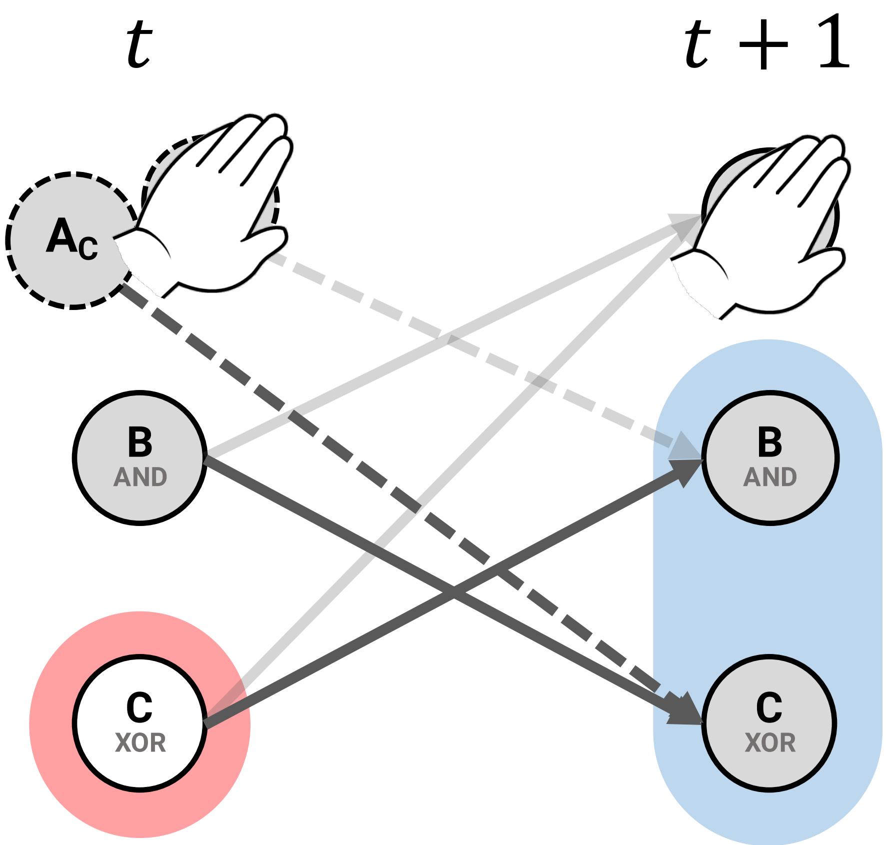

Next state

Current state: A_B	A_C	B	C	B: ○ C: ○	B: ● C: ○	B: ○ C: ●	B: ● C: ●
○	○	○	○	1	0	0	0
●	○	○	○	1	0	0	0
○	●	○	○	0	0	1	0
●	●	○	○	0	0	1	0
○	○	●	○	0	0	1	0
●	○	●	○	0	0	1	0
○	●	●	○	1	0	0	0
●	●	●	○	1	0	0	0
○	○	○	●	1	0	0	0
●	○	○	●	0	1	0	0
○	●	○	●	0	0	1	0
●	●	○	●	0	0	0	1
○	○	●	●	0	0	1	0
●	○	●	●	0	0	0	1
○	●	●	●	1	0	0	0
●	●	●	●	0	1	0	0

First we'll marginalize-out $\mathbf{A_B}$

Calculating an effect repertoire:
Marginalizing-out non-mechanism elements

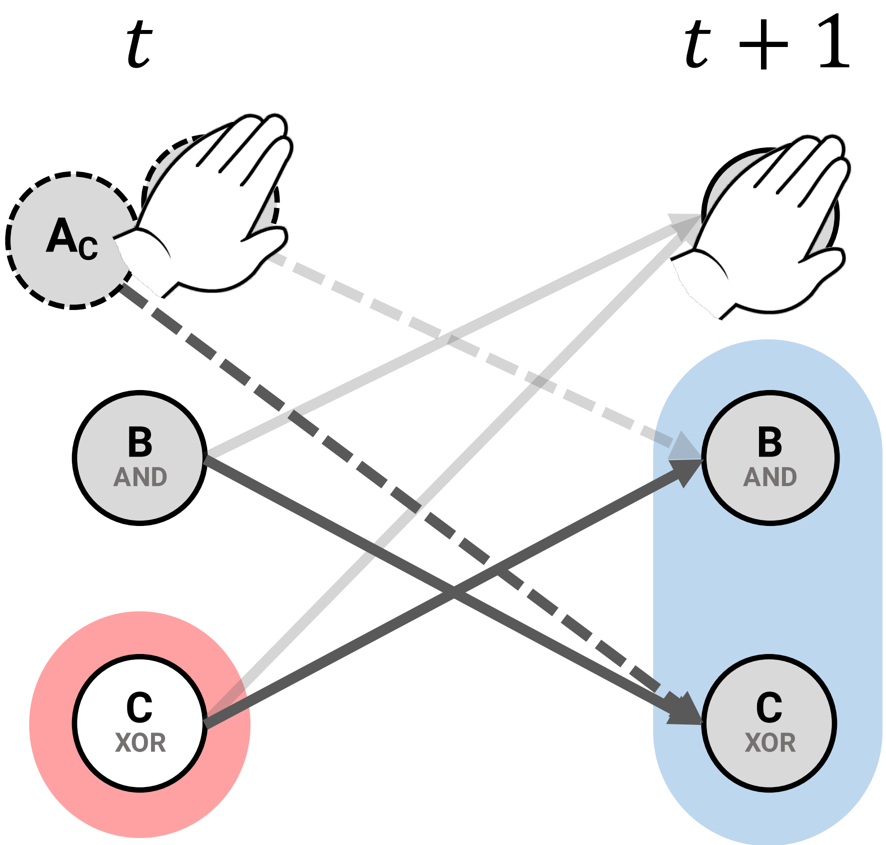

First we'll marginalize-out $\mathbf{A_B}$

Next state

				B	○	●	○	●
				C	○	○	●	●
A_B	A_C	B	C					
○	○	○	○		1	0	0	0
●	○	○	○		1	0	0	0
○	●	○	○		0	0	1	0
●	●	○	○		0	0	1	0
○	○	●	○		0	0	1	0
●	○	●	○		0	0	1	0
○	●	●	○		1	0	0	0
●	●	●	○		1	0	0	0
○	○	○	●		1	0	0	0
●	○	○	●		0	1	0	0
○	●	○	●		0	0	1	0
●	●	○	●		0	0	0	1
○	○	●	●		0	0	1	0
●	○	●	●		0	0	0	1
○	●	●	●		1	0	0	0
●	●	●	●		0	1	0	0

Current state

Calculating an effect repertoire:
Marginalizing-out non-mechanism elements

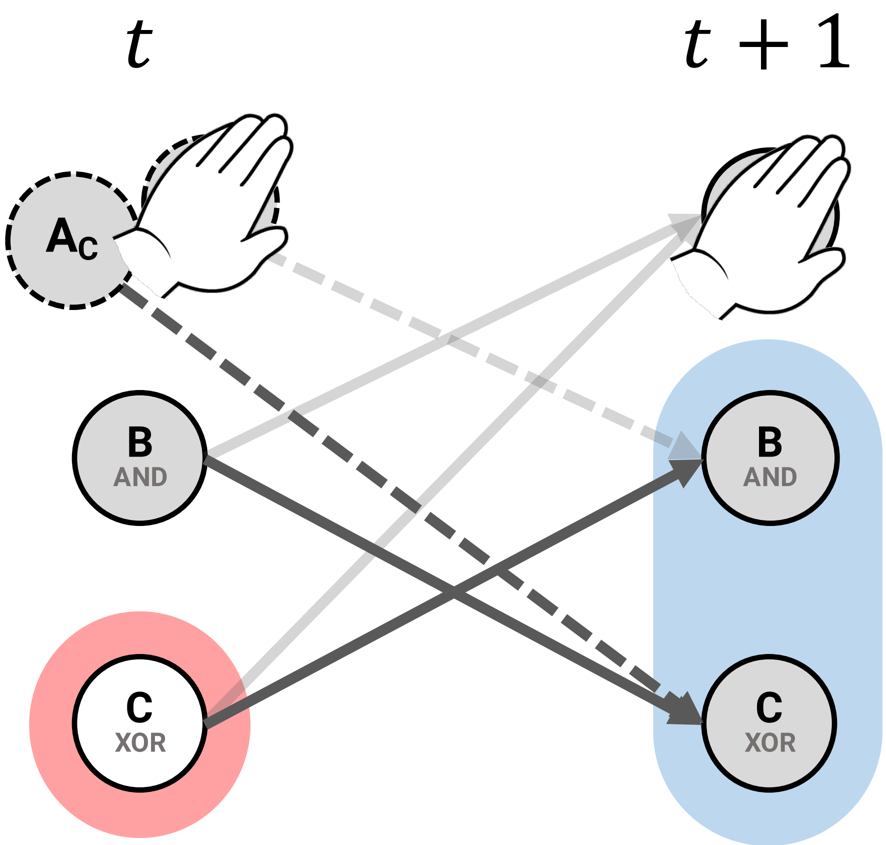

First we'll marginalize-out $\mathbf{A_B}$

Next state

Current state

A_C	B	C	B: ○ / C: ○	B: ● / C: ○	B: ○ / C: ●	B: ● / C: ●
○	○	○	1	0	0	0
○	○	○	1	0	0	0
●	○	○	0	0	1	0
●	○	○	0	0	1	0
○	●	○	0	0	1	0
○	●	○	0	0	1	0
●	●	○	1	0	0	0
●	●	○	1	0	0	0
○	○	●	1	0	0	0
○	○	●	0	1	0	0
●	○	●	0	0	1	0
●	○	●	0	0	0	1
○	●	●	0	0	1	0
○	●	●	0	0	0	1
●	●	●	1	0	0	0
●	●	●	0	1	0	0

Calculating an effect repertoire:
Marginalizing-out non-mechanism elements

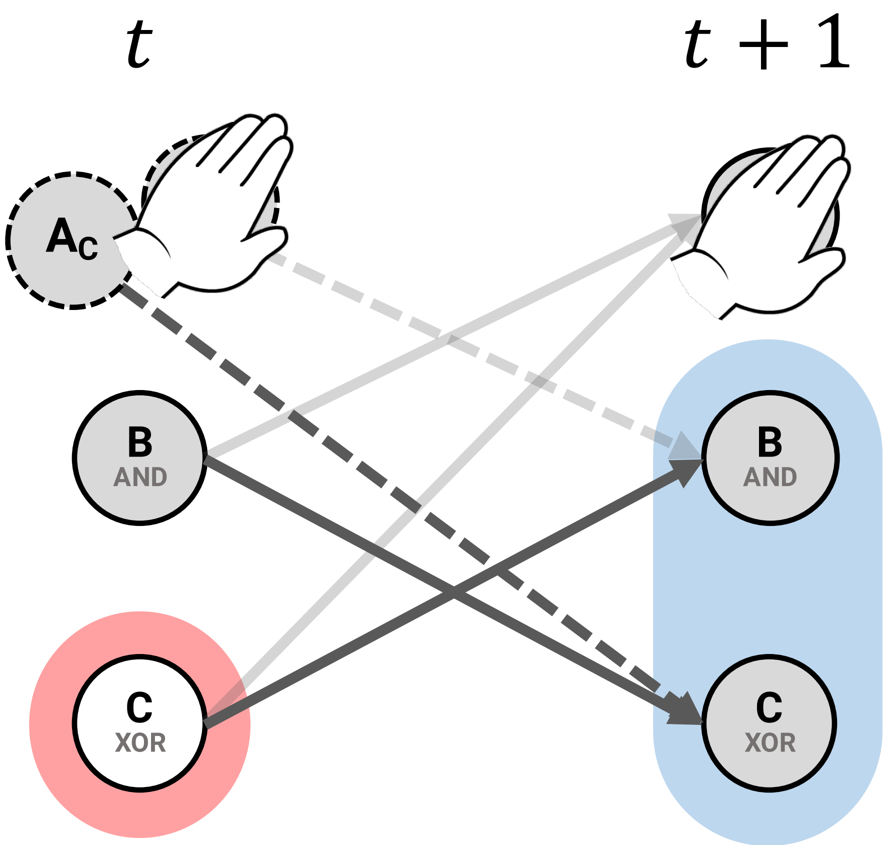

First we'll marginalize-out $\mathbf{A_B}$

Next state

Current state: A_C	B	C	Next B ○, C ○	Next B ●, C ○	Next B ○, C ●	Next B ●, C ●
○	○	○	2	0	0	0
●	○	○	0	0	2	0
○	●	○	0	0	2	0
●	●	○	2	0	0	0
○	○	●	1	1	0	0
●	○	●	0	0	1	1
○	●	●	0	0	1	1
●	●	●	1	1	0	0

Calculating an effect repertoire:

Marginalizing-out non-mechanism elements

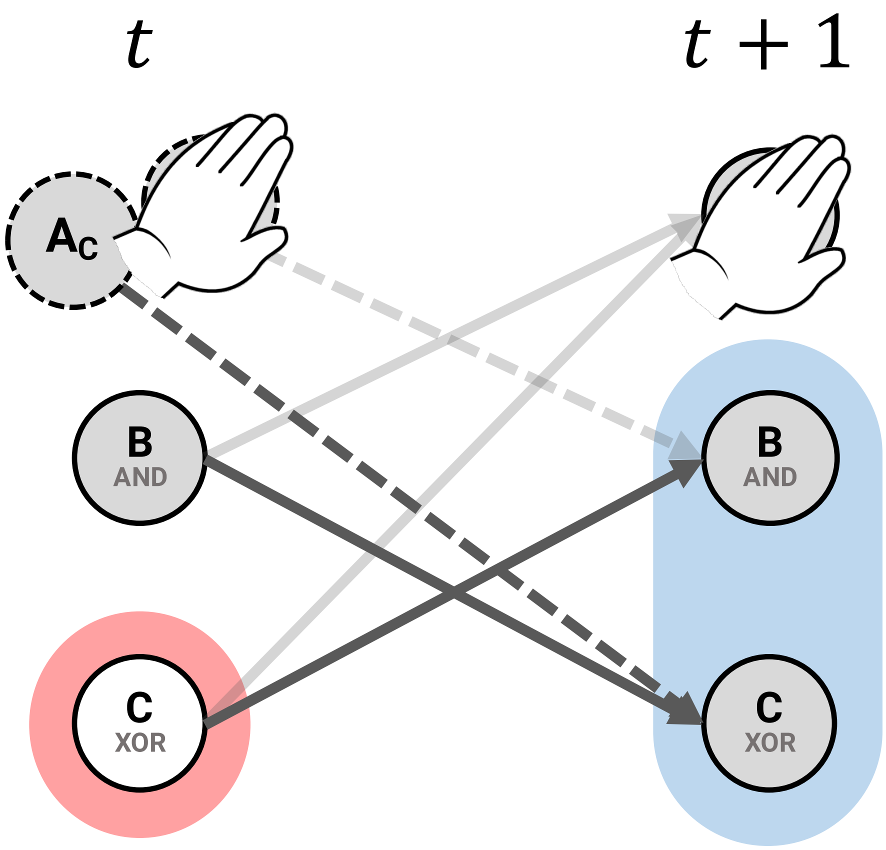

Next state

Current state: A_C	B	C	B ○ / C ○	B ● / C ○	B ○ / C ●	B ● / C ●
○	○	○	1	0	0	0
●	○	○	0	0	1	0
○	●	○	0	0	1	0
●	●	○	1	0	0	0
○	○	●	$1/2$	$1/2$	0	0
●	○	●	0	0	$1/2$	$1/2$
○	●	●	0	0	$1/2$	$1/2$
●	●	●	$1/2$	$1/2$	0	0

First we'll marginalize-out $\mathbf{A_B}$

Calculating an effect repertoire:
Marginalizing-out non-mechanism elements

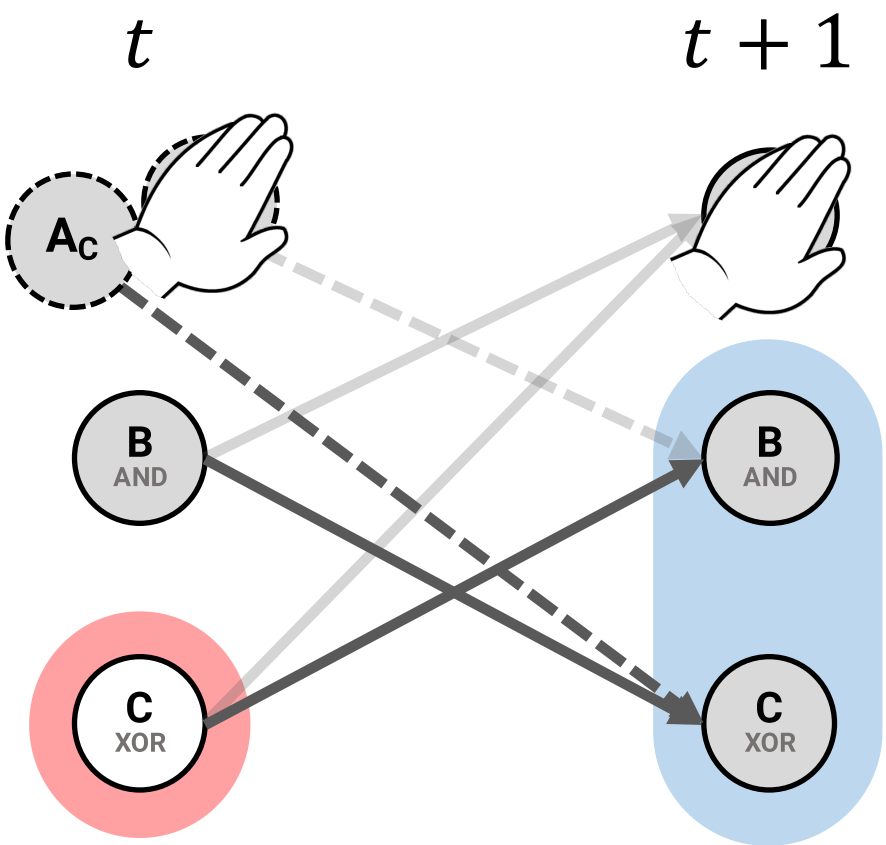

Next state

Current state: A_C	B	C	B: ○, C: ○	B: ●, C: ○	B: ○, C: ●	B: ●, C: ●
○	○	○	1	0	0	0
●	○	○	0	0	1	0
○	●	○	0	0	1	0
●	●	○	1	0	0	0
○	○	●	$1/2$	$1/2$	0	0
●	○	●	0	0	$1/2$	$1/2$
○	●	●	0	0	$1/2$	$1/2$
●	●	●	$1/2$	$1/2$	0	0

Now we’ll marginalize-out **A$_C$**

Calculating an effect repertoire:
Marginalizing-out non-mechanism elements

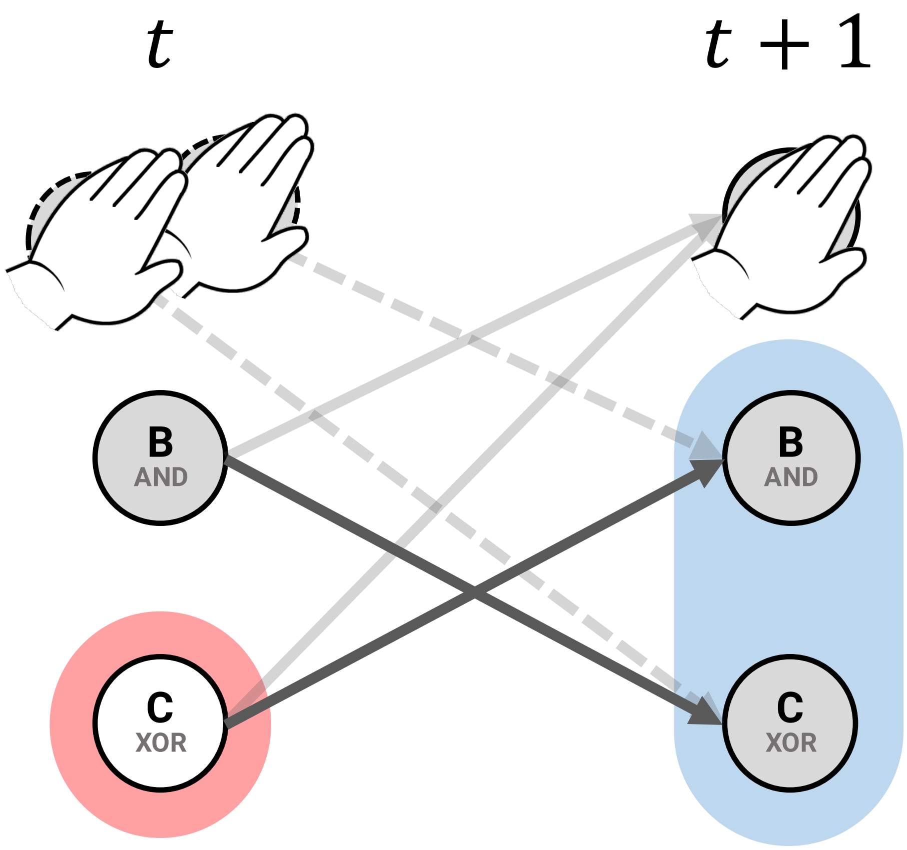

Now we'll marginalize-out $\mathbf{A_C}$

Next state

Current state: A_C	B	C	Next state B: ○, C: ○	B: ●, C: ○	B: ○, C: ●	B: ●, C: ●
○	○	○	1	0	0	0
●	○	○	0	0	1	0
○	●	○	0	0	1	0
●	●	○	1	0	0	0
○	○	●	$^1/_2$	$^1/_2$	0	0
●	○	●	0	0	$^1/_2$	$^1/_2$
○	●	●	0	0	$^1/_2$	$^1/_2$
●	●	●	$^1/_2$	$^1/_2$	0	0

Calculating an effect repertoire:
Marginalizing-out non-mechanism elements

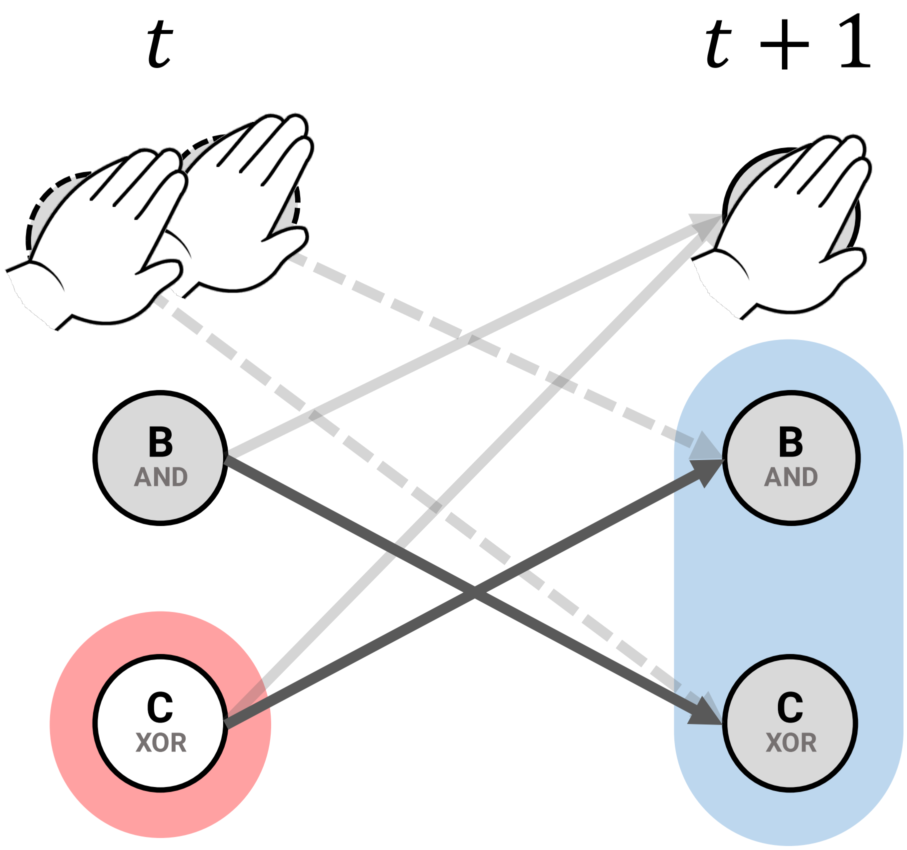

Next state

Current state A_C	B	C	B ○ C ○	B ● C ○	B ○ C ●	B ● C ●
○	○	○	1	0	0	0
●	○	○	0	0	1	0
○	●	○	0	0	1	0
●	●	○	1	0	0	0
○	○	●	$^1/_2$	$^1/_2$	0	0
●	○	●	0	0	$^1/_2$	$^1/_2$
○	●	●	0	0	$^1/_2$	$^1/_2$
●	●	●	$^1/_2$	$^1/_2$	0	0

Now we'll marginalize-out $\mathbf{A_C}$

Calculating an effect repertoire:

Marginalizing-out non-mechanism elements

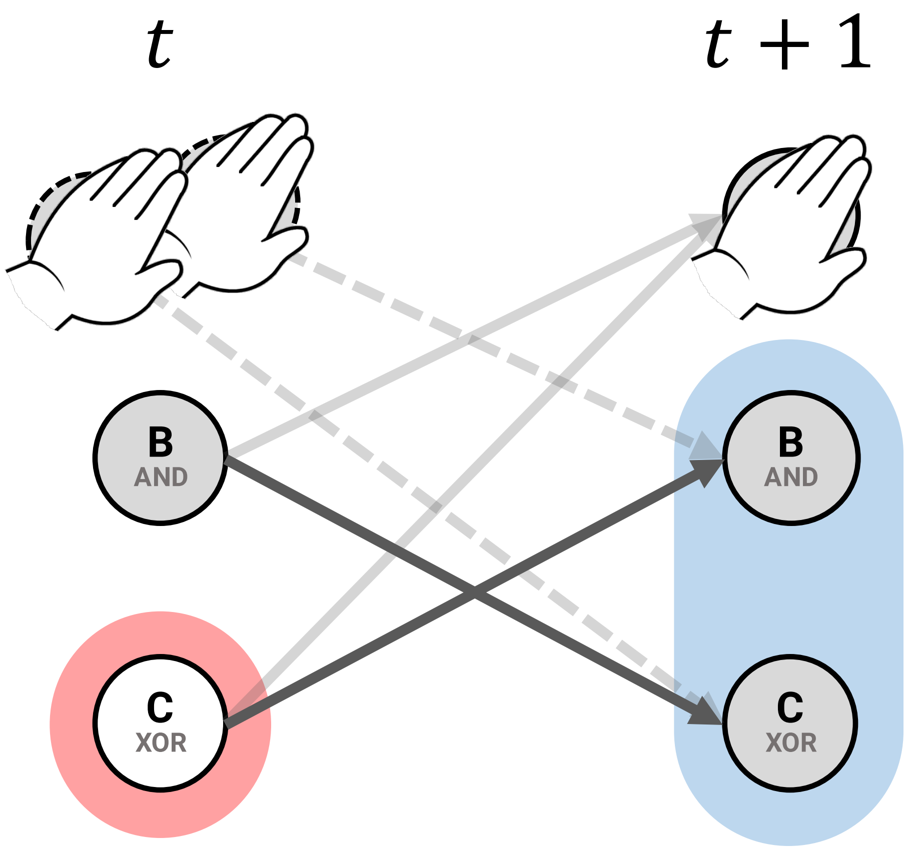

Now we'll marginalize-out $\mathbf{A_C}$

Next state

Current state: B	Current state: C	B ○ / C ○	B ● / C ○	B ○ / C ●	B ● / C ●
○	○	1	0	1	0
●	○	1	0	1	0
○	●	$1/2$	$1/2$	$1/2$	$1/2$
●	●	$1/2$	$1/2$	$1/2$	$1/2$

Calculating an effect repertoire:
Marginalizing-out non-mechanism elements

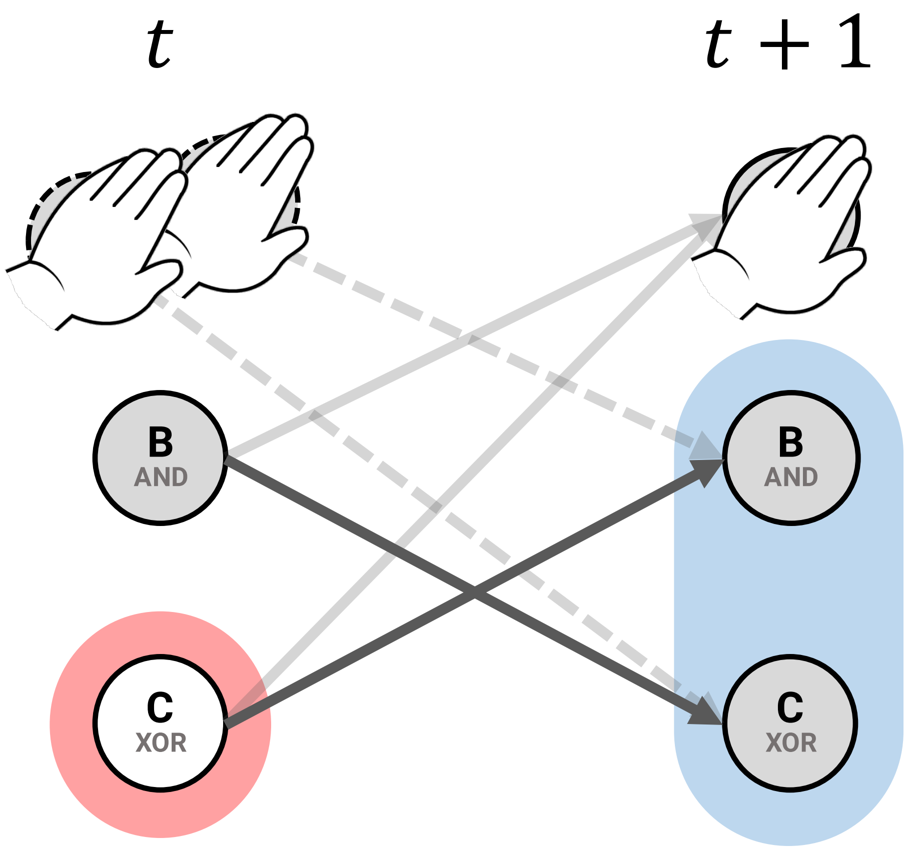

Now we'll marginalize-out $\mathbf{A_C}$

Next state

Current state B	Current state C	B ○ C ○	B ● C ○	B ○ C ●	B ● C ●
○	○	$^1/_2$	0	$^1/_2$	0
●	○	$^1/_2$	0	$^1/_2$	0
○	●	$^1/_4$	$^1/_4$	$^1/_4$	$^1/_4$
●	●	$^1/_4$	$^1/_4$	$^1/_4$	$^1/_4$

Calculating an effect repertoire:

Marginalizing-out non-mechanism elements

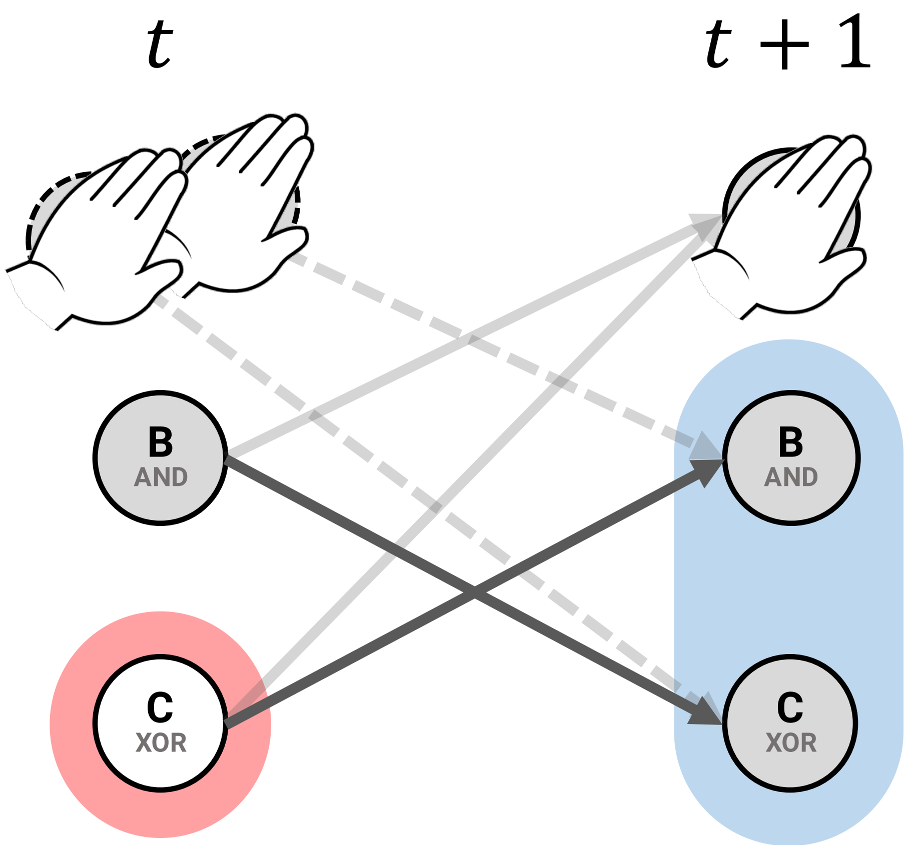

And finally we'll marginalize-out **B**

Next state (columns) / Current state (rows)

Current state B	Current state C	Next B: ○, C: ○	Next B: ●, C: ○	Next B: ○, C: ●	Next B: ●, C: ●
○	○	$1/2$	0	$1/2$	0
●	○	$1/2$	0	$1/2$	0
○	●	$1/4$	$1/4$	$1/4$	$1/4$
●	●	$1/4$	$1/4$	$1/4$	$1/4$

Calculating an effect repertoire:
Marginalizing-out non-mechanism elements

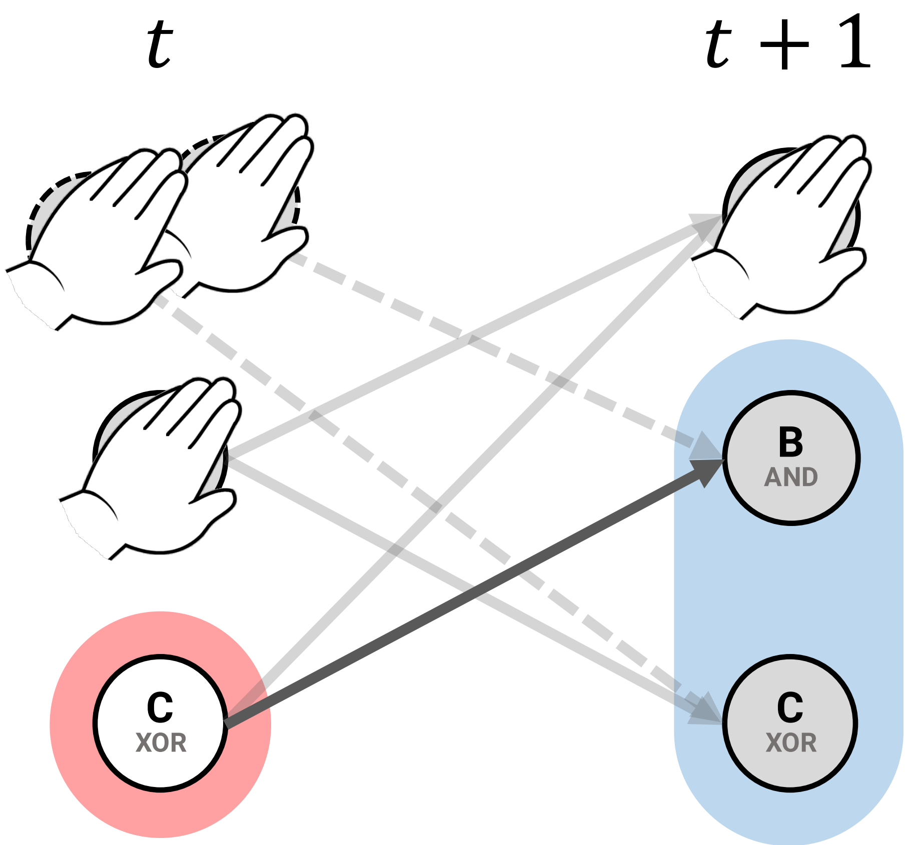

Next state

Current state B	Current state C	B ○ C ○	B ● C ○	B ○ C ●	B ● C ●
○	○	$^1/_2$	0	$^1/_2$	0
●	○	$^1/_2$	0	$^1/_2$	0
○	●	$^1/_4$	$^1/_4$	$^1/_4$	$^1/_4$
●	●	$^1/_4$	$^1/_4$	$^1/_4$	$^1/_4$

And finally we'll marginalize-out **B**

Calculating an effect repertoire:
Marginalizing-out non-mechanism elements

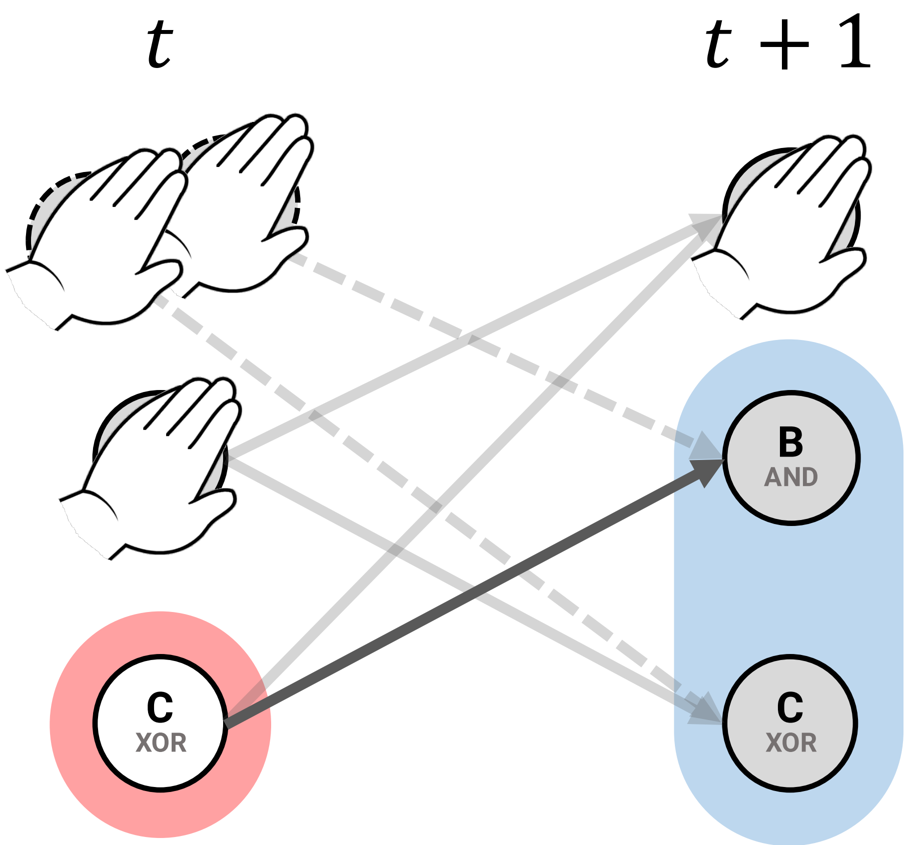

And finally we'll marginalize-out **B**

Next state

Current state (B, C)	B ○, C ○	B ●, C ○	B ○, C ●	B ●, C ●
○ ○	$1/2$	0	$1/2$	0
● ○	$1/2$	0	$1/2$	0
○ ●	$1/4$	$1/4$	$1/4$	$1/4$
● ●	$1/4$	$1/4$	$1/4$	$1/4$

Calculating an effect repertoire:
Marginalizing-out non-mechanism elements

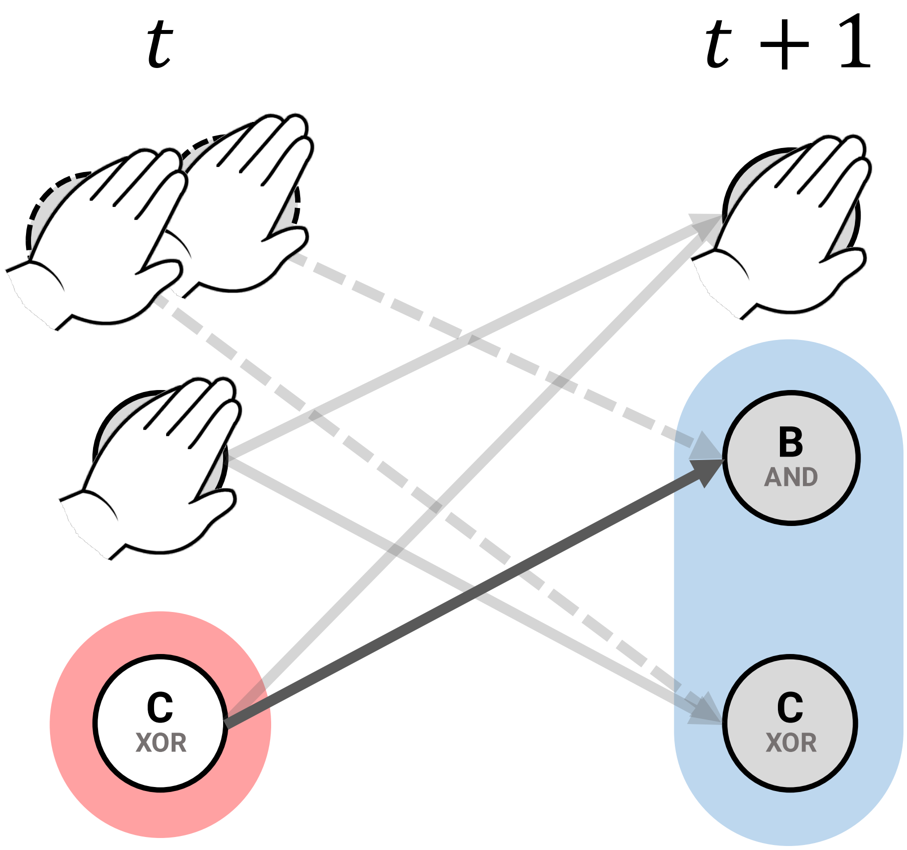

Next state

**B**	○	●	○	●
**C**	○	○	●	●
**C** (Current state)				
○	$^1/_2$	0	$^1/_2$	0
○	$^1/_2$	0	$^1/_2$	0
●	$^1/_4$	$^1/_4$	$^1/_4$	$^1/_4$
●	$^1/_4$	$^1/_4$	$^1/_4$	$^1/_4$

And finally we'll marginalize-out **B**

Calculating an effect repertoire:
Marginalizing-out non-mechanism elements

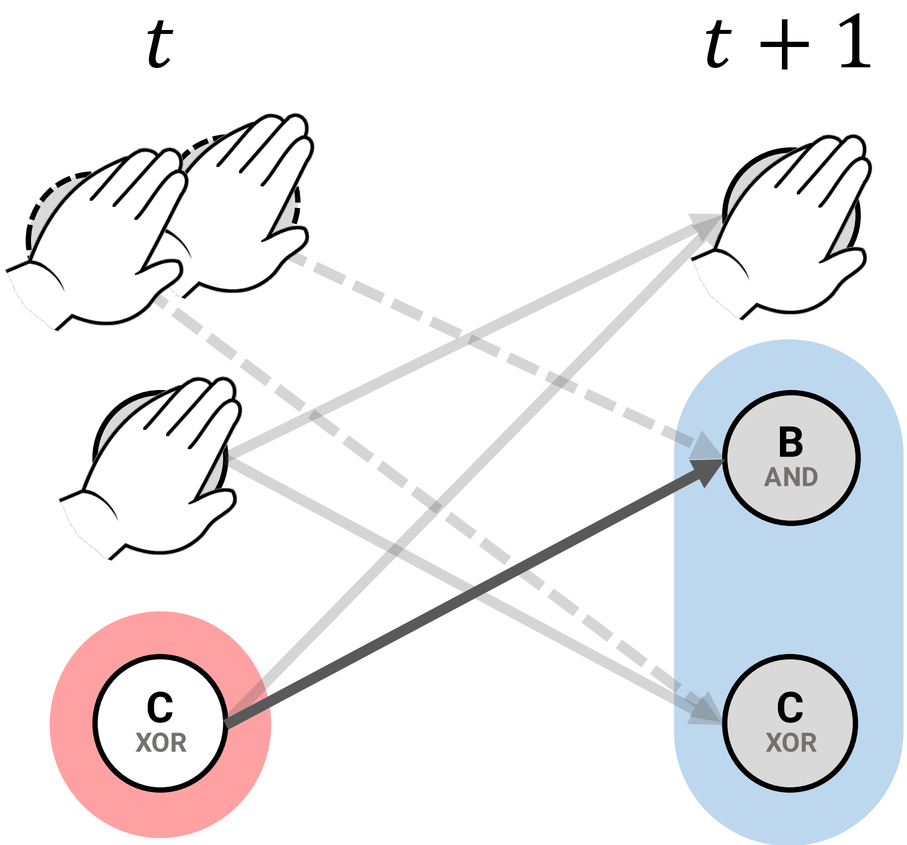

And finally we'll marginalize-out **B**

Next state

	B	○	●	○	●
	**C**	○	○	●	●
Current state: **C**					
○		1	0	1	0
●		$^1/_2$	$^1/_2$	$^1/_2$	$^1/_2$

Calculating an effect repertoire:
Marginalizing-out non-mechanism elements

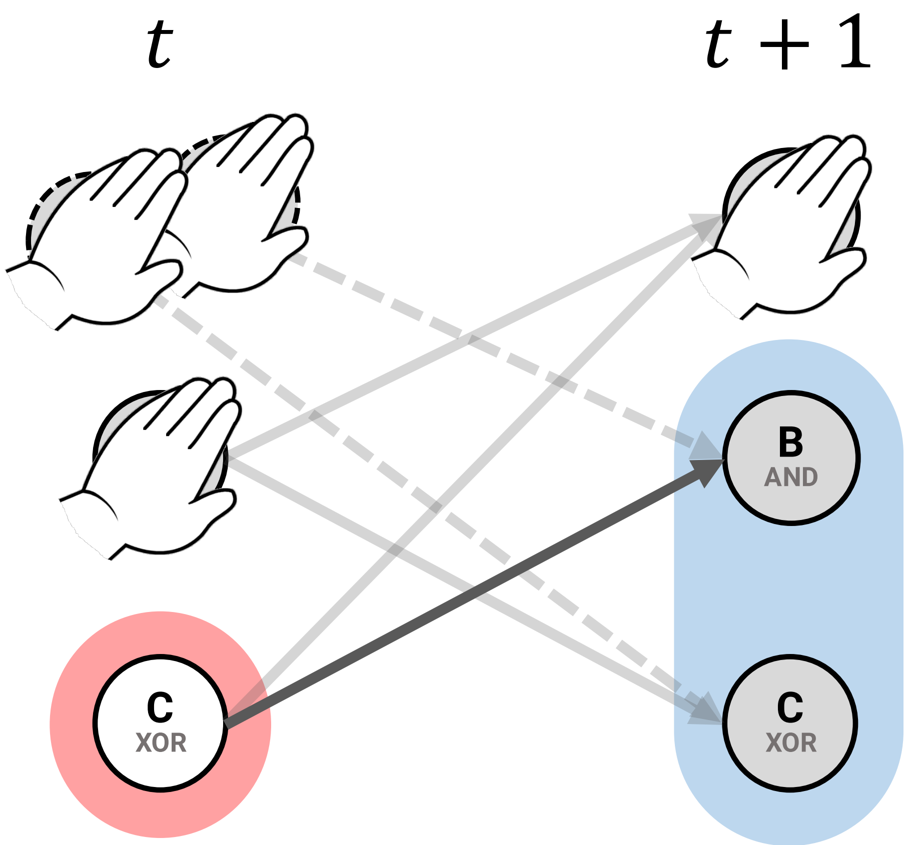

		Next state			
	**B**	○	●	○	●
	**C**	○	○	●	●
Current state	**C**				
	○	$^1/_2$	0	$^1/_2$	0
	●	$^1/_4$	$^1/_4$	$^1/_4$	$^1/_4$

And finally we'll marginalize-out **B**

Calculating an effect repertoire:
Marginalizing-out non-mechanism elements

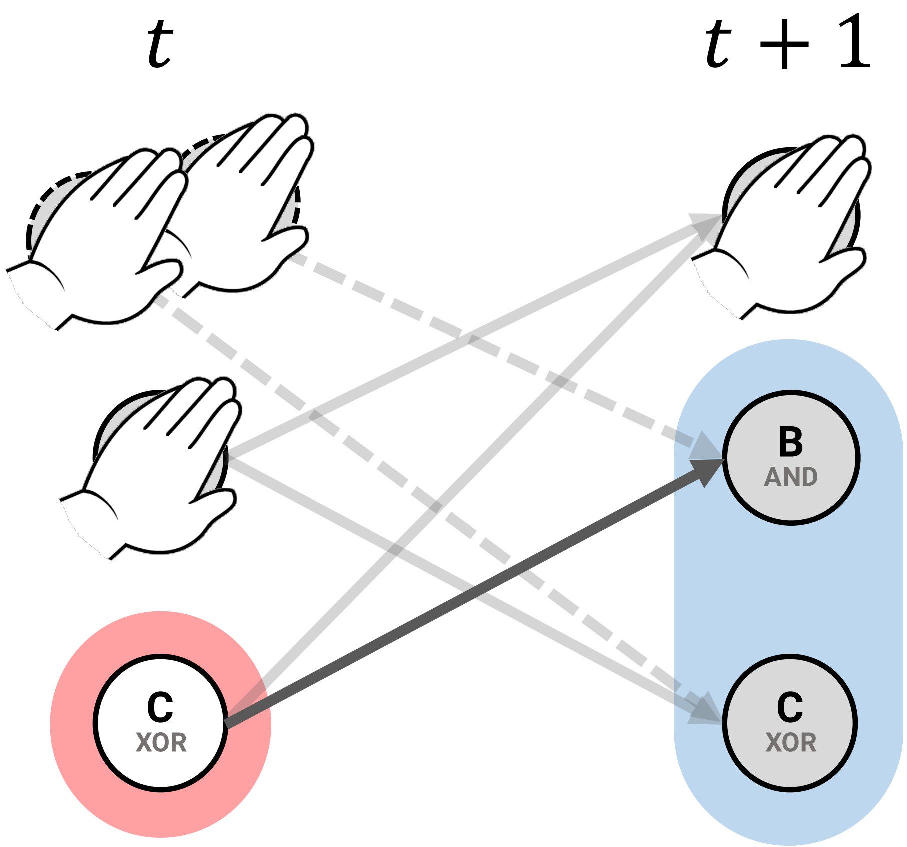

And finally we'll marginalize-out **B**

Next state

Current state					
	B	○	●	○	●
	C	○	○	●	●
C					
○		$^1/_2$	0	$^1/_2$	0
●		$^1/_4$	$^1/_4$	$^1/_4$	$^1/_4$

Calculating an effect repertoire:
Marginalizing-out non-mechanism elements

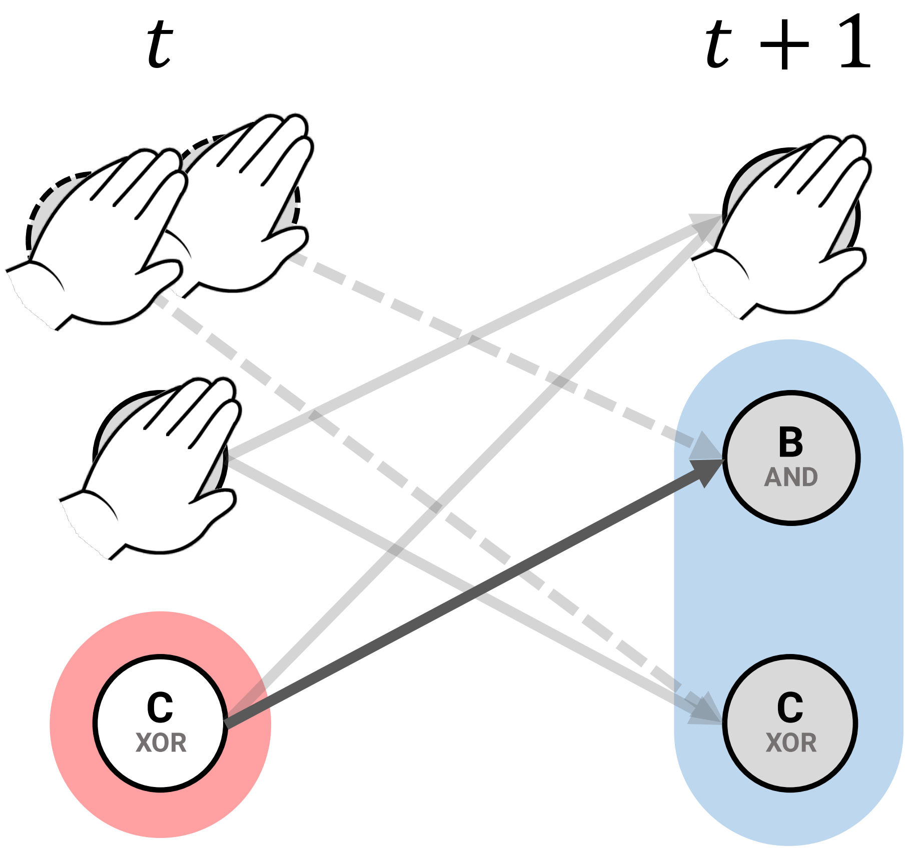

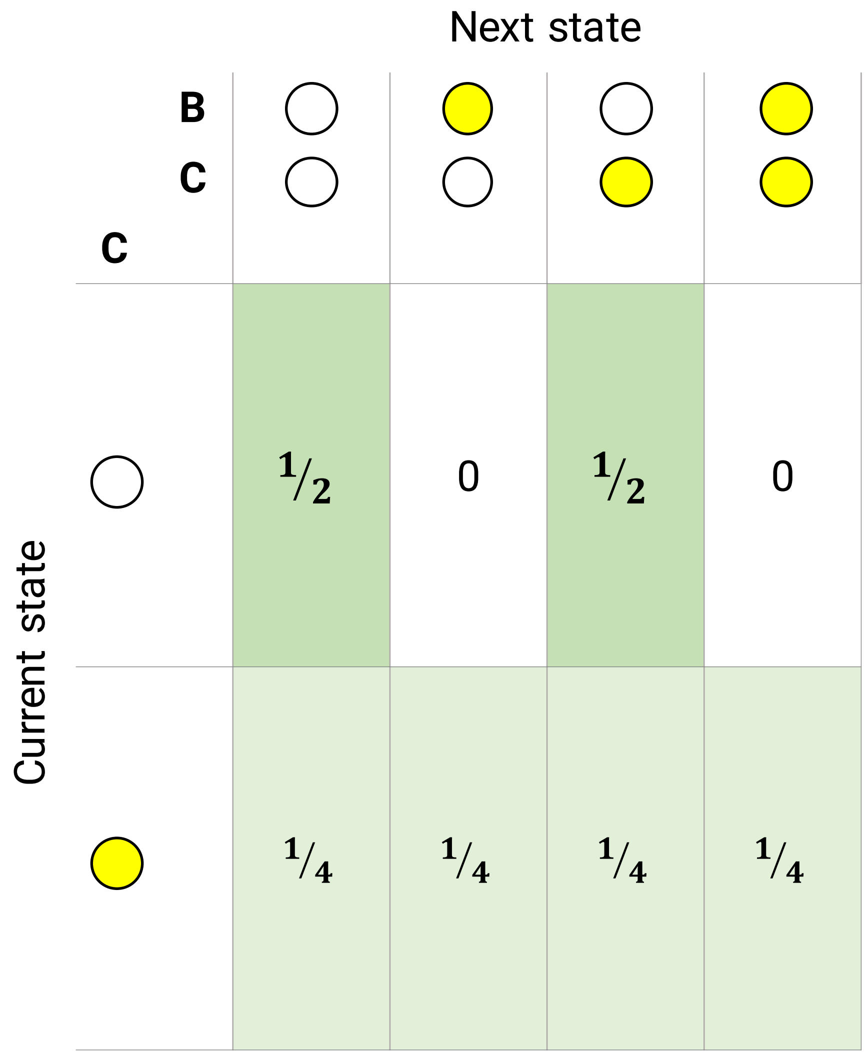

Now we have a table of probabilities of next purview states given each possible current state of **C**

Calculating an effect repertoire:
Marginalizing-out non-mechanism elements

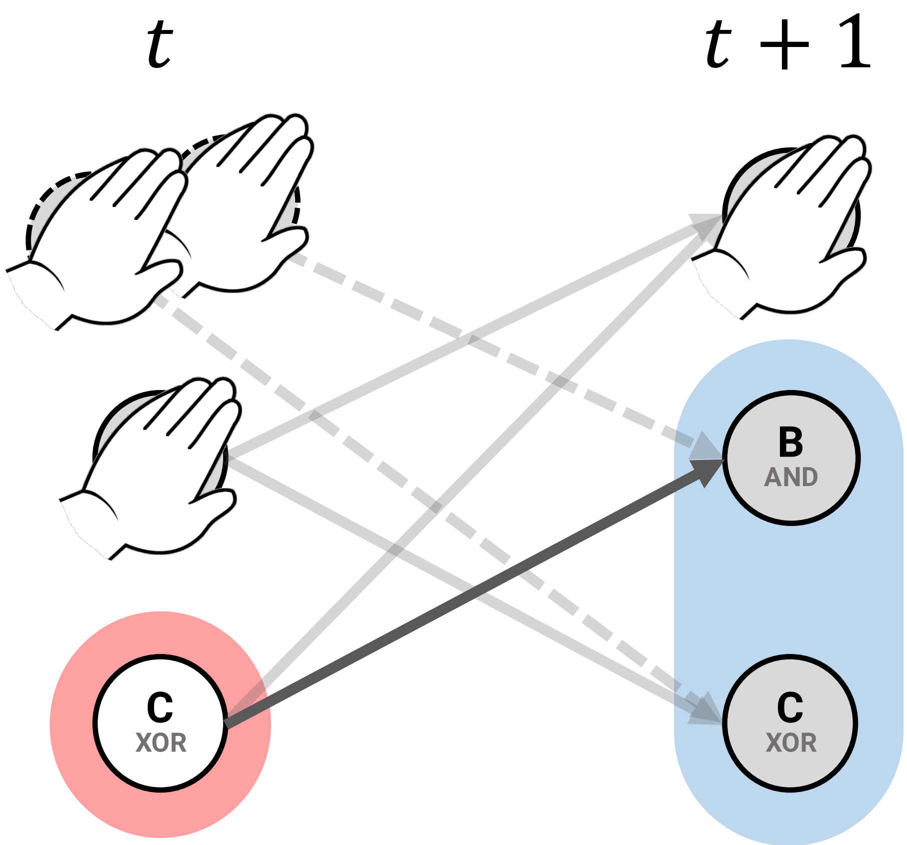

Next state

	B	○	●	○	●
	**C**	○	○	●	●
**C** (Current state)					
○		$^1/_2$	0	$^1/_2$	0
●		$^1/_4$	$^1/_4$	$^1/_4$	$^1/_4$

With this TPM, we can now simply look up the effect repertoire, by conditioning on **C**'s current state (taking the row that corresponds to it)

Calculating an effect repertoire:

Marginalizing-out non-mechanism elements

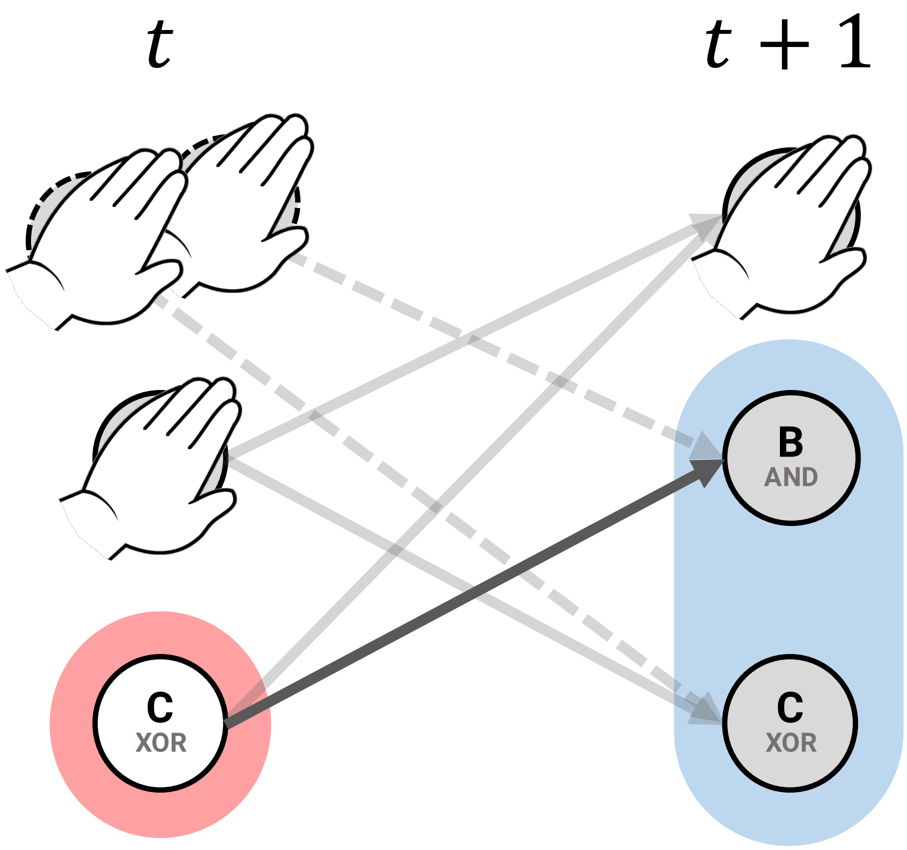

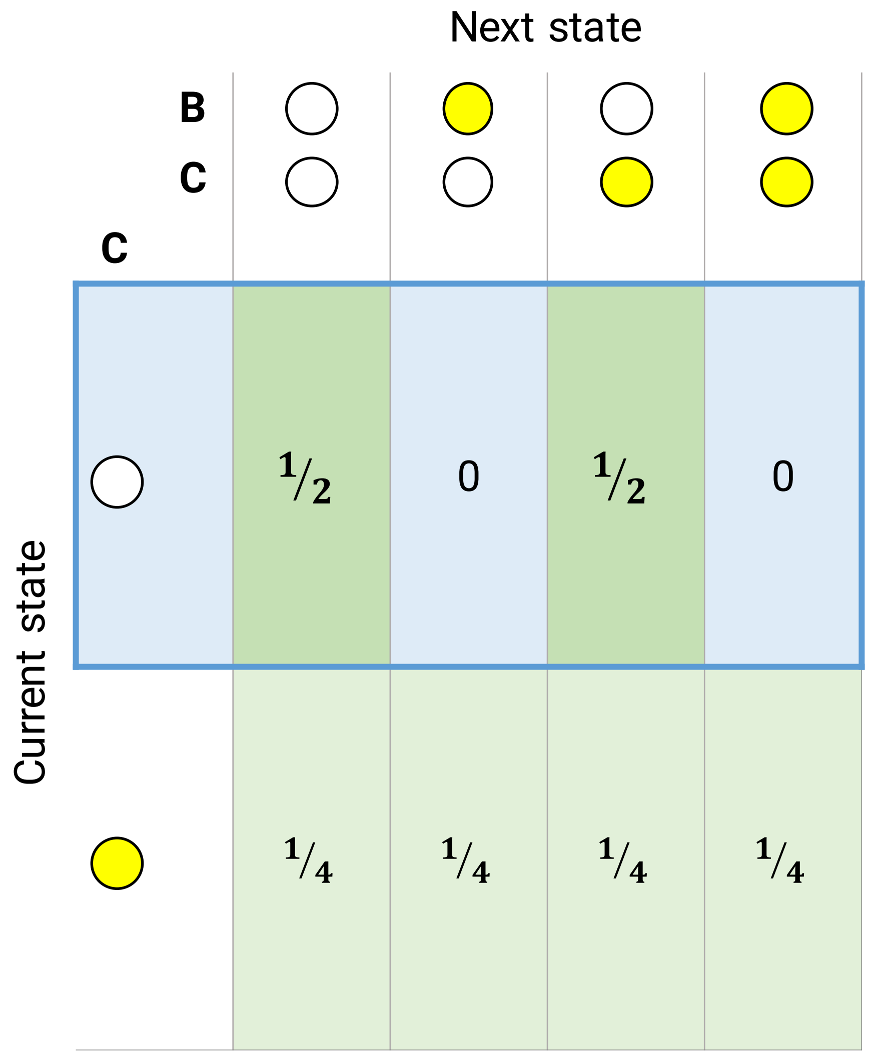

With this TPM, we can now simply look up the effect repertoire, by conditioning on **C**'s current state (taking the row that corresponds to it)

Calculating an effect repertoire:
Marginalizing-out non-mechanism elements

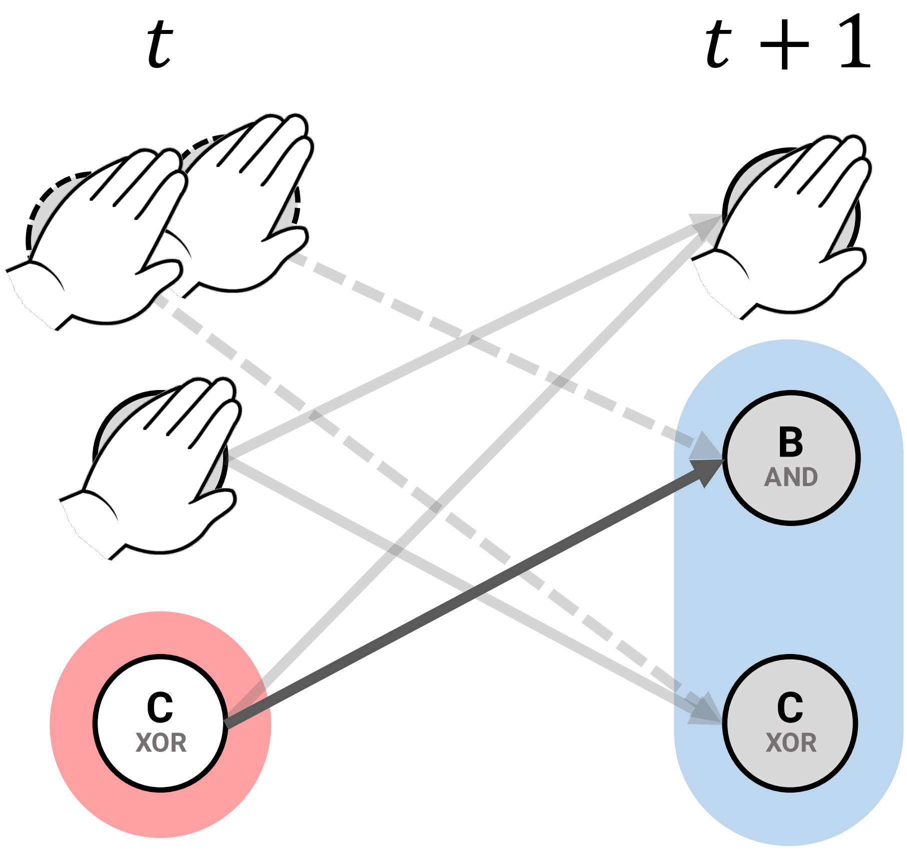

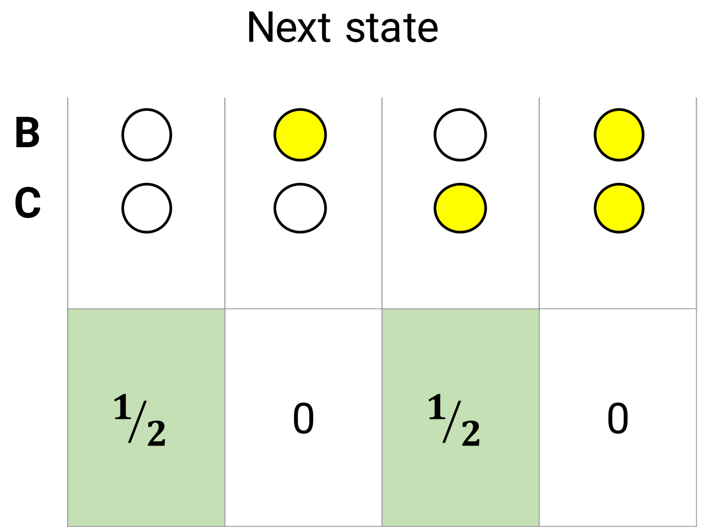

And this is the effect repertoire of mechanism **C** over purview **BC** when the system is in state (1, 0, 0)

Calculating an effect repertoire:
Marginalizing-out non-mechanism elements

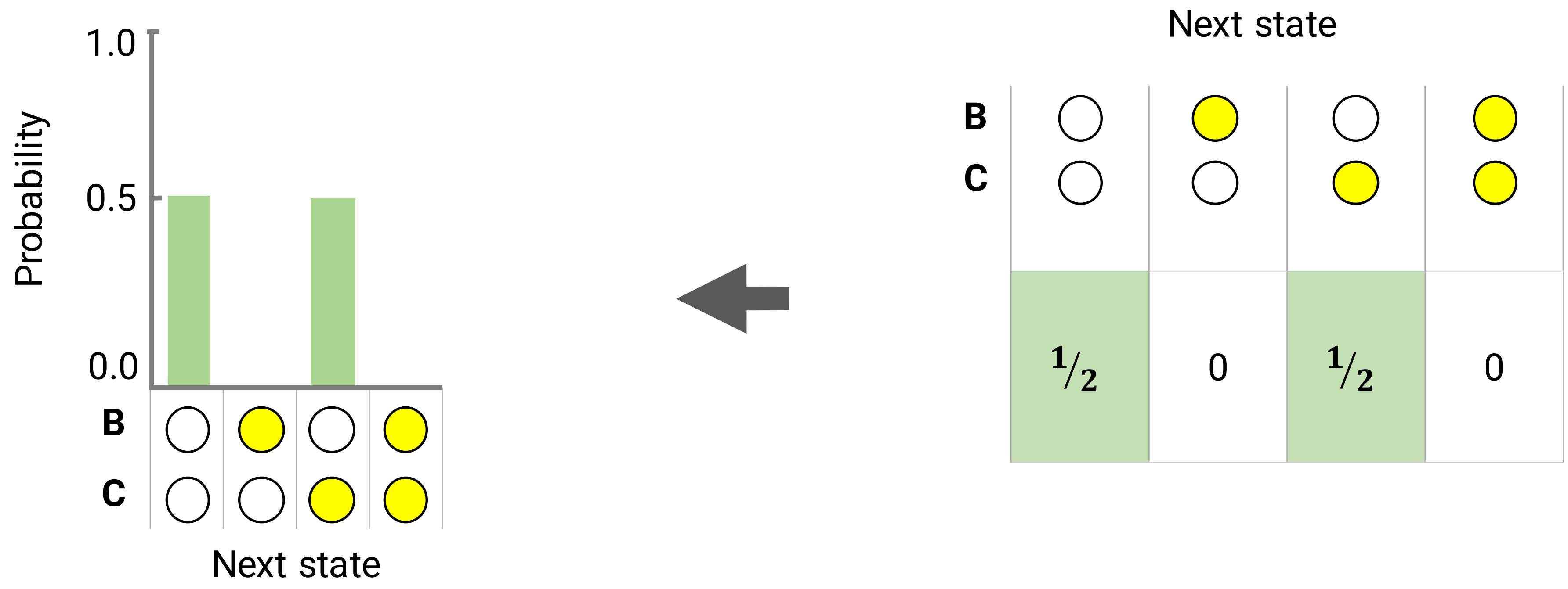

And this is the effect repertoire of mechanism **C** over purview **BC** when the system is in state (1, 0, 0)

Calculating an effect repertoire:

Recap

We've shown how to determine the effect repertoire by:

SYSTEM		TPM
• Introducing **virtual elements** to remove effects due to common input	↔	• Finding the **virtual TPM** via perturbation
• **Ignoring** the elements outside the purview	↔	• **Marginalizing-out** the elements outside the purview
• **Ignoring** the elements outside the mechanism	↔	• **Marginalizing-out** the elements outside the mechanism
• **Fixing** the current state of the mechanism	↔	• **Conditioning** the TPM on the state of the mechanism

Calculating an effect repertoire:
Expanding to the full state-space

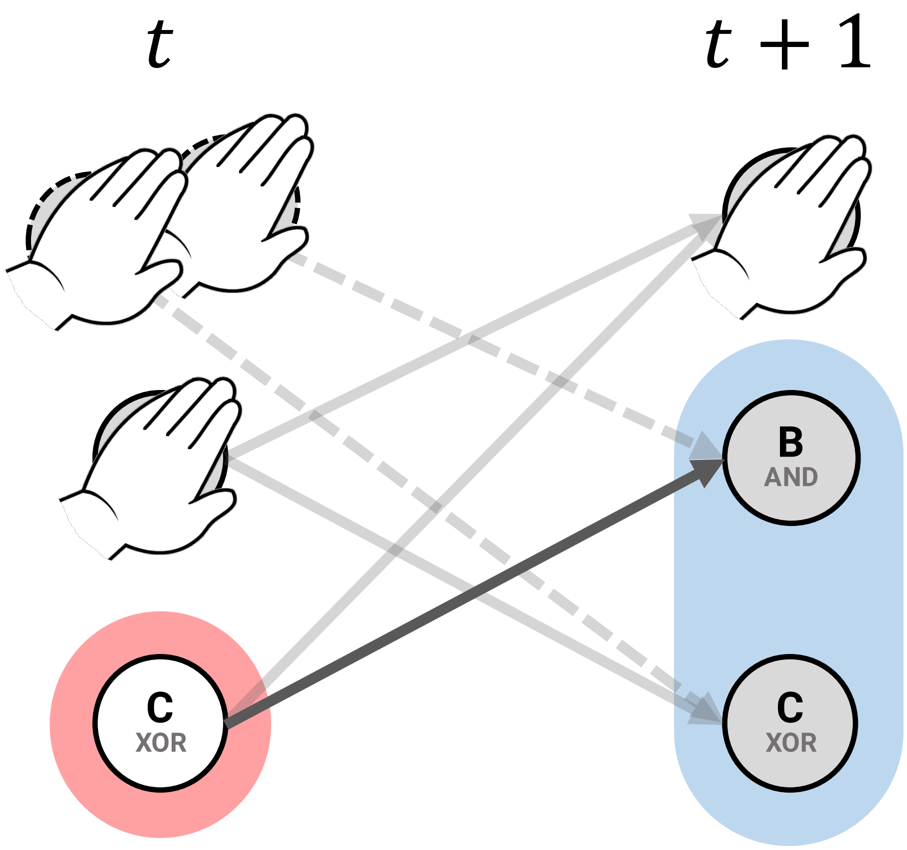

Next state

**B**	○	●	○	●
**C**	○	○	●	●
	$1/2$	0	$1/2$	0

Note that we can expand this repertoire into a distribution over states of the entire system by multiplying it by the **unconstrained distribution** over non-purview elements

Calculating an effect repertoire:
Expanding to the full state-space

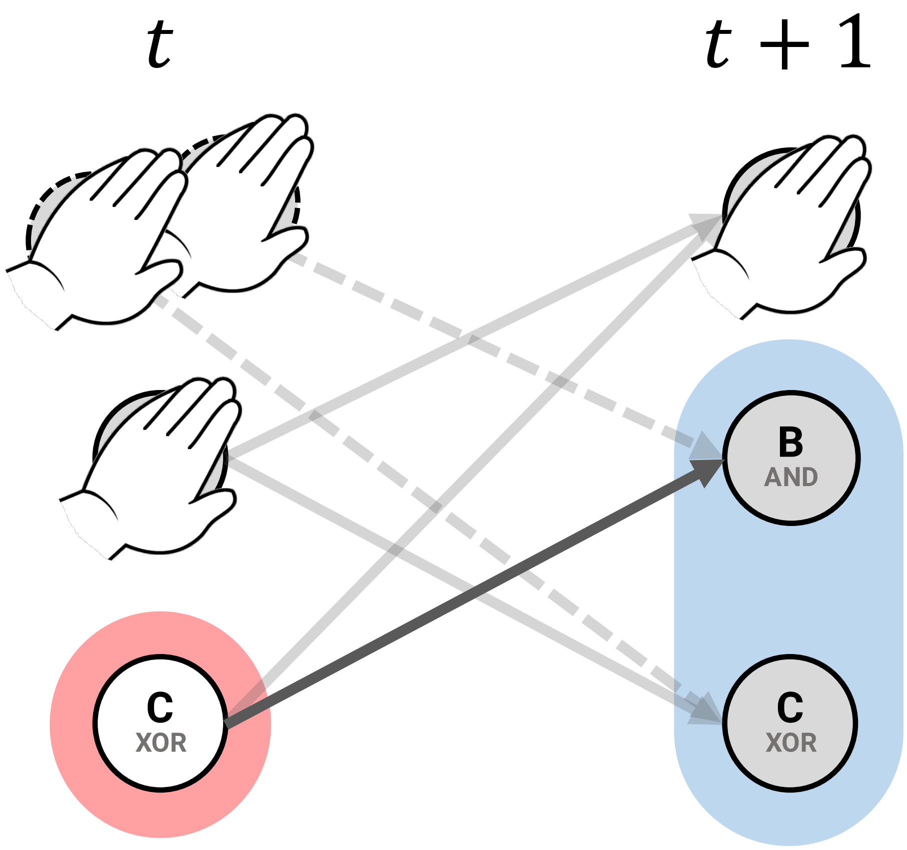

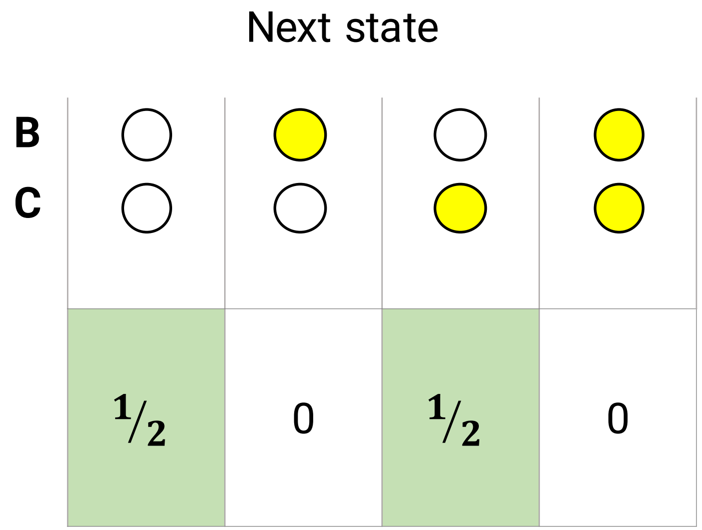

To calculate the **unconstrained distribution**, we use the same method that we just did but **without conditioning on any mechanism**

Calculating an effect repertoire:
Expanding to the full state-space

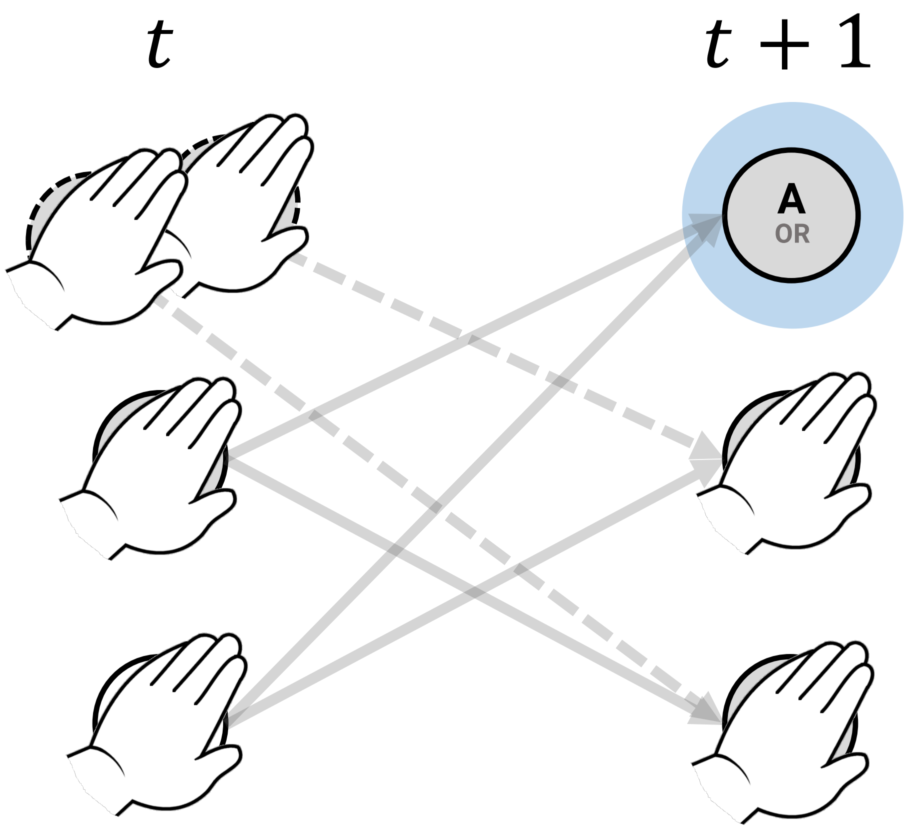

To calculate the **unconstrained distribution**, we use the same method that we just did but **without conditioning on any mechanism**

Calculating an effect repertoire:
Expanding to the full state-space

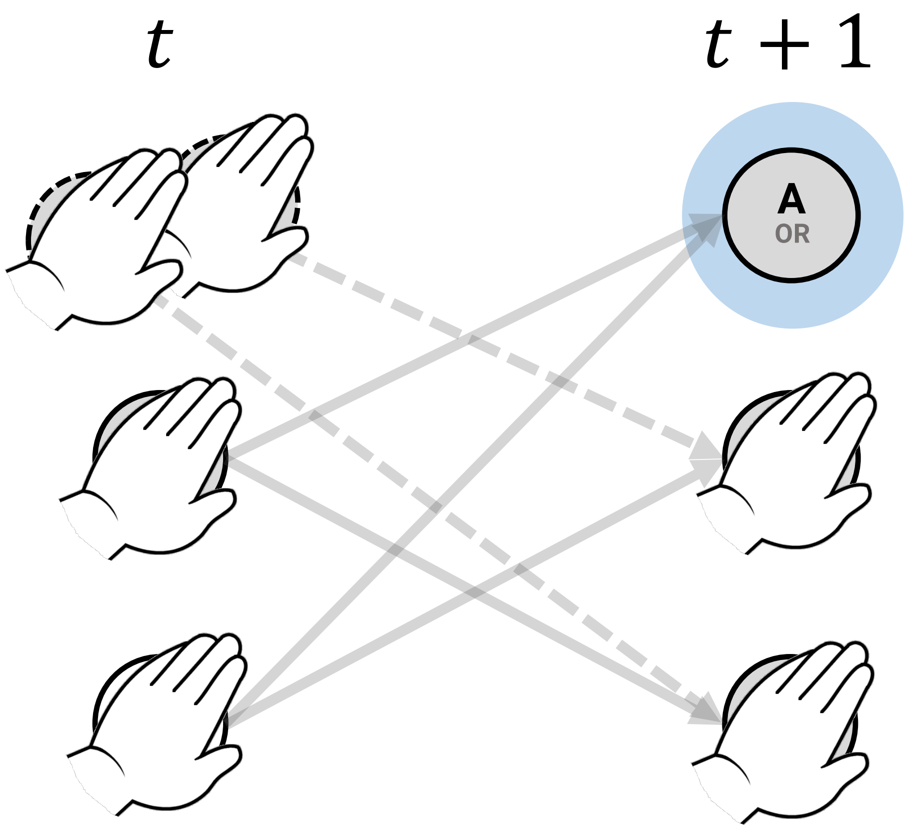

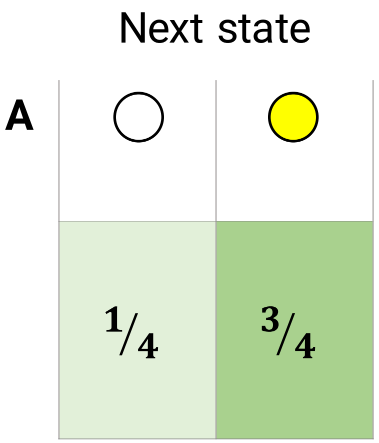

In this example, this is the unconstrained distribution over **A**’s next states
(here this can be obtained immediately by observing that **A** is an OR gate)

Calculating an effect repertoire:
Expanding to the full state-space

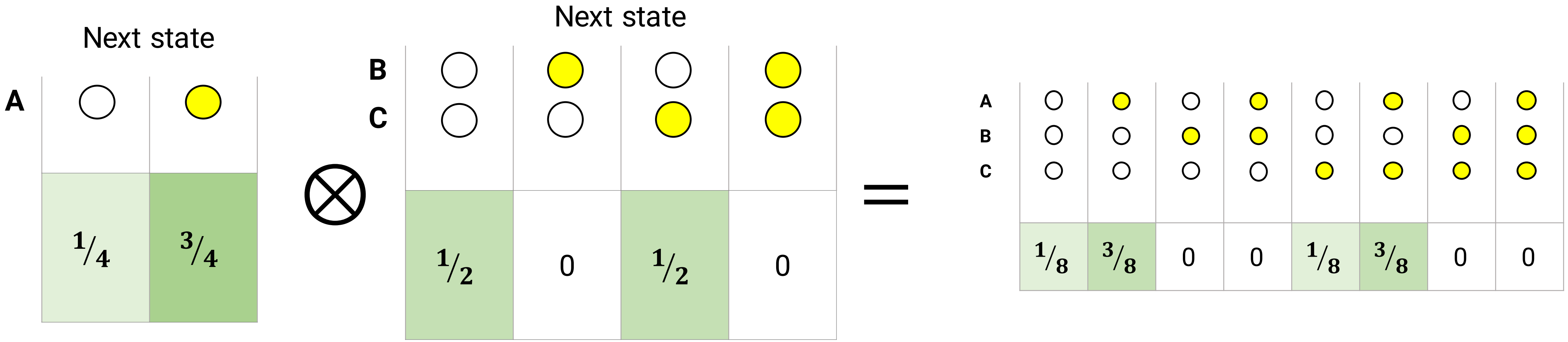

Taking the tensor product yields the final effect repertoire over the whole system

Calculating an effect repertoire:
Expanding to the full state-space

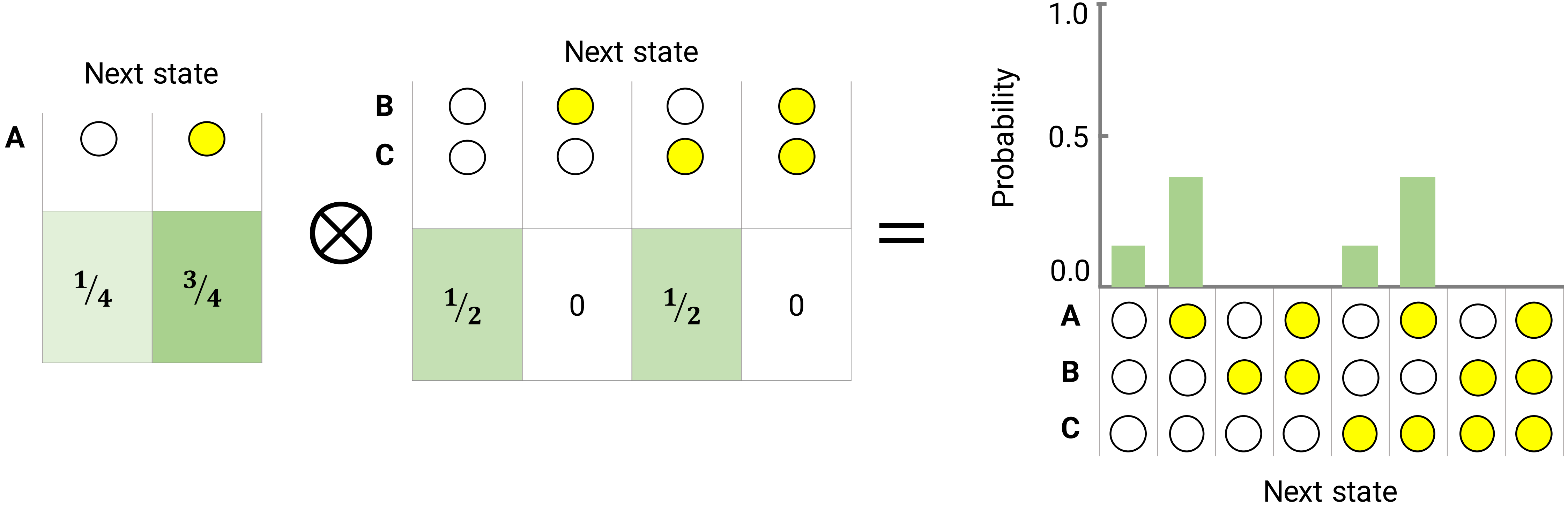

Taking the tensor product yields the final effect repertoire over the whole system

Calculating an effect repertoire:

A more practical method

- In practice, calculations can be made simpler than described so far
- One trick we can use to simplify things stems from the fact that in our model of physical systems, we rule out instantaneous causation
- This is captured by the requirement that elements be conditionally independent
- That is, each element's state at $t+1$ depends only the system's state at t and not on other elements' states at $t+1$

Calculating an effect repertoire:
A more practical method

- Conditional independence implies that if *p* is a distribution over the states of an element **X** and *q* is the distribution over the states of **Y**, then the **joint distribution** of **X** and **Y** is the product *pq*
- So, when we calculate an effect repertoire over some purview, we can simply take the product of all the purview elements' individual effect repertoires
- This holds for the cause repertoires as well, though in that case the repertoires are over the individual mechanism elements
- This way we only ever need to calculate the effect repertoire over single-element purviews—so there can be no common input, and thus there's no need to actually implement virtual elements

Calculating a cause repertoire

- Now we'll discuss the cause repertoire
- The goal is again to obtain a distribution over purview states given the mechanism's current state
- Now, however, the distribution is over previous states of the purview
- The idea remains the same: use perturbation to determine how the mechanism in its current state constrains the purview

Calculating a cause repertoire

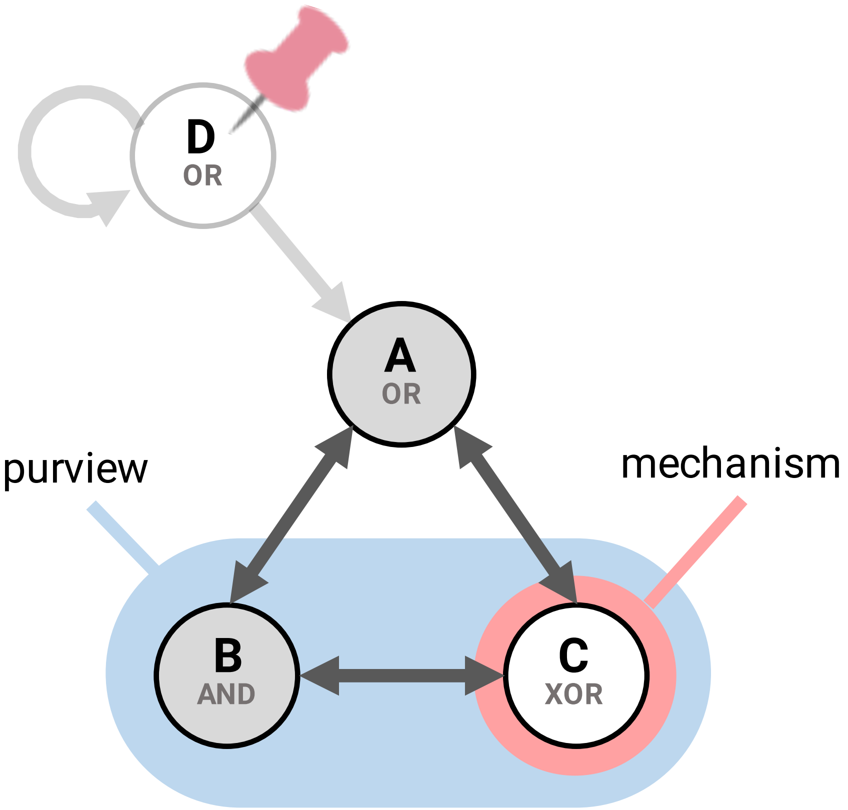

Next state

Current state A	Current state B	Current state C	A ○ B ○ C ○	A ● B ○ C ○	A ○ B ● C ○	A ● B ● C ○	A ○ B ○ C ●	A ● B ○ C ●	A ○ B ● C ●	A ● B ● C ●
○	○	○	1	0	0	0	0	0	0	0
●	○	○	0	0	0	0	1	0	0	0
○	●	○	0	0	0	0	0	1	0	0
●	●	○	0	1	0	0	0	0	0	0
○	○	●	0	1	0	0	0	0	0	0
●	○	●	0	0	0	0	0	0	0	1
○	●	●	0	0	0	0	0	1	0	0
●	●	●	0	0	0	1	0	0	0	0

Now we'll calculate the cause repertoire of **C** over the purview **BC** in our example system

Calculating a cause repertoire

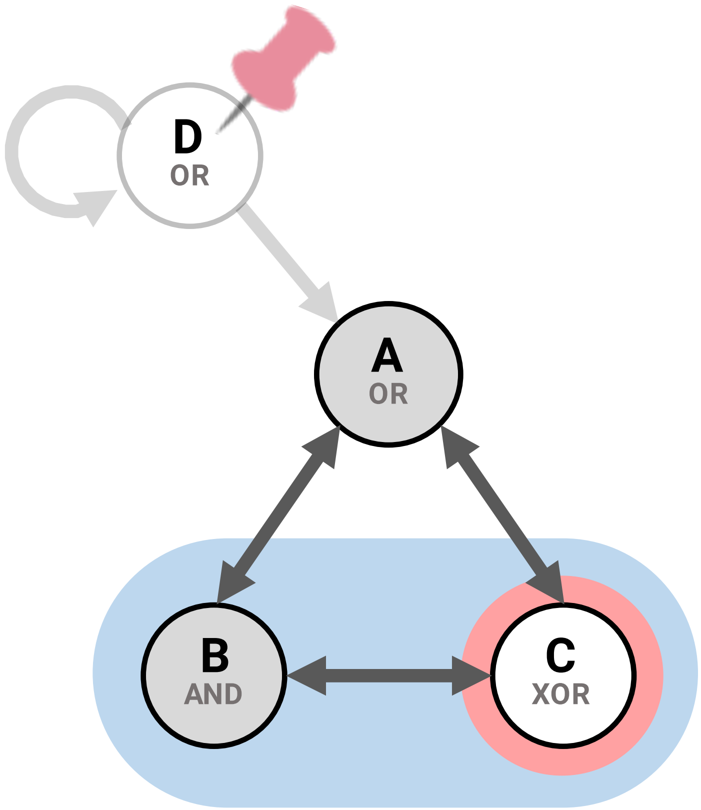

Next state

			A	○	●	○	●	○	●	○	●
			B	○	○	●	●	○	○	●	●
			C	○	○	○	○	●	●	●	●
A	B	C									
○	○	○		1	0	0	0	0	0	0	0
●	○	○		0	0	0	0	1	0	0	0
○	●	○		0	0	0	0	0	1	0	0
●	●	○		0	1	0	0	0	0	0	0
○	○	●		0	1	0	0	0	0	0	0
●	○	●		0	0	0	0	0	0	0	1
○	●	●		0	0	0	0	0	1	0	0
●	●	●		0	0	0	1	0	0	0	0

Current state

We start by interpreting the TPM as giving the transition probabilities from the state at $t-1$ to t

Calculating a cause repertoire

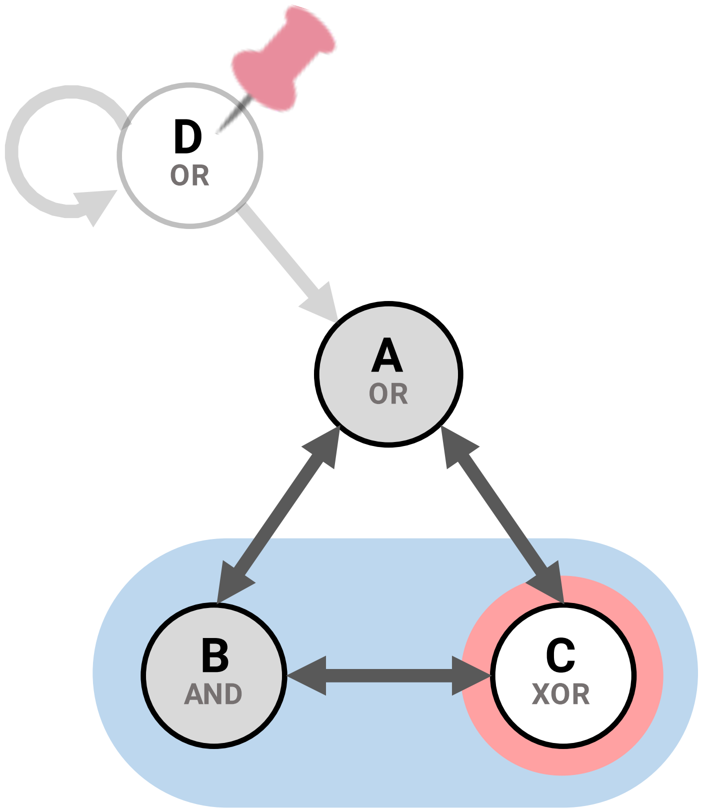

Current state

Previous state

			A	○	●	○	●	○	●	○	●
			B	○	○	●	●	○	○	●	●
			C	○	○	○	○	●	●	●	●
A	B	C									
○	○	○		1	0	0	0	0	0	0	0
●	○	○		0	0	0	0	1	0	0	0
○	●	○		0	0	0	0	0	1	0	0
●	●	○		0	1	0	0	0	0	0	0
○	○	●		0	1	0	0	0	0	0	0
●	○	●		0	0	0	0	0	0	0	1
○	●	●		0	0	0	0	0	1	0	0
●	●	●		0	0	0	1	0	0	0	0

We start by interpreting the TPM as giving the transition probabilities from the state at $t-1$ to t

Calculating a cause repertoire

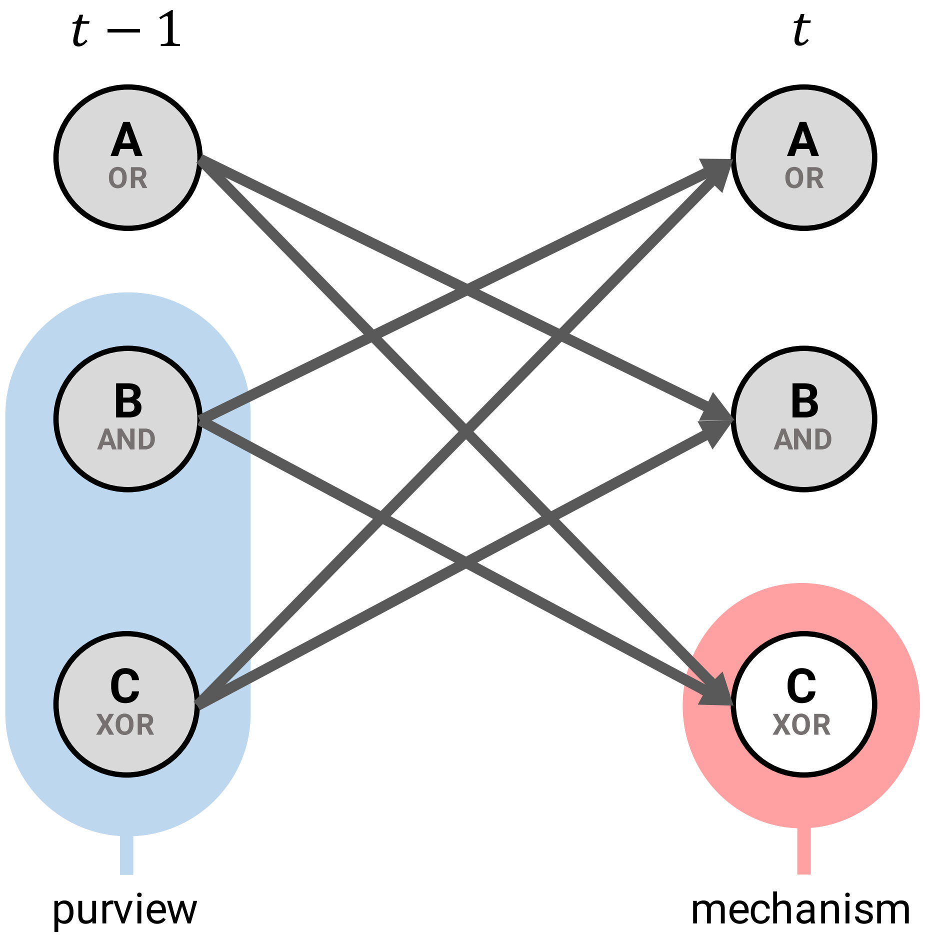

Current state

		A	○	●	○	●	○	●	○	●
		B	○	○	●	●	○	○	●	●
		C	○	○	○	○	●	●	●	●
**A**	**B**	**C**								
○	○	○	1	0	0	0	0	0	0	0
●	○	○	0	0	0	0	1	0	0	0
○	●	○	0	0	0	0	0	1	0	0
●	●	○	0	1	0	0	0	0	0	0
○	○	●	0	1	0	0	0	0	0	0
●	○	●	0	0	0	0	0	0	0	1
○	●	●	0	0	0	0	0	1	0	0
●	●	●	0	0	0	1	0	0	0	0

Previous state

Now we'll unfold the graph in time again

Calculating a cause repertoire:
Marginalizing-out non-purview elements

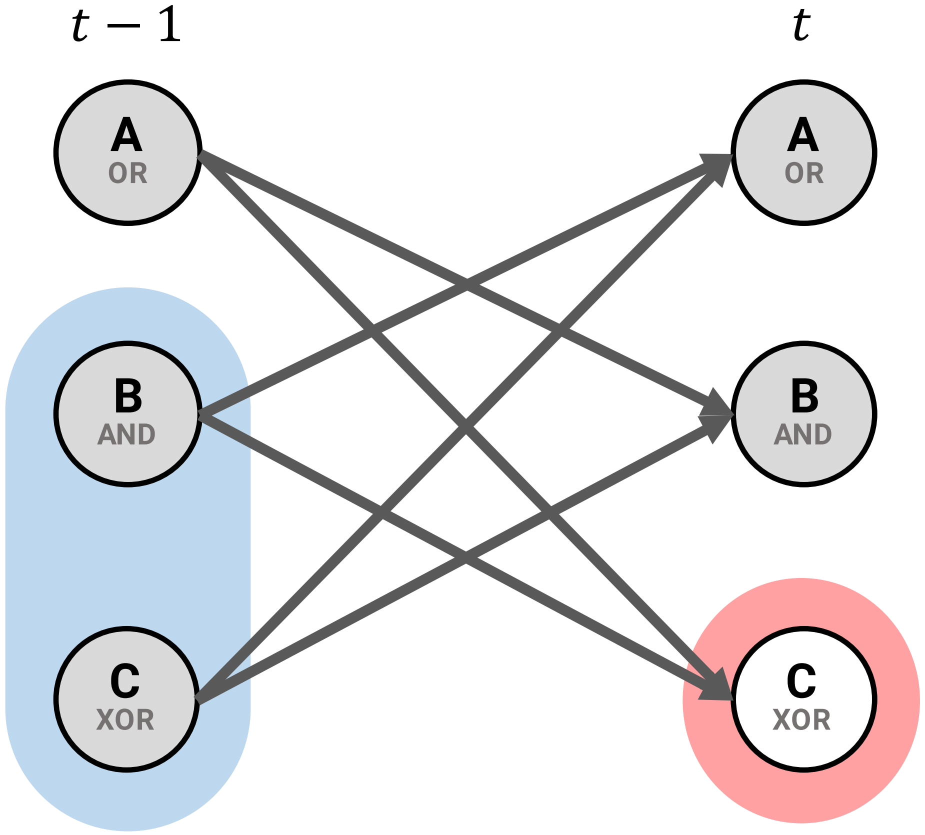

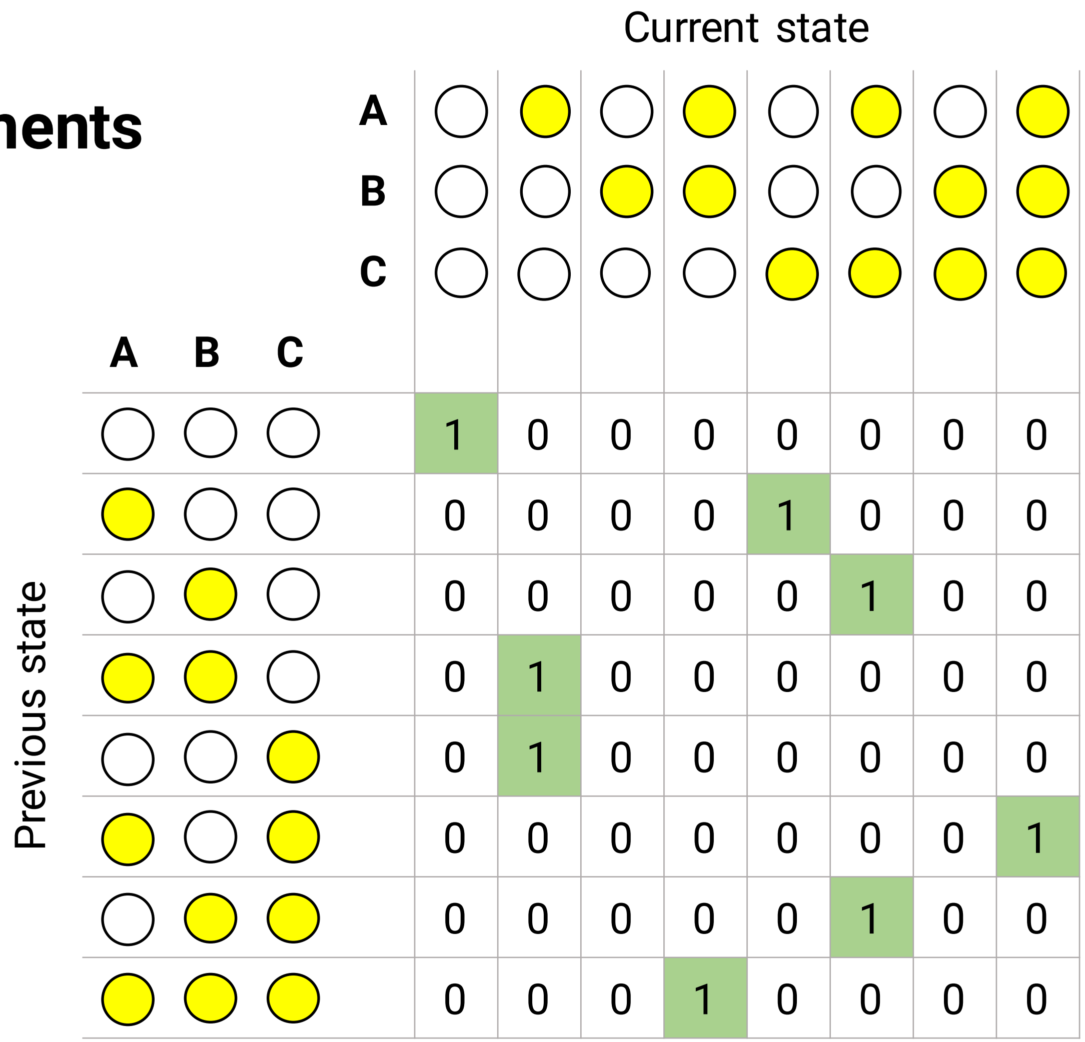

The first step is then to ignore the elements outside the purview (in this case **A**) and marginalize them out of the TPM

Calculating a cause repertoire:

Marginalizing-out non-purview elements

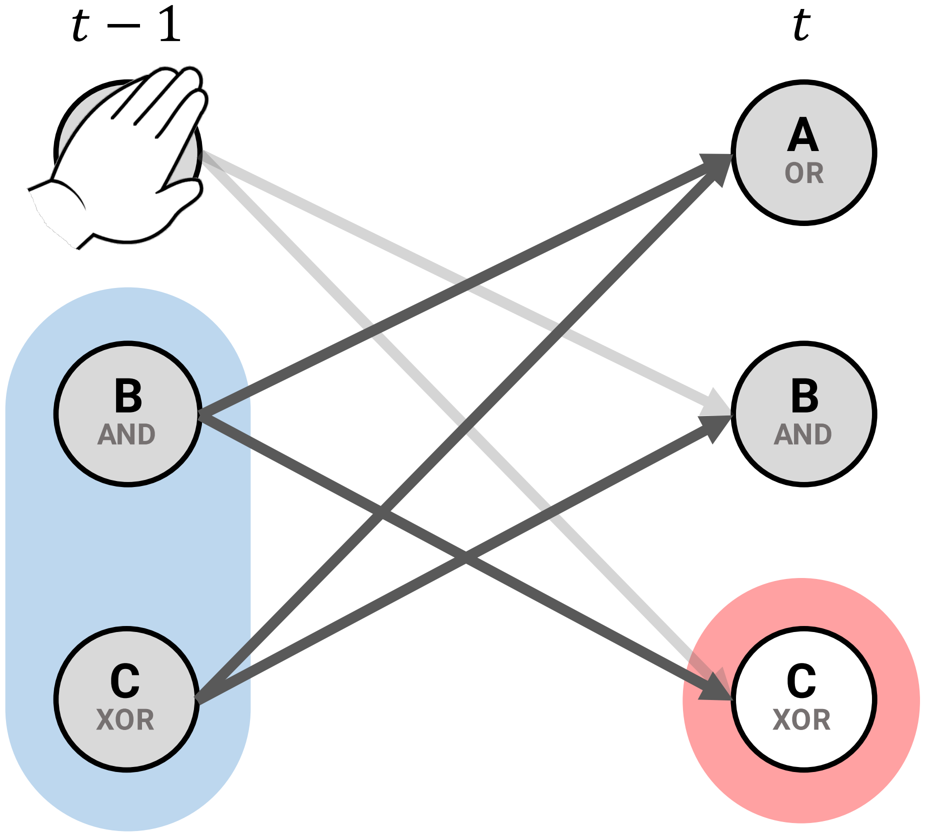

Current state

		A	○	●	○	●	○	●	○	●
		B	○	○	●	●	○	○	●	●
		C	○	○	○	○	●	●	●	●
A	B	C								
○	○	○	1	0	0	0	0	0	0	0
●	○	○	0	0	0	0	1	0	0	0
○	●	○	0	0	0	0	0	1	0	0
●	●	○	0	1	0	0	0	0	0	0
○	○	●	0	1	0	0	0	0	0	0
●	○	●	0	0	0	0	0	0	0	1
○	●	●	0	0	0	0	0	1	0	0
●	●	●	0	0	0	1	0	0	0	0

Previous state

The first step is then to ignore the elements outside the purview (in this case **A**) and marginalize them out of the TPM

Calculating a cause repertoire:

Marginalizing-out non-purview elements

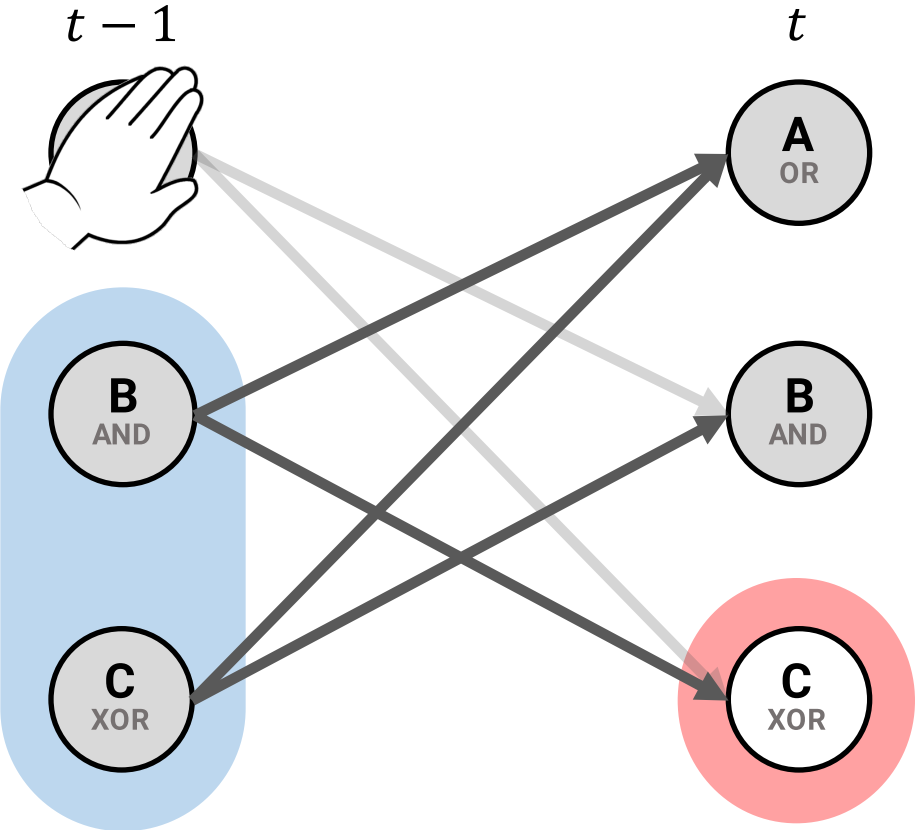

				Current state							
			A	○	●	○	●	○	●	○	●
			B	○	○	●	●	○	○	●	●
			C	○	○	○	○	●	●	●	●
**A**	**B**	**C**	Previous state								
○	○	○		1	0	0	0	0	0	0	0
●	○	○		0	0	0	0	1	0	0	0
○	●	○		0	0	0	0	0	1	0	0
●	●	○		0	1	0	0	0	0	0	0
○	○	●		0	1	0	0	0	0	0	0
●	○	●		0	0	0	0	0	0	0	1
○	●	●		0	0	0	0	0	1	0	0
●	●	●		0	0	0	1	0	0	0	0

Note that since the purview is now at $t-1$, the roles of columns and rows in the TPM have switched

Calculating a cause repertoire:

Marginalizing-out non-purview elements

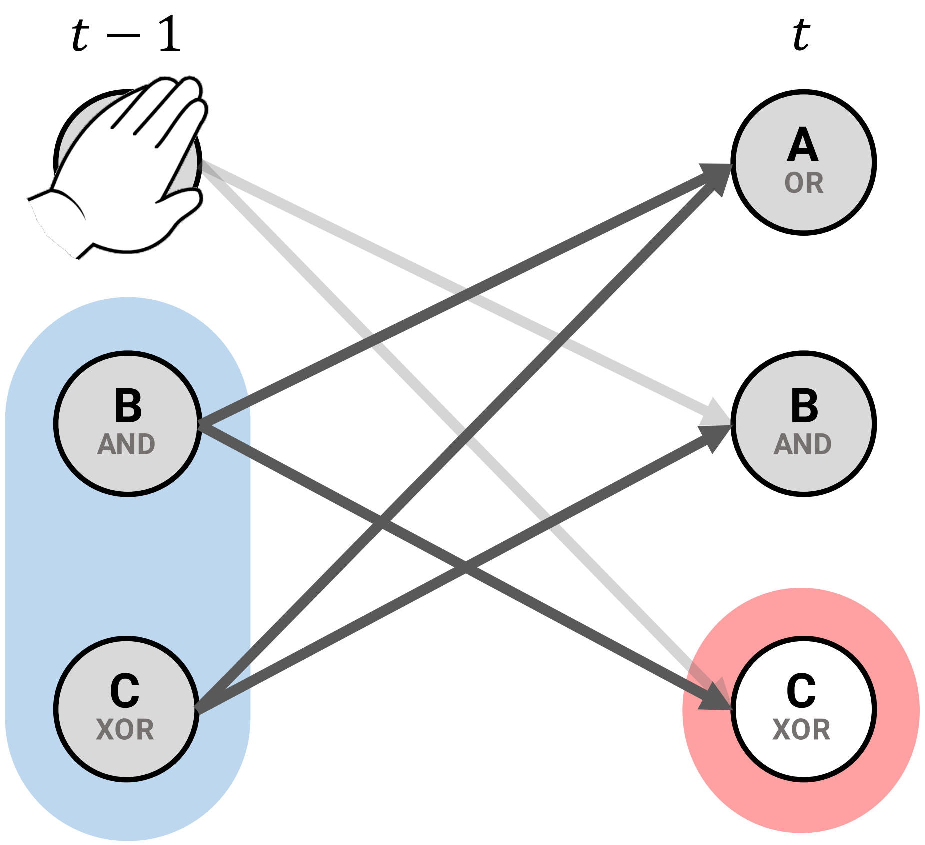

Current state / Previous state

A	B	C								
		A	○	●	○	●	○	●	○	●
		B	○	○	●	●	○	○	●	●
		C	○	○	○	○	●	●	●	●
○	○	○	1	0	0	0	0	0	0	0
●	○	○	0	0	0	0	1	0	0	0
○	●	○	0	0	0	0	0	1	0	0
●	●	○	0	1	0	0	0	0	0	0
○	○	●	0	1	0	0	0	0	0	0
●	○	●	0	0	0	0	0	0	0	1
○	●	●	0	0	0	0	0	1	0	0
●	●	●	0	0	0	1	0	0	0	0

We now sum and renormalize pairs of **rows** corresponding to states at $t-1$ that differ only by **A**'s state

Calculating a cause repertoire:

Marginalizing-out non-purview elements

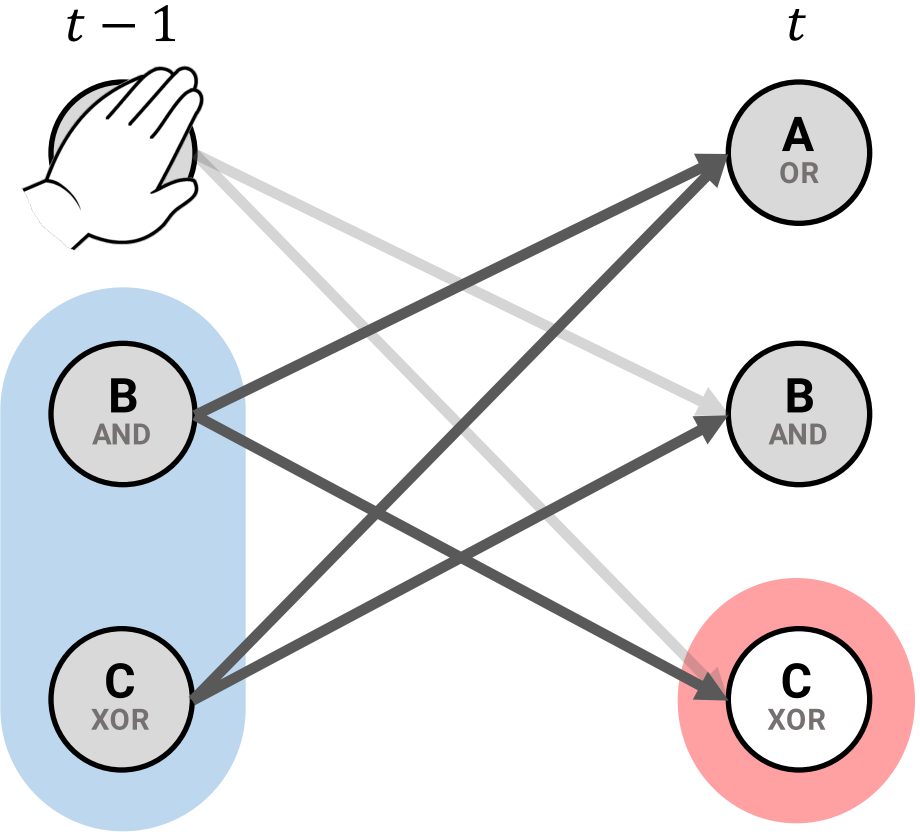

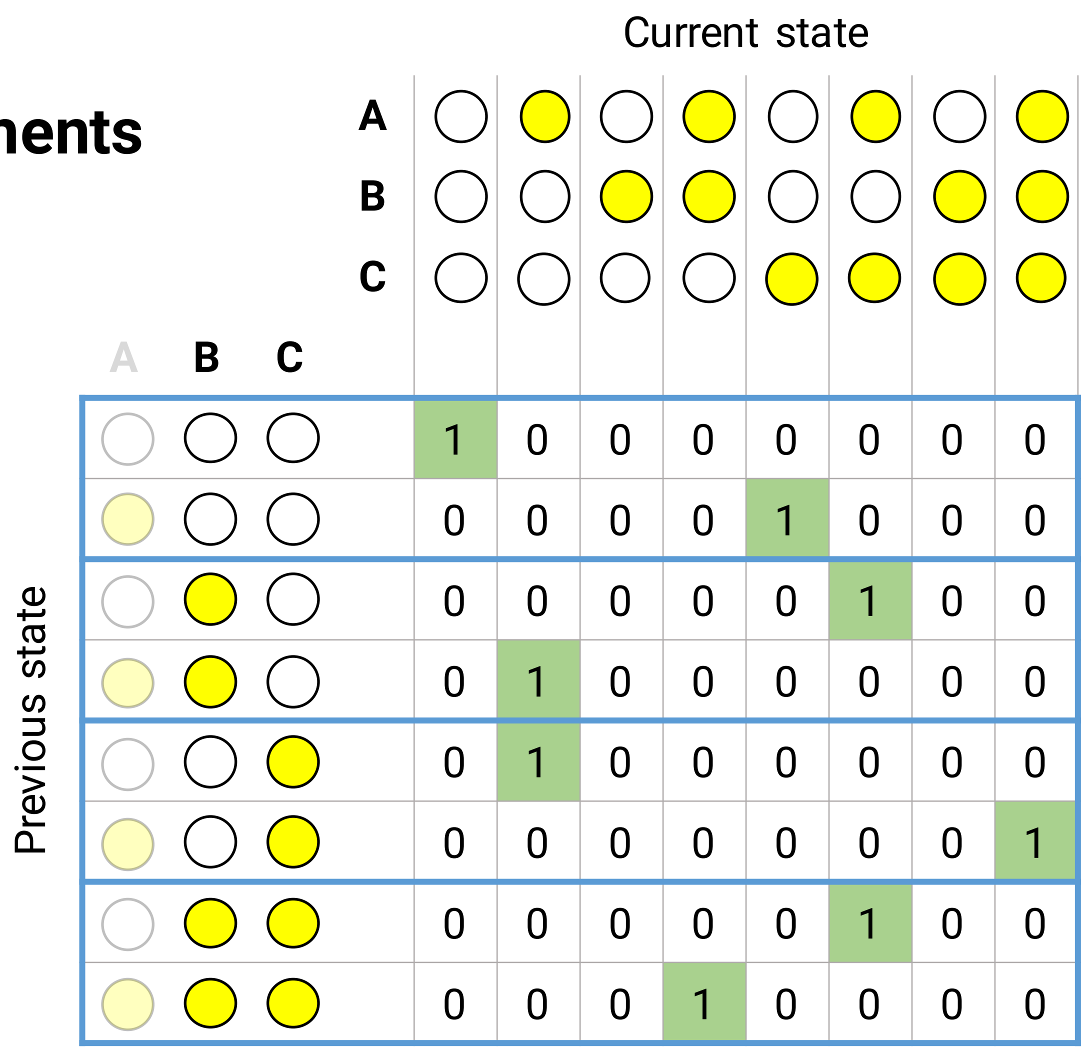

1	0	0	0	0	0	0	0
0	0	0	0	1	0	0	0
0	0	0	0	0	1	0	0
0	1	0	0	0	0	0	0
0	1	0	0	0	0	0	0
0	0	0	0	0	0	0	1
0	0	0	0	0	1	0	0
0	0	0	1	0	0	0	0

We now sum and renormalize pairs of **rows** corresponding to states at $t-1$ that differ only by **A**’s state

Calculating a cause repertoire:
Marginalizing-out non-purview elements

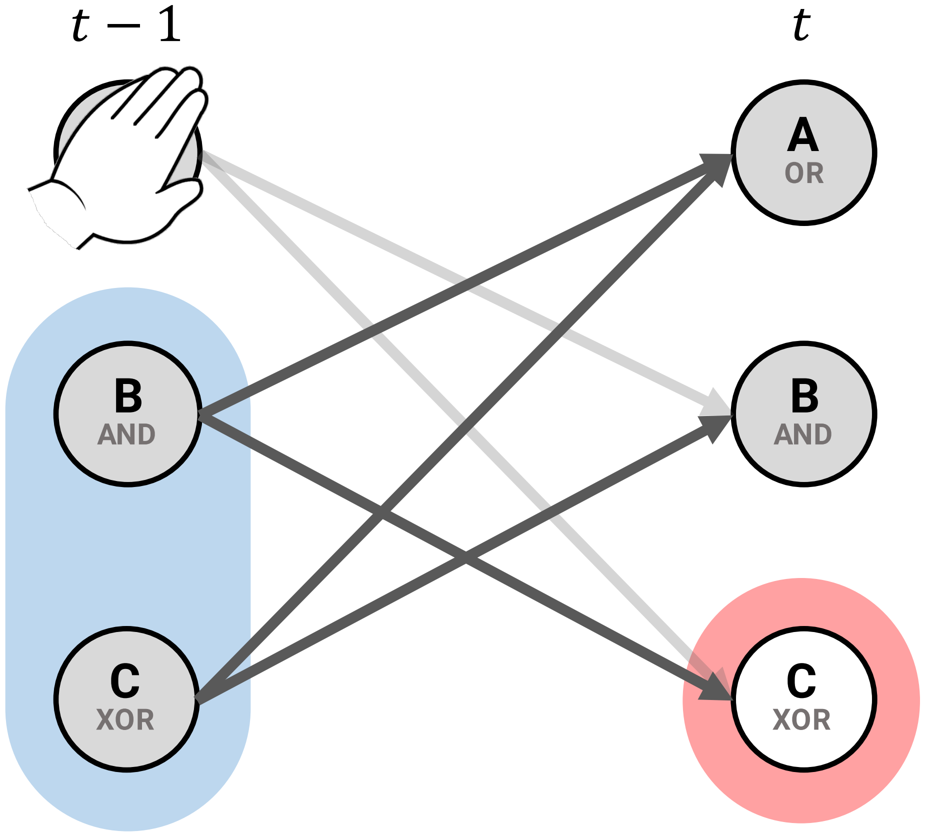

Current state

B	C	A: ○<br>B: ○<br>C: ○	A: ●<br>B: ○<br>C: ○	A: ○<br>B: ●<br>C: ○	A: ●<br>B: ●<br>C: ○	A: ○<br>B: ○<br>C: ●	A: ●<br>B: ○<br>C: ●	A: ○<br>B: ●<br>C: ●	A: ●<br>B: ●<br>C: ●
○	○	1	0	0	0	0	0	0	0
○	○	0	0	0	0	1	0	0	0
●	○	0	0	0	0	0	1	0	0
●	○	0	1	0	0	0	0	0	0
○	●	0	1	0	0	0	0	0	0
○	●	0	0	0	0	0	0	0	1
●	●	0	0	0	0	0	1	0	0
●	●	0	0	0	1	0	0	0	0

Previous state

We now sum and renormalize pairs of **rows** corresponding to states at $t-1$ that differ only by **A**'s state

Calculating a cause repertoire:

Marginalizing-out non-purview elements

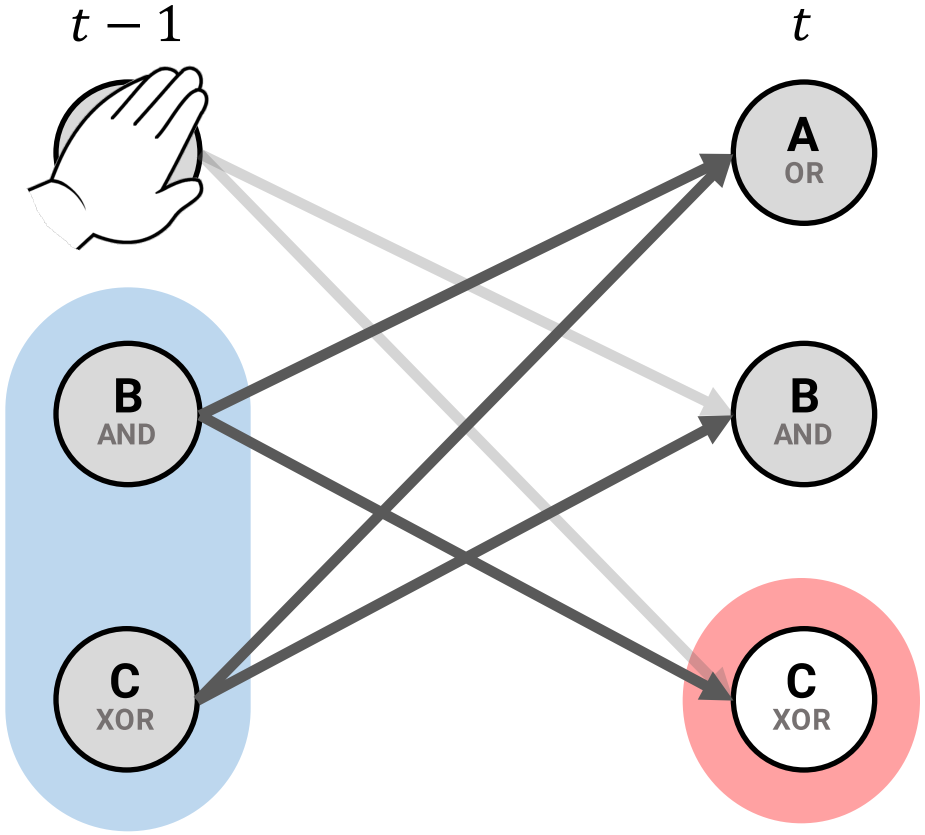

Current state

		A	○	●	○	●	○	●	○	●
		B	○	○	●	●	○	○	●	●
		C	○	○	○	○	●	●	●	●
**B**	**C**									
○	○		1	0	0	0	1	0	0	0
●	○		0	1	0	0	0	1	0	0
○	●		0	1	0	0	0	0	0	1
●	●		0	0	0	1	0	1	0	0

Previous state

We now sum and renormalize pairs of **rows** corresponding to states at $t-1$ that differ only by **A**'s state

Calculating a cause repertoire:

Marginalizing-out non-purview elements

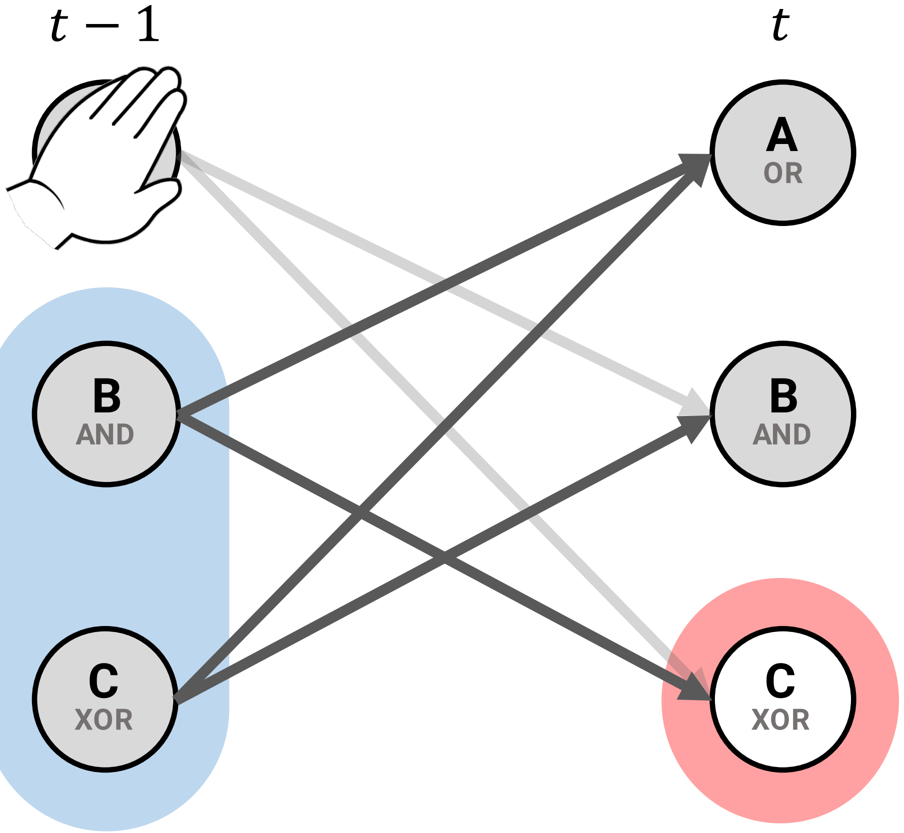

		Current state							
	A	○	●	○	●	○	●	○	●
	B	○	○	●	●	○	○	●	●
	C	○	○	○	○	●	●	●	●
Previous state: B	C								
○	○	$^1/_2$	0	0	0	$^1/_2$	0	0	0
●	○	0	$^1/_2$	0	0	0	$^1/_2$	0	0
○	●	0	$^1/_2$	0	0	0	0	0	$^1/_2$
●	●	0	0	0	$^1/_2$	0	$^1/_2$	0	0

We now sum and renormalize pairs of **rows** corresponding to states at $t-1$ that differ only by **A**'s state

Calculating a cause repertoire:
Marginalizing-out non-purview elements

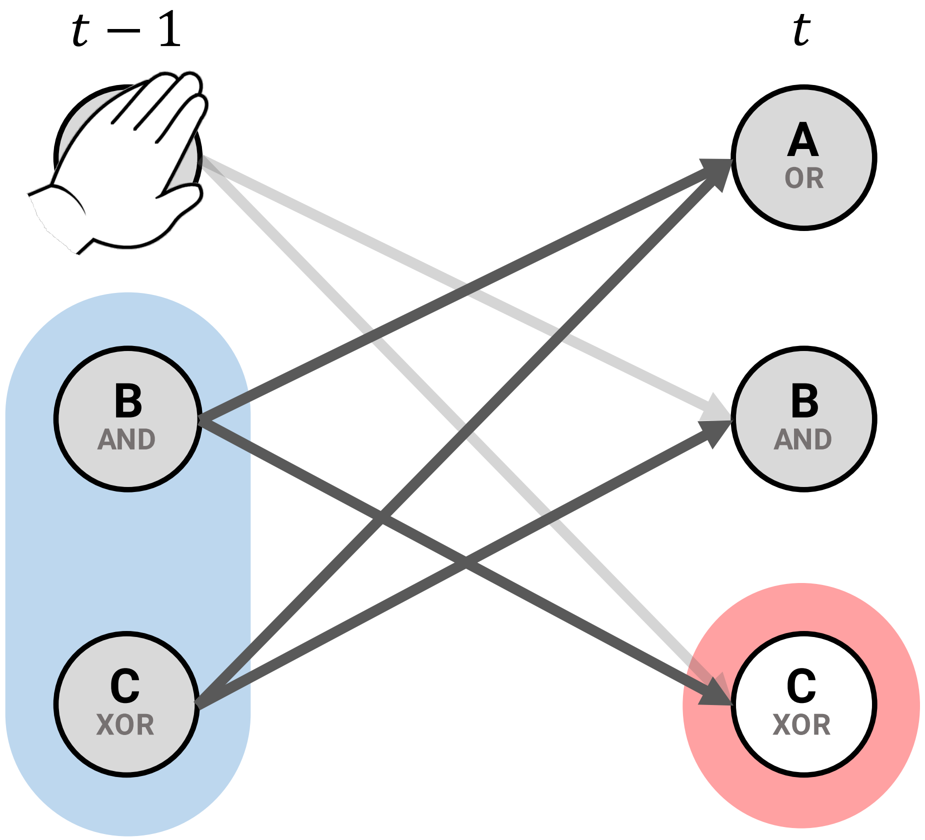

		Current state							
	**A**	○	●	○	●	○	●	○	●
	**B**	○	○	●	●	○	○	●	●
	**C**	○	○	○	○	●	●	●	●
**Previous state: B**	**C**								
○	○	$^1/_2$	0	0	0	$^1/_2$	0	0	0
●	○	0	$^1/_2$	0	0	0	$^1/_2$	0	0
○	●	0	$^1/_2$	0	0	0	0	0	$^1/_2$
●	●	0	0	0	$^1/_2$	0	$^1/_2$	0	0

We now sum and renormalize pairs of **rows** corresponding to states at $t-1$ that differ only by **A**'s state

Calculating a cause repertoire:

Marginalizing-out non-mechanism elements

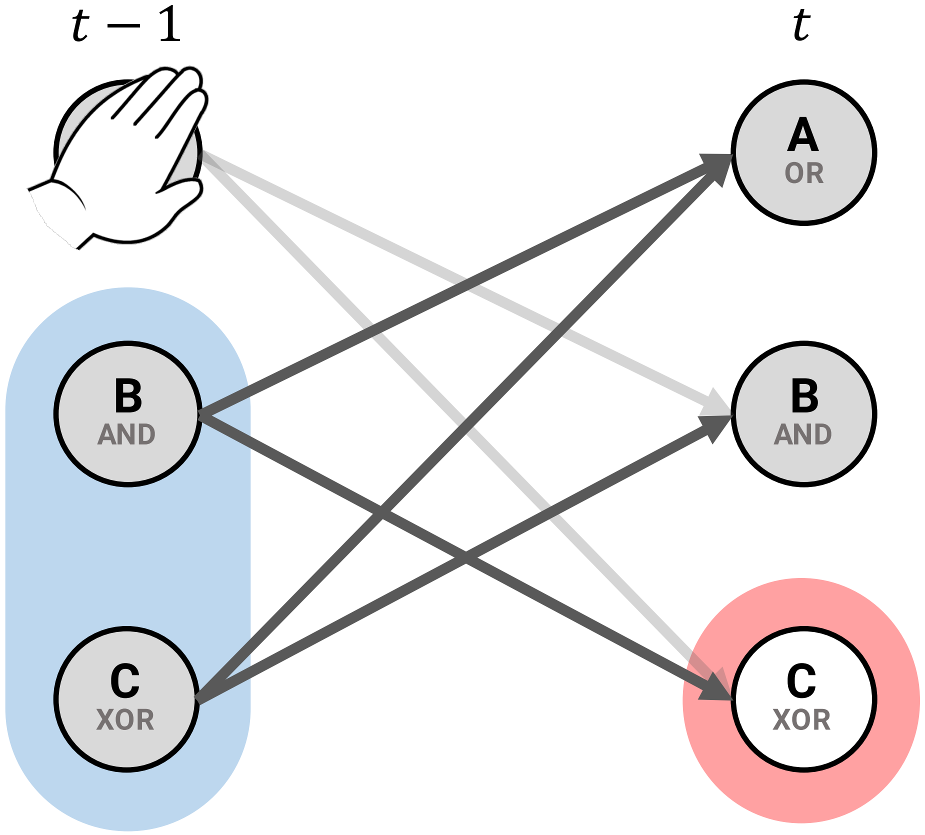

			Current state							
		A	○	●	○	●	○	●	○	●
		B	○	○	●	●	○	○	●	●
		C	○	○	○	○	●	●	●	●
Previous state	B	C								
	○	○	$^1/_2$	0	0	0	$^1/_2$	0	0	0
	●	○	0	$^1/_2$	0	0	0	$^1/_2$	0	0
	○	●	0	$^1/_2$	0	0	0	0	0	$^1/_2$
	●	●	0	0	0	$^1/_2$	0	$^1/_2$	0	0

Now we marginalize over the current states of elements outside the mechanism (**A** and **B**)

Calculating a cause repertoire:

Marginalizing-out non-mechanism elements

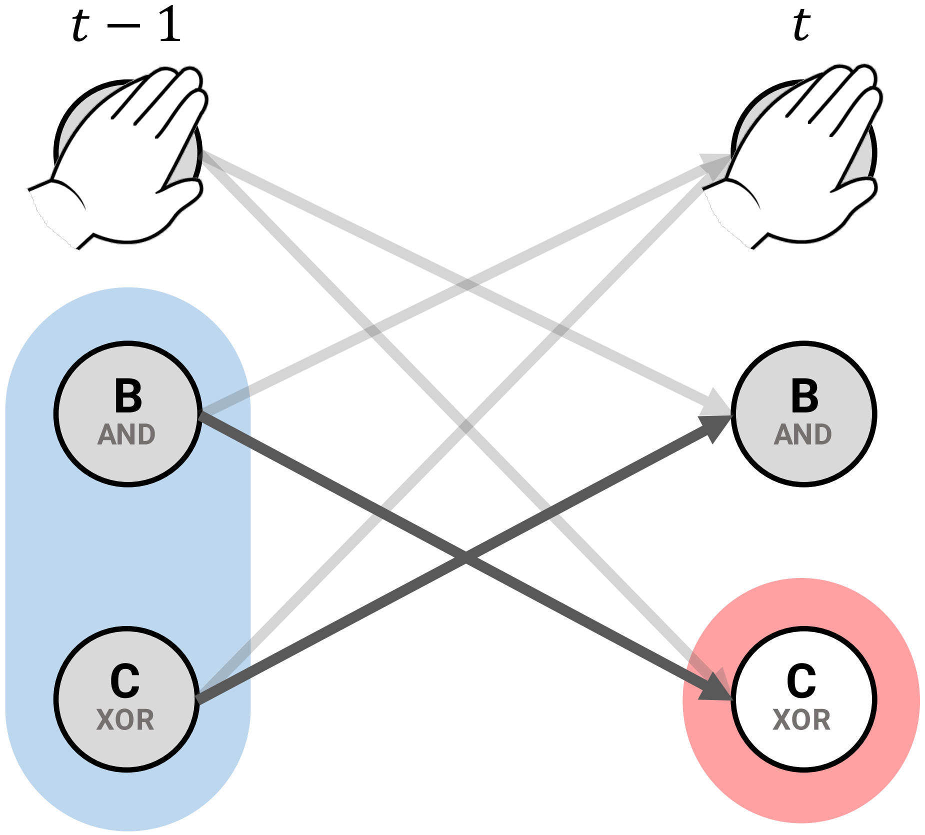

Current state

B	C	Previous state							
		A	○	●	○	●	○	●	○	●
		B	○	○	●	●	○	○	●	●
		C	○	○	○	○	●	●	●	●
○	○		$^1/_2$	0	0	0	$^1/_2$	0	0	0
●	○		0	$^1/_2$	0	0	0	$^1/_2$	0	0
○	●		0	$^1/_2$	0	0	0	0	0	$^1/_2$
●	●		0	0	0	$^1/_2$	0	$^1/_2$	0	0

Now we marginalize over the current states of elements outside the mechanism (**A** and **B**)

Calculating a cause repertoire:

Marginalizing-out non-mechanism elements

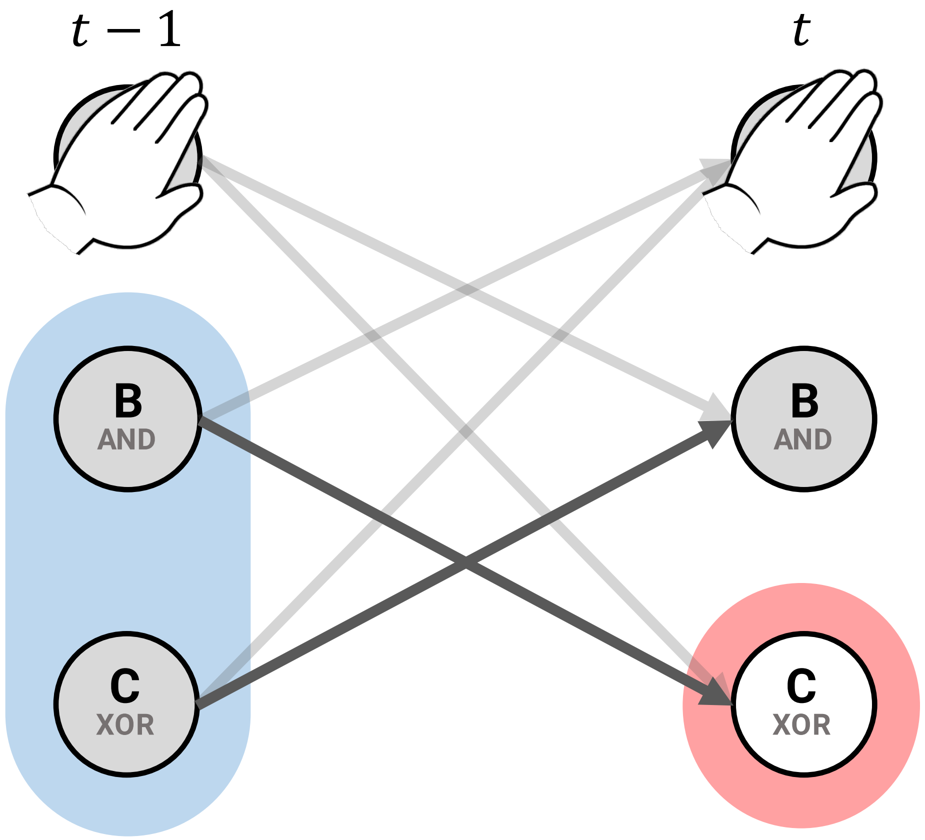

Current state

		A	○	●	○	●	○	●	○	●
		B	○	○	●	●	○	○	●	●
		C	○	○	○	○	●	●	●	●
Previous state B	C									
○	○		$^1/_2$	0	0	0	$^1/_2$	0	0	0
●	○		0	$^1/_2$	0	0	0	$^1/_2$	0	0
○	●		0	$^1/_2$	0	0	0	0	0	$^1/_2$
●	●		0	0	0	$^1/_2$	0	$^1/_2$	0	0

Now we marginalize over the current states of elements outside the mechanism (**A** and **B**)

Calculating a cause repertoire:

Marginalizing-out non-mechanism elements

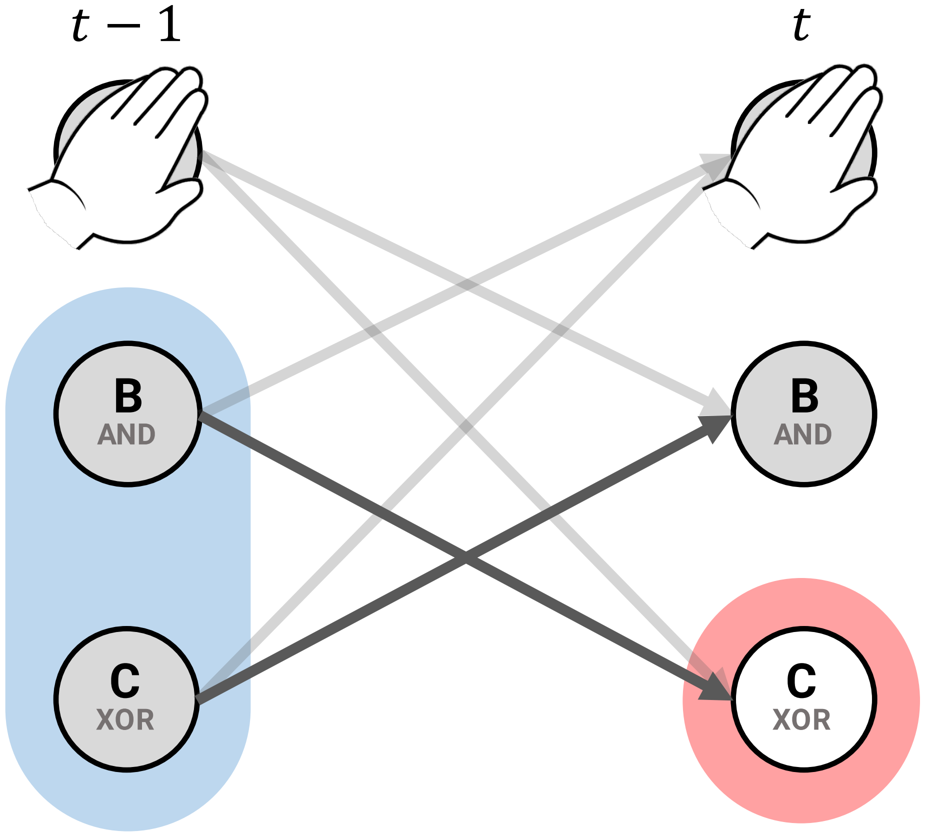

Current state

Previous state (B C)	B ○ C ○	B ○ C ○	B ● C ○	B ● C ○	B ○ C ●	B ○ C ●	B ● C ●	B ● C ●
○ ○	$1/2$	0	0	0	$1/2$	0	0	0
● ○	0	$1/2$	0	0	0	$1/2$	0	0
○ ●	0	$1/2$	0	0	0	0	0	$1/2$
● ●	0	0	0	$1/2$	0	$1/2$	0	0

Now we marginalize over the current states of elements outside the mechanism (**A** and **B**)

Calculating a cause repertoire:
Marginalizing-out non-mechanism elements

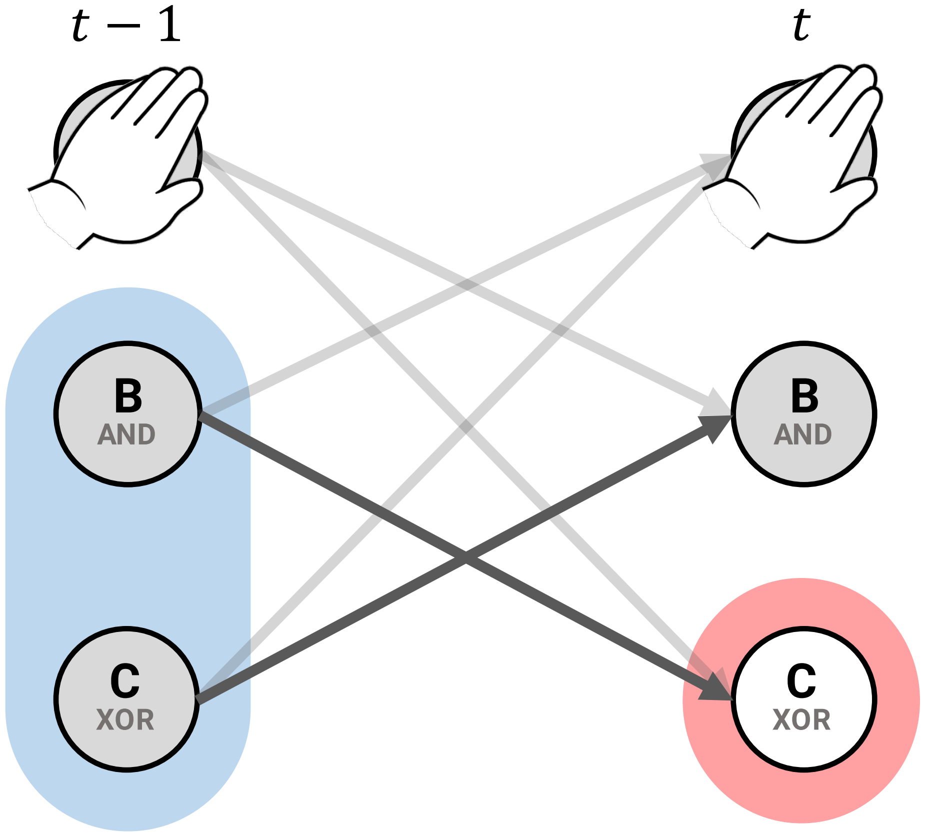

Current state

Previous state B	Previous state C	B ○ C ○	B ● C ○	B ○ C ●	B ● C ●
○	○	$1/2$	0	$1/2$	0
●	○	$1/2$	0	$1/2$	0
○	●	$1/2$	0	0	$1/2$
●	●	0	$1/2$	$1/2$	0

Now we marginalize over the current states of elements outside the mechanism (**A** and **B**)

Calculating a cause repertoire:

Marginalizing-out non-mechanism elements

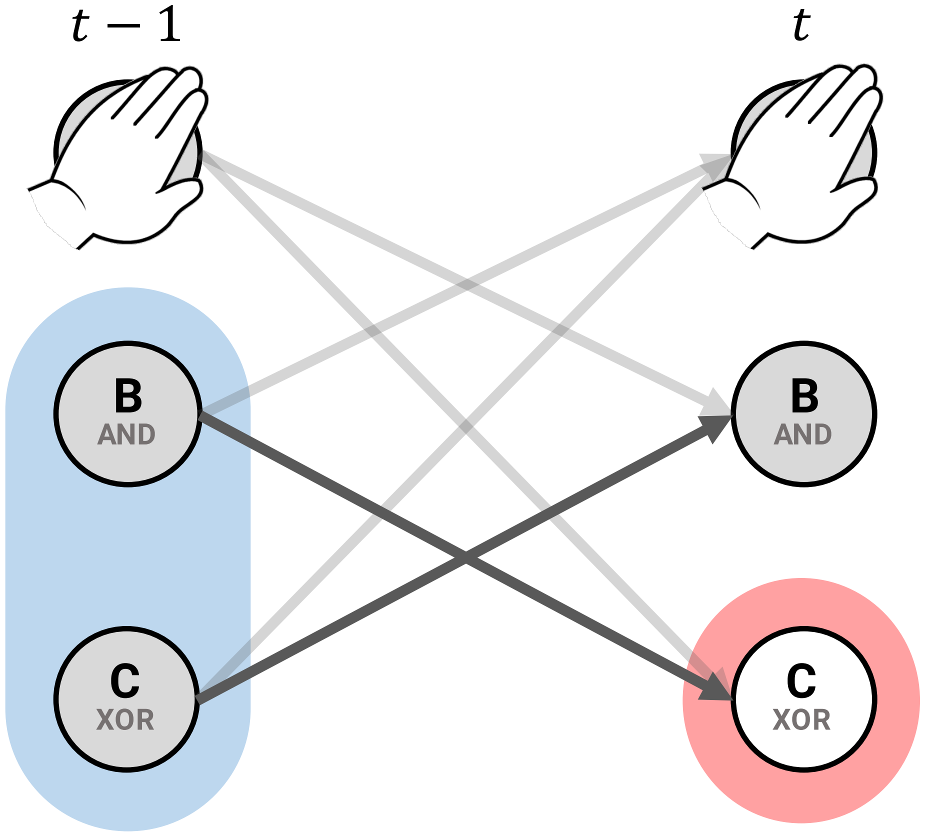

Current state

		B: ○	B: ●	B: ○	B: ●
**B**	**C**	C: ○	C: ○	C: ●	C: ●
○	○	$^1/_2$	0	$^1/_2$	0
●	○	$^1/_2$	0	$^1/_2$	0
○	●	$^1/_2$	0	0	$^1/_2$
●	●	0	$^1/_2$	$^1/_2$	0

Previous state

Now we marginalize over the current states of elements outside the mechanism (**A** and **B**)

Calculating a cause repertoire:

Marginalizing-out non-mechanism elements

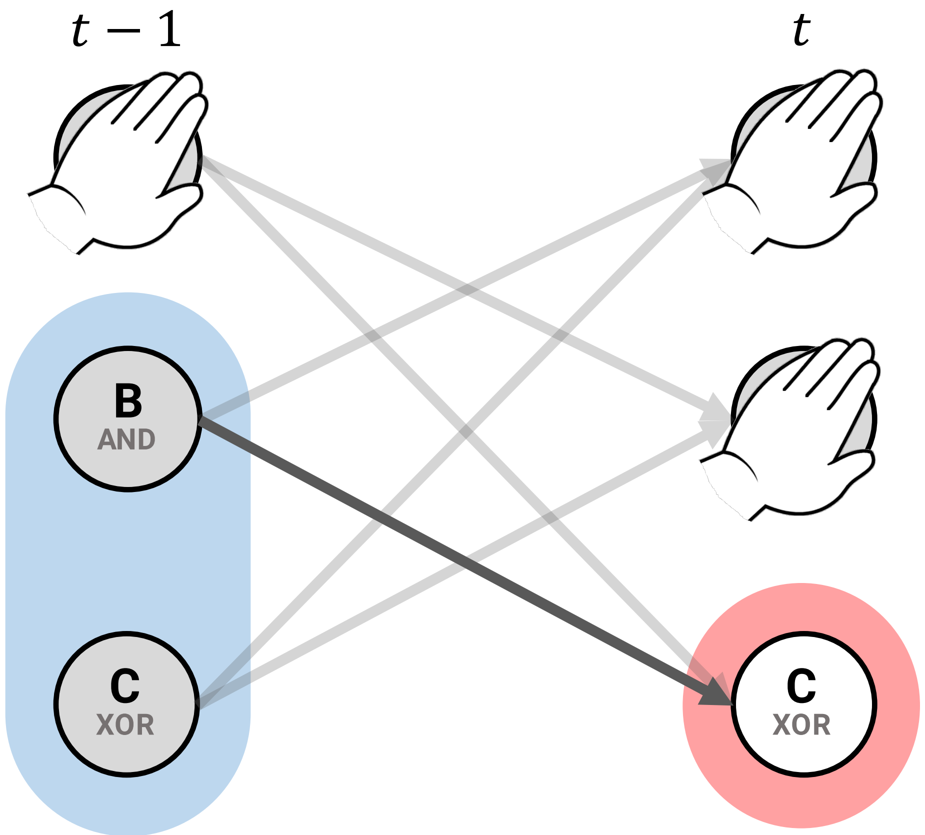

Previous state B	Previous state C	Current state B: ○, C: ○	Current state B: ●, C: ○	Current state B: ○, C: ●	Current state B: ●, C: ●
○	○	$^1/_2$	0	$^1/_2$	0
●	○	$^1/_2$	0	$^1/_2$	0
○	●	$^1/_2$	0	0	$^1/_2$
●	●	0	$^1/_2$	$^1/_2$	0

Now we marginalize over the current states of elements outside the mechanism (**A** and **B**)

Calculating a cause repertoire:

Marginalizing-out non-mechanism elements

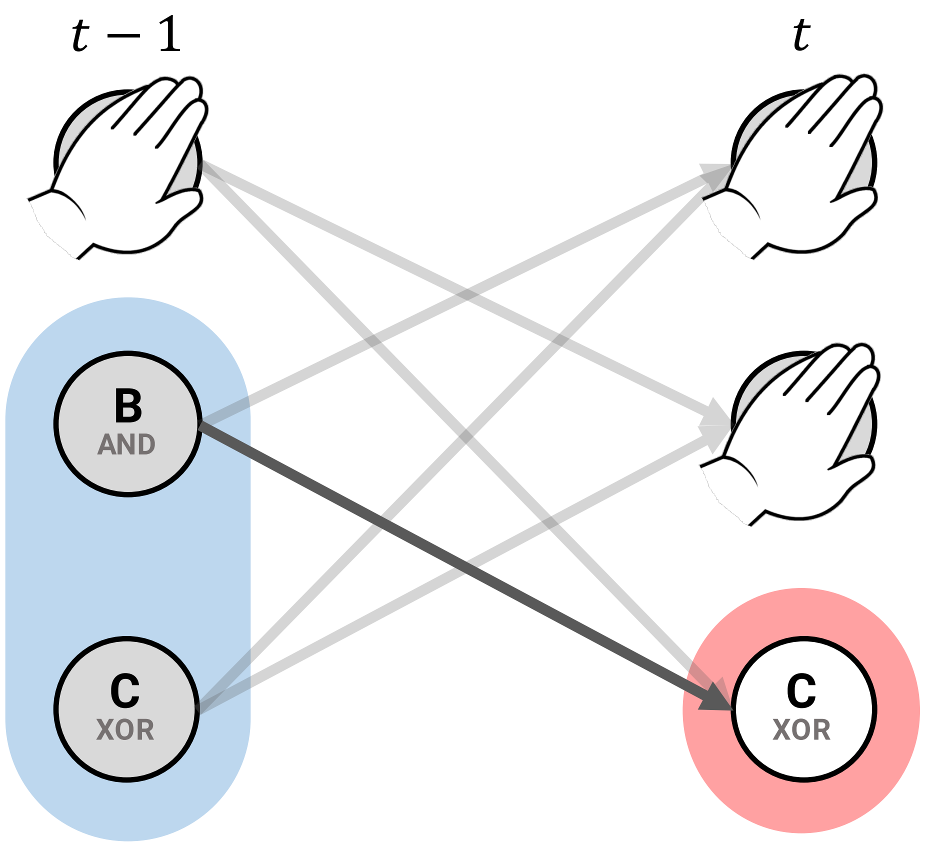

Current state

		B	○	●	○	●
		**C**	○	○	●	●
**B**	**C**					
○	○		$^1/_2$	0	$^1/_2$	0
●	○		$^1/_2$	0	$^1/_2$	0
○	●		$^1/_2$	0	0	$^1/_2$
●	●		0	$^1/_2$	$^1/_2$	0

Previous state

Now we marginalize over the current states of elements outside the mechanism (**A** and **B**)

Calculating a cause repertoire:

Marginalizing-out non-mechanism elements

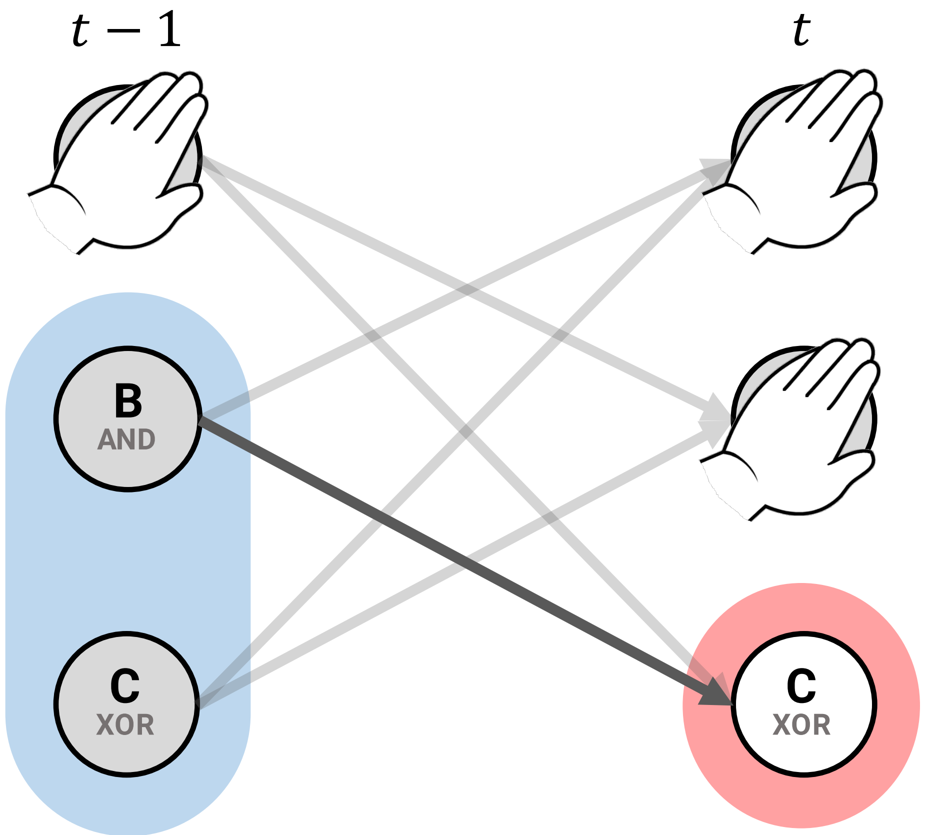

		Current state			
	**C**	○	○	●	●
**B**	**C**				
○	○	$^1/_2$	0	$^1/_2$	0
●	○	$^1/_2$	0	$^1/_2$	0
○	●	$^1/_2$	0	0	$^1/_2$
●	●	0	$^1/_2$	$^1/_2$	0

Previous state

Now we marginalize over the current states of elements outside the mechanism (**A** and **B**)

Calculating a cause repertoire:

Marginalizing-out non-mechanism elements

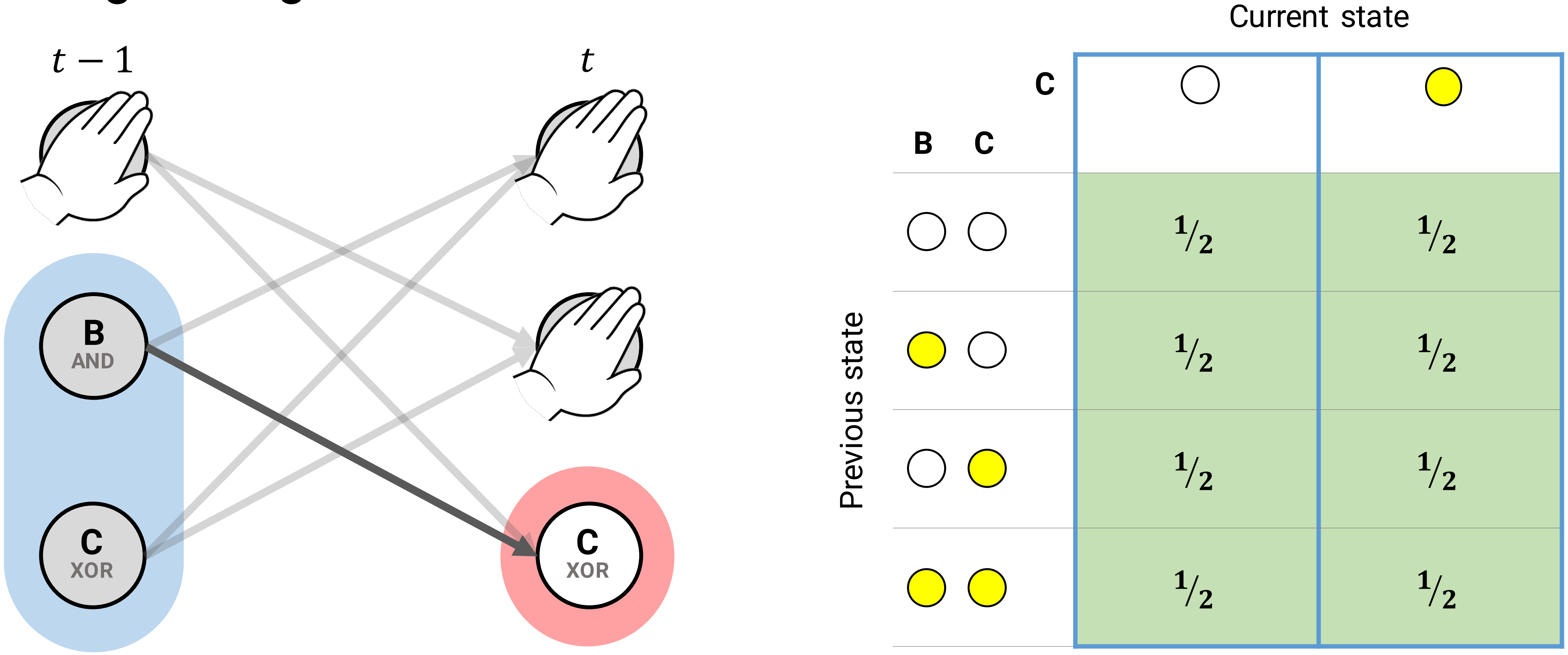

Now we marginalize over the current states of elements outside the mechanism (**A** and **B**)

Calculating a cause repertoire:
Marginalizing-out non-mechanism elements

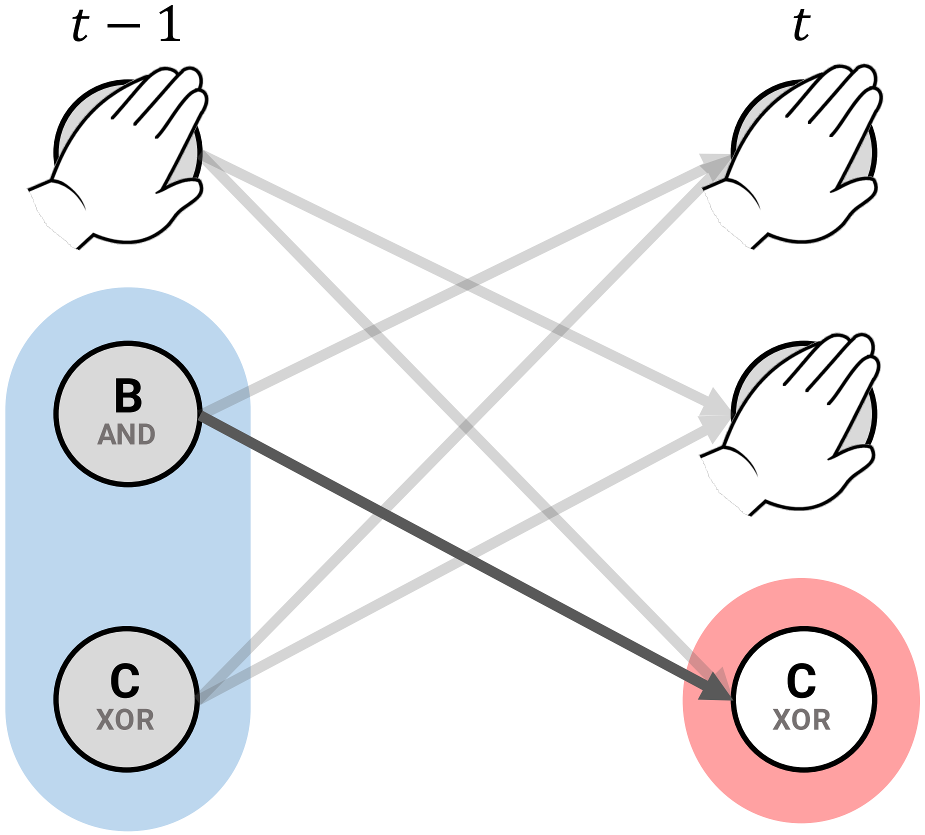

Current state

Previous state: B	Previous state: C	C = ○	C = ●
○	○	$^1/_2$	$^1/_2$
●	○	$^1/_2$	$^1/_2$
○	●	$^1/_2$	$^1/_2$
●	●	$^1/_2$	$^1/_2$

Now we marginalize over the current states of elements outside the mechanism (**A** and **B**)

Calculating a cause repertoire:
Conditioning on the mechanism

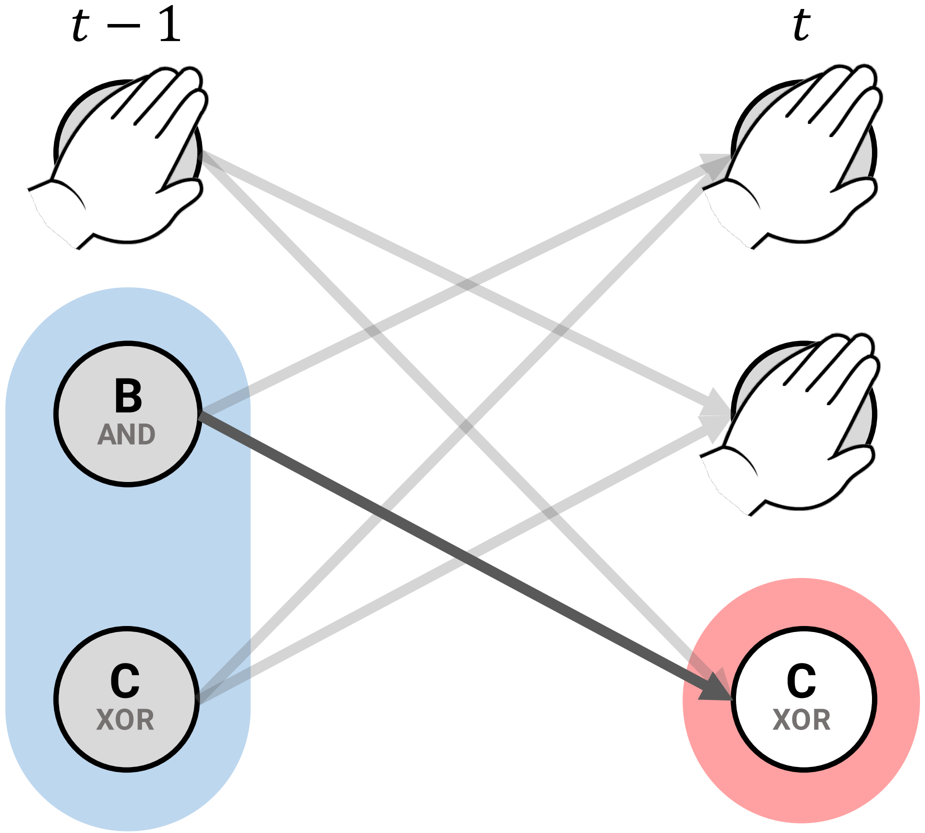

The next step is to condition on the current state of the mechanism, **C**

Current state

Previous state: B	Previous state: C	C = ○	C = ●
○	○	$^1/_2$	$^1/_2$
●	○	$^1/_2$	$^1/_2$
○	●	$^1/_2$	$^1/_2$
●	●	$^1/_2$	$^1/_2$

Calculating a cause repertoire:
Conditioning on the mechanism

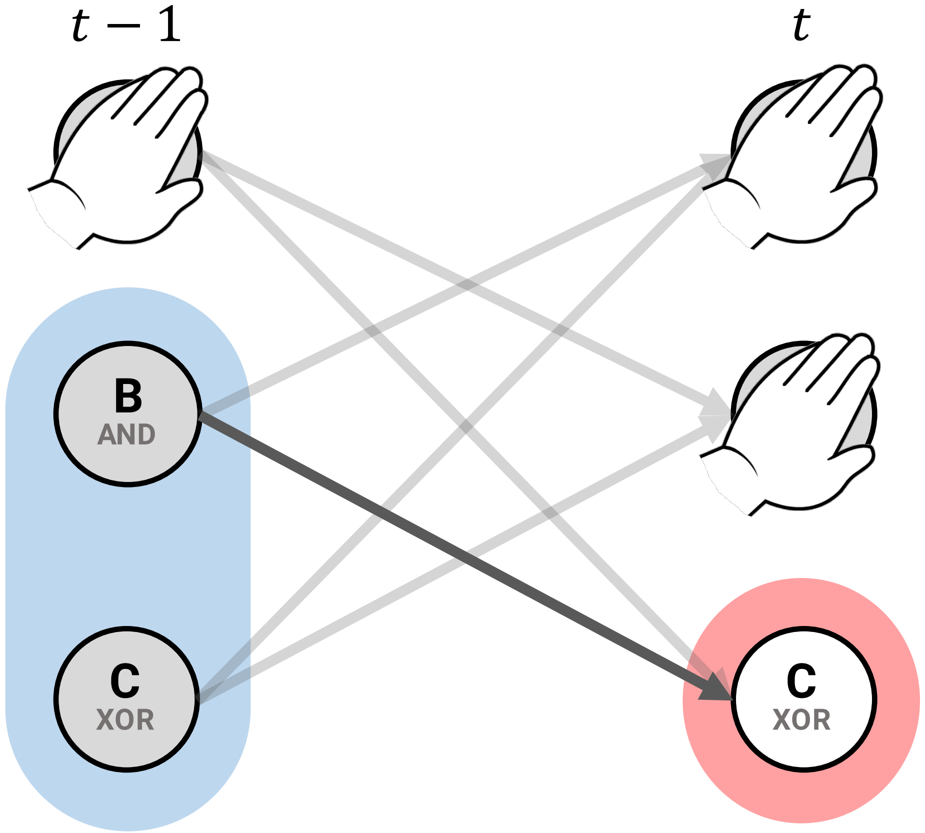

Current state

Previous state

B	C	C: ○	C: ●
○	○	$^1/_2$	$^1/_2$
●	○	$^1/_2$	$^1/_2$
○	●	$^1/_2$	$^1/_2$
●	●	$^1/_2$	$^1/_2$

This is done by simply taking the column corresponding to **C**'s current state

Calculating a cause repertoire:
Conditioning on the mechanism

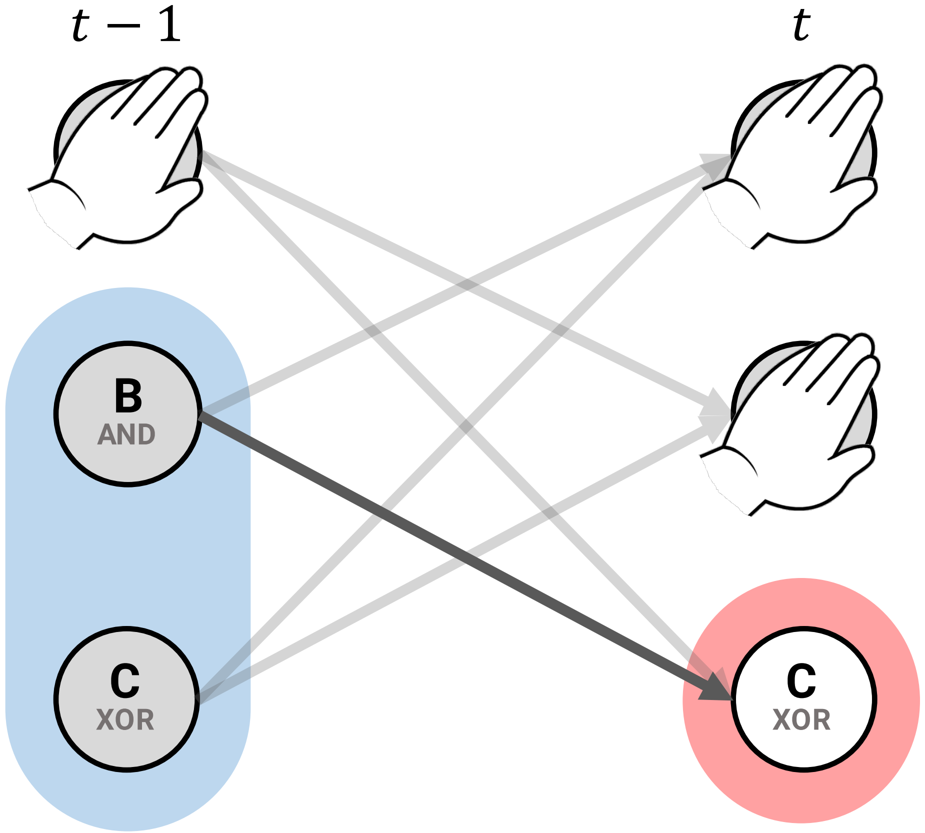

Current state

Previous state: B	C	C = ○	C = ●
○	○	$^1/_2$	$^1/_2$
●	○	$^1/_2$	$^1/_2$
○	●	$^1/_2$	$^1/_2$
●	●	$^1/_2$	$^1/_2$

This is done by simply taking the column corresponding to **C**'s current state

Calculating a cause repertoire:

Conditioning on the mechanism

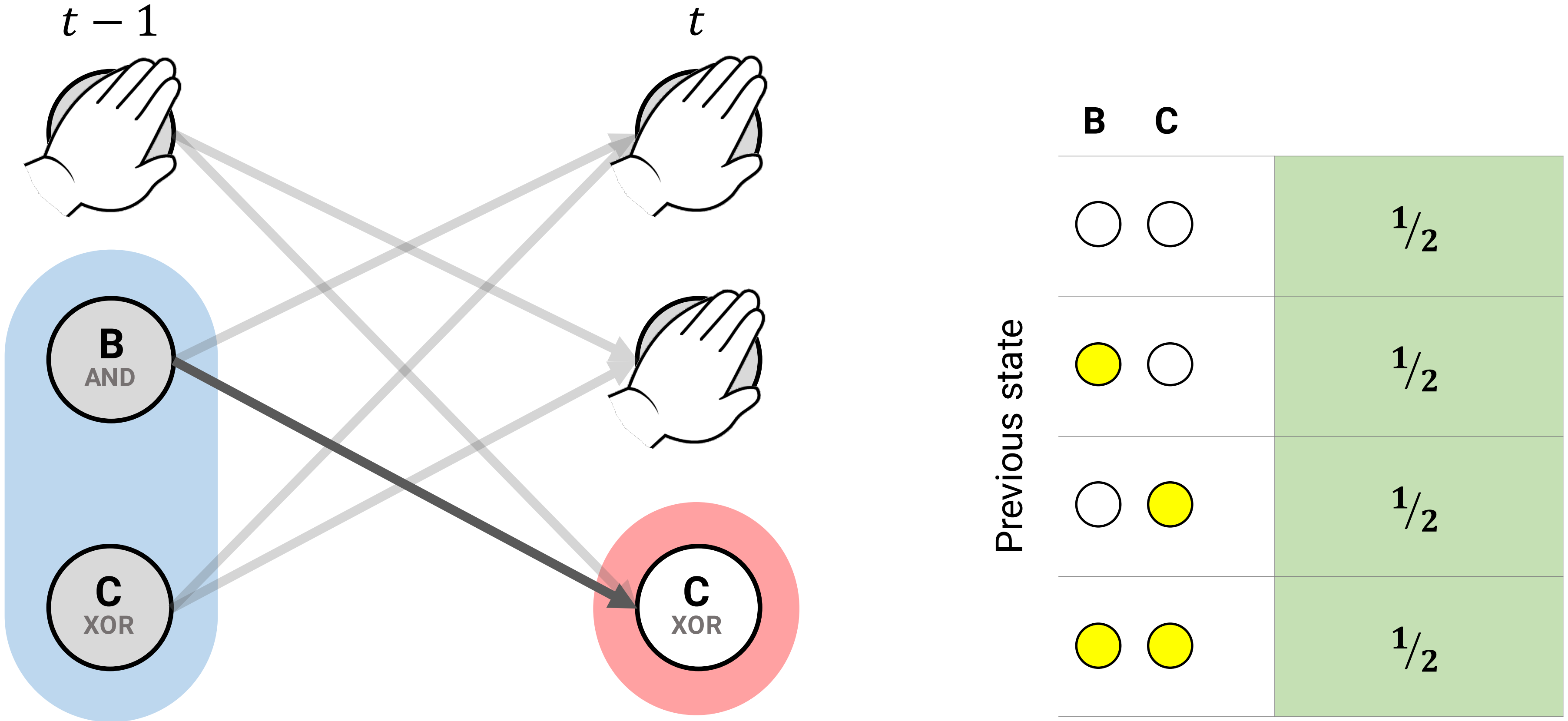

This is done by simply taking the column corresponding to **C**'s current state

Calculating a cause repertoire:
Renormalizing

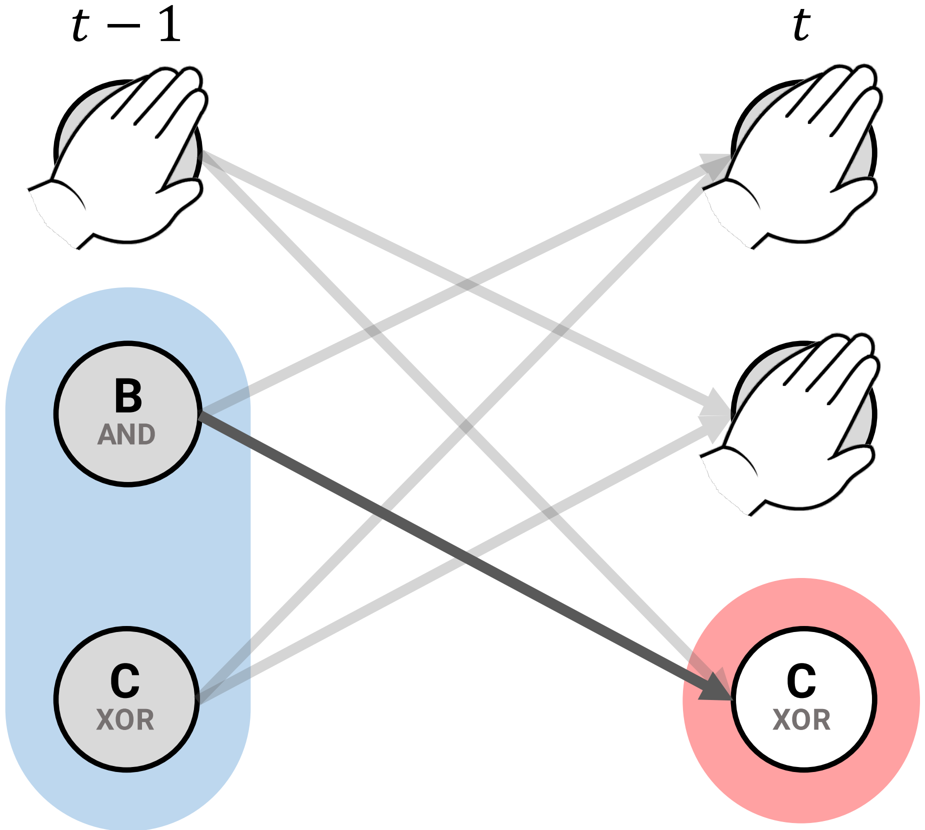

Previous state: B	C	
○	○	$^1/_2$
●	○	$^1/_2$
○	●	$^1/_2$
●	●	$^1/_2$

And finally, we renormalize to obtain a proper distribution (not needed in this example, but required in general since columns of the TPM do not necessarily sum to 1)

Calculating a cause repertoire:
Renormalizing

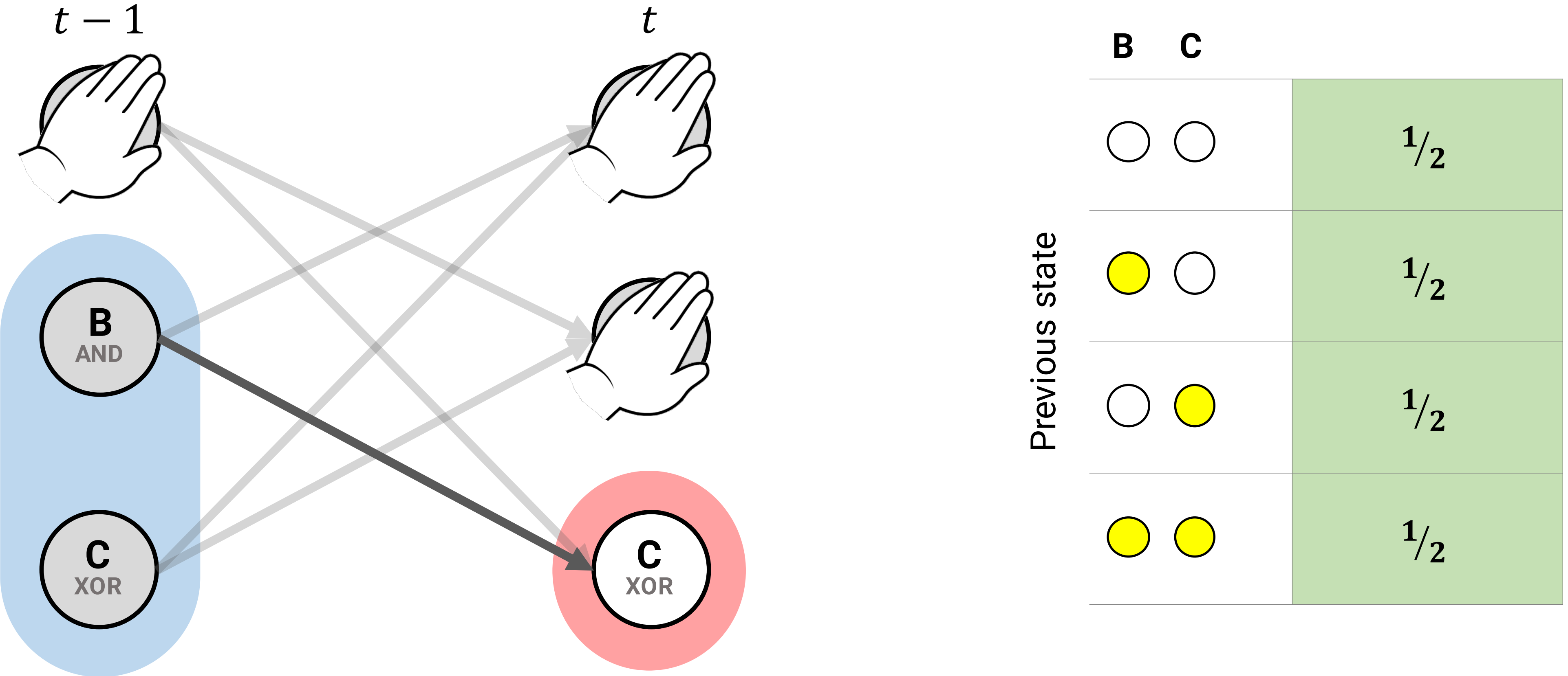

This is the cause repertoire of **C** over **BC** when the system is in state (1, 0, 0)

Calculating a cause repertoire:
Renormalizing

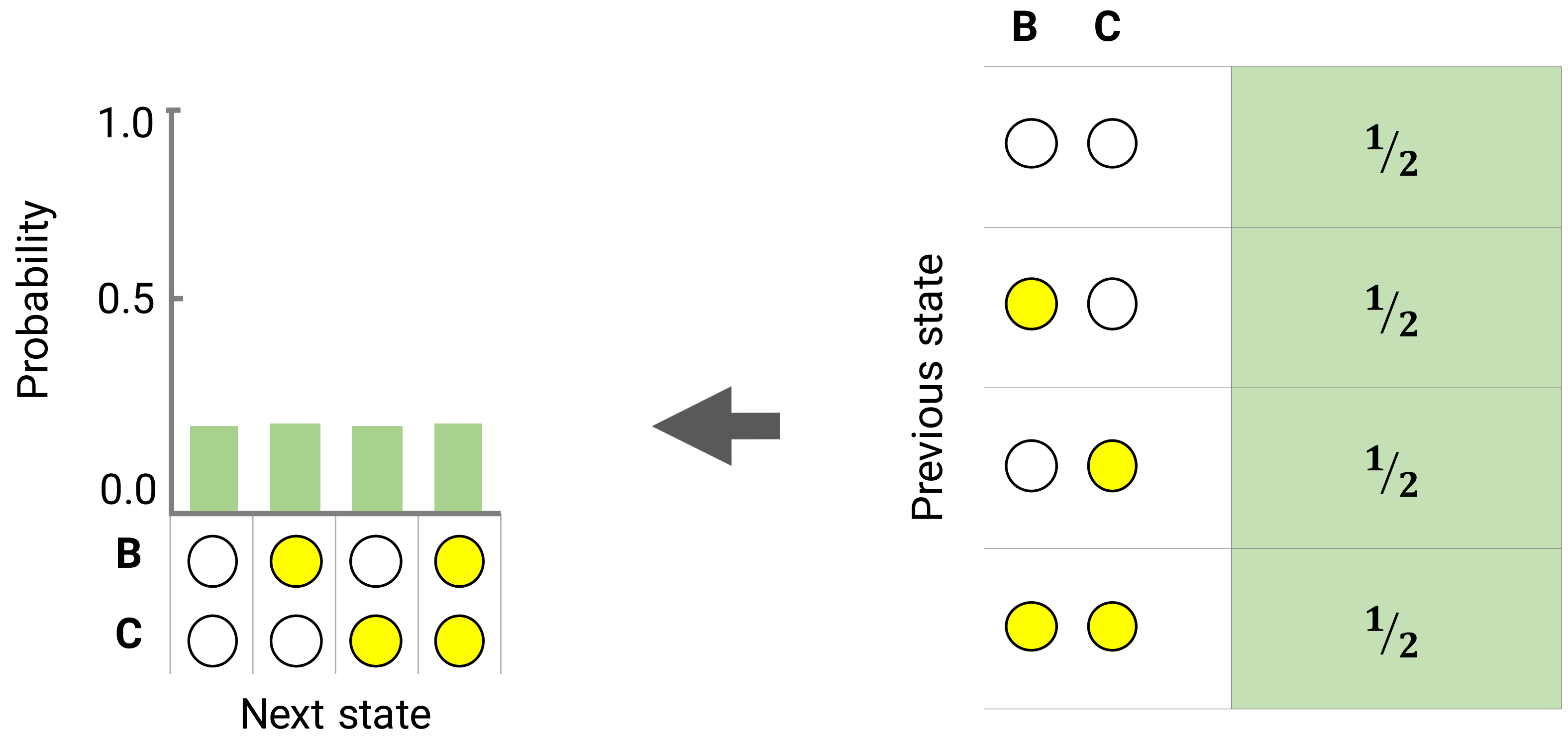

This is the cause repertoire of **C** over **BC** when the system is in state (1, 0, 0)

Calculating a cause repertoire:
Expanding to the full state-space

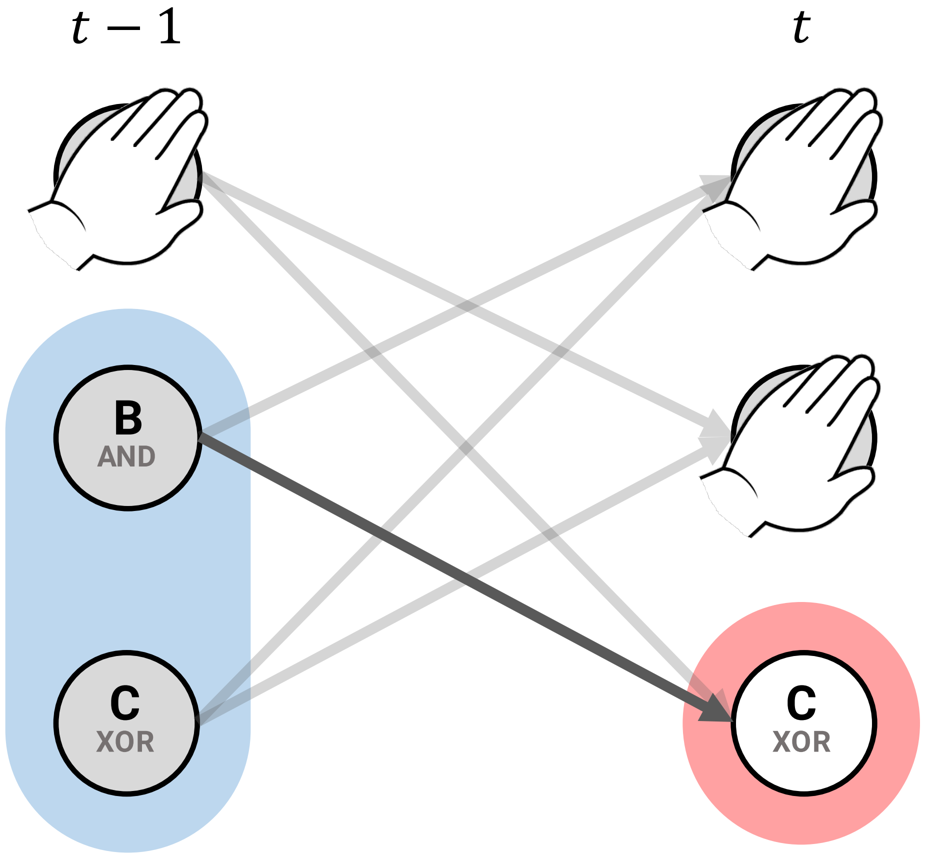

Previous state

B	C	
○	○	$^1/_4$
●	○	$^1/_4$
○	●	$^1/_4$
●	●	$^1/_4$

Now, as with the effect repertoire, we can multiply this distribution by the unconstrained cause repertoire of the non-purview elements to get a distribution over the entire state space

Calculating a cause repertoire:
Expanding to the full state-space

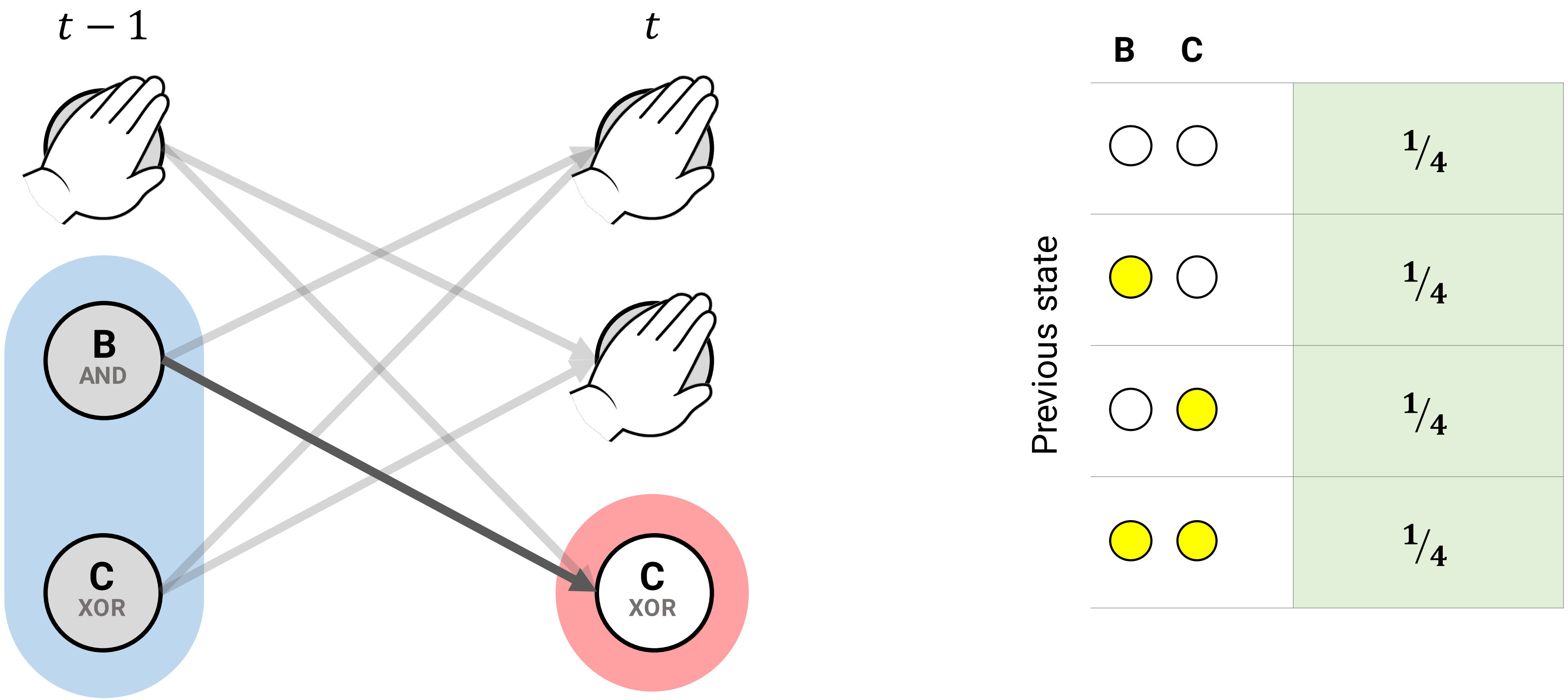

Recall that all previous states are equally likely in the perturbation, so the unconstrained cause repertoire of the non-mechanism elements is simply the uniform distribution

Calculating a cause repertoire:

Expanding to the full state-space

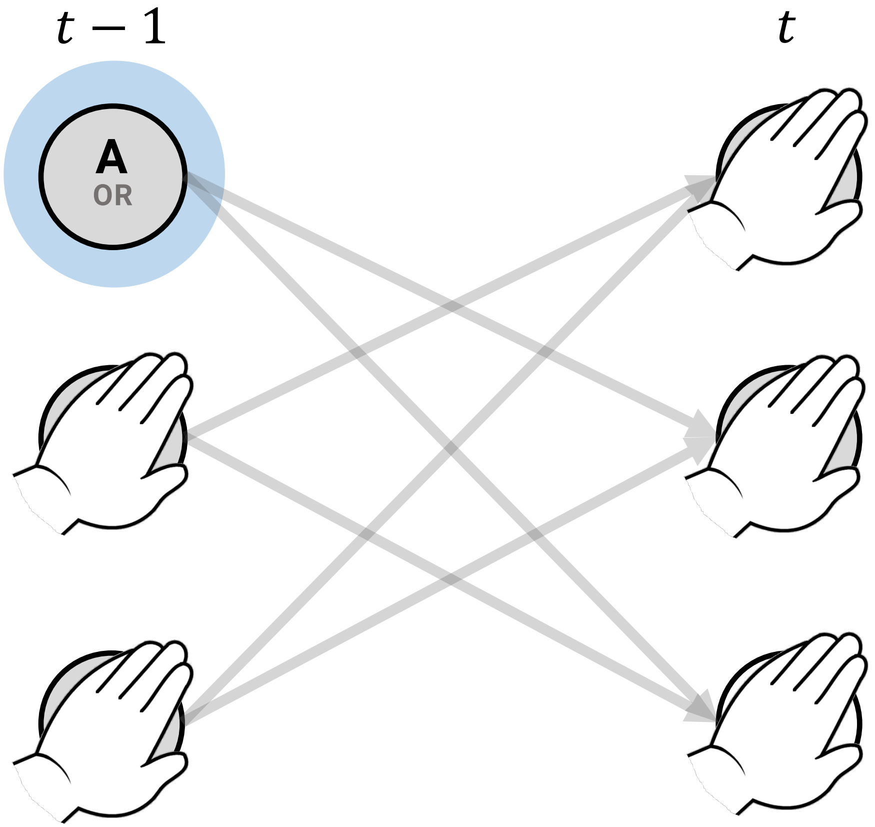

Recall that all previous states are equally likely in the perturbation, so the unconstrained cause repertoire of the non-mechanism elements is simply the uniform distribution

Calculating a cause repertoire:
Expanding to the full state-space

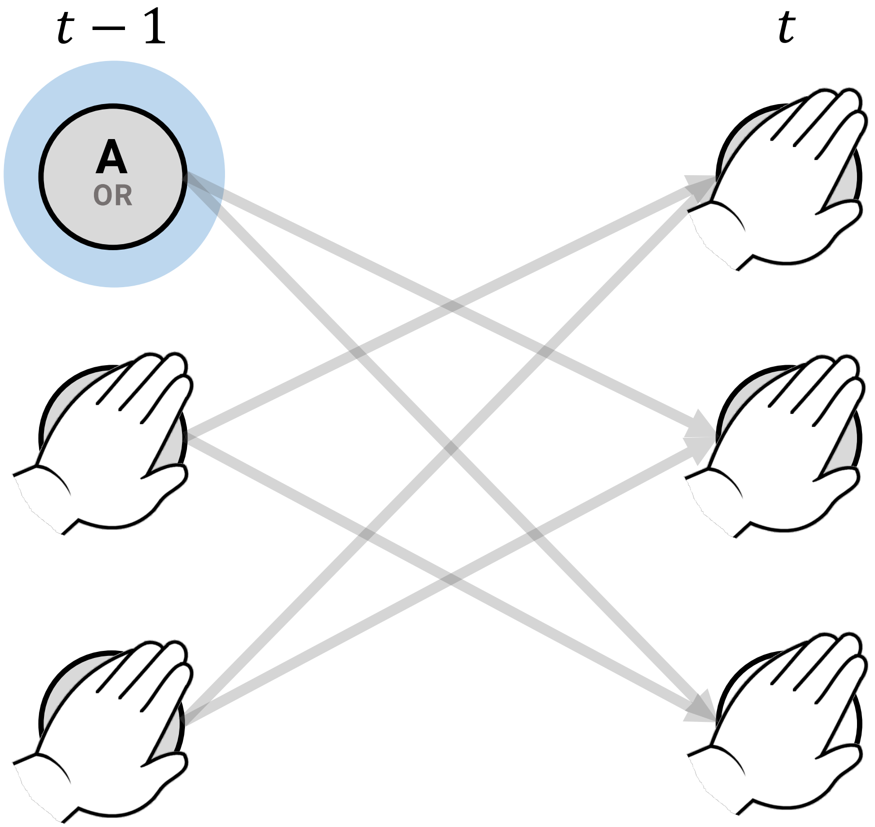

Previous state	A	
	○ (off)	$^1/_2$
	● (on)	$^1/_2$

Recall that all previous states are equally likely in the perturbation, so the unconstrained cause repertoire of the non-mechanism elements is simply the uniform distribution

Calculating a cause repertoire:
Expanding to the full state-space

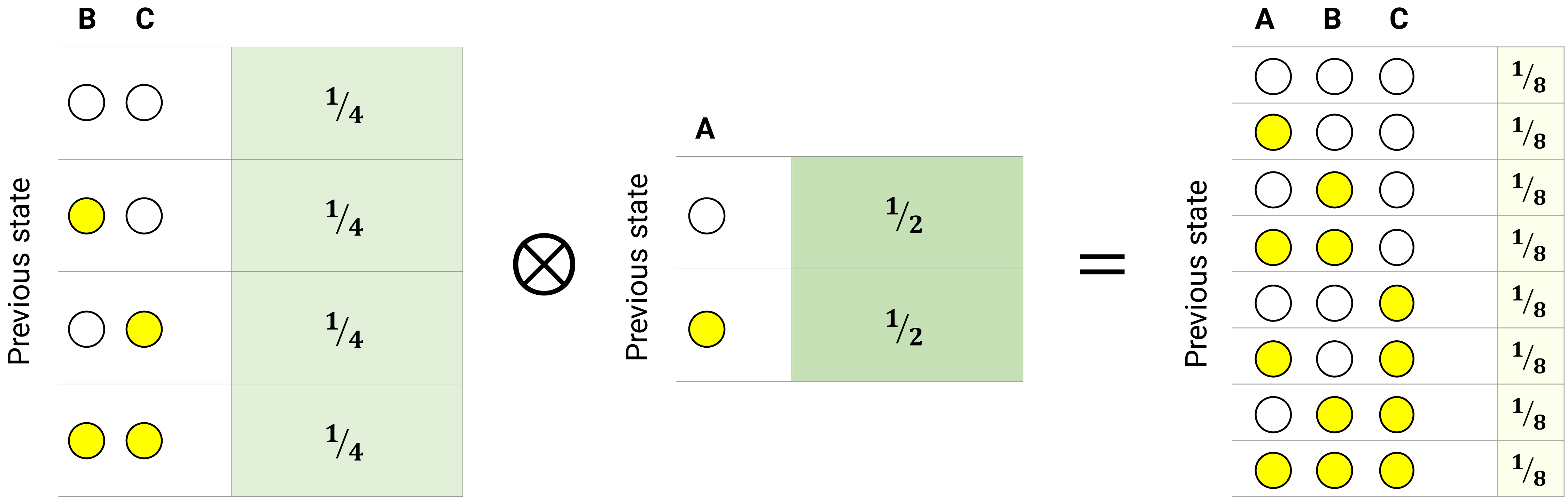

Now we can multiply the cause repertoire over the purview by the unconstrained repertoire to get the cause repertoire over the whole system's state at $t - 1$

Calculating a cause repertoire:

Expanding to the full state-space

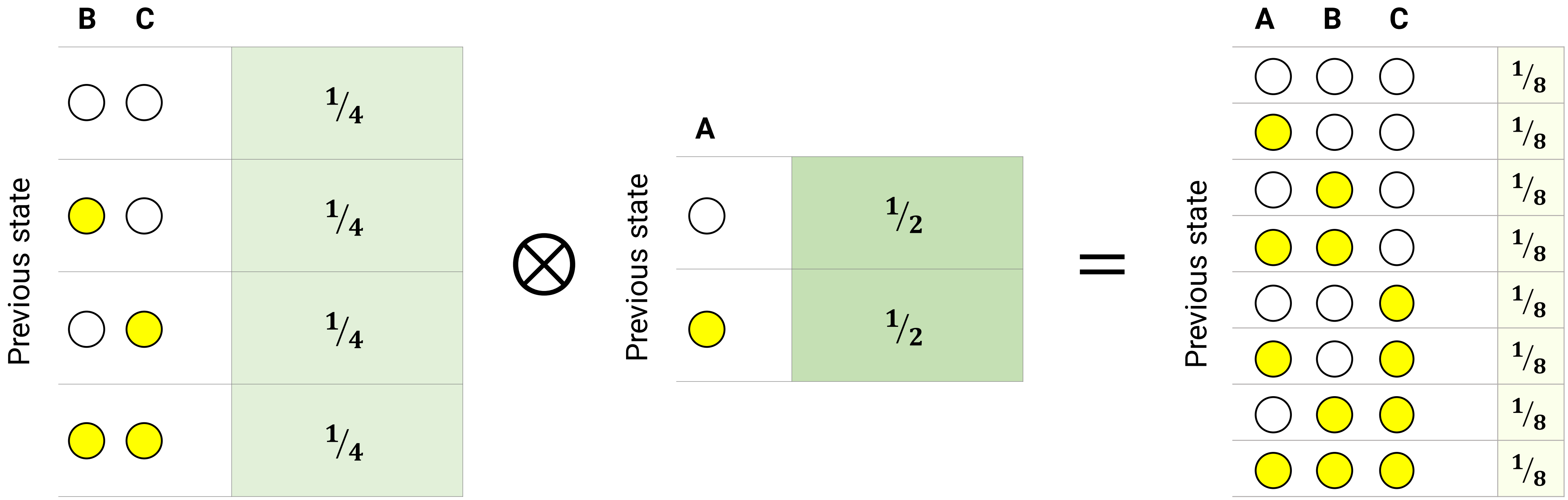

This is the expanded cause repertoire

Outline

- Elements, states, and the TPM
- Background conditions
- Cause-effect repertoires
- Integrated mechanisms: φ
- Concepts and cause-effect structures
- Integrated systems: Φ
- Complexes

Integration and irreducibility

- The cause and effect repertoires quantify to what extent a candidate mechanism has selective causes and effects within the system
- Since IIT is concerned with the **intrinsic perspective** of the system, we are interested in whether or not a given candidate mechanism's causes and effects are **reducible** to the causes and effects of its parts
- If the candidate mechanism's causes and effects reduce to those of its parts, then there is nothing gained in terms of information by grouping the parts together in the first place
- The set of elements *per se* doesn't make a difference to the system

Integration and reducibility:

An example of a reducible candidate mechanism

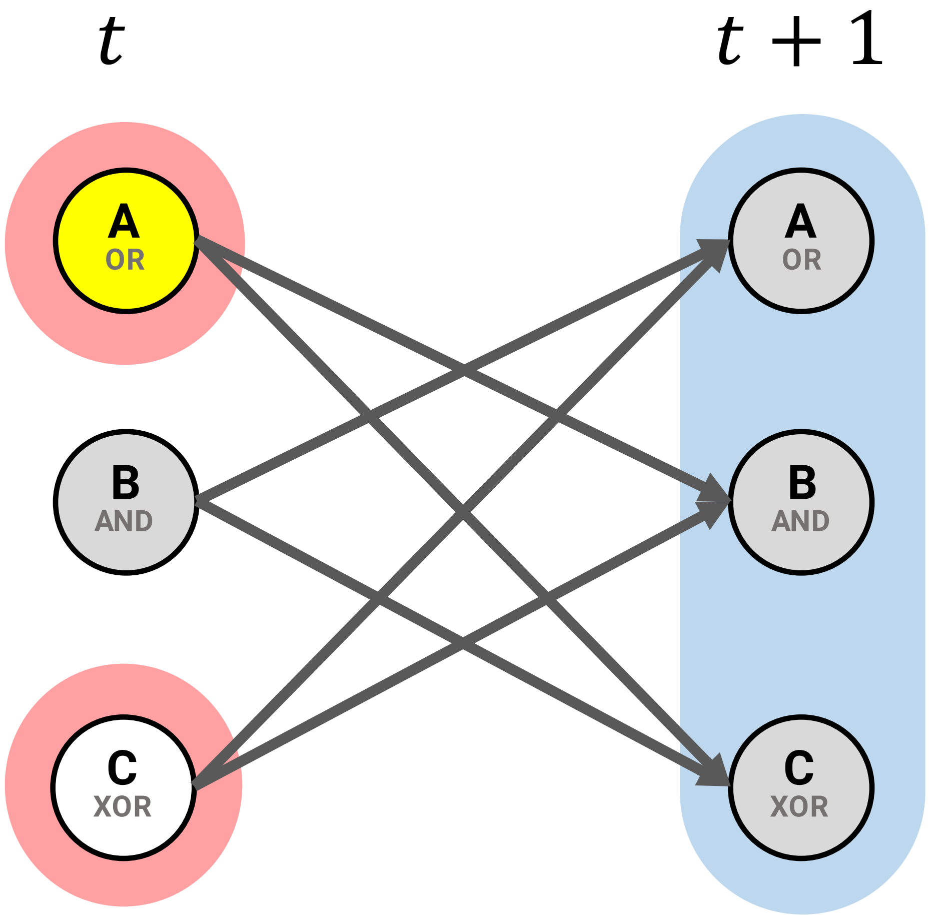

- Consider the mechanism **AC** over the purview **ABC**

Integration and reducibility:

An example of a reducible candidate mechanism

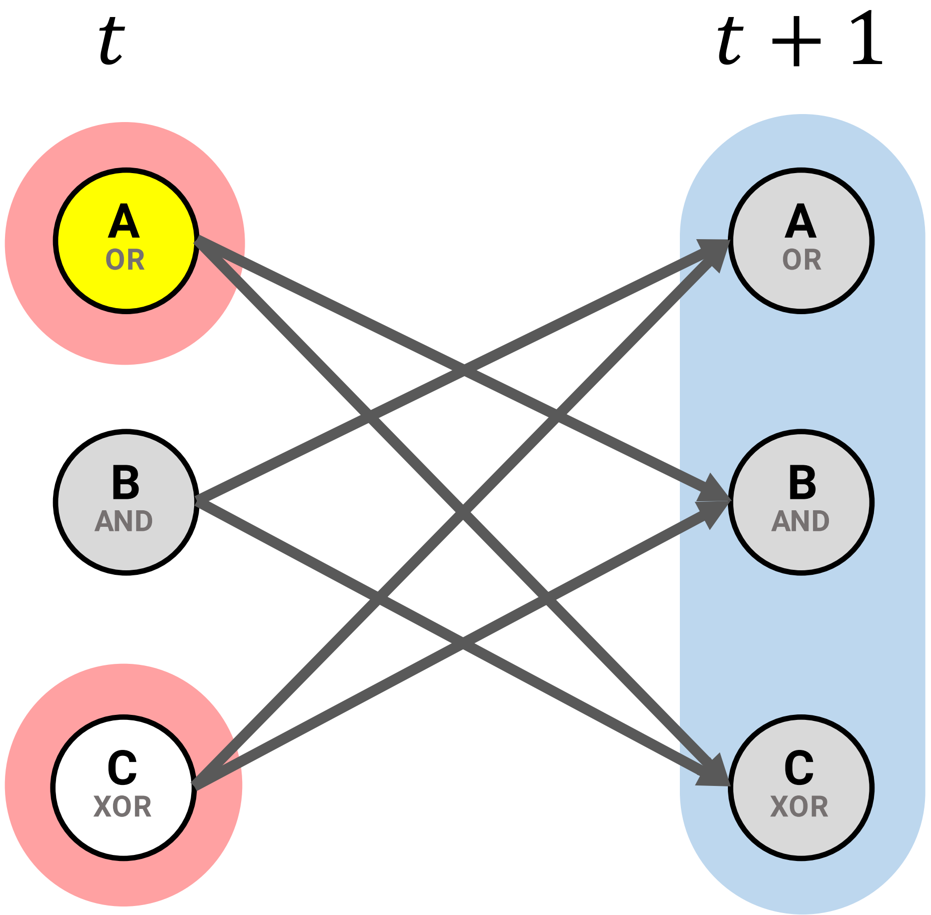

- Consider the mechanism **AC** over the purview **ABC**
- It has the following effect repertoire:

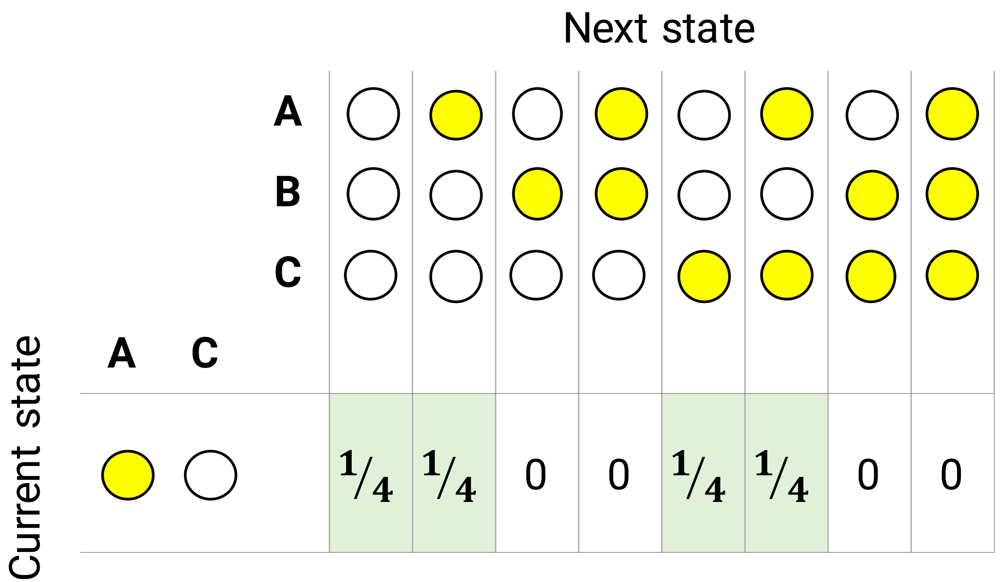

Integration and reducibility:

An example of a reducible candidate mechanism

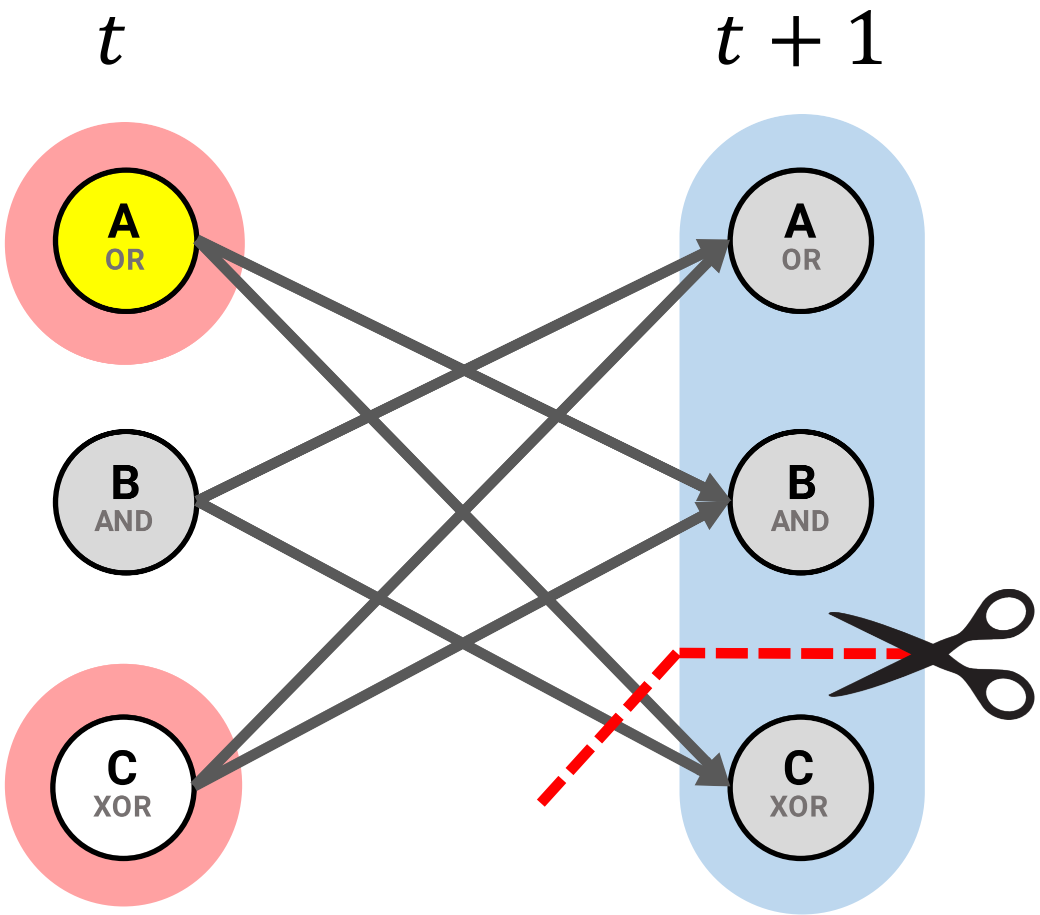

- Now we can partition the purview into **AB** and **C**
- Then we consider the effect repertoire of the mechanism **AC** over **AB** and the unconstrained repertoire of **C**
- In other words, we can separate the repertoire $\frac{AC}{ABC}$ into $\frac{AC}{AB}$ and $\frac{\emptyset}{C}$

Integration and reducibility:
An example of a reducible mechanism

- We calculate the unconstrained repertoire of **C**:

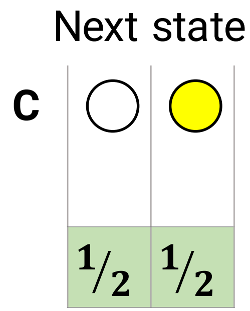

- And the repertoire **AC** over **AB**:

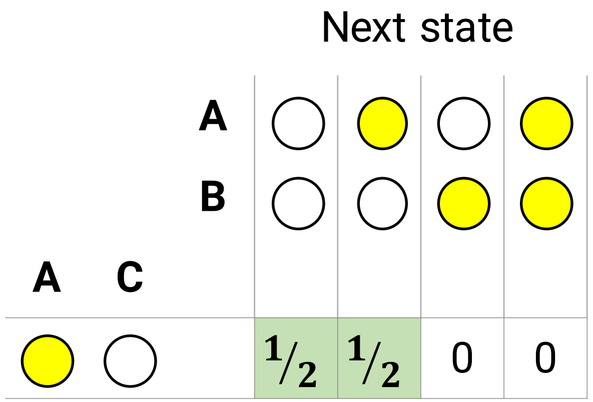

Integration and reducibility:
An example of a reducible mechanism

- Now we take the tensor product to obtain a repertoire over the original purview, **ABC**:

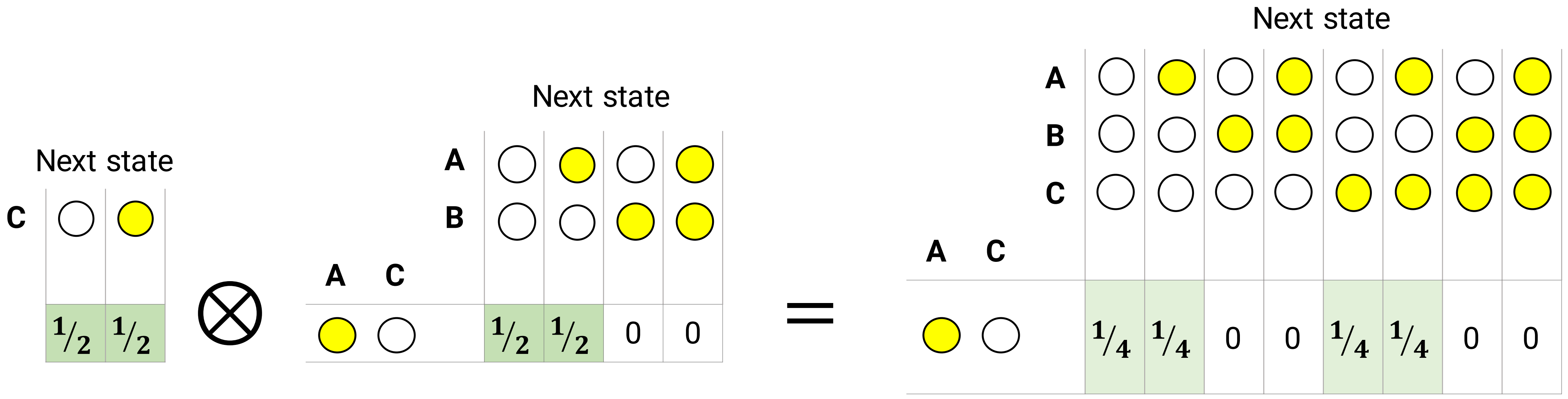

Integration and reducibility:

An example of a reducible mechanism

- And we see that we've recovered the original effect repertoire of **AC** over **ABC**
- This means that the repertoire of $\frac{AC}{ABC}$ can be "factored" into $\frac{AC}{AB}$ and $\frac{\emptyset}{C}$
- In other words, the repertoire of **AC** over **ABC** is **reducible** to that of **AC** over **AB**
- There is no information gained by including **C** in the purview

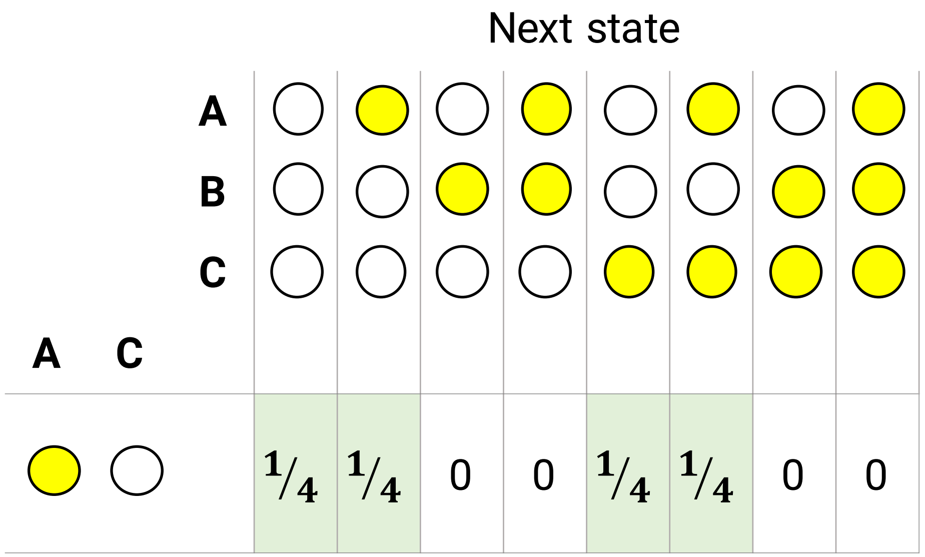

Un-partitioned

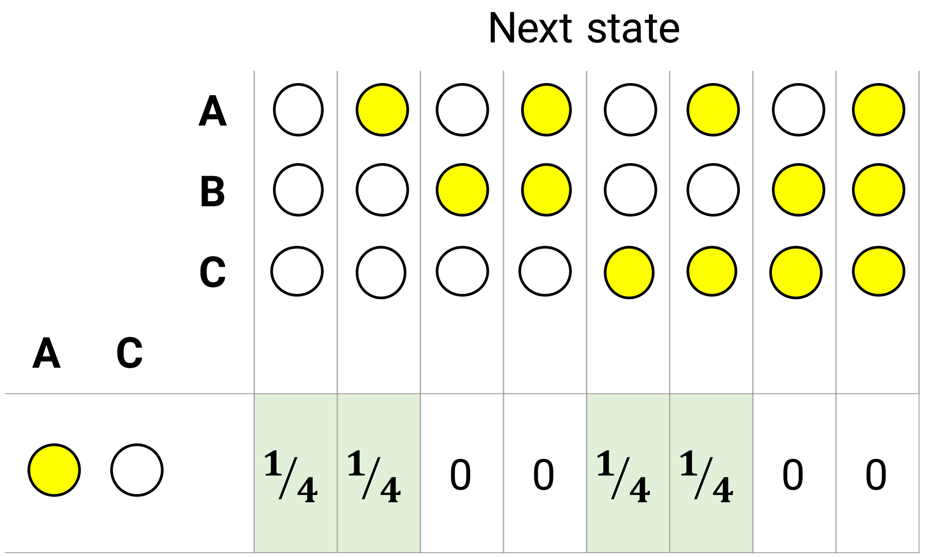

Partitioned

Integration and reducibility:
Minimum information partition and “small-phi”

- However, note that we can try to factor the repertoire in many different ways:

$$\frac{\emptyset}{A} \times \frac{AC}{BC} \qquad \frac{\emptyset}{B} \times \frac{AC}{AC} \qquad \frac{\emptyset}{AB} \times \frac{AC}{C}$$

$$\frac{\emptyset}{C} \times \frac{AC}{BC} \qquad \frac{\emptyset}{AC} \times \frac{AC}{B} \qquad \frac{\emptyset}{BC} \times \frac{AC}{A} \qquad \frac{\emptyset}{ABC} \times \frac{AC}{\emptyset}$$

$$\frac{A}{\emptyset} \times \frac{C}{ABC} \qquad \frac{A}{A} \times \frac{C}{BC} \qquad \frac{A}{B} \times \frac{C}{AC} \qquad \frac{A}{AB} \times \frac{C}{C}$$

$$\frac{A}{C} \times \frac{C}{BC} \qquad \frac{A}{AC} \times \frac{C}{B} \qquad \frac{A}{BC} \times \frac{C}{A} \qquad \frac{A}{ABC} \times \frac{C}{\emptyset}$$

Integration and reducibility:
Minimum information partition and “small-phi”

- However, note that we can try to factor the repertoire in many different ways:
- We calculate the repertoire for each of these possible partitions
- Then we compare each of the partitioned repertoires to the original repertoire by calculating the distance between them
- PyPhi supports various distance measures, but we’ll explore the Earth Mover’s Distance (EMD) used in IIT 3.0

Integration and reducibility:
The Earth Mover's Distance

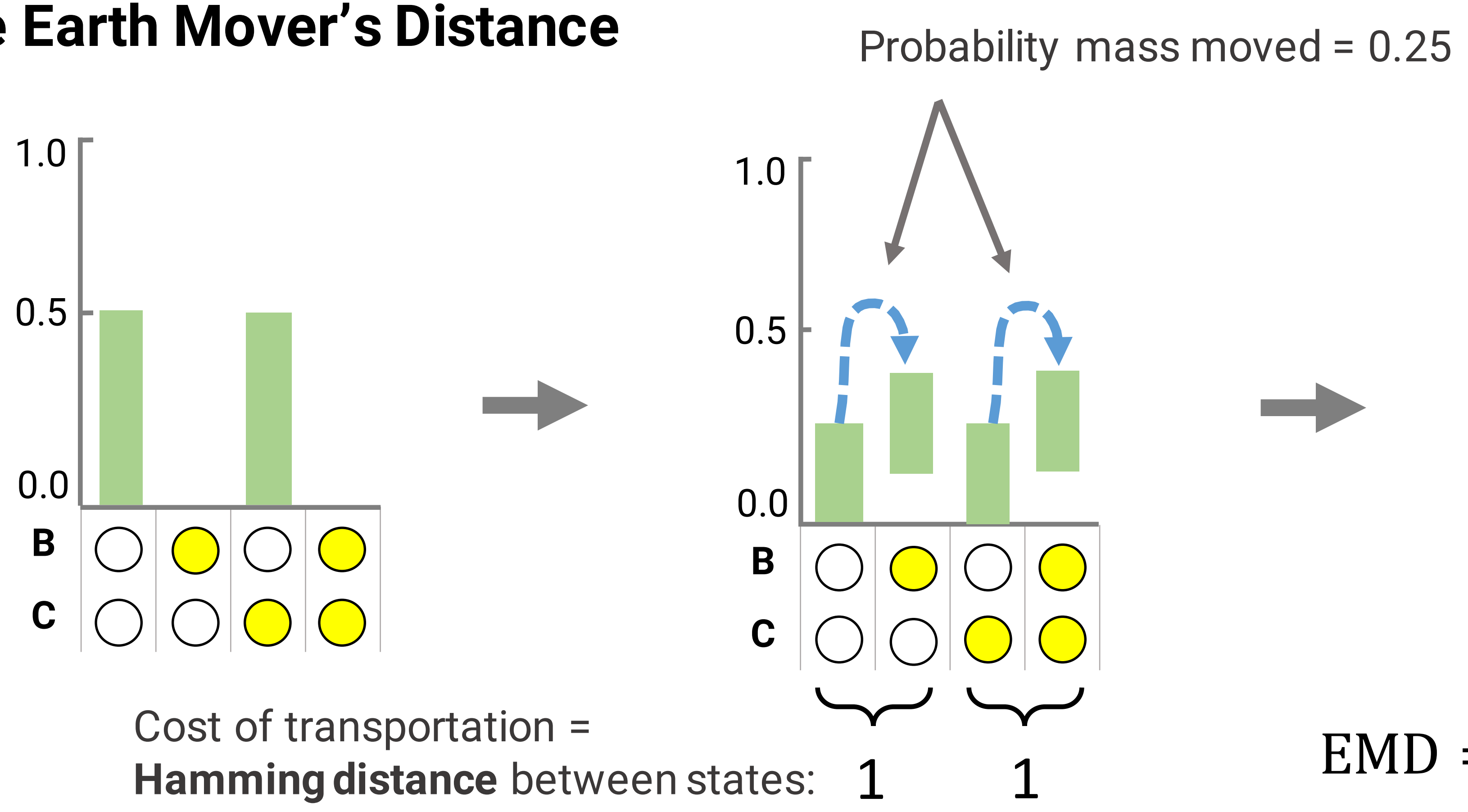

$$\mathrm{EMD} = 0.25 \times 1 + 0.25 \times 1$$
$$= 0.50$$

The EMD is the minimum cost of transforming one pile of "dirt" into the other, where the cost is the **amount of dirt moved** multiplied by the **distance it travels**

Integration and reducibility:
Minimum information partition and "small-phi"

- The partition corresponding to the minimal distance from the original repertoire is the **minimum information partition**
- It's the partition that results in the smallest loss of information
- The EMD between the unpartitioned repertoire and the repertoire of the MIP quantifies **how irreducible** the unpartitioned repertoire is
- This quantity is called **integrated information**, denoted φ ("small-phi"), because it's the information that is contained in the repertoire *by virtue of considering the mechanism as an integrated whole*

Outline

- Elements, states, and the TPM
- Background conditions
- Cause-effect repertoires
- Integrated mechanisms: φ
- Concepts and cause-effect structures
- Integrated systems: Φ
- Complexes

Integration and reducibility:

Maximally-irreducible cause-effect repertoire of mechanism ABC

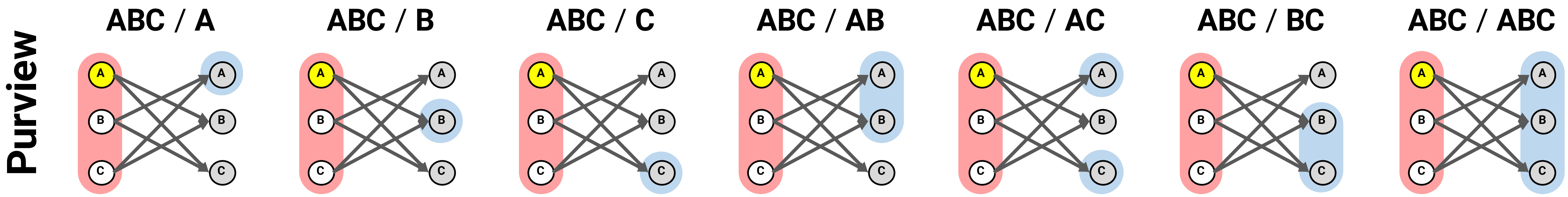

For a given candidate mechanism, we can find the cause and effect repertoires over all possible purviews (the power set of the system)

Integration and reducibility:

Maximally-irreducible cause-effect repertoire of mechanism ABC

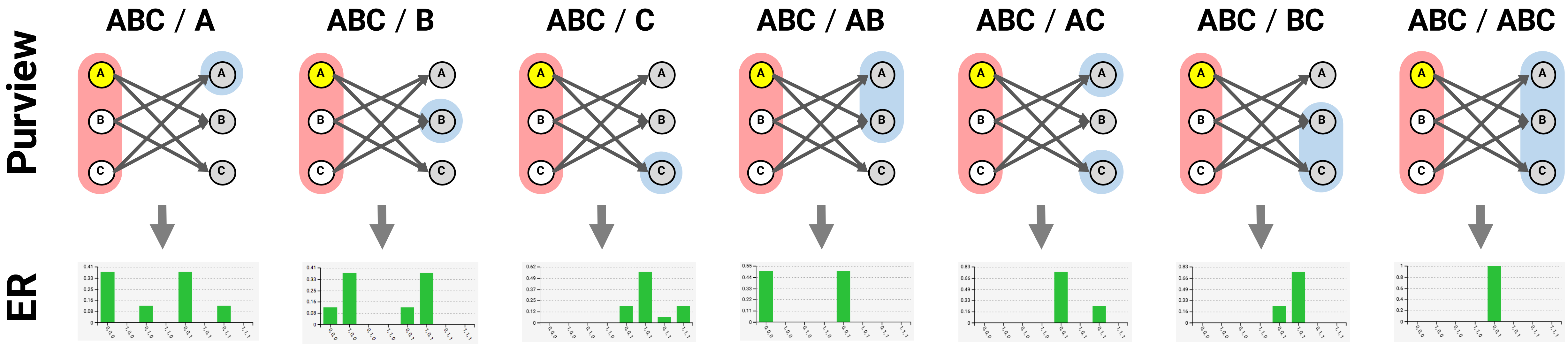

For a given candidate mechanism, we can find the cause and effect repertoires over all possible purviews (the power set of the system)

Integration and reducibility:

Maximally-irreducible cause-effect repertoire of mechanism ABC

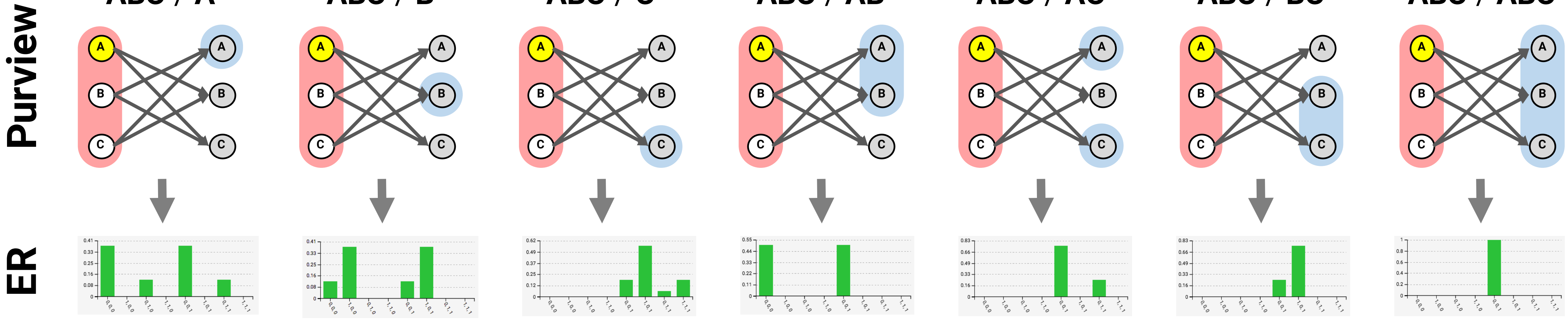

	ABC / A	ABC / B	ABC / C	ABC / AB	ABC / AC	ABC / BC	ABC / ABC
MIP	$\frac{A}{\emptyset}\times\frac{BC}{A}$	$\frac{A}{\emptyset}\times\frac{BC}{B}$	$\frac{C}{\emptyset}\times\frac{AB}{C}$	$\frac{A}{\emptyset}\times\frac{BC}{AB}$	$\frac{\emptyset}{C}\times\frac{ABC}{A}$	$\frac{AB}{C}\times\frac{C}{B}$	$\frac{\emptyset}{B}\times\frac{ABC}{AC}$
φ	$\varphi = 0$	$\varphi = 0$	$\varphi = 0$	$\varphi = 0$	$\varphi = 0.5$	$\varphi = 0$	$\varphi = 0.25$

Then we can find the MIP and φ value for each repertoire

Integration and reducibility:

Maximally-irreducible cause-effect repertoire of mechanism ABC

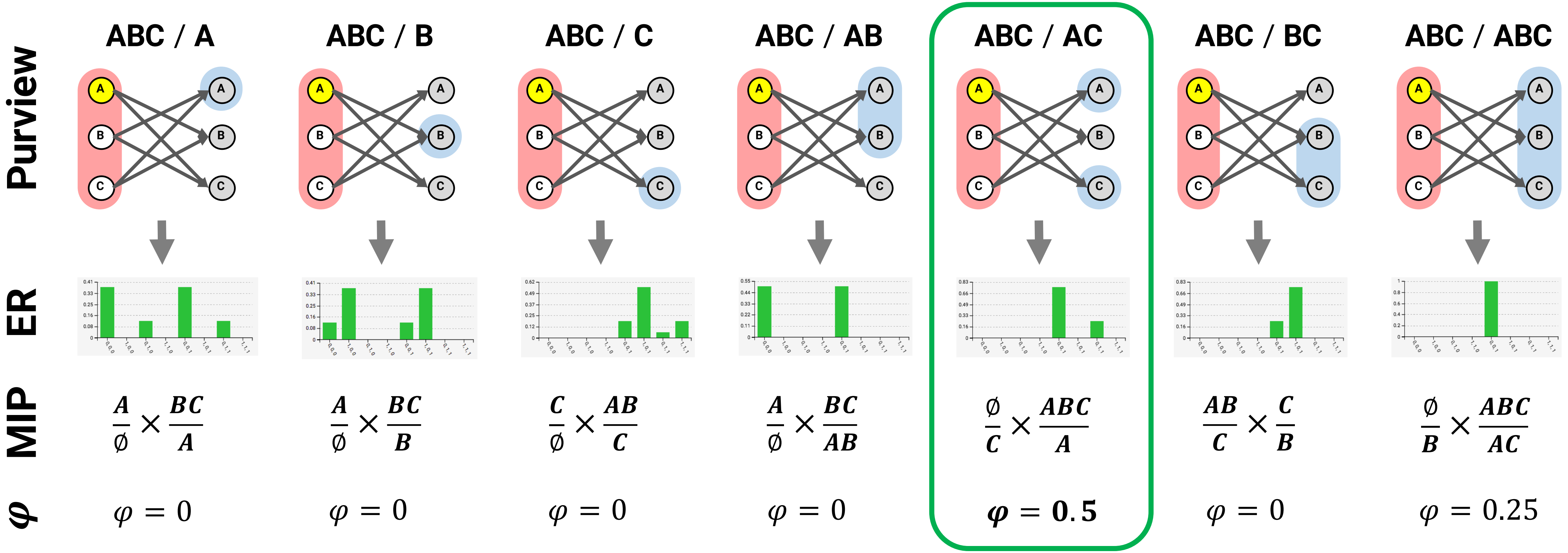

The repertoire whose MIP has the highest φ value ($\varphi^{\max}$) is the **maximally-irreducible effect repertoire** for mechanism **ABC**
(the maximally-irreducible cause repertoire is defined similarly)

Integration and reducibility:
Concepts

- The maximally-irreducible cause and effect repertoires of **ABC**, and their φ_{cause} and φ_{effect} values, together form the **concept** specified by **ABC**
- The irreducibility of the concept as a whole is the minimum of its maximally-irreducible cause and effect:

$$\varphi = \min(\varphi_{\text{cause}}, \varphi_{\text{effect}})$$

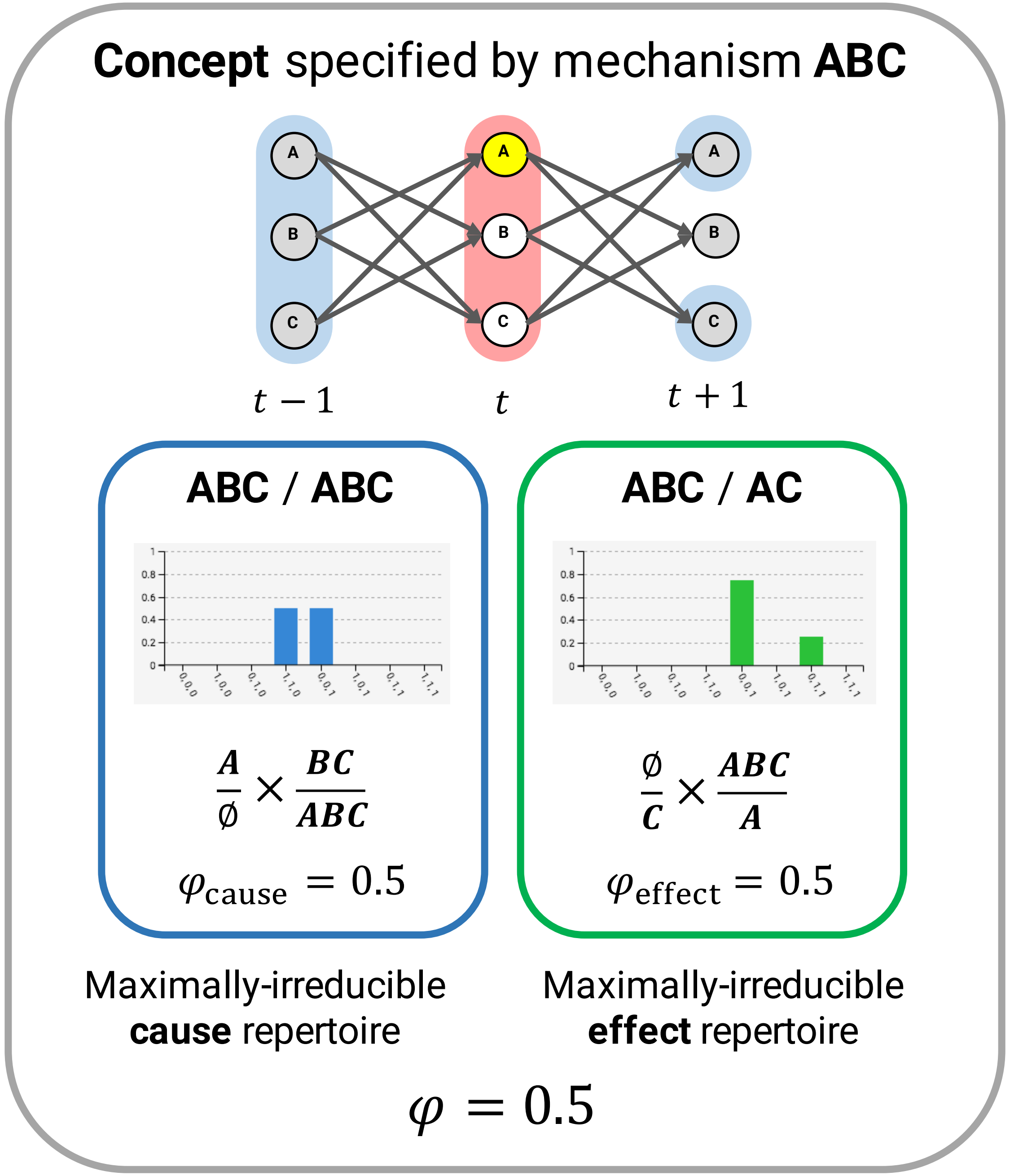

Integration and reducibility:
Cause-effect structures

- In this way we can calculate the concept specified by every candidate mechanism
- The collection of all the concepts with nonzero φ is the system's **cause-effect structure**:

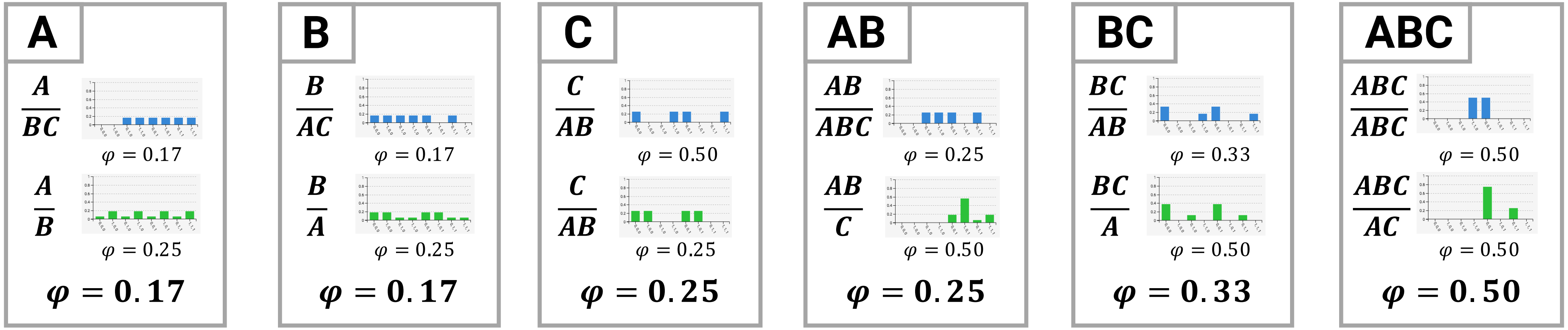

Outline

- Elements, states, and the TPM
- Background conditions
- Cause-effect repertoires
- Integrated mechanisms: φ
- Concepts and cause-effect structures
- Integrated systems: Φ
- Complexes

Integration and reducibility:

System-level irreducibility and system cuts

- At this point, we have assessed which subsets of elements of the candidate system exist intrinsically as integrated mechanisms with irreducible cause-effect power
- But what about the system as a whole?
- We can determine whether our candidate system is an integrated, irreducible entity using the same general scheme as when calculating φ

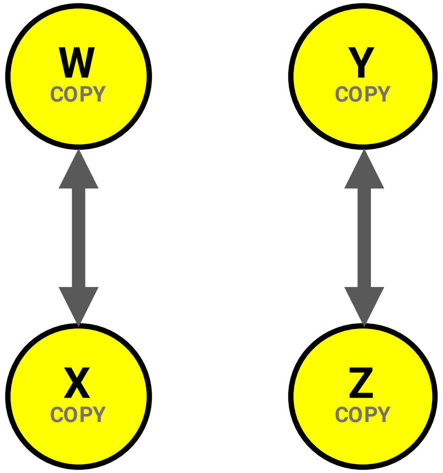

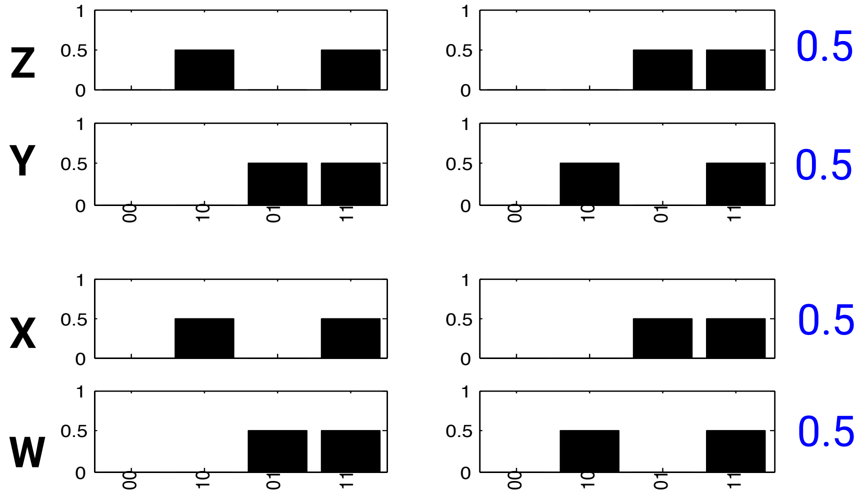

Integration and reducibility:
System-level irreducibility and system cuts

- The idea is to cut the system into two groups of elements, and remove the causal link from the first group to the second (a **unidirectional cut**)
- Then we can see whether the cut "makes a difference"
- If it doesn't, then the system reduces to the two parts separated by the cut

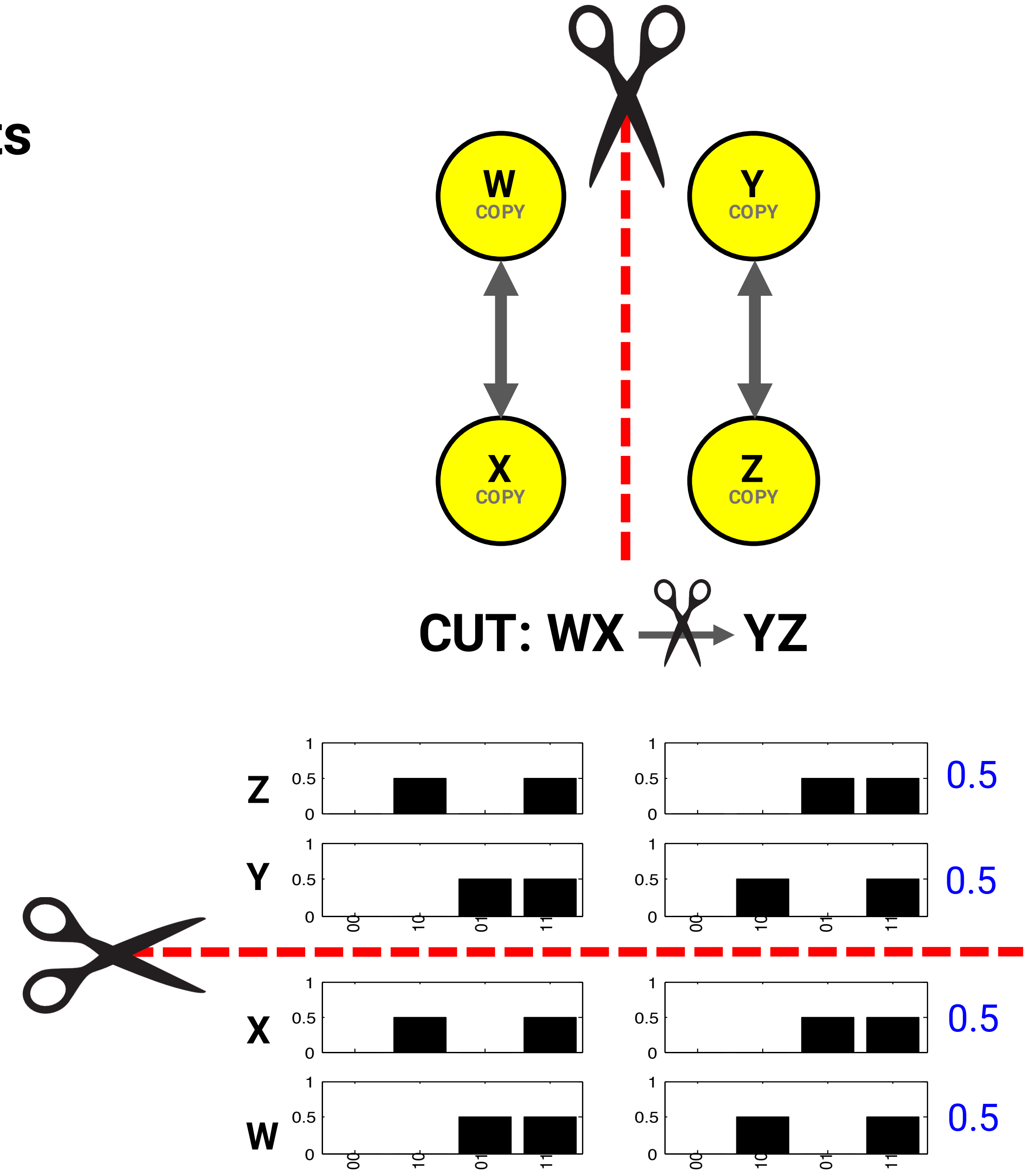

Integration and reducibility:

System-level irreducibility and system cuts

- Here, we can see immediately that the cut makes no difference to the system
- The cause-effect structure is unchanged by the cut
- **WXYZ** reduces to **WX** and **YZ**

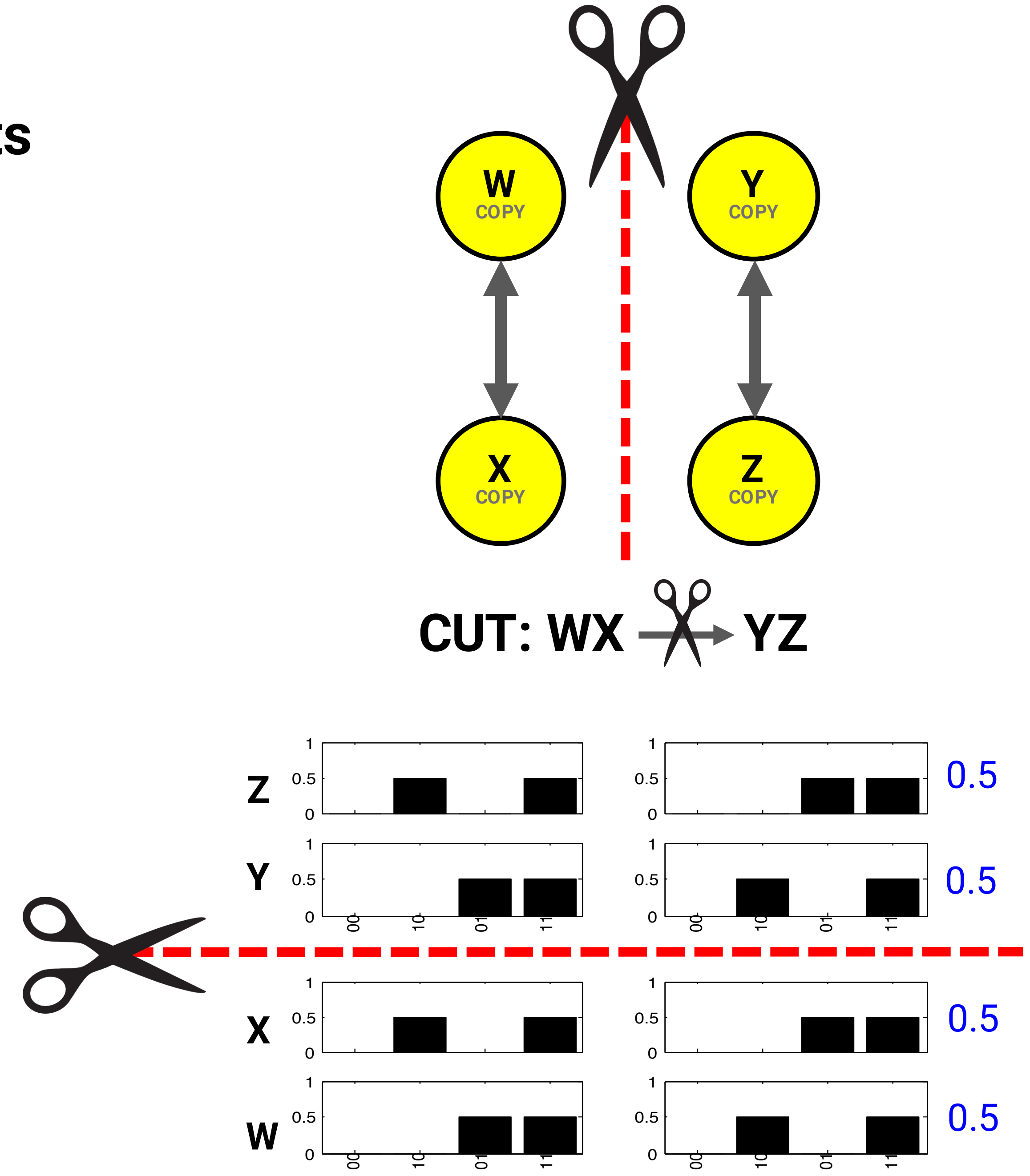

Integration and reducibility:
System-level irreducibility and system cuts

- But what is the proper way to “remove the causal link” from one group of elements to the other when there are connections between them?
- The right way to cut a connection is to **inject noise** into it, rather than simply removing it
- In this example, the outgoing connections from **A** *independently* provide random input to elements **B** and **C**

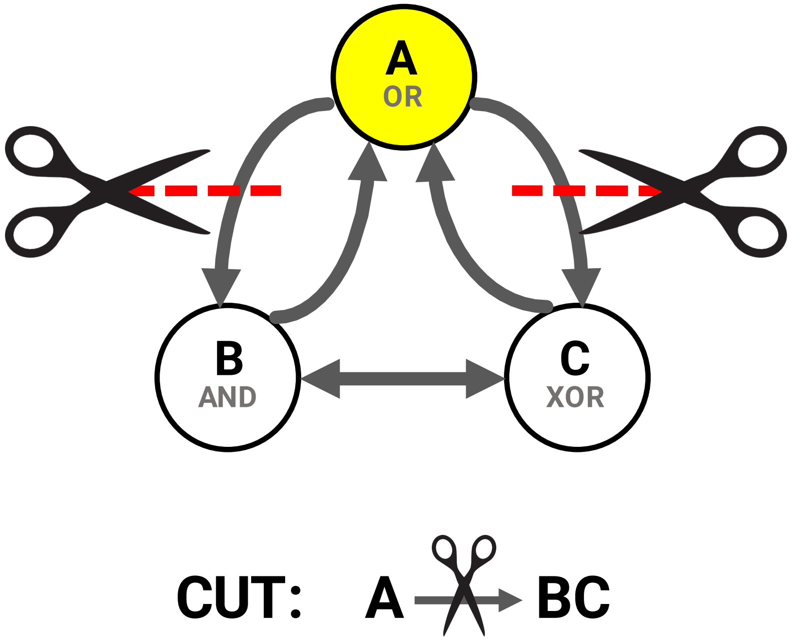

Integration and reducibility:
System-level irreducibility and system cuts

- We find the TPM for each individual mechanism, and combine them to get the full TPM (again, this works because of conditional independence)
- This makes the virtual elements implicit, as usual

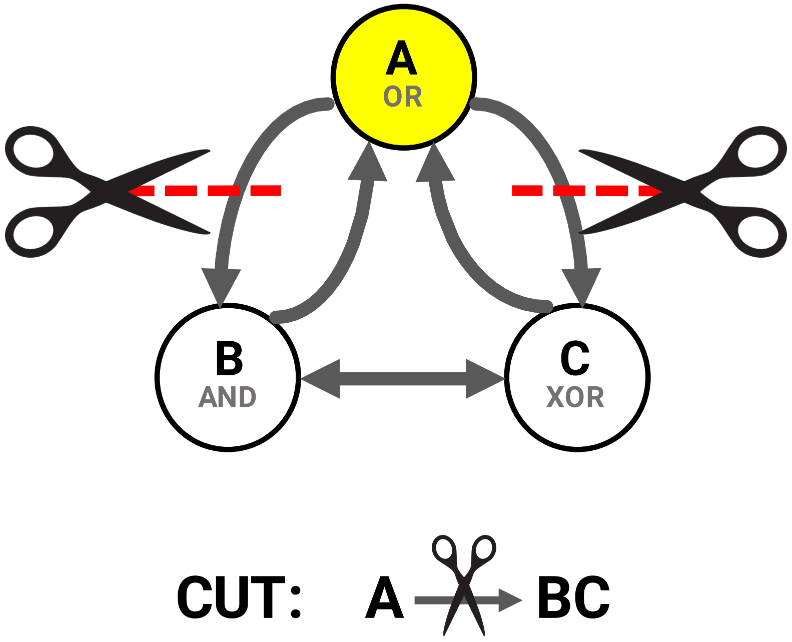

Integration and reducibility:

System-level irreducibility and system cuts

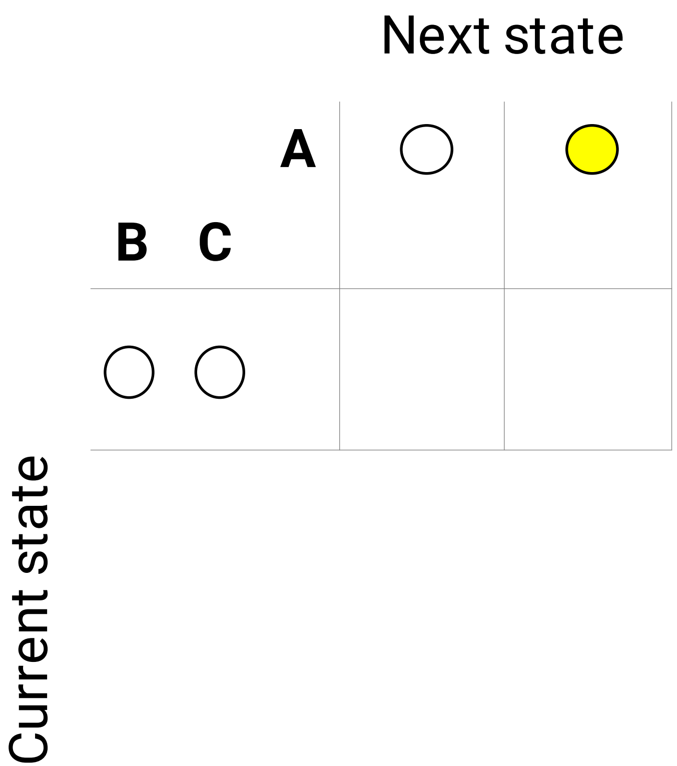

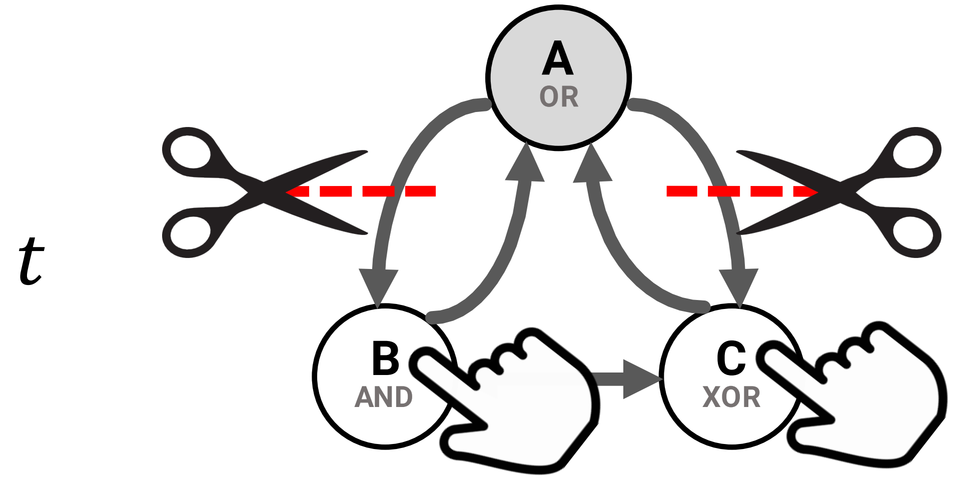

We start by finding the TPM for just **A**, which takes input from **B** and **C**

Integration and reducibility:

System-level irreducibility and system cuts

Next state

Current state

B	C	A: ○	A: ●
○	○	1	0

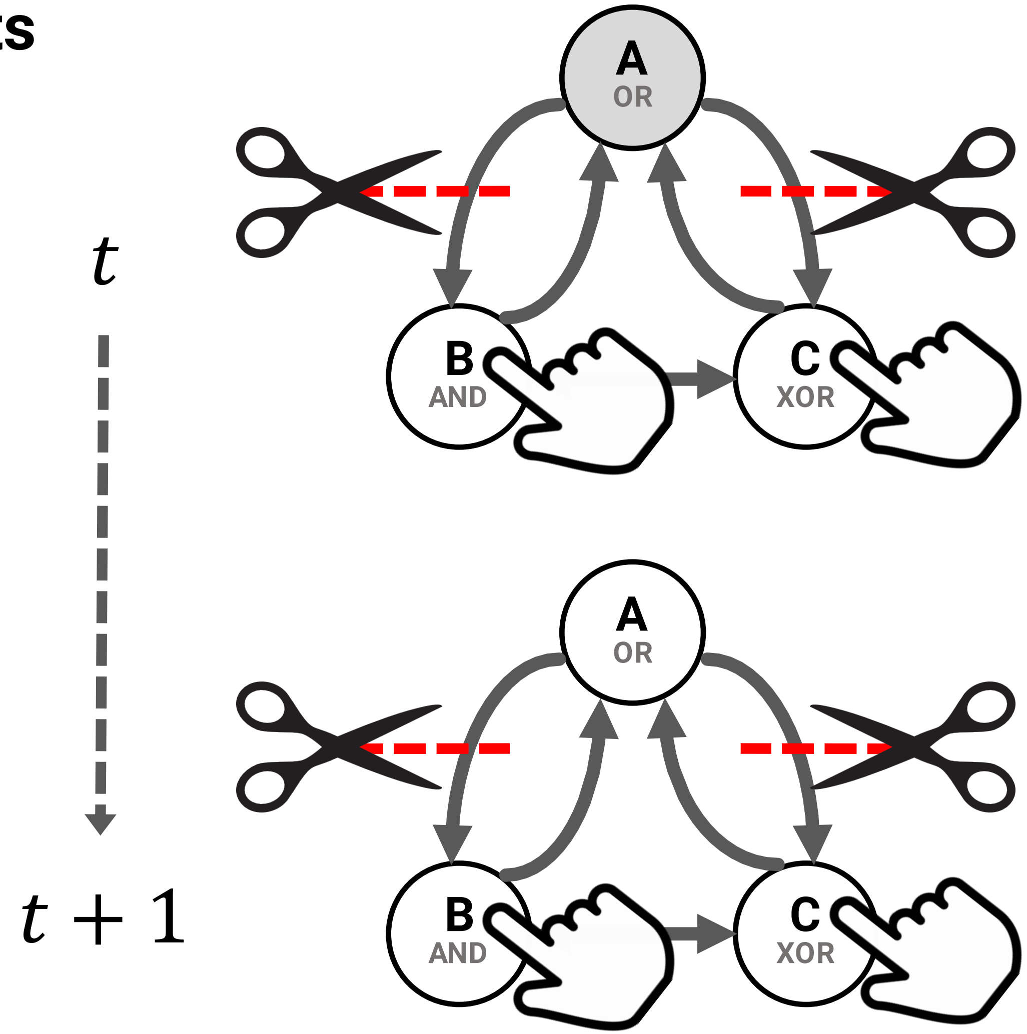

We start by finding the TPM for just **A**, which takes input from **B** and **C**

Integration and reducibility:

System-level irreducibility and system cuts

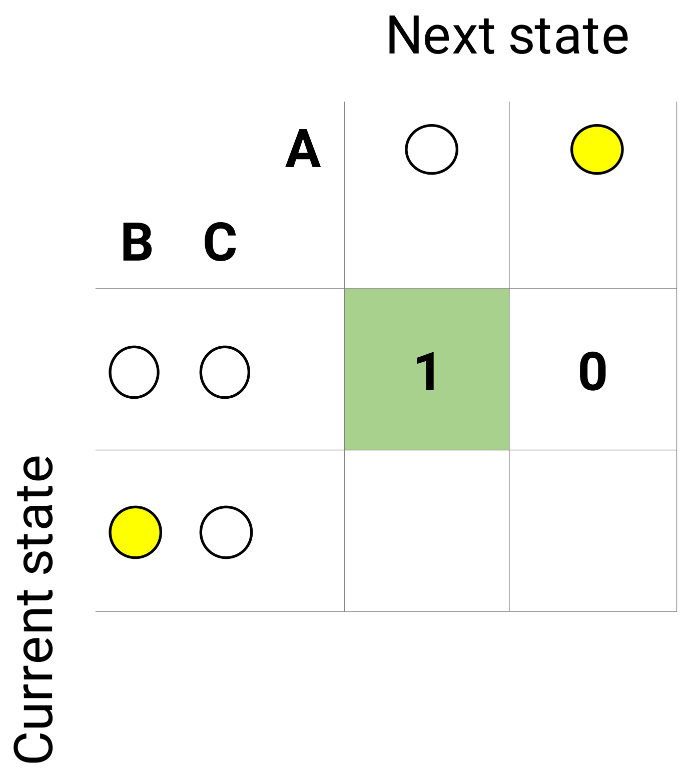

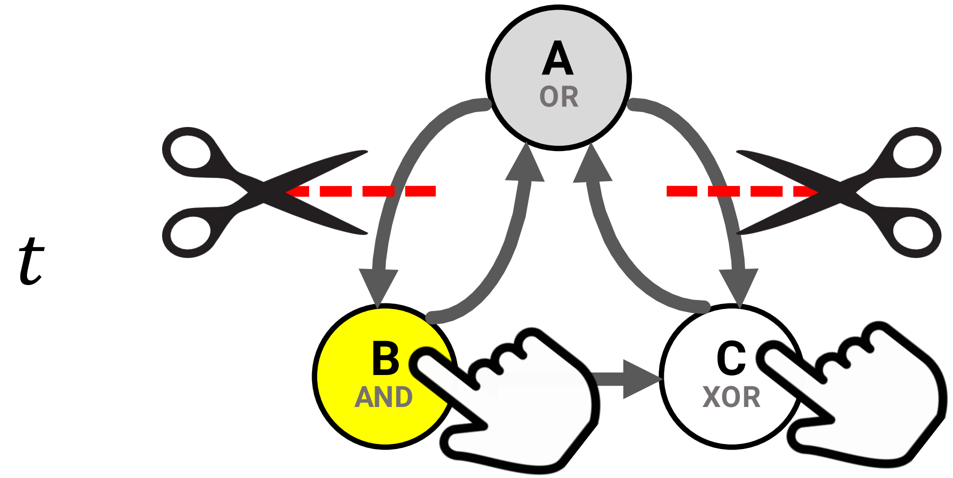

We start by finding the TPM for just **A**, which takes input from **B** and **C**

Integration and reducibility:

System-level irreducibility and system cuts

Next state

Current state

B C \ A	○	●
○ ○	1	0
● ○	0	1

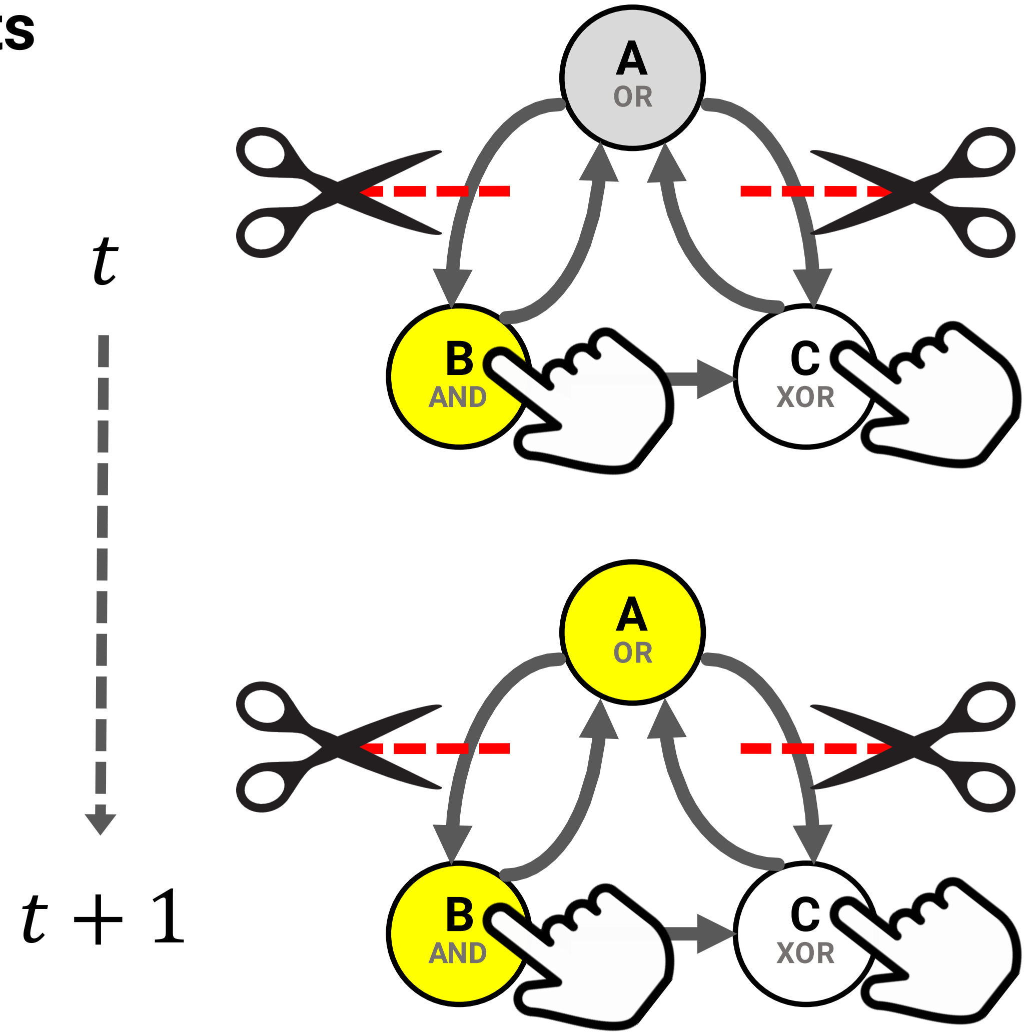

We start by finding the TPM for just **A**, which takes input from **B** and **C**

Integration and reducibility:

System-level irreducibility and system cuts

Next state

Current state

B	C	A ○	A ●
○	○	1	0
●	○	0	1
○	●		

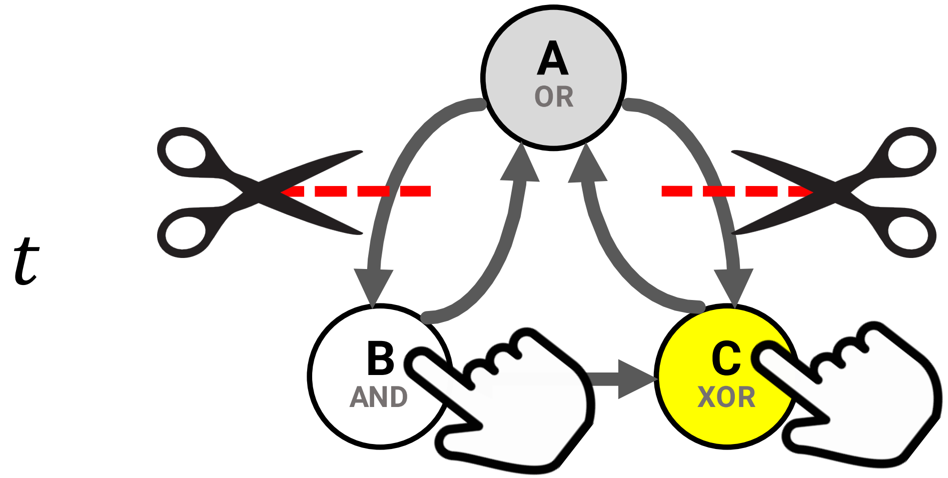

We start by finding the TPM for just **A**, which takes input from **B** and **C**

Integration and reducibility:

System-level irreducibility and system cuts

Next state

Current state

B	C	A: ○	A: ●
○	○	1	0
●	○	0	1
○	●	0	1

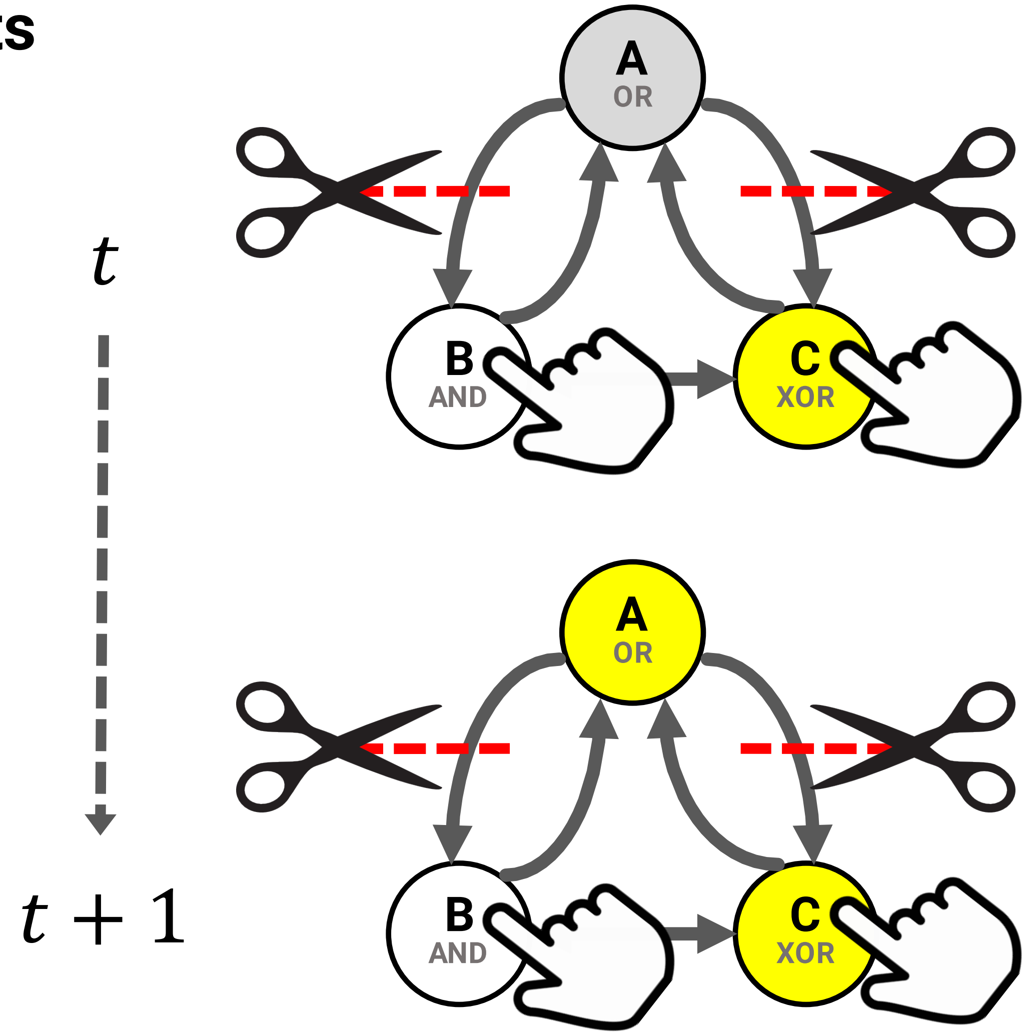

We start by finding the TPM for just **A**, which takes input from **B** and **C**

Integration and reducibility:

System-level irreducibility and system cuts

Next state

Current state: B	C	A: ○	A: ● (yellow)
○	○	1	0
●	○	0	1
○	●	0	1
●	●		

t

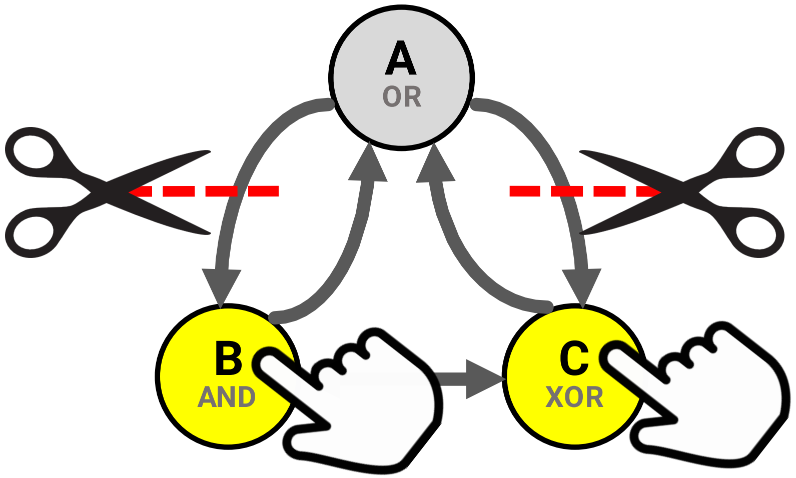

We start by finding the TPM for just **A**, which takes input from **B** and **C**

Integration and reducibility:

System-level irreducibility and system cuts

Next state

Current state B	Current state C	A ○	A ●
○	○	**1**	**0**
●	○	**0**	**1**
○	●	**0**	**1**
●	●	**0**	**1**

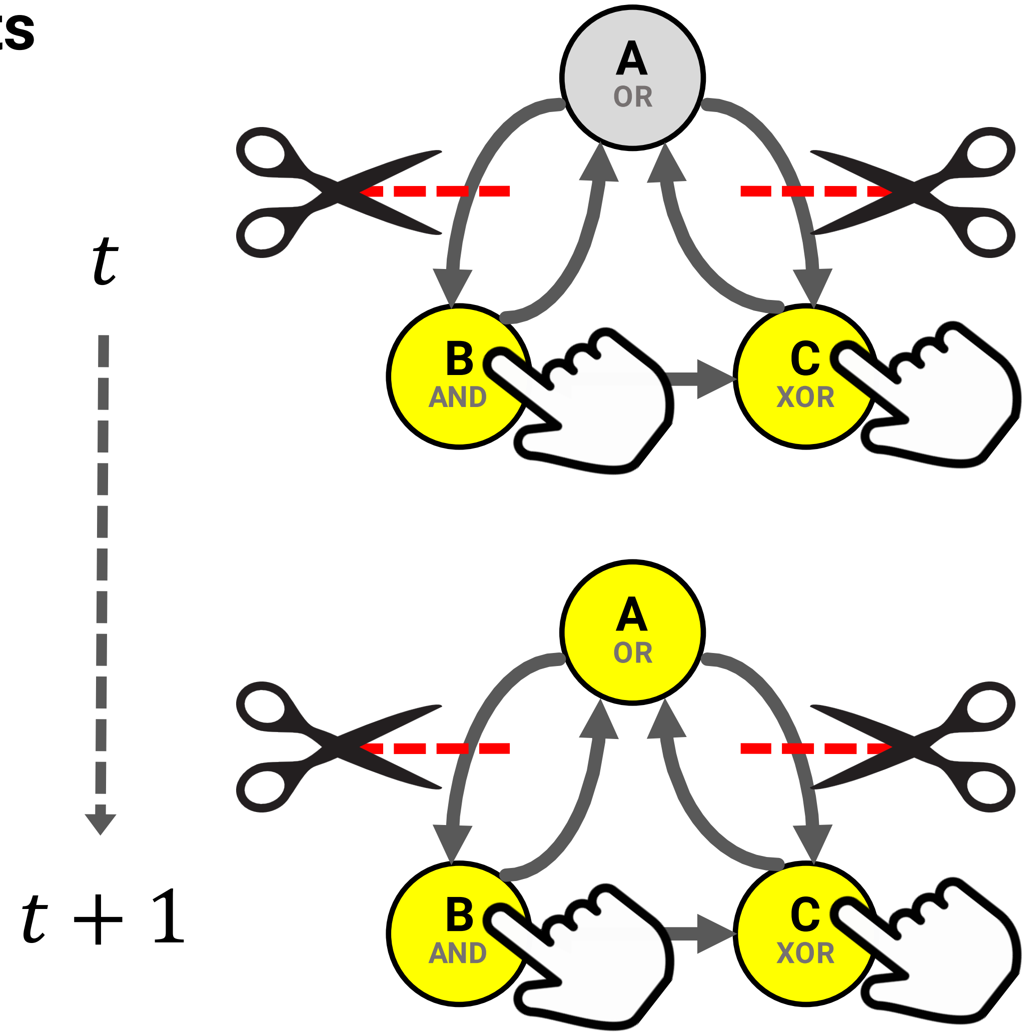

We start by finding the TPM for just **A**, which takes input from **B** and **C**

Integration and reducibility:
System-level irreducibility and system cuts

- Next we find the TPM for **B**, which takes input from **C** and *noised* input from **A**
- We account for **A**’s noisy output by computing the TPM as if the output were not noised, then marginalizing **A** out

Next state

Current state

A	C	B: ○	B: ●
○	○	1	0
●	○	1	0
○	●	1	0
●	●	0	1

Integration and reducibility:
System-level irreducibility and system cuts

- Next we find the TPM for **B**, which takes input from **C** and *noised* input from **A**
- We account for **A**'s noisy output by computing the TPM as if the output were not noised, then marginalizing **A** out

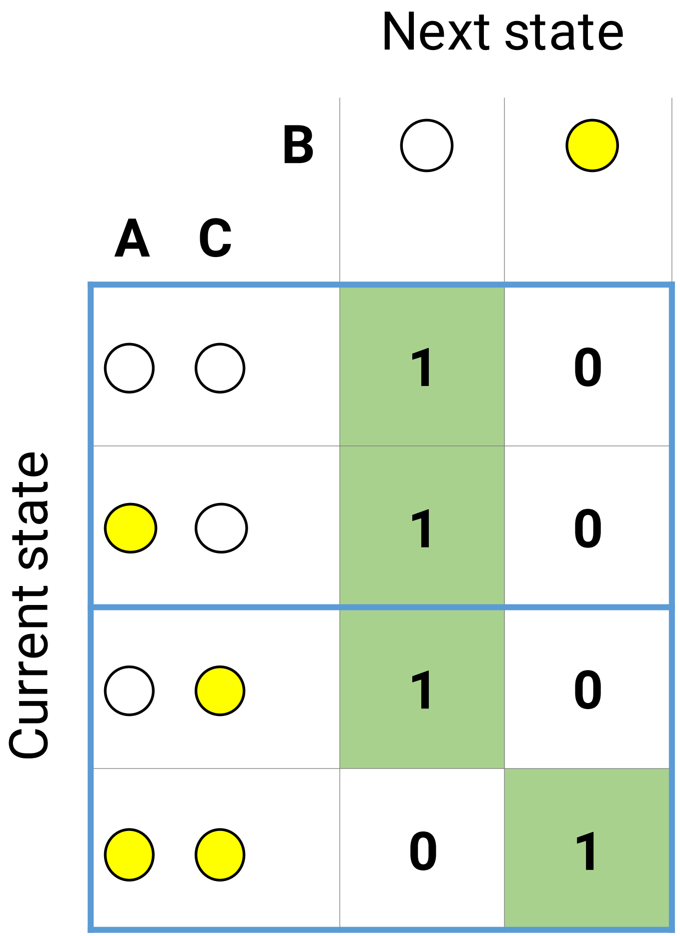

Integration and reducibility:
System-level irreducibility and system cuts

- Next we find the TPM for **B**, which takes input from **C** and *noised* input from **A**
- We account for **A**'s noisy output by computing the TPM as if the output were not noised, then marginalizing **A** out

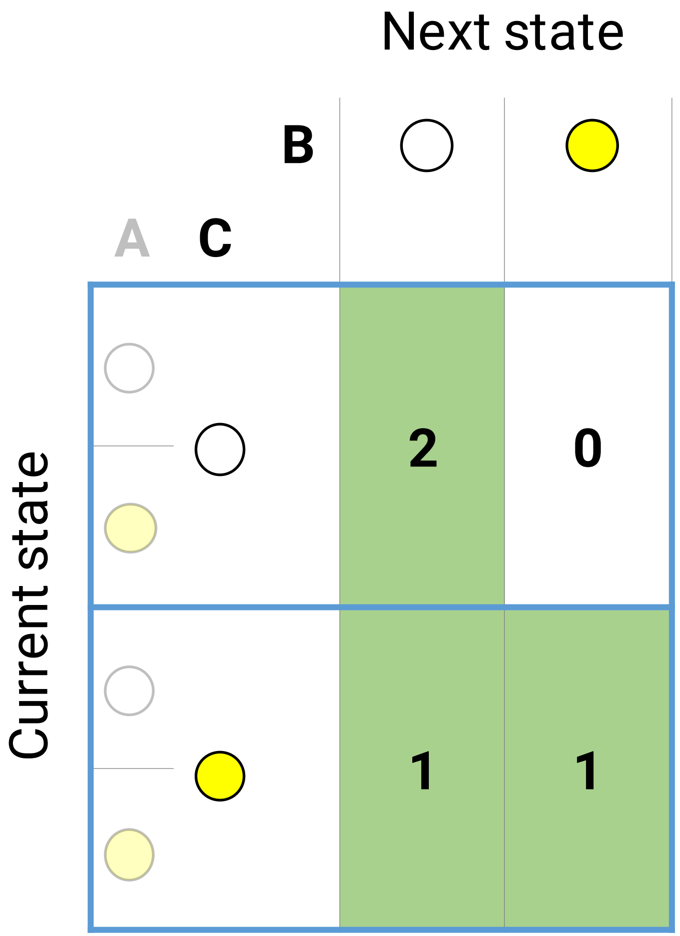

Integration and reducibility:
System-level irreducibility and system cuts

- Next we find the TPM for **B**, which takes input from **C** and *noised* input from **A**
- We account for **A**'s noisy output by computing the TPM as if the output were not noised, then marginalizing **A** out

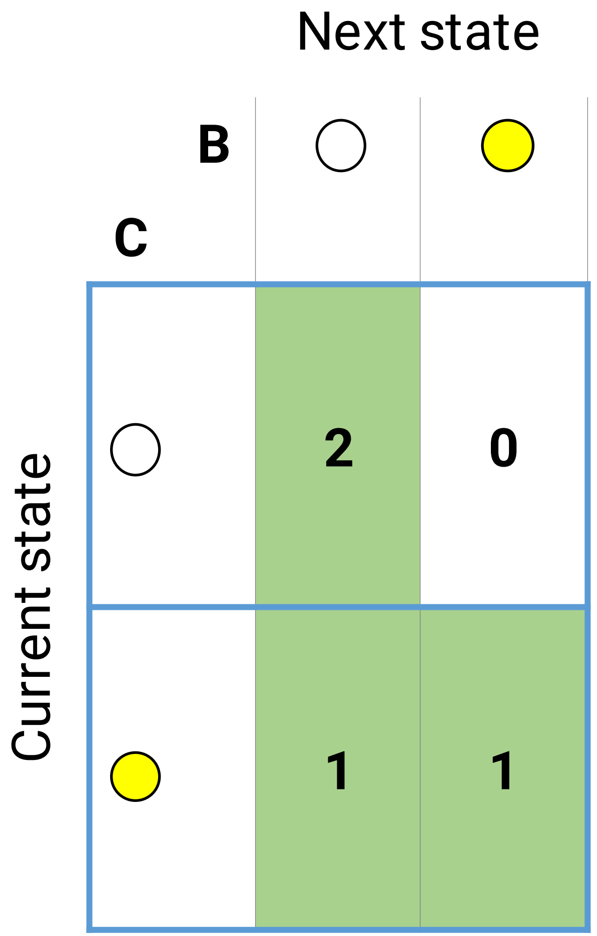

Integration and reducibility:

System-level irreducibility and system cuts

- Next we find the TPM for **B**, which takes input from **C** and *noised* input from **A**
- We account for **A**’s noisy output by computing the TPM as if the output were not noised, then marginalizing **A** out

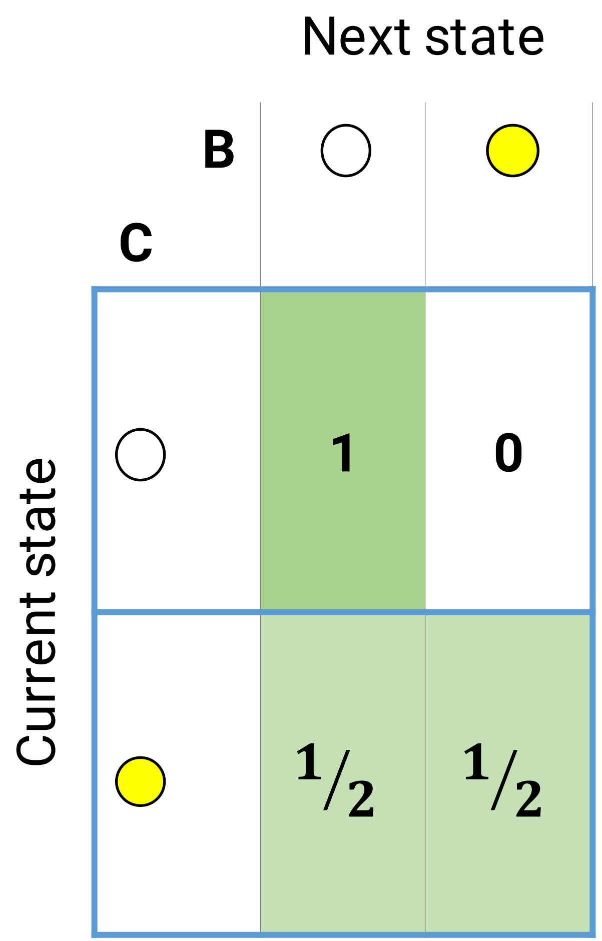

Integration and reducibility:

System-level irreducibility and system cuts

- Next we find the TPM for **B**, which takes input from **C** and *noised* input from **A**
- We account for **A**’s noisy output by computing the TPM as if the output were not noised, then marginalizing **A** out

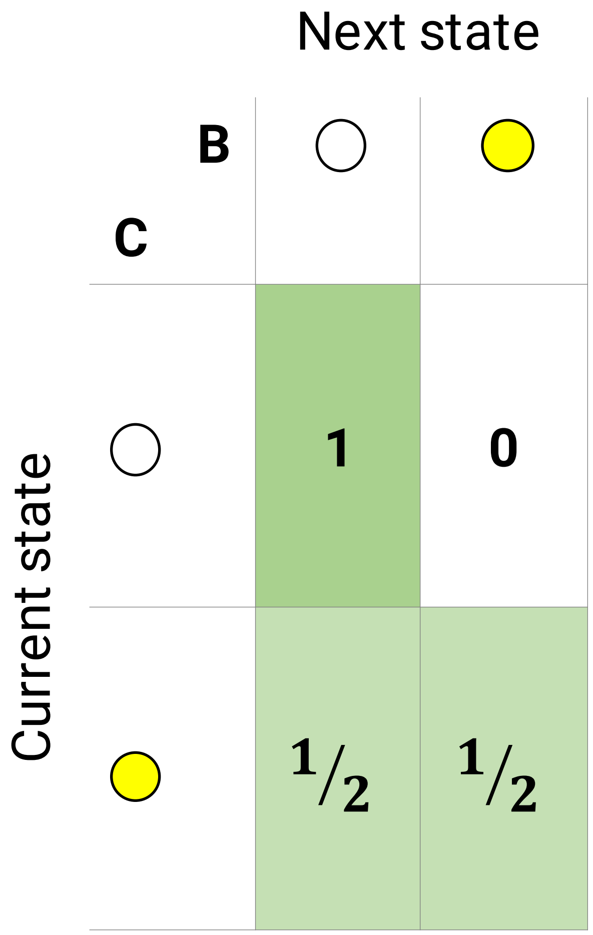

Integration and reducibility:

System-level irreducibility and system cuts

- Finally we do the same procedure for **C**, (which only gets input from B after the cut), which results in this TPM:

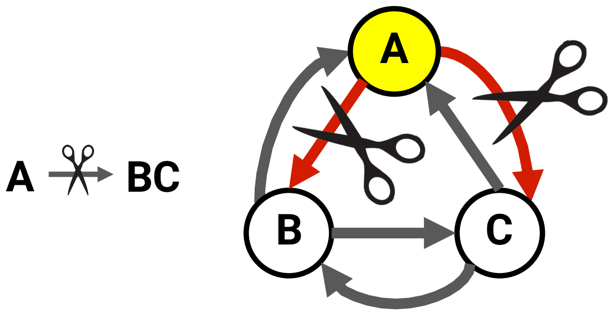

Next state

Current state

B \ C	○	●
○	$^1/_2$	$^1/_2$
●	$^1/_2$	$^1/_2$

Integration and reducibility:
System-level irreducibility and system cuts

Then we expand these TPMs to the full state space so they can be combined:

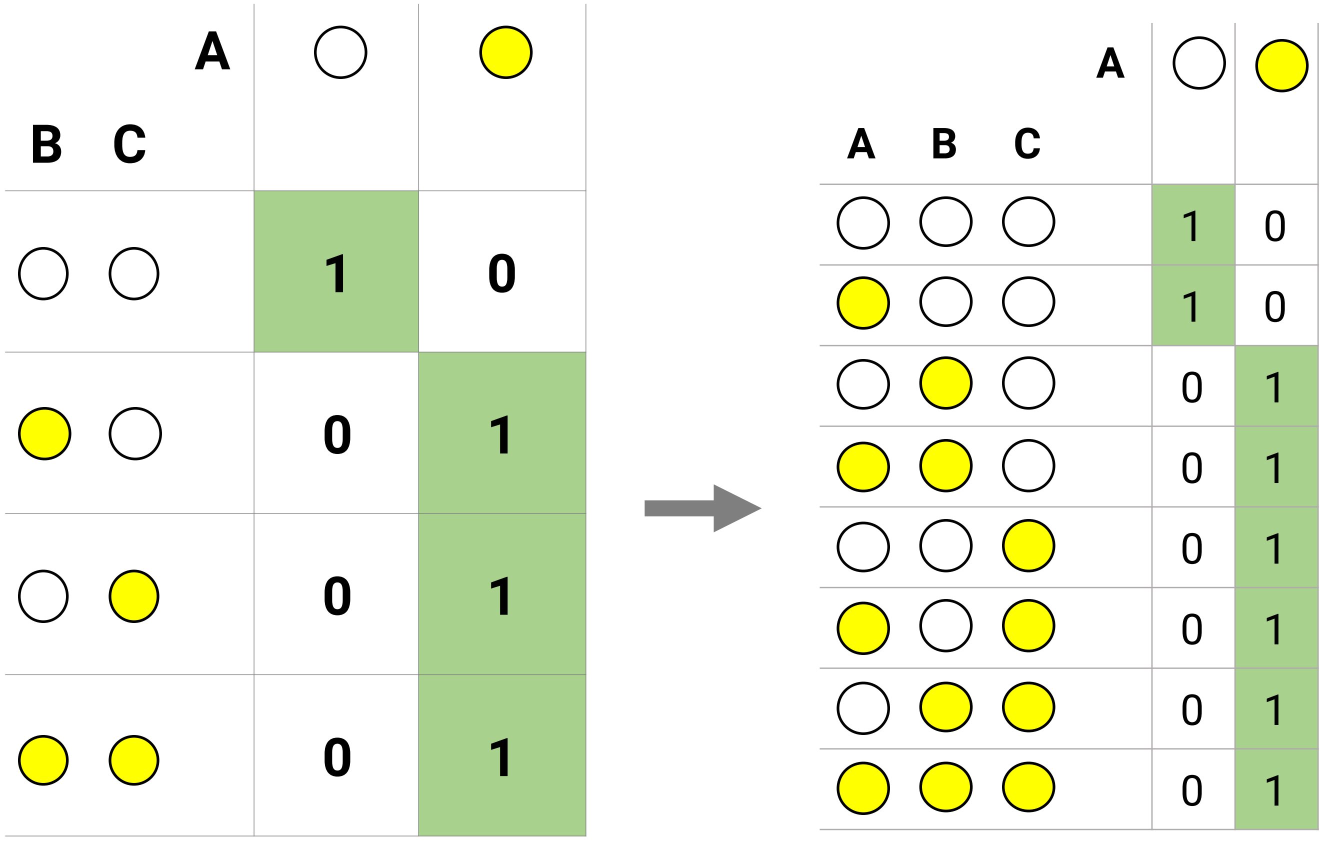

Integration and reducibility:

System-level irreducibility and system cuts

Then we expand these TPMs to the full state space so they can be combined:

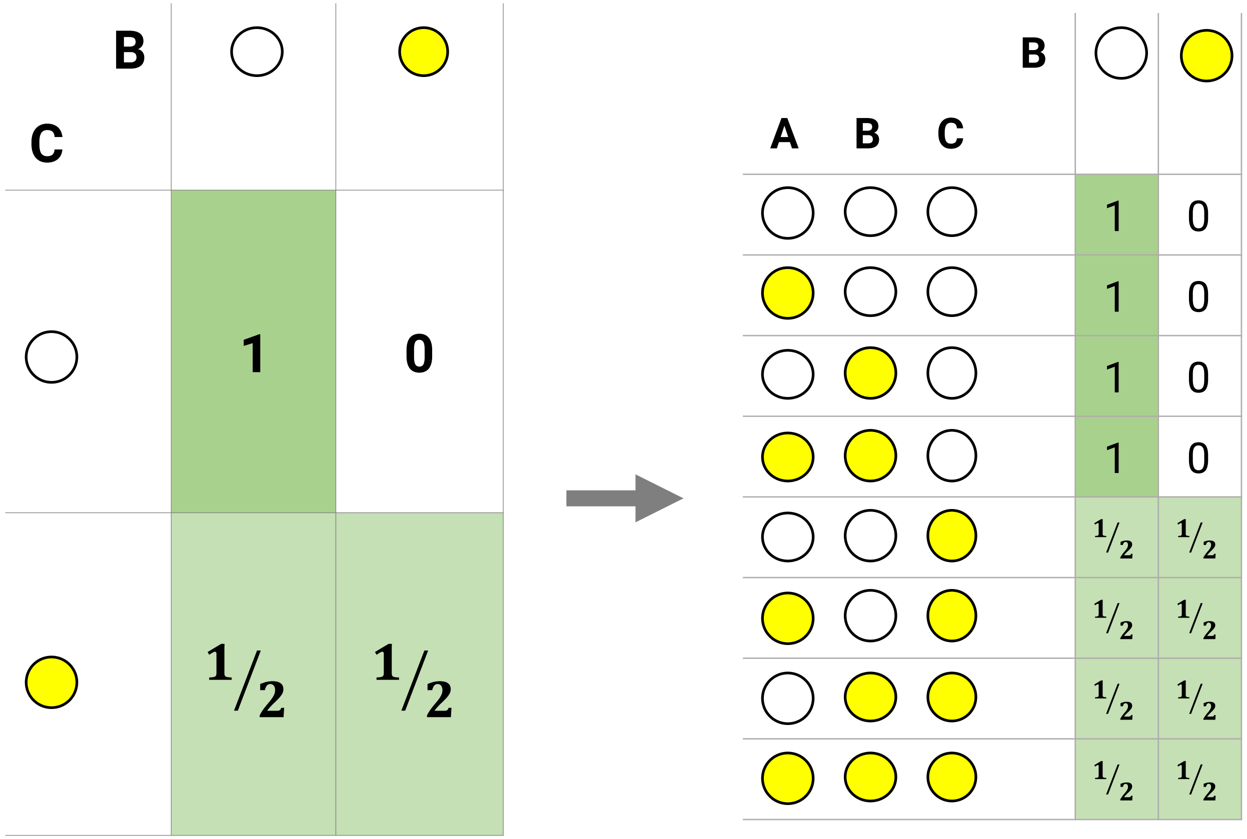

Integration and reducibility:

System-level irreducibility and system cuts

Then we expand these TPMs to the full state space so they can be combined:

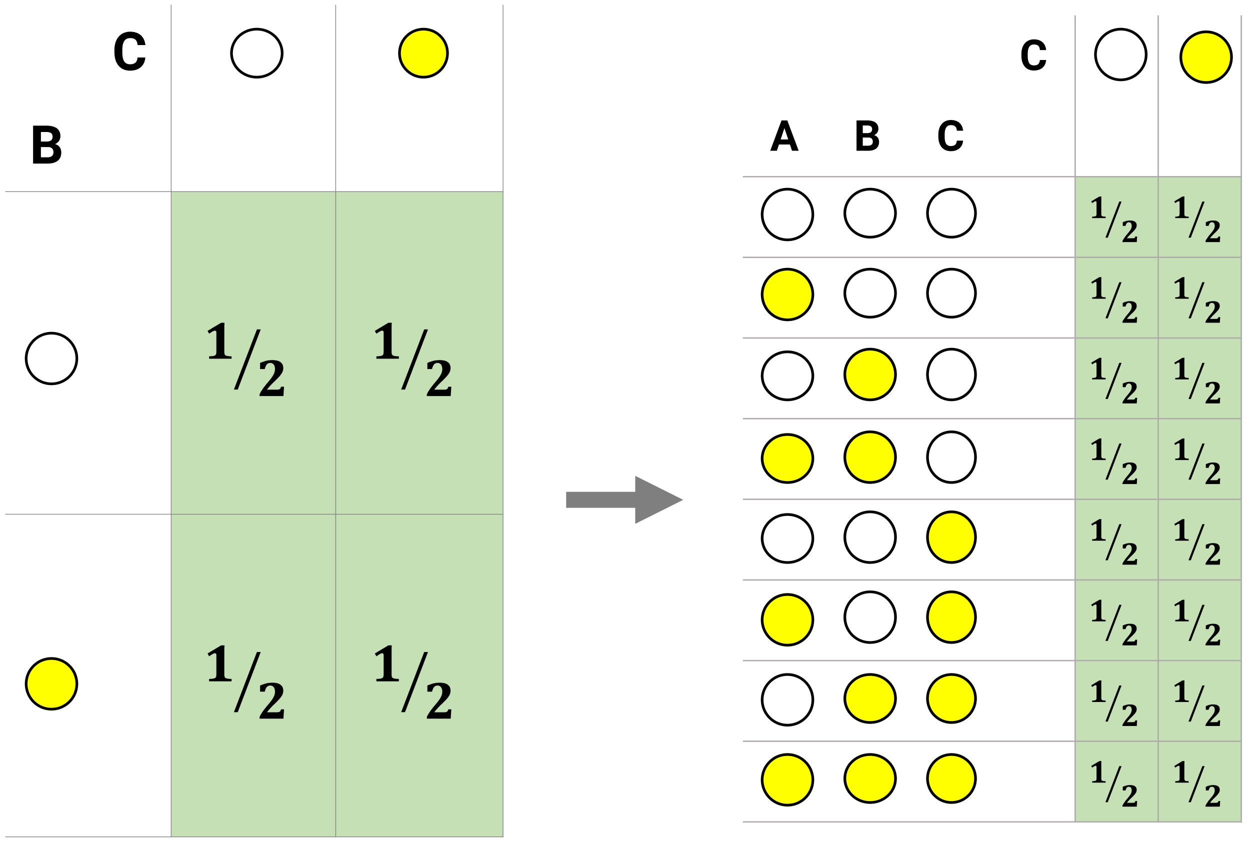

Integration and reducibility:

System-level irreducibility and system cuts

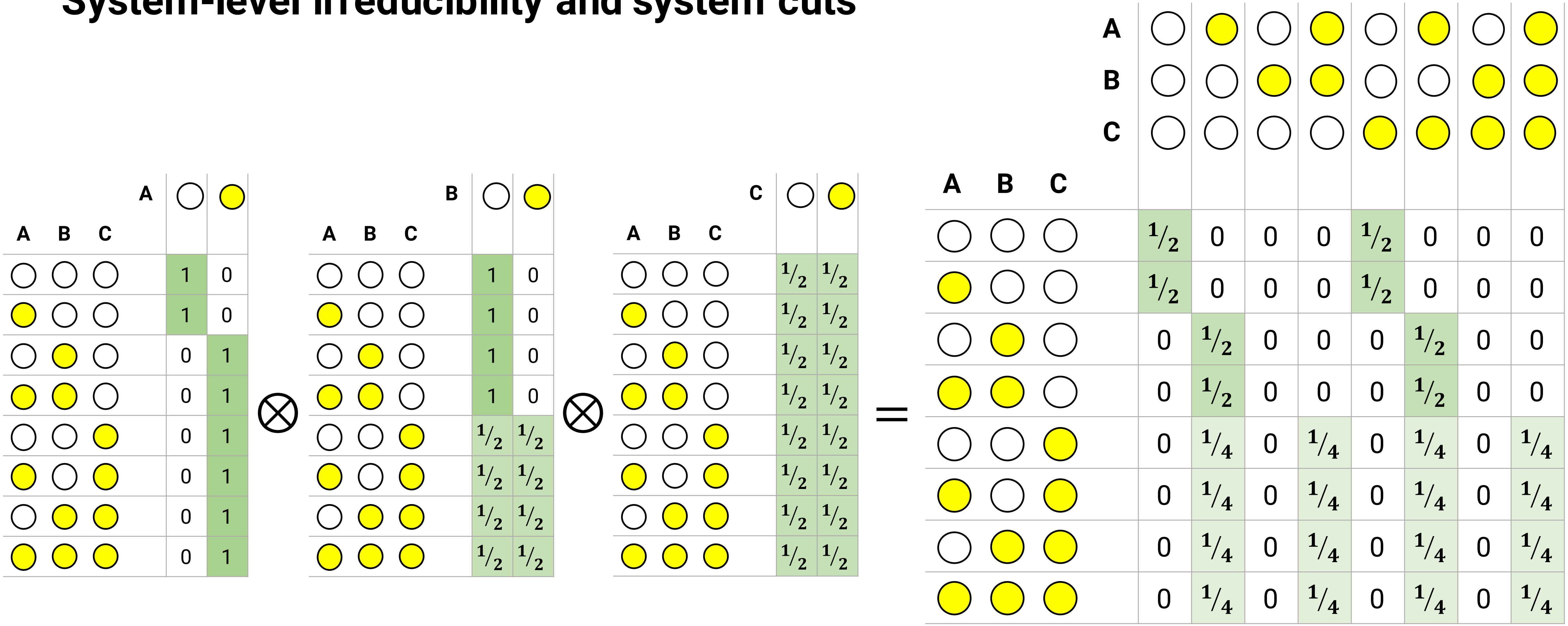

Now we can get the full TPM of the cut system by taking the tensor product of the individual TPMs

Integration and reducibility:

System-level irreducibility and system cuts

			A	○	●	○	●	○	●	○	●
			B	○	○	●	●	○	○	●	●
			C	○	○	○	○	●	●	●	●
A	B	C									
○	○	○		1	0	0	0	0	0	0	0
●	○	○		0	0	0	0	1	0	0	0
○	●	○		0	0	0	0	0	1	0	0
●	●	○		0	1	0	0	0	0	0	0
○	○	●		0	1	0	0	0	0	0	0
●	○	●		0	0	0	0	0	0	0	1
○	●	●		0	0	0	0	0	1	0	0
●	●	●		0	0	0	1	0	0	0	0

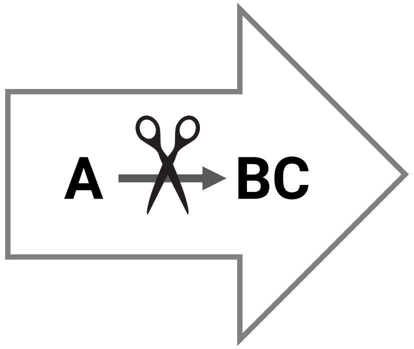

			A	○	●	○	●	○	●	○	●
			B	○	○	●	●	○	○	●	●
			C	○	○	○	○	●	●	●	●
A	B	C									
○	○	○		$^1/_2$	0	0	0	$^1/_2$	0	0	0
●	○	○		$^1/_2$	0	0	0	$^1/_2$	0	0	0
○	●	○		0	$^1/_2$	0	0	0	$^1/_2$	0	0
●	●	○		0	$^1/_2$	0	0	0	$^1/_2$	0	0
○	○	●		0	$^1/_4$	0	$^1/_4$	0	$^1/_4$	0	$^1/_4$
●	○	●		0	$^1/_4$	0	$^1/_4$	0	$^1/_4$	0	$^1/_4$
○	●	●		0	$^1/_4$	0	$^1/_4$	0	$^1/_4$	0	$^1/_4$
●	●	●		0	$^1/_4$	0	$^1/_4$	0	$^1/_4$	0	$^1/_4$

In sum: this is how the system cut **A** $\nRightarrow$ **BC** changes the TPM

Integration and reducibility:

System-level irreducibility and system cuts

- Now that we've recalculated the TPM, we can calculate the cut system's cause-effect structure
- We need to determine if the cut "makes a difference" from the intrinsic perspective of the system
- This will tell us whether the system reduces to the parts separated by the cut
- We do this by comparing the cause-effect structure of the uncut system to that of the cut system

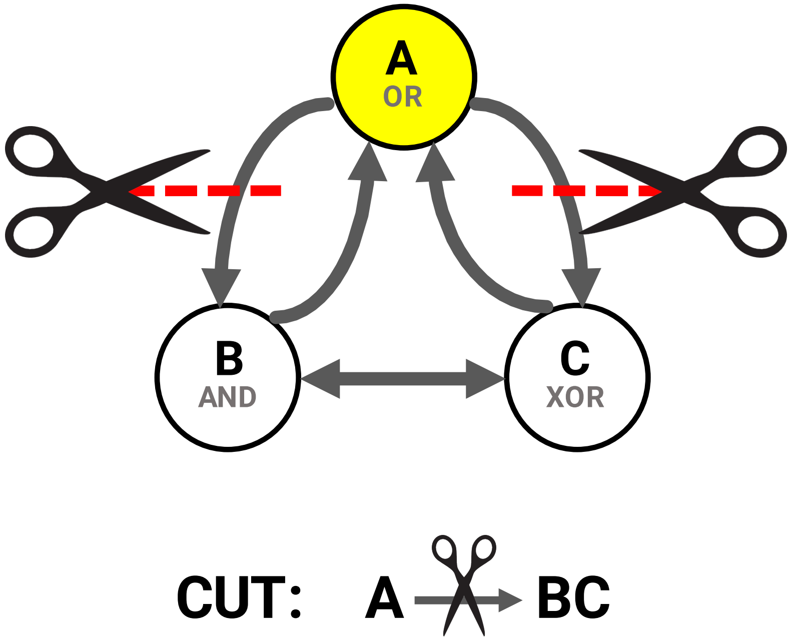

Integration and reducibility:

System-level irreducibility and system cuts

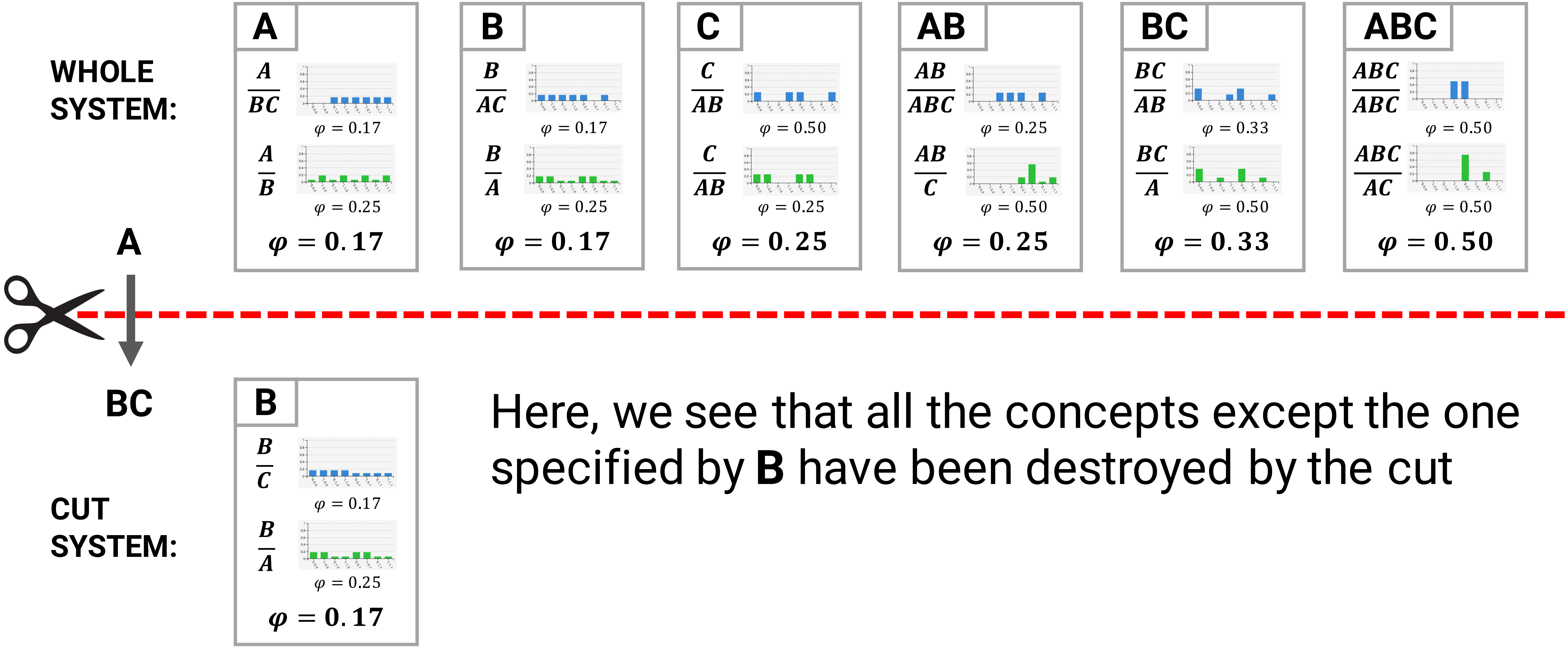

Here, we see that all the concepts except the one specified by **B** have been destroyed by the cut

Integration and reducibility:

Extended earth mover's distance

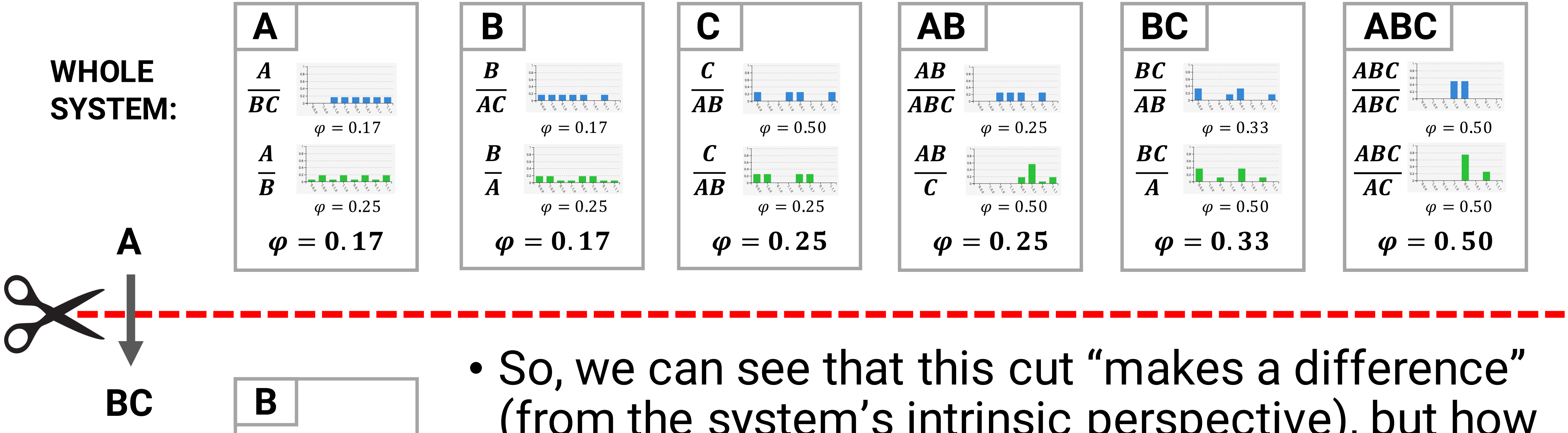

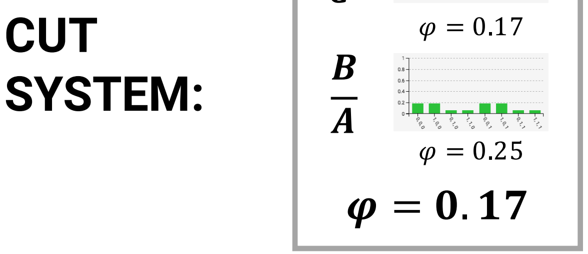

- So, we can see that this cut "makes a difference" (from the system's intrinsic perspective), but how do we quantify that difference?
- As with calculating the φ of a repertoire, we can use the Earth Mover's Distance to measure the difference between the unpartitioned and partitioned cause-effect structures

Integration and reducibility:

Extended earth mover's distance

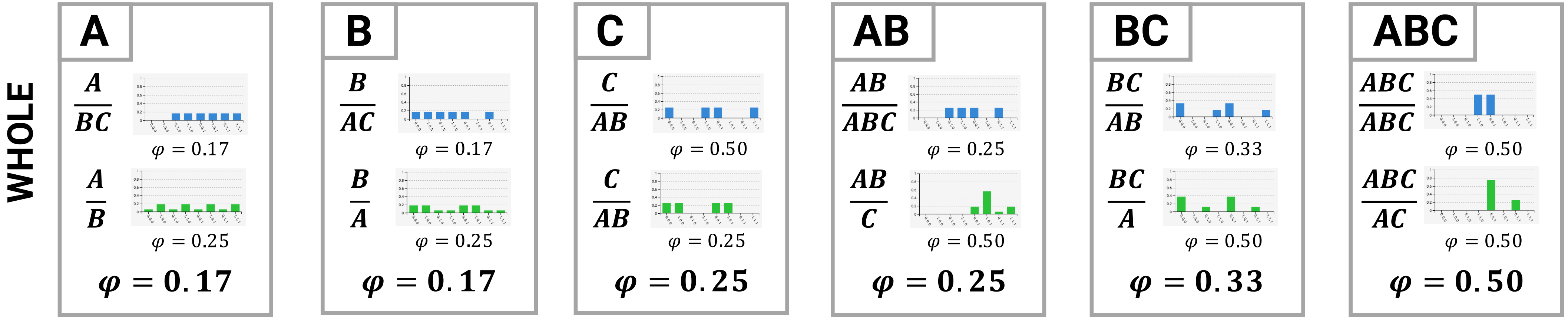

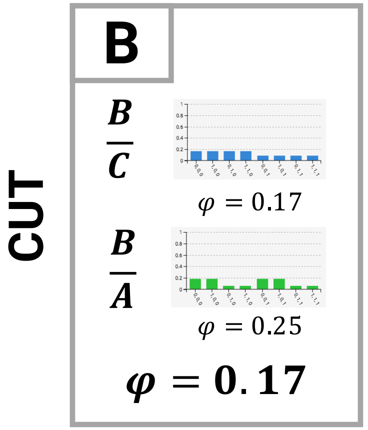

- In this case, the "earth" that we're moving is the φ-value of each concept
- The cost of transporting φ from one concept to another is the **concept distance**
- This is the sum of the EMD between their cause repertoires and the EMD between their effect repertoires

Integration and reducibility:

Extended earth mover's distance

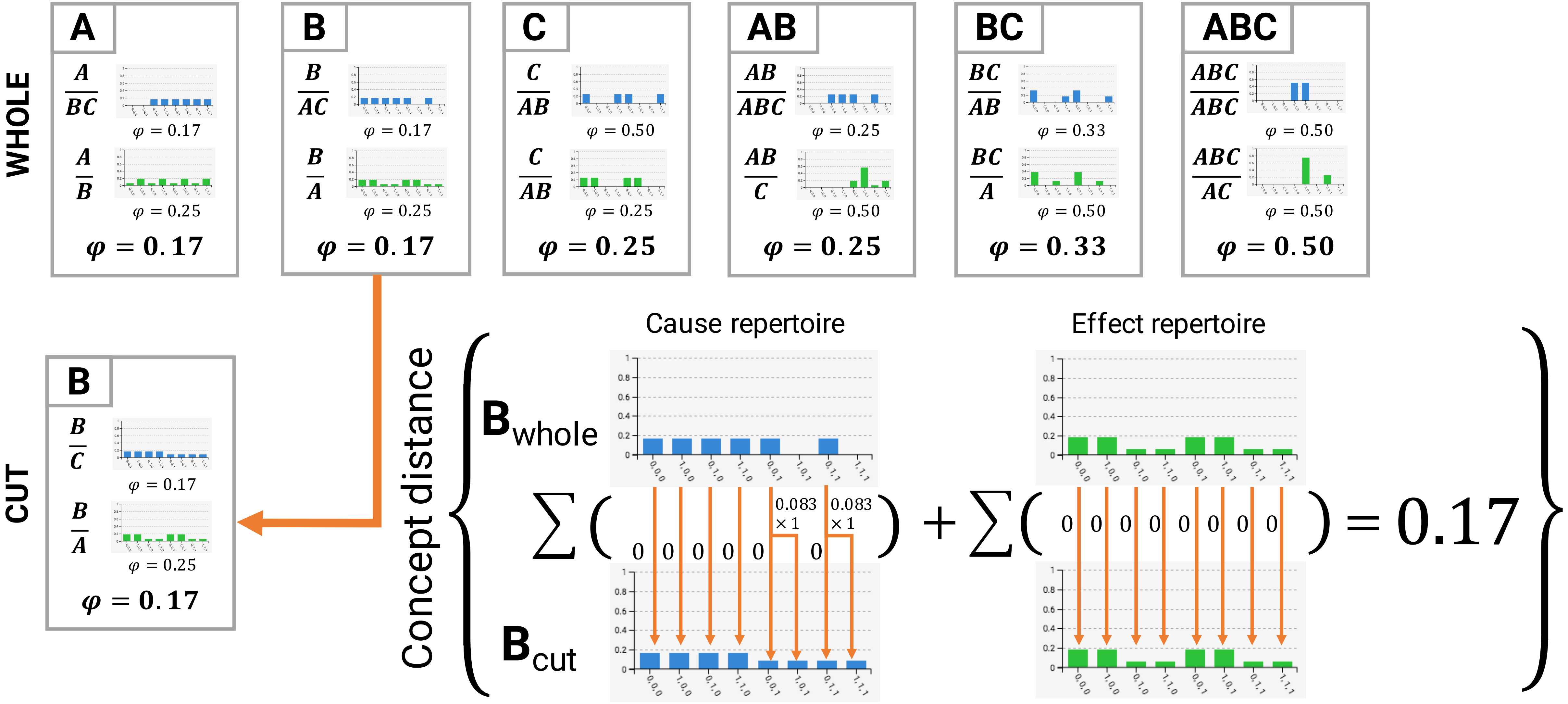

Integration and reducibility:

Extended earth mover's distance

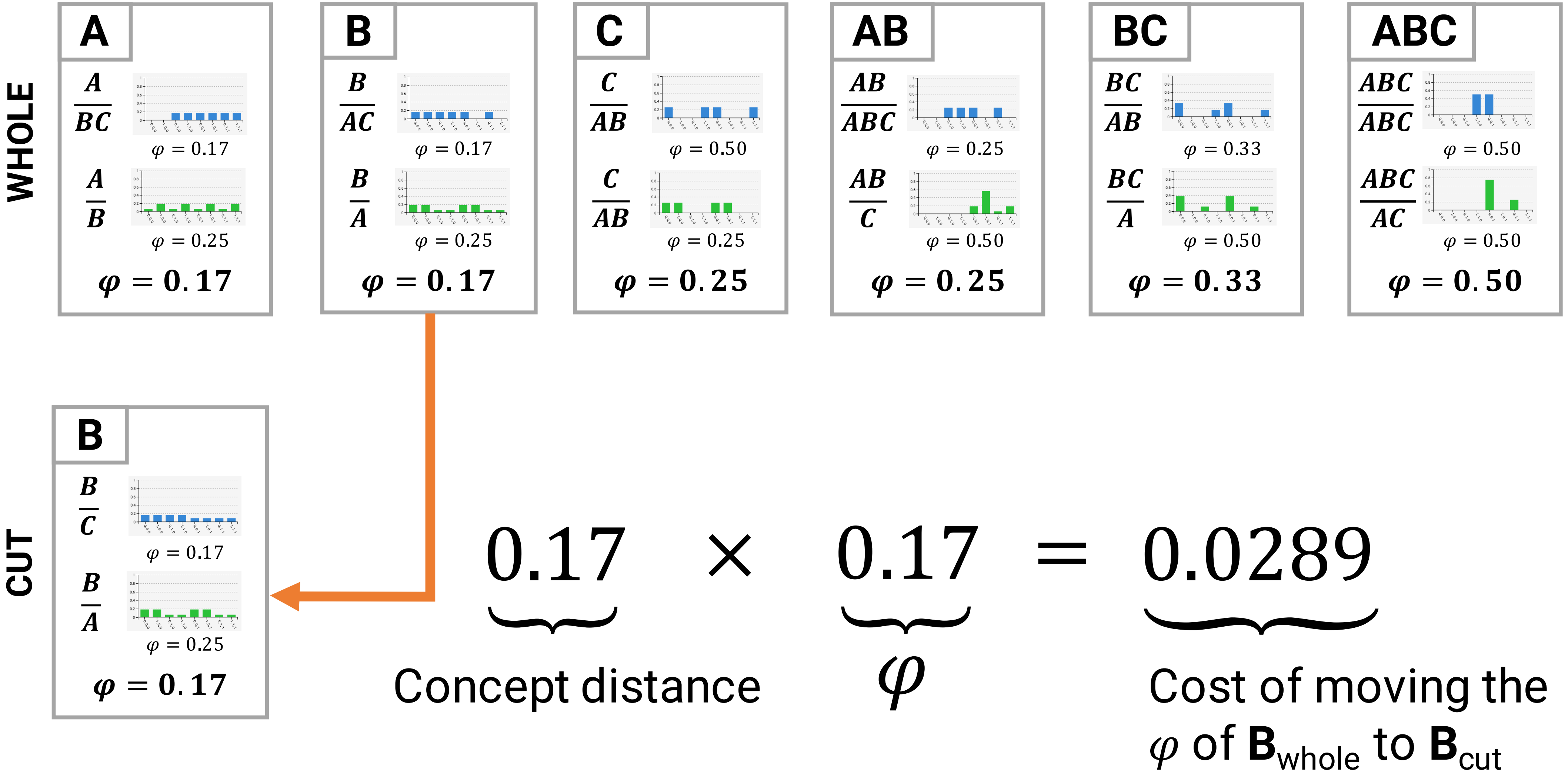

Integration and reducibility:

Extended earth mover's distance

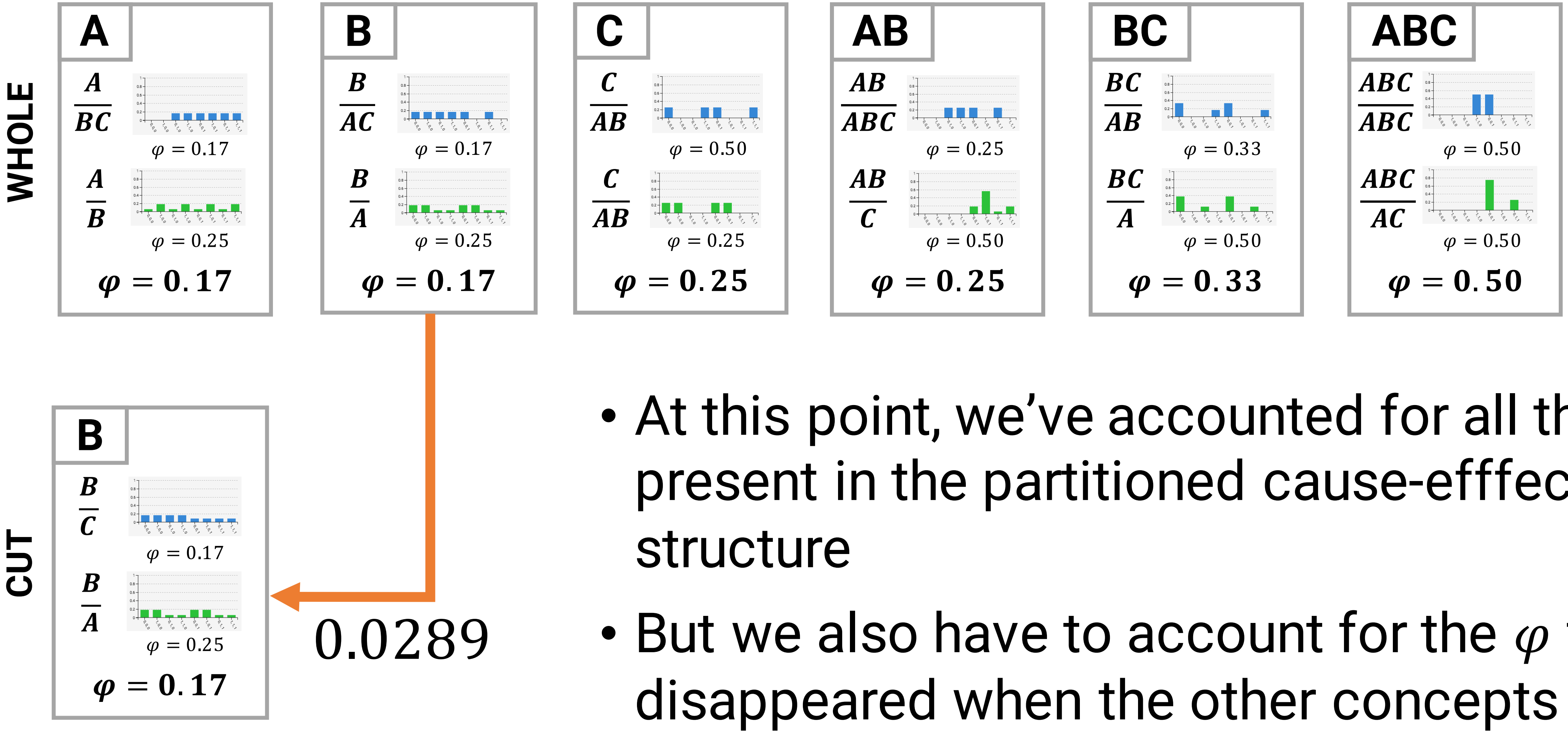

- At this point, we've accounted for all the φ present in the partitioned cause-efffect structure
- But we also have to account for the φ that disappeared when the other concepts were destroyed

Integration and reducibility:
Extended earth mover’s distance

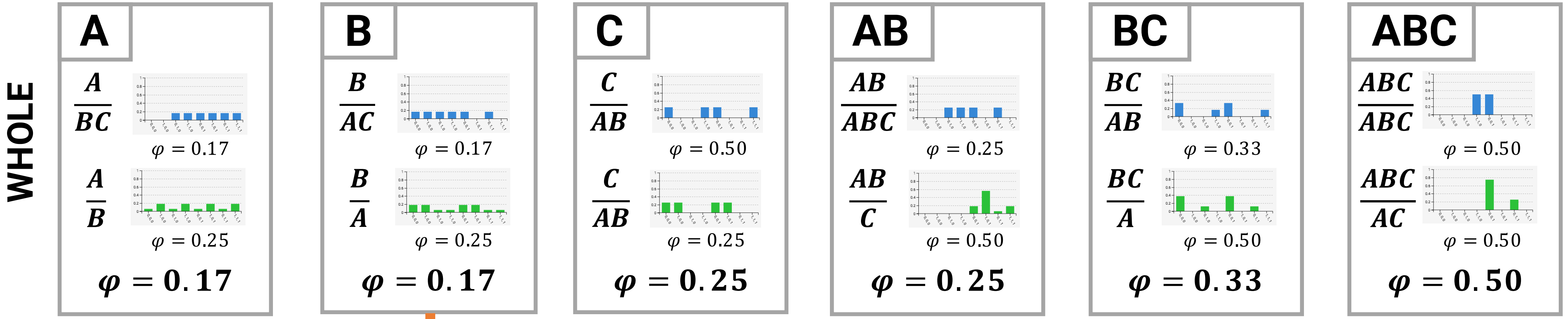

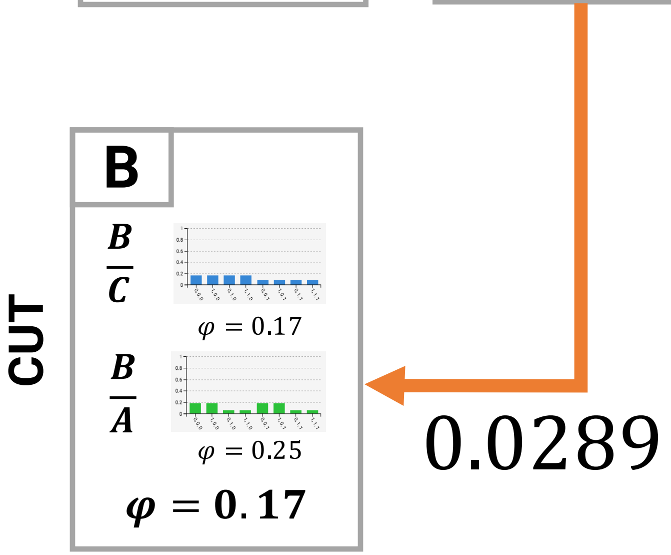

- To do this, we transport all the “extra” φ to the **null concept**
- This is the concept that is specified by no mechanism (strictly speaking, it’s not a concept since it has φ = 0)

Integration and reducibility:

Extended earth mover's distance

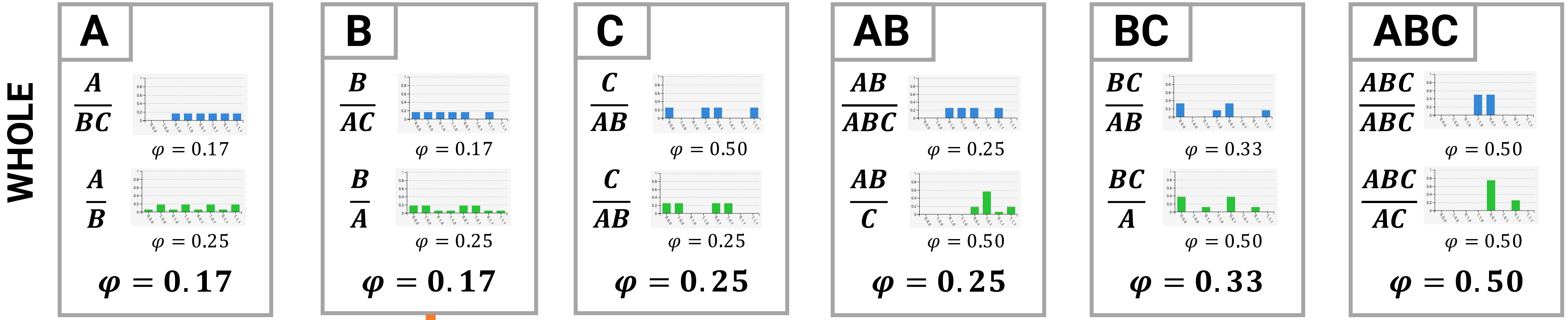

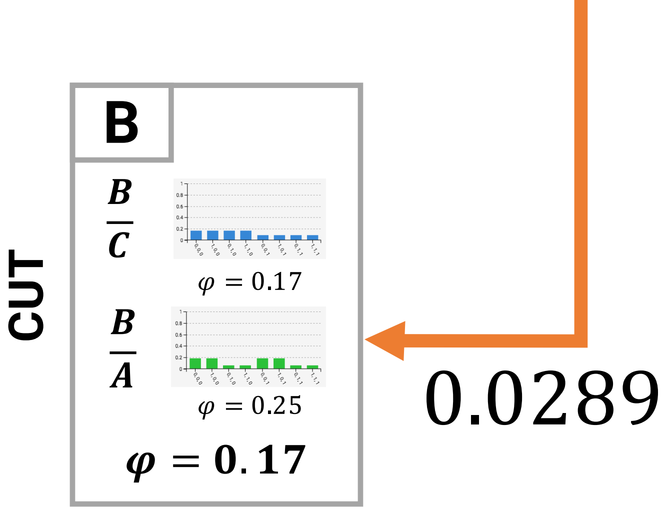

- Since the null concept's mechanism is empty, its cause and effect repertoires are simply the unconstrained repertoires over the entire system
- So, the distance to the null concept is the sum of the distances to the unconstrained cause and effect repertoires

Integration and reducibility:

Extended earth mover's distance

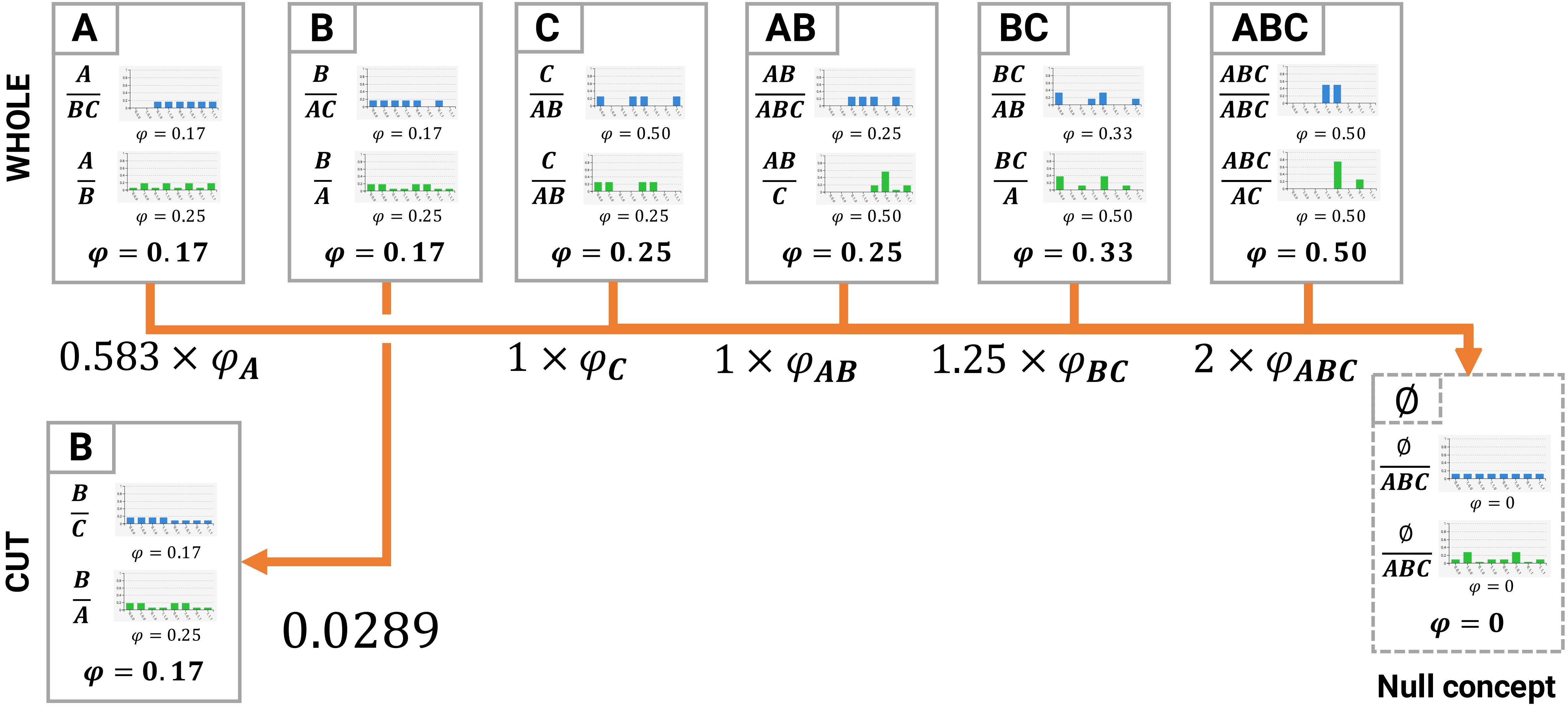

Integration and reducibility:

Extended earth mover's distance

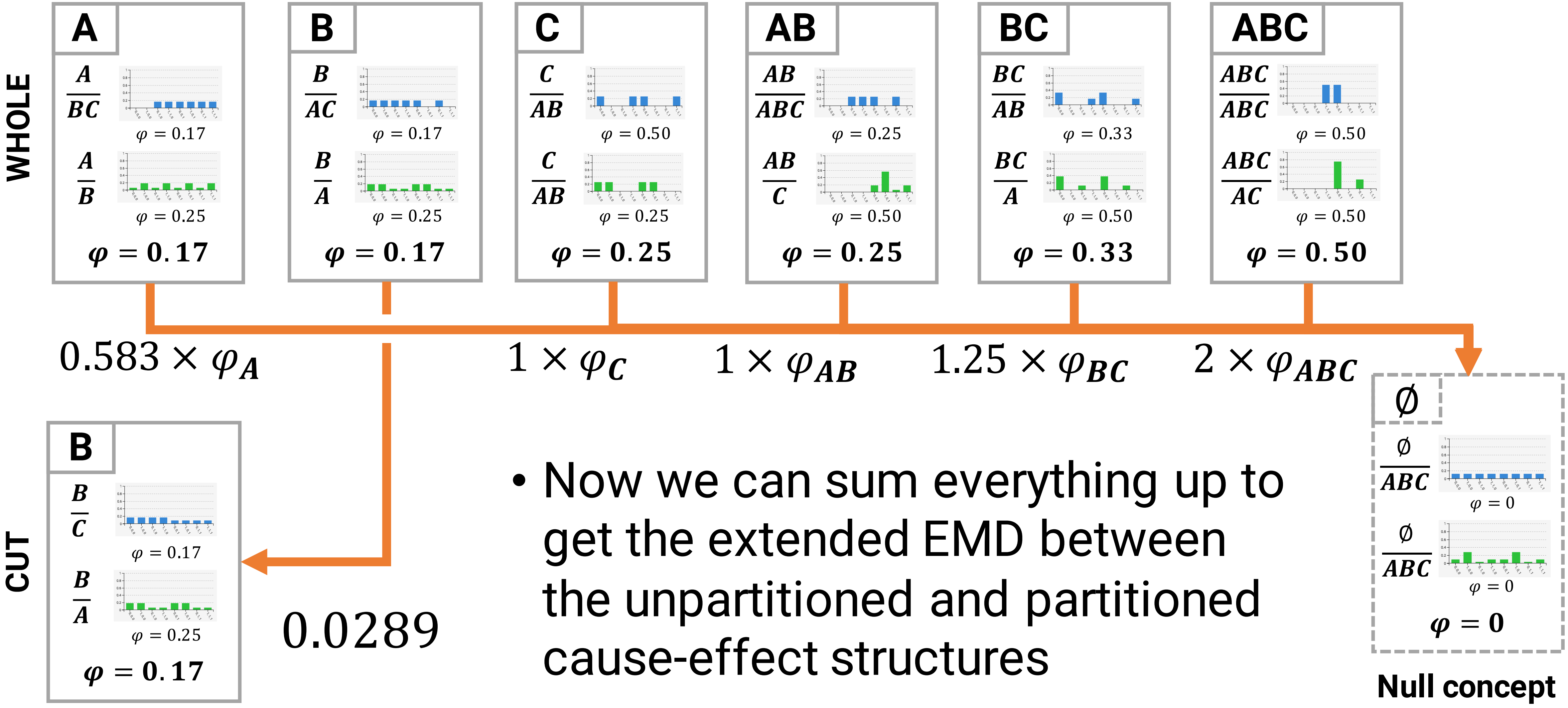

- Now we can sum everything up to get the extended EMD between the unpartitioned and partitioned cause-effect structures

Integration and reducibility:

Extended earth mover's distance

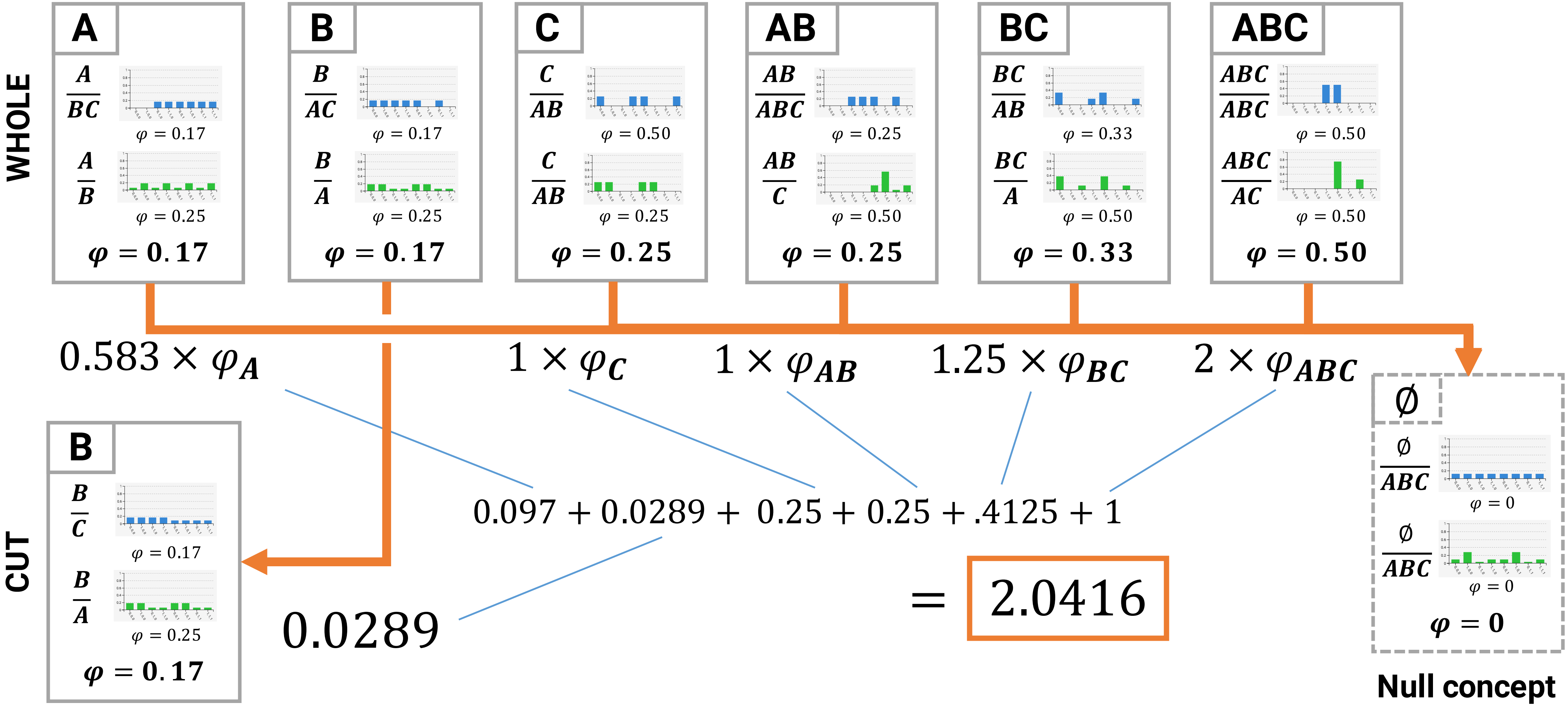

Integration and reducibility:

Extended earth mover’s distance

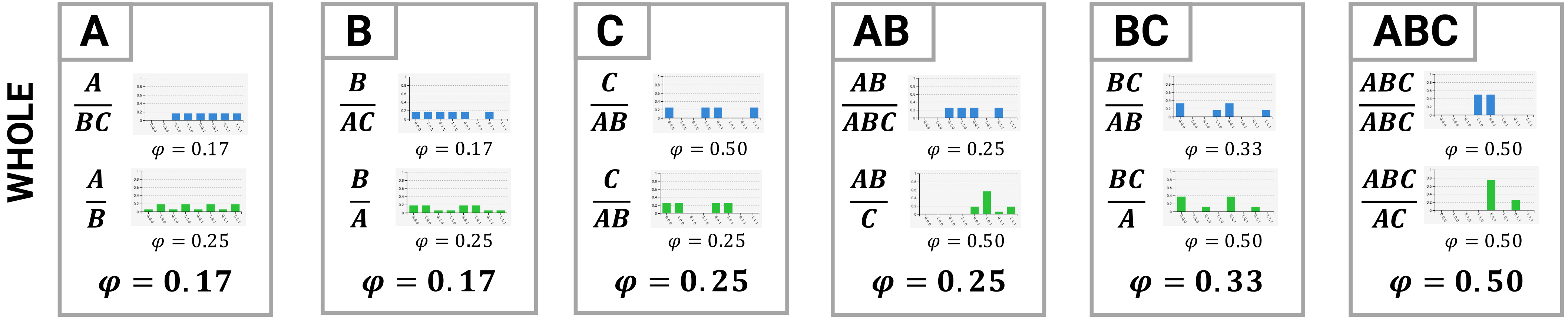

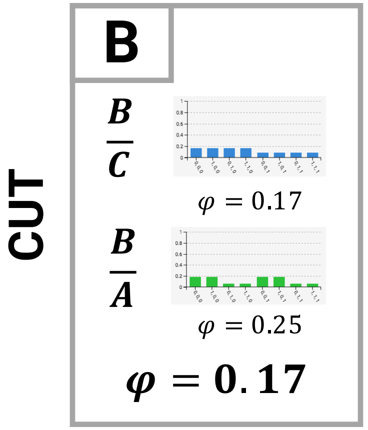

- This quantity is called **integrated conceptual information**, and is denoted $\boldsymbol{\Phi}$ (“big-phi”)
- It captures how irreducible the cause-effect structure of the system is, with respect to this particular cut

Integration and reducibility:

System-level minimum information partition

- However, as with partitioning mechanisms, there are different ways to cut the system in two:

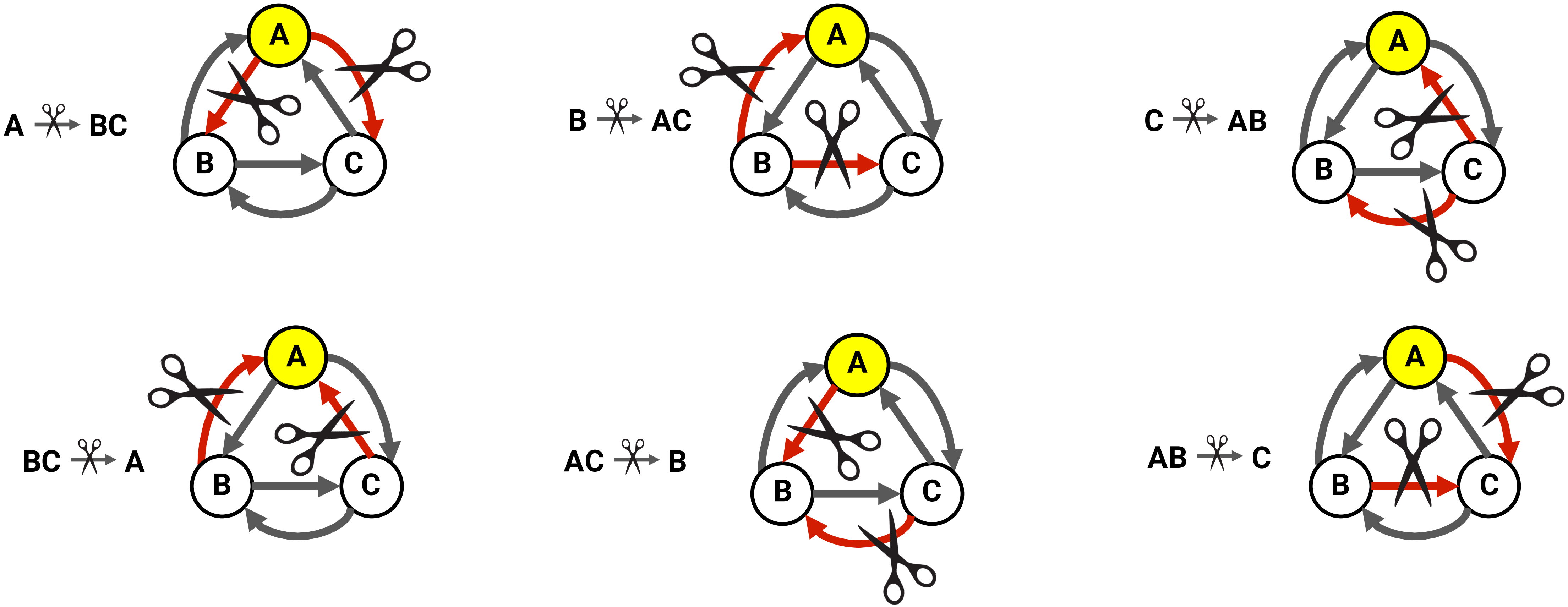

Integration and reducibility:
System-level minimum information partition

- So, we perform every possible cut, determine the cause-effect structure for each of the cut systems, and calculate the Φ-value associated with each
- The cut that yields the minimal Φ-value is again called the **minimum information partition** (MIP) for the system

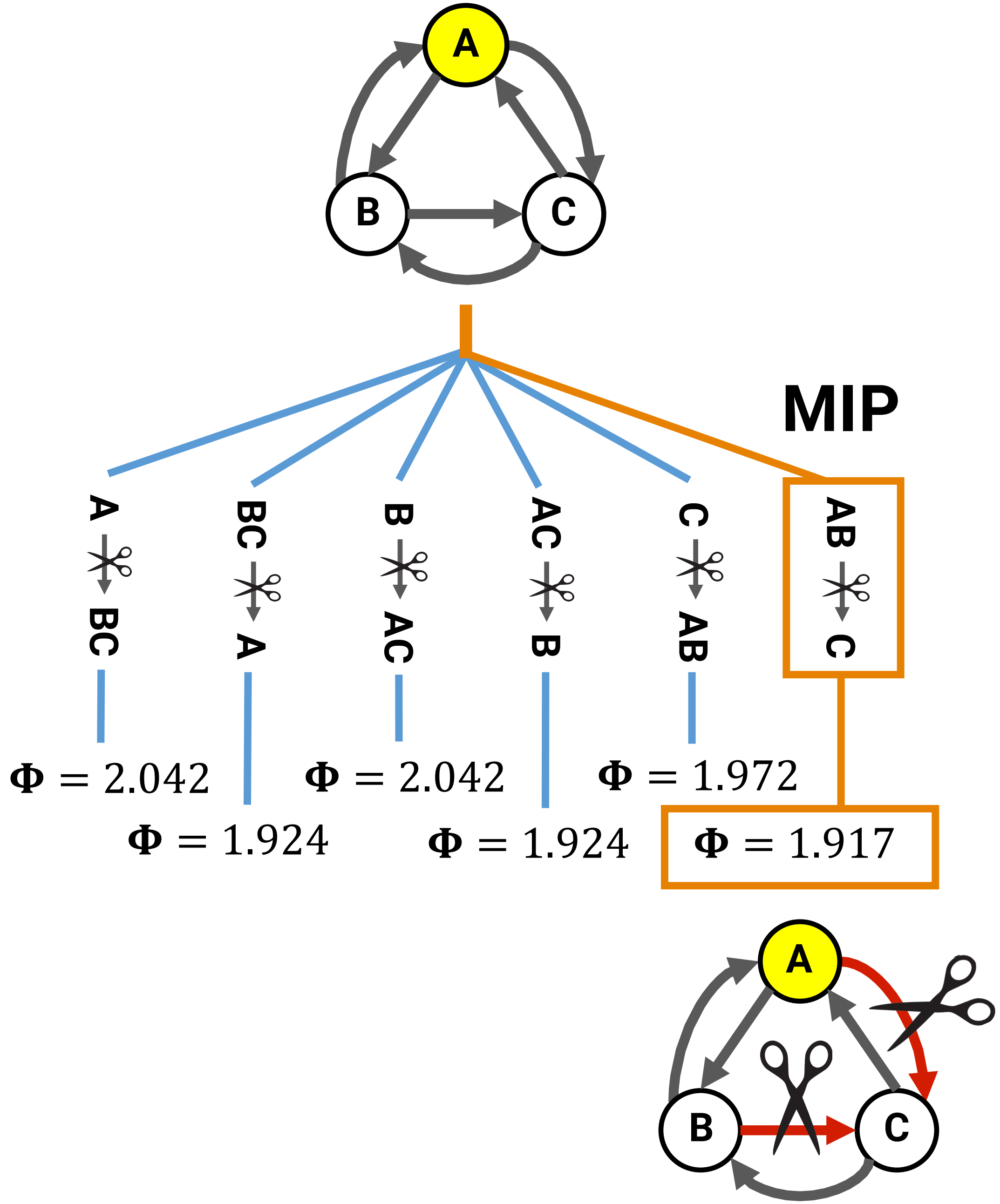

Integration and reducibility:
Integrated information

- The minimal Φ-value, Φ^{MIP}, is the Φ of the whole candidate system
- As with mechanisms, the cut that makes the *least difference* to the candidate system captures how intrinsically irreducible it is

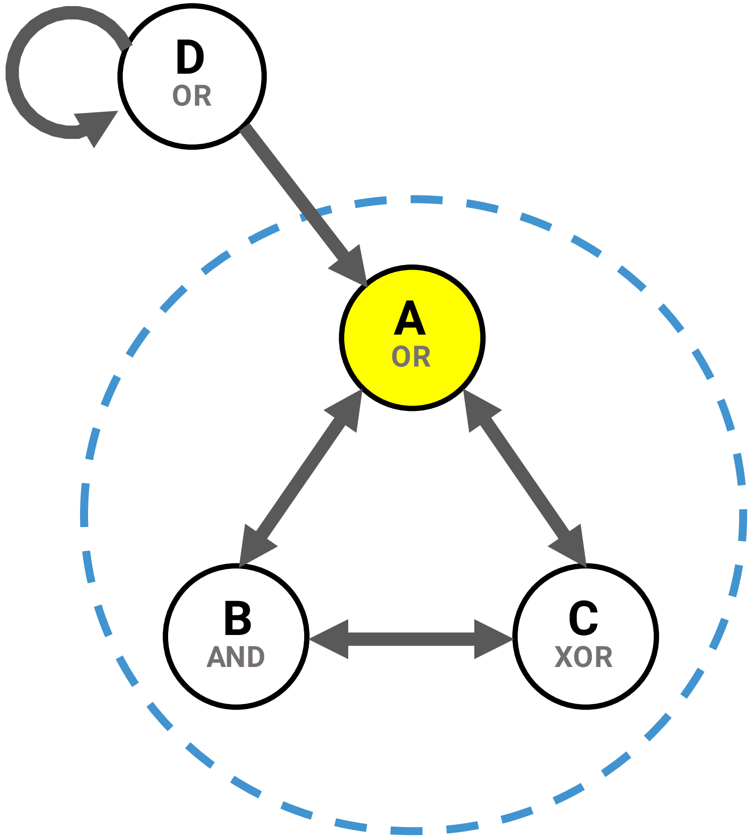

Candidate system **ABC**

$$\Phi = 1.917$$

Outline

- Elements, states, and the TPM
- Background conditions
- Cause-effect repertoires
- Integrated mechanisms: φ
- Concepts and cause-effect structures
- Integrated systems: Φ
- Complexes

Integration and reducibility:
Complexes

- Now, recall that we began the analysis by choosing a candidate system to evaluate
- $\mathbf{\Phi}$ is evaluated for each possible candidate system, and the candidate system with the maximal value, $\mathbf{\Phi}^{\text{max}}$, is called a **complex**
- For brevity we don't consider all candidate systems that include **D**, since the cut **ABC** $\nRightarrow$ **D** will trivially have $\mathbf{\Phi} = 0$

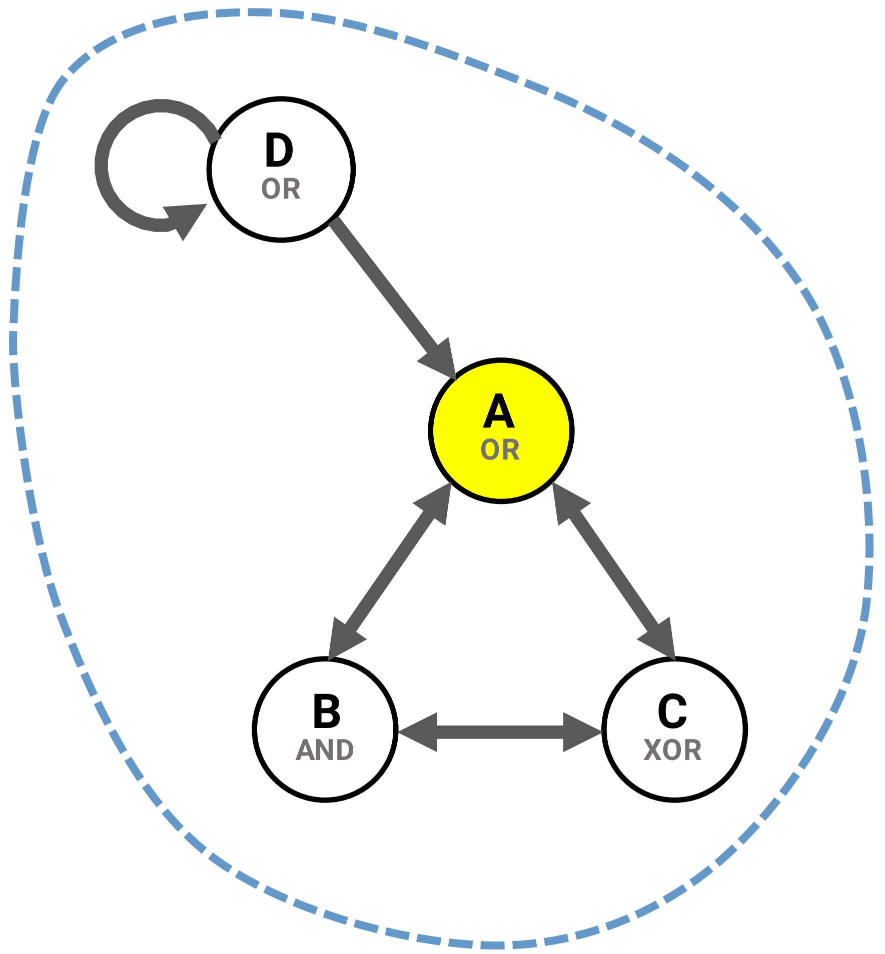

Candidate system **ABCD**

$$\mathbf{\Phi} = 0$$

Integration and reducibility:
Complexes

- Now, recall that we began the analysis by choosing a candidate system to evaluate
- Φ is evaluated for each possible candidate system, and the candidate system with the maximal value, Φ^{max}, is called a **complex**
- For brevity we don't consider all candidate systems that include **D**, since the cut **ABC** $\not\Rightarrow$ **D** will trivially have $\Phi = 0$

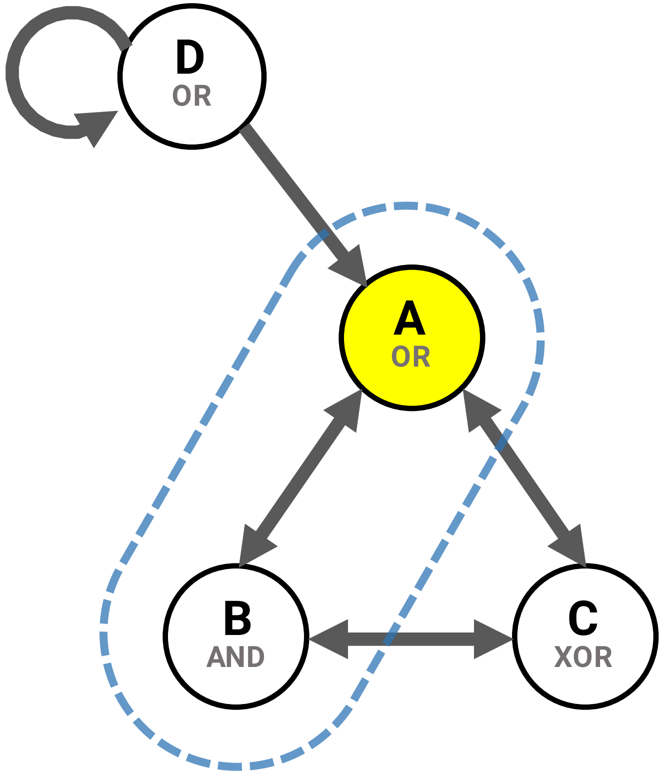

Candidate system **AB**

$$\Phi = 0$$

Integration and reducibility:
Complexes

- Now, recall that we began the analysis by choosing a candidate system to evaluate
- $\mathbf{\Phi}$ is evaluated for each possible candidate system, and the candidate system with the maximal value, $\mathbf{\Phi}^{\text{max}}$, is called a **complex**
- For brevity we don't consider all candidate systems that include **D**, since the cut **ABC** $\not\Rightarrow$ **D** will trivially have $\mathbf{\Phi} = 0$

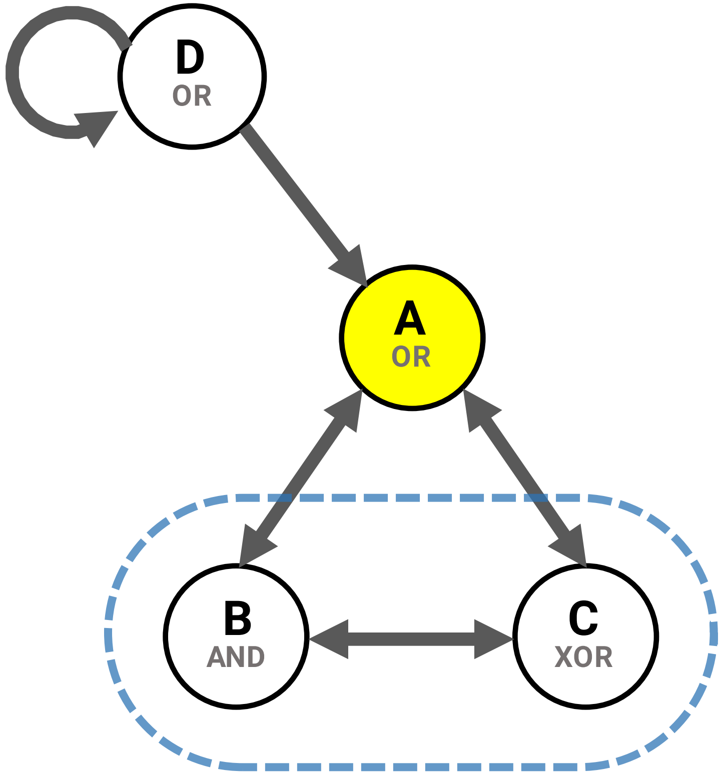

Candidate system **BC**

$$\mathbf{\Phi} = 1.0$$

Integration and reducibility:
Complexes

- Now, recall that we began the analysis by choosing a subset of the network to evaluate as a candidate system
- The next step is to evaluated $\mathbf{\Phi}$ for every candidate system
- The system with the maximal value, $\mathbf{\Phi}^{\text{max}}$, is called a **complex**
- For brevity we don't consider all candidate systems that include **D**, since the cut **ABC** $\not\Rightarrow$ **D** will trivially have $\mathbf{\Phi} = 0$

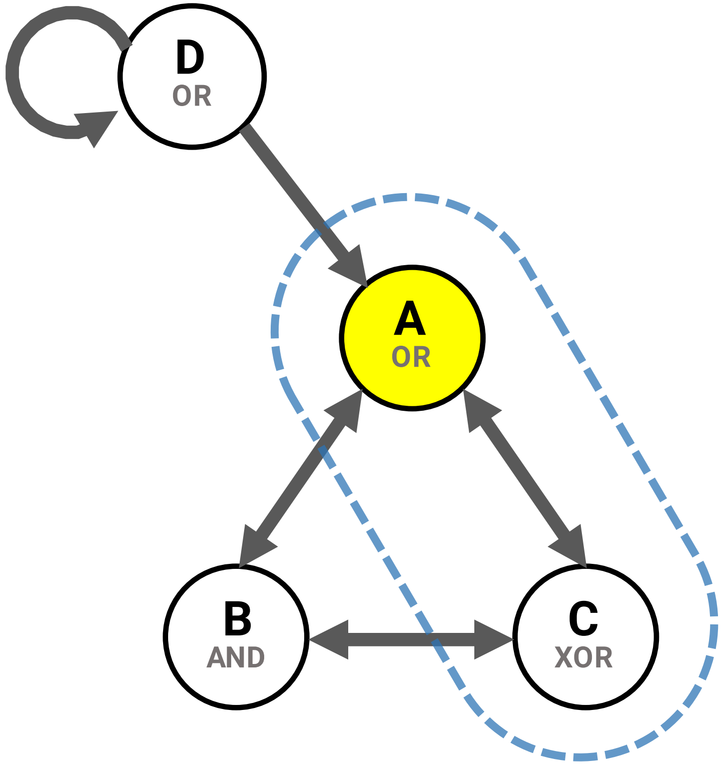

Candidate system **AC**

$$\mathbf{\Phi} = 1.0$$

Integration and reducibility:
Complexes

- The exclusion postulate of IIT dictates that only a complex exists as an integrated entity with a subjective experience
- This defines the “borders” of the physical substrate of consciousness (e.g. the brain, without the sensory or motor neurons)

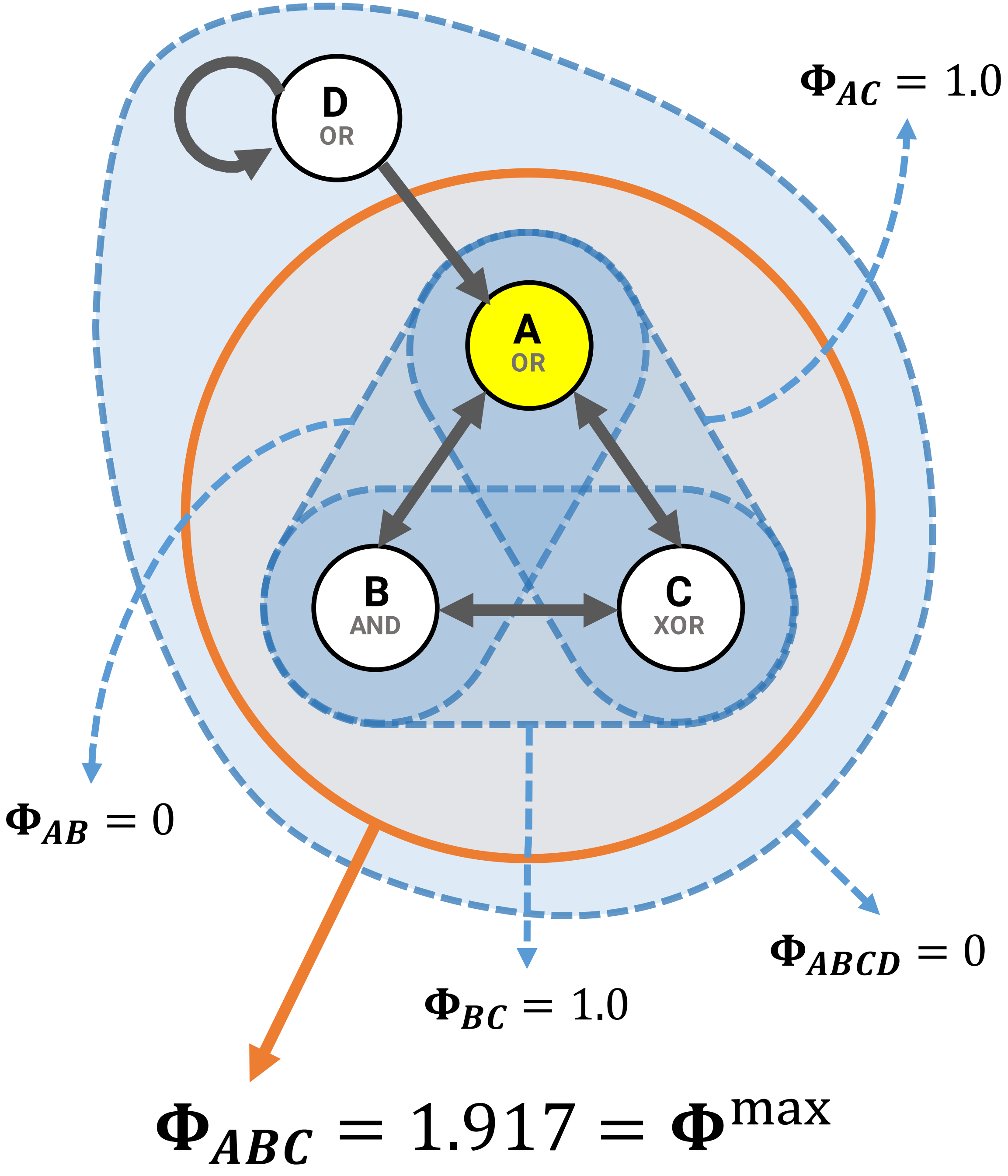

Integration and reducibility:
Complexes

- Finally, note that in general, the search for the system with Φ^{max} must also be carried out over all spatiotemporal groupings of elements

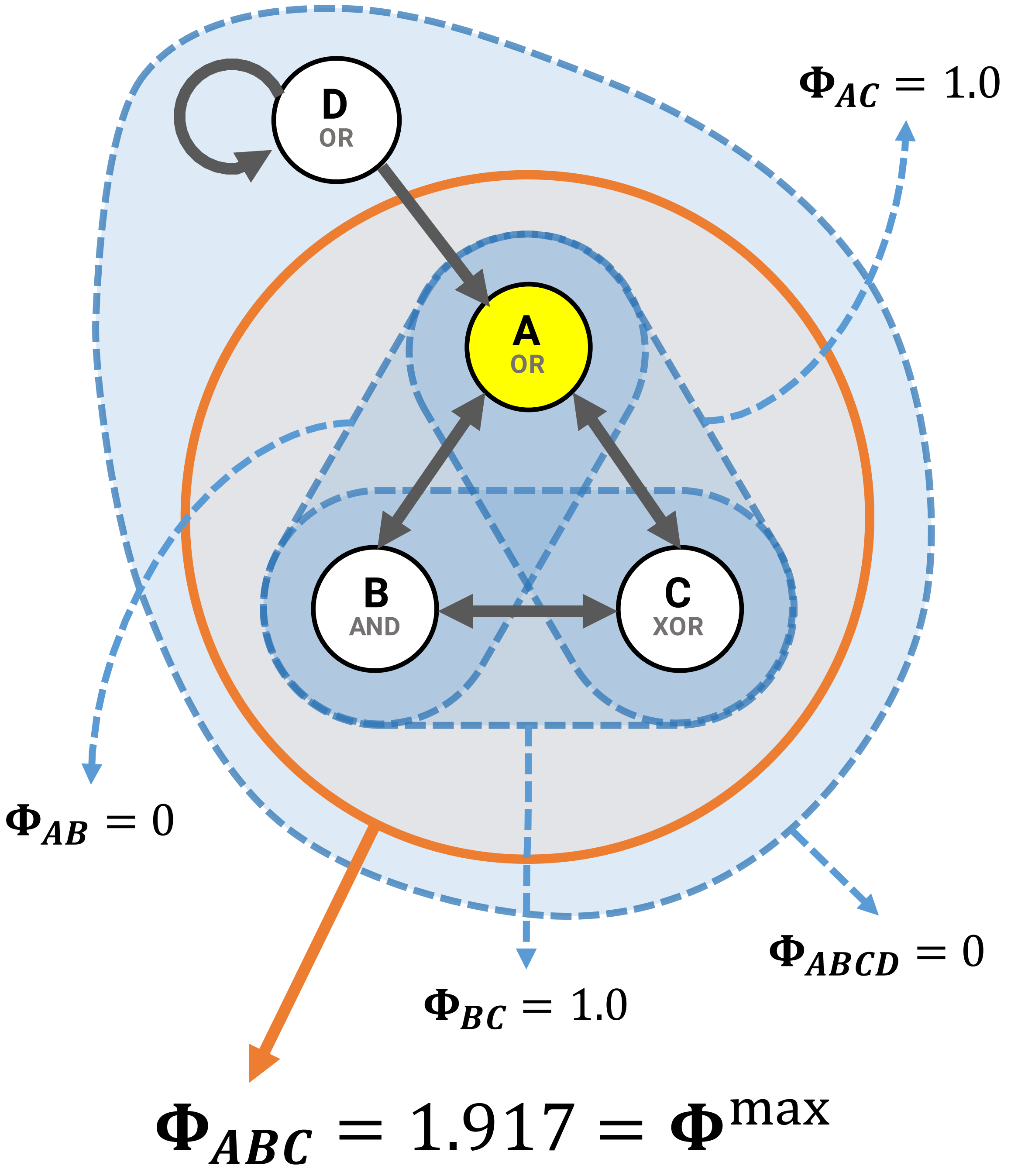

Supplement: S1 Text — Illustration of the algorithm. (PDF) [file pcbi.1006343.s001.pdf]
