# Supplementary material for: PyPhi: A toolbox for integrated information theory
Source: PLoS Comput Biol. 2018 Jul 26;14(7):e1006343. doi: 10.1371/journal.pcbi.1006343 (PMC6080800; doi:10.1371/journal.pcbi.1006343)
Supplement: S2 Text — (PDF) [file pcbi.1006343.s002.pdf]

S2 Text

Memoization and caching optimizations.

During the course of computing a `SystemIrreducibilityAnalysis`, several functions in PyPhi are called multiple times with the same input. For example, calculating `cause_repertoire((A,B), (A,B,C)` and `cause_repertoire((A,C), (A,B,C))` both require calculating `cause_repertoire((A,), (A,B,C))`. Similarly, `cause_repertoire((A,), (B,C))` is both the unpartitioned repertoire of the candidate MIP of mechanism A over purview BC and the first term in the expression for the partitioned repertoire of the candidate MIP of mechanism AB over purview ABC under the partition

$$\frac{A}{BC} \times \frac{B}{A}.$$

In such situations, a natural optimization technique to reduce expensive re-computation of these functions is *memoization*: when a function is computed for a given input, the input-output pair is stored in a lookup table; if the function is called again with that input, the output is simply looked up in the table and returned, without computing the function again.

In PyPhi, memoization is applied to various functions at different levels of the algorithm, listed here:

- `Subsystem._single_node_cause_repertoire()` and `Subsystem._single_node_effect_repertoire()`, the un-normalized cause repertoire of a single-node mechanism and the effect repertoire over a single-node purview, respectively (note that these functions are meant to be called internally by other PyPhi functions and not by the user, as indicated by the leading underscore);
- `Subsystem.cause_repertoire()` and `Subsystem.effect_repertoire()`, the cause and effect repertoires of arbitrary mechanism-purview pairs;
- `Subsystem.mic()` and `Subsystem.mie()`, the MIC and MIE of a mechanism;
- `Network.potential_purviews()`, the purviews which are not necessarily reducible based on the CM;
- Various utility functions such as `pyphi.distribution.max_entropy_distribution()`; and
- `pyphi.compute.sia()`, the full IIT analysis of a `Subsystem`.

At the highest level, `pyphi.compute.sia()` is memoized such that the `SystemIrreducibilityAnalysis` object is stored persistently on the filesystem,

rather than in memory, in a directory named `__pyphi_cache__` (which is automatically created in the directory where the Python session was started). This means that the `SystemIrreducibilityAnalysis` objects are automatically saved across Python sessions and can be quickly retrieved simply by running the same code, which is useful for interactive, exploratory work. This behavior can be controlled with the `pyphi.config.CACHE_SIAS` configuration setting. Note, however, that this feature is primarily for convenience and is not intended to replace explicit data management. Additionally, care must be taken to erase or disable the cache when upgrading to new versions of PyPhi, as changes to the algorithm may invalidate previously computed output. A final caveat: because the results are stored on the filesystem, they can accumulate and occupy a large amount of disk space if the `__pyphi_cache__` directory is not periodically removed.
