# Supplementary material for: PyPhi: A toolbox for integrated information theory
Source: PLoS Comput Biol. 2018 Jul 26;14(7):e1006343. doi: 10.1371/journal.pcbi.1006343 (PMC6080800; doi:10.1371/journal.pcbi.1006343)
Supplement: S3 Text — (PDF) [file pcbi.1006343.s003.pdf]

S3 Text

Proof of the strong connectivity optimization.

Theorem (strong connectivity). *If $G = (V, E)$ is a directed graph that is not strongly connected, then $\Phi(G) = 0$.*

Proof. Since G is not strongly connected, G contains $n \geq 2$ strongly connected components, which we arbitrarily label

$$G_1, G_2, \ldots, G_n \;=\; (V_1, E_1), (V_2, E_2), \ldots, (V_n, E_n).$$

Let $E_{j,k}$ denote the set of directed edges from nodes in component G_j to those in component G_k, $\{(a, b) \in E \mid a \in V_j \text{ and } b \in V_k\}$.

Consider the first component G_1. For every other component G_i, $i \neq 1$, either $E_{1,i} = \varnothing$ or $E_{i,1} = \varnothing$, because otherwise G_1 and G_i would not be distinct connected components. Now let $\overline{G_1}$ be the indices of components that receive no edges from G_1, $\{i \in 1, \ldots, n \mid E_{1,i} = \varnothing\}$. Then let Y be the union of the nodes in these components,

$$Y = \bigcup_{i \,\in\, \overline{G_1}} V_i \;,$$

and let $X = V \setminus Y$. Then X and Y form a partition of V such that there are no edges from any nodes in X to any nodes in Y.

Now consider the system cut $c(X, Y)$ that cuts edges from nodes in X to nodes in Y. Because there are no such edges, none of the node TPMs are changed after applying the cut, and thus the subsystem TPM is unchanged because it is the product of the node TPMs. Since the cause-effect structure of a system is a function of the subsystem's TPM, the cause-effect structure $C_{c(X,Y)}$ of the partitioned subsystem and the cause-effect structure C of the unpartitioned subsystem are identical.

Let $\Phi_{c(X,Y)}(G)$ be the Φ value of G with respect to $c(X, Y)$. By definition, this is the `ces_distance` between C and $C_{c(X,Y)}$. The `ces_distance` function is a metric, so since $C_{c(X,Y)} = C$ we have that

$$\texttt{ces\_distance}(C, C_{c(X,Y)}) \;=\; 0$$

by non-negativity of metrics, and thus $\Phi_{c(X,Y)}(G) = 0$. Now, by definition,

$$\Phi(G) \;=\; \min_{c \,\in\, \mathbb{C}} \; \Phi_c(G)$$

where $\mathbb{C}$ is the set of all system cuts. Since $\Phi_{c(X,Y)}(G) = 0$, by non-negativity we have $\Phi(G) = 0$.

■
