# Supplementary material for: PyPhi: A toolbox for integrated information theory
Source: PLoS Comput Biol. 2018 Jul 26;14(7):e1006343. doi: 10.1371/journal.pcbi.1006343 (PMC6080800; doi:10.1371/journal.pcbi.1006343)
Supplement: S4 Text — (PDF) [file pcbi.1006343.s004.pdf]

S4 Text

Proof of the block-factorable optimization.

Definition (block diagonal). *An* $n \times m$ *matrix* $\mathbf{A}_{n,m}$ *is said to be block diagonal if it can be written as*

$$\mathbf{A}_{n,m} \;=\; \begin{bmatrix} \mathbf{B}_{s,t} & \mathbf{0}_{s,m-t} \\ \mathbf{0}_{n-s,t} & \mathbf{C}_{n-s,m-t} \end{bmatrix},$$

where $1 \leq s < n$ *and* $1 \leq t < m$*.*

We consider sub-matrices of the connectivity matrix CM of the form $\mathrm{CM}(\pi_{\mathrm{row}}, \pi_{\mathrm{col}})$, where

$$[\mathrm{CM}(\pi_{\mathrm{row}}, \pi_{\mathrm{col}})]_{i,j} \;=\; [\mathrm{CM}]_{\pi_{\mathrm{row}}(i), \pi_{\mathrm{col}}(j)}.$$

Definition (block factorable). *A mechanism-purview pair* (M, P) *is said to be block factorable if there exists a permutation* π_M *of the mechanism indices and a permutation* π_P *of the purview indices such that* $\mathrm{CM}(\pi_M, \pi_P)$ *is block diagonal (for effect purviews) or* $\mathrm{CM}(\pi_P, \pi_M)$ *is block diagonal (for cause purviews).*

Theorem (block reducibility). *If a mechanism-purview pair is block factorable, then it is reducible* $(\varphi = 0)$*.*

Proof. Consider a mechanism M constituted of n elements and a purview P constituted of m elements. Assume without loss of generality that P is an effect purview. Since (M, P) is block factorable, there exist permutations π_M and π_P such that $\mathrm{CM}(\pi_M, \pi_P)$ is block diagonal, *i.e.*,

$$\mathrm{CM}(\pi_M, \pi_P) \;=\; \begin{bmatrix} \mathbf{B}_{s,t} & \mathbf{0}_{s,m-t} \\ \mathbf{0}_{n-s,t} & \mathbf{C}_{n-s,m-t} \end{bmatrix},$$

where $1 \leq s < n$ and $1 \leq t < m$.

We define a mechanism-purview partition

$$c \;\coloneqq\; \frac{M_1}{P_1} \times \frac{M_2}{P_2}$$

that cuts edges from M_1 to P_2 and from M_2 to P_1, where

$$\begin{aligned} M_1 &= \{\, \pi_M(i) \mid 1 \leq i < s+1 \,\} \\ M_2 &= \{\, \pi_M(i) \mid s+1 \leq i < n+1 \,\} \\ P_1 &= \{\, \pi_P(i) \mid 1 \leq i < t+1 \,\} \\ P_2 &= \{\, \pi_P(i) \mid t+1 \leq i < m+1 \,\} \end{aligned}$$

Note that $[\mathrm{CM}(\pi_M, \pi_P)]_{i,j} = 0$ if either $i \in M_1$ and $j \in P_2$ or $i \in M_2$ and $j \in P_1$. Thus there are no edges cut by c, and it leaves the subsystem's TPM unchanged.

Since the effect repertoire of a mechanism-purview combination is a function of the subsystem's TPM, the unpartitioned effect repertoire, $\text{ER}(M, P)$ and the partitioned repertoire $\text{ER}_c(M, P)$ are identical.

By definition, $\varphi_c(M, P)$ is the distance between $\text{ER}(M, P)$ and $\text{ER}_c(M, P)$, so $\varphi_c(M, P) = 0$. Now, by definition,

$$\varphi(M, P) = \min_{p \in \mathbb{P}} \varphi_p(M, P),$$

where $\mathbb{P}$ is the set of all partitions of (M, P). Since $\varphi_c(M, P) = 0$, by the non-negativity of metrics we have $\varphi(M, P) = 0$.

■
