# Supplementary material for: PyPhi: A toolbox for integrated information theory
Source: PLoS Comput Biol. 2018 Jul 26;14(7):e1006343. doi: 10.1371/journal.pcbi.1006343 (PMC6080800; doi:10.1371/journal.pcbi.1006343)
Supplement: S5 Text — (PDF) [file pcbi.1006343.s005.pdf]

S5 Text

Proof of an analytical solution to the EMD between effect repertoires.

Theorem (analytical EMD). *Consider two random variables X_1, X_2 with corresponding state spaces Ω_1, Ω_2 and an 'additive' metric D,*

$$D((i_1, i_2), (j_1, j_2)) \; = \; D(i_1, j_1) + D(i_2, j_2) \quad \forall \; (i_1, i_2) \text{ and } (j_1, j_2) \in \Omega_1 \times \Omega_2.$$

Let p_1 and q_1 be two probability distributions on X_1, and let p_2 and q_2 be probability distributions on X_2. If X_1 and X_2 are independent, then the EMD *between the joint distributions $p = p_1 p_2$ and $q = q_1 q_2$, with D as the ground metric, is equal to the sum of the* EMD*s between the marginal distributions:*

$$\text{EMD}(p, q) \; = \; \text{EMD}(p_1, q_1) + \text{EMD}(p_2, q_2).$$

Proof. First, we demonstrate that

$$\text{EMD}(p, q) \; \leq \; \text{EMD}(p_1, q_1) + \text{EMD}(p_2, q_2).$$

To do this, we define a third probability distribution as an intermediate point,

$$r \; \coloneqq \; q_1 p_2.$$

We define the following flow from p to r,

$$f_{p,r}(i_1, i_2, j_1, j_2) \; \coloneqq \; \begin{cases} p_2(i_2) f^*_{p_1,q_1}(i_1, j_1) & \text{if } i_2 = j_2 \\ 0 & \text{otherwise,} \end{cases}$$

where $f^*_{p_1,q_1}$ is the optimal flow for the EMD between p_1 and q_1. With this flow, we have

$$\begin{aligned} \text{EMD}(p, r) \; &\leq \; \sum_{i_1,i_2,j_1,j_2} f_{p,r}(i_1, i_2, j_1, j_2) D((i_1, i_2), (j_1, j_2)) \\ &= \; \sum_{i_1,i_2,j_1} p_2(i_2) f^*_{p_1,q_1}(i_1, j_1) D(i_1, j_1) \\ &= \; \sum_{i_2} p_2(i_2) \sum_{i_1,j_1} f^*_{p_1,q_1}(i_1, j_1) D(i_1, j_1) \\ &= \; \text{EMD}(p_1, q_1) \end{aligned}$$

We next define a flow from r to q,

$$f_{r,q}(i_1, i_2, j_1, j_2) \; \coloneqq \; \begin{cases} q_1(i_1) f^*_{p_2,q_2}(i_2, j_2) & \text{if } i_1 = j_1 \\ 0 & \text{otherwise,} \end{cases}$$

where $f^*_{p_2,q_2}$ is the optimal flow for the EMD between p_2 and q_2. With this flow, we have

$$\begin{aligned}
\mathrm{EMD}(r,q) &\le \sum_{i_1,i_2,j_1,j_2} f_{r,q}(i_1,i_2,j_1,j_2) D((i_1,i_2),\,(j_1,j_2)) \\
&= \sum_{i_1,i_2,j_2} q_1(i_1) f^*_{p_2,q_2}(i_2,j_2) D(i_2,j_2) \\
&= \sum_{i_1} q_1(i_1) \sum_{i_2,j_2} f^*_{p_2,q_2}(i_2,j_2) D(i_2,j_2) \\
&= \mathrm{EMD}(p_2,q_2)
\end{aligned}$$

Then using the triangle inequality (the EMD is a metric), we have

$$\mathrm{EMD}(p,q) \;\le\; \mathrm{EMD}(p,r) + \mathrm{EMD}(r,q) \;\le\; \mathrm{EMD}(p_1,q_1) + \mathrm{EMD}(p_2,q_2).$$

To complete the proof, we next demonstrate that

$$\mathrm{EMD}(p,q) \;\ge\; \mathrm{EMD}(p_1,q_1) + \mathrm{EMD}(p_2,q_2).$$

If $f^*_{p,q}$ is the optimal flow for $\mathrm{EMD}(p,q)$, then define a flow between p_1 and q_1,

$$f_1(i_1,j_1) \;\coloneqq\; \sum_{i_2,j_2} f^*_{p,q}(i_1,i_2,j_1,j_2),$$

and a flow between p_2 and q_2

$$f_2(i_2,j_2) \;\coloneqq\; \sum_{i_1,j_1} f^*_{p,q}(i_1,i_2,j_1,j_2).$$

Then using the additive property of the ground metric D,

$$\begin{aligned}
\mathrm{EMD}(p,q) &= \sum_{i_1,i_2,j_1,j_2} f^*_{p,q}(i_1,i_2,j_1,j_2) D((i_1,i_2),\,(j_1,j_2)) \\
&= \sum_{i_1,i_2,j_1,j_2} f^*_{p,q}(i_1,i_2,j_1,j_2) D(i_1,j_1) + \sum_{i_1,i_2,j_1,j_2} f^*_{p,q}(i_1,i_2,j_1,j_2) D(i_2,j_2) \\
&= \sum_{i_1,j_1} \left(\sum_{i_2,j_2} f^*_{p,q}(i_1,i_2,j_1,j_2) \right) D(i_1,j_1) \\
&\qquad + \sum_{i_2,j_2} \left(\sum_{i_1,j_1} f^*_{p,q}(i_1,i_2,j_1,j_2) \right) D(i_2,j_2) \\
&= \sum_{i_1,j_1} f_1(i_1,j_1) D(i_1,j_1) + \sum_{i_2,j_2} f_2(i_2,j_2) D(i_2,j_2) \\
&\ge \mathrm{EMD}(p_1,q_1) + \mathrm{EMD}(p_2,q_2).
\end{aligned}$$

Therefore $\mathrm{EMD}(p,q) = \mathrm{EMD}(p_1,q_1) + \mathrm{EMD}(p_2,q_2)$.

■

Perhaps it is worth demonstrating that the flows f_1, f_2, $f_{p,r}$ and $f_{r,q}$ satisfy the EMD requirements. Consider $f_{p,r}$,

$$f_{p,r}(i_1, i_2, j_1, j_2) = \begin{cases} p_2(i_2) f^*_{p_1,q_1}(i_1, j_1) & \text{if } i_2 = j_2 \\ 0 & \text{otherwise,} \end{cases}$$

where $f^*_{p_1,q_1}$ is the optimal flow for the EMD between p_1 and q_1.

Since $p_2(i_2) \geq 0$ (probability) and $f^*_{p_1,q_1}(i_1, j_1) \geq 0$ (definition of optimal flow),

$$f_{p,r}(i_1, i_2, j_1, j_2) \geq 0.$$

Next,

$$\begin{aligned} \sum_{j_1,j_2} f_{p,r}(i_1, i_2, j_1, j_2) &= \sum_{j_1} p_2(i_2) f^*_{p_1,q_1}(i_1, j_1) \\ &= q_1(i_1) p_2(i_2) \\ &= r(i_1, i_2), \end{aligned}$$

and

$$\begin{aligned} \sum_{i_1,i_2} f_{p,r}(i_1, i_2, j_1, j_2) &= \sum_{i_1} p_2(j_2) f^*_{p_1,q_1}(i_1, j_1) \\ &= q_1(j_1) p_2(i_2) \\ &= r(j_1, j_2). \end{aligned}$$

Finally,

$$\begin{aligned} \sum_{i_1,i_2,j_1,j_2} f_{p,r}(i_1, i_2, j_1, j_2) &= \sum_{i_2} p_2(i_2) \sum_{i_1,j_1} f^*_{p_1,q_1}(i_1, j_1) \\ &= \sum_{i_1,j_1} f^*_{p_1,q_1}(i_1, j_1) \\ &= 1 \end{aligned}$$

Thus $f_{p,r}$ satisfies the criteria for a potential flow. The others follow similarly.
