# Supplementary material for: PyPhi: A toolbox for integrated information theory
Source: PLoS Comput Biol. 2018 Jul 26;14(7):e1006343. doi: 10.1371/journal.pcbi.1006343 (PMC6080800; doi:10.1371/journal.pcbi.1006343)
Supplement: S1 File — Note that installing PyPhi via ‘pip’ or downloading the source code from GitHub is recommended in order to obtain the most up-to-date version of the software. (ZIP) [file pcbi.1006343.s006.zip › S6_File/pyphi-v1.1.0/docs/_themes/kr/layout.html]

{%- extends "basic/layout.html" %}
{%- block extrahead %}
{{ super() }}
{% if theme\_touch\_icon %}
{% endif %}

{% endblock %}
{%- block relbar2 %}{% endblock %}
{%- block footer %}

© Copyright {{ copyright }}.

{%- endblock %}
