# Supplementary material for: PyPhi: A toolbox for integrated information theory
Source: PLoS Comput Biol. 2018 Jul 26;14(7):e1006343. doi: 10.1371/journal.pcbi.1006343 (PMC6080800; doi:10.1371/journal.pcbi.1006343)
Supplement: S1 File — Note that installing PyPhi via ‘pip’ or downloading the source code from GitHub is recommended in order to obtain the most up-to-date version of the software. (ZIP) [file pcbi.1006343.s006.zip › S6_File/pyphi-v1.1.0/docs/_themes/kr/relations.html]

### Related Topics

- Documentation overview
  {%- for parent in parents %}- {{ parent.title }}
    {%- endfor %}
    {%- if prev %}- Previous: {{ prev.title }}
    {%- endif %}
    {%- if next %}- Next: {{ next.title }}
    {%- endif %}
    {%- for parent in parents %}
  {%- endfor %}
