# Supplementary material for: PyPhi: A toolbox for integrated information theory
Source: PLoS Comput Biol. 2018 Jul 26;14(7):e1006343. doi: 10.1371/journal.pcbi.1006343 (PMC6080800; doi:10.1371/journal.pcbi.1006343)
Supplement: S1 File — Note that installing PyPhi via ‘pip’ or downloading the source code from GitHub is recommended in order to obtain the most up-to-date version of the software. (ZIP) [file pcbi.1006343.s006.zip › S6_File/pyphi-v1.1.0/docs/_themes/kr_small/layout.html]

{% extends "basic/layout.html" %}
{% block header %}
{{ super() }}
{% if pagename == 'index' %}

{% endif %}
{% endblock %}
{% block footer %}
{% if pagename == 'index' %}

{% endif %}
{% endblock %}
{# do not display relbars #}
{% block relbar1 %}{% endblock %}
{% block relbar2 %}
{% if theme\_github\_fork %}
{% endif %}
{% endblock %}
{% block sidebar1 %}{% endblock %}
{% block sidebar2 %}{% endblock %}
