# Supplementary material for: PyPhi: A toolbox for integrated information theory
Source: PLoS Comput Biol. 2018 Jul 26;14(7):e1006343. doi: 10.1371/journal.pcbi.1006343 (PMC6080800; doi:10.1371/journal.pcbi.1006343)
Supplement: S1 File — Note that installing PyPhi via ‘pip’ or downloading the source code from GitHub is recommended in order to obtain the most up-to-date version of the software. (ZIP) [file pcbi.1006343.s006.zip › S6_File/pyphi-v1.1.0/docs/_templates/layout.html]

{# layout.html #}
{# Import the theme's layout. #}
{% extends "!layout.html" %}
{# Add custom css. #}
{% set css\_files = css\_files + ['\_static/custom.css'] %}
