# Supplementary figures and images for: PyPhi: A toolbox for integrated information theory
Source: PLoS Comput Biol. 2018 Jul 26;14(7):e1006343. doi: 10.1371/journal.pcbi.1006343 (PMC6080800; doi:10.1371/journal.pcbi.1006343)

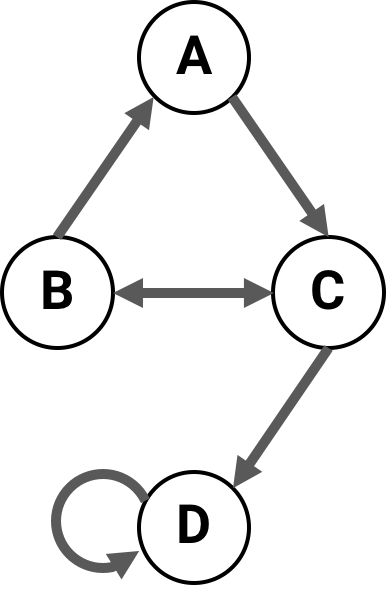

Supplement: S1 File — Note that installing PyPhi via ‘pip’ or downloading the source code from GitHub is recommended in order to obtain the most up-to-date version of the software. (ZIP) [file pcbi.1006343.s006.zip › S6_File/pyphi-v1.1.0/docs/_static/connectivity-matrix-example-network.png]

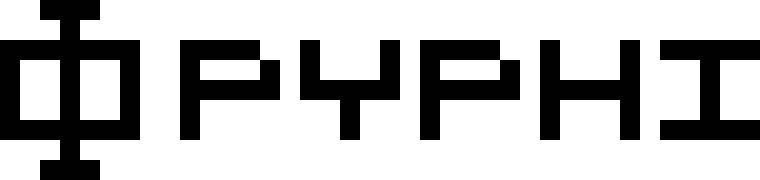

Supplement: S1 File — Note that installing PyPhi via ‘pip’ or downloading the source code from GitHub is recommended in order to obtain the most up-to-date version of the software. (ZIP) [file pcbi.1006343.s006.zip › S6_File/pyphi-v1.1.0/docs/_static/pyphi-logo-text-760x180.png]

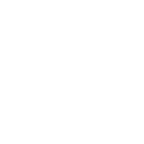

Supplement: S1 File — Note that installing PyPhi via ‘pip’ or downloading the source code from GitHub is recommended in order to obtain the most up-to-date version of the software. (ZIP) [file pcbi.1006343.s006.zip › S6_File/pyphi-v1.1.0/docs/_static/blank.png]

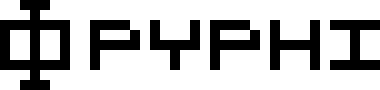

Supplement: S1 File — Note that installing PyPhi via ‘pip’ or downloading the source code from GitHub is recommended in order to obtain the most up-to-date version of the software. (ZIP) [file pcbi.1006343.s006.zip › S6_File/pyphi-v1.1.0/docs/_static/pyphi-logo-text-380x90.png]

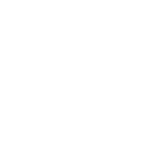

Supplement: S1 File — Note that installing PyPhi via ‘pip’ or downloading the source code from GitHub is recommended in order to obtain the most up-to-date version of the software. (ZIP) [file pcbi.1006343.s006.zip › S6_File/pyphi-v1.1.0/docs/_static/phi_144x144.png]

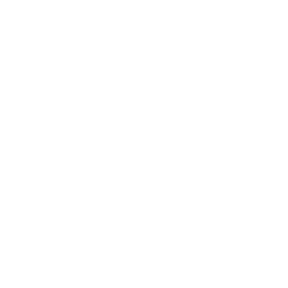

Supplement: S1 File — Note that installing PyPhi via ‘pip’ or downloading the source code from GitHub is recommended in order to obtain the most up-to-date version of the software. (ZIP) [file pcbi.1006343.s006.zip › S6_File/pyphi-v1.1.0/docs/_static/phi_288x288.png]

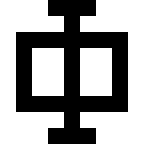

Supplement: S1 File — Note that installing PyPhi via ‘pip’ or downloading the source code from GitHub is recommended in order to obtain the most up-to-date version of the software. (ZIP) [file pcbi.1006343.s006.zip › S6_File/pyphi-v1.1.0/docs/_static/phi-black.png]

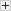

Supplement: S2 File — Note that accessing the documentation online at https://pyphi.readthedocs.io is recommended, as it is updated for each new version of the software. (ZIP) [file pcbi.1006343.s007.zip › pyphi-v1.1.0-documentation/_static/plus.png]

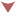

Supplement: S2 File — Note that accessing the documentation online at https://pyphi.readthedocs.io is recommended, as it is updated for each new version of the software. (ZIP) [file pcbi.1006343.s007.zip › pyphi-v1.1.0-documentation/_static/down-pressed.png]

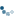

Supplement: S2 File — Note that accessing the documentation online at https://pyphi.readthedocs.io is recommended, as it is updated for each new version of the software. (ZIP) [file pcbi.1006343.s007.zip › pyphi-v1.1.0-documentation/_static/ajax-loader.gif]

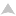

Supplement: S2 File — Note that accessing the documentation online at https://pyphi.readthedocs.io is recommended, as it is updated for each new version of the software. (ZIP) [file pcbi.1006343.s007.zip › pyphi-v1.1.0-documentation/_static/up.png]

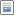

Supplement: S2 File — Note that accessing the documentation online at https://pyphi.readthedocs.io is recommended, as it is updated for each new version of the software. (ZIP) [file pcbi.1006343.s007.zip › pyphi-v1.1.0-documentation/_static/file.png]

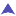

Supplement: S2 File — Note that accessing the documentation online at https://pyphi.readthedocs.io is recommended, as it is updated for each new version of the software. (ZIP) [file pcbi.1006343.s007.zip › pyphi-v1.1.0-documentation/_static/up-pressed.png]

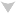

Supplement: S2 File — Note that accessing the documentation online at https://pyphi.readthedocs.io is recommended, as it is updated for each new version of the software. (ZIP) [file pcbi.1006343.s007.zip › pyphi-v1.1.0-documentation/_static/down.png]

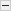

Supplement: S2 File — Note that accessing the documentation online at https://pyphi.readthedocs.io is recommended, as it is updated for each new version of the software. (ZIP) [file pcbi.1006343.s007.zip › pyphi-v1.1.0-documentation/_static/minus.png]

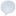

Supplement: S2 File — Note that accessing the documentation online at https://pyphi.readthedocs.io is recommended, as it is updated for each new version of the software. (ZIP) [file pcbi.1006343.s007.zip › pyphi-v1.1.0-documentation/_static/comment.png]

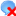

Supplement: S2 File — Note that accessing the documentation online at https://pyphi.readthedocs.io is recommended, as it is updated for each new version of the software. (ZIP) [file pcbi.1006343.s007.zip › pyphi-v1.1.0-documentation/_static/comment-close.png]

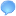

Supplement: S2 File — Note that accessing the documentation online at https://pyphi.readthedocs.io is recommended, as it is updated for each new version of the software. (ZIP) [file pcbi.1006343.s007.zip › pyphi-v1.1.0-documentation/_static/comment-bright.png]
