# Supplementary material for: PyPhi: A toolbox for integrated information theory
Source: PLoS Comput Biol. 2018 Jul 26;14(7):e1006343. doi: 10.1371/journal.pcbi.1006343 (PMC6080800; doi:10.1371/journal.pcbi.1006343)
Supplement: S2 File — Note that accessing the documentation online at https://pyphi.readthedocs.io is recommended, as it is updated for each new version of the software. (ZIP) [file pcbi.1006343.s007.zip › pyphi-v1.1.0-documentation/index.html]

  


v1.1.0 documentation


PyPhi

stable

Usage and Examples

- Installation
- Getting started
- Basic Usage
- IIT 3.0 Paper (2014)
- Conditional Independence
- XOR Network
- Emergence (coarse-graining and blackboxing)
- Actual Causation
- Residue
- Magic Cuts
- Detailed installation guide for macOS

Conventions

- Transition probability matrix conventions
- Connectivity matrix conventions

Configuration

- Loading a configuration
- Approximations and theoretical options
- Parallelization and system resources
- Memoization and caching
- Logging
- Numerical precision
- The `config` API

API Reference

- `actual`
- `cache`
- `compute`
- `compute.distance`
- `compute.network`
- `compute.parallel`
- `compute.subsystem`
- `conf`
- `connectivity`
- `constants`
- `convert`
- `direction`
- `distance`
- `distribution`
- `examples`
- `exceptions`
- `jsonify`
- `macro`
- `models`
- `models.actual_causation`
- `models.cuts`
- `models.mechanism`
- `models.subsystem`
- `network`
- `node`
- `partition`
- `subsystem`
- `timescale`
- `tpm`
- `utils`
- `validate`

PyPhi

- Docs »
- v1.1.0 documentation
- Edit on GitHub

---

# PyPhi¶

PyPhi is a Python library for computing integrated information.

To report issues, use the issue tracker on the GitHub repository. Bug reports and pull requests are
welcome.

For general discussion, you are welcome to join the pyphi-users group.

## Installation¶

To install the latest stable release, run

```
pip install pyphi
```

To install the latest development version, which is a work in progress and may
have bugs, run

```
pip install "git+https://github.com/wmayner/pyphi@develop#egg=pyphi"
```

Tip

For detailed instructions on how to install PyPhi on macOS, see the
Detailed installation guide for macOS.

Note

**Windows users:** PyPhi is only supported on Linux and macOS operating
systems. However, you can run it on Windows by using the Anaconda Python distribution and installing
PyPhi with conda: `conda install -c
wmayner pyphi`

### Installation¶

To install the latest stable release, run

```
pip install pyphi
```

To install the latest development version, which is a work in progress and may
have bugs, run

```
pip install "git+https://github.com/wmayner/pyphi@develop#egg=pyphi"
```

Tip

For detailed instructions on how to install PyPhi on macOS, see the
Detailed installation guide for macOS.

Note

**Windows users:** PyPhi is only supported on Linux and macOS operating
systems. However, you can run it on Windows by using the Anaconda Python distribution and installing
PyPhi with conda: `conda install -c
wmayner pyphi`

### Getting started¶

To explore the following examples, install IPython by running `pip install ipython` on the
command line. Then run it with the command `ipython`.

Lines of code beginning with `>>>` and `...` can be pasted directly into
IPython.

---

### Basic Usage¶

Let’s make a simple 3-node network and compute its \(\Phi\).

To make a network, we need a TPM and (optionally) a connectivity matrix. The
TPM can be in more than one form; see the documentation for `Network`. Here
we’ll use the 2-dimensional state-by-node form.

```
>>> import pyphi
>>> import numpy as np
>>> tpm = np.array([
...     [0, 0, 0],
...     [0, 0, 1],
...     [1, 0, 1],
...     [1, 0, 0],
...     [1, 1, 0],
...     [1, 1, 1],
...     [1, 1, 1],
...     [1, 1, 0]
... ])
```

The connectivity matrix is a square matrix such that the \((i,j)^{\textrm{th}}\) entry is 1 if
there is a connection from node \(i\) to node \(j\), and 0 otherwise.

```
>>> cm = np.array([
...     [0, 0, 1],
...     [1, 0, 1],
...     [1, 1, 0]
... ])
```

We’ll also make labels for the network nodes so that PyPhi’s output is easier
to read.

```
>>> labels = ('A', 'B', 'C')
```

Now we construct the network itself with the arguments we just created:

```
>>> network = pyphi.Network(tpm, cm=cm, node_labels=labels)
```

The next step is to define a subsystem for which we want to evaluate \(\Phi\).
To make a subsystem, we need the network that it belongs to, the state of that
network, and the indices of the subset of nodes which should be included.

The state should be an \(n\)-tuple, where \(n\) is the number of nodes in the
network, and where the \(i^{\textrm{th}}\) element is the state of the \(i^{\textrm{th}}\) node in the
network.

```
>>> state = (1, 0, 0)
```

In this case, we want the \(\Phi\) of the entire network, so we simply include
every node in the network in our subsystem:

```
>>> node_indices = (0, 1, 2)
>>> subsystem = pyphi.Subsystem(network, state, node_indices)
```

Tip

If you do not explicitly provide node indices to a `Subsystem` the system
will, by default, cover the entire network. For example, the following is
equivalent to the above definition of `subsystem`:

```
>>> subsystem = pyphi.Subsystem(network, state)
```

Tip

Node labels can be used instead of indices when constructing a `Subsystem`:

```
>>> pyphi.Subsystem(network, state, ('B', 'C'))
Subsystem(B, C)
```

Now we use the `phi()` function to compute the \(\Phi\) of our
subsystem:

```
>>> pyphi.compute.phi(subsystem)
2.3125
```

If we want to take a deeper look at the integrated-information-theoretic
properties of our network, we can access all the intermediate quantities and
structures that are calculated in the course of arriving at a final \(\Phi\)
value by using `sia()`. This returns a nested object,
`SystemIrreducibilityAnalysis`, that contains data about the subsystem’s
cause-effect structure, cause and effect repertoires, etc.

```
>>> sia = pyphi.compute.sia(subsystem)
```

For instance, we can see that this network has 4 concepts:

```
>>> len(sia.ces)
4
```

See the documentation for `SystemIrreducibilityAnalysis` and `Concept` for more
information on these objects.

Tip

The network and subsystem discussed here are returned by the
`pyphi.examples.basic_network()` and
`pyphi.examples.basic_subsystem()` functions.

### IIT 3.0 Paper (2014)¶

This section is meant to serve as a companion to the paper From the
Phenomenology to the Mechanisms of Consciousness: Integrated Information Theory
3.0
by Oizumi, Albantakis, and Tononi, and as a demonstration of how to use PyPhi.
Readers are encouraged to follow along and analyze the systems shown in the
figures, in order to become more familiar with both the theory and the
software.

Install IPython by running `pip install
ipython` on the command line. Then run it with the command `ipython`.

Lines of code beginning with `>>>` and `...` can be pasted directly into
IPython.

We begin by importing PyPhi and NumPy:

```
>>> import pyphi
>>> import numpy as np
```

#### Figure 1¶

**Existence: Mechanisms in a state having causal power.**

For the first figure, we’ll demonstrate how to set up a network and a candidate
set. In PyPhi, networks are built by specifying a transition probability matrix
and (optionally) a connectivity matrix. (If no connectivity matrix is given,
full connectivity is assumed.) So, to set up the system shown in Figure 1,
we’ll start by defining its TPM.

Note

The TPM in the figure is given in **state-by-state** form; there is a row
and a column for each state. However, in PyPhi, we use a more compact
representation: **state-by-node** form, in which there is a row for each
state, but a column for each node. The \((i,j)^{\textrm{th}}\) entry gives the probability
that the \(j^{\textrm{th}}\) node is ON in the \(i^{\textrm{th}}\) state. For more information on how
TPMs are represented in PyPhi, see Transition probability matrix conventions.

In the figure, the TPM is shown only for the candidate set. We’ll define the
entire network’s TPM. Also, nodes \(D\), \(E\) and \(F\) are not assigned mechanisms;
for the purposes of this example we will assume they are OR gates. With that
assumption, we get the following TPM (before copying and pasting, see note
below):

```
>>> tpm = np.array([
...     [0, 0, 0, 0, 0, 0],
...     [0, 0, 1, 0, 0, 0],
...     [1, 0, 1, 0, 1, 0],
...     [1, 0, 0, 0, 1, 0],
...     [1, 0, 0, 0, 0, 0],
...     [1, 1, 1, 0, 0, 0],
...     [1, 0, 1, 0, 1, 0],
...     [1, 1, 0, 0, 1, 0],
...     [1, 0, 0, 0, 0, 0],
...     [1, 0, 1, 0, 0, 0],
...     [1, 0, 1, 0, 1, 0],
...     [1, 0, 0, 0, 1, 0],
...     [1, 0, 0, 0, 0, 0],
...     [1, 1, 1, 0, 0, 0],
...     [1, 0, 1, 0, 1, 0],
...     [1, 1, 0, 0, 1, 0],
...     [0, 0, 0, 0, 0, 0],
...     [0, 0, 1, 0, 0, 0],
...     [1, 0, 1, 0, 1, 0],
...     [1, 0, 0, 0, 1, 0],
...     [1, 0, 0, 0, 0, 0],
...     [1, 1, 1, 0, 0, 0],
...     [1, 0, 1, 0, 1, 0],
...     [1, 1, 0, 0, 1, 0],
...     [1, 0, 0, 0, 0, 0],
...     [1, 0, 1, 0, 0, 0],
...     [1, 0, 1, 0, 1, 0],
...     [1, 0, 0, 0, 1, 0],
...     [1, 0, 0, 0, 0, 0],
...     [1, 1, 1, 0, 0, 0],
...     [1, 0, 1, 0, 1, 0],
...     [1, 1, 0, 0, 1, 0],
...     [0, 0, 0, 0, 0, 0],
...     [0, 0, 1, 0, 0, 0],
...     [1, 0, 1, 0, 1, 0],
...     [1, 0, 0, 0, 1, 0],
...     [1, 0, 0, 0, 0, 0],
...     [1, 1, 1, 0, 0, 0],
...     [1, 0, 1, 0, 1, 0],
...     [1, 1, 0, 0, 1, 0],
...     [1, 0, 0, 0, 0, 0],
...     [1, 0, 1, 0, 0, 0],
...     [1, 0, 1, 0, 1, 0],
...     [1, 0, 0, 0, 1, 0],
...     [1, 0, 0, 0, 0, 0],
...     [1, 1, 1, 0, 0, 0],
...     [1, 0, 1, 0, 1, 0],
...     [1, 1, 0, 0, 1, 0],
...     [0, 0, 0, 0, 0, 0],
...     [0, 0, 1, 0, 0, 0],
...     [1, 0, 1, 0, 1, 0],
...     [1, 0, 0, 0, 1, 0],
...     [1, 0, 0, 0, 0, 0],
...     [1, 1, 1, 0, 0, 0],
...     [1, 0, 1, 0, 1, 0],
...     [1, 1, 0, 0, 1, 0],
...     [1, 0, 0, 0, 0, 0],
...     [1, 0, 1, 0, 0, 0],
...     [1, 0, 1, 0, 1, 0],
...     [1, 0, 0, 0, 1, 0],
...     [1, 0, 0, 0, 0, 0],
...     [1, 1, 1, 0, 0, 0],
...     [1, 0, 1, 0, 1, 0],
...     [1, 1, 0, 0, 1, 0]
... ])
```

Note

This network is already built for you; you can get it from the `examples`
module with `network = pyphi.examples.fig0a()`. The TPM can then be
accessed with `network.tpm`.

Next we’ll define the connectivity matrix. In PyPhi, the \((i,j)^{\textrm{th}}\) entry in a
connectivity matrix indicates whether node \(i\) is connected to node \(j\). Thus,
this network’s connectivity matrix is

```
>>> cm = np.array([
...     [0, 1, 1, 0, 0, 0],
...     [1, 0, 1, 0, 1, 0],
...     [1, 1, 0, 0, 0, 0],
...     [1, 0, 0, 0, 0, 0],
...     [0, 0, 0, 0, 0, 0],
...     [0, 0, 0, 0, 0, 0]
... ])
```

Now we can pass the TPM and connectivity matrix as arguments to the network
constructor:

```
>>> network = pyphi.Network(tpm, cm=cm)
```

Now the network shown in the figure is stored in a variable called `network`.
You can find more information about the network object we just created by
running `help(network)` or by consulting the documentation for `Network`.

The next step is to define the candidate set shown in the figure, consisting of
nodes \(A\), \(B\) and \(C\). In PyPhi, a candidate set for \(\Phi\) evaluation is
represented by the `Subsystem` class. Subsystems are built by giving the
network it is a part of, the state of the network, and indices of the nodes to
be included in the subsystem. So, we define our candidate set like so:

```
>>> state = (1, 0, 0, 0, 1, 0)
>>> ABC = pyphi.Subsystem(network, state, [0, 1, 2])
```

For more information on the subsystem object, see the documentation for
`Subsystem`.

That covers the basic workflow with PyPhi and introduces the two types of
objects we use to represent and analyze networks. First you define the network
of interest with a TPM and connectivity matrix; then you define a candidate set
you want to analyze.

#### Figure 3¶

**Information requires selectivity.**

##### (A)¶

We’ll start by setting up the subsytem depicted in the figure and labeling the
nodes. In this case, the subsystem is just the entire network.

```
>>> network = pyphi.examples.fig3a()
>>> state = (1, 0, 0, 0)
>>> subsystem = pyphi.Subsystem(network, state)
>>> A, B, C, D = subsystem.node_indices
```

Since the connections are noisy, we see that \(A = 1\) is unselective; all
previous states are equally likely:

```
>>> subsystem.cause_repertoire((A,), (B, C, D))
array([[[[0.125, 0.125],
         [0.125, 0.125]],

        [[0.125, 0.125],
         [0.125, 0.125]]]])
```

And this gives us zero cause information:

```
>>> subsystem.cause_info((A,), (B, C, D))
0.0
```

##### (B)¶

The same as (A) but without noisy connections:

```
>>> network = pyphi.examples.fig3b()
>>> subsystem = pyphi.Subsystem(network, state)
>>> A, B, C, D = subsystem.node_indices
```

Now, \(A\)‘s cause repertoire is maximally selective.

```
>>> cr = subsystem.cause_repertoire((A,), (B, C, D))
>>> cr
array([[[[0., 0.],
         [0., 0.]],

        [[0., 0.],
         [0., 1.]]]])
```

Since the cause repertoire is over the purview \(BCD\), the first dimension
(which corresponds to \(A\)‘s states) is a singleton. We can squeeze out \(A\)‘s
singleton dimension with

```
>>> cr = cr.squeeze()
```

and now we can see that the probability of \(B\), \(C\), and \(D\) having been all ON
is 1:

```
>>> cr[(1, 1, 1)]
1.0
```

Now the cause information specified by \(A = 1\) is \(1.5\):

```
>>> subsystem.cause_info((A,), (B, C, D))
1.5
```

##### (C)¶

The same as (B) but with \(A = 0\):

```
>>> state = (0, 0, 0, 0)
>>> subsystem = pyphi.Subsystem(network, state)
>>> A, B, C, D = subsystem.node_indices
```

And here the cause repertoire is minimally selective, only ruling out the state
where \(B\), \(C\), and \(D\) were all ON:

```
>>> subsystem.cause_repertoire((A,), (B, C, D))
array([[[[0.14285714, 0.14285714],
         [0.14285714, 0.14285714]],

        [[0.14285714, 0.14285714],
         [0.14285714, 0.        ]]]])
```

And so we have less cause information:

```
>>> subsystem.cause_info((A,), (B, C, D))
0.214284
```

#### Figure 4¶

**Information: “Differences that make a difference to a system from its own
intrinsic perspective.”**

First we’ll get the network from the `examples` module, set up a subsystem, and
label the nodes, as usual:

```
>>> network = pyphi.examples.fig4()
>>> state = (1, 0, 0)
>>> subsystem = pyphi.Subsystem(network, state)
>>> A, B, C = subsystem.node_indices
```

Then we’ll compute the cause and effect repertoires of mechanism \(A\) over
purview \(ABC\):

```
>>> subsystem.cause_repertoire((A,), (A, B, C))
array([[[0.        , 0.16666667],
        [0.16666667, 0.16666667]],

       [[0.        , 0.16666667],
        [0.16666667, 0.16666667]]])
>>> subsystem.effect_repertoire((A,), (A, B, C))
array([[[0.0625, 0.0625],
        [0.0625, 0.0625]],

       [[0.1875, 0.1875],
        [0.1875, 0.1875]]])
```

And the unconstrained repertoires over the same (these functions don’t take a
mechanism; they only take a purview):

```
>>> subsystem.unconstrained_cause_repertoire((A, B, C))
array([[[0.125, 0.125],
        [0.125, 0.125]],

       [[0.125, 0.125],
        [0.125, 0.125]]])
>>> subsystem.unconstrained_effect_repertoire((A, B, C))
array([[[0.09375, 0.09375],
        [0.03125, 0.03125]],

       [[0.28125, 0.28125],
        [0.09375, 0.09375]]])
```

The Earth Mover’s distance between them gives the cause and effect information:

```
>>> subsystem.cause_info((A,), (A, B, C))
0.333332
>>> subsystem.effect_info((A,), (A, B, C))
0.25
```

And the minimum of those gives the cause-effect information:

```
>>> subsystem.cause_effect_info((A,), (A, B, C))
0.25
```

#### Figure 5¶

**A mechanism generates information only if it has both selective causes and
selective effects within the system.**

##### (A)¶

```
>>> network = pyphi.examples.fig5a()
>>> state = (1, 1, 1)
>>> subsystem = pyphi.Subsystem(network, state)
>>> A, B, C = subsystem.node_indices
```

\(A\) has inputs, so its cause repertoire is selective and it has cause
information:

```
>>> subsystem.cause_repertoire((A,), (A, B, C))
array([[[0. , 0. ],
        [0. , 0.5]],

       [[0. , 0. ],
        [0. , 0.5]]])
>>> subsystem.cause_info((A,), (A, B, C))
1.0
```

But because it has no outputs, its effect repertoire no different from the
unconstrained effect repertoire, so it has no effect information:

```
>>> np.array_equal(subsystem.effect_repertoire((A,), (A, B, C)),
...                subsystem.unconstrained_effect_repertoire((A, B, C)))
True
>>> subsystem.effect_info((A,), (A, B, C))
0.0
```

And thus its cause effect information is zero.

```
>>> subsystem.cause_effect_info((A,), (A, B, C))
0.0
```

##### (B)¶

```
>>> network = pyphi.examples.fig5b()
>>> state = (1, 0, 0)
>>> subsystem = pyphi.Subsystem(network, state)
>>> A, B, C = subsystem.node_indices
```

Symmetrically, \(A\) now has outputs, so its effect repertoire is selective and
it has effect information:

```
>>> subsystem.effect_repertoire((A,), (A, B, C))
array([[[0., 0.],
        [0., 0.]],

       [[0., 0.],
        [0., 1.]]])
>>> subsystem.effect_info((A,), (A, B, C))
0.5
```

But because it now has no inputs, its cause repertoire is no different from the
unconstrained effect repertoire, so it has no cause information:

```
>>> np.array_equal(subsystem.cause_repertoire((A,), (A, B, C)),
...                subsystem.unconstrained_cause_repertoire((A, B, C)))
True
>>> subsystem.cause_info((A,), (A, B, C))
0.0
```

And its cause effect information is again zero.

```
>>> subsystem.cause_effect_info((A,), (A, B, C))
0.0
```

#### Figure 6¶

**Integrated information: The information generated by the whole that is
irreducible to the information generated by its parts.**

```
>>> network = pyphi.examples.fig6()
>>> state = (1, 0, 0)
>>> subsystem = pyphi.Subsystem(network, state)
>>> ABC = subsystem.node_indices
```

Here we demonstrate the functions that find the minimum information partition a
mechanism over a purview:

```
>>> mip_c = subsystem.cause_mip(ABC, ABC)
>>> mip_e = subsystem.effect_mip(ABC, ABC)
```

These objects contain the \(\varphi^{\textrm{MIP}}\_{\textrm{cause}}\) and
\(\varphi^{\textrm{MIP}}\_{\textrm{effect}}\) values in their respective
`phi` attributes, and the minimal partitions in their `partition`
attributes:

```
>>> mip_c.phi
0.499999
>>> mip_c.partition  
 A     B,C
─── ✕ ─────
 ∅    A,B,C
>>> mip_e.phi
0.25
>>> mip_e.partition  
 ∅    A,B,C
─── ✕ ─────
 B     A,C
```

For more information on these objects, see the documentation for the
`RepertoireIrreducibilityAnalysis` class, or use `help(mip_c)`.

Note that the minimal partition found for the cause is

\[\frac{A^{c}}{\varnothing} \times \frac{BC^{c}}{ABC^{p}},\]

rather than the one shown in the figure. However, both partitions result in a
difference of \(0.5\) between the unpartitioned and partitioned cause
repertoires. So we see that in small networks like this, there can be multiple
choices of partition that yield the same, minimal
\(\varphi^{\textrm{MIP}}\). In these cases, which partition the software
chooses is left undefined.

#### Figure 7¶

**A mechanism generates integrated information only if it has both integrated
causes and integrated effects.**

It is left as an exercise for the reader to use the subsystem methods
`cause_mip` and `effect_mip`, introduced in the previous section, to
demonstrate the points made in Figure 7.

To avoid building TPMs and connectivity matrices by hand, you can use the
graphical user interface for PyPhi available online at
http://integratedinformationtheory.org/calculate.html. You can build the
networks shown in the figure there, and then use the **Export** button to
obtain a JSON file representing the
network. You can then import the file into Python like so:

```
network = pyphi.network.from_json('path/to/network.json')
```

#### Figure 8¶

**The maximally integrated cause repertoire over the power set of purviews is
the “core cause” specified by a mechanism.**

```
>>> network = pyphi.examples.fig8()
>>> state = (1, 0, 0)
>>> subsystem = pyphi.Subsystem(network, state)
>>> A, B, C = subsystem.node_indices
```

In PyPhi, the “core cause” is called the *maximally-irreducible cause* (MIC).
To find the MIC of a mechanism over all purviews, use the `mic()`
method:

```
>>> mic = subsystem.mic((B, C))
>>> mic.phi
0.333334
```

Similarly, the `mie()` method returns the “core effect” or
*maximally-irreducible effect* (MIE).

For a detailed description of the MIC and MIE objects returned by these
methods, see the documentation for `MaximallyIrreducibleCause` or use `help(subsystem.mic)` and
`help(subsystem.mie)`.

#### Figure 9¶

**A mechanism that specifies a maximally irreducible cause-effect repertoire.**

This figure and the next few use the same network as in Figure 8, so we don’t
need to reassign the `network` and `subsystem` variables.

Together, the MIC and MIE of a mechanism specify a *concept*. In PyPhi, this is
represented by the `Concept` object. Concepts are computed using the
`concept()` method of a subsystem:

```
>>> concept_A = subsystem.concept((A,))
>>> concept_A.phi
0.166667
```

As usual, please consult the documentation or use `help(concept_A)` for a
detailed description of the `Concept` object.

#### Figure 10¶

**Information: A conceptual structure C (constellation of concepts) is the set
of all concepts generated by a set of elements in a state.**

For functions of entire subsystems rather than mechanisms within them, we use
the `compute` module. In this figure, we see the constellation of concepts of
the powerset of \(ABC\)‘s mechanisms. A constellation of concepts is
represented in PyPhi by a `CauseEffectStructure`. We can compute the
cause-effect structure of the subsystem like so:

```
>>> ces = pyphi.compute.ces(subsystem)
```

And verify that the \(\varphi\) values match:

```
>>> ces.labeled_mechanisms
(['A'], ['B'], ['C'], ['A', 'B'], ['B', 'C'], ['A', 'B', 'C'])
>>> ces.phis
[0.166667, 0.166667, 0.25, 0.25, 0.333334, 0.499999]
```

The null concept (the small black cross shown in concept-space) is available as
an attribute of the subsystem:

```
>>> subsystem.null_concept.phi
0.0
```

#### Figure 11¶

**Assessing the conceptual information CI of a conceptual structure
(constellation of concepts).**

Conceptual information can be computed using the function named, as you might
expect, `conceptual_info()`:

```
>>> pyphi.compute.conceptual_info(subsystem)
2.111109
```

#### Figure 12¶

**Assessing the integrated conceptual information Φ of a constellation C.**

To calculate \(\Phi^{\textrm{MIP}}\) for a candidate set, we use the
function `sia()`:

```
>>> sia = pyphi.compute.sia(subsystem)
```

The returned value is a large object containing the \(\Phi^{\textrm{MIP}}\)
value, the minimal cut, the cause-effect structure of the whole set and that of
the partitioned set \(C\_{\rightarrow}^{\textrm{MIP}}\), the total
calculation time, the calculation time for just the unpartitioned cause-effect
structure, a reference to the subsystem that was analyzed, and a reference to
the subsystem with the minimal unidirectional cut applied. For details see the
documentation for `SystemIrreducibilityAnalysis` or use `help(sia)`.

We can verify that the \(\Phi^{\textrm{MIP}}\) value and minimal cut are as
shown in the figure:

```
>>> sia.phi
1.916665
>>> sia.cut
Cut [A, B] ━━/ /━━➤ [C]
```

Note

This `Cut` represents removing any connections from the nodes with
indices `0` and `1` to the node with index `2`.

#### Figure 13¶

**A set of elements generates integrated conceptual information Φ only if each
subset has both causes and effects in the rest of the set.**

It is left as an exercise for the reader to demonstrate that of the networks
shown, only **(B)** has \(\Phi > 0\).

#### Figure 14¶

**A complex: A local maximum of integrated conceptual information Φ.**

```
>>> network = pyphi.examples.fig14()
>>> state = (1, 0, 0, 0, 1, 0)
```

To find the subsystem within a network that is the major complex, we use the
function of that name, which returns a `SystemIrreducibilityAnalysis` object:

```
>>> major_complex = pyphi.compute.major_complex(network, state)
```

And we see that the nodes in the complex are indeed \(A\), \(B\), and \(C\):

```
>>> major_complex.subsystem.nodes
(A, B, C)
```

#### Figure 15¶

**A quale: The maximally irreducible conceptual structure (MICS) generated by a
complex.**

You can use the visual interface at
http://integratedinformationtheory.org/calculate.html to view a conceptual
structure structure in a 3D projection of qualia space. The network in the
figure is already built for you; click the **Load Example** button and select
“IIT 3.0 Paper, Figure 1” (this network is the same as the candidate set in
Figure 1).

#### Figure 16¶

**A system can condense into a major complex and minor complexes that may or
may not interact with it.**

For this figure, we omit nodes \(H\), \(I\), \(J\), \(K\) and
\(L\), since the TPM of the full 12-node network is very large, and the
point can be illustrated without them.

```
>>> network = pyphi.examples.fig16()
>>> state = (1, 0, 0, 1, 1, 1, 0)
```

To find the maximal set of non-overlapping complexes that a network condenses
into, use `condensed()`:

```
>>> condensed = pyphi.compute.condensed(network, state)
```

We find that there are two complexes: the major complex \(ABC\) with \(\Phi
\approx 1.92\), and a minor complex \(FG\) with \(\Phi \approx 0.069\) (note
that there is typo in the figure: \(FG\)‘s \(\Phi\) value should be \(0.069\)).
Furthermore, the program has been updated to only consider background
conditions of current states, not previous states; as a result the minor
complex \(DE\) shown in the paper no longer exists.

```
>>> len(condensed)
2
>>> ABC, FG = condensed
>>> (ABC.subsystem.nodes, ABC.phi)
((A, B, C), 1.916665)
>>> (FG.subsystem.nodes, FG.phi)
((F, G), 0.069445)
```

There are several other functions available for working with complexes; see the
documentation for `subsystems()`, `all_complexes()`,
`possible_complexes()`, and `complexes()`.

### Conditional Independence¶

Conditional independence is the property of a TPM that *each node’s state at
time* \(t+1\) *must be independent of the state of the others, given the state of
the network at time* \(t\):

\[\Pr(S\_{t+1} \mid S\_t = s\_t) \;=
\prod\_{N \,\in\, S} \Pr(N\_{t+1} \mid S\_t = s\_t)\;,
\quad \forall \; s\_t \in S.\]

This example explores the assumption of conditional independence, and the
behaviour of the program when it is not satisfied.

Every state-by-node TPM corresponds to a unique state-by-state TPM which
satisfies the conditional independence property (see Transition probability matrix conventions for
a discussion of the different TPM forms). If a state-by-node TPM is given as
input for a `Network`, PyPhi assumes that it is from a system with the
corresponding conditionally independent state-by-state TPM.

When a state-by-state TPM is given as input for a `Network`, the state-by-state
TPM is first converted to a state-by-node TPM. PyPhi then assumes that the
system corresponds to the unique conditionally independent representation of
the state-by-node TPM.

Note

Every **deterministic** state-by-state TPM satisfies the conditional
independence property.

Consider a system of two binary nodes (\(A\) and \(B\)) which do not change if they
have the same value, but flip with probability 50% if they have different
values.

We’ll load the state-by-state TPM for such a system from the `examples` module:

```
>>> import pyphi
>>> tpm = pyphi.examples.cond_depend_tpm()
>>> print(tpm)
[[1.  0.  0.  0. ]
 [0.  0.5 0.5 0. ]
 [0.  0.5 0.5 0. ]
 [0.  0.  0.  1. ]]
```

This system does not satisfy the conditional independence property; given a
previous state of `(1, 0)`, the current state of node \(A\) depends on whether
or not \(B\) has flipped.

If a conditionally dependent TPM is used to create a `Network`, PyPhi will
raise an error:

```
>>> network = pyphi.Network(tpm)
Traceback (most recent call last):
    ...
pyphi.exceptions.ConditionallyDependentError: TPM is not conditionally independent.
See the conditional independence example in the documentation for more info.
```

To see the conditionally independent TPM that corresponds to the conditionally
dependent TPM, convert it to state-by-node form and then back to state-by-state
form:

```
>>> sbn_tpm = pyphi.convert.state_by_state2state_by_node(tpm)
>>> print(sbn_tpm)
[[[0.  0. ]
  [0.5 0.5]]

 [[0.5 0.5]
  [1.  1. ]]]
>>> sbs_tpm = pyphi.convert.state_by_node2state_by_state(sbn_tpm)
>>> print(sbs_tpm)
[[1.   0.   0.   0.  ]
 [0.25 0.25 0.25 0.25]
 [0.25 0.25 0.25 0.25]
 [0.   0.   0.   1.  ]]
```

A system which does not satisfy the conditional independence property exhibits
“instantaneous causality.” In such situations, there must be additional
exogenous variable(s) which explain the dependence.

Now consider the above example, but with the addition of a third node (\(C\))
which is equally likely to be ON or OFF, and such that when nodes \(A\) and \(B\)
are in different states, they will flip when \(C\) is ON, but stay the same when
\(C\) is OFF.

```
>>> tpm2 = pyphi.examples.cond_independ_tpm()
>>> print(tpm2)
[[0.5 0.  0.  0.  0.5 0.  0.  0. ]
 [0.  0.5 0.  0.  0.  0.5 0.  0. ]
 [0.  0.  0.5 0.  0.  0.  0.5 0. ]
 [0.  0.  0.  0.5 0.  0.  0.  0.5]
 [0.5 0.  0.  0.  0.5 0.  0.  0. ]
 [0.  0.  0.5 0.  0.  0.  0.5 0. ]
 [0.  0.5 0.  0.  0.  0.5 0.  0. ]
 [0.  0.  0.  0.5 0.  0.  0.  0.5]]
```

The resulting state-by-state TPM now satisfies the conditional independence
property.

```
>>> sbn_tpm2 = pyphi.convert.state_by_state2state_by_node(tpm2)
>>> print(sbn_tpm2)
[[[[0.  0.  0.5]
   [0.  0.  0.5]]

  [[0.  1.  0.5]
   [1.  0.  0.5]]]


 [[[1.  0.  0.5]
   [0.  1.  0.5]]

  [[1.  1.  0.5]
   [1.  1.  0.5]]]]
```

The node indices are `0` and `1` for \(A\) and \(B\), and `2` for \(C\):

```
>>> AB = [0, 1]
>>> C = [2]
```

From here, if we marginalize out the node \(C\);

```
>>> tpm2_marginalizeC = pyphi.tpm.marginalize_out(C, sbn_tpm2)
```

And then restrict the purview to only nodes \(A\) and \(B\);

```
>>> import numpy as np
>>> tpm2_purviewAB = np.squeeze(tpm2_marginalizeC[:,:,:,AB])
```

We get back the original state-by-node TPM from the system with just \(A\) and
\(B\).

```
>>> np.all(tpm2_purviewAB == sbn_tpm)
True
```

### XOR Network¶

This example describes a system of three fully connected XOR nodes, \(A\), \(B\)
and \(C\) (no self-connections).

First let’s create the XOR network:

```
>>> import pyphi
>>> network = pyphi.examples.xor_network()
```

We’ll consider the state with all nodes OFF.

```
>>> state = (0, 0, 0)
```

According to IIT, existence is a holistic notion; the whole is more important
than its parts. The first step is to confirm the existence of the whole, by
finding the major complex of the network:

```
>>> major_complex = pyphi.compute.major_complex(network, state)
```

The major complex exists (\(\Phi > 0\)),

```
>>> major_complex.phi
1.874999
```

and it consists of the entire network:

```
>>> major_complex.subsystem
Subsystem(A, B, C)
```

Knowing what exists at the system level, we can now investigate the existence
of concepts within the complex.

```
>>> ces = major_complex.ces
>>> len(ces)
3
>>> ces.labeled_mechanisms
(['A', 'B'], ['A', 'C'], ['B', 'C'])
```

There are three concepts in the cause-effect structure. They are all the
possible second order mechanisms: \(AB\), \(AC\) and \(BC\).

Focusing on the concept specified by mechanism \(AB\), we investigate existence,
and the irreducible cause and effect. Based on the symmetry of the network, the
results will be similar for the other second order mechanisms.

```
>>> concept = ces[0]
>>> concept.mechanism
(0, 1)
>>> concept.phi
0.5
```

The concept has \(\varphi = \frac{1}{2}\).

```
>>> concept.cause.purview
(0, 1, 2)
>>> concept.cause.repertoire
array([[[0.5, 0. ],
        [0. , 0. ]],

       [[0. , 0. ],
        [0. , 0.5]]])
```

So we see that the cause purview of this mechanism is the whole system \(ABC\),
and that the repertoire shows a \(0.5\) of probability the previous state
being `(0, 0, 0)` and the same for `(1, 1, 1)`:

```
>>> concept.cause.repertoire[(0, 0, 0)]
0.5
>>> concept.cause.repertoire[(1, 1, 1)]
0.5
```

This tells us that knowing both \(A\) and \(B\) are currently OFF means that
the previous state of the system was either all OFF or all ON with equal
probability.

For any reduced purview, we would still have the same information about the
elements in the purview (either all ON or all OFF), but we would lose
the information about the elements outside the purview.

```
>>> concept.effect.purview
(2,)
>>> concept.effect.repertoire
array([[[1., 0.]]])
```

The effect purview of this concept is the node \(C\). The mechanism \(AB\) is able
to completely specify the next state of \(C\). Since both nodes are OFF, the
next state of \(C\) will be OFF.

The mechanism \(AB\) does not provide any information about the next state of
either \(A\) or \(B\), because the relationship depends on the value of \(C\). That
is, the next state of \(A\) (or \(B\)) may be either ON or OFF, depending
on the value of \(C\). Any purview larger than \(C\) would be reducible by pruning
away the additional elements.

| Major Complex: \(ABC\) with \(\Phi = 1.875\) | | | |
| --- | --- | --- | --- |
| Mechanism | \(\varphi\) | Cause Purview | Effect Purview |
| \(AB\) | 0.5 | \(ABC\) | \(C\) |
| \(AC\) | 0.5 | \(ABC\) | \(B\) |
| \(BC\) | 0.5 | \(ABC\) | \(A\) |

An analysis of the intrinsic existence of this system reveals that the major
complex of the system is the entire network of XOR nodes. Furthermore, the
concepts which exist within the complex are those specified by the second-order
mechanisms \(AB\), \(AC\), and \(BC\).

To understand the notion of intrinsic existence, in addition to determining
what exists for the system, it is useful to consider also what does not exist.

Specifically, it may be surprising that none of the first order mechanisms \(A\),
\(B\) or \(C\) exist. This physical system of XOR gates is sitting on the table in
front of me; I can touch the individual elements of the system, so how can it
be that they do not exist?

That sort of existence is what we term extrinsic existence. The XOR gates
exist for me as an observer, external to the system. I am able to manipulate
them, and observe their causes and effects, but the question that matters for
intrinsic existence is, do they have irreducible causes and effects within
the system? There are two reasons a mechanism may have no irreducible
cause-effect power: either the cause-effect power is completely reducible, or
there was no cause-effect power to begin with. In the case of elementary
mechanisms, it must be the latter.

To see this, again due to symmetry of the system, we will focus only on the
mechanism \(A\).

```
>>> subsystem = pyphi.examples.xor_subsystem()
>>> A = (0,)
>>> ABC = (0, 1, 2)
```

In order to exist, a mechanism must have irreducible cause and effect power
within the system.

```
>>> subsystem.cause_info(A, ABC)
0.5
>>> subsystem.effect_info(A, ABC)
0.0
```

The mechanism has no effect power over the entire subsystem, so it cannot have
effect power over any purview within the subsystem. Furthermore, if a mechanism
has no effect power, it certainly has no irreducible effect power. The
first-order mechanisms of this system do not exist intrinsically, because they
have no effect power (having causal power is not enough).

To see why this is true, consider the effect of \(A\). There is no self-loop, so
\(A\) can have no effect on itself. Without knowing the current state of \(A\), in
the next state \(B\) could be either ON or OFF. If we know that the current state
of \(A\) is ON, then \(B\) could still be either ON or OFF, depending on the state
of \(C\). Thus, on its own, the current state of \(A\) does not provide any
information about the next state of \(B\). A similar result holds for the effect
of \(A\) on \(C\). Since \(A\) has no effect power over any element of the system, it
does not exist from the intrinsic perspective.

To complete the discussion, we can also investigate the potential third order
mechanism \(ABC\). Consider the cause information over the purview \(ABC\):

```
>>> subsystem.cause_info(ABC, ABC)
0.749999
```

Since the mechanism has nonzero cause information, it has causal power over the
system—but is it irreducible?

```
>>> mip = subsystem.cause_mip(ABC, ABC)
>>> mip.phi
0.0
>>> mip.partition  
 A     B,C
─── ✕ ─────
 ∅    A,B,C
```

The mechanism has \(ci = 0.75\), but it is completely reducible
(\(\varphi = 0\)) to the partition

\[\frac{A}{\varnothing} \times \frac{BC}{ABC}\]

This result can be understood as follows: knowing that \(B\) and \(C\) are OFF in
the current state is sufficient to know that \(A\), \(B\), and \(C\) were all OFF in
the previous state; there is no additional information gained by knowing that
\(A\) is currently OFF.

Similarly for any other potential purview, the current state of \(B\) and \(C\)
being `(0, 0)` is always enough to fully specify the previous state, so the
mechanism is reducible for all possible purviews, and hence does not exist.

### Emergence (coarse-graining and blackboxing)¶

#### Coarse-graining¶

We’ll use the `macro` module to explore alternate spatial scales of a network.
The network under consideration is a 4-node non-deterministic network,
available from the `examples` module.

```
>>> import pyphi
>>> network = pyphi.examples.macro_network()
```

The connectivity matrix is all-to-all:

```
>>> network.cm
array([[1., 1., 1., 1.],
       [1., 1., 1., 1.],
       [1., 1., 1., 1.],
       [1., 1., 1., 1.]])
```

We’ll set the state so that nodes are OFF.

```
>>> state = (0, 0, 0, 0)
```

At the “micro” spatial scale, we can compute the major complex, and determine
the \(\Phi\) value:

```
>>> major_complex = pyphi.compute.major_complex(network, state)
>>> major_complex.phi
0.113889
```

The question is whether there are other spatial scales which have greater
values of \(\Phi\). This is accomplished by considering all possible
coarse-graining of micro-elements to form macro-elements. A coarse-graining of
nodes is any partition of the elements of the micro system. First we’ll get a
list of all possible coarse-grainings:

```
>>> grains = list(pyphi.macro.all_coarse_grains(network.node_indices))
```

We start by considering the first coarse grain:

```
>>> coarse_grain = grains[0]
```

Each `CoarseGrain` has two attributes: the `partition` of states into macro
elements, and the `grouping` of micro-states into macro-states. Let’s first
look at the partition:

```
>>> coarse_grain.partition
((0, 1, 2), (3,))
```

There are two macro-elements in this partition: one consists of micro-elements
`(0, 1, 2)` and the other is simply micro-element `3`.

We must then determine the relationship between micro-elements and
macro-elements. When coarse-graining the system we assume that the resulting
macro-elements do not differentiate the different micro-elements. Thus any
correspondence between states must be stated solely in terms of the number of
micro-elements which are ON, and not depend on which micro-elements are ON.

For example, consider the macro-element `(0, 1, 2)`. We may say that the
macro-element is ON if at least one micro-element is ON, or if all
micro-elements are ON; however, we may not say that the macro-element is ON if
micro-element `1` is ON, because this relationship involves identifying
specific micro-elements.

The `grouping` attribute of the `CoarseGrain` describes how the state of
micro-elements describes the state of macro-elements:

```
>>> grouping = coarse_grain.grouping
>>> grouping
(((0, 1, 2), (3,)), ((0,), (1,)))
```

The grouping consists of two lists, one for each macro-element:

```
>>> grouping[0]
((0, 1, 2), (3,))
```

For the first macro-element, this grouping means that the element will be OFF
if zero, one or two of its micro-elements are ON, and will be ON if all three
micro-elements are ON.

```
>>> grouping[1]
((0,), (1,))
```

For the second macro-element, the grouping means that the element will be OFF
if its micro-element is OFF, and ON if its micro-element is ON.

One we have selected a partition and grouping for analysis, we can create a
mapping between micro-states and macro-states:

```
>>> mapping = coarse_grain.make_mapping()
>>> mapping
array([0, 0, 0, 0, 0, 0, 0, 1, 2, 2, 2, 2, 2, 2, 2, 3])
```

The interpretation of the mapping uses the little-endian convention of indexing
(see Little-endian convention).

```
>>> mapping[7]
1
```

This says that micro-state 7 corresponds to macro-state 1:

```
>>> pyphi.convert.le_index2state(7, 4)
(1, 1, 1, 0)
```

```
>>> pyphi.convert.le_index2state(1, 2)
(1, 0)
```

In micro-state 7, all three elements corresponding to the first macro-element
are ON, so that macro-element is ON. The micro-element corresponding to the
second macro-element is OFF, so that macro-element is OFF.

The `CoarseGrain` object uses the mapping internally to create a state-by-state
TPM for the macro-system corresponding to the selected partition and grouping

```
>>> coarse_grain.macro_tpm(network.tpm)
Traceback (most recent call last):
    ...
pyphi.exceptions.ConditionallyDependentError...
```

However, this macro-TPM does not satisfy the conditional independence
assumption, so this particular partition and grouping combination is not a
valid coarse-graining of the system. Constructing a `MacroSubsystem` with this
coarse-graining will also raise a `ConditionallyDependentError`.

Let’s consider a different coarse-graining instead.

```
>>> coarse_grain = grains[14]
>>> coarse_grain.partition
((0, 1), (2, 3))
>>> coarse_grain.grouping
(((0, 1), (2,)), ((0, 1), (2,)))
```

```
>>> mapping = coarse_grain.make_mapping()
>>> mapping
array([0, 0, 0, 1, 0, 0, 0, 1, 0, 0, 0, 1, 2, 2, 2, 3])
```

```
>>> coarse_grain.macro_tpm(network.tpm)
array([[[0.09, 0.09],
        [1.  , 0.09]],

       [[0.09, 1.  ],
        [1.  , 1.  ]]])
```

We can now construct a `MacroSubsystem` using this coarse-graining:

```
>>> macro_subsystem = pyphi.macro.MacroSubsystem(
...     network, state, coarse_grain=coarse_grain)
>>> macro_subsystem
MacroSubsystem((m0, m1))
```

We can then consider the integrated information of this macro-network and
compare it to the micro-network.

```
>>> macro_sia = pyphi.compute.sia(macro_subsystem)
>>> macro_sia.phi
0.597212
```

The integrated information of the macro subsystem (\(\Phi = 0.597212\)) is
greater than the integrated information of the micro system (\(\Phi =
0.113889\)). We can conclude that a macro-scale is appropriate for this system,
but to determine which one, we must check all possible partitions and all
possible groupings to find the maximum of integrated information across all
scales.

```
>>> M = pyphi.macro.emergence(network, state)
>>> M.emergence
0.483323
>>> M.system
(0, 1, 2, 3)
>>> M.coarse_grain.partition
((0, 1), (2, 3))
>>> M.coarse_grain.grouping
(((0, 1), (2,)), ((0, 1), (2,)))
```

The analysis determines the partition and grouping which results in the maximum
value of integrated information, as well as the emergence (increase in
\(\Phi\)) from the micro-scale to the macro-scale.

#### Blackboxing¶

- `pyphi.examples.blackbox_network()`

The `macro` module also provides tools for studying the emergence of systems
using blackboxing.

```
>>> import pyphi
>>> network = pyphi.examples.blackbox_network()
```

We consider the state where all nodes are OFF:

```
>>> state = (0, 0, 0, 0, 0, 0)
```

The system has minimal \(\Phi\) without blackboxing:

```
>>> subsys = pyphi.Subsystem(network, state)
>>> pyphi.compute.phi(subsys)
0.215278
```

We will consider the blackbox system consisting of two blackbox elements, \(ABC\)
and \(DEF\), where \(C\) and \(F\) are output elements and \(AB\) and \(DE\) are hidden
within their respective blackboxes.

Blackboxing is done with a `Blackbox` object. As with `CoarseGrain`, we pass it
a partition of micro-elements:

```
>>> partition = ((0, 1, 2), (3, 4, 5))
>>> output_indices = (2, 5)
>>> blackbox = pyphi.macro.Blackbox(partition, output_indices)
```

Blackboxes have a few convenient attributes and methods. The `hidden_indices`
attribute returns the elements which are hidden within blackboxes:

```
>>> blackbox.hidden_indices
(0, 1, 3, 4)
```

The `micro_indices` attribute lists all the micro-elements in the box:

```
>>> blackbox.micro_indices
(0, 1, 2, 3, 4, 5)
```

The `macro_indices` attribute generates a set of indices which index the
blackbox macro-elements. Since there are two blackboxes in our example, and
each has one output element, there are two macro-indices:

```
>>> blackbox.macro_indices
(0, 1)
```

The `macro_state` method converts a state of the micro elements to the state
of the macro-elements. The macro-state of a blackbox system is simply the state
of the system’s output elements:

```
>>> micro_state = (0, 0, 0, 0, 0, 1)
>>> blackbox.macro_state(micro_state)
(0, 1)
```

Let us also define a time scale over which to perform our analysis:

```
>>> time_scale = 2
```

As in the coarse-graining example, the blackbox and time scale are passed to
`MacroSubsystem`:

```
>>> macro_subsystem = pyphi.macro.MacroSubsystem(network, state,
...                                              blackbox=blackbox,
...                                              time_scale=time_scale)
```

We can now compute \(\Phi\) for this macro system:

```
>>> pyphi.compute.phi(macro_subsystem)
0.638888
```

We find that the macro subsystem has greater integrated information
(\(\Phi = 0.638888\)) than the micro system (\(\Phi = 0.215278\))—the
system demonstrates emergence.

### Actual Causation¶

This section demonstrates how to use PyPhi to evaluate actual causation as
described in

Albantakis L, Marshall W, Hoel E, Tononi G (2017). What caused what? An
irreducible account of actual causation. arXiv:1708.06716 [cs.AI].

```
>>> import pyphi
>>> from pyphi import actual, config, Direction
```

#### Configuration¶

Before we begin we need to set some configuration values. The correct way of
partitioning for actual causation is using the `'ALL'` partitions setting;
`'TRI'`-partitions are a reasonable approximation. In case of ties the
smaller purview should be chosen. IIT 3.0 style bipartitions will give
incorrect results.

```
>>> config.PARTITION_TYPE = 'TRI'
>>> config.PICK_SMALLEST_PURVIEW = True
```

When calculating a causal account of the transition between a set of elements
\(X\) at time \(t-1\) and a set of elements \(Y\) at time \(t\), with \(X\) and \(Y\) being
subsets of the same system, the transition should be valid according to the
system’s TPM. However, the state of \(X\) at \(t-1\) does not necessarily need to
have a valid previous state so we can disable state validation:

```
>>> config.VALIDATE_SUBSYSTEM_STATES = False
```

#### Computation¶

We will look at how to perform computations over the basic OR-AND network
introduced in Figure 1 of the paper.

```
>>> network = pyphi.examples.actual_causation()
```

This is a standard PyPhi `Network` so we can look at its TPM:

```
>>> pyphi.convert.state_by_node2state_by_state(network.tpm)
array([[1., 0., 0., 0.],
       [0., 1., 0., 0.],
       [0., 1., 0., 0.],
       [0., 0., 0., 1.]])
```

The `OR` gate is element `0`, and the `AND` gate is element `1` in the
network.

```
>>> OR = 0
>>> AND = 1
```

We want to observe both elements at \(t-1\) and \(t\), with `OR` ON and `AND`
OFF in both observations:

```
>>> X = Y = (OR, AND)
>>> X_state = Y_state = (1, 0)
```

The `Transition` object is the core of all actual causation calculations. To
instantiate a `Transition`, we pass it a `Network`, the state of the network at
\(t-1\) and \(t\), and elements of interest at \(t-1\) and \(t\). Note that PyPhi
requires the state to be the state of the entire network, not just the state of
the nodes in the transition.

```
>>> transition = actual.Transition(network, X_state, Y_state, X, Y)
```

Cause and effect repertoires can be obtained for the transition. For example,
as shown on the right side of Figure 2B, we can compute the effect repertoire
to see how \(X\_{t-1} = \{OR = 1\}\) constrains the probability distribution of the
purview \(Y\_t = \{OR, AND\}\):

```
>>> transition.effect_repertoire((OR,), (OR, AND))
array([[0. , 0. ],
       [0.5, 0.5]])
```

Similarly, as in Figure 2C, we can compute the cause repertoire of
\(Y\_t = \{OR, AND = 10\}\) to see how it constrains the purview \(X\_{t-1} = \{OR\}\):

```
>>> transition.cause_repertoire((OR, AND), (OR,))
array([[0.5],
       [0.5]])
```

Note

In all `Transition` methods the constraining occurence is passed as
the `mechanism` argument and the constrained occurence is the `purview`
argument. This mirrors the terminology introduced in the IIT code.

`Transition` also provides methods for computing cause and effect
ratios. For example, the effect ratio of \(X\_{t-1} = \{OR = 1\}\) constraining
\(Y\_t = \{OR\}\) (as shown in Figure 3A) is computed as follows:

```
>>> transition.effect_ratio((OR,), (OR,))
0.415037
```

The effect ratio of \(X\_{t-1} = \{OR = 1\}\) constraining \(Y\_t = \{AND\}\) is negative:

```
>>> transition.effect_ratio((OR,), (AND,))
-0.584963
```

And the cause ratio of \(Y\_t = \{OR = 1\}\) constraining \(X\_{t-1} = \{OR, AND\}\)
(Figure 3B) is:

```
>>> transition.cause_ratio((OR,), (OR, AND))
0.415037
```

We can evaluate \(\alpha\) for a particular pair of occurences, as in Figure 3C.
For example, to find the irreducible effect ratio of \(\{OR, AND\} \rightarrow \{OR, AND\}\),
we use the `find_mip` method:

```
>>> link = transition.find_mip(Direction.EFFECT, (OR, AND), (OR, AND))
```

This returns a `AcRepertoireIrreducibilityAnalysis` object, with a number of
useful properties. This particular MIP is reducible, as we can see by checking
the value of \(\alpha\):

```
>>> link.alpha
0.0
```

The `partition` property shows the minimum information partition that
reduces the occurence and candidate effect:

```
>>> link.partition  
 ∅     OR     AND
─── ✕ ─── ✕ ───
 ∅     OR     AND
```

Let’s look at the MIP for the irreducible occurence \(Y\_t = \{OR, AND\}\)
constraining \(X\_{t-1} = \{OR, AND\}\) (Figure 3D). This candidate causal link has
positive \(\alpha\):

```
>>> link = transition.find_mip(Direction.CAUSE, (OR, AND), (OR, AND))
>>> link.alpha
0.169925
```

To find the actual cause or actual effect of a particular occurence, use the
`find_actual_cause` or `find_actual_effect` methods:

```
>>> transition.find_actual_cause((OR, AND))
CausalLink
  α = 0.1699  [OR, AND] ◀━━ [OR, AND]
```

#### Accounts¶

The complete causal account of our transition can be computed with the
`account` function:

```
>>> account = actual.account(transition)
>>> print(account)  

      Account (5 causal links)
***********************************
Irreducible effects
α = 0.415  [OR] ━━▶ [OR]
α = 0.415  [AND] ━━▶ [AND]
Irreducible causes
α = 0.415  [OR] ◀━━ [OR]
α = 0.415  [AND] ◀━━ [AND]
α = 0.1699  [OR, AND] ◀━━ [OR, AND]
```

We see that this function produces the causal links shown in Figure 4. The
`Account` object is a subclass of `tuple`, and can manipulated the same:

```
>>> len(account)
5
```

#### Irreducible Accounts¶

The irreducibility of the causal account of our transition of interest can be
evaluated using the following function:

```
>>> sia = actual.sia(transition)
>>> sia.alpha
0.169925
```

As shown in Figure 4, the second order occurence \(Y\_t = \{OR, AND = 10\}\) is
destroyed by the MIP:

```
>>> sia.partitioned_account  

 Account (4 causal links)
**************************
Irreducible effects
α = 0.415  [OR] ━━▶ [OR]
α = 0.415  [AND] ━━▶ [AND]
Irreducible causes
α = 0.415  [OR] ◀━━ [OR]
α = 0.415  [AND] ◀━━ [AND]
```

The partition of the MIP is available in the `cut` property:

```
>>> sia.cut  
KCut CAUSE
 ∅     OR    AND
─── ✕ ─── ✕ ───
 ∅     OR    AND
```

To find all irreducible accounts within the transition of interest, use
`nexus`:

```
>>> all_accounts = actual.nexus(network, X_state, Y_state)
```

This computes \(\mathcal{A}\) for all permutations of of elements in \(X\_{t-1}\) and
\(Y\_t\) and returns a `tuple` of all `AcSystemIrreducibilityAnalysis` objects
with \(\mathcal{A} > 0\):

```
>>> for n in all_accounts:
...     print(n.transition, n.alpha)
Transition([OR] ━━▶ [OR]) 2.0
Transition([AND] ━━▶ [AND]) 2.0
Transition([OR, AND] ━━▶ [OR, AND]) 0.169925
```

The `causal_nexus` function computes the maximally irreducible account for
the transition of interest:

```
>>> cn = actual.causal_nexus(network, X_state, Y_state)
>>> cn.alpha
2.0
>>> cn.transition
Transition([OR] ━━▶ [OR])
```

#### Disjunction of conjunctions¶

If you are interested in exploring further, the disjunction of conjunctions
network from Figure 7 is provided as well:

```
>>> network = pyphi.examples.disjunction_conjunction_network()
>>> cn = actual.causal_nexus(network, (1, 0, 1, 0), (0, 0, 0, 1))
```

The only irreducible transition is from \(X\_{t-1} = C\) to \(Y\_t = D\), with
\(\mathcal{A}\) of 2.0:

```
>>> cn.transition
Transition([C] ━━▶ [D])
>>> cn.alpha
2.0
```

### Residue¶

This example describes a system containing two AND gates, \(A\) and \(B\), with a
single overlapping input node.

First let’s create the subsystem corresponding to the residue network, with all
nodes OFF in the current and previous states.

```
>>> import pyphi
>>> subsystem = pyphi.examples.residue_subsystem()
```

Next, we can define the mechanisms of interest. Mechanisms and purviews are
represented by tuples of node indices in the network:

```
>>> A = (0,)
>>> B = (1,)
>>> AB = (0, 1)
```

And the possible cause purviews that we’re interested in:

```
>>> CD = (2, 3)
>>> DE = (3, 4)
>>> CDE = (2, 3, 4)
```

We can then evaluate the cause information for each of the mechanisms over the
cause purview \(CDE\).

```
>>> subsystem.cause_info(A, CDE)
0.333332
```

```
>>> subsystem.cause_info(B, CDE)
0.333332
```

```
>>> subsystem.cause_info(AB, CDE)
0.5
```

The composite mechanism \(AB\) has greater cause information than either of the
individual mechanisms. This contradicts the idea that \(AB\) should exist
minimally in this system.

Instead, we can quantify existence as the irreducible cause information of a
mechanism. The MIP of a mechanism is the partition of mechanism and purview
which makes the least difference to the cause repertoire (see the documentation
for the `RepertoireIrreducibilityAnalysis` object). The irreducible cause
information is the distance between the unpartitioned and partitioned
repertoires.

To analyze the irreducibility of the mechanism \(AB\) on the cause side:

```
>>> mip_AB = subsystem.cause_mip(AB, CDE)
```

We can then determine what the specific partition is.

```
>>> mip_AB.partition  
 ∅    A,B
─── ✕ ───
 C    D,E
```

The indices `(0, 1, 2, 3, 4)` correspond to nodes \(A, B, C, D, E\)
respectively. Thus the MIP is \(\frac{AB}{DE} \times \frac{\varnothing}{C}\), where \(\varnothing\)
denotes the empty mechanism.

The partitioned repertoire of the MIP can also be retrieved:

```
>>> mip_AB.partitioned_repertoire
array([[[[[0.2, 0.2],
          [0.1, 0. ]],

         [[0.2, 0.2],
          [0.1, 0. ]]]]])
```

And we can then calculate the irreducible cause information as the difference
between partitioned and unpartitioned repertoires.

```
>>> mip_AB.phi
0.1
```

One counterintuitive result that merits discussion is that since irreducible
cause information is what defines existence, we must also evaluate the
irreducible cause information of the mechanisms \(A\) and \(B\).

The mechanism \(A\) over the purview \(CDE\) is completely reducible to
\(\frac{A}{CD} \times \frac{\varnothing}{E}\) because \(E\) has no effect on \(A\), so it has zero
\(\varphi\).

```
>>> subsystem.cause_mip(A, CDE).phi
0.0
>>> subsystem.cause_mip(A, CDE).partition  
 ∅     A
─── ✕ ───
 E    C,D
```

Instead, we should evaluate \(A\) over the purview \(CD\).

```
>>> mip_A = subsystem.cause_mip(A, CD)
```

In this case, there is a well-defined MIP

```
>>> mip_A.partition  
 ∅     A
─── ✕ ───
 C     D
```

which is \(\frac{\varnothing}{C} \times \frac{A}{D}\). It has partitioned repertoire

```
>>> mip_A.partitioned_repertoire
array([[[[[0.33333333],
          [0.16666667]],

         [[0.33333333],
          [0.16666667]]]]])
```

and irreducible cause information

```
>>> mip_A.phi
0.166667
```

A similar result holds for \(B\). Thus the mechanisms \(A\) and \(B\) exist at levels
of \(\varphi = \frac{1}{6}\), while the higher-order mechanism \(AB\) exists only as the
residual of causes, at a level of \(\varphi = \frac{1}{10}\).

### Magic Cuts¶

This example explores a system of three fully connected elements \(A\), \(B\) and
\(C\), which follow the logic of the Rule 110 cellular automaton. The point of
this example is to highlight an unexpected behaviour of system cuts: that the
minimum information partition of a system can result in new concepts being
created.

First let’s create the the Rule 110 network, with all nodes OFF in the current
state.

```
>>> import pyphi
>>> network = pyphi.examples.rule110_network()
>>> state = (0, 0, 0)
```

Next, we want to identify the spatial scale and major complex of the network:

```
>>> macro = pyphi.macro.emergence(network, state)
>>> print(macro.emergence)
-1.112671
```

Since the emergence value is negative, there is no macro scale which has
greater integrated information than the original micro scale. We can now
analyze the micro scale to determine the major complex of the system:

```
>>> major_complex = pyphi.compute.major_complex(network, state)
>>> major_complex.subsystem
Subsystem(A, B, C)
>>> print(major_complex.phi)
1.35708
```

The major complex of the system contains all three nodes of the system, and it
has integrated information \(\Phi = 1.35708\). Now that we have identified
the major complex of the system, we can explore its cause-effect structure and
the effect of the MIP.

```
>>> ces = major_complex.ces
```

There two equivalent cuts for this system; for concreteness we sever all
connections from elements \(A\) and \(B\) to \(C\).

```
>>> cut = pyphi.models.Cut(from_nodes=(0, 1), to_nodes=(2,))
>>> cut_subsystem = pyphi.Subsystem(network, state, cut=cut)
>>> cut_ces = pyphi.compute.ces(cut_subsystem)
```

Let’s investigate the concepts in the unpartitioned cause-effect structure,

```
>>> ces.labeled_mechanisms
(['A'], ['B'], ['C'], ['A', 'B'], ['A', 'C'], ['B', 'C'])
>>> ces.phis
[0.125, 0.125, 0.125, 0.499999, 0.499999, 0.499999]
>>> sum(ces.phis)
1.8749970000000002
```

and also the concepts of the partitioned cause-effect structure.

```
>>> cut_ces.labeled_mechanisms
(['A'], ['B'], ['C'], ['A', 'B'], ['B', 'C'], ['A', 'B', 'C'])
>>> cut_ces.phis
[0.125, 0.125, 0.125, 0.499999, 0.266666, 0.333333]
>>> sum(_)
1.4749980000000003
```

The unpartitioned cause-effect structure includes all possible first and second
order concepts, but there is no third order concept. After applying the cut and
severing the connections from \(A\) and \(B\) to \(C\), the third order concept \(ABC\)
is created and the second order concept \(AC\) is destroyed. The overall amount
of \(\varphi\) in the system decreases from \(1.875\) to \(1.475\).

Let’s explore the concept which was created to determine why it does not exist
in the unpartitioned cause-effect structure and what changed in the partitioned
cause-effect structure.

```
>>> subsystem = major_complex.subsystem
>>> ABC = subsystem.node_indices
>>> subsystem.cause_info(ABC, ABC)
0.749999
>>> subsystem.effect_info(ABC, ABC)
1.875
```

The mechanism does have cause and effect power over the system. But, since it
doesn’t specify a concept, it must be that this power is reducible:

```
>>> mic = subsystem.mic(ABC)
>>> mic.phi
0.0
>>> mie = subsystem.mie(ABC)
>>> mie.phi
0.625
```

The reason ABC does not exist as a concept is that its cause is reducible.
Looking at the TPM of the system, there are no possible states where two
elements are OFF. This means that knowing two elements are OFF is enough to
know that the third element must also be OFF, and thus the third element can
always be cut from the concept without a loss of information. This will be true
for any purview, so the cause information is reducible.

```
>>> BC = (1, 2)
>>> A = (0,)
>>> repertoire = subsystem.cause_repertoire(ABC, ABC)
>>> cut_repertoire = (subsystem.cause_repertoire(BC, ABC) *
...                   subsystem.cause_repertoire(A, ()))
>>> pyphi.distance.hamming_emd(repertoire, cut_repertoire)
0.0
```

Next, let’s look at the cut subsystem to understand how the new concept comes
into existence.

```
>>> ABC = (0, 1, 2)
>>> C = (2,)
>>> AB = (0, 1)
```

The cut applied to the subsystem severs the connections going to \(C\) from
either \(A\) or \(B\). In this circumstance, knowing the state of \(A\) or \(B\) does
not tell us anything about the state of \(C\); only the previous state of \(C\) can
tell us about the next state of \(C\). `C_node.tpm_on` gives us the probability
of \(C\) being ON in the next state, while `C_node.tpm_off` would give us the
probability of \(C\) being OFF.

```
>>> C_node = cut_subsystem.indices2nodes(C)[0]
>>> C_node.tpm_on.flatten()
array([0.5 , 0.75])
```

This states that \(C\) has a 50% chance of being ON in the next state if it
currently OFF, but a 75% chance of being ON in the next state if it is
currently ON. Thus, unlike the unpartitioned case, knowing the current state of
\(C\) gives us additional information over and above knowing the state of \(A\) or
\(B\).

```
>>> repertoire = cut_subsystem.cause_repertoire(ABC, ABC)
>>> cut_repertoire = (cut_subsystem.cause_repertoire(AB, ABC) *
...                   cut_subsystem.cause_repertoire(C, ()))
>>> print(pyphi.distance.hamming_emd(repertoire, cut_repertoire))
0.500001
```

With this partition, the integrated information is \(\varphi = 0.5\), but
we must check all possible partitions to find the maximally-irreducible cause:

```
>>> mic = cut_subsystem.mic(ABC)
>>> mic.purview
(0, 1, 2)
>>> mic.phi
0.333333
```

It turns out that the MIP of the maximally-irreducible cause is

\[\frac{AB}{\varnothing} \times \frac{C}{ABC}\]

and the integrated information of mechanism \(ABC\) is \(\varphi = 1/3\).

Note that in order for a new concept to be created by a cut, there must be a
within-mechanism connection severed by the cut.

In the previous example, the MIP created a new concept, but the amount of
\(\varphi\) in the cause-effect structure still decreased. This is not always
the case. Next we will look at an example of system whoes MIP increases the
amount of \(\varphi\). This example is based on a five-node network that
implements the logic of the Rule 154 cellular automaton. Let’s first load the
network:

```
>>> network = pyphi.examples.rule154_network()
>>> state = (1, 0, 0, 0, 0)
```

For this example, it is the subsystem consisting of \(A\), \(B\), and \(E\) that we
explore. This is not the major complex of the system, but it serves as a proof
of principle regardless.

```
>>> subsystem = pyphi.Subsystem(network, state, (0, 1, 4))
```

Calculating the MIP of the system,

```
>>> sia = pyphi.compute.sia(subsystem)
>>> sia.phi
0.217829
>>> sia.cut
Cut [A, E] ━━/ /━━➤ [B]
```

we see that this subsystem has a \(\Phi\) value of 0.15533, and the MIP cuts
the connections from \(AE\) to \(B\). Investigating the concepts in both the
partitioned and unpartitioned cause-effect structures,

```
>>> sia.ces.labeled_mechanisms
(['A'], ['B'], ['A', 'B'])
>>> sia.ces.phis
[0.25, 0.166667, 0.178572]
>>> print(sum(_))
0.5952390000000001
```

We see that the unpartitioned cause-effect structure has mechanisms \(A\), \(B\)
and \(AB\) with \(\sum\varphi = 0.595239\).

```
>>> sia.partitioned_ces.labeled_mechanisms
(['A'], ['B'], ['A', 'B'])
>>> sia.partitioned_ces.phis
[0.25, 0.166667, 0.214286]
>>> print(sum(_))
0.630953
```

The partitioned cause-effect structure has mechanisms \(A\), \(B\) and \(AB\) but
with \(\sum\varphi = 0.630953\). There are the same number of concepts in
both cause-effect structures, over the same mechanisms; however, the
partitioned cause-effect structure has a greater \(\varphi\) value for the
concept \(AB\), resulting in an overall greater \(\sum\varphi\) for the
partitioned cause-effect structure.

Although situations described above are rare, they do occur, so one must be
careful when analyzing the integrated information of physical systems not to
dismiss the possibility of partitions creating new concepts or increasing the
amount of \(\varphi\); otherwise, an incorrect major complex may be identified.

### Detailed installation guide for macOS¶

This is a step-by-step guide intended for those unfamiliar with Python
or the command-line (*a.k.a.* the “shell”).

A shell can be opened by opening a new tab in the Terminal app (located in
Utilities). Text that is `formatted like code` is meant to be copied and
pasted into the terminal (hit the Enter key to run the command).

The fist step is to install the versions of Python that we need. The most
convenient way of doing this is to use the OS X package manager Homebrew. Install Homebrew by running this command:

```
/usr/bin/ruby -e "$(curl -fsSL https://raw.githubusercontent.com/Homebrew/install/master/install)"
```

Now you should have access to the `brew` command. First, we need to install
Python 2 and 3. Using these so-called “brewed” Python versions, rather than the
version of Python that comes with your computer, will protect your computer’s
Python version from unwanted changes that could interfere with other
applications.

```
brew install python python3
```

Then we need to ensure that the terminal “knows about” the newly-installed
Python versions:

```
brew link --overwrite python
brew link --overwrite python3
```

Now that we’re using our shiny new Python versions, it is highly recommended to
set up a **virtual environment** in which to install PyPhi. Virtual
environments allow different projects to isolate their dependencies from one
another, so that they don’t interact in unexpected ways. Please see this guide for more information.

To do this, you must install `virtualenvwrapper`, a tool for manipulating
virtual environments. This tool
is available on PyPI, the Python package
index, and can be installed with `pip`, the command-line utility for
installing and managing Python packages (`pip` was installed automatically
with the brewed Python):

```
pip install virtualenvwrapper
```

Now we need to edit your shell startup file. This is a file that runs
automatically every time you open a new shell (a new window or tab in the
Terminal app). This file should be in your home directory, though it will be
invisible in the Finder because the filename is preceded by a period. On most
Macs it is called `.bash_profile`. You can open this in a text editor by
running this command:

```
open -a TextEdit ~/.bash_profile
```

If you get an error that says the file doesn’t exist, then run `touch
~/.bash_profile` first to create it.

Now, you’ll add three lines to the shell startup file. These lines will set the
location where the virtual environments will live, the location of your
development project directories, and the location of the script installed with
this package, respectively. **Note:** The location of the script can be found
by running `which virtualenvwrapper.sh`.

The filepath after the equals sign on the second line will different for
everyone, but here is an example:

```
export WORKON_HOME=$HOME/.virtualenvs
export PROJECT_HOME=$HOME/dev
source /usr/local/bin/virtualenvwrapper.sh
```

After editing the startup file and saving it, open a new terminal shell by
opening a new tab or window (or just reload the startup file by running
`source ~/.bash_profile`).

Now that `virtualenvwrapper` is fully installed, use it to create a Python 3
virtual environment, like so:

```
mkvirtualenv -p `which python3` <name_of_your_project>
```

The option `` -p `which python3` `` ensures that when the virtual environment is
activated, the commands `python` and `pip` will refer to their Python 3
counterparts.

The virtual environment should have been activated automatically after creating
it. Virtual environments can be manually activated with `workon
<name_of_your_project>`, and deactivated with `deactivate`.

**Important:** Remember to activate the virtual environment with the `workon`
command **every time you begin working on your project**. Also, note that the
currently active virtual environment is *not* associated with any particular
folder; it is associated with a terminal shell. In other words, each time you
open a new Terminal tab or terminal window, you need to run `workon
<name_of_your_project` (with some extra setup, this can be done automatically;
see here).
When a virtual environment is active, your command-line prompt will be
prepended with the name of the virtual environment in parentheses.

Once you’ve checked that the new virtual environment is active, you’re finally
ready to install PyPhi into it (note that this may take a few minutes):

```
pip install pyphi
```

Congratulations, you’ve just installed PyPhi!

To play around with the software, ensure that you’ve activated the virtual
environment with `workon <name_of_your_project>`. Then run `python` to
start a Python 3 interpreter. Then, in the interpreter’s command-line (which is
preceded by the `>>>` prompt), run

```
import pyphi
```

Optionally, you can also install IPython with `pip
install ipython` to get a more useful Python interpreter that offers things
like tab-completion. Once you’ve installed it, you can start the IPython
interpreter with the command `ipython`.

Next, please see the documentation for some examples of how to use PyPhi and
information on how to configure it.

### Transition probability matrix conventions¶

A `Network` can be created with a transition probability matrix (TPM) in any of
the three forms described below. However, in PyPhi the canonical TPM
representation is **multidimensional state-by-node form**. The TPM will be
converted to this form when the `Network` is built.

Tip

Functions for converting TPMs from one form to another are available in the
`convert` module.

#### State-by-node form¶

A TPM in **state-by-node form** is a matrix where the entry \((i,j)\) gives the
probability that the \(j^{\textrm{th}}\) node will be ON at time \(t+1\) if the system is in
the \(i^{\textrm{th}}\) state at time \(t\).

#### Multidimensional state-by-node form¶

A TPM in **multidimensional state-by-node** form is a state-by-node form that
has been reshaped so that it has \(n+1\) dimensions instead of two. The first \(n\)
dimensions correspond to each of the \(n\) nodes at time \(t\), while the last
dimension corresponds to the probabilities of each node being ON at \(t+1\).

With this form, we can take advantage of NumPy array indexing and use a
network state as an index directly:

```
>>> from pyphi.examples import basic_noisy_selfloop_network
>>> tpm = basic_noisy_selfloop_network().tpm
>>> state = (0, 0, 1)  # A network state is a binary tuple
>>> tpm[state]
array([0.919, 0.91 , 0.756])
```

This tells us that if the current state is \(N\_0 = 0, N\_1 = 0, N\_2 = 1\), then
the for the next state, \(\Pr(N\_0 = 1) = 0.919\), \(\Pr(N\_1 = 1) =
0.91\) and \(\Pr(N\_2 = 1) = 0.756\).

Important

The multidimensional state-by-node form is used throughout PyPhi,
regardless of the form that was used to create the `Network`.

#### State-by-state form¶

A TPM in **state-by-state form** is a matrix where the entry \((i,j)\) gives the
probability that the state at time \(t+1\) will be \(j\) if the state at time \(t\)
is labeled by \(i\).

Warning

**When converting a state-by-state TPM to one of the other forms, information
may be lost!**

This is because the space of possible state-by-state TPMs is larger than
the space of state-by-node TPMs (so the conversion cannot be injective).
However, if we restrict the state-by-state TPMs to only those that satisfy
the conditional independence property, then the mapping becomes bijective.

See Conditional Independence for a more detailed discussion.

#### Little-endian convention¶

Even after choosing one of the above representations, there are several ways to
write down the TPM.

With both state-by-state and state-by-node TPMs, one is confronted with a
choice about which rows correspond to which states. In state-by-state TPMs,
this choice must also be made for the columns.

There are two possible choices for the rows. Either the first node changes
state every other row:

> | State at \(t\) | \(\Pr(N = ON)\) at \(t+1\) | |
> | --- | --- | --- |
> | A, B | A | B |
> | (0, 0) | 0.1 | 0.2 |
> | (1, 0) | 0.3 | 0.4 |
> | (0, 1) | 0.5 | 0.6 |
> | (1, 1) | 0.7 | 0.8 |

Or the last node does:

> | State at \(t\) | \(\Pr(N = ON)\) at \(t+1\) | |
> | --- | --- | --- |
> | A, B | A | B |
> | (0, 0) | 0.1 | 0.2 |
> | (0, 1) | 0.5 | 0.6 |
> | (1, 0) | 0.3 | 0.4 |
> | (1, 1) | 0.7 | 0.8 |

Note that the index \(i\) of a row in a TPM encodes a network state: convert the
index to binary, and each bit gives the state of a node. The question is, which
node?

**Throughout PyPhi, we always choose the first convention—the state of the
first node (the one with the lowest index) varies the fastest.** So, the
least-signficant bit—the one’s place—gives the state of the lowest-index
node.

This is analogous to the little-endian convention in organizing computer
memory. The other convention, where the highest-index node varies the fastest,
is analogous to the big-endian convention (see Endianness).

The rationale for this choice of convention is that the little-endian mapping
is stable under changes in the number of nodes, in the sense that the same bit
always corresponds to the same node index. The big-endian mapping does not have
this property.

Tip

Functions to convert states to indices and vice versa, according to either
the little-endian or big-endian convention, are available in the `convert`
module.

Note

This applies to only situations where decimal indices are encoding states.
Whenever a network state is represented as a list or tuple, we use the only
sensible convention: the \(i^{\textrm{th}}\) element gives the state of the \(i^{\textrm{th}}\) node.

### Connectivity matrix conventions¶

Throughout PyPhi, if \(CM\) is a connectivity matrix, then \([CM]\_{i,j} = 1\) means
that there is a directed edge \((i,j)\) from node \(i\) to node \(j\), and
\([CM]\_{i,j} = 0\) means there is no edge from \(i\) to \(j\).

For example, this network of four nodes

has the following connectivity matrix:

```
>>> cm = [[0, 0, 1, 0],
...       [1, 0, 1, 0],
...       [0, 1, 0, 1],
...       [0, 0, 0, 1]]
```

### Loading a configuration¶

Various aspects of PyPhi’s behavior can be configured.

When PyPhi is imported, it checks for a YAML file named `pyphi_config.yml` in
the current directory and automatically loads it if it exists; otherwise the
default configuration is used.

The various settings are listed here with their defaults.

```
>>> import pyphi
>>> defaults = pyphi.config.defaults()
```

Print the `config` object to see the current settings:

```
>>> print(pyphi.config)  
{ 'ASSUME_CUTS_CANNOT_CREATE_NEW_CONCEPTS': False,
  'CACHE_SIAS': False,
  'CACHE_POTENTIAL_PURVIEWS': True,
  'CACHING_BACKEND': 'fs',
  ...
```

Setting can be changed on the fly by assigning them a new value:

```
>>> pyphi.config.PROGRESS_BARS = False
```

It is also possible to manually load a configuration file:

```
>>> pyphi.config.load_file('pyphi_config.yml')
```

Or load a dictionary of configuration values:

```
>>> pyphi.config.load_dict({'PRECISION': 1})
```

### Approximations and theoretical options¶

These settings control the algorithms PyPhi uses.

- `ASSUME_CUTS_CANNOT_CREATE_NEW_CONCEPTS`
- `CUT_ONE_APPROXIMATION`
- `MEASURE`
- `PARTITION_TYPE`
- `PICK_SMALLEST_PURVIEW`
- `USE_SMALL_PHI_DIFFERENCE_FOR_CES_DISTANCE`
- `SYSTEM_CUTS`
- `SINGLE_MICRO_NODES_WITH_SELFLOOPS_HAVE_PHI`
- `VALIDATE_SUBSYSTEM_STATES`
- `VALIDATE_CONDITIONAL_INDEPENDENCE`

### Parallelization and system resources¶

These settings control how much processing power and memory is available for
PyPhi to use. The default values may not be appropriate for your use-case or
machine, so **please check these settings before running anything**. Otherwise,
there is a risk that simulations might crash (potentially after running for a
long time!), resulting in data loss.

- `PARALLEL_CONCEPT_EVALUATION`
- `PARALLEL_CUT_EVALUATION`
- `PARALLEL_COMPLEX_EVALUATION`
- `NUMBER_OF_CORES`
- `MAXIMUM_CACHE_MEMORY_PERCENTAGE`

  Important

  Only one of `PARALLEL_CONCEPT_EVALUATION`, `PARALLEL_CUT_EVALUATION`,
  and `PARALLEL_COMPLEX_EVALUATION` can be set to `True` at a time.

  **For most networks,** `PARALLEL_CUT_EVALUATION` **is the most
  efficient.** This is because the algorithm is exponential time in the
  number of nodes, so the most of the time is spent on the largest subsystem.

  You should only parallelize concept evaluation if you are just computing a
  `CauseEffectStructure`.

### Memoization and caching¶

PyPhi provides a number of ways to cache intermediate results.

- `CACHE_SIAS`
- `CACHE_REPERTOIRES`
- `CACHE_POTENTIAL_PURVIEWS`
- `CLEAR_SUBSYSTEM_CACHES_AFTER_COMPUTING_SIA`
- `CACHING_BACKEND`
- `FS_CACHE_VERBOSITY`
- `FS_CACHE_DIRECTORY`
- `MONGODB_CONFIG`
- `REDIS_CACHE`
- `REDIS_CONFIG`

### Logging¶

These settings control how PyPhi handles log messages. Logs can be written to
standard output, a file, both, or none. If these simple default controls are
not flexible enough for you, you can override the entire logging configuration.
See the documentation on Python’s logger for more information.

- `LOG_STDOUT_LEVEL`
- `LOG_FILE_LEVEL`
- `LOG_FILE`
- `PROGRESS_BARS`
- `REPR_VERBOSITY`
- `PRINT_FRACTIONS`

### Numerical precision¶

- `PRECISION`

### The `config` API¶

*class* `pyphi.conf.``Option`(*default*, *values=None*, *on\_change=None*, *doc=None*)¶
:   A descriptor implementing PyPhi configuration options.

    |  |  |
    | --- | --- |
    | Parameters: | **default** – The default value of this `Option`. |
    | Keyword Arguments: | |
    |  | - **values** (*list*) – Allowed values for this option. A `ValueError` will   be raised if `values` is not `None` and the option is set to   be a value not in the list. - **on\_change** (*function*) – Optional callback that is called when the value   of the option is changed. The `Config` instance is passed as   the only argument to the callback. - **doc** (*str*) – Optional docstring for the option. |

*class* `pyphi.conf.``ConfigMeta`(*cls\_name*, *bases*, *namespace*)¶
:   Metaclass for `Config`.

    Responsible for setting the name of each `Option` when a subclass of
    `Config` is created; because `Option` objects are defined on the class,
    not the instance, their name should only be set once.

    Python 3.6 handles this exact need with the special descriptor method
    `__set_name__` (see PEP 487). We should use that once we drop support
    for 3.4 & 3.5.

*class* `pyphi.conf.``Config`¶
:   Base configuration object.

    See `PyphiConfig` for usage.

    *classmethod* `options`()¶
    :   Return a dictionary the `Option` objects for this config

    `defaults`()¶
    :   Return the default values of this configuration.

    `load_dict`(*dct*)¶
    :   Load a dictionary of configuration values.

    `load_file`(*filename*)¶
    :   Load config from a YAML file.

    `snapshot`()¶
    :   Return a snapshot of the current values of this configuration.

    `override`(*\*\*new\_values*)¶
    :   Decorator and context manager to override configuration values.

        The initial configuration values are reset after the decorated function
        returns or the context manager completes it block, even if the function
        or block raises an exception. This is intended to be used by tests
        which require specific configuration values.

        Example

        ```
        >>> from pyphi import config
        >>> @config.override(PRECISION=20000)
        ... def test_something():
        ...     assert config.PRECISION == 20000
        ...
        >>> test_something()
        >>> with config.override(PRECISION=100):
        ...     assert config.PRECISION == 100
        ...
        ```

`pyphi.conf.``configure_logging`(*conf*)¶
:   Reconfigure PyPhi logging based on the current configuration.

*class* `pyphi.conf.``PyphiConfig`¶
:   `pyphi.config` is an instance of this class.

    `ASSUME_CUTS_CANNOT_CREATE_NEW_CONCEPTS`¶
    :   `default=False`

        In certain cases, making a cut can actually cause a previously reducible
        concept to become a proper, irreducible concept. Assuming this can never
        happen can increase performance significantly, however the obtained results
        are not strictly accurate.

    `CUT_ONE_APPROXIMATION`¶
    :   `default=False`

        When determining the MIP for \(\Phi\), this restricts the set of system
        cuts that are considered to only those that cut the inputs or outputs of a
        single node. This restricted set of cuts scales linearly with the size of
        the system; the full set of all possible bipartitions scales
        exponentially. This approximation is more likely to give theoretically
        accurate results with modular, sparsely-connected, or homogeneous
        networks.

    `MEASURE`¶
    :   `default='EMD'`

        The measure to use when computing distances between repertoires and
        concepts. A full list of currently installed measures is available by
        calling `print(pyphi.distance.measures.all())`. Note that some measures
        cannot be used for calculating \(\Phi\) because they are asymmetric.

        Custom measures can be added using the `pyphi.distance.measures.register`
        decorator. For example:

        ```
        from pyphi.distance import measures

        @measures.register('ALWAYS_ZERO')
        def always_zero(a, b):
            return 0
        ```

        This measures can then be used by setting
        `config.MEASURE = 'ALWAYS_ZERO'`.

        If the measure is asymmetric you should register it using the
        `asymmetric` keyword argument. See `distance` for examples.

    `PARALLEL_CONCEPT_EVALUATION`¶
    :   `default=False`

        Controls whether concepts are evaluated in parallel when computing
        cause-effect structures.

    `PARALLEL_CUT_EVALUATION`¶
    :   `default=True`

        Controls whether system cuts are evaluated in parallel, which is faster but
        requires more memory. If cuts are evaluated sequentially, only two
        `SystemIrreducibilityAnalysis` instances need to be in memory at once.

    `PARALLEL_COMPLEX_EVALUATION`¶
    :   `default=False`

        Controls whether systems are evaluated in parallel when computing
        complexes.

    `NUMBER_OF_CORES`¶
    :   `default=-1`

        Controls the number of CPU cores used to evaluate unidirectional cuts.
        Negative numbers count backwards from the total number of available cores,
        with `-1` meaning ‘use all available cores.’

    `MAXIMUM_CACHE_MEMORY_PERCENTAGE`¶
    :   `default=50`

        PyPhi employs several in-memory caches to speed up computation. However,
        these can quickly use a lot of memory for large networks or large numbers
        of them; to avoid thrashing, this setting limits the percentage of a
        system’s RAM that the caches can collectively use.

    `CACHE_SIAS`¶
    :   `default=False`

        PyPhi is equipped with a transparent caching system for
        `SystemIrreducibilityAnalysis` objects which stores them as they are
        computed to avoid having to recompute them later. This makes it easy to
        play around interactively with the program, or to accumulate results with
        minimal effort. For larger projects, however, it is recommended that you
        manage the results explicitly, rather than relying on the cache. For this
        reason it is disabled by default.

    `CACHE_REPERTOIRES`¶
    :   `default=True`

        PyPhi caches cause and effect repertoires. This greatly improves speed, but
        can consume a significant amount of memory. If you are experiencing memory
        issues, try disabling this.

    `CACHE_POTENTIAL_PURVIEWS`¶
    :   `default=True`

        Controls whether the potential purviews of mechanisms of a network are
        cached. Caching speeds up computations by not recomputing expensive
        reducibility checks, but uses additional memory.

    `CLEAR_SUBSYSTEM_CACHES_AFTER_COMPUTING_SIA`¶
    :   `default=False`

        Controls whether a `Subsystem`’s repertoire and MICE caches are cleared
        with `clear_caches()` after computing the
        `SystemIrreducibilityAnalysis`. If you don’t need to do any more
        computations after running `sia()`, then enabling this may help
        conserve memory.

    `CACHING_BACKEND`¶
    :   `default='fs'`

        Controls whether precomputed results are stored and read from a local
        filesystem-based cache in the current directory or from a database. Set
        this to `'fs'` for the filesystem, `'db'` for the database.

    `FS_CACHE_VERBOSITY`¶
    :   `default=0`

        Controls how much caching information is printed if the filesystem cache is
        used. Takes a value between `0` and `11`.

    `FS_CACHE_DIRECTORY`¶
    :   `default='__pyphi_cache__'`

        If the filesystem is used for caching, the cache will be stored in this
        directory. This directory can be copied and moved around if you want to
        reuse results *e.g.* on a another computer, but it must be in the same
        directory from which Python is being run.

    `MONGODB_CONFIG`¶
    :   `default={'port' -- 27017, 'host' -- 'localhost', 'database_name': 'pyphi', 'collection_name': 'cache'}`

        Set the configuration for the MongoDB database backend (only has an
        effect if `CACHING_BACKEND` is `'db'`).

    `REDIS_CACHE`¶
    :   `default=False`

        Specifies whether to use Redis to cache `MaximallyIrreducibleCauseOrEffect`.

    `REDIS_CONFIG`¶
    :   `default={'port' -- 6379, 'host' -- 'localhost', 'db': 0, 'test_db': 1}`

        Configure the Redis database backend. These are the defaults in the
        provided `redis.conf` file.

    `LOG_FILE`¶
    :   `default='pyphi.log'`, `on_change=configure_logging`

        Controls the name of the log file.

    `LOG_FILE_LEVEL`¶
    :   `default='INFO'`, `on_change=configure_logging`

        Controls the level of log messages written to the log
        file. This setting has the same possible values as
        `LOG_STDOUT_LEVEL`.

    `LOG_STDOUT_LEVEL`¶
    :   `default='WARNING'`, `on_change=configure_logging`

        Controls the level of log messages written to standard
        output. Can be one of `'DEBUG'`, `'INFO'`, `'WARNING'`, `'ERROR'`,
        `'CRITICAL'`, or `None`. `'DEBUG'` is the least restrictive level and
        will show the most log messages. `'CRITICAL'` is the most restrictive
        level and will only display information about fatal errors. If set to
        `None`, logging to standard output will be disabled entirely.

    `PROGRESS_BARS`¶
    :   `default=True`

        Controls whether to show progress bars on the console.

        > Tip
        >
        > If you are iterating over many systems rather than doing one
        > long-running calculation, consider disabling this for speed.

    `PRECISION`¶
    :   `default=6`

        If `MEASURE` is `EMD`, then the Earth Mover’s Distance is calculated
        with an external C++ library that a numerical optimizer to find a good
        approximation. Consequently, systems with analytically zero \(\Phi\) will
        sometimes be numerically found to have a small but non-zero amount. This
        setting controls the number of decimal places to which PyPhi will consider
        EMD calculations accurate. Values of \(\Phi\) lower than `10e-PRECISION`
        will be considered insignificant and treated as zero. The default value is
        about as accurate as the EMD computations get.

    `VALIDATE_SUBSYSTEM_STATES`¶
    :   `default=True`

        Controls whether PyPhi checks if the subsystems’s state is possible
        (reachable with nonzero probability from some previous state), given the
        subsystem’s TPM (**which is conditioned on background conditions**). If
        this is turned off, then **calculated** \(\Phi\) **values may not be
        valid**, since they may be associated with a subsystem that could never be
        in the given state.

    `VALIDATE_CONDITIONAL_INDEPENDENCE`¶
    :   `default=True`

        Controls whether PyPhi checks if a system’s TPM is conditionally
        independent.

    `SINGLE_MICRO_NODES_WITH_SELFLOOPS_HAVE_PHI`¶
    :   `default=False`

        If set to `True`, the \(\Phi\) value of single micro-node subsystems is
        the difference between their unpartitioned `CauseEffectStructure` (a single
        concept) and the null concept. If set to False, their \(\Phi\) is defined
        to be zero. Single macro-node subsystems may always be cut, regardless of
        circumstances.

    `REPR_VERBOSITY`¶
    :   `default=2`, `values=[0, 1, 2]`

        Controls the verbosity of `__repr__` methods on PyPhi objects. Can be set
        to `0`, `1`, or `2`. If set to `1`, calling `repr` on PyPhi
        objects will return pretty-formatted and legible strings, excluding
        repertoires. If set to `2`, `repr` calls also include repertoires.

        Although this breaks the convention that `__repr__` methods should return
        a representation which can reconstruct the object, readable representations
        are convenient since the Python REPL calls `repr` to represent all
        objects in the shell and PyPhi is often used interactively with the
        REPL. If set to `0`, `repr` returns more traditional object
        representations.

    `PRINT_FRACTIONS`¶
    :   `default=True`

        Controls whether numbers in a `repr` are printed as fractions. Numbers
        are still printed as decimals if the fraction’s denominator would be
        large. This only has an effect if `REPR_VERBOSITY > 0`.

    `PARTITION_TYPE`¶
    :   `default='BI'`

        Controls the type of partition used for \(\varphi\) computations.

        If set to `'BI'`, partitions will have two parts.

        If set to `'TRI'`, partitions will have three parts. In addition,
        computations will only consider partitions that strictly partition the
        mechanism the mechanism. That is, for the mechanism `(A, B)` and purview
        `(B, C, D)` the partition:

        ```
        A,B    ∅
        ─── ✕ ───
         B    C,D
        ```

        is not considered, but:

        ```
         A     B
        ─── ✕ ───
         B    C,D
        ```

        is. The following is also valid:

        ```
        A,B     ∅
        ─── ✕ ─────
         ∅    B,C,D
        ```

        In addition, this setting introduces “wedge” tripartitions of the form:

        ```
         A     B     ∅
        ─── ✕ ─── ✕ ───
         B     C     D
        ```

        where the mechanism in the third part is always empty.

        In addition, in the case of a \(\varphi\)-tie when computing a `MaximallyIrreducibleCause` or
        `MaximallyIrreducibleEffect`, The `'TRIPARTITION'` setting choses the MIP with smallest purview
        instead of the largest (which is the default).

        Finally, if set to `'ALL'`, all possible partitions will be tested.

    `PICK_SMALLEST_PURVIEW`¶
    :   `default=False`

        When computing a `MaximallyIrreducibleCause` or `MaximallyIrreducibleEffect`, it is possible for several MIPs to have
        the same \(\varphi\) value. If this setting is set to `True` the MIP with
        the smallest purview is chosen; otherwise, the one with largest purview is
        chosen.

    `USE_SMALL_PHI_DIFFERENCE_FOR_CES_DISTANCE`¶
    :   `default=False`

        If set to `True`, the distance between cause-effect structures (when
        computing a `SystemIrreducibilityAnalysis`) is calculated using the
        difference between the sum of \(\varphi\) in the cause-effect structures
        instead of the extended EMD.

    `SYSTEM_CUTS`¶
    :   `default='3.0_STYLE'`, `values=['3.0_STYLE', 'CONCEPT_STYLE']`

        If set to `'3.0_STYLE'`, then traditional IIT 3.0 cuts will be used when
        computing \(\Phi\). If set to `'CONCEPT_STYLE'`, then experimental
        concept-style system cuts will be used instead.

    `log`()¶
    :   Log current settings.

### `actual`¶

Methods for computing actual causation of subsystems and mechanisms.

`pyphi.actual.``log2`(*x*)¶
:   Rounded version of `log2`.

*class* `pyphi.actual.``Transition`(*network*, *before\_state*, *after\_state*, *cause\_indices*, *effect\_indices*, *cut=None*, *noise\_background=False*)¶
:   A state transition between two sets of nodes in a network.

    A `Transition` is implemented with two `Subsystem` objects: one
    representing the system at time \(t-1\) used to compute effect coefficients,
    and another representing the system at time \(t\) which is used to compute
    cause coefficients. These subsystems are accessed with the
    `effect_system` and `cause_system` attributes, and are mapped to the
    causal directions via the `system` attribute.

    |  |  |
    | --- | --- |
    | Parameters: | - **network** (*Network*) – The network the subsystem belongs to. - **before\_state** (*tuple**[**int**]*) – The state of the network at   time \(t-1\). - **after\_state** (*tuple**[**int**]*) – The state of the network at   time \(t\). - **cause\_indices** (*tuple**[**int**] or* *tuple**[**str**]*) – Indices of nodes in the cause   system. (TODO: clarify) - **effect\_indices** (*tuple**[**int**] or* *tuple**[**str**]*) – Indices of nodes in the   effect system. (TODO: clarify) |
    | Keyword Arguments: | |
    |  | **noise\_background** (*bool*) – If `True`, background conditions are noised instead of frozen. |

    `node_indices`¶
    :   *tuple[int]* – The indices of the nodes in the system.

    `network`¶
    :   *Network* – The network the system belongs to.

    `before_state`¶
    :   *tuple[int]* – The state of the network at time \(t-1\).

    `after_state`¶
    :   *tuple[int]* – The state of the network at time \(t\).

    `effect_system`¶
    :   *Subsystem* – The system in `before_state` used to
        compute effect repertoires and coefficients.

    `cause_system`¶
    :   *Subsystem* – The system in `after_state` used to compute
        cause repertoires and coefficients.

    `cause_system`
    :   *Subsystem*

    `system`¶
    :   *dict* – A dictionary mapping causal directions to the system
        used to compute repertoires in that direction.

    `cut`¶
    :   *ActualCut* – The cut that has been applied to this transition.

    Note

    During initialization, both the cause and effect systems are
    conditioned on `before_state` as the background state. After
    conditioning the `effect_system` is then properly reset to
    `after_state`.

    `node_labels`¶

    `to_json`()¶
    :   Return a JSON-serializable representation.

    `apply_cut`(*cut*)¶
    :   Return a cut version of this transition.

    `cause_repertoire`(*mechanism*, *purview*)¶
    :   Return the cause repertoire.

    `effect_repertoire`(*mechanism*, *purview*)¶
    :   Return the effect repertoire.

    `unconstrained_cause_repertoire`(*purview*)¶
    :   Return the unconstrained cause repertoire of the occurence.

    `unconstrained_effect_repertoire`(*purview*)¶
    :   Return the unconstrained effect repertoire of the occurence.

    `repertoire`(*direction*, *mechanism*, *purview*)¶
    :   Return the cause or effect repertoire function based on a direction.

        |  |  |
        | --- | --- |
        | Parameters: | **direction** (*str*) – The temporal direction, specifiying the cause or effect repertoire. |

    `state_probability`(*direction*, *repertoire*, *purview*)¶
    :   Compute the probability of the purview in its current state given
        the repertoire.

        Collapses the dimensions of the repertoire that correspond to the
        purview nodes onto their state. All other dimension are already
        singular and thus receive 0 as the conditioning index.

        |  |  |
        | --- | --- |
        | Returns: | A single probabilty. |
        | Return type: | float |

    `probability`(*direction*, *mechanism*, *purview*)¶
    :   Probability that the purview is in it’s current state given the
        state of the mechanism.

    `unconstrained_probability`(*direction*, *purview*)¶
    :   Unconstrained probability of the purview.

    `purview_state`(*direction*)¶
    :   The state of the purview when we are computing coefficients in
        `direction`.

        For example, if we are computing the cause coefficient of a mechanism
        in `after_state`, the direction is``CAUSE`` and the `purview_state`
        is `before_state`.

    `mechanism_state`(*direction*)¶
    :   The state of the mechanism when computing coefficients in
        `direction`.

    `mechanism_indices`(*direction*)¶
    :   The indices of nodes in the mechanism system.

    `purview_indices`(*direction*)¶
    :   The indices of nodes in the purview system.

    `cause_ratio`(*mechanism*, *purview*)¶
    :   The cause ratio of the `purview` given `mechanism`.

    `effect_ratio`(*mechanism*, *purview*)¶
    :   The effect ratio of the `purview` given `mechanism`.

    `partitioned_repertoire`(*direction*, *partition*)¶
    :   Compute the repertoire over the partition in the given direction.

    `partitioned_probability`(*direction*, *partition*)¶
    :   Compute the probability of the mechanism over the purview in
        the partition.

    `find_mip`(*direction*, *mechanism*, *purview*, *allow\_neg=False*)¶
    :   Find the ratio minimum information partition for a mechanism
        over a purview.

        |  |  |
        | --- | --- |
        | Parameters: | - **direction** (*str*) – `CAUSE` or `EFFECT` - **mechanism** (*tuple**[**int**]*) – A mechanism. - **purview** (*tuple**[**int**]*) – A purview. |
        | Keyword Arguments: | |
        |  | **allow\_neg** (*boolean*) – If true, `alpha` is allowed to be negative. Otherwise, negative values of `alpha` will be treated as if they were 0. |
        | Returns: | The irreducibility analysis for the mechanism. |
        | Return type: | AcRepertoireIrreducibilityAnalysis |

    `potential_purviews`(*direction*, *mechanism*, *purviews=False*)¶
    :   Return all purviews that could belong to the `MaximallyIrreducibleCause`/`MaximallyIrreducibleEffect`.

        Filters out trivially-reducible purviews.

        |  |  |
        | --- | --- |
        | Parameters: | - **direction** (*str*) – Either `CAUSE` or `EFFECT`. - **mechanism** (*tuple**[**int**]*) – The mechanism of interest. |
        | Keyword Arguments: | |
        |  | **purviews** (*tuple**[**int**]*) – Optional subset of purviews of interest. |

    `find_causal_link`(*direction*, *mechanism*, *purviews=False*, *allow\_neg=False*)¶
    :   Return the maximally irreducible cause or effect ratio for a
        mechanism.

        |  |  |
        | --- | --- |
        | Parameters: | - **direction** (*str*) – The temporal direction, specifying cause or   effect. - **mechanism** (*tuple**[**int**]*) – The mechanism to be tested for   irreducibility. |
        | Keyword Arguments: | |
        |  | **purviews** (*tuple**[**int**]*) – Optionally restrict the possible purviews to a subset of the subsystem. This may be useful for \_e.g.\_ finding only concepts that are “about” a certain subset of nodes. |
        | Returns: | The maximally-irreducible actual cause or effect. |
        | Return type: | CausalLink |

    `find_actual_cause`(*mechanism*, *purviews=False*)¶
    :   Return the actual cause of a mechanism.

    `find_actual_effect`(*mechanism*, *purviews=False*)¶
    :   Return the actual effect of a mechanism.

    `find_mice`(*\*args*, *\*\*kwargs*)¶
    :   Backwards-compatible alias for `find_causal_link()`.

`pyphi.actual.``directed_account`(*transition*, *direction*, *mechanisms=False*, *purviews=False*, *allow\_neg=False*)¶
:   Return the set of all `CausalLink` of the specified direction.

`pyphi.actual.``account`(*transition*, *direction=<Direction.BIDIRECTIONAL: 2>*)¶
:   Return the set of all causal links for a `Transition`.

    |  |  |
    | --- | --- |
    | Parameters: | **transition** (*Transition*) – The transition of interest. |
    | Keyword Arguments: | |
    |  | **direction** (*Direction*) – By default the account contains actual causes and actual effects. |

`pyphi.actual.``account_distance`(*A1*, *A2*)¶
:   Return the distance between two accounts. Here that is just the
    difference in sum(alpha)

    |  |  |
    | --- | --- |
    | Parameters: | - **A1** (*Account*) – The first account. - **A2** (*Account*) – The second account |
    | Returns: | The distance between the two accounts. |
    | Return type: | float |

`pyphi.actual.``sia`(*transition*, *direction=<Direction.BIDIRECTIONAL: 2>*)¶
:   Return the minimal information partition of a transition in a specific
    direction.

    |  |  |
    | --- | --- |
    | Parameters: | **transition** (*Transition*) – The candidate system. |
    | Returns: | A nested structure containing all the data from the intermediate calculations. The top level contains the basic irreducibility information for the given subsystem. |
    | Return type: | AcSystemIrreducibilityAnalysis |

*class* `pyphi.actual.``ComputeACSystemIrreducibility`(*iterable*, *\*context*)¶
:   Computation engine for AC SIAs.

    `description` *= 'Evaluating AC cuts'*¶

    `empty_result`(*transition*, *direction*, *unpartitioned\_account*)¶
    :   Return the default result with which to begin the computation.

    *static* `compute`(*cut*, *transition*, *direction*, *unpartitioned\_account*)¶
    :   Map over a single object from `self.iterable`.

    `process_result`(*new\_sia*, *min\_sia*)¶
    :   Reduce handler.

        Every time a new result is generated by `compute`, this method is
        called with the result and the previous (accumulated) result. This
        method compares or collates these two values, returning the new result.

        Setting `self.done` to `True` in this method will abort the
        remainder of the computation, returning this final result.

`pyphi.actual.``transitions`(*network*, *before\_state*, *after\_state*)¶
:   Return a generator of all **possible** transitions of a network.

`pyphi.actual.``nexus`(*network*, *before\_state*, *after\_state*, *direction=<Direction.BIDIRECTIONAL: 2>*)¶
:   Return a tuple of all irreducible nexus of the network.

`pyphi.actual.``causal_nexus`(*network*, *before\_state*, *after\_state*, *direction=<Direction.BIDIRECTIONAL: 2>*)¶
:   Return the causal nexus of the network.

`pyphi.actual.``nice_true_ces`(*tc*)¶
:   Format a true `CauseEffectStructure`.

`pyphi.actual.``events`(*network*, *previous\_state*, *current\_state*, *next\_state*, *nodes*, *mechanisms=False*)¶
:   Find all events (mechanisms with actual causes and actual effects).

`pyphi.actual.``true_ces`(*subsystem*, *previous\_state*, *next\_state*)¶
:   Set of all sets of elements that have true causes and true effects.

    Note

    Since the true `CauseEffectStructure` is always about the full system,
    the background conditions don’t matter and the subsystem should be
    conditioned on the current state.

`pyphi.actual.``true_events`(*network*, *previous\_state*, *current\_state*, *next\_state*, *indices=None*, *major\_complex=None*)¶
:   Return all mechanisms that have true causes and true effects within the
    complex.

    |  |  |
    | --- | --- |
    | Parameters: | - **network** (*Network*) – The network to analyze. - **previous\_state** (*tuple**[**int**]*) – The state of the network at `t - 1`. - **current\_state** (*tuple**[**int**]*) – The state of the network at `t`. - **next\_state** (*tuple**[**int**]*) – The state of the network at `t + 1`. |
    | Keyword Arguments: | |
    |  | - **indices** (*tuple**[**int**]*) – The indices of the major complex. - **major\_complex** (*AcSystemIrreducibilityAnalysis*) – The major complex. If   `major_complex` is given then `indices` is ignored. |
    | Returns: | List of true events in the major complex. |
    | Return type: | tuple[Event] |

`pyphi.actual.``extrinsic_events`(*network*, *previous\_state*, *current\_state*, *next\_state*, *indices=None*, *major\_complex=None*)¶
:   Set of all mechanisms that are in the major complex but which have true
    causes and effects within the entire network.

    |  |  |
    | --- | --- |
    | Parameters: | - **network** (*Network*) – The network to analyze. - **previous\_state** (*tuple**[**int**]*) – The state of the network at `t - 1`. - **current\_state** (*tuple**[**int**]*) – The state of the network at `t`. - **next\_state** (*tuple**[**int**]*) – The state of the network at `t + 1`. |
    | Keyword Arguments: | |
    |  | - **indices** (*tuple**[**int**]*) – The indices of the major complex. - **major\_complex** (*AcSystemIrreducibilityAnalysis*) – The major complex. If   `major_complex` is given then `indices` is ignored. |
    | Returns: | List of extrinsic events in the major complex. |
    | Return type: | tuple(actions) |

### `cache`¶

Memoization and caching utilities.

`pyphi.cache.``memory_full`()¶
:   Check if the memory is too full for further caching.

`pyphi.cache.``cache`(*cache={}*, *maxmem=50*, *typed=False*)¶
:   Memory-limited cache decorator.

    `maxmem` is a float between 0 and 100, inclusive, specifying the maximum
    percentage of physical memory that the cache can use.

    If `typed` is `True`, arguments of different types will be cached
    separately. For example, f(3.0) and f(3) will be treated as distinct calls
    with distinct results.

    Arguments to the cached function must be hashable.

    View the cache statistics named tuple (hits, misses, currsize)
    with f.cache\_info(). Clear the cache and statistics with f.cache\_clear().
    Access the underlying function with f.\_\_wrapped\_\_.

*class* `pyphi.cache.``DictCache`¶
:   A generic dictionary-based cache.

    Intended to be used as an object-level cache of method results.

    `clear`()¶

    `size`()¶
    :   Number of items in cache

    `info`()¶
    :   Return info about cache hits, misses, and size

    `get`(*key*)¶
    :   Get a value out of the cache.

        Returns None if the key is not in the cache. Updates cache
        statistics.

    `set`(*key*, *value*)¶
    :   Set a value in the cache

    `key`(*\*args*, *\_prefix=None*, *\*\*kwargs*)¶
    :   Get the cache key for the given function args.

        Kwargs:
        :   prefix: A constant to prefix to the key.

`pyphi.cache.``redis_init`(*db*)¶

`pyphi.cache.``redis_available`()¶
:   Check if the Redis server is connected.

*class* `pyphi.cache.``RedisCache`¶
:   `clear`()¶
    :   Flush the cache.

    *static* `size`()¶
    :   Size of the Redis cache.

        Note

        This is the size of the entire Redis database.

    `info`()¶
    :   Return cache information.

        Note

        This is not the cache info for the entire Redis key space.

    `get`(*key*)¶
    :   Get a value from the cache.

        Returns None if the key is not in the cache.

    `set`(*key*, *value*)¶
    :   Set a value in the cache.

    `key`()¶
    :   Delegate to subclasses.

`pyphi.cache.``validate_parent_cache`(*parent\_cache*)¶

*class* `pyphi.cache.``RedisMICECache`(*subsystem*, *parent\_cache=None*)¶
:   A Redis-backed cache for `find_mice()`.

    See `MICECache` for more info.

    `get`(*key*)¶
    :   Get a value from the cache.

        If the `MaximallyIrreducibleCauseOrEffect` cannot be found in this cache, try and find it in the
        parent cache.

    `set`(*key*, *value*)¶
    :   Only need to set if the subsystem is uncut.

        Caches are only inherited from uncut subsystems.

    `key`(*direction*, *mechanism*, *purviews=False*, *\_prefix=None*)¶
    :   Cache key. This is the call signature of `find_mice()`.

*class* `pyphi.cache.``DictMICECache`(*subsystem*, *parent\_cache=None*)¶
:   A subsystem-local cache for `MaximallyIrreducibleCauseOrEffect` objects.

    See `MICECache` for more info.

    `set`(*key*, *mice*)¶
    :   Set a value in the cache.

        Only cache if:
        :   - The subsystem is uncut (caches are only inherited from
              uncut subsystems so there is no reason to cache on cut
              subsystems.)
            - \(\varphi\) > 0. Ideally we would cache all mice, but the size
              of the cache grows way too large, making parallel computations
              incredibly inefficient because the caches have to be passed
              between process. This will be changed once global caches are
              implemented.
            - Memory is not too full.

    `key`(*direction*, *mechanism*, *purviews=False*, *\_prefix=None*)¶
    :   Cache key. This is the call signature of `find_mice()`.

`pyphi.cache.``MICECache`(*subsystem*, *parent\_cache=None*)¶
:   Construct a `MaximallyIrreducibleCauseOrEffect` cache.

    Uses either a Redis-backed cache or a local dict cache on the object.

    |  |  |
    | --- | --- |
    | Parameters: | **subsystem** (*Subsystem*) – The subsystem that this is a cache for. |

    Kwargs:
    :   parent\_cache (MICECache): The cache generated by the uncut
        :   version of `subsystem`. Any cached `MaximallyIrreducibleCauseOrEffect` which are
            unaffected by the cut are reused in this cache. If None,
            the cache is initialized empty.

*class* `pyphi.cache.``PurviewCache`¶
:   A network-level cache for possible purviews.

    `set`(*key*, *value*)¶
    :   Only set if purview caching is enabled

`pyphi.cache.``method`(*cache\_name*, *key\_prefix=None*)¶
:   Caching decorator for object-level method caches.

    Cache key generation is delegated to the cache.

    |  |  |
    | --- | --- |
    | Parameters: | - **cache\_name** (*str*) – The name of the (already-instantiated) cache   on the decorated object which should be used to store results   of this method. - **\*key\_prefix** – A constant to use as part of the cache key in addition   to the method arguments. |

### `compute`¶

See `pyphi.compute.subsystem`, `pyphi.compute.network`, `pyphi.compute.distance`, and
`pyphi.compute.parallel` for documentation.

`pyphi.compute.``all_complexes`¶
:   Alias for `pyphi.compute.network.all_complexes()`.

`pyphi.compute.``ces`¶
:   Alias for `pyphi.compute.subsystem.ces()`.

`pyphi.compute.``ces_distance`¶
:   Alias for `pyphi.compute.distance.ces_distance()`.

`pyphi.compute.``complexes`¶
:   Alias for `pyphi.compute.network.complexes()`.

`pyphi.compute.``concept_distance`¶
:   Alias for
    `pyphi.compute.distance.concept_distance()`.

`pyphi.compute.``conceptual_info`¶
:   Alias for `pyphi.compute.subsystem.conceptual_info()`.

`pyphi.compute.``condensed`¶
:   Alias for `pyphi.compute.network.condensed()`.

`pyphi.compute.``evaluate_cut`¶
:   Alias for `pyphi.compute.subsystem.evaluate_cut()`.

`pyphi.compute.``major_complex`¶
:   Alias for `pyphi.compute.network.major_complex()`.

`pyphi.compute.``phi`¶
:   Alias for `pyphi.compute.subsystem.phi()`.

`pyphi.compute.``possible_complexes`¶
:   Alias for
    `pyphi.compute.network.possible_complexes()`.

`pyphi.compute.``sia`¶
:   Alias for `pyphi.compute.subsystem.sia()`.

`pyphi.compute.``subsystems`¶
:   Alias for `pyphi.compute.network.subsystems()`.

### `compute.distance`¶

Functions for computing distances between various PyPhi objects.

`pyphi.compute.distance.``concept_distance`(*c1*, *c2*)¶
:   Return the distance between two concepts in concept space.

    |  |  |
    | --- | --- |
    | Parameters: | - **c1** (*Concept*) – The first concept. - **c2** (*Concept*) – The second concept. |
    | Returns: | The distance between the two concepts in concept space. |
    | Return type: | float |

`pyphi.compute.distance.``ces_distance`(*C1*, *C2*)¶
:   Return the distance between two cause-effect structures.

    |  |  |
    | --- | --- |
    | Parameters: | - **C1** (*CauseEffectStructure*) – The first `CauseEffectStructure`. - **C2** (*CauseEffectStructure*) – The second `CauseEffectStructure`. |
    | Returns: | The distance between the two cause-effect structures in concept space. |
    | Return type: | float |

`pyphi.compute.distance.``small_phi_ces_distance`(*C1*, *C2*)¶
:   Return the difference in \(\varphi\) between `CauseEffectStructure`.

### `compute.network`¶

Functions for computing network-level properties.

`pyphi.compute.network.``subsystems`(*network*, *state*)¶
:   Return a generator of all **possible** subsystems of a network.

    Note

    Does not return subsystems that are in an impossible state (after
    conditioning the subsystem TPM on the state of the other nodes).

    |  |  |
    | --- | --- |
    | Parameters: | - **network** (*Network*) – The `Network` of interest. - **state** (*tuple**[**int**]*) – The state of the network (a binary tuple). |
    | Yields: | *Subsystem* – A `Subsystem` for each subset of nodes in the network, excluding subsystems that would be in an impossible state. |

`pyphi.compute.network.``possible_complexes`(*network*, *state*)¶
:   Return a generator of subsystems of a network that could be a complex.

    This is the just powerset of the nodes that have at least one input and
    output (nodes with no inputs or no outputs cannot be part of a main
    complex, because they do not have a causal link with the rest of the
    subsystem in the previous or next timestep, respectively).

    Note

    Does not return subsystems that are in an impossible state (after
    conditioning the subsystem TPM on the state of the other nodes).

    |  |  |
    | --- | --- |
    | Parameters: | - **network** (*Network*) – The `Network` of interest. - **state** (*tuple**[**int**]*) – The state of the network (a binary tuple). |
    | Yields: | *Subsystem* – The next subsystem that could be a complex. |

*class* `pyphi.compute.network.``FindAllComplexes`(*iterable*, *\*context*)¶
:   Computation engine for finding all complexes.

    `description` *= 'Finding complexes'*¶

    `empty_result`()¶
    :   Return the default result with which to begin the computation.

    *static* `compute`(*subsystem*)¶
    :   Map over a single object from `self.iterable`.

    `process_result`(*new\_sia*, *sias*)¶
    :   Reduce handler.

        Every time a new result is generated by `compute`, this method is
        called with the result and the previous (accumulated) result. This
        method compares or collates these two values, returning the new result.

        Setting `self.done` to `True` in this method will abort the
        remainder of the computation, returning this final result.

`pyphi.compute.network.``all_complexes`(*network*, *state*)¶
:   Return a generator for all complexes of the network.

    Note

    Includes reducible, zero-\(\Phi\) complexes (which are not, strictly
    speaking, complexes at all).

    |  |  |
    | --- | --- |
    | Parameters: | - **network** (*Network*) – The `Network` of interest. - **state** (*tuple**[**int**]*) – The state of the network (a binary tuple). |
    | Yields: | *SystemIrreducibilityAnalysis* – A `SystemIrreducibilityAnalysis` for each `Subsystem` of the `Network`. |

*class* `pyphi.compute.network.``FindIrreducibleComplexes`(*iterable*, *\*context*)¶
:   Computation engine for finding irreducible complexes of a network.

    `process_result`(*new\_sia*, *sias*)¶
    :   Reduce handler.

        Every time a new result is generated by `compute`, this method is
        called with the result and the previous (accumulated) result. This
        method compares or collates these two values, returning the new result.

        Setting `self.done` to `True` in this method will abort the
        remainder of the computation, returning this final result.

`pyphi.compute.network.``complexes`(*network*, *state*)¶
:   Return all irreducible complexes of the network.

    |  |  |
    | --- | --- |
    | Parameters: | - **network** (*Network*) – The `Network` of interest. - **state** (*tuple**[**int**]*) – The state of the network (a binary tuple). |
    | Yields: | *SystemIrreducibilityAnalysis* – A `SystemIrreducibilityAnalysis` for each `Subsystem` of the `Network`, excluding those with \(\Phi = 0\). |

`pyphi.compute.network.``major_complex`(*network*, *state*)¶
:   Return the major complex of the network.

    |  |  |
    | --- | --- |
    | Parameters: | - **network** (*Network*) – The `Network` of interest. - **state** (*tuple**[**int**]*) – The state of the network (a binary tuple). |
    | Returns: | The `SystemIrreducibilityAnalysis` for the `Subsystem` with maximal \(\Phi\). |
    | Return type: | SystemIrreducibilityAnalysis |

`pyphi.compute.network.``condensed`(*network*, *state*)¶
:   Return a list of maximal non-overlapping complexes.

    |  |  |
    | --- | --- |
    | Parameters: | - **network** (*Network*) – The `Network` of interest. - **state** (*tuple**[**int**]*) – The state of the network (a binary tuple). |
    | Returns: | A list of `SystemIrreducibilityAnalysis` for non-overlapping complexes with maximal \(\Phi\) values. |
    | Return type: | list[SystemIrreducibilityAnalysis] |

### `compute.parallel`¶

Utilities for parallel computation.

`pyphi.compute.parallel.``get_num_processes`()¶
:   Return the number of processes to use in parallel.

*class* `pyphi.compute.parallel.``ExceptionWrapper`(*exception*)¶
:   A picklable wrapper suitable for passing exception tracebacks through
    instances of `multiprocessing.Queue`.

    |  |  |
    | --- | --- |
    | Parameters: | **exception** (*Exception*) – The exception to wrap. |

    `reraise`()¶
    :   Re-raise the exception.

*class* `pyphi.compute.parallel.``MapReduce`(*iterable*, *\*context*)¶
:   An engine for doing heavy computations over an iterable.

    This is similar to `multiprocessing.Pool`, but allows computations to
    shortcircuit, and supports both parallel and sequential computations.

    |  |  |
    | --- | --- |
    | Parameters: | - **iterable** (*Iterable*) – A collection of objects to perform a computation   over. - **\*context** – Any additional data necessary to complete the computation. |

    Any subclass of `MapReduce` must implement three methods:

    ```
    - ``empty_result``,
    - ``compute``, (map), and
    - ``process_result`` (reduce).
    ```

    The engine includes a builtin `tqdm` progress bar; this can be disabled
    by setting `pyphi.config.PROGRESS_BARS` to `False`.

    Parallel operations start a daemon thread which handles log messages sent
    from worker processes.

    Subprocesses spawned by `MapReduce` cannot spawn more subprocesses; be
    aware of this when composing nested computations. This is not an issue in
    practice because it is typically most efficient to only parallelize the top
    level computation.

    `description` *= ''*¶

    `empty_result`(*\*context*)¶
    :   Return the default result with which to begin the computation.

    *static* `compute`(*obj*, *\*context*)¶
    :   Map over a single object from `self.iterable`.

    `process_result`(*new\_result*, *old\_result*)¶
    :   Reduce handler.

        Every time a new result is generated by `compute`, this method is
        called with the result and the previous (accumulated) result. This
        method compares or collates these two values, returning the new result.

        Setting `self.done` to `True` in this method will abort the
        remainder of the computation, returning this final result.

    `init_progress_bar`()¶
    :   Initialize and return a progress bar.

    *static* `worker`(*compute*, *task\_queue*, *result\_queue*, *log\_queue*, *complete*, *\*context*)¶
    :   A worker process, run by `multiprocessing.Process`.

    `start_parallel`()¶
    :   Initialize all queues and start the worker processes and the log
        thread.

    `initialize_tasks`()¶
    :   Load the input queue to capacity.

        Overfilling causes a deadlock when queue.put blocks when
        full, so further tasks are enqueued as results are returned.

    `maybe_put_task`()¶
    :   Enqueue the next task, if there are any waiting.

    `run_parallel`()¶
    :   Perform the computation in parallel, reading results from the output
        queue and passing them to `process_result`.

    `finish_parallel`()¶
    :   Orderly shutdown of workers.

    `run_sequential`()¶
    :   Perform the computation sequentially, only holding two computed
        objects in memory at a time.

    `run`(*parallel=True*)¶
    :   Perform the computation.

        | Keyword Arguments: | |
        | --- | --- |
        |  | **parallel** (*boolean*) – If True, run the computation in parallel. Otherwise, operate sequentially. |

*class* `pyphi.compute.parallel.``LogThread`(*q*)¶
:   Thread which handles log records sent from `MapReduce` processes.

    It listens to an instance of `multiprocessing.Queue`, rewriting log
    messages to the PyPhi log handler.

    `run`()¶
    :   Method representing the thread’s activity.

        You may override this method in a subclass. The standard run() method
        invokes the callable object passed to the object’s constructor as the
        target argument, if any, with sequential and keyword arguments taken
        from the args and kwargs arguments, respectively.

`pyphi.compute.parallel.``configure_worker_logging`(*queue*)¶
:   Configure a worker process to log all messages to `queue`.

### `compute.subsystem`¶

Functions for computing subsystem-level properties.

*class* `pyphi.compute.subsystem.``ComputeCauseEffectStructure`(*iterable*, *\*context*)¶
:   Engine for computing a `CauseEffectStructure`.

    `description` *= 'Computing concepts'*¶

    `subsystem`¶

    `empty_result`(*\*args*)¶
    :   Return the default result with which to begin the computation.

    *static* `compute`(*mechanism*, *subsystem*, *purviews*, *cause\_purviews*, *effect\_purviews*)¶
    :   Compute a `Concept` for a mechanism, in this `Subsystem` with the
        provided purviews.

    `process_result`(*new\_concept*, *concepts*)¶
    :   Save all concepts with non-zero \(\varphi\) to the
        `CauseEffectStructure`.

`pyphi.compute.subsystem.``ces`(*subsystem*, *mechanisms=False*, *purviews=False*, *cause\_purviews=False*, *effect\_purviews=False*, *parallel=False*)¶
:   Return the conceptual structure of this subsystem, optionally restricted
    to concepts with the mechanisms and purviews given in keyword arguments.

    If you don’t need the full `CauseEffectStructure`, restricting the possible
    mechanisms and purviews can make this function much faster.

    |  |  |
    | --- | --- |
    | Parameters: | **subsystem** (*Subsystem*) – The subsystem for which to determine the `CauseEffectStructure`. |
    | Keyword Arguments: | |
    |  | - **mechanisms** (*tuple**[**tuple**[**int**]**]*) – Restrict possible mechanisms to those   in this list. - **purviews** (*tuple**[**tuple**[**int**]**]*) – Same as in `concept()`. - **cause\_purviews** (*tuple**[**tuple**[**int**]**]*) – Same as in `concept()`. - **effect\_purviews** (*tuple**[**tuple**[**int**]**]*) – Same as in `concept()`. - **parallel** (*bool*) – Whether to compute concepts in parallel. If `True`,   overrides `config.PARALLEL_CONCEPT_EVALUATION`. |
    | Returns: | A tuple of every `Concept` in the cause-effect structure. |
    | Return type: | CauseEffectStructure |

`pyphi.compute.subsystem.``conceptual_info`(*subsystem*)¶
:   Return the conceptual information for a `Subsystem`.

    This is the distance from the subsystem’s `CauseEffectStructure` to the
    null concept.

`pyphi.compute.subsystem.``evaluate_cut`(*uncut\_subsystem*, *cut*, *unpartitioned\_ces*)¶
:   Compute the system irreducibility for a given cut.

    |  |  |
    | --- | --- |
    | Parameters: | - **uncut\_subsystem** (*Subsystem*) – The subsystem without the cut applied. - **cut** (*Cut*) – The cut to evaluate. - **unpartitioned\_ces** (*CauseEffectStructure*) – The cause-effect structure of   the uncut subsystem. |
    | Returns: | The `SystemIrreducibilityAnalysis` for that cut. |
    | Return type: | SystemIrreducibilityAnalysis |

*class* `pyphi.compute.subsystem.``ComputeSystemIrreducibility`(*iterable*, *\*context*)¶
:   Computation engine for system-level irreducibility.

    `description` *= 'Evaluating Φ cuts'*¶

    `empty_result`(*subsystem*, *unpartitioned\_ces*)¶
    :   Begin with a `SystemIrreducibilityAnalysis` with infinite \(\Phi\); all actual SIAs will
        have less.

    *static* `compute`(*cut*, *subsystem*, *unpartitioned\_ces*)¶
    :   Evaluate a cut.

    `process_result`(*new\_sia*, *min\_sia*)¶
    :   Check if the new SIA has smaller \(\Phi\) than the standing
        result.

`pyphi.compute.subsystem.``sia_bipartitions`(*nodes*, *node\_labels=None*)¶
:   Return all \(\Phi\) cuts for the given nodes.

    This value changes based on `config.CUT_ONE_APPROXIMATION`.

    |  |  |
    | --- | --- |
    | Parameters: | **nodes** (*tuple**[**int**]*) – The node indices to partition. |
    | Returns: | All unidirectional partitions. |
    | Return type: | list[Cut] |

`pyphi.compute.subsystem.``sia`(*cache\_key*, *subsystem*)¶
:   Return the minimal information partition of a subsystem.

    |  |  |
    | --- | --- |
    | Parameters: | **subsystem** (*Subsystem*) – The candidate set of nodes. |
    | Returns: | A nested structure containing all the data from the intermediate calculations. The top level contains the basic irreducibility information for the given subsystem. |
    | Return type: | SystemIrreducibilityAnalysis |

`pyphi.compute.subsystem.``phi`(*subsystem*)¶
:   Return the \(\Phi\) value of a subsystem.

*class* `pyphi.compute.subsystem.``ConceptStyleSystem`(*subsystem*, *direction*, *cut=None*)¶
:   A functional replacement for `Subsystem` implementing concept-style
    system cuts.

    `apply_cut`(*cut*)¶

    `__getattr__`(*name*)¶
    :   Pass attribute access through to the basic subsystem.

    `cause_system`¶

    `effect_system`¶

    `concept`(*mechanism*, *purviews=False*, *cause\_purviews=False*, *effect\_purviews=False*)¶
    :   Compute a concept, using the appropriate system for each side of the
        cut.

`pyphi.compute.subsystem.``concept_cuts`(*direction*, *node\_indices*, *node\_labels=None*)¶
:   Generator over all concept-syle cuts for these nodes.

`pyphi.compute.subsystem.``directional_sia`(*subsystem*, *direction*, *unpartitioned\_ces=None*)¶
:   Calculate a concept-style SystemIrreducibilityAnalysisCause or
    SystemIrreducibilityAnalysisEffect.

*class* `pyphi.compute.subsystem.``SystemIrreducibilityAnalysisConceptStyle`(*sia\_cause*, *sia\_effect*)¶
:   Represents a `SystemIrreducibilityAnalysis` computed using concept-style system cuts.

    `min_sia`¶

    `__getattr__`(*name*)¶
    :   Pass attribute access through to the minimal SIA.

    `unorderable_unless_eq` *= ['network']*¶

    `order_by`()¶
    :   Return a list of values to compare for ordering.

        The first value in the list has the greatest priority; if the first
        objects are equal the second object is compared, etc.

`pyphi.compute.subsystem.``sia_concept_style`(*subsystem*)¶
:   Compute a concept-style SystemIrreducibilityAnalysis

### `conf`¶

#### Loading a configuration¶

Various aspects of PyPhi’s behavior can be configured.

When PyPhi is imported, it checks for a YAML file named `pyphi_config.yml` in
the current directory and automatically loads it if it exists; otherwise the
default configuration is used.

The various settings are listed here with their defaults.

```
>>> import pyphi
>>> defaults = pyphi.config.defaults()
```

Print the `config` object to see the current settings:

```
>>> print(pyphi.config)  
{ 'ASSUME_CUTS_CANNOT_CREATE_NEW_CONCEPTS': False,
  'CACHE_SIAS': False,
  'CACHE_POTENTIAL_PURVIEWS': True,
  'CACHING_BACKEND': 'fs',
  ...
```

Setting can be changed on the fly by assigning them a new value:

```
>>> pyphi.config.PROGRESS_BARS = False
```

It is also possible to manually load a configuration file:

```
>>> pyphi.config.load_file('pyphi_config.yml')
```

Or load a dictionary of configuration values:

```
>>> pyphi.config.load_dict({'PRECISION': 1})
```

#### Approximations and theoretical options¶

These settings control the algorithms PyPhi uses.

- `ASSUME_CUTS_CANNOT_CREATE_NEW_CONCEPTS`
- `CUT_ONE_APPROXIMATION`
- `MEASURE`
- `PARTITION_TYPE`
- `PICK_SMALLEST_PURVIEW`
- `USE_SMALL_PHI_DIFFERENCE_FOR_CES_DISTANCE`
- `SYSTEM_CUTS`
- `SINGLE_MICRO_NODES_WITH_SELFLOOPS_HAVE_PHI`
- `VALIDATE_SUBSYSTEM_STATES`
- `VALIDATE_CONDITIONAL_INDEPENDENCE`

#### Parallelization and system resources¶

These settings control how much processing power and memory is available for
PyPhi to use. The default values may not be appropriate for your use-case or
machine, so **please check these settings before running anything**. Otherwise,
there is a risk that simulations might crash (potentially after running for a
long time!), resulting in data loss.

- `PARALLEL_CONCEPT_EVALUATION`
- `PARALLEL_CUT_EVALUATION`
- `PARALLEL_COMPLEX_EVALUATION`
- `NUMBER_OF_CORES`
- `MAXIMUM_CACHE_MEMORY_PERCENTAGE`

  Important

  Only one of `PARALLEL_CONCEPT_EVALUATION`, `PARALLEL_CUT_EVALUATION`,
  and `PARALLEL_COMPLEX_EVALUATION` can be set to `True` at a time.

  **For most networks,** `PARALLEL_CUT_EVALUATION` **is the most
  efficient.** This is because the algorithm is exponential time in the
  number of nodes, so the most of the time is spent on the largest subsystem.

  You should only parallelize concept evaluation if you are just computing a
  `CauseEffectStructure`.

#### Memoization and caching¶

PyPhi provides a number of ways to cache intermediate results.

- `CACHE_SIAS`
- `CACHE_REPERTOIRES`
- `CACHE_POTENTIAL_PURVIEWS`
- `CLEAR_SUBSYSTEM_CACHES_AFTER_COMPUTING_SIA`
- `CACHING_BACKEND`
- `FS_CACHE_VERBOSITY`
- `FS_CACHE_DIRECTORY`
- `MONGODB_CONFIG`
- `REDIS_CACHE`
- `REDIS_CONFIG`

#### Logging¶

These settings control how PyPhi handles log messages. Logs can be written to
standard output, a file, both, or none. If these simple default controls are
not flexible enough for you, you can override the entire logging configuration.
See the documentation on Python’s logger for more information.

- `LOG_STDOUT_LEVEL`
- `LOG_FILE_LEVEL`
- `LOG_FILE`
- `PROGRESS_BARS`
- `REPR_VERBOSITY`
- `PRINT_FRACTIONS`

#### Numerical precision¶

- `PRECISION`

#### The `config` API¶

*class* `pyphi.conf.``Option`(*default*, *values=None*, *on\_change=None*, *doc=None*)
:   A descriptor implementing PyPhi configuration options.

    |  |  |
    | --- | --- |
    | Parameters: | **default** – The default value of this `Option`. |
    | Keyword Arguments: | |
    |  | - **values** (*list*) – Allowed values for this option. A `ValueError` will   be raised if `values` is not `None` and the option is set to   be a value not in the list. - **on\_change** (*function*) – Optional callback that is called when the value   of the option is changed. The `Config` instance is passed as   the only argument to the callback. - **doc** (*str*) – Optional docstring for the option. |

*class* `pyphi.conf.``ConfigMeta`(*cls\_name*, *bases*, *namespace*)
:   Metaclass for `Config`.

    Responsible for setting the name of each `Option` when a subclass of
    `Config` is created; because `Option` objects are defined on the class,
    not the instance, their name should only be set once.

    Python 3.6 handles this exact need with the special descriptor method
    `__set_name__` (see PEP 487). We should use that once we drop support
    for 3.4 & 3.5.

*class* `pyphi.conf.``Config`
:   Base configuration object.

    See `PyphiConfig` for usage.

    *classmethod* `options`()
    :   Return a dictionary the `Option` objects for this config

    `defaults`()
    :   Return the default values of this configuration.

    `load_dict`(*dct*)
    :   Load a dictionary of configuration values.

    `load_file`(*filename*)
    :   Load config from a YAML file.

    `snapshot`()
    :   Return a snapshot of the current values of this configuration.

    `override`(*\*\*new\_values*)
    :   Decorator and context manager to override configuration values.

        The initial configuration values are reset after the decorated function
        returns or the context manager completes it block, even if the function
        or block raises an exception. This is intended to be used by tests
        which require specific configuration values.

        Example

        ```
        >>> from pyphi import config
        >>> @config.override(PRECISION=20000)
        ... def test_something():
        ...     assert config.PRECISION == 20000
        ...
        >>> test_something()
        >>> with config.override(PRECISION=100):
        ...     assert config.PRECISION == 100
        ...
        ```

`pyphi.conf.``configure_logging`(*conf*)
:   Reconfigure PyPhi logging based on the current configuration.

*class* `pyphi.conf.``PyphiConfig`
:   `pyphi.config` is an instance of this class.

    `ASSUME_CUTS_CANNOT_CREATE_NEW_CONCEPTS`
    :   `default=False`

        In certain cases, making a cut can actually cause a previously reducible
        concept to become a proper, irreducible concept. Assuming this can never
        happen can increase performance significantly, however the obtained results
        are not strictly accurate.

    `CUT_ONE_APPROXIMATION`
    :   `default=False`

        When determining the MIP for \(\Phi\), this restricts the set of system
        cuts that are considered to only those that cut the inputs or outputs of a
        single node. This restricted set of cuts scales linearly with the size of
        the system; the full set of all possible bipartitions scales
        exponentially. This approximation is more likely to give theoretically
        accurate results with modular, sparsely-connected, or homogeneous
        networks.

    `MEASURE`
    :   `default='EMD'`

        The measure to use when computing distances between repertoires and
        concepts. A full list of currently installed measures is available by
        calling `print(pyphi.distance.measures.all())`. Note that some measures
        cannot be used for calculating \(\Phi\) because they are asymmetric.

        Custom measures can be added using the `pyphi.distance.measures.register`
        decorator. For example:

        ```
        from pyphi.distance import measures

        @measures.register('ALWAYS_ZERO')
        def always_zero(a, b):
            return 0
        ```

        This measures can then be used by setting
        `config.MEASURE = 'ALWAYS_ZERO'`.

        If the measure is asymmetric you should register it using the
        `asymmetric` keyword argument. See `distance` for examples.

    `PARALLEL_CONCEPT_EVALUATION`
    :   `default=False`

        Controls whether concepts are evaluated in parallel when computing
        cause-effect structures.

    `PARALLEL_CUT_EVALUATION`
    :   `default=True`

        Controls whether system cuts are evaluated in parallel, which is faster but
        requires more memory. If cuts are evaluated sequentially, only two
        `SystemIrreducibilityAnalysis` instances need to be in memory at once.

    `PARALLEL_COMPLEX_EVALUATION`
    :   `default=False`

        Controls whether systems are evaluated in parallel when computing
        complexes.

    `NUMBER_OF_CORES`
    :   `default=-1`

        Controls the number of CPU cores used to evaluate unidirectional cuts.
        Negative numbers count backwards from the total number of available cores,
        with `-1` meaning ‘use all available cores.’

    `MAXIMUM_CACHE_MEMORY_PERCENTAGE`
    :   `default=50`

        PyPhi employs several in-memory caches to speed up computation. However,
        these can quickly use a lot of memory for large networks or large numbers
        of them; to avoid thrashing, this setting limits the percentage of a
        system’s RAM that the caches can collectively use.

    `CACHE_SIAS`
    :   `default=False`

        PyPhi is equipped with a transparent caching system for
        `SystemIrreducibilityAnalysis` objects which stores them as they are
        computed to avoid having to recompute them later. This makes it easy to
        play around interactively with the program, or to accumulate results with
        minimal effort. For larger projects, however, it is recommended that you
        manage the results explicitly, rather than relying on the cache. For this
        reason it is disabled by default.

    `CACHE_REPERTOIRES`
    :   `default=True`

        PyPhi caches cause and effect repertoires. This greatly improves speed, but
        can consume a significant amount of memory. If you are experiencing memory
        issues, try disabling this.

    `CACHE_POTENTIAL_PURVIEWS`
    :   `default=True`

        Controls whether the potential purviews of mechanisms of a network are
        cached. Caching speeds up computations by not recomputing expensive
        reducibility checks, but uses additional memory.

    `CLEAR_SUBSYSTEM_CACHES_AFTER_COMPUTING_SIA`
    :   `default=False`

        Controls whether a `Subsystem`’s repertoire and MICE caches are cleared
        with `clear_caches()` after computing the
        `SystemIrreducibilityAnalysis`. If you don’t need to do any more
        computations after running `sia()`, then enabling this may help
        conserve memory.

    `CACHING_BACKEND`
    :   `default='fs'`

        Controls whether precomputed results are stored and read from a local
        filesystem-based cache in the current directory or from a database. Set
        this to `'fs'` for the filesystem, `'db'` for the database.

    `FS_CACHE_VERBOSITY`
    :   `default=0`

        Controls how much caching information is printed if the filesystem cache is
        used. Takes a value between `0` and `11`.

    `FS_CACHE_DIRECTORY`
    :   `default='__pyphi_cache__'`

        If the filesystem is used for caching, the cache will be stored in this
        directory. This directory can be copied and moved around if you want to
        reuse results *e.g.* on a another computer, but it must be in the same
        directory from which Python is being run.

    `MONGODB_CONFIG`
    :   `default={'port' -- 27017, 'host' -- 'localhost', 'database_name': 'pyphi', 'collection_name': 'cache'}`

        Set the configuration for the MongoDB database backend (only has an
        effect if `CACHING_BACKEND` is `'db'`).

    `REDIS_CACHE`
    :   `default=False`

        Specifies whether to use Redis to cache `MaximallyIrreducibleCauseOrEffect`.

    `REDIS_CONFIG`
    :   `default={'port' -- 6379, 'host' -- 'localhost', 'db': 0, 'test_db': 1}`

        Configure the Redis database backend. These are the defaults in the
        provided `redis.conf` file.

    `LOG_FILE`
    :   `default='pyphi.log'`, `on_change=configure_logging`

        Controls the name of the log file.

    `LOG_FILE_LEVEL`
    :   `default='INFO'`, `on_change=configure_logging`

        Controls the level of log messages written to the log
        file. This setting has the same possible values as
        `LOG_STDOUT_LEVEL`.

    `LOG_STDOUT_LEVEL`
    :   `default='WARNING'`, `on_change=configure_logging`

        Controls the level of log messages written to standard
        output. Can be one of `'DEBUG'`, `'INFO'`, `'WARNING'`, `'ERROR'`,
        `'CRITICAL'`, or `None`. `'DEBUG'` is the least restrictive level and
        will show the most log messages. `'CRITICAL'` is the most restrictive
        level and will only display information about fatal errors. If set to
        `None`, logging to standard output will be disabled entirely.

    `PROGRESS_BARS`
    :   `default=True`

        Controls whether to show progress bars on the console.

        > Tip
        >
        > If you are iterating over many systems rather than doing one
        > long-running calculation, consider disabling this for speed.

    `PRECISION`
    :   `default=6`

        If `MEASURE` is `EMD`, then the Earth Mover’s Distance is calculated
        with an external C++ library that a numerical optimizer to find a good
        approximation. Consequently, systems with analytically zero \(\Phi\) will
        sometimes be numerically found to have a small but non-zero amount. This
        setting controls the number of decimal places to which PyPhi will consider
        EMD calculations accurate. Values of \(\Phi\) lower than `10e-PRECISION`
        will be considered insignificant and treated as zero. The default value is
        about as accurate as the EMD computations get.

    `VALIDATE_SUBSYSTEM_STATES`
    :   `default=True`

        Controls whether PyPhi checks if the subsystems’s state is possible
        (reachable with nonzero probability from some previous state), given the
        subsystem’s TPM (**which is conditioned on background conditions**). If
        this is turned off, then **calculated** \(\Phi\) **values may not be
        valid**, since they may be associated with a subsystem that could never be
        in the given state.

    `VALIDATE_CONDITIONAL_INDEPENDENCE`
    :   `default=True`

        Controls whether PyPhi checks if a system’s TPM is conditionally
        independent.

    `SINGLE_MICRO_NODES_WITH_SELFLOOPS_HAVE_PHI`
    :   `default=False`

        If set to `True`, the \(\Phi\) value of single micro-node subsystems is
        the difference between their unpartitioned `CauseEffectStructure` (a single
        concept) and the null concept. If set to False, their \(\Phi\) is defined
        to be zero. Single macro-node subsystems may always be cut, regardless of
        circumstances.

    `REPR_VERBOSITY`
    :   `default=2`, `values=[0, 1, 2]`

        Controls the verbosity of `__repr__` methods on PyPhi objects. Can be set
        to `0`, `1`, or `2`. If set to `1`, calling `repr` on PyPhi
        objects will return pretty-formatted and legible strings, excluding
        repertoires. If set to `2`, `repr` calls also include repertoires.

        Although this breaks the convention that `__repr__` methods should return
        a representation which can reconstruct the object, readable representations
        are convenient since the Python REPL calls `repr` to represent all
        objects in the shell and PyPhi is often used interactively with the
        REPL. If set to `0`, `repr` returns more traditional object
        representations.

    `PRINT_FRACTIONS`
    :   `default=True`

        Controls whether numbers in a `repr` are printed as fractions. Numbers
        are still printed as decimals if the fraction’s denominator would be
        large. This only has an effect if `REPR_VERBOSITY > 0`.

    `PARTITION_TYPE`
    :   `default='BI'`

        Controls the type of partition used for \(\varphi\) computations.

        If set to `'BI'`, partitions will have two parts.

        If set to `'TRI'`, partitions will have three parts. In addition,
        computations will only consider partitions that strictly partition the
        mechanism the mechanism. That is, for the mechanism `(A, B)` and purview
        `(B, C, D)` the partition:

        ```
        A,B    ∅
        ─── ✕ ───
         B    C,D
        ```

        is not considered, but:

        ```
         A     B
        ─── ✕ ───
         B    C,D
        ```

        is. The following is also valid:

        ```
        A,B     ∅
        ─── ✕ ─────
         ∅    B,C,D
        ```

        In addition, this setting introduces “wedge” tripartitions of the form:

        ```
         A     B     ∅
        ─── ✕ ─── ✕ ───
         B     C     D
        ```

        where the mechanism in the third part is always empty.

        In addition, in the case of a \(\varphi\)-tie when computing a `MaximallyIrreducibleCause` or
        `MaximallyIrreducibleEffect`, The `'TRIPARTITION'` setting choses the MIP with smallest purview
        instead of the largest (which is the default).

        Finally, if set to `'ALL'`, all possible partitions will be tested.

    `PICK_SMALLEST_PURVIEW`
    :   `default=False`

        When computing a `MaximallyIrreducibleCause` or `MaximallyIrreducibleEffect`, it is possible for several MIPs to have
        the same \(\varphi\) value. If this setting is set to `True` the MIP with
        the smallest purview is chosen; otherwise, the one with largest purview is
        chosen.

    `USE_SMALL_PHI_DIFFERENCE_FOR_CES_DISTANCE`
    :   `default=False`

        If set to `True`, the distance between cause-effect structures (when
        computing a `SystemIrreducibilityAnalysis`) is calculated using the
        difference between the sum of \(\varphi\) in the cause-effect structures
        instead of the extended EMD.

    `SYSTEM_CUTS`
    :   `default='3.0_STYLE'`, `values=['3.0_STYLE', 'CONCEPT_STYLE']`

        If set to `'3.0_STYLE'`, then traditional IIT 3.0 cuts will be used when
        computing \(\Phi\). If set to `'CONCEPT_STYLE'`, then experimental
        concept-style system cuts will be used instead.

    `log`()
    :   Log current settings.

### `connectivity`¶

Functions for determining network connectivity properties.

`pyphi.connectivity.``apply_boundary_conditions_to_cm`(*external\_indices*, *cm*)¶
:   Remove connections to or from external nodes.

`pyphi.connectivity.``get_inputs_from_cm`(*index*, *cm*)¶
:   Return indices of inputs to the node with the given index.

`pyphi.connectivity.``get_outputs_from_cm`(*index*, *cm*)¶
:   Return indices of the outputs of node with the given index.

`pyphi.connectivity.``causally_significant_nodes`(*cm*)¶
:   Return indices of nodes that have both inputs and outputs.

`pyphi.connectivity.``relevant_connections`(*n*, *\_from*, *to*)¶
:   Construct a connectivity matrix.

    |  |  |
    | --- | --- |
    | Parameters: | - **n** (*int*) – The dimensions of the matrix - **\_from** (*tuple**[**int**]*) – Nodes with outgoing connections to `to` - **to** (*tuple**[**int**]*) – Nodes with incoming connections from `_from` |
    | Returns: | An \(N \times N\) connectivity matrix with the \((i,j)^{\textrm{th}}\) entry is `1` if \(i\) is in `_from` and \(j\) is in `to`, and 0 otherwise. |
    | Return type: | np.ndarray |

`pyphi.connectivity.``block_cm`(*cm*)¶
:   Return whether `cm` can be arranged as a block connectivity matrix.

    If so, the corresponding mechanism/purview is trivially reducible.
    Technically, only square matrices are “block diagonal”, but the notion of
    connectivity carries over.

    We test for block connectivity by trying to grow a block of nodes such
    that:

    - ‘source’ nodes only input to nodes in the block
    - ‘sink’ nodes only receive inputs from source nodes in the block

    For example, the following connectivity matrix represents connections from
    `nodes1 = A, B, C` to `nodes2 = D, E, F, G` (without loss of
    generality, note that `nodes1` and `nodes2` may share elements):

    ```
       D  E  F  G
    A [1, 1, 0, 0]
    B [1, 1, 0, 0]
    C [0, 0, 1, 1]
    ```

    Since nodes \(AB\) only connect to nodes \(DE\), and node \(C\) only connects to
    nodes \(FG\), the subgraph is reducible, because the cut

    ```
    A,B    C
    ─── ✕ ───
    D,E   F,G
    ```

    does not change the structure of the graph.

`pyphi.connectivity.``block_reducible`(*cm*, *nodes1*, *nodes2*)¶
:   Return whether connections from `nodes1` to `nodes2` are reducible.

    |  |  |
    | --- | --- |
    | Parameters: | - **cm** (*np.ndarray*) – The network’s connectivity matrix. - **nodes1** (*tuple**[**int**]*) – Source nodes - **nodes2** (*tuple**[**int**]*) – Sink nodes |

`pyphi.connectivity.``is_strong`(*cm*, *nodes=None*)¶
:   Return whether the connectivity matrix is strongly connected.

    Remember that a singleton graph is strongly connected.

    |  |  |
    | --- | --- |
    | Parameters: | **cm** (*np.ndarray*) – A square connectivity matrix. |
    | Keyword Arguments: | |
    |  | **nodes** (*tuple**[**int**]*) – A subset of nodes to consider. |

`pyphi.connectivity.``is_weak`(*cm*, *nodes=None*)¶
:   Return whether the connectivity matrix is weakly connected.

    |  |  |
    | --- | --- |
    | Parameters: | **cm** (*np.ndarray*) – A square connectivity matrix. |
    | Keyword Arguments: | |
    |  | **nodes** (*tuple**[**int**]*) – A subset of nodes to consider. |

`pyphi.connectivity.``is_full`(*cm*, *nodes1*, *nodes2*)¶
:   Test connectivity of one set of nodes to another.

    |  |  |
    | --- | --- |
    | Parameters: | - **cm** (`np.ndarrray`) – The connectivity matrix - **nodes1** (*tuple**[**int**]*) – The nodes whose outputs to `nodes2` will be   tested. - **nodes2** (*tuple**[**int**]*) – The nodes whose inputs from `nodes1` will   be tested. |
    | Returns: | `True` if all elements in `nodes1` output to some element in `nodes2` and all elements in `nodes2` have an input from some element in `nodes1`, or if either set of nodes is empty; `False` otherwise. |
    | Return type: | bool |

### `constants`¶

Package-wide constants.

`pyphi.constants.``EPSILON` *= 1e-06*¶
:   The threshold below which we consider differences in phi values to be zero.

`pyphi.constants.``FILESYSTEM` *= 'fs'*¶
:   Label for the filesystem cache backend.

`pyphi.constants.``DATABASE` *= 'db'*¶
:   Label for the MongoDB cache backend.

`pyphi.constants.``PICKLE_PROTOCOL` *= 4*¶
:   The protocol used for pickling objects.

`pyphi.constants.``joblib_memory` *= Memory(cachedir='\_\_pyphi\_cache\_\_/joblib')*¶
:   The joblib `Memory` object for persistent caching without a database.

`pyphi.constants.``OFF` *= (0,)*¶
:   Node states

### `convert`¶

Conversion functions.

See the documentation on PyPhi Transition probability matrix conventions for information on the
different representations that these functions convert between.

`pyphi.convert.``reverse_bits`(*i*, *n*)¶
:   Reverse the bits of the `n`-bit decimal number `i`.

    Examples

    ```
    >>> reverse_bits(12, 7)
    24
    >>> reverse_bits(0, 1)
    0
    >>> reverse_bits(1, 2)
    2
    ```

`pyphi.convert.``nodes2indices`(*nodes*)¶
:   Convert nodes to a tuple of their indices.

`pyphi.convert.``nodes2state`(*nodes*)¶
:   Convert nodes to a tuple of their states.

`pyphi.convert.``be2le`(*i*, *n*)¶
:   Convert between big-endian and little-endian for indices in
    `range(n)`.

`pyphi.convert.``le2be`(*i*, *n*)¶
:   Convert between big-endian and little-endian for indices in
    `range(n)`.

`pyphi.convert.``state2be_index`(*state*)¶
:   Convert a PyPhi state-tuple to a decimal index according to the
    big-endian convention.

    |  |  |
    | --- | --- |
    | Parameters: | **state** (*tuple**[**int**]*) – A state-tuple where the \(i^{\textrm{th}}\) element of the tuple gives the state of the \(i^{\textrm{th}}\) node. |
    | Returns: | A decimal integer corresponding to a network state under the big-endian convention. |
    | Return type: | int |

    Examples

    ```
    >>> state2be_index((1, 0, 0, 0, 0))
    16
    >>> state2be_index((1, 1, 1, 0, 0, 0, 0, 0))
    224
    ```

`pyphi.convert.``state2le_index`(*state*)¶
:   Convert a PyPhi state-tuple to a decimal index according to the
    little-endian convention.

    |  |  |
    | --- | --- |
    | Parameters: | **state** (*tuple**[**int**]*) – A state-tuple where the \(i^{\textrm{th}}\) element of the tuple gives the state of the \(i^{\textrm{th}}\) node. |
    | Returns: | A decimal integer corresponding to a network state under the little-endian convention. |
    | Return type: | int |

    Examples

    ```
    >>> state2le_index((1, 0, 0, 0, 0))
    1
    >>> state2le_index((1, 1, 1, 0, 0, 0, 0, 0))
    7
    ```

`pyphi.convert.``le_index2state`(*i*, *number\_of\_nodes*)¶
:   Convert a decimal integer to a PyPhi state tuple with the little-endian
    convention.

    The output is the reverse of `be_index2state()`.

    |  |  |
    | --- | --- |
    | Parameters: | **i** (*int*) – A decimal integer corresponding to a network state under the little-endian convention. |
    | Returns: | A state-tuple where the \(i^{\textrm{th}}\) element of the tuple gives the state of the \(i^{\textrm{th}}\) node. |
    | Return type: | tuple[int] |

    Examples

    ```
    >>> number_of_nodes = 5
    >>> le_index2state(1, number_of_nodes)
    (1, 0, 0, 0, 0)
    >>> number_of_nodes = 8
    >>> le_index2state(7, number_of_nodes)
    (1, 1, 1, 0, 0, 0, 0, 0)
    ```

`pyphi.convert.``be_index2state`(*i*, *number\_of\_nodes*)¶
:   Convert a decimal integer to a PyPhi state tuple using the big-endian
    convention that the most-significant bits correspond to low-index nodes.

    The output is the reverse of `le_index2state()`.

    |  |  |
    | --- | --- |
    | Parameters: | **i** (*int*) – A decimal integer corresponding to a network state under the big-endian convention. |
    | Returns: | A state-tuple where the \(i^{\textrm{th}}\) element of the tuple gives the state of the \(i^{\textrm{th}}\) node. |
    | Return type: | tuple[int] |

    Examples

    ```
    >>> number_of_nodes = 5
    >>> be_index2state(1, number_of_nodes)
    (0, 0, 0, 0, 1)
    >>> number_of_nodes = 8
    >>> be_index2state(7, number_of_nodes)
    (0, 0, 0, 0, 0, 1, 1, 1)
    ```

`pyphi.convert.``be2le_state_by_state`(*tpm*)¶
:   Convert a state-by-state TPM from big-endian to little-endian or vice
    versa.

    |  |  |
    | --- | --- |
    | Parameters: | **tpm** (*np.ndarray*) – A state-by-state TPM. |
    | Returns: | The state-by-state TPM in the other indexing format. |
    | Return type: | np.ndarray |

    Example

    ```
    >>> tpm = np.arange(16).reshape([4, 4])
    >>> be2le_state_by_state(tpm)
    array([[ 0.,  1.,  2.,  3.],
           [ 8.,  9., 10., 11.],
           [ 4.,  5.,  6.,  7.],
           [12., 13., 14., 15.]])
    ```

`pyphi.convert.``le2be_state_by_state`(*tpm*)¶
:   Convert a state-by-state TPM from big-endian to little-endian or vice
    versa.

    |  |  |
    | --- | --- |
    | Parameters: | **tpm** (*np.ndarray*) – A state-by-state TPM. |
    | Returns: | The state-by-state TPM in the other indexing format. |
    | Return type: | np.ndarray |

    Example

    ```
    >>> tpm = np.arange(16).reshape([4, 4])
    >>> be2le_state_by_state(tpm)
    array([[ 0.,  1.,  2.,  3.],
           [ 8.,  9., 10., 11.],
           [ 4.,  5.,  6.,  7.],
           [12., 13., 14., 15.]])
    ```

`pyphi.convert.``to_multidimensional`(*tpm*)¶
:   Reshape a state-by-node TPM to the multidimensional form.

    See documentation for the `Network` object for more information on TPM
    formats.

`pyphi.convert.``to_2dimensional`(*tpm*)¶
:   Reshape a state-by-node TPM to the 2-dimensional form.

    See Transition probability matrix conventions and documentation for the `Network` object for
    more information on TPM representations.

`pyphi.convert.``state_by_state2state_by_node`(*tpm*)¶
:   Convert a state-by-state TPM to a state-by-node TPM.

    Danger

    Many nondeterministic state-by-state TPMs can be represented by a
    single a state-by-state TPM. However, the mapping can be made to be
    one-to-one if we assume the state-by-state TPM is conditionally
    independent, as this function does. **If the given TPM is not
    conditionally independent, the conditional dependencies will be
    silently lost.**

    Note

    The indices of the rows and columns of the state-by-state TPM are
    assumed to follow the little-endian convention. The indices of the rows
    of the resulting state-by-node TPM also follow the little-endian
    convention. See the documentation on PyPhi the Transition probability matrix conventions
    more information.

    |  |  |
    | --- | --- |
    | Parameters: | **tpm** (*list**[**list**] or* *np.ndarray*) – A square state-by-state TPM with row and column indices following the little-endian convention. |
    | Returns: | A state-by-node TPM, with row indices following the little-endian convention. |
    | Return type: | np.ndarray |

    Example

    ```
    >>> tpm = np.array([[0.5, 0.5, 0.0, 0.0],
    ...                 [0.0, 1.0, 0.0, 0.0],
    ...                 [0.0, 0.2, 0.0, 0.8],
    ...                 [0.0, 0.3, 0.7, 0.0]])
    >>> state_by_state2state_by_node(tpm)
    array([[[0.5, 0. ],
            [1. , 0.8]],

           [[1. , 0. ],
            [0.3, 0.7]]])
    ```

`pyphi.convert.``state_by_node2state_by_state`(*tpm*)¶
:   Convert a state-by-node TPM to a state-by-state TPM.

    Important

    A nondeterministic state-by-node TPM can have more than one
    representation as a state-by-state TPM. However, the mapping can be
    made to be one-to-one if we assume the TPMs to be conditionally
    independent. Therefore, **this function returns the corresponding
    conditionally independent state-by-state TPM.**

    Note

    The indices of the rows of the state-by-node TPM are assumed to follow
    the little-endian convention, while the indices of the columns follow
    the big-endian convention. The indices of the rows and columns of the
    resulting state-by-state TPM both follow the big-endian convention. See
    the documentation on PyPhi Transition probability matrix conventions for more info.

    |  |  |
    | --- | --- |
    | Parameters: | **tpm** (*list**[**list**] or* *np.ndarray*) – A state-by-node TPM with row indices following the little-endian convention and column indices following the big-endian convention. |
    | Returns: | A state-by-state TPM, with both row and column indices following the big-endian convention. |
    | Return type: | np.ndarray |

    ```
    >>> tpm = np.array([[1, 1, 0],
    ...                 [0, 0, 1],
    ...                 [0, 1, 1],
    ...                 [1, 0, 0],
    ...                 [0, 0, 1],
    ...                 [1, 0, 0],
    ...                 [1, 1, 1],
    ...                 [1, 0, 1]])
    >>> state_by_node2state_by_state(tpm)
    array([[0., 0., 0., 1., 0., 0., 0., 0.],
           [0., 0., 0., 0., 1., 0., 0., 0.],
           [0., 0., 0., 0., 0., 0., 1., 0.],
           [0., 1., 0., 0., 0., 0., 0., 0.],
           [0., 0., 0., 0., 1., 0., 0., 0.],
           [0., 1., 0., 0., 0., 0., 0., 0.],
           [0., 0., 0., 0., 0., 0., 0., 1.],
           [0., 0., 0., 0., 0., 1., 0., 0.]])
    ```

`pyphi.convert.``b2l`(*i*, *n*)¶
:   Convert between big-endian and little-endian for indices in
    `range(n)`.

`pyphi.convert.``l2b`(*i*, *n*)¶
:   Convert between big-endian and little-endian for indices in
    `range(n)`.

`pyphi.convert.``l2s`(*i*, *number\_of\_nodes*)¶
:   Convert a decimal integer to a PyPhi state tuple with the little-endian
    convention.

    The output is the reverse of `be_index2state()`.

    |  |  |
    | --- | --- |
    | Parameters: | **i** (*int*) – A decimal integer corresponding to a network state under the little-endian convention. |
    | Returns: | A state-tuple where the \(i^{\textrm{th}}\) element of the tuple gives the state of the \(i^{\textrm{th}}\) node. |
    | Return type: | tuple[int] |

    Examples

    ```
    >>> number_of_nodes = 5
    >>> le_index2state(1, number_of_nodes)
    (1, 0, 0, 0, 0)
    >>> number_of_nodes = 8
    >>> le_index2state(7, number_of_nodes)
    (1, 1, 1, 0, 0, 0, 0, 0)
    ```

`pyphi.convert.``b2s`(*i*, *number\_of\_nodes*)¶
:   Convert a decimal integer to a PyPhi state tuple using the big-endian
    convention that the most-significant bits correspond to low-index nodes.

    The output is the reverse of `le_index2state()`.

    |  |  |
    | --- | --- |
    | Parameters: | **i** (*int*) – A decimal integer corresponding to a network state under the big-endian convention. |
    | Returns: | A state-tuple where the \(i^{\textrm{th}}\) element of the tuple gives the state of the \(i^{\textrm{th}}\) node. |
    | Return type: | tuple[int] |

    Examples

    ```
    >>> number_of_nodes = 5
    >>> be_index2state(1, number_of_nodes)
    (0, 0, 0, 0, 1)
    >>> number_of_nodes = 8
    >>> be_index2state(7, number_of_nodes)
    (0, 0, 0, 0, 0, 1, 1, 1)
    ```

`pyphi.convert.``s2l`(*state*)¶
:   Convert a PyPhi state-tuple to a decimal index according to the
    little-endian convention.

    |  |  |
    | --- | --- |
    | Parameters: | **state** (*tuple**[**int**]*) – A state-tuple where the \(i^{\textrm{th}}\) element of the tuple gives the state of the \(i^{\textrm{th}}\) node. |
    | Returns: | A decimal integer corresponding to a network state under the little-endian convention. |
    | Return type: | int |

    Examples

    ```
    >>> state2le_index((1, 0, 0, 0, 0))
    1
    >>> state2le_index((1, 1, 1, 0, 0, 0, 0, 0))
    7
    ```

`pyphi.convert.``s2b`(*state*)¶
:   Convert a PyPhi state-tuple to a decimal index according to the
    big-endian convention.

    |  |  |
    | --- | --- |
    | Parameters: | **state** (*tuple**[**int**]*) – A state-tuple where the \(i^{\textrm{th}}\) element of the tuple gives the state of the \(i^{\textrm{th}}\) node. |
    | Returns: | A decimal integer corresponding to a network state under the big-endian convention. |
    | Return type: | int |

    Examples

    ```
    >>> state2be_index((1, 0, 0, 0, 0))
    16
    >>> state2be_index((1, 1, 1, 0, 0, 0, 0, 0))
    224
    ```

`pyphi.convert.``b2l_sbs`(*tpm*)¶
:   Convert a state-by-state TPM from big-endian to little-endian or vice
    versa.

    |  |  |
    | --- | --- |
    | Parameters: | **tpm** (*np.ndarray*) – A state-by-state TPM. |
    | Returns: | The state-by-state TPM in the other indexing format. |
    | Return type: | np.ndarray |

    Example

    ```
    >>> tpm = np.arange(16).reshape([4, 4])
    >>> be2le_state_by_state(tpm)
    array([[ 0.,  1.,  2.,  3.],
           [ 8.,  9., 10., 11.],
           [ 4.,  5.,  6.,  7.],
           [12., 13., 14., 15.]])
    ```

`pyphi.convert.``l2b_sbs`(*tpm*)¶
:   Convert a state-by-state TPM from big-endian to little-endian or vice
    versa.

    |  |  |
    | --- | --- |
    | Parameters: | **tpm** (*np.ndarray*) – A state-by-state TPM. |
    | Returns: | The state-by-state TPM in the other indexing format. |
    | Return type: | np.ndarray |

    Example

    ```
    >>> tpm = np.arange(16).reshape([4, 4])
    >>> be2le_state_by_state(tpm)
    array([[ 0.,  1.,  2.,  3.],
           [ 8.,  9., 10., 11.],
           [ 4.,  5.,  6.,  7.],
           [12., 13., 14., 15.]])
    ```

`pyphi.convert.``to_md`(*tpm*)¶
:   Reshape a state-by-node TPM to the multidimensional form.

    See documentation for the `Network` object for more information on TPM
    formats.

`pyphi.convert.``to_2d`(*tpm*)¶
:   Reshape a state-by-node TPM to the 2-dimensional form.

    See Transition probability matrix conventions and documentation for the `Network` object for
    more information on TPM representations.

`pyphi.convert.``sbn2sbs`(*tpm*)¶
:   Convert a state-by-node TPM to a state-by-state TPM.

    Important

    A nondeterministic state-by-node TPM can have more than one
    representation as a state-by-state TPM. However, the mapping can be
    made to be one-to-one if we assume the TPMs to be conditionally
    independent. Therefore, **this function returns the corresponding
    conditionally independent state-by-state TPM.**

    Note

    The indices of the rows of the state-by-node TPM are assumed to follow
    the little-endian convention, while the indices of the columns follow
    the big-endian convention. The indices of the rows and columns of the
    resulting state-by-state TPM both follow the big-endian convention. See
    the documentation on PyPhi Transition probability matrix conventions for more info.

    |  |  |
    | --- | --- |
    | Parameters: | **tpm** (*list**[**list**] or* *np.ndarray*) – A state-by-node TPM with row indices following the little-endian convention and column indices following the big-endian convention. |
    | Returns: | A state-by-state TPM, with both row and column indices following the big-endian convention. |
    | Return type: | np.ndarray |

    ```
    >>> tpm = np.array([[1, 1, 0],
    ...                 [0, 0, 1],
    ...                 [0, 1, 1],
    ...                 [1, 0, 0],
    ...                 [0, 0, 1],
    ...                 [1, 0, 0],
    ...                 [1, 1, 1],
    ...                 [1, 0, 1]])
    >>> state_by_node2state_by_state(tpm)
    array([[0., 0., 0., 1., 0., 0., 0., 0.],
           [0., 0., 0., 0., 1., 0., 0., 0.],
           [0., 0., 0., 0., 0., 0., 1., 0.],
           [0., 1., 0., 0., 0., 0., 0., 0.],
           [0., 0., 0., 0., 1., 0., 0., 0.],
           [0., 1., 0., 0., 0., 0., 0., 0.],
           [0., 0., 0., 0., 0., 0., 0., 1.],
           [0., 0., 0., 0., 0., 1., 0., 0.]])
    ```

`pyphi.convert.``sbs2sbn`(*tpm*)¶
:   Convert a state-by-state TPM to a state-by-node TPM.

    Danger

    Many nondeterministic state-by-state TPMs can be represented by a
    single a state-by-state TPM. However, the mapping can be made to be
    one-to-one if we assume the state-by-state TPM is conditionally
    independent, as this function does. **If the given TPM is not
    conditionally independent, the conditional dependencies will be
    silently lost.**

    Note

    The indices of the rows and columns of the state-by-state TPM are
    assumed to follow the little-endian convention. The indices of the rows
    of the resulting state-by-node TPM also follow the little-endian
    convention. See the documentation on PyPhi the Transition probability matrix conventions
    more information.

    |  |  |
    | --- | --- |
    | Parameters: | **tpm** (*list**[**list**] or* *np.ndarray*) – A square state-by-state TPM with row and column indices following the little-endian convention. |
    | Returns: | A state-by-node TPM, with row indices following the little-endian convention. |
    | Return type: | np.ndarray |

    Example

    ```
    >>> tpm = np.array([[0.5, 0.5, 0.0, 0.0],
    ...                 [0.0, 1.0, 0.0, 0.0],
    ...                 [0.0, 0.2, 0.0, 0.8],
    ...                 [0.0, 0.3, 0.7, 0.0]])
    >>> state_by_state2state_by_node(tpm)
    array([[[0.5, 0. ],
            [1. , 0.8]],

           [[1. , 0. ],
            [0.3, 0.7]]])
    ```

### `direction`¶

Causal directions.

*class* `pyphi.direction.``Direction`¶
:   Constant that parametrizes cause and effect methods.

    Accessed using `Direction.CAUSE` and `Direction.EFFECT`, etc.

    `CAUSE` *= 0*¶

    `EFFECT` *= 1*¶

    `BIDIRECTIONAL` *= 2*¶

### `distance`¶

Functions for measuring distances.

*class* `pyphi.distance.``MeasureRegistry`¶
:   Storage for measures registered with PyPhi.

    Users can define custom measures:

    Examples

    ```
    >>> @measures.register('ALWAYS_ZERO')  
    ... def always_zero(a, b):
    ...    return 0
    ```

    And use them by setting `config.MEASURE = 'ALWAYS_ZERO'`.

    `desc` *= 'measures'*¶

    `register`(*name*, *asymmetric=False*)¶
    :   Decorator for registering a measure with PyPhi.

        |  |  |
        | --- | --- |
        | Parameters: | **name** (*string*) – The name of the measure. |
        | Keyword Arguments: | |
        |  | **asymmetric** (*boolean*) – `True` if the measure is asymmetric. |

    `asymmetric`()¶
    :   Return a list of asymmetric measures.

*class* `pyphi.distance.``np_suppress`¶
:   Decorator to suppress NumPy warnings about divide-by-zero and
    multiplication of `NaN`.

    Note

    This should only be used in cases where you are *sure* that these
    warnings are not indicative of deeper issues in your code.

`pyphi.distance.``hamming_emd`(*d1*, *d2*)¶
:   Return the Earth Mover’s Distance between two distributions (indexed
    by state, one dimension per node) using the Hamming distance between states
    as the transportation cost function.

    Singleton dimensions are sqeezed out.

`pyphi.distance.``effect_emd`(*d1*, *d2*)¶
:   Compute the EMD between two effect repertoires.

    Because the nodes are independent, the EMD between effect repertoires is
    equal to the sum of the EMDs between the marginal distributions of each
    node, and the EMD between marginal distribution for a node is the absolute
    difference in the probabilities that the node is OFF.

    |  |  |
    | --- | --- |
    | Parameters: | - **d1** (*np.ndarray*) – The first repertoire. - **d2** (*np.ndarray*) – The second repertoire. |
    | Returns: | The EMD between `d1` and `d2`. |
    | Return type: | float |

`pyphi.distance.``l1`(*d1*, *d2*)¶
:   Return the L1 distance between two distributions.

    |  |  |
    | --- | --- |
    | Parameters: | - **d1** (*np.ndarray*) – The first distribution. - **d2** (*np.ndarray*) – The second distribution. |
    | Returns: | The sum of absolute differences of `d1` and `d2`. |
    | Return type: | float |

`pyphi.distance.``kld`(*d1*, *d2*)¶
:   Return the Kullback-Leibler Divergence (KLD) between two distributions.

    |  |  |
    | --- | --- |
    | Parameters: | - **d1** (*np.ndarray*) – The first distribution. - **d2** (*np.ndarray*) – The second distribution. |
    | Returns: | The KLD of `d1` from `d2`. |
    | Return type: | float |

`pyphi.distance.``entropy_difference`(*d1*, *d2*)¶
:   Return the difference in entropy between two distributions.

`pyphi.distance.``psq2`(*d1*, *d2*)¶
:   Compute the PSQ2 measure.

    |  |  |
    | --- | --- |
    | Parameters: | - **d1** (*np.ndarray*) – The first distribution. - **d2** (*np.ndarray*) – The second distribution. |

`pyphi.distance.``mp2q`(*p*, *q*)¶
:   Compute the MP2Q measure.

    |  |  |
    | --- | --- |
    | Parameters: | - **p** (*np.ndarray*) – The unpartitioned repertoire - **q** (*np.ndarray*) – The partitioned repertoire |

`pyphi.distance.``bld`(*p*, *q*)¶
:   Compute the Buzz Lightyear (Billy-Leo) Divergence.

`pyphi.distance.``directional_emd`(*direction*, *d1*, *d2*)¶
:   Compute the EMD between two repertoires for a given direction.

    The full EMD computation is used for cause repertoires. A fast analytic
    solution is used for effect repertoires.

    |  |  |
    | --- | --- |
    | Parameters: | - **direction** (*Direction*) – `CAUSE` or `EFFECT`. - **d1** (*np.ndarray*) – The first repertoire. - **d2** (*np.ndarray*) – The second repertoire. |
    | Returns: | The EMD between `d1` and `d2`, rounded to `PRECISION`. |
    | Return type: | float |
    | Raises: | `ValueError` – If `direction` is invalid. |

`pyphi.distance.``repertoire_distance`(*direction*, *r1*, *r2*)¶
:   Compute the distance between two repertoires for the given direction.

    |  |  |
    | --- | --- |
    | Parameters: | - **direction** (*Direction*) – `CAUSE` or `EFFECT`. - **r1** (*np.ndarray*) – The first repertoire. - **r2** (*np.ndarray*) – The second repertoire. |
    | Returns: | The distance between `d1` and `d2`, rounded to `PRECISION`. |
    | Return type: | float |

`pyphi.distance.``system_repertoire_distance`(*r1*, *r2*)¶
:   Compute the distance between two repertoires of a system.

    |  |  |
    | --- | --- |
    | Parameters: | - **r1** (*np.ndarray*) – The first repertoire. - **r2** (*np.ndarray*) – The second repertoire. |
    | Returns: | The distance between `r1` and `r2`. |
    | Return type: | float |

### `distribution`¶

Functions for manipulating probability distributions.

`pyphi.distribution.``normalize`(*a*)¶
:   Normalize a distribution.

    |  |  |
    | --- | --- |
    | Parameters: | **a** (*np.ndarray*) – The array to normalize. |
    | Returns: | `a` normalized so that the sum of its entries is 1. |
    | Return type: | np.ndarray |

`pyphi.distribution.``uniform_distribution`(*number\_of\_nodes*)¶
:   Return the uniform distribution for a set of binary nodes, indexed by state
    (so there is one dimension per node, the size of which is the number of
    possible states for that node).

    |  |  |
    | --- | --- |
    | Parameters: | **nodes** (*np.ndarray*) – A set of indices of binary nodes. |
    | Returns: | The uniform distribution over the set of nodes. |
    | Return type: | np.ndarray |

`pyphi.distribution.``marginal_zero`(*repertoire*, *node\_index*)¶
:   Return the marginal probability that the node is OFF.

`pyphi.distribution.``marginal`(*repertoire*, *node\_index*)¶
:   Get the marginal distribution for a node.

`pyphi.distribution.``independent`(*repertoire*)¶
:   Check whether the repertoire is independent.

`pyphi.distribution.``purview`(*repertoire*)¶
:   The purview of the repertoire.

    |  |  |
    | --- | --- |
    | Parameters: | **repertoire** (*np.ndarray*) – A repertoire |
    | Returns: | The purview that the repertoire was computed over. |
    | Return type: | tuple[int] |

`pyphi.distribution.``purview_size`(*repertoire*)¶
:   Return the size of the purview of the repertoire.

    |  |  |
    | --- | --- |
    | Parameters: | **repertoire** (*np.ndarray*) – A repertoire |
    | Returns: | The size of purview that the repertoire was computed over. |
    | Return type: | int |

`pyphi.distribution.``repertoire_shape`(*purview*, *N*)¶
:   Return the shape a repertoire.

    |  |  |
    | --- | --- |
    | Parameters: | - **purview** (*tuple**[**int**]*) – The purview over which the repertoire is   computed. - **N** (*int*) – The number of elements in the system. |
    | Returns: | The shape of the repertoire. Purview nodes have two dimensions and non-purview nodes are collapsed to a unitary dimension. |
    | Return type: | list[int] |

    Example

    ```
    >>> purview = (0, 2)
    >>> N = 3
    >>> repertoire_shape(purview, N)
    [2, 1, 2]
    ```

`pyphi.distribution.``flatten`(*repertoire*, *big\_endian=False*)¶
:   Flatten a repertoire, removing empty dimensions.

    By default, the flattened repertoire is returned in little-endian order.

    |  |  |
    | --- | --- |
    | Parameters: | **repertoire** (*np.ndarray* *or* *None*) – A repertoire. |
    | Keyword Arguments: | |
    |  | **big\_endian** (*boolean*) – If `True`, flatten the repertoire in big-endian order. |
    | Returns: | The flattened repertoire. |
    | Return type: | np.ndarray |

`pyphi.distribution.``max_entropy_distribution`(*node\_indices*, *number\_of\_nodes*)¶
:   Return the maximum entropy distribution over a set of nodes.

    This is different from the network’s uniform distribution because nodes
    outside `node_indices` are fixed and treated as if they have only 1
    state.

    |  |  |
    | --- | --- |
    | Parameters: | - **node\_indices** (*tuple**[**int**]*) – The set of node indices over which to take   the distribution. - **number\_of\_nodes** (*int*) – The total number of nodes in the network. |
    | Returns: | The maximum entropy distribution over the set of nodes. |
    | Return type: | np.ndarray |

### `examples`¶

Example networks and subsystems to go along with the documentation.

`pyphi.examples.``basic_network`(*cm=False*)¶
:   A 3-node network of logic gates.

    Diagram:

    ```
            +~~~~~~~~+
      +~~~~>|   A    |<~~~~+
      |     |  (OR)  +~~~+ |
      |     +~~~~~~~~+   | |
      |                  | |
      |                  v |
    +~+~~~~~~+       +~~~~~+~+
    |   B    |<~~~~~~+   C   |
    | (COPY) +~~~~~~>| (XOR) |
    +~~~~~~~~+       +~~~~~~~+
    ```

    TPM:

    | Previous state | Current state |
    | --- | --- |
    | A, B, C | A, B, C |
    | 0, 0, 0 | 0, 0, 0 |
    | 1, 0, 0 | 0, 0, 1 |
    | 0, 1, 0 | 1, 0, 1 |
    | 1, 1, 0 | 1, 0, 0 |
    | 0, 0, 1 | 1, 1, 0 |
    | 1, 0, 1 | 1, 1, 1 |
    | 0, 1, 1 | 1, 1, 1 |
    | 1, 1, 1 | 1, 1, 0 |

    Connectivity matrix:

    |  |  |  |  |
    | --- | --- | --- | --- |
    | . | A | B | C |
    | A | 0 | 0 | 1 |
    | B | 1 | 0 | 1 |
    | C | 1 | 1 | 0 |

    Note

    \([CM]\_{i,j} = 1\) means that there is a directed edge \((i,j)\) from node
    \(i\) to node \(j\) and \([CM]\_{i,j} = 0\) means there is no edge from \(i\) to
    \(j\).

`pyphi.examples.``basic_state`()¶
:   The state of nodes in `basic_network()`.

`pyphi.examples.``basic_subsystem`()¶
:   A subsystem containing all the nodes of the
    `basic_network()`.

`pyphi.examples.``basic_noisy_selfloop_network`()¶
:   Based on the basic\_network, but with added selfloops and noisy edges.

    Nodes perform deterministic functions of their inputs, but those inputs
    may be flipped (i.e. what should be a 0 becomes a 1, and vice versa) with
    probability epsilon (eps = 0.1 here).

    Diagram:

    ```
                 +~~+
                 |  v
              +~~~~~~~~+
        +~~~~>|   A    |<~~~~+
        |     |  (OR)  +~~~+ |
        |     +~~~~~~~~+   | |
        |                  | |
        |                  v |
      +~+~~~~~~+       +~~~~~+~+
      |   B    |<~~~~~~+   C   |
    +>| (COPY) +~~~~~~>| (XOR) |<+
    | +~~~~~~~~+       +~~~~~~~+ |
    |   |                    |   |
    +~~~+                    +~~~+
    ```

`pyphi.examples.``basic_noisy_selfloop_subsystem`()¶
:   A subsystem containing all the nodes of the
    `basic_noisy_selfloop_network()`.

`pyphi.examples.``residue_network`()¶
:   The network for the residue example.

    Current and previous state are all nodes OFF.

    Diagram:

    ```
            +~~~~~~~+         +~~~~~~~+
            |   A   |         |   B   |
        +~~>| (AND) |         | (AND) |<~~+
        |   +~~~~~~~+         +~~~~~~~+   |
        |        ^               ^        |
        |        |               |        |
        |        +~~~~~+   +~~~~~+        |
        |              |   |              |
    +~~~+~~~+        +~+~~~+~+        +~~~+~~~+
    |   C   |        |   D   |        |   E   |
    |       |        |       |        |       |
    +~~~~~~~+        +~~~~~~~+        +~~~~~~~+
    ```

    Connectivity matrix:

    |  |  |  |  |  |  |
    | --- | --- | --- | --- | --- | --- |
    | . | A | B | C | D | E |
    | A | 0 | 0 | 0 | 0 | 0 |
    | B | 0 | 0 | 0 | 0 | 0 |
    | C | 1 | 0 | 0 | 0 | 0 |
    | D | 1 | 1 | 0 | 0 | 0 |
    | E | 0 | 1 | 0 | 0 | 0 |

`pyphi.examples.``residue_subsystem`()¶
:   The subsystem containing all the nodes of the
    `residue_network()`.

`pyphi.examples.``xor_network`()¶
:   A fully connected system of three XOR gates. In the state `(0, 0, 0)`,
    none of the elementary mechanisms exist.

    Diagram:

    ```
    +~~~~~~~+       +~~~~~~~+
    |   A   +<~~~~~~+   B   |
    | (XOR) +~~~~~~>| (XOR) |
    +~+~~~~~+       +~~~~~+~+
      | ^               ^ |
      | |   +~~~~~~~+   | |
      | +~~~+   C   +~~~+ |
      +~~~~>| (XOR) +<~~~~+
            +~~~~~~~+
    ```

    Connectivity matrix:

    |  |  |  |  |
    | --- | --- | --- | --- |
    | . | A | B | C |
    | A | 0 | 1 | 1 |
    | B | 1 | 0 | 1 |
    | C | 1 | 1 | 0 |

`pyphi.examples.``xor_subsystem`()¶
:   The subsystem containing all the nodes of the
    `xor_network()`.

`pyphi.examples.``cond_depend_tpm`()¶
:   A system of two general logic gates A and B such if they are in the same
    state they stay the same, but if they are in different states, they flip
    with probability 50%.

    Diagram:

    ```
    +~~~~~+         +~~~~~+
    |  A  |<~~~~~~~~+  B  |
    |     +~~~~~~~~>|     |
    +~~~~~+         +~~~~~+
    ```

    TPM:

    |  |  |  |  |  |
    | --- | --- | --- | --- | --- |
    |  | (0, 0) | (1, 0) | (0, 1) | (1, 1) |
    | (0, 0) | 1.0 | 0.0 | 0.0 | 0.0 |
    | (1, 0) | 0.0 | 0.5 | 0.5 | 0.0 |
    | (0, 1) | 0.0 | 0.5 | 0.5 | 0.0 |
    | (1, 1) | 0.0 | 0.0 | 0.0 | 1.0 |

    Connectivity matrix:

    |  |  |  |
    | --- | --- | --- |
    | . | A | B |
    | A | 0 | 1 |
    | B | 1 | 0 |

`pyphi.examples.``cond_independ_tpm`()¶
:   A system of three general logic gates A, B and C such that: if A and B
    are in the same state then they stay the same; if they are in different
    states, they flip if C is ON and stay the same if C is OFF; and C is ON 50%
    of the time, independent of the previous state.

    Diagram:

    ```
    +~~~~~+         +~~~~~+
    |  A  +~~~~~~~~>|  B  |
    |     |<~~~~~~~~+     |
    +~+~~~+         +~~~+~+
      | ^             ^ |
      | |   +~~~~~+   | |
      | ~~~~+  C  +~~~+ |
      +~~~~>|     |<~~~~+
            +~~~~~+
    ```

    TPM:

    |  |  |  |  |  |  |  |  |  |
    | --- | --- | --- | --- | --- | --- | --- | --- | --- |
    |  | (0, 0, 0) | (1, 0, 0) | (0, 1, 0) | (1, 1, 0) | (0, 0, 1) | (1, 0, 1) | (0, 1, 1) | (1, 1, 1) |
    | (0, 0, 0) | 0.5 | 0.0 | 0.0 | 0.0 | 0.5 | 0.0 | 0.0 | 0.0 |
    | (1, 0, 0) | 0.0 | 0.5 | 0.0 | 0.0 | 0.0 | 0.5 | 0.0 | 0.0 |
    | (0, 1, 0) | 0.0 | 0.0 | 0.5 | 0.0 | 0.0 | 0.0 | 0.5 | 0.0 |
    | (1, 1, 0) | 0.0 | 0.0 | 0.0 | 0.5 | 0.0 | 0.0 | 0.0 | 0.5 |
    | (0, 0, 1) | 0.5 | 0.0 | 0.0 | 0.0 | 0.5 | 0.0 | 0.0 | 0.0 |
    | (1, 0, 1) | 0.0 | 0.0 | 0.5 | 0.0 | 0.0 | 0.0 | 0.5 | 0.0 |
    | (0, 1, 1) | 0.0 | 0.5 | 0.0 | 0.0 | 0.0 | 0.5 | 0.0 | 0.0 |
    | (1, 1, 1) | 0.0 | 0.0 | 0.0 | 0.5 | 0.0 | 0.0 | 0.0 | 0.5 |

    Connectivity matrix:

    |  |  |  |  |
    | --- | --- | --- | --- |
    | . | A | B | C |
    | A | 0 | 1 | 0 |
    | B | 1 | 0 | 0 |
    | C | 1 | 1 | 0 |

`pyphi.examples.``propagation_delay_network`()¶
:   A version of the primary example from the IIT 3.0 paper with
    deterministic COPY gates on each connection. These copy gates essentially
    function as propagation delays on the signal between OR, AND and XOR gates
    from the original system.

    The current and previous states of the network are also selected to mimic
    the corresponding states from the IIT 3.0 paper.

    Diagram:

    ```
                               +----------+
            +------------------+ C (COPY) +<----------------+
            v                  +----------+                 |
    +-------+-+                                           +-+-------+
    |         |                +----------+               |         |
    | A (OR)  +--------------->+ B (COPY) +-------------->+ D (XOR) |
    |         |                +----------+               |         |
    +-+-----+-+                                           +-+-----+-+
      |     ^                                               ^     |
      |     |                                               |     |
      |     |   +----------+                 +----------+   |     |
      |     +---+ H (COPY) +<----+     +---->+ F (COPY) +---+     |
      |         +----------+     |     |     +----------+         |
      |                          |     |                          |
      |                        +-+-----+-+                        |
      |         +----------+   |         |   +----------+         |
      +-------->+ I (COPY) +-->| G (AND) |<--+ E (COPY) +<--------+
                +----------+   |         |   +----------+
                               +---------+
    ```

    Connectivity matrix:

    |  |  |  |  |  |  |  |  |  |  |
    | --- | --- | --- | --- | --- | --- | --- | --- | --- | --- |
    | . | A | B | C | D | E | F | G | H | I |
    | A | 0 | 1 | 0 | 0 | 0 | 0 | 0 | 0 | 1 |
    | B | 0 | 0 | 0 | 1 | 0 | 0 | 0 | 0 | 0 |
    | C | 1 | 0 | 0 | 0 | 0 | 0 | 0 | 0 | 0 |
    | D | 0 | 0 | 1 | 0 | 1 | 0 | 0 | 0 | 0 |
    | E | 0 | 0 | 0 | 0 | 0 | 0 | 1 | 0 | 0 |
    | F | 0 | 0 | 0 | 1 | 0 | 0 | 0 | 0 | 0 |
    | G | 0 | 0 | 0 | 0 | 0 | 1 | 0 | 1 | 0 |
    | H | 1 | 0 | 0 | 0 | 0 | 0 | 0 | 0 | 0 |
    | I | 0 | 0 | 0 | 0 | 0 | 0 | 1 | 0 | 0 |

    States:

    In the IIT 3.0 paper example, the previous state of the system has only the
    XOR gate ON. For the propagation delay network, this corresponds to a state
    of
    `(0, 0, 0, 1, 0, 0, 0, 0, 0)`.

    The current state of the IIT 3.0 example has only the OR gate ON. By
    advancing the propagation delay system two time steps, the current state
    `(1, 0, 0, 0, 0, 0, 0, 0, 0)` is achieved, with corresponding previous
    state `(0, 0, 1, 0, 1, 0, 0, 0, 0)`.

`pyphi.examples.``macro_network`()¶
:   A network of micro elements which has greater integrated information
    after coarse graining to a macro scale.

`pyphi.examples.``macro_subsystem`()¶
:   A subsystem containing all the nodes of
    `macro_network()`.

`pyphi.examples.``blackbox_network`()¶
:   A micro-network to demonstrate blackboxing.

    Diagram:

    ```
                            +----------+
      +-------------------->+ A (COPY) + <---------------+
      |                     +----------+                 |
      |                 +----------+                     |
      |     +-----------+ B (COPY) + <-------------+     |
      v     v           +----------+               |     |
    +-+-----+-+                                  +-+-----+-+
    |         |                                  |         |
    | C (AND) |                                  | F (AND) |
    |         |                                  |         |
    +-+-----+-+                                  +-+-----+-+
      |     |                                      ^     ^
      |     |           +----------+               |     |
      |     +---------> + D (COPY) +---------------+     |
      |                 +----------+                     |
      |                     +----------+                 |
      +-------------------> + E (COPY) +-----------------+
                            +----------+
    ```

    Connectivity Matrix:

    |  |  |  |  |  |  |  |
    | --- | --- | --- | --- | --- | --- | --- |
    | . | A | B | C | D | E | F |
    | A | 0 | 0 | 1 | 0 | 0 | 0 |
    | B | 0 | 0 | 1 | 0 | 0 | 0 |
    | C | 0 | 0 | 0 | 1 | 1 | 0 |
    | D | 0 | 0 | 0 | 0 | 0 | 1 |
    | E | 0 | 0 | 0 | 0 | 0 | 1 |
    | F | 1 | 1 | 0 | 0 | 0 | 0 |

    In the documentation example, the state is (0, 0, 0, 0, 0, 0).

`pyphi.examples.``rule110_network`()¶
:   A network of three elements which follows the logic of the Rule 110
    cellular automaton with current and previous state (0, 0, 0).

`pyphi.examples.``rule154_network`()¶
:   A network of three elements which follows the logic of the Rule 154
    cellular automaton.

`pyphi.examples.``fig1a`()¶
:   The network shown in Figure 1A of the 2014 IIT 3.0 paper.

`pyphi.examples.``fig3a`()¶
:   The network shown in Figure 3A of the 2014 IIT 3.0 paper.

`pyphi.examples.``fig3b`()¶
:   The network shown in Figure 3B of the 2014 IIT 3.0 paper.

`pyphi.examples.``fig4`()¶
:   The network shown in Figure 4 of the 2014 IIT 3.0 paper.

    Diagram:

    ```
            +~~~~~~~+
      +~~~~>|   A   |<~~~~+
      | +~~~+ (OR)  +~~~+ |
      | |   +~~~~~~~+   | |
      | |               | |
      | v               v |
    +~+~~~~~+       +~~~~~+~+
    |   B   |<~~~~~~+   C   |
    | (AND) +~~~~~~>| (XOR) |
    +~~~~~~~+       +~~~~~~~+
    ```

`pyphi.examples.``fig5a`()¶
:   The network shown in Figure 5A of the 2014 IIT 3.0 paper.

    Diagram:

    ```
             +~~~~~~~+
       +~~~~>|   A   |<~~~~+
       |     | (AND) |     |
       |     +~~~~~~~+     |
       |                   |
    +~~+~~~~~+       +~~~~~+~~+
    |    B   |<~~~~~~+   C    |
    | (COPY) +~~~~~~>| (COPY) |
    +~~~~~~~~+       +~~~~~~~~+
    ```

`pyphi.examples.``fig5b`()¶
:   The network shown in Figure 5B of the 2014 IIT 3.0 paper.

    Diagram:

    ```
             +~~~~~~~+
        +~~~~+   A   +~~~~+
        |    | (AND) |    |
        |    +~~~~~~~+    |
        v                 v
    +~~~~~~~~+       +~~~~~~~~+
    |    B   |<~~~~~~+   C    |
    | (COPY) +~~~~~~>| (COPY) |
    +~~~~~~~~+       +~~~~~~~~+
    ```

`pyphi.examples.``fig6`()¶
:   The network shown in Figure 4 of the 2014 IIT 3.0 paper.

    Diagram:

    ```
            +~~~~~~~+
      +~~~~>|   A   |<~~~~+
      | +~~~+ (OR)  +~~~+ |
      | |   +~~~~~~~+   | |
      | |               | |
      | v               v |
    +~+~~~~~+       +~~~~~+~+
    |   B   |<~~~~~~+   C   |
    | (AND) +~~~~~~>| (XOR) |
    +~~~~~~~+       +~~~~~~~+
    ```

`pyphi.examples.``fig8`()¶
:   The network shown in Figure 4 of the 2014 IIT 3.0 paper.

    Diagram:

    ```
            +~~~~~~~+
      +~~~~>|   A   |<~~~~+
      | +~~~+ (OR)  +~~~+ |
      | |   +~~~~~~~+   | |
      | |               | |
      | v               v |
    +~+~~~~~+       +~~~~~+~+
    |   B   |<~~~~~~+   C   |
    | (AND) +~~~~~~>| (XOR) |
    +~~~~~~~+       +~~~~~~~+
    ```

`pyphi.examples.``fig9`()¶
:   The network shown in Figure 4 of the 2014 IIT 3.0 paper.

    Diagram:

    ```
            +~~~~~~~+
      +~~~~>|   A   |<~~~~+
      | +~~~+ (OR)  +~~~+ |
      | |   +~~~~~~~+   | |
      | |               | |
      | v               v |
    +~+~~~~~+       +~~~~~+~+
    |   B   |<~~~~~~+   C   |
    | (AND) +~~~~~~>| (XOR) |
    +~~~~~~~+       +~~~~~~~+
    ```

`pyphi.examples.``fig10`()¶
:   The network shown in Figure 4 of the 2014 IIT 3.0 paper.

    Diagram:

    ```
            +~~~~~~~+
      +~~~~>|   A   |<~~~~+
      | +~~~+ (OR)  +~~~+ |
      | |   +~~~~~~~+   | |
      | |               | |
      | v               v |
    +~+~~~~~+       +~~~~~+~+
    |   B   |<~~~~~~+   C   |
    | (AND) +~~~~~~>| (XOR) |
    +~~~~~~~+       +~~~~~~~+
    ```

`pyphi.examples.``fig14`()¶
:   The network shown in Figure 1A of the 2014 IIT 3.0 paper.

`pyphi.examples.``fig16`()¶
:   The network shown in Figure 5B of the 2014 IIT 3.0 paper.

`pyphi.examples.``actual_causation`()¶
:   The actual causation example network, consisting of an `OR` and
    `AND` gate with self-loops.

`pyphi.examples.``disjunction_conjunction_network`()¶
:   The disjunction-conjunction example from Actual Causation Figure 7.

    A network of four elements, one output `D` with three inputs `A B C`.
    The output turns ON if `A` AND `B` are ON or if `C` is ON.

`pyphi.examples.``prevention`()¶
:   The `Transition` for the prevention example from Actual Causation
    Figure 5D.

### `exceptions`¶

PyPhi exceptions.

*exception* `pyphi.exceptions.``StateUnreachableError`(*state*)¶
:   The current state cannot be reached from any previous state.

*exception* `pyphi.exceptions.``ConditionallyDependentError`¶
:   The TPM is conditionally dependent.

*exception* `pyphi.exceptions.``JSONVersionError`¶
:   JSON was serialized with a different version of PyPhi.

*exception* `pyphi.exceptions.``WrongDirectionError`¶
:   The wrong direction was provided.

### `jsonify`¶

PyPhi- and NumPy-aware JSON serialization.

To be properly serialized and deserialized, PyPhi objects must implement a
`to_json` method which returns a dictionary of attribute names and attribute
values. These attributes should be the names of arguments passed to the object
constructor. If the constructor takes additional, fewer, or different
arguments, the object needs to implement a custom `classmethod` called
`from_json` that takes a Python dictionary as an argument and returns a PyPhi
object. For example:

```
class Phi:
    def __init__(self, phi):
        self.phi = phi

    def to_json(self):
        return {'phi': self.phi, 'twice_phi': 2 * self.phi}

    @classmethod
    def from_json(cls, json):
        return Phi(json['phi'])
```

The object must also be added to `jsonify._loadable_models`.

The JSON encoder adds the name of the object and the current PyPhi version to
the JSON stream. The JSON decoder uses this metadata to recursively deserialize
the stream to a nested PyPhi object structure. The decoder will raise an
exception if current PyPhi version doesn’t match the version in the JSON data.

`pyphi.jsonify.``jsonify`(*obj*)¶
:   Return a JSON-encodable representation of an object, recursively using
    any available `to_json` methods, converting NumPy arrays and datatypes to
    native lists and types along the way.

*class* `pyphi.jsonify.``PyPhiJSONEncoder`(*skipkeys=False*, *ensure\_ascii=True*, *check\_circular=True*, *allow\_nan=True*, *sort\_keys=False*, *indent=None*, *separators=None*, *default=None*)¶
:   JSONEncoder that allows serializing PyPhi objects with `jsonify`.

    Constructor for JSONEncoder, with sensible defaults.

    If skipkeys is false, then it is a TypeError to attempt
    encoding of keys that are not str, int, float or None. If
    skipkeys is True, such items are simply skipped.

    If ensure\_ascii is true, the output is guaranteed to be str
    objects with all incoming non-ASCII characters escaped. If
    ensure\_ascii is false, the output can contain non-ASCII characters.

    If check\_circular is true, then lists, dicts, and custom encoded
    objects will be checked for circular references during encoding to
    prevent an infinite recursion (which would cause an OverflowError).
    Otherwise, no such check takes place.

    If allow\_nan is true, then NaN, Infinity, and -Infinity will be
    encoded as such. This behavior is not JSON specification compliant,
    but is consistent with most JavaScript based encoders and decoders.
    Otherwise, it will be a ValueError to encode such floats.

    If sort\_keys is true, then the output of dictionaries will be
    sorted by key; this is useful for regression tests to ensure
    that JSON serializations can be compared on a day-to-day basis.

    If indent is a non-negative integer, then JSON array
    elements and object members will be pretty-printed with that
    indent level. An indent level of 0 will only insert newlines.
    None is the most compact representation.

    If specified, separators should be an (item\_separator, key\_separator)
    tuple. The default is (‘, ‘, ‘: ‘) if *indent* is `None` and
    (‘,’, ‘: ‘) otherwise. To get the most compact JSON representation,
    you should specify (‘,’, ‘:’) to eliminate whitespace.

    If specified, default is a function that gets called for objects
    that can’t otherwise be serialized. It should return a JSON encodable
    version of the object or raise a `TypeError`.

    `encode`(*obj*)¶
    :   Encode the output of `jsonify` with the default encoder.

    `iterencode`(*obj*, *\*\*kwargs*)¶
    :   Analog to encode used by json.dump.

`pyphi.jsonify.``dumps`(*obj*, *\*\*user\_kwargs*)¶
:   Serialize `obj` as JSON-formatted stream.

`pyphi.jsonify.``dump`(*obj*, *fp*, *\*\*user\_kwargs*)¶
:   Serialize `obj` as a JSON-formatted stream and write to `fp` (a
    `.write()`-supporting file-like object.

*class* `pyphi.jsonify.``PyPhiJSONDecoder`(*\*args*, *\*\*kwargs*)¶
:   Extension of the default encoder which automatically deserializes
    PyPhi JSON to the appropriate model classes.

`pyphi.jsonify.``loads`(*string*)¶
:   Deserialize a JSON string to a Python object.

`pyphi.jsonify.``load`(*fp*)¶
:   Deserialize a JSON stream to a Python object.

### `macro`¶

Methods for coarse-graining systems to different levels of spatial analysis.

`pyphi.macro.``reindex`(*indices*)¶
:   Generate a new set of node indices, the size of indices.

`pyphi.macro.``rebuild_system_tpm`(*node\_tpms*)¶
:   Reconstruct the network TPM from a collection of node TPMs.

`pyphi.macro.``remove_singleton_dimensions`(*tpm*)¶
:   Remove singleton dimensions from the TPM.

    Singleton dimensions are created by conditioning on a set of elements.
    This removes those elements from the TPM, leaving a TPM that only
    describes the non-conditioned elements.

    Note that indices used in the original TPM must be reindexed for the
    smaller TPM.

`pyphi.macro.``run_tpm`(*system*, *steps*, *blackbox*)¶
:   Iterate the TPM for the given number of timesteps.

    |  |  |
    | --- | --- |
    | Returns: | tpm \* (noise\_tpm^(t-1)) |
    | Return type: | np.ndarray |

*class* `pyphi.macro.``SystemAttrs`¶
:   An immutable container that holds all the attributes of a subsystem.

    Versions of this object are passed down the steps of the micro-to-macro
    pipeline.

    Create new instance of SystemAttrs(tpm, cm, node\_indices, state)

    `node_labels`¶
    :   Return the labels for macro nodes.

    `nodes`¶

    *static* `pack`(*system*)¶

    `apply`(*system*)¶

*class* `pyphi.macro.``MacroSubsystem`(*network*, *state*, *nodes=None*, *cut=None*, *mice\_cache=None*, *time\_scale=1*, *blackbox=None*, *coarse\_grain=None*)¶
:   A subclass of `Subsystem` implementing macro computations.

    This subsystem performs blackboxing and coarse-graining of elements.

    Unlike `Subsystem`, whose TPM has dimensionality equal to that of the
    subsystem’s network and represents nodes external to the system using
    singleton dimensions, `MacroSubsystem` squeezes the TPM to remove these
    singletons. As a result, the node indices of the system are also squeezed
    to `0..n` so they properly index the TPM, and the state-tuple is
    reduced to the size of the system.

    After each macro update (temporal blackboxing, spatial blackboxing, and
    spatial coarse-graining) the TPM, CM, nodes, and state are updated so that
    they correctly represent the updated system.

    `cut_indices`¶
    :   The indices of this system to be cut for \(\Phi\) computations.

        For macro computations the cut is applied to the underlying
        micro-system.

    `cut_mechanisms`¶
    :   The mechanisms of this system that are currently cut.

        Note that although `cut_indices` returns micro indices, this
        returns macro mechanisms.

        |  |  |
        | --- | --- |
        | Yields: | tuple[int] |

    `cut_node_labels`¶
    :   Labels for the nodes that can be cut.

        These are the labels of the micro elements.

    `apply_cut`(*cut*)¶
    :   Return a cut version of this `MacroSubsystem`.

        |  |  |
        | --- | --- |
        | Parameters: | **cut** (*Cut*) – The cut to apply to this `MacroSubsystem`. |
        | Returns: | The cut version of this `MacroSubsystem`. |
        | Return type: | MacroSubsystem |

    `potential_purviews`(*direction*, *mechanism*, *purviews=False*)¶
    :   Override Subsystem implementation using Network-level indices.

    `macro2micro`(*macro\_indices*)¶
    :   Return all micro indices which compose the elements specified by
        `macro_indices`.

    `macro2blackbox_outputs`(*macro\_indices*)¶
    :   Given a set of macro elements, return the blackbox output elements
        which compose these elements.

    `__eq__`(*other*)¶
    :   Two macro systems are equal if each underlying `Subsystem` is equal
        and all macro attributes are equal.

*class* `pyphi.macro.``CoarseGrain`¶
:   Represents a coarse graining of a collection of nodes.

    `partition`¶
    :   *tuple[tuple]* – The partition of micro-elements into
        macro-elements.

    `grouping`¶
    :   *tuple[tuple[tuple]]* – The grouping of micro-states into
        macro-states.

    Create new instance of CoarseGrain(partition, grouping)

    `micro_indices`¶
    :   Indices of micro elements represented in this coarse-graining.

    `macro_indices`¶
    :   Indices of macro elements of this coarse-graining.

    `reindex`()¶
    :   Re-index this coarse graining to use squeezed indices.

        The output grouping is translated to use indices `0..n`, where `n`
        is the number of micro indices in the coarse-graining. Re-indexing does
        not effect the state grouping, which is already index-independent.

        |  |  |
        | --- | --- |
        | Returns: | A new `CoarseGrain` object, indexed from `0..n`. |
        | Return type: | CoarseGrain |

        Example

        ```
        >>> partition = ((1, 2),)
        >>> grouping = (((0,), (1, 2)),)
        >>> coarse_grain = CoarseGrain(partition, grouping)
        >>> coarse_grain.reindex()
        CoarseGrain(partition=((0, 1),), grouping=(((0,), (1, 2)),))
        ```

    `macro_state`(*micro\_state*)¶
    :   Translate a micro state to a macro state

        |  |  |
        | --- | --- |
        | Parameters: | **micro\_state** (*tuple**[**int**]*) – The state of the micro nodes in this coarse-graining. |
        | Returns: | The state of the macro system, translated as specified by this coarse-graining. |
        | Return type: | tuple[int] |

        Example

        ```
        >>> coarse_grain = CoarseGrain(((1, 2),), (((0,), (1, 2)),))
        >>> coarse_grain.macro_state((0, 0))
        (0,)
        >>> coarse_grain.macro_state((1, 0))
        (1,)
        >>> coarse_grain.macro_state((1, 1))
        (1,)
        ```

    `make_mapping`()¶
    :   Return a mapping from micro-state to the macro-states based on the
        partition and state grouping of this coarse-grain.

        |  |  |
        | --- | --- |
        | Returns: | A mapping from micro-states to macro-states. The \(i^{\textrm{th}}\) entry in the mapping is the macro-state corresponding to the \(i^{\textrm{th}}\) micro-state. |
        | Return type: | (nd.ndarray) |

    `macro_tpm_sbs`(*state\_by\_state\_micro\_tpm*)¶
    :   Create a state-by-state coarse-grained macro TPM.

        |  |  |
        | --- | --- |
        | Parameters: | **micro\_tpm** (*nd.array*) – The state-by-state TPM of the micro-system. |
        | Returns: | The state-by-state TPM of the macro-system. |
        | Return type: | np.ndarray |

    `macro_tpm`(*micro\_tpm*, *check\_independence=True*)¶
    :   Create a coarse-grained macro TPM.

        |  |  |
        | --- | --- |
        | Parameters: | - **micro\_tpm** (*nd.array*) – The TPM of the micro-system. - **check\_independence** (*bool*) – Whether to check that the macro TPM is   conditionally independent. |
        | Raises: | `ConditionallyDependentError` – If `check_independence` is `True` and the macro TPM is not conditionally independent. |
        | Returns: | The state-by-node TPM of the macro-system. |
        | Return type: | np.ndarray |

*class* `pyphi.macro.``Blackbox`¶
:   Class representing a blackboxing of a system.

    `partition`¶
    :   *tuple[tuple[int]]* – The partition of nodes into boxes.

    `output_indices`¶
    :   *tuple[int]* – Outputs of the blackboxes.

    Create new instance of Blackbox(partition, output\_indices)

    `hidden_indices`¶
    :   All elements hidden inside the blackboxes.

    `micro_indices`¶
    :   Indices of micro-elements in this blackboxing.

    `macro_indices`¶
    :   Fresh indices of macro-elements of the blackboxing.

    `outputs_of`(*partition\_index*)¶
    :   The outputs of the partition at `partition_index`.

        Note that this returns a tuple of element indices, since coarse-
        grained blackboxes may have multiple outputs.

    `reindex`()¶
    :   Squeeze the indices of this blackboxing to `0..n`.

        |  |  |
        | --- | --- |
        | Returns: | a new, reindexed `Blackbox`. |
        | Return type: | Blackbox |

        Example

        ```
        >>> partition = ((3,), (2, 4))
        >>> output_indices = (2, 3)
        >>> blackbox = Blackbox(partition, output_indices)
        >>> blackbox.reindex()
        Blackbox(partition=((1,), (0, 2)), output_indices=(0, 1))
        ```

    `macro_state`(*micro\_state*)¶
    :   Compute the macro-state of this blackbox.

        This is just the state of the blackbox’s output indices.

        |  |  |
        | --- | --- |
        | Parameters: | **micro\_state** (*tuple**[**int**]*) – The state of the micro-elements in the blackbox. |
        | Returns: | The state of the output indices. |
        | Return type: | tuple[int] |

    `in_same_box`(*a*, *b*)¶
    :   Return `True` if nodes `a` and `` b` `` are in the same box.

    `hidden_from`(*a*, *b*)¶
    :   Return True if `a` is hidden in a different box than `b`.

`pyphi.macro.``all_partitions`(*indices*)¶
:   Return a list of all possible coarse grains of a network.

    |  |  |
    | --- | --- |
    | Parameters: | **indices** (*tuple**[**int**]*) – The micro indices to partition. |
    | Yields: | *tuple[tuple]* – A possible partition. Each element of the tuple is a tuple of micro-elements which correspond to macro-elements. |

`pyphi.macro.``all_groupings`(*partition*)¶
:   Return all possible groupings of states for a particular coarse graining
    (partition) of a network.

    |  |  |
    | --- | --- |
    | Parameters: | **partition** (*tuple**[**tuple**]*) – A partition of micro-elements into macro elements. |
    | Yields: | *tuple[tuple[tuple]]* – A grouping of micro-states into macro states of system. |

    TODO: document exactly how to interpret the grouping.

`pyphi.macro.``all_coarse_grains`(*indices*)¶
:   Generator over all possible `CoarseGrain` of these indices.

    |  |  |
    | --- | --- |
    | Parameters: | **indices** (*tuple**[**int**]*) – Node indices to coarse grain. |
    | Yields: | *CoarseGrain* – The next `CoarseGrain` for `indices`. |

`pyphi.macro.``all_coarse_grains_for_blackbox`(*blackbox*)¶
:   Generator over all `CoarseGrain` for the given blackbox.

    If a box has multiple outputs, those outputs are partitioned into the same
    coarse-grain macro-element.

`pyphi.macro.``all_blackboxes`(*indices*)¶
:   Generator over all possible blackboxings of these indices.

    |  |  |
    | --- | --- |
    | Parameters: | **indices** (*tuple**[**int**]*) – Nodes to blackbox. |
    | Yields: | *Blackbox* – The next `Blackbox` of `indices`. |

*class* `pyphi.macro.``MacroNetwork`(*network*, *system*, *macro\_phi*, *micro\_phi*, *coarse\_grain*, *time\_scale=1*, *blackbox=None*)¶
:   A coarse-grained network of nodes.

    See the Emergence (coarse-graining and blackboxing) example in the documentation for more
    information.

    `network`¶
    :   *Network* – The network object of the macro-system.

    `phi`¶
    :   *float* – The \(\Phi\) of the network’s major complex.

    `micro_network`¶
    :   *Network* – The network object of the corresponding micro
        system.

    `micro_phi`¶
    :   *float* – The \(\Phi\) of the major complex of the
        corresponding micro-system.

    `coarse_grain`¶
    :   *CoarseGrain* – The coarse-graining of micro-elements
        into macro-elements.

    `time_scale`¶
    :   *int* – The time scale the macro-network run over.

    `blackbox`¶
    :   *Blackbox* – The blackboxing of micro elements in the network.

    `emergence`¶
    :   *float* – The difference between the \(\Phi\) of the macro-
        and the micro-system.

    `emergence`
    :   Difference between the \(\Phi\) of the macro and micro systems

`pyphi.macro.``coarse_graining`(*network*, *state*, *internal\_indices*)¶
:   Find the maximal coarse-graining of a micro-system.

    |  |  |
    | --- | --- |
    | Parameters: | - **network** (*Network*) – The network in question. - **state** (*tuple**[**int**]*) – The state of the network. - **internal\_indices** (*tuple**[**int**]*) – Nodes in the micro-system. |
    | Returns: | The phi-value of the maximal `CoarseGrain`. |
    | Return type: | tuple[int, CoarseGrain] |

`pyphi.macro.``all_macro_systems`(*network*, *state*, *do\_blackbox=False*, *do\_coarse\_grain=False*, *time\_scales=None*)¶
:   Generator over all possible macro-systems for the network.

`pyphi.macro.``emergence`(*network*, *state*, *do\_blackbox=False*, *do\_coarse\_grain=True*, *time\_scales=None*)¶
:   Check for the emergence of a micro-system into a macro-system.

    Checks all possible blackboxings and coarse-grainings of a system to find
    the spatial scale with maximum integrated information.

    Use the `do_blackbox` and `do_coarse_grain` args to specifiy whether to
    use blackboxing, coarse-graining, or both. The default is to just
    coarse-grain the system.

    |  |  |
    | --- | --- |
    | Parameters: | - **network** (*Network*) – The network of the micro-system under investigation. - **state** (*tuple**[**int**]*) – The state of the network. - **do\_blackbox** (*bool*) – Set to `True` to enable blackboxing. Defaults to   `False`. - **do\_coarse\_grain** (*bool*) – Set to `True` to enable coarse-graining.   Defaults to `True`. - **time\_scales** (*list**[**int**]*) – List of all time steps over which to check   for emergence. |
    | Returns: | The maximal macro-system generated from the micro-system. |
    | Return type: | MacroNetwork |

`pyphi.macro.``phi_by_grain`(*network*, *state*)¶

`pyphi.macro.``effective_info`(*network*)¶
:   Return the effective information of the given network.

    Note

    For details, see:

    Hoel, Erik P., Larissa Albantakis, and Giulio Tononi.
    “Quantifying causal emergence shows that macro can beat micro.”
    Proceedings of the
    National Academy of Sciences 110.49 (2013): 19790-19795.

    Available online: doi: 10.1073/pnas.1314922110.

### `models`¶

See `pyphi.models.subsystem`, `pyphi.models.mechanism`, and `pyphi.models.cuts` for
documentation.

`pyphi.models.``Account`¶
:   Alias for `pyphi.models.actual_causation.Account`.

`pyphi.models.``AcRepertoireIrreducibilityAnalysis`¶
:   Alias for
    `pyphi.models.actual_causation.AcRepertoireIrreducibilityAnalysis`.

`pyphi.models.``AcSystemIrreducibilityAnalysis`¶
:   Alias for
    `pyphi.models.actual_causation.AcSystemIrreducibilityAnalysis`.

`pyphi.models.``ActualCut`¶
:   Alias for `pyphi.models.cuts.ActualCut`.

`pyphi.models.``Bipartition`¶
:   Alias for `pyphi.models.cuts.Bipartition`.

`pyphi.models.``CausalLink`¶
:   Alias for `pyphi.models.actual_causation.CausalLink`.

`pyphi.models.``CauseEffectStructure`¶
:   Alias for
    `pyphi.models.subsystem.CauseEffectStructure`.

`pyphi.models.``Concept`¶
:   Alias for `pyphi.models.mechanism.Concept`.

`pyphi.models.``Cut`¶
:   Alias for `pyphi.models.cuts.Cut`.

`pyphi.models.``DirectedAccount`¶
:   Alias for
    `pyphi.models.actual_causation.DirectedAccount`.

`pyphi.models.``MaximallyIrreducibleCause`¶
:   Alias for
    `pyphi.models.mechanism.MaximallyIrreducibleCause`.

`pyphi.models.``MaximallyIrreducibleEffect`¶
:   Alias for
    `pyphi.models.mechanism.MaximallyIrreducibleEffect`.

`pyphi.models.``MaximallyIrreducibleCauseOrEffect`¶
:   Alias for
    `pyphi.models.mechanism.MaximallyIrreducibleCauseOrEffect`.

`pyphi.models.``Part`¶
:   Alias for `pyphi.models.cuts.Part`.

`pyphi.models.``RepertoireIrreducibilityAnalysis`¶
:   Alias for
    `pyphi.models.mechanism.RepertoireIrreducibilityAnalysis`.

`pyphi.models.``SystemIrreducibilityAnalysis`¶
:   Alias for
    `pyphi.models.subsystem.SystemIrreducibilityAnalysis`.

### `models.actual_causation`¶

Objects that represent structures used in actual causation.

`pyphi.models.actual_causation.``greater_than_zero`(*alpha*)¶
:   Return `True` if alpha is greater than zero, accounting for
    numerical errors.

*class* `pyphi.models.actual_causation.``AcRepertoireIrreducibilityAnalysis`(*alpha*, *state*, *direction*, *mechanism*, *purview*, *partition*, *probability*, *partitioned\_probability*, *node\_labels=None*)¶
:   A minimum information partition for ac\_coef calculation.

    These can be compared with the built-in Python comparison operators (`<`,
    `>`, etc.). First, \(\alpha\) values are compared. Then, if these are equal
    up to `PRECISION`, the size of the mechanism is compared.

    `alpha`¶
    :   *float* – This is the difference between the mechanism’s unpartitioned and
        partitioned actual probability.

    `state`¶
    :   *tuple[int]* – state of system in specified direction (cause, effect)

    `direction`¶
    :   *str* – The temporal direction specifiying whether this analysis should be
        calculated with cause or effect repertoires.

    `mechanism`¶
    :   *tuple[int]* – The mechanism to analyze.

    `purview`¶
    :   *tuple[int]* – The purview over which the unpartitioned actual probability differs
        the least from the actual probability of the partition.

    `partition`¶
    :   *tuple[Part, Part]* – The partition that makes the least difference to the mechanism’s
        repertoire.

    `probability`¶
    :   *float* – The probability of the state in the previous/next timestep.

    `partitioned_probability`¶
    :   *float* – The probability of the state in the partitioned repertoire.

    `unorderable_unless_eq` *= ['direction']*¶

    `order_by`()¶
    :   Return a list of values to compare for ordering.

        The first value in the list has the greatest priority; if the first
        objects are equal the second object is compared, etc.

    `__bool__`()¶
    :   An `AcRepertoireIrreducibilityAnalysis` is `True` if it has
        \(\alpha > 0\).

    `phi`¶
    :   Alias for \(\alpha\) for PyPhi utility functions.

    `to_json`()¶
    :   Return a JSON-serializable representation.

*class* `pyphi.models.actual_causation.``CausalLink`(*ria*)¶
:   A maximally irreducible actual cause or effect.

    These can be compared with the built-in Python comparison operators (`<`,
    `>`, etc.). First, \(\alpha\) values are compared. Then, if these are equal
    up to `PRECISION`, the size of the mechanism is compared.

    `alpha`¶
    :   *float* – The difference between the mechanism’s unpartitioned and
        partitioned actual probabilities.

    `phi`¶
    :   Alias for \(\alpha\) for PyPhi utility functions.

    `direction`¶
    :   *Direction* – Either `CAUSE` or `EFFECT`.

    `mechanism`¶
    :   *list[int]* – The mechanism for which the action is evaluated.

    `purview`¶
    :   *list[int]* – The purview over which this mechanism’s \(\alpha\) is
        maximal.

    `ria`¶
    :   *AcRepertoireIrreducibilityAnalysis* – The irreducibility analysis for
        this mechanism.

    `node_labels`¶

    `unorderable_unless_eq` *= ['direction']*¶

    `order_by`()¶
    :   Return a list of values to compare for ordering.

        The first value in the list has the greatest priority; if the first
        objects are equal the second object is compared, etc.

    `__bool__`()¶
    :   An `CausalLink` is `True` if \(\alpha > 0\).

    `to_json`()¶
    :   Return a JSON-serializable representation.

*class* `pyphi.models.actual_causation.``Event`¶
:   A mechanism which has both an actual cause and an actual effect.

    `actual_cause`¶
    :   *CausalLink* – The actual cause of the mechanism.

    `actual_effect`¶
    :   *CausalLink* – The actual effect of the mechanism.

    Create new instance of Event(actual\_cause, actual\_effect)

    `mechanism`¶
    :   The mechanism of the event.

*class* `pyphi.models.actual_causation.``Account`(*causal\_links*)¶
:   The set of `CausalLink` with \(\alpha > 0\). This includes both actual
    causes and actual effects.

    `irreducible_causes`¶
    :   The set of irreducible causes in this `Account`.

    `irreducible_effects`¶
    :   The set of irreducible effects in this `Account`.

    `to_json`()¶

    *classmethod* `from_json`(*dct*)¶

*class* `pyphi.models.actual_causation.``DirectedAccount`(*causal\_links*)¶
:   The set of `CausalLink` with \(\alpha > 0\) for one direction of a
    transition.

*class* `pyphi.models.actual_causation.``AcSystemIrreducibilityAnalysis`(*alpha=None*, *direction=None*, *account=None*, *partitioned\_account=None*, *transition=None*, *cut=None*)¶
:   An analysis of transition-level irreducibility (\(\mathcal{A}\)).

    Contains the \(\mathcal{A}\) value of the `Transition`, the causal account, and
    all the intermediate results obtained in the course of computing them.

    `alpha`¶
    :   *float* – The \(\mathcal{A}\) value for the transition when taken
        against this analysis, *i.e.* the difference between the
        unpartitioned account and this analysis’s partitioned account.

    `account`¶
    :   *Account* – The account of the whole transition.

    `partitioned_account`¶
    :   *Account* – The account of the partitioned
        transition.

    `transition`¶
    :   *Transition* – The transition this analysis was calculated
        for.

    `cut`¶
    :   *ActualCut* – The minimal partition.

    `before_state`¶
    :   Return the actual previous state of the `Transition`.

    `after_state`¶
    :   Return the actual current state of the `Transition`.

    `unorderable_unless_eq` *= ['direction']*¶

    `order_by`()¶
    :   Return a list of values to compare for ordering.

        The first value in the list has the greatest priority; if the first
        objects are equal the second object is compared, etc.

    `__bool__`()¶
    :   An `AcSystemIrreducibilityAnalysis` is `True` if it has
        \(\mathcal{A} > 0\).

    `to_json`()¶

### `models.cuts`¶

Objects that represent partitions of sets of nodes.

*class* `pyphi.models.cuts.``NullCut`(*indices*, *node\_labels=None*)¶
:   The cut that does nothing.

    `is_null`¶
    :   This is the only cut where `is_null == True`.

    `indices`¶
    :   Indices of the cut.

    `cut_matrix`(*n*)¶
    :   Return a matrix of zeros.

    `to_json`()¶

*class* `pyphi.models.cuts.``Cut`(*from\_nodes*, *to\_nodes*, *node\_labels=None*)¶
:   Represents a unidirectional cut.

    `from_nodes`¶
    :   *tuple[int]* – Connections from this group of nodes to those
        in `to_nodes` are from\_nodes.

    `to_nodes`¶
    :   *tuple[int]* – Connections to this group of nodes from those in
        `from_nodes` are from\_nodes.

    `from_nodes`

    `to_nodes`

    `node_labels`¶

    `indices`¶
    :   Indices of this cut.

    `cut_matrix`(*n*)¶
    :   Compute the cut matrix for this cut.

        The cut matrix is a square matrix which represents connections severed
        by the cut.

        |  |  |
        | --- | --- |
        | Parameters: | **n** (*int*) – The size of the network. |

        Example

        ```
        >>> cut = Cut((1,), (2,))
        >>> cut.cut_matrix(3)
        array([[0., 0., 0.],
               [0., 0., 1.],
               [0., 0., 0.]])
        ```

    `to_json`()¶
    :   Return a JSON-serializable representation.

*class* `pyphi.models.cuts.``KCut`(*direction*, *partition*, *node\_labels=None*)¶
:   A cut that severs all connections between parts of a K-partition.

    `indices`¶
    :   Indices of this cut.

    `cut_matrix`(*n*)¶
    :   The matrix of connections that are severed by this cut.

    `to_json`()¶

*class* `pyphi.models.cuts.``ActualCut`(*direction*, *partition*, *node\_labels=None*)¶
:   Represents an cut for a `Transition`.

    `indices`¶
    :   Indices of this cut.

*class* `pyphi.models.cuts.``Part`¶
:   Represents one part of a `Bipartition`.

    `mechanism`¶
    :   *tuple[int]* – The nodes in the mechanism for this part.

    `purview`¶
    :   *tuple[int]* – The nodes in the mechanism for this part.

    Example

    When calculating \(\varphi\) of a 3-node subsystem, we partition the
    system in the following way:

    ```
    mechanism:  A,C    B
                ─── ✕ ───
      purview:   B    A,C
    ```

    This class represents one term in the above product.

    Create new instance of Part(mechanism, purview)

    `to_json`()¶
    :   Return a JSON-serializable representation.

*class* `pyphi.models.cuts.``KPartition`(*\*parts*, *node\_labels=None*)¶
:   A partition with an arbitrary number of parts.

    `parts`¶

    `node_labels`¶

    `mechanism`¶
    :   *tuple[int]* – The nodes of the mechanism in the partition.

    `purview`¶
    :   *tuple[int]* – The nodes of the purview in the partition.

    `normalize`()¶
    :   Normalize the order of parts in the partition.

    `to_json`()¶

    *classmethod* `from_json`(*dct*)¶

*class* `pyphi.models.cuts.``Bipartition`(*\*parts*, *node\_labels=None*)¶
:   A bipartition of a mechanism and purview.

    `part0`¶
    :   *Part* – The first part of the partition.

    `part1`¶
    :   *Part* – The second part of the partition.

    `to_json`()¶
    :   Return a JSON-serializable representation.

    *classmethod* `from_json`(*dct*)¶

    `node_labels`¶

    `parts`¶

*class* `pyphi.models.cuts.``Tripartition`(*\*parts*, *node\_labels=None*)¶
:   A partition with three parts.

    `node_labels`¶

    `parts`¶

### `models.mechanism`¶

Mechanism-level objects.

*class* `pyphi.models.mechanism.``RepertoireIrreducibilityAnalysis`(*phi*, *direction*, *mechanism*, *purview*, *partition*, *repertoire*, *partitioned\_repertoire*, *node\_labels=None*)¶
:   An analysis of the irreducibility (\(\varphi\)) of a mechanism over a
    purview, for a given partition, in one temporal direction.

    These can be compared with the built-in Python comparison operators (`<`,
    `>`, etc.). First, \(\varphi\) values are compared. Then, if these are
    equal up to `PRECISION`, the size of the mechanism is compared (see the
    `PICK_SMALLEST_PURVIEW` option in `config`.)

    `phi`¶
    :   *float* – This is the difference between the mechanism’s unpartitioned
        and partitioned repertoires.

    `direction`¶
    :   *Direction* – `CAUSE` or `EFFECT`.

    `mechanism`¶
    :   *tuple[int]* – The mechanism that was analyzed.

    `purview`¶
    :   *tuple[int]* – The purview over which the the mechanism was
        analyzed.

    `partition`¶
    :   *KPartition* – The partition of the mechanism-purview pair that was
        analyzed.

    `repertoire`¶
    :   *np.ndarray* – The repertoire of the mechanism over the purview.

    `partitioned_repertoire`¶
    :   *np.ndarray* – The partitioned repertoire of the mechanism over the
        purview. This is the product of the repertoires of each part of the
        partition.

    `node_labels`¶
    :   `NodeLabels` for this system.

    `unorderable_unless_eq` *= ['direction']*¶

    `order_by`()¶
    :   Return a list of values to compare for ordering.

        The first value in the list has the greatest priority; if the first
        objects are equal the second object is compared, etc.

    `__bool__`()¶
    :   A `RepertoireIrreducibilityAnalysis` is `True` if it has
        \(\varphi > 0\).

    `to_json`()¶

*class* `pyphi.models.mechanism.``MaximallyIrreducibleCauseOrEffect`(*ria*)¶
:   A maximally irreducible cause or effect (MICE).

    These can be compared with the built-in Python comparison operators (`<`,
    `>`, etc.). First, \(\varphi\) values are compared. Then, if these are
    equal up to `PRECISION`, the size of the mechanism is compared (see the
    `PICK_SMALLEST_PURVIEW` option in `config`.)

    `phi`¶
    :   *float* – The difference between the mechanism’s unpartitioned and
        partitioned repertoires.

    `direction`¶
    :   *Direction* – `CAUSE` or `EFFECT`.

    `mechanism`¶
    :   *list[int]* – The mechanism for which the MICE is evaluated.

    `purview`¶
    :   *list[int]* – The purview over which this mechanism’s \(\varphi\) is
        maximal.

    `mip`¶
    :   *KPartition* – The partition that makes the least difference to the
        mechanism’s repertoire.

    `repertoire`¶
    :   *np.ndarray* – The unpartitioned repertoire of the mechanism over the
        purview.

    `partitioned_repertoire`¶
    :   *np.ndarray* – The partitioned repertoire of the mechanism over the
        purview.

    `ria`¶
    :   *RepertoireIrreducibilityAnalysis* – The irreducibility analysis for
        this mechanism.

    `unorderable_unless_eq` *= ['direction']*¶

    `order_by`()¶
    :   Return a list of values to compare for ordering.

        The first value in the list has the greatest priority; if the first
        objects are equal the second object is compared, etc.

    `to_json`()¶

    `damaged_by_cut`(*subsystem*)¶
    :   Return `True` if this MICE is affected by the subsystem’s cut.

        The cut affects the MICE if it either splits the MICE’s mechanism
        or splits the connections between the purview and mechanism.

*class* `pyphi.models.mechanism.``MaximallyIrreducibleCause`(*ria*)¶
:   A maximally irreducible cause (MIC).

    These can be compared with the built-in Python comparison operators (`<`,
    `>`, etc.). First, \(\varphi\) values are compared. Then, if these are
    equal up to `PRECISION`, the size of the mechanism is compared (see the
    `PICK_SMALLEST_PURVIEW` option in `config`.)

    `direction`¶
    :   *Direction* – `CAUSE`.

*class* `pyphi.models.mechanism.``MaximallyIrreducibleEffect`(*ria*)¶
:   A maximally irreducible effect (MIE).

    These can be compared with the built-in Python comparison operators (`<`,
    `>`, etc.). First, \(\varphi\) values are compared. Then, if these are
    equal up to `PRECISION`, the size of the mechanism is compared (see the
    `PICK_SMALLEST_PURVIEW` option in `config`.)

    `direction`¶
    :   *Direction* – `EFFECT`.

*class* `pyphi.models.mechanism.``Concept`(*mechanism=None*, *cause=None*, *effect=None*, *subsystem=None*, *time=None*)¶
:   The maximally irreducible cause and effect specified by a mechanism.

    These can be compared with the built-in Python comparison operators (`<`,
    `>`, etc.). First, \(\varphi\) values are compared. Then, if these are
    equal up to `PRECISION`, the size of the mechanism is compared.

    `mechanism`¶
    :   *tuple[int]* – The mechanism that the concept consists of.

    `cause`¶
    :   *MaximallyIrreducibleCause* – The `MaximallyIrreducibleCause` representing the
        maximally-irreducible cause of this concept.

    `effect`¶
    :   *MaximallyIrreducibleEffect* – The `MaximallyIrreducibleEffect` representing the
        maximally-irreducible effect of this concept.

    `subsystem`¶
    :   *Subsystem* – This concept’s parent subsystem.

    `time`¶
    :   *float* – The number of seconds it took to calculate.

    `phi`¶
    :   *float* – The size of the concept.

        This is the minimum of the \(\varphi\) values of the concept’s `MaximallyIrreducibleCause`
        and `MaximallyIrreducibleEffect`.

    `cause_purview`¶
    :   *tuple[int]* – The cause purview.

    `effect_purview`¶
    :   *tuple[int]* – The effect purview.

    `cause_repertoire`¶
    :   *np.ndarray* – The cause repertoire.

    `effect_repertoire`¶
    :   *np.ndarray* – The effect repertoire.

    `mechanism_state`¶
    :   *tuple(int)* – The state of this mechanism.

    `unorderable_unless_eq` *= ['subsystem']*¶

    `order_by`()¶
    :   Return a list of values to compare for ordering.

        The first value in the list has the greatest priority; if the first
        objects are equal the second object is compared, etc.

    `__bool__`()¶
    :   A concept is `True` if \(\varphi > 0\).

    `eq_repertoires`(*other*)¶
    :   Return whether this concept has the same repertoires as another.

        Warning

        This only checks if the cause and effect repertoires are equal as
        arrays; mechanisms, purviews, or even the nodes that the mechanism
        and purview indices refer to, might be different.

    `emd_eq`(*other*)¶
    :   Return whether this concept is equal to another in the context of
        an EMD calculation.

    `expand_cause_repertoire`(*new\_purview=None*)¶
    :   See `expand_repertoire()`.

    `expand_effect_repertoire`(*new\_purview=None*)¶
    :   See `expand_repertoire()`.

    `expand_partitioned_cause_repertoire`()¶
    :   See `expand_repertoire()`.

    `expand_partitioned_effect_repertoire`()¶
    :   See `expand_repertoire()`.

    `to_json`()¶
    :   Return a JSON-serializable representation.

    *classmethod* `from_json`(*dct*)¶

### `models.subsystem`¶

Subsystem-level objects.

*class* `pyphi.models.subsystem.``CauseEffectStructure`(*concepts=()*, *subsystem=None*, *time=None*)¶
:   A collection of concepts.

    `order_by`()¶
    :   Return a list of values to compare for ordering.

        The first value in the list has the greatest priority; if the first
        objects are equal the second object is compared, etc.

    `to_json`()¶

    `mechanisms`¶
    :   The mechanism of each concept.

    `phis`¶
    :   The \(\varphi\) values of each concept.

    `labeled_mechanisms`¶
    :   The labeled mechanism of each concept.

*class* `pyphi.models.subsystem.``SystemIrreducibilityAnalysis`(*phi=None*, *ces=None*, *partitioned\_ces=None*, *subsystem=None*, *cut\_subsystem=None*, *time=None*)¶
:   An analysis of system irreducibility (\(\Phi\)).

    Contains the \(\Phi\) value of the `Subsystem`, the cause-effect
    structure, and all the intermediate results obtained in the course of
    computing them.

    These can be compared with the built-in Python comparison operators (`<`,
    `>`, etc.). First, \(\Phi\) values are compared. Then, if these are
    equal up to `PRECISION`, the one with the larger subsystem is greater.

    `phi`¶
    :   *float* – The \(\Phi\) value for the subsystem when taken against
        this analysis, *i.e.* the difference between the cause-effect
        structure and the partitioned cause-effect structure for this
        analysis.

    `ces`¶
    :   *CauseEffectStructure* – The cause-effect structure of
        the whole subsystem.

    `partitioned_ces`¶
    :   *CauseEffectStructure* – The cause-effect structure when
        the subsystem is cut.

    `subsystem`¶
    :   *Subsystem* – The subsystem this analysis was calculated for.

    `cut_subsystem`¶
    :   *Subsystem* – The subsystem with the minimal cut applied.

    `time`¶
    :   *float* – The number of seconds it took to calculate.

    `print`(*ces=True*)¶
    :   Print this `SystemIrreducibilityAnalysis`, optionally without
        cause-effect structures.

    `small_phi_time`¶
    :   The number of seconds it took to calculate the CES.

    `cut`¶
    :   The unidirectional cut that makes the least difference to the
        subsystem.

    `network`¶
    :   The network the subsystem belongs to.

    `unorderable_unless_eq` *= ['network']*¶

    `order_by`()¶
    :   Return a list of values to compare for ordering.

        The first value in the list has the greatest priority; if the first
        objects are equal the second object is compared, etc.

    `__bool__`()¶
    :   A `SystemIrreducibilityAnalysis` is `True` if it has
        \(\Phi > 0\).

    `to_json`()¶
    :   Return a JSON-serializable representation.

    *classmethod* `from_json`(*dct*)¶

### `network`¶

Represents the network of interest. This is the primary object of PyPhi and the
context of all \(\varphi\) and \(\Phi\) computation.

*class* `pyphi.network.``Network`(*tpm*, *cm=None*, *node\_labels=None*, *purview\_cache=None*)¶
:   A network of nodes.

    Represents the network under analysis and holds auxilary data about it.

    |  |  |
    | --- | --- |
    | Parameters: | **tpm** (*np.ndarray*) – The transition probability matrix of the network.  The TPM can be provided in any of three forms: **state-by-state**, **state-by-node**, or **multidimensional state-by-node** form. In the state-by-node forms, row indices must follow the little-endian convention (see Little-endian convention). In state-by-state form, column indices must also follow the little-endian convention.  If the TPM is given in state-by-node form, it can be either 2-dimensional, so that `tpm[i]` gives the probabilities of each node being ON if the previous state is encoded by \(i\) according to the little-endian convention, or in multidimensional form, so that `tpm[(0, 0, 1)]` gives the probabilities of each node being ON if the previous state is \(N\_0 = 0, N\_1 = 0, N\_2 = 1\).  The shape of the 2-dimensional form of a state-by-node TPM must be `(s, n)`, and the shape of the multidimensional form of the TPM must be `[2] * n + [n]`, where `s` is the number of states and `n` is the number of nodes in the network. |
    | Keyword Arguments: | |
    |  | - **cm** (*np.ndarray*) – A square binary adjacency matrix indicating the   connections between nodes in the network. `cm[i][j] == 1` means   that node \(i\) is connected to node \(j\) (see Connectivity matrix conventions).   **If no connectivity matrix is given, PyPhi assumes that every node   is connected to every node (including itself)**. - **node\_labels** (tuple[str] or `NodeLabels`) – Human-readable labels for   each node in the network. |

    Example

    In a 3-node network, `the_network.tpm[(0, 0, 1)]` gives the
    transition probabilities for each node at \(t\) given that state at \(t-1\)
    was \(N\_0 = 0, N\_1 = 0, N\_2 = 1\).

    `tpm`¶
    :   *np.ndarray* – The network’s transition probability matrix, in
        multidimensional form.

    `cm`¶
    :   *np.ndarray* – The network’s connectivity matrix.

        A square binary adjacency matrix indicating the connections between
        nodes in the network.

    `connectivity_matrix`¶
    :   *np.ndarray* – Alias for `cm`.

    `causally_significant_nodes`¶
    :   See `pyphi.connectivity.causally_significant_nodes()`.

    `size`¶
    :   *int* – The number of nodes in the network.

    `num_states`¶
    :   *int* – The number of possible states of the network.

    `node_indices`¶
    :   *tuple[int]* – The indices of nodes in the network.

        This is equivalent to `tuple(range(network.size))`.

    `node_labels`¶
    :   *tuple[str]* – The labels of nodes in the network.

    `potential_purviews`(*direction*, *mechanism*)¶
    :   All purviews which are not clearly reducible for mechanism.

        |  |  |
        | --- | --- |
        | Parameters: | - **direction** (*Direction*) – `CAUSE` or `EFFECT`. - **mechanism** (*tuple**[**int**]*) – The mechanism which all purviews are   checked for reducibility over. |
        | Returns: | All purviews which are irreducible over `mechanism`. |
        | Return type: | list[tuple[int]] |

    `__len__`()¶
    :   int: The number of nodes in the network.

    `__eq__`(*other*)¶
    :   Return whether this network equals the other object.

        Networks are equal if they have the same TPM and CM.

    `to_json`()¶
    :   Return a JSON-serializable representation.

    *classmethod* `from_json`(*json\_dict*)¶
    :   Return a `Network` object from a JSON dictionary representation.

`pyphi.network.``irreducible_purviews`(*cm*, *direction*, *mechanism*, *purviews*)¶
:   Return all purviews which are irreducible for the mechanism.

    |  |  |
    | --- | --- |
    | Parameters: | - **cm** (*np.ndarray*) – An \(N \times N\) connectivity matrix. - **direction** (*Direction*) – `CAUSE` or `EFFECT`. - **purviews** (*list**[**tuple**[**int**]**]*) – The purviews to check. - **mechanism** (*tuple**[**int**]*) – The mechanism in question. |
    | Returns: | All purviews in `purviews` which are not reducible over `mechanism`. |
    | Return type: | list[tuple[int]] |
    | Raises: | `ValueError` – If `direction` is invalid. |

`pyphi.network.``from_json`(*filename*)¶
:   Convert a JSON network to a PyPhi network.

    |  |  |
    | --- | --- |
    | Parameters: | **filename** (*str*) – A path to a JSON file representing a network. |
    | Returns: | The corresponding PyPhi network object. |
    | Return type: | Network |

### `node`¶

Represents a node in a network. Each node has a unique index, its position in
the network’s list of nodes.

*class* `pyphi.node.``Node`(*tpm*, *cm*, *index*, *state*, *node\_labels*)¶
:   A node in a subsystem.

    |  |  |
    | --- | --- |
    | Parameters: | - **tpm** (*np.ndarray*) – The TPM of the subsystem. - **cm** (*np.ndarray*) – The CM of the subsystem. - **index** (*int*) – The node’s index in the network. - **state** (*int*) – The state of this node. - **node\_labels** (`NodeLabels`) – Labels for these nodes. |

    `tpm`¶
    :   *np.ndarray* – The node TPM is a 2^(n\_inputs)-by-2 matrix, where
        node.tpm[i][j] gives the marginal probability that the node is in
        state j at t+1 if the state of its inputs is i at t. If the node is
        a single element with a cut selfloop, (i.e. it has no inputs), the
        tpm is simply its unconstrained effect repertoire.

    `tpm_off`¶
    :   The TPM of this node containing only the ‘OFF’ probabilities.

    `tpm_on`¶
    :   The TPM of this node containing only the ‘ON’ probabilities.

    `inputs`¶
    :   The set of nodes with connections to this node.

    `outputs`¶
    :   The set of nodes this node has connections to.

    `label`¶
    :   The textual label for this node.

    `__eq__`(*other*)¶
    :   Return whether this node equals the other object.

        Two nodes are equal if they belong to the same subsystem and have the
        same index (their TPMs must be the same in that case, so this method
        doesn’t need to check TPM equality).

        Labels are for display only, so two equal nodes may have different
        labels.

    `to_json`()¶
    :   Return a JSON-serializable representation.

`pyphi.node.``generate_nodes`(*tpm*, *cm*, *network\_state*, *indices*, *node\_labels=None*)¶
:   Generate `Node` objects for a subsystem.

    |  |  |
    | --- | --- |
    | Parameters: | - **tpm** (*np.ndarray*) – The system’s TPM - **cm** (*np.ndarray*) – The corresponding CM. - **network\_state** (*tuple*) – The state of the network. - **indices** (*tuple**[**int**]*) – Indices to generate nodes for. |
    | Keyword Arguments: | |
    |  | **node\_labels** (`NodeLabels`) – Textual labels for each node. |
    | Returns: | The nodes of the system. |
    | Return type: | tuple[Node] |

`pyphi.node.``expand_node_tpm`(*tpm*)¶
:   Broadcast a node TPM over the full network.

    This is different from broadcasting the TPM of a full system since the last
    dimension (containing the state of the node) contains only the probability
    of *this* node being on, rather than the probabilities for each node.

### `partition`¶

Functions for generating partitions.

`pyphi.partition.``partitions`(*collection*)¶
:   Generate all set partitions of a collection.

    Example

    ```
    >>> list(partitions(range(3)))  
    [[[0, 1, 2]],
     [[0], [1, 2]],
     [[0, 1], [2]],
     [[1], [0, 2]],
     [[0], [1], [2]]]
    ```

`pyphi.partition.``bipartition_indices`(*N*)¶
:   Return indices for undirected bipartitions of a sequence.

    |  |  |
    | --- | --- |
    | Parameters: | **N** (*int*) – The length of the sequence. |
    | Returns: | A list of tuples containing the indices for each of the two parts. |
    | Return type: | list |

    Example

    ```
    >>> N = 3
    >>> bipartition_indices(N)
    [((), (0, 1, 2)), ((0,), (1, 2)), ((1,), (0, 2)), ((0, 1), (2,))]
    ```

`pyphi.partition.``bipartition`(*seq*)¶
:   Return a list of bipartitions for a sequence.

    |  |  |
    | --- | --- |
    | Parameters: | **a** (*Iterable*) – The sequence to partition. |
    | Returns: | A list of tuples containing each of the two partitions. |
    | Return type: | list[tuple[tuple]] |

    Example

    ```
    >>> bipartition((1,2,3))
    [((), (1, 2, 3)), ((1,), (2, 3)), ((2,), (1, 3)), ((1, 2), (3,))]
    ```

`pyphi.partition.``directed_bipartition_indices`(*N*)¶
:   Return indices for directed bipartitions of a sequence.

    |  |  |
    | --- | --- |
    | Parameters: | **N** (*int*) – The length of the sequence. |
    | Returns: | A list of tuples containing the indices for each of the two parts. |
    | Return type: | list |

    Example

    ```
    >>> N = 3
    >>> directed_bipartition_indices(N)  
    [((), (0, 1, 2)),
     ((0,), (1, 2)),
     ((1,), (0, 2)),
     ((0, 1), (2,)),
     ((2,), (0, 1)),
     ((0, 2), (1,)),
     ((1, 2), (0,)),
     ((0, 1, 2), ())]
    ```

`pyphi.partition.``directed_bipartition`(*seq*, *nontrivial=False*)¶
:   Return a list of directed bipartitions for a sequence.

    |  |  |
    | --- | --- |
    | Parameters: | **seq** (*Iterable*) – The sequence to partition. |
    | Returns: | A list of tuples containing each of the two parts. |
    | Return type: | list[tuple[tuple]] |

    Example

    ```
    >>> directed_bipartition((1, 2, 3))  
    [((), (1, 2, 3)),
     ((1,), (2, 3)),
     ((2,), (1, 3)),
     ((1, 2), (3,)),
     ((3,), (1, 2)),
     ((1, 3), (2,)),
     ((2, 3), (1,)),
     ((1, 2, 3), ())]
    ```

`pyphi.partition.``bipartition_of_one`(*seq*)¶
:   Generate bipartitions where one part is of length 1.

`pyphi.partition.``reverse_elements`(*seq*)¶
:   Reverse the elements of a sequence.

`pyphi.partition.``directed_bipartition_of_one`(*seq*)¶
:   Generate directed bipartitions where one part is of length 1.

    |  |  |
    | --- | --- |
    | Parameters: | **seq** (*Iterable*) – The sequence to partition. |
    | Returns: | A list of tuples containing each of the two partitions. |
    | Return type: | list[tuple[tuple]] |

    Example

    ```
    >>> partitions = directed_bipartition_of_one((1, 2, 3))
    >>> list(partitions)  
    [((1,), (2, 3)),
     ((2,), (1, 3)),
     ((3,), (1, 2)),
     ((2, 3), (1,)),
     ((1, 3), (2,)),
     ((1, 2), (3,))]
    ```

`pyphi.partition.``directed_tripartition_indices`(*N*)¶
:   Return indices for directed tripartitions of a sequence.

    |  |  |
    | --- | --- |
    | Parameters: | **N** (*int*) – The length of the sequence. |
    | Returns: | A list of tuples containing the indices for each partition. |
    | Return type: | list[tuple] |

    Example

    ```
    >>> N = 1
    >>> directed_tripartition_indices(N)
    [((0,), (), ()), ((), (0,), ()), ((), (), (0,))]
    ```

`pyphi.partition.``directed_tripartition`(*seq*)¶
:   Generator over all directed tripartitions of a sequence.

    |  |  |
    | --- | --- |
    | Parameters: | **seq** (*Iterable*) – a sequence. |
    | Yields: | *tuple[tuple]* – A tripartition of `seq`. |

    Example

    ```
    >>> seq = (2, 5)
    >>> list(directed_tripartition(seq))  
    [((2, 5), (), ()),
     ((2,), (5,), ()),
     ((2,), (), (5,)),
     ((5,), (2,), ()),
     ((), (2, 5), ()),
     ((), (2,), (5,)),
     ((5,), (), (2,)),
     ((), (5,), (2,)),
     ((), (), (2, 5))]
    ```

`pyphi.partition.``k_partitions`(*collection*, *k*)¶
:   Generate all `k`-partitions of a collection.

    Example

    ```
    >>> list(k_partitions(range(3), 2))
    [[[0, 1], [2]], [[0], [1, 2]], [[0, 2], [1]]]
    ```

*class* `pyphi.partition.``PartitionRegistry`¶
:   Storage for partition schemes registered with PyPhi.

    Users can define custom partitions:

    Examples

    ```
    >>> @partition_registry.register('NONE')  
    ... def no_partitions(mechanism, purview):
    ...    return []
    ```

    And use them by setting `config.PARTITION_TYPE = 'NONE'`

    `desc` *= 'partitions'*¶

`pyphi.partition.``mip_partitions`(*mechanism*, *purview*, *node\_labels=None*)¶
:   Return a generator over all mechanism-purview partitions, based on the
    current configuration.

`pyphi.partition.``mip_bipartitions`(*mechanism*, *purview*, *node\_labels=None*)¶
:   Return an generator of all \(\varphi\) bipartitions of a mechanism over
    a purview.

    Excludes all bipartitions where one half is entirely empty, *e.g*:

    ```
     A     ∅
    ─── ✕ ───
     B     ∅
    ```

    is not valid, but

    ```
     A     ∅
    ─── ✕ ───
     ∅     B
    ```

    is.

    |  |  |
    | --- | --- |
    | Parameters: | - **mechanism** (*tuple**[**int**]*) – The mechanism to partition - **purview** (*tuple**[**int**]*) – The purview to partition |
    | Yields: | *Bipartition* –  Where each bipartition is:  ``` bipart[0].mechanism   bipart[1].mechanism ─────────────────── ✕ ─────────────────── bipart[0].purview     bipart[1].purview ``` |

    Example

    ```
    >>> mechanism = (0,)
    >>> purview = (2, 3)
    >>> for partition in mip_bipartitions(mechanism, purview):
    ...     print(partition, '\n')  
     ∅     0
    ─── ✕ ───
     2     3

     ∅     0
    ─── ✕ ───
     3     2

     ∅     0
    ─── ✕ ───
    2,3    ∅
    ```

`pyphi.partition.``wedge_partitions`(*mechanism*, *purview*, *node\_labels=None*)¶
:   Return an iterator over all wedge partitions.

    These are partitions which strictly split the mechanism and allow a subset
    of the purview to be split into a third partition, e.g.:

    ```
     A     B     ∅
    ─── ✕ ─── ✕ ───
     B     C     D
    ```

    See `PARTITION_TYPE` in `config` for more information.

    |  |  |
    | --- | --- |
    | Parameters: | - **mechanism** (*tuple**[**int**]*) – A mechanism. - **purview** (*tuple**[**int**]*) – A purview. |
    | Yields: | *Tripartition* – all unique tripartitions of this mechanism and purview. |

`pyphi.partition.``all_partitions`(*mechanism*, *purview*, *node\_labels=None*)¶
:   Return all possible partitions of a mechanism and purview.

    Partitions can consist of any number of parts.

    |  |  |
    | --- | --- |
    | Parameters: | - **mechanism** (*tuple**[**int**]*) – A mechanism. - **purview** (*tuple**[**int**]*) – A purview. |
    | Yields: | *KPartition* – A partition of this mechanism and purview into `k` parts. |

### `subsystem`¶

Represents a candidate system for \(\varphi\) and \(\Phi\) evaluation.

*class* `pyphi.subsystem.``Subsystem`(*network*, *state*, *nodes=None*, *cut=None*, *mice\_cache=None*, *repertoire\_cache=None*, *single\_node\_repertoire\_cache=None*, *\_external\_indices=None*)¶
:   A set of nodes in a network.

    |  |  |
    | --- | --- |
    | Parameters: | - **network** (*Network*) – The network the subsystem belongs to. - **state** (*tuple**[**int**]*) – The state of the network. |
    | Keyword Arguments: | |
    |  | - **nodes** (*tuple**[**int**] or* *tuple**[**str**]*) – The nodes of the network which are in   this subsystem. Nodes can be specified either as indices or as   labels if the `Network` was passed `node_labels`. If this is   `None` then the full network will be used. - **cut** (*Cut*) – The unidirectional `Cut` to apply to this subsystem. |

    `network`¶
    :   *Network* – The network the subsystem belongs to.

    `tpm`¶
    :   *np.ndarray* – The TPM conditioned on the state of the external
        nodes.

    `cm`¶
    :   *np.ndarray* – The connectivity matrix after applying the cut.

    `state`¶
    :   *tuple[int]* – The state of the network.

    `node_indices`¶
    :   *tuple[int]* – The indices of the nodes in the subsystem.

    `cut`¶
    :   *Cut* – The cut that has been applied to this subsystem.

    `null_cut`¶
    :   *Cut* – The cut object representing no cut.

    `nodes`¶
    :   *tuple[Node]* – The nodes in this `Subsystem`.

    `proper_state`¶
    :   *tuple[int]* – The state of the subsystem.

        `proper_state[i]` gives the state of the \(i^{\textrm{th}}\) node **in the
        subsystem**. Note that this is **not** the state of `nodes[i]`.

    `connectivity_matrix`¶
    :   *np.ndarray* – Alias for `cm`.

    `size`¶
    :   *int* – The number of nodes in the subsystem.

    `is_cut`¶
    :   *bool* – `True` if this Subsystem has a cut applied to it.

    `cut_indices`¶
    :   *tuple[int]* – The nodes of this subsystem to cut for \(\Phi\)
        computations.

        This was added to support `MacroSubsystem`, which cuts indices other
        than `node_indices`.

        |  |  |
        | --- | --- |
        | Yields: | tuple[int] |

    `cut_mechanisms`¶
    :   *list[tuple[int]]* – The mechanisms that are cut in this system.

    `cut_node_labels`¶
    :   `NodeLabels` – Labels for the nodes of this system that will be
        cut.

    `tpm_size`¶
    :   *int* – The number of nodes in the TPM.

    `cache_info`()¶
    :   Report repertoire cache statistics.

    `clear_caches`()¶
    :   Clear the mice and repertoire caches.

    `__bool__`()¶
    :   Return `False` if the Subsystem has no nodes, `True`
        otherwise.

    `__eq__`(*other*)¶
    :   Return whether this Subsystem is equal to the other object.

        Two Subsystems are equal if their sets of nodes, networks, and cuts are
        equal.

    `__lt__`(*other*)¶
    :   Return whether this subsystem has fewer nodes than the other.

    `__gt__`(*other*)¶
    :   Return whether this subsystem has more nodes than the other.

    `__len__`()¶
    :   Return the number of nodes in this Subsystem.

    `to_json`()¶
    :   Return a JSON-serializable representation.

    `apply_cut`(*cut*)¶
    :   Return a cut version of this `Subsystem`.

        |  |  |
        | --- | --- |
        | Parameters: | **cut** (*Cut*) – The cut to apply to this `Subsystem`. |
        | Returns: | The cut subsystem. |
        | Return type: | Subsystem |

    `indices2nodes`(*indices*)¶
    :   Return `Node` for these indices.

        |  |  |
        | --- | --- |
        | Parameters: | **indices** (*tuple**[**int**]*) – The indices in question. |
        | Returns: | The `Node` objects corresponding to these indices. |
        | Return type: | tuple[Node] |
        | Raises: | `ValueError` – If requested indices are not in the subsystem. |

    `cause_repertoire`(*mechanism*, *purview*)¶
    :   Return the cause repertoire of a mechanism over a purview.

        |  |  |
        | --- | --- |
        | Parameters: | - **mechanism** (*tuple**[**int**]*) – The mechanism for which to calculate the   cause repertoire. - **purview** (*tuple**[**int**]*) – The purview over which to calculate the   cause repertoire. |
        | Returns: | The cause repertoire of the mechanism over the purview. |
        | Return type: | np.ndarray |

        Note

        The returned repertoire is a distribution over purview node states,
        not the states of the whole network.

    `effect_repertoire`(*mechanism*, *purview*)¶
    :   Return the effect repertoire of a mechanism over a purview.

        |  |  |
        | --- | --- |
        | Parameters: | - **mechanism** (*tuple**[**int**]*) – The mechanism for which to calculate the   effect repertoire. - **purview** (*tuple**[**int**]*) – The purview over which to calculate the   effect repertoire. |
        | Returns: | The effect repertoire of the mechanism over the purview. |
        | Return type: | np.ndarray |

        Note

        The returned repertoire is a distribution over purview node states,
        not the states of the whole network.

    `repertoire`(*direction*, *mechanism*, *purview*)¶
    :   Return the cause or effect repertoire based on a direction.

        |  |  |
        | --- | --- |
        | Parameters: | - **direction** (*Direction*) – `CAUSE` or `EFFECT`. - **mechanism** (*tuple**[**int**]*) – The mechanism for which to calculate the   repertoire. - **purview** (*tuple**[**int**]*) – The purview over which to calculate the   repertoire. |
        | Returns: | The cause or effect repertoire of the mechanism over the purview. |
        | Return type: | np.ndarray |
        | Raises: | `ValueError` – If `direction` is invalid. |

    `unconstrained_repertoire`(*direction*, *purview*)¶
    :   Return the unconstrained cause/effect repertoire over a purview.

    `unconstrained_cause_repertoire`(*purview*)¶
    :   Return the unconstrained cause repertoire for a purview.

        This is just the cause repertoire in the absence of any mechanism.

    `unconstrained_effect_repertoire`(*purview*)¶
    :   Return the unconstrained effect repertoire for a purview.

        This is just the effect repertoire in the absence of any mechanism.

    `partitioned_repertoire`(*direction*, *partition*)¶
    :   Compute the repertoire of a partitioned mechanism and purview.

    `expand_repertoire`(*direction*, *repertoire*, *new\_purview=None*)¶
    :   Distribute an effect repertoire over a larger purview.

        |  |  |
        | --- | --- |
        | Parameters: | - **direction** (*Direction*) – `CAUSE` or `EFFECT`. - **repertoire** (*np.ndarray*) – The repertoire to expand. |
        | Keyword Arguments: | |
        |  | **new\_purview** (*tuple**[**int**]*) – The new purview to expand the repertoire over. If `None` (the default), the new purview is the entire network. |
        | Returns: | A distribution over the new purview, where probability is spread out over the new nodes. |
        | Return type: | np.ndarray |
        | Raises: | `ValueError` – If the expanded purview doesn’t contain the original purview. |

    `expand_cause_repertoire`(*repertoire*, *new\_purview=None*)¶
    :   Alias for `expand_repertoire()` with `direction` set to `CAUSE`.

    `expand_effect_repertoire`(*repertoire*, *new\_purview=None*)¶
    :   Alias for `expand_repertoire()` with `direction` set to `EFFECT`.

    `cause_info`(*mechanism*, *purview*)¶
    :   Return the cause information for a mechanism over a purview.

    `effect_info`(*mechanism*, *purview*)¶
    :   Return the effect information for a mechanism over a purview.

    `cause_effect_info`(*mechanism*, *purview*)¶
    :   Return the cause-effect information for a mechanism over a purview.

        This is the minimum of the cause and effect information.

    `evaluate_partition`(*direction*, *mechanism*, *purview*, *partition*, *repertoire=None*)¶
    :   Return the \(\varphi\) of a mechanism over a purview for the given
        partition.

        |  |  |
        | --- | --- |
        | Parameters: | - **direction** (*Direction*) – `CAUSE` or `EFFECT`. - **mechanism** (*tuple**[**int**]*) – The nodes in the mechanism. - **purview** (*tuple**[**int**]*) – The nodes in the purview. - **partition** (*Bipartition*) – The partition to evaluate. |
        | Keyword Arguments: | |
        |  | **repertoire** (*np.array*) – The unpartitioned repertoire. If not supplied, it will be computed. |
        | Returns: | The distance between the unpartitioned and partitioned repertoires, and the partitioned repertoire. |
        | Return type: | tuple[int, np.ndarray] |

    `find_mip`(*direction*, *mechanism*, *purview*)¶
    :   Return the minimum information partition for a mechanism over a
        purview.

        |  |  |
        | --- | --- |
        | Parameters: | - **direction** (*Direction*) – `CAUSE` or `EFFECT`. - **mechanism** (*tuple**[**int**]*) – The nodes in the mechanism. - **purview** (*tuple**[**int**]*) – The nodes in the purview. |
        | Returns: | The irreducibility analysis for the mininum-information partition in one temporal direction. |
        | Return type: | RepertoireIrreducibilityAnalysis |

    `cause_mip`(*mechanism*, *purview*)¶
    :   Return the irreducibility analysis for the cause MIP.

        Alias for `find_mip()` with `direction` set to `CAUSE`.

    `effect_mip`(*mechanism*, *purview*)¶
    :   Return the irreducibility analysis for the effect MIP.

        Alias for `find_mip()` with `direction` set to `EFFECT`.

    `phi_cause_mip`(*mechanism*, *purview*)¶
    :   Return the \(\varphi\) of the cause MIP.

        This is the distance between the unpartitioned cause repertoire and the
        MIP cause repertoire.

    `phi_effect_mip`(*mechanism*, *purview*)¶
    :   Return the \(\varphi\) of the effect MIP.

        This is the distance between the unpartitioned effect repertoire and
        the MIP cause repertoire.

    `phi`(*mechanism*, *purview*)¶
    :   Return the \(\varphi\) of a mechanism over a purview.

    `potential_purviews`(*direction*, *mechanism*, *purviews=False*)¶
    :   Return all purviews that could belong to the `MaximallyIrreducibleCause`/`MaximallyIrreducibleEffect`.

        Filters out trivially-reducible purviews.

        |  |  |
        | --- | --- |
        | Parameters: | - **direction** (*Direction*) – `CAUSE` or `EFFECT`. - **mechanism** (*tuple**[**int**]*) – The mechanism of interest. |
        | Keyword Arguments: | |
        |  | **purviews** (*tuple**[**int**]*) – Optional subset of purviews of interest. |

    `find_mice`(*direction*, *mechanism*, *purviews=False*)¶
    :   Return the `MaximallyIrreducibleCause` or `MaximallyIrreducibleEffect` for a mechanism.

        |  |  |
        | --- | --- |
        | Parameters: | - **direction** (*Direction*) – :`CAUSE` or `EFFECT`. - **mechanism** (*tuple**[**int**]*) – The mechanism to be tested for   irreducibility. |
        | Keyword Arguments: | |
        |  | **purviews** (*tuple**[**int**]*) – Optionally restrict the possible purviews to a subset of the subsystem. This may be useful for \_e.g.\_ finding only concepts that are “about” a certain subset of nodes. |
        | Returns: | The `MaximallyIrreducibleCause` or `MaximallyIrreducibleEffect`. |
        | Return type: | MaximallyIrreducibleCauseOrEffect |

    `mic`(*mechanism*, *purviews=False*)¶
    :   Return the mechanism’s maximally-irreducible cause (`MaximallyIrreducibleCause`).

        Alias for `find_mice()` with `direction` set to `CAUSE`.

    `mie`(*mechanism*, *purviews=False*)¶
    :   Return the mechanism’s maximally-irreducible effect (`MaximallyIrreducibleEffect`).

        Alias for `find_mice()` with `direction` set to `EFFECT`.

    `phi_max`(*mechanism*)¶
    :   Return the \(\varphi^{\textrm{max}}\) of a mechanism.

        This is the maximum of \(\varphi\) taken over all possible purviews.

    `null_concept`¶
    :   Return the null concept of this subsystem.

        The null concept is a point in concept space identified with
        the unconstrained cause and effect repertoire of this subsystem.

    `concept`(*mechanism*, *purviews=False*, *cause\_purviews=False*, *effect\_purviews=False*)¶
    :   Return the concept specified by a mechanism within this subsytem.

        |  |  |
        | --- | --- |
        | Parameters: | **mechanism** (*tuple**[**int**]*) – The candidate set of nodes. |
        | Keyword Arguments: | |
        |  | - **purviews** (*tuple**[**tuple**[**int**]**]*) – Restrict the possible purviews to   those in this list. - **cause\_purviews** (*tuple**[**tuple**[**int**]**]*) – Restrict the possible cause   purviews to those in this list. Takes precedence over   `purviews`. - **effect\_purviews** (*tuple**[**tuple**[**int**]**]*) – Restrict the possible effect   purviews to those in this list. Takes precedence over   `purviews`. |
        | Returns: | The pair of maximally irreducible cause/effect repertoires that constitute the concept specified by the given mechanism. |
        | Return type: | Concept |

### `timescale`¶

Functions for converting the timescale of a TPM.

`pyphi.timescale.``sparse`(*matrix*, *threshold=0.1*)¶

`pyphi.timescale.``sparse_time`(*tpm*, *time\_scale*)¶

`pyphi.timescale.``dense_time`(*tpm*, *time\_scale*)¶

`pyphi.timescale.``run_tpm`(*tpm*, *time\_scale*)¶
:   Iterate a TPM by the specified number of time steps.

    |  |  |
    | --- | --- |
    | Parameters: | - **tpm** (*np.ndarray*) – A state-by-node tpm. - **time\_scale** (*int*) – The number of steps to run the tpm. |
    | Returns: | np.ndarray |

`pyphi.timescale.``run_cm`(*cm*, *time\_scale*)¶
:   Iterate a connectivity matrix the specified number of steps.

    |  |  |
    | --- | --- |
    | Parameters: | - **cm** (*np.ndarray*) – A connectivity matrix. - **time\_scale** (*int*) – The number of steps to run. |
    | Returns: | The connectivity matrix at the new timescale. |
    | Return type: | np.ndarray |

### `tpm`¶

Functions for manipulating transition probability matrices.

`pyphi.tpm.``tpm_indices`(*tpm*)¶
:   Return the indices of nodes in the TPM.

`pyphi.tpm.``is_state_by_state`(*tpm*)¶
:   Return `True` if `tpm` is in state-by-state form, otherwise
    `False`.

`pyphi.tpm.``condition_tpm`(*tpm*, *fixed\_nodes*, *state*)¶
:   Return a TPM conditioned on the given fixed node indices, whose states
    are fixed according to the given state-tuple.

    The dimensions of the new TPM that correspond to the fixed nodes are
    collapsed onto their state, making those dimensions singletons suitable for
    broadcasting. The number of dimensions of the conditioned TPM will be the
    same as the unconditioned TPM.

`pyphi.tpm.``expand_tpm`(*tpm*)¶
:   Broadcast a state-by-node TPM so that singleton dimensions are expanded
    over the full network.

`pyphi.tpm.``marginalize_out`(*node\_indices*, *tpm*)¶
:   Marginalize out nodes from a TPM.

    |  |  |
    | --- | --- |
    | Parameters: | - **node\_indices** (*list**[**int**]*) – The indices of nodes to be marginalized out. - **tpm** (*np.ndarray*) – The TPM to marginalize the node out of. |
    | Returns: | A TPM with the same number of dimensions, with the nodes marginalized out. |
    | Return type: | np.ndarray |

`pyphi.tpm.``infer_edge`(*tpm*, *a*, *b*, *contexts*)¶
:   Infer the presence or absence of an edge from node A to node B.

    Let \(S\) be the set of all nodes in a network. Let \(A' = S - \{A\}\). We call
    the state of \(A'\) the context \(C\) of \(A\). There is an edge from \(A\) to \(B\)
    if there exists any context \(C(A)\) such that \(\Pr(B \mid C(A), A = 0) \neq \Pr(B \mid C(A), A = 1)\).

    |  |  |
    | --- | --- |
    | Parameters: | - **tpm** (*np.ndarray*) – The TPM in state-by-node, multidimensional form. - **a** (*int*) – The index of the putative source node. - **b** (*int*) – The index of the putative sink node. |
    | Returns: | `True` if the edge \(A \rightarrow B\) exists, `False` otherwise. |
    | Return type: | bool |

`pyphi.tpm.``infer_cm`(*tpm*)¶
:   Infer the connectivity matrix associated with a state-by-node TPM in
    multidimensional form.

### `utils`¶

Functions used by more than one PyPhi module or class, or that might be of
external use.

`pyphi.utils.``state_of`(*nodes*, *network\_state*)¶
:   Return the state-tuple of the given nodes.

`pyphi.utils.``all_states`(*n*, *big\_endian=False*)¶
:   Return all binary states for a system.

    |  |  |
    | --- | --- |
    | Parameters: | - **n** (*int*) – The number of elements in the system. - **big\_endian** (*bool*) – Whether to return the states in big-endian order   instead of little-endian order. |
    | Yields: | *tuple[int]* – The next state of an `n`-element system, in little-endian order unless `big_endian` is `True`. |

`pyphi.utils.``np_immutable`(*a*)¶
:   Make a NumPy array immutable.

`pyphi.utils.``np_hash`(*a*)¶
:   Return a hash of a NumPy array.

*class* `pyphi.utils.``np_hashable`(*array*)¶
:   A hashable wrapper around a NumPy array.

`pyphi.utils.``eq`(*x*, *y*)¶
:   Compare two values up to `PRECISION`.

`pyphi.utils.``combs`(*a*, *r*)¶
:   NumPy implementation of `itertools.combinations`.

    Return successive `r`-length combinations of elements in the array `a`.

    |  |  |
    | --- | --- |
    | Parameters: | - **a** (*np.ndarray*) – The array from which to get combinations. - **r** (*int*) – The length of the combinations. |
    | Returns: | An array of combinations. |
    | Return type: | np.ndarray |

`pyphi.utils.``comb_indices`(*n*, *k*)¶
:   `n`-dimensional version of itertools.combinations.

    |  |  |
    | --- | --- |
    | Parameters: | - **a** (*np.ndarray*) – The array from which to get combinations. - **k** (*int*) – The desired length of the combinations. |
    | Returns: | Indices that give the `k`-combinations of `n` elements. |
    | Return type: | np.ndarray |

    Example

    ```
    >>> n, k = 3, 2
    >>> data = np.arange(6).reshape(2, 3)
    >>> data[:, comb_indices(n, k)]
    array([[[0, 1],
            [0, 2],
            [1, 2]],

           [[3, 4],
            [3, 5],
            [4, 5]]])
    ```

`pyphi.utils.``powerset`(*iterable*, *nonempty=False*, *reverse=False*)¶
:   Generate the power set of an iterable.

    |  |  |
    | --- | --- |
    | Parameters: | **iterable** (*Iterable*) – The iterable from which to generate the power set. |
    | Keyword Arguments: | |
    |  | - **nonempty** (*boolean*) – If True, don’t include the empty set. - **reverse** (*boolean*) – If True, reverse the order of the powerset. |
    | Returns: | An iterator over the power set. |
    | Return type: | Iterable |

    Example

    ```
    >>> ps = powerset(np.arange(2))
    >>> list(ps)
    [(), (0,), (1,), (0, 1)]
    >>> ps = powerset(np.arange(2), nonempty=True)
    >>> list(ps)
    [(0,), (1,), (0, 1)]
    >>> ps = powerset(np.arange(2), nonempty=True, reverse=True)
    >>> list(ps)
    [(1, 0), (1,), (0,)]
    ```

`pyphi.utils.``load_data`(*directory*, *num*)¶
:   Load numpy data from the data directory.

    The files should stored in `../data/<dir>` and named
    `0.npy, 1.npy, ... <num - 1>.npy`.

    |  |  |
    | --- | --- |
    | Returns: | A list of loaded data, such that `list[i]` contains the the contents of `i.npy`. |
    | Return type: | list |

`pyphi.utils.``time_annotated`(*func*, *\*args*, *\*\*kwargs*)¶
:   Annotate the decorated function or method with the total execution
    time.

    The result is annotated with a time attribute.

### `validate`¶

Methods for validating arguments.

`pyphi.validate.``direction`(*direction*, *allow\_bi=False*)¶
:   Validate that the given direction is one of the allowed constants.

    If `allow_bi` is `True` then `Direction.BIDIRECTIONAL` is
    acceptable.

`pyphi.validate.``tpm`(*tpm*, *check\_independence=True*)¶
:   Validate a TPM.

    The TPM can be in

    > - 2-dimensional state-by-state form,
    > - 2-dimensional state-by-node form, or
    > - multidimensional state-by-node form.

`pyphi.validate.``conditionally_independent`(*tpm*)¶
:   Validate that the TPM is conditionally independent.

`pyphi.validate.``connectivity_matrix`(*cm*)¶
:   Validate the given connectivity matrix.

`pyphi.validate.``node_labels`(*node\_labels*, *node\_indices*)¶
:   Validate that there is a label for each node.

`pyphi.validate.``network`(*n*)¶
:   Validate a `Network`.

    Checks the TPM and connectivity matrix.

`pyphi.validate.``is_network`(*network*)¶
:   Validate that the argument is a `Network`.

`pyphi.validate.``node_states`(*state*)¶
:   Check that the state contains only zeros and ones.

`pyphi.validate.``state_length`(*state*, *size*)¶
:   Check that the state is the given size.

`pyphi.validate.``state_reachable`(*subsystem*)¶
:   Return whether a state can be reached according to the network’s TPM.

`pyphi.validate.``cut`(*cut*, *node\_indices*)¶
:   Check that the cut is for only the given nodes.

`pyphi.validate.``subsystem`(*s*)¶
:   Validate a `Subsystem`.

    Checks its state and cut.

`pyphi.validate.``time_scale`(*time\_scale*)¶
:   Validate a macro temporal time scale.

`pyphi.validate.``partition`(*partition*)¶
:   Validate a partition - used by blackboxes and coarse grains.

`pyphi.validate.``coarse_grain`(*coarse\_grain*)¶
:   Validate a macro coarse-graining.

`pyphi.validate.``blackbox`(*blackbox*)¶
:   Validate a macro blackboxing.

`pyphi.validate.``blackbox_and_coarse_grain`(*blackbox*, *coarse\_grain*)¶
:   Validate that a coarse-graining properly combines the outputs of a
    blackboxing.

---

© Copyright 2014--2017 William GP Mayner.
Revision `d29e93a6`.

Read the Docs
v: stable

Versions
:   latest
:   stable
:   1.1.0
:   1.0.0
:   0.9.0
:   0.8.1
:   0.7.0
:   develop

Downloads
:   pdf
:   htmlzip

On Read the Docs
:   Project Home
:   Builds

---

Free document hosting provided by Read the Docs.
